# Product Selectivity in Baeyer–Villiger Monooxygenase-Catalyzed Bacterial Alkaloid Core Structure Maturation

**DOI:** 10.1021/jacs.4c04115

**Published:** 2024-06-03

**Authors:** Manuel Einsiedler, Katharina Lamm, Jonas F. Ohlrogge, Sebastian Schuler, Ivana J. Richter, Tilo Lübken, Tobias A. M. Gulder

**Affiliations:** † Helmholtz Institute for Pharmaceutical Research Saarland (HIPS), Department of Natural Product Biotechnology, Helmholtz Centre for Infection Research (HZI) and Department of Pharmacy at Saarland University, Campus E8.1, 66123 Saarbrücken, Germany; ‡ Chair of Technical Biochemistry, Technische Universität Dresden, Bergstraße 66, 01069 Dresden, Germany; § Chair of Organic Chemistry I, Technische Universität Dresden, Bergstraße 66, 01069 Dresden, Germany

## Abstract

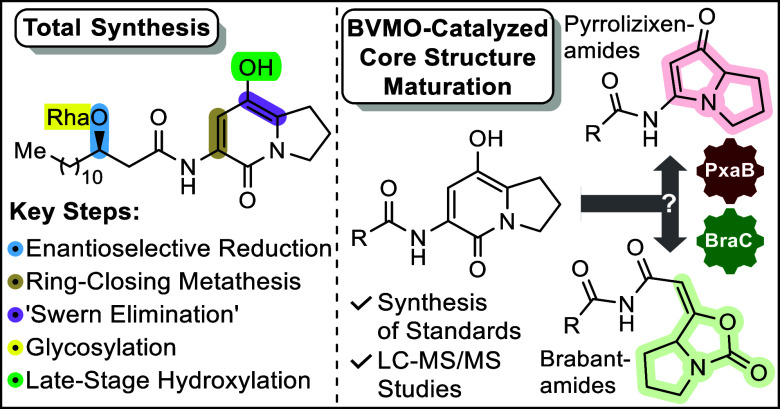

Baeyer–Villiger monooxygenases (BVMOs) play crucial roles in the core-structure modification of natural products. They catalyze lactone formation by selective oxygen insertion into a carbon–carbon bond adjacent to a carbonyl group (Baeyer–Villiger oxidation, BVO). The homologous bacterial BVMOs, BraC and PxaB, thereby process bicyclic dihydroindolizinone substrates originating from a bimodular nonribosomal peptide synthetase (BraB or PxaA). While both enzymes initially catalyze the formation of oxazepine-dione intermediates following the identical mechanism, the final natural product spectrum diverges. For the pathway involving BraC, the exclusive formation of lipocyclocarbamates, the brabantamides, was reported. The pathway utilizing PxaB solely produces pyrrolizidine alkaloids, the pyrrolizixenamides. Surprisingly, replacing *pxaB* within the pyrrolizixenamide biosynthetic pathway by *braC* does not change the product spectrum to brabantamides. Factors controlling this product selectivity have remained elusive. In this study, we set out to solve this puzzle by combining the total synthesis of crucial pathway intermediates and anticipated products with in-depth functional in vitro studies on both recombinant BVMOs. This work shows that the joint oxazepine-dione intermediate initially formed by both BVMOs leads to pyrrolizixenamides upon nonenzymatic hydrolysis, decarboxylative ring contraction, and dehydration. Brabantamide biosynthesis is enzyme-controlled, with BraC efficiently transforming all the accepted substrates into its cognate final product scaffold. PxaB, in contrast, shows only considerable activity toward brabantamide formation for the substrate analog with a natural brabantamide-type side chain structure, revealing substrate-controlled product selectivity.

## Introduction

Oxygenases are a class of essential enzymes found in all kingdoms of life that catalyze many complex transformations in primary and secondary metabolism. One special class of these enzymes are Baeyer–Villiger monooxygenases (BVMOs), which insert an oxygen atom into C–C-bonds adjacent to a carbonyl group to generate esters or lactones.^1^ The probably best-known and investigated BVMO is the cyclohexanone oxygenase from *Acinetobacter* sp. NCIB 9871, which converts its substrate into ε-caprolactone.^2^ This enzyme is very promiscuous, accepting many different substrates while retaining high regio- and stereoselectivity.^3,4^

**Figure 1:**
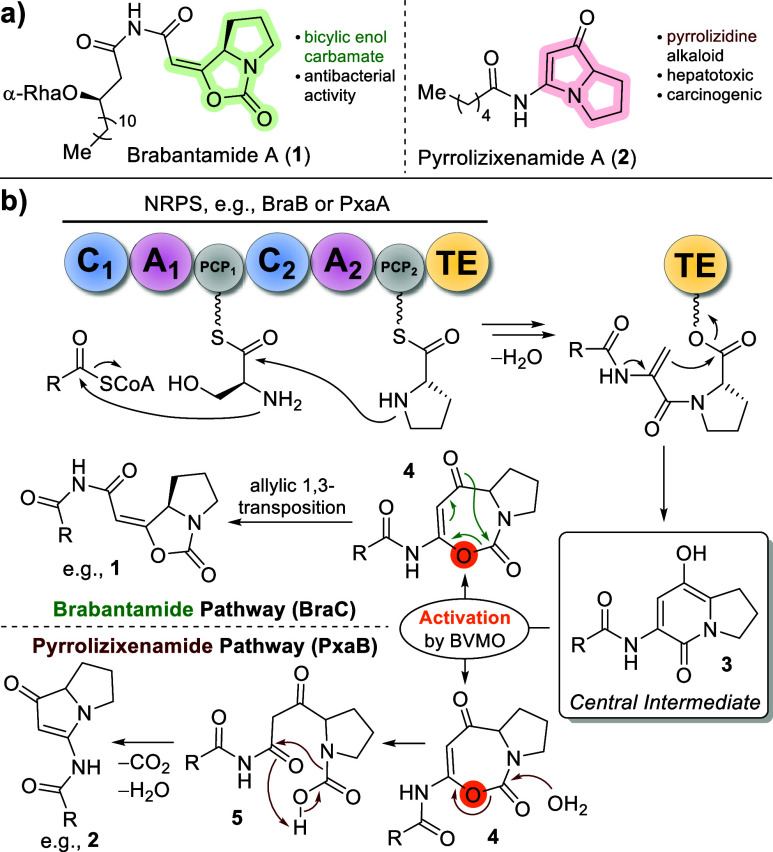
(a) Structures of brabantamide A (**1**) and pyrrolizixenamide A (**2**). (b) Proposed biosynthesis of the central biosynthetic intermediate **3** by a bimodular NRPS and tailoring by BVMOs, BraC and PxaB, via **4**, ultimately diverging toward **1** and **2**, respectively. Rha—rhamnose; C—condensation domain; A—adenylation domain; PCP—peptidyl carrier protein; TE—thioesterase.

BVMOs also play crucial roles in the biosynthesis of unusual bacterial alkaloids, such as legonmycins,^5^ brabantamides [e.g., brabantamide A (**1**)],^6−9^ and pyrrolizixenamides (e.g., pyrrolizixenamide A (**2**), Figure 1a).^10,11^ These compounds stand out due to their biological activities. The lipocyclocarbamates (LCCs) brabantamides, such as **1**, inhibit lipoprotein-associated lipoprotease (Lp-PLA_2_) that induces inflammation and thus serves as a new target for drugs against atherosclerosis.^6,7^ This finding led to the development of the synthetic drug candidate darapladib.^12,13^ In addition, **1** exhibits antibacterial activity, e.g., against *Bacillus subtilis* 168 with a MIC of 12.5 μg/mL.^14^ Metabolites such as **2** belong to the pyrrolizidine alkaloids (PAs), a widespread natural product class that mainly occurs in plants and is notorious for its high hepatotoxicity and carcinogenicity.^15^ Interestingly, the biosyntheses of **1** and **2** proceed via central intermediate **3** with an indolizin-5(1*H*)-one structure. A bimodular nonribosomal peptide synthetase (NRPS) utilizes serine, proline, and a ketoacyl building block to initially deliver an enamine bound to the NRPS thioesterase (TE), which upon cyclization and concomitant offloading provides **3** (Figure 1b).^9,10,14^

Intermediate **3** is then activated by a pathway-specific FAD-dependent BVMO, BraC or PxaB, initially resulting in the expansion of the 6-membered ring to give common intermediate **4**.^5,10,14^ Compound **4** is thought to be further processed by pathway-specific diverging mechanisms resulting in the entirely different molecular scaffolds of brabantamides (intramolecular allylic 1,3-transposition) or pyrrolizixenamides (hydrolysis/decarboxylation to **5**, followed by recyclization/dehydration). However, replacing *pxaB* with *braC* in the pyrrolizixenamide biosynthetic pathway in vivo still resulted in the exclusive formation of pyrrolizixenamides, identical to the expression of the native pathway *pxaAB*.^10^ It was thus hypothesized that additional yet unknown enzymes might be required to control product selectivity.^10^ An alternative explanation might be substrate-structure induced product control, as recently observed in AsqJ biocatalysis.^16,17^ It is important to note that the only difference between the structures of biosynthetic intermediates **4** across both pathways resides at the R group of the acyl side chain, which is not involved in the final rearrangement cascades. While for brabantamides, the R group in intermediate **4** is characterized by long chain lengths (up to 16 carbons) and a β-hydroxy function that is rhamnosylated, and the fatty acid side chains of pyrrolizixenamides are unsubstituted and relatively short (6 to 8 carbons).^18^

Taken together, the biosynthetic pathways for brabantamides and pyrrolizixenamides only diverge at the final step of the catalytic mechanism of the corresponding BVMOs (BraC and PxaB) on substrates with identical indolizinone core structure **4**, yet different side chains R (Figure 1). To shed light on the underlying mechanism of product control, we set out to explore this phenomenon by production of substrates **3** with different side chains [**a**: caproic acid (C_6_), **b**: caprylic acid (C_8_), **c**: capric acid (C_10_), **d**: myristic acid (C_14_), **e**: (*R*)-3-hydroxymyristic acid (C_14_-3-OH), and **f**: (3*R*)-α-rhamnosyloxymyristic acid (C_14_-3-ORha)] and selected final product standards allowing in-depth functional characterization of the recombinant BVMOs BraC and PxaB.

## Results and Discussion

**Scheme 1:**
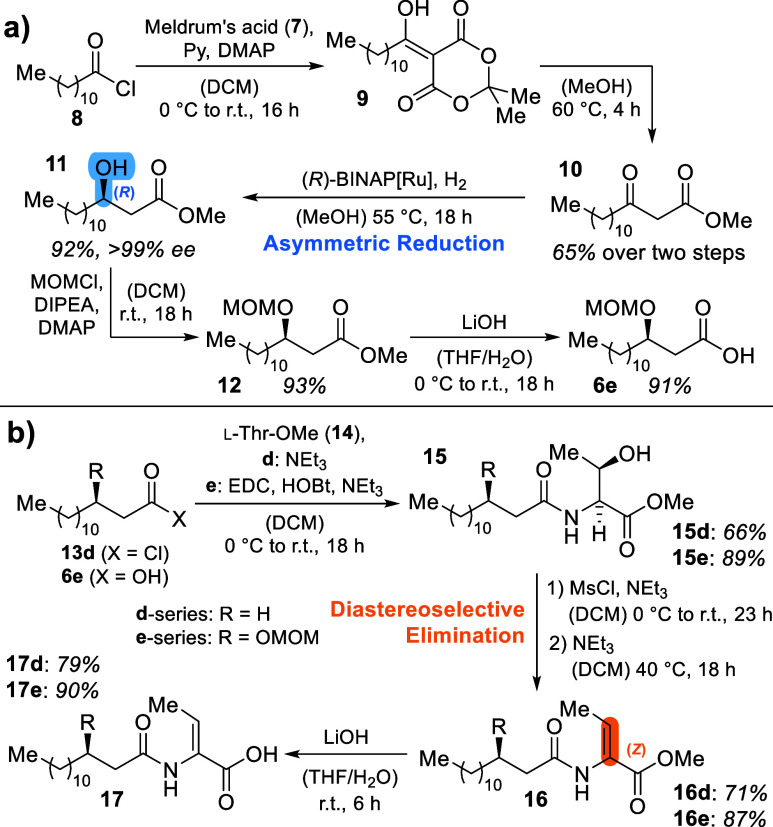
(a) Enantioselective (Blue) Synthesis of the Chiral Fatty Acid Building Block **6e**; (b) Synthesis of Western Building Blocks, α,β-Unsaturated Acids **17** The (*Z*)-olefin formed by diastereoselective elimination is labeled in orange.

We started the synthesis of the putative central brabantamide intermediates **3** with the enantioselective production of the β-hydroxy fatty acid **6e** (Scheme 1a). Acylation of Meldrum’s acid (**7**) with lauroyl chloride (**8**) in the presence of pyridine and DMAP delivered **9**, which was directly used for the next step. Heating of **9** in MeOH yielded the corresponding β-ketoester **10** in 65% yield over two steps. This compound was selectively reduced to the (*R*)-configured β-hydroxyester **11** by Noyori-hydrogenation with a BINAP-ruthenium-based catalyst in an excellent 92% yield.^19^ Enantiomeric excess (*ee*) was determined by derivatization of **11** as its (*R*)-Mosher ester **SI-1** (see Supporting Information). High-performance liquid chromatography (HPLC) analysis revealed perfect stereocontrol with an *ee* of >99%. MOM-protection of **11** by alkylation with freshly distilled MOMCl in the presence of Hünig’s base and DMAP delivered acetal **12** in 93% yield. Saponification with LiOH yielded the chiral acid **6e** in 91%.

The following steps were conducted with both **6e** and myristoyl chloride **13d** (Scheme 1b). l-threonine methyl ester (**14**) was acylated using either the corresponding commercial **13d** and NEt_3_ to yield amide **15d** (66%) or the free acid **6e** with EDC, HOBt, and NEt_3_ (**15e**, 89%). These amides were then subjected to (*Z*)-selective elimination^20^ of the hydroxy group using MsCl and NEt_3_, delivering the olefins **16d** and **16e** in 71 and 87% yield, respectively. The resulting esters were saponified by LiOH, yielding free unsaturated acids **17d** (79%) and **17e** (90%).

**Scheme 2:**
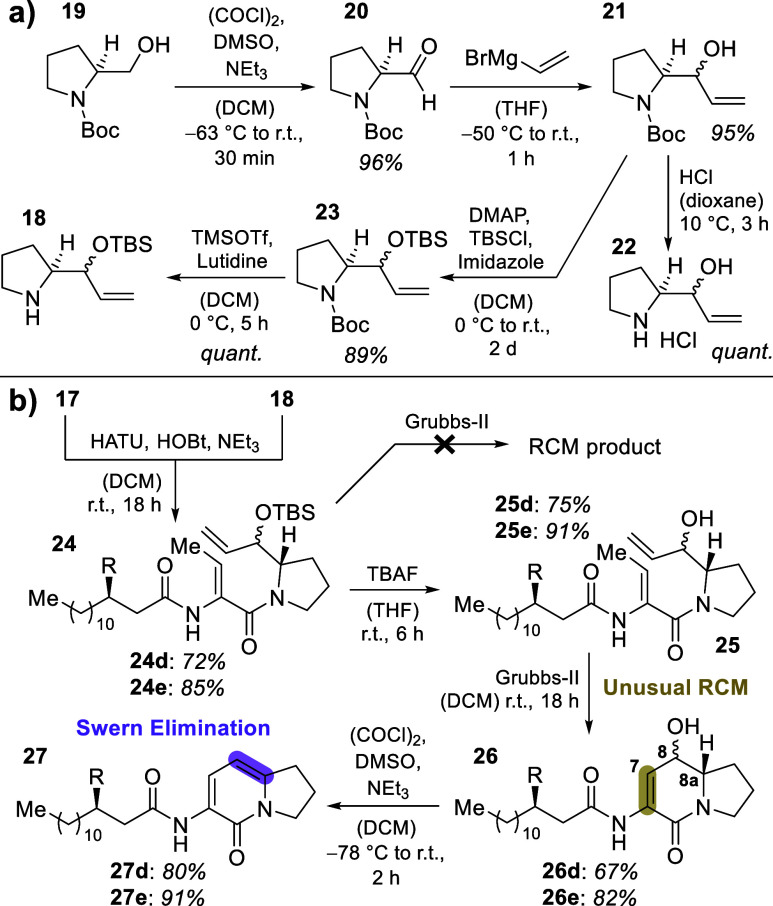
(a) Synthesis of Eastern Building Block **18**; (b) Synthesis of the [4.3.0]-Bicyclic Central Ring System **26** by RCM and Subsequent “Swern Elimination” to **27**

The synthesis of proline-derived building block **18** commenced by Swern oxidation of *N*-Boc-l-prolinol (**19**) to aldehyde **20** in 96% yield. Grignard addition of vinyl magnesium bromide to **20** delivered a mixture of diastereomers of the corresponding alcohol **21** in 95%. This step did not need stereochemical control, as the chiral center formed was to be removed again later in the synthesis (cf. Scheme 2).

For the first coupling attempts with acids **17**, compound **21** was Boc-deprotected using HCl in dioxane to yield highly polar compound **22**. An amide coupling reaction of **17d** with **22** in the presence of HATU, HOBt, and NEt_3_ was conducted in DMF due to the poor solubility of amine **22** in other solvents, resulting in tedious isolation and purification procedures. Moreover, screening different coupling reagents all resulted in low chemical yields below 40%. Therefore, we introduced a TBS-protecting group to alcohol **21**. This was achieved with TBSCl in the presence of DMAP and imidazole, yielding silyl ether **23** in 89%. Boc-deprotection with TMSOTf and lutidine^20^ delivered free amine **18** quantitatively. The decreased polarity of this compound compared with **22** allowed coupling reactions to acids **17** with HATU, HOBt, and NEt_3_ in DCM (Scheme 2b). This resulted in a more convenient reaction workup and, more importantly, drastically improved yields of the TBS-protected amides of 72% (**24d**) and 85% (**24e**).

As a test system for the planned ring-closing metathesis (RCM) on **24**, we *N*-acrylated **22** by treatment with acryloyl chloride in the presence of NEt_3_ to yield metathesis precursor **SI-3** in 52% yield (data shown in Supporting Information, chapter 3.1.6). This compound was then subjected to RCM using Grubbs-II catalyst (5 mol %) in DCM, which delivered the corresponding desired cyclic olefin in 48% yield.

Attempted RCM on **24** with a Grubbs-II catalyst showed no conversion (Scheme 2b). We assumed that the sterically demanding TBS group prevents the ruthenium carbene from addition to the terminal olefin.^21^ Therefore, TBS-deprotection of the amides was conducted first and achieved by treatment with TBAF to yield free alcohols **25d** (75%) and **25e** (91%). To our delight, the Grubbs-II catalyst was highly active for these systems, facilitating the unusual RCMs on Michael acceptor systems with excision of propene toward the bicycles **26d** and **26e** in 67 and 82% yield, respectively. The reaction worked best at concentrations of 20 to 25 mm starting material in degassed DCM and a catalyst loading between 5 and 10 mol % at room temperature over 18 h. Interestingly, especially when heated to reflux, these reactions in some cases showed the formation of an additional compound, which was identified to be the subsequently desired elimination product **27**.

We observed that one diastereoisomer of the metathesis product was prone to elimination, while the other was stable. This was often observed already during NMR analysis, as obvious from comparison of the ^1^H signals representing the protons at positions 7 and 8 of the alcohols **26** (cf. Scheme 2b). This observation was more closely inspected using analog **26a**. After preliminary computational structure optimization and estimation of dihedral angles (H–C^7^–C^8^–H and H–C^8^–C^8a^–H), we transformed these angles into ^3^*J* coupling constants (by Bothner-By function).^22,23^ By comparison of the calculated data with the experimental constants in the ^1^H NMR spectra, we conclude that the labile compound is the corresponding (8*S*,8a*S*)-stereoisomer. As the hydroxyl function at C-8 in this isomer is in the *anti*-position to the proton at C-8a, this promotes the spontaneous elimination of water following an E2 mechanism that is favored by the antiperiplanar position of the leaving group (OH) and proton in this stereoisomer (see Supporting Information, chapter 4.1 for detailed analysis and figures).

With the alcohols **26** in hands, we next aimed at oxidizing them to the desired intermediates **3**. However, different oxidation methods that are commonly used for conversions of secondary alcohols to ketones did not work for this system; for example, while Dess–Martin periodinane and TEMPO-based systems resulted in decomposition, MnO_2_-based allylic oxidations led to an overoxidation to an assumed enone, as observed by HRMS. Attempted Swern oxidation resulted in the clean formation of one single product. However, this compound was identified to be the elimination product^27^ instead of desired ketone **3**. Thus, we herein discovered a molecular system, where Swern conditions selectively lead to dehydration instead of oxidation (“Swern elimination”). To probe whether the observed reaction outcome may result from simple acid- (HCl) or base- (NEt_3_) catalyzed elimination, we investigated test reactions with substrate **26e** (cf. Supporting Information, chapter 4.2). While stirring **26e** under reflux conditions in DCM with NEt_3_ did not lead to any conversion, treatment with HCl in dioxane at 0 °C led to trace amounts of the elimination product **27e**. However, TLC and HPLC showed a complex mixture of decomposition products, thus implicating that only Swern-type conditions lead to a quantitative and selective transformation to the dehydrated product. Hence, we investigated whether the Swern-type elimination takes place directly after formation of the alkoxysulfonium ion or if the addition of a base is necessary. Therefore, we quenched a small amount of the reaction before the addition of NEt_3_ and analyzed it by HPLC, which already showed a clean formation of the elimination product. We thus hypothesize that as soon as the alcohol attacks the chlorosulfonium ion, DMSO is eliminated from the system, generating the observed olefin. The driving force is thus the formation of the highly stable S–O double bond of DMSO, together with the formation of indolizinone **27**. As these reactions were operationally simple with excellent yields (**27d**: 80%, **27e**: 91%) and the diverse attempted oxidations of alcohols **26** delivered only negative results, we integrated this newly discovered reaction type into our synthetic route. Moreover, we hypothesized that compounds **3** predominantly exist as their (ph)enol-tautomer, and this is the reason for the failed oxidation reactions described above. This assumption was corroborated by extensive NMR analysis of C_6_-intermediate (**3a**) produced in vivo by heterologous expression of the NRPS PxaA in *Escherichia coli* BAP1 or DH10β cells, followed by recombinant product isolation (cf. Supporting Information, chapters 1.2.5 and 2), where a broad singlet at 8.63 ppm (^1^H NMR) was identified to represent the corresponding OH group, bound to a carbon atom generating a ^13^C NMR signal at 133.45 ppm.

**Scheme 3:**
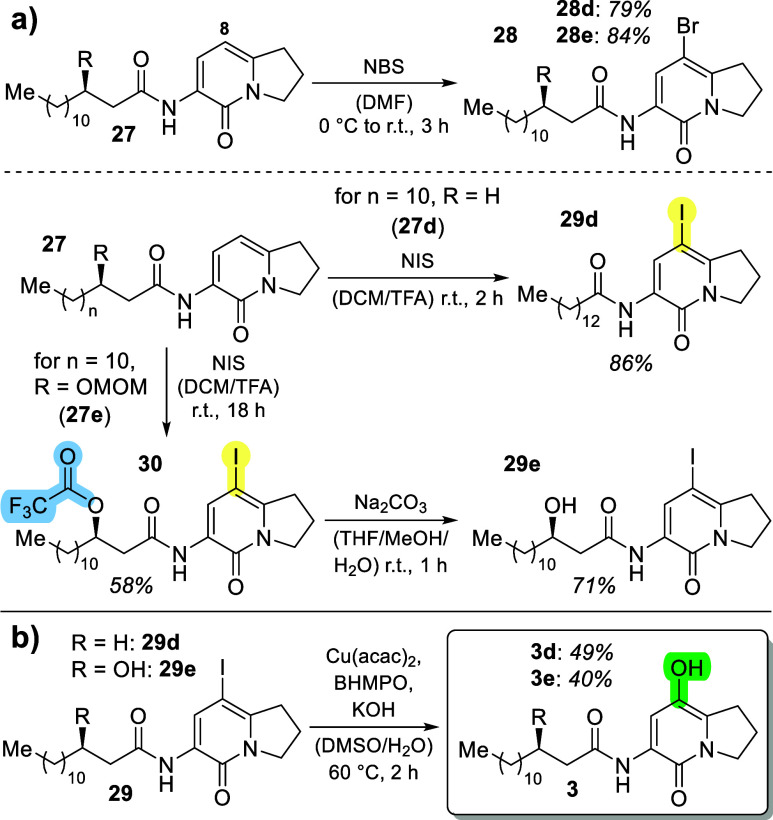
(a) Halogenation Reactions on the 6-Membered Ring **27**; (b) Copper-Catalyzed Hydroxylation of 8-Iodoindolizinones **29**(25) BHMPO—*N*^*1*^,*N*^*2*^-bis(4-hydroxy-2,6-dimethylphenyl)oxalamide.

Since direct and selective arene hydroxylation is difficult to achieve, we aimed at the introduction of the hydroxyl group by installation of an intermediate halide. Direct bromination of **27** with *N*-bromosuccinimide (NBS) in DMF delivered the desired compounds **28** in 79% (**28d**) and 84% (**28e**) yield (Scheme 3). The regioselectivity was investigated by 2D-NMR studies, corroborating bromination exclusively at the desired C-8 carbon atom. However, an attempted palladium-catalyzed hydroxylation with KOH, the *t*BuBrettPhos ligand, and a palladacycle precatalyst at 80 °C in dioxane^24^ did not work in the test reactions conducted with **28d**, but it only led to reisolation of the starting material. Therefore, we switched to iodides instead of bromides and then aimed at testing a mild copper-catalyzed Ullmann-type hydroxylation that was recently described by Xia et al.^25^

Direct iodination of **27** was impossible under the conditions used for bromination (*N*-iodosuccinimide (NIS) instead of NBS). However, additional activation of NIS with TFA^26^ resulted in the clean formation of iodinated product **29d** in 86% yield. In the case of **27e**, concomitant MOM-deprotection and Fischer-esterification to the TFA-ester of the alcohol were observed, resulting in the formation of **30** in 58% yield. This ester was easily hydrolyzed with Na_2_CO_3_ in a mixture of THF, MeOH, and water, resulting in the desired **29e** in 71% yield.

With these compounds readily available, copper-catalyzed hydroxylation reactions were attempted next. To our delight, employing Cu(acac)_2_, an oxalate-derived ligand BHMPO, and KOH in a mixture of DMSO and water^25^ delivered the desired hydroxylated compounds **3d** and **3e** very efficiently within 2 to 3 h (cf. Scheme 3b). In contrast to the originally reported method, where a solvent ratio of 4/1 (DMSO/H_2_O) was used, we employed 7/1 due to solubility problems. In this way, the desired final products of our synthetic route were isolated in 49% (**3d**) and 40% (**3e**) yield. After the establishment of this synthetic route to the central biosynthetic brabantamide/pyrrolizixenamide intermediate **3d/e**, we generated further analogs of this compound class with differing unbranched saturated side chains, namely, C_6_ (**3a**) and C_10_ (**3c**), following the identical procedures (see Supporting Information for yields of individual steps).

**Scheme 4:**
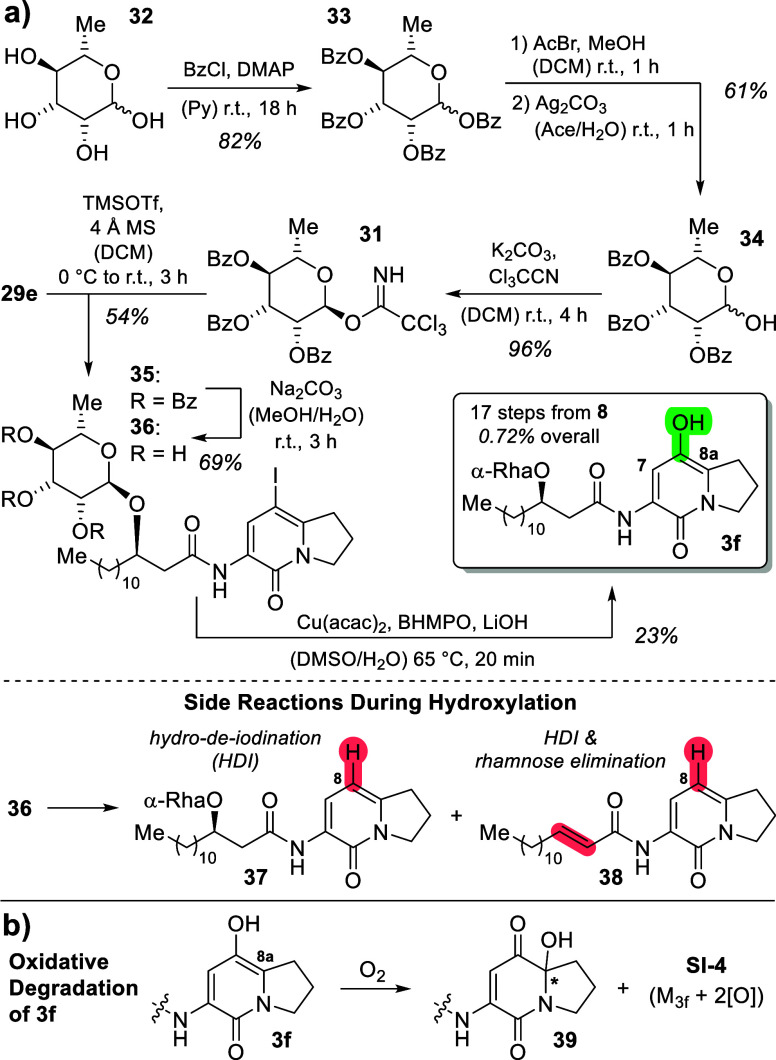
(a) Completion of the Synthesis of Glycosylated Putative Native BraC Substrate **3f** with Observed Side Reactions During the Last Step; (b) Oxidative Degradation of **3f** after Isolation

To conclude the synthesis of putative native glycosylated brabantamide A intermediate **3f**, we attempted rhamnosylation of iodide **29e**. Therefore, synthesis of trichloroacetamide-activated rhamnose building block **31** was conducted in three steps (Scheme 4),^27^ including 4-fold benzoylation of l-rhamnose (**32**) toward **33** with BzCl and pyridine in 82% yield and subsequent formal selective deprotection of the anomeric hydroxyl group of **33**. This was achieved by the formation of the intermediate bromide by treatment of **33** with AcBr in DCM/MeOH and hydrolysis following the Königs–Knorr protocol with Ag_2_CO_3_ in aqueous acetone to yield hemiacetal **34** in 61% yield. K_2_CO_3_-induced addition of Cl_3_CCN (trichloroacetonitrile) to **34** furnished imidate **31** in 96% yield. This building block was used in a TMSOTf-catalyzed glycosylation of alcohol **29e** to selectively yield the α-glycoside **35** in 54% yield (as corroborated by NOESY-NMR; cf. Supporting Information, Figure S257). Benzoyl-deprotection with aqueous Na_2_CO_3_ in MeOH furnished iodide **36** in 69% yield. This compound was then used in the copper-catalyzed hydroxylation, which we had employed for synthesis of the other substrate analogs **3** (cf. Scheme 3b) with LiOH instead of KOH. However, we observed that this reaction suffered from poor reproducibility for iodide **36**. Nevertheless, HRMS (*m*/*z* calcd for **3f**: [M + Na]^+^, 561.3147; found, 561.3149) showed that the transformation worked in principle and that the glycoside is not affected by the strong basic reaction conditions, at least when the reaction time is kept short (<15 min). After tedious optimization of this reaction, best conditions were found using small substrate amounts (2–3 mg of iodide **36**) with high loadings of Cu-catalyst, ligand and base (LiOH), as well as stirring at 65 °C for 10 to 20 min (monitored by HPLC). The reaction was then put on liquid nitrogen to avoid previously observed side and degradation reactions, and the product was directly purified by preparative HPLC. Nevertheless, during the reaction, two prominent side products were repeatedly observed. Isolation and ^1^H NMR analysis showed these to be the products of hydro-de-iodination (HDI, **37**) and subsequent rhamnose elimination (**38**) of iodide **36**. To investigate the origin of the replacing hydrogen atom in the HDI side reaction, the hydroxylation was conducted in DMSO-*d*_6_/D_2_O,^28^ followed by isolation of the corresponding (side) product **37**. Indeed, HRMS revealed a large amount of deuteration (57% by mass counts), indicating the solvents as a source of the introduced hydrogen in the 8-position in **37** or **38** (cf. Scheme 4a, bottom; Supporting Information, chapter 3.1.14.2).

Given the small amounts of **3f** producible and its instability in solution (as observed by HPLC after NMR measurements), in-depth structure analysis and verification were challenging. However, by collecting the isolated products of several reactions (e.g., 1.5 mg from three small-scale reactions, i.e., 23%) for extensive 2D NMR spectroscopy, all hydrogen and carbon atoms of the desired final product **3f** could be unequivocally assigned (see Supporting Information, chapter 3.1.14.2). Taken together, alcohol **3f** was synthesized de novo in 24 steps (17 linear steps from acid chloride **8** and 0.72% overall yield) to yield sufficient amounts for enzyme activity/selectivity assays.

It is noteworthy that even directly after purification, NMR showed small amounts of various degradation products, which were identified by HRMS to be mono- and dioxygenated substances (“+O”, “+2O”). After prolonged NMR measurements, the amounts of these compounds increased drastically. We isolated the three most prominent oxidative degradation products by preparative HPLC. NMR analysis indicated that the oxidative processes resulted in two diastereomers of the corresponding 8a-hydroxyketones **39** (cf. Scheme 4b; Supporting Information, chapter 3.1.14.2). This observation goes in hand with a recent study on bohemamine biosynthesis, where highly similar 8a-hydroxylated compounds were observed and presumed to originate from spontaneous air-oxidation.^29^ The third degradation product could not be isolated in sufficient amounts and purity for characterization; however, ^1^H NMR and HRMS data led to the assumption that this compound was additionally oxidized (**SI-4**).

**Scheme 5:**
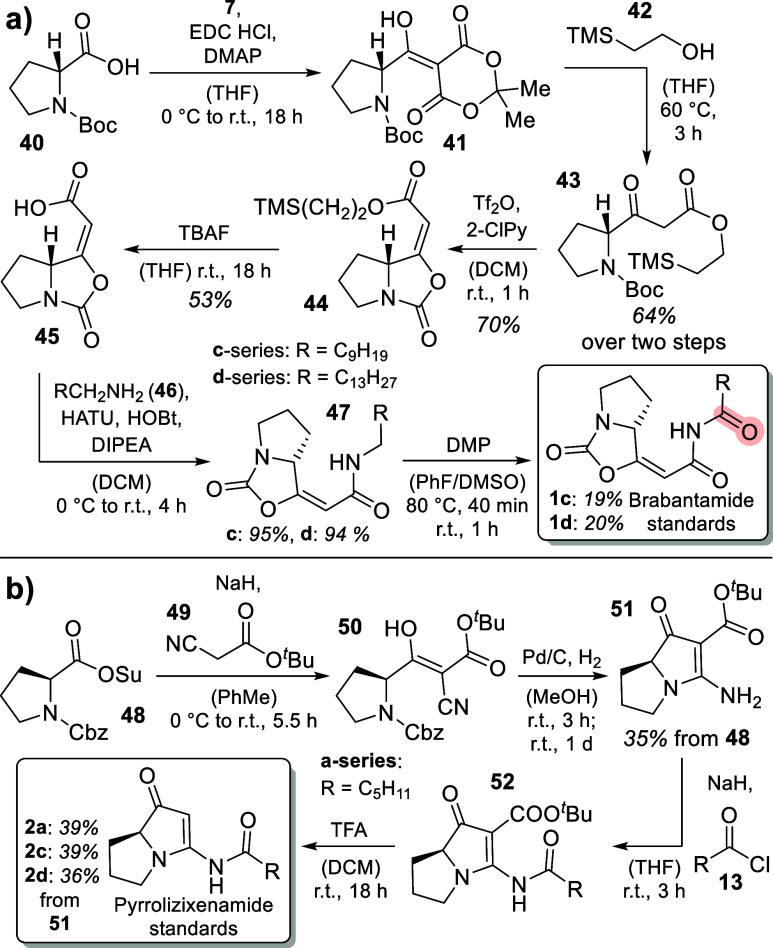
Synthesis of (a) Brabantamide (**1c/d**) and (b) Pyrrolizixenamide (**2a/c/d**) Standards for Corroboration of Enzymatic Products

For unambiguous product identification during our planned in vitro studies on BraC and PxaB, we next aimed at the total synthesis of a set of side-chain-modified brabantamide and pyrrolizixenamide analytical standards. Their availability was particularly important to allow discrimination between the [5.3.0]-bicyclic oxidized intermediates **4** and the rearranged brabantamides, e.g., **1e** (identical molecular masses), based on comparison of enzyme reaction products to the standards. For the synthesis of brabantamide standards **1c/1d**, we commenced by application of a synthetic route published by Záborský et al.^30^ After acylation of Meldrum’s acid (**7**) with Boc-protected d-proline (**40**) to intermediate **41**, alcoholysis with 2-TMS-ethanol (**42**) yielded the β-ketoester **43** in 64% yield over 2 steps. Tf_2_O- and 2-chloropyridine-mediated cyclization yielded (*E*)-configured carbamate **44** in 70% yield. Deprotection with TBAF gave free acid **45** in 53% yield. Since the direct coupling of acid **45** with primary amides to furnish the desired imides **1c/1d** was not successful, we first coupled acid **45** with different unbranched alkyl amines (C_10_: **46c**; C_14_: **46d**), resulting in formation of amides **47c** and **47d** in 95 and 94% yield, respectively. To expand the published synthesis to the required generation of brabantamide congeners containing the characteristic imide moieties, we engaged in the oxidation of the side-chain amides. To achieve this, DMP-mediated amide oxidation employing a modified protocol as reported by Nicolaou et al.^31^ yielded the corresponding brabantamide standards **1c** and **1d** (19 and 20%; Scheme 5a), hence also establishing the first total synthesis of this type of compounds (see Supporting Information, chapter 3.2.1 for all synthetic details).

To synthesize pyrrolizixenamide standards **2a/c/d** (Scheme 5b), we adopted a synthetic route published by Duvall et al.,^32^ starting from Cbz-protected and succinimide-activated proline building block **48** and *tert*-butylcyanoacetate (**49**). Aldol reaction of these starting materials with NaH in toluene gave enol **50**, which was directly deprotected with hydrogen and palladium on activated charcoal. After direct cyclization upon standing at room temperature for 1 day to yield bicycle **51** in 35% yield over 2/3 steps, the C_6_ (**52a**), C_10_ (**52c**) and C_14_ (**52d**) side chains were attached using the corresponding acyl chlorides **13** after deprotonation of amine **51** with NaH. Subsequent TFA-mediated deprotection of the carboxyl moiety followed by decarboxylation gave the desired pyrrolizixenamide derivatives **2a**, **2c**, and **2d** (39, 39, 36%; see Supporting Information, chapter 3.2.2 for synthetic details).

Our next aim was to produce recombinant BraC and PxaB to elucidate the influence of the side chain composition in substrate **3** on the overall product outcome for both enzymes. Therefore, we cloned the corresponding biosynthetic genes with *N*-terminal SUMO- and His-tag. We used restriction digestion to linearize the pET28b vector template and PCR for amplification of genes *braC* and *pxaB* from gDNA of the strains *Pseudomonas* sp. SH-C52 and *Xenorhabdus stockiae* DSM17904, respectively, and SLIC^33^ to assemble the corresponding plasmids (cf. Supporting Information, chapter 1.2). These were then transformed into different expression strains, namely, *E. coli* BAP1, BL21(DE3), and Δmtn for BraC and *E. coli* BL21(DE3) for PxaB; all of them containing kanamycin resistance cassettes. For both enzymes, large-scale expression was performed in 8 L of kanamycin-supplemented (50 mg/L) TB-medium with induction by 0.1 mm IPTG (cf. Supporting Information, chapter 1.2 for details). For BraC production, SUMO-BraC *E. coli* Δmtn was selected, and for PxaB expression, we used SUMO-PxaB *E. coli* BL21(DE3). Nickel NTA affinity chromatography was used to purify the recombinant enzymes. PxaB expression and purification resulted in high amounts of pure and soluble enzyme (∼8.4 mg/L culture or ∼1.6 mg/g pellet). BraC suffered from lower solubility and hence generally lower isolated amounts (∼2.5 mg/L culture or ∼0.8 mg/g pellet). However, sufficient material for in vitro assays with both enzymes was obtained (for SDS-PAGE analysis, see Supporting Information, chapter 1.2.3).

With the recombinant enzymes as well as all substrates and product standards in hands, we next aimed at developing an assay to test the substrate promiscuity and product outcome of the target proteins, BraC and PxaB. After solubility tests for both substrates and enzymes, we used the following concentrations and conditions for the in vitro assays: 300 μm substrate, 100 μm NADPH, 50 μm FAD, and 30 μm BVMO (10 mol-%) in TRIS HCl buffer (50 mm, pH 7.5, 10% glycerol), and 10% (v/v) DMSO. While concentrated PxaB solutions appeared strongly yellow, BraC solutions were only slightly yellowish. We therefore supplemented all in vitro assays with additional FAD (for identification of FAD binding sites, see Supporting Information, chapter 1.2.4). After aerobic incubation at 30 °C for 18 h, assays were extracted with EtOAc, the solvent was removed in vacuo, and the residues were dissolved in MeOH and analyzed by RP-HPLC-MS/MS.

**Figure 2:**
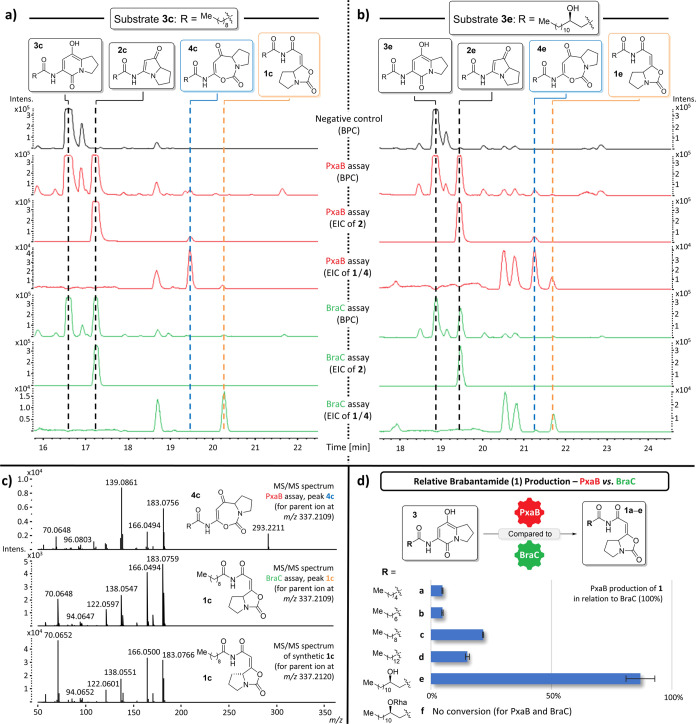
Comparison of PxaB assays (red) and BraC assays (green) exemplarily shown for (a) C_10_- and (b) C_14_OH-substrates **3c** and **3e**. Structures of substrates and products observed in the assays are indicated at the top of the figure. Other peaks were already present in the negative controls (cf. Supporting Information), presumably representing degradation products of the substrates. (c) MS/MS spectra of **4c** and **1c** from assays, as well as of synthetic **1c**, used for product identification. (d) Comparison of catalytic competence of both enzymes toward **1a**–**e**. BPC—base peak chromatogram showing all compounds detected in MS analysis; EIC—extracted ion chromatogram set at the masses of the target compounds.

We performed the described in vitro assays for both enzymes with the six synthesized substrates **3a**–**f** (cf. Figure 2; Supporting Information, chapter 5.3). All negative controls (assays without enzymes) showed small amounts of different degradation products, most probably resulting from air-oxidation (Scheme 4b), such as formation of 8a-hydroxyketones (e.g., **39**, cf. Supporting Information, chapter 5.3.6).^29^

Evaluation of the assay results was enabled by comparison of the observed product masses with the respective analytical standards using HPLC-HRMS (cf. Supporting Information, chapter 5.2). We observed that for substrates **3a**–**e**, the corresponding pyrrolizixenamide derivatives **2a**–**e** were formed in high quantities by both enzymes (exemplarily depicted in Figure 2a/b for substrates **3c** and **3e**, respectively). While this is not surprising for PxaB, it indicated that BraC can also induce PA formation. This explains previous observations by Schimming et al. during exchange experiments in vivo.^10^ In addition, all assays with PxaB still contained significant amounts of pathway intermediate **4** (cf. compounds **4c**/**e** in Figure 2a/b; blue label). In BraC assays, by contrast, this intermediate was either completely absent (for substrates **3a**–**c**) or present in only barely detectable amounts (for substrates **3d/e**; cf. product **4e** in Figure 2b). This indicates a better overall substrate conversion by this BVMO.

Most interestingly, BraC assays with all substrates **3a**–**e** showed additional formation of significant amounts of another product with an identical mass when compared to pathway intermediates **4**. Analysis of the MS/MS fragmentation patterns in comparison to our synthetic standards (exemplarily shown for **4c** versus **1c** in Figure 2c; for details see Supporting Information, chapter 4.4) allowed for the unambiguous identification of these compounds to be brabantamide analogs **1** (cf. Figure 2a/b for analogs **1c/e** labeled in orange). A close inspection of the PxaB assays revealed that these brabantamide-type products were barely detectable (for **3a/b**), formed in small amounts (for **3c/d**), or formed in almost equal levels (only **3e**) when compared to the respective BraC assays. Figure 2d depicts the catalytic competence of PxaB for the production of brabantamide analogs **1** in direct comparison to that of BraC (BraC activity set to 100% for each substrate; relative activity of PxaB depicted, experiments performed in triplicate; see Supporting Information, chapter 5.5). This clearly corroborates the very low activity of PxaB toward products **1** for short-chain substrates **3a/b** (<5%) and sluggish performance for **3c/d** (approximately 15–20%). Only substrate **3e** corresponding to the natural brabantamide A precursor structure was also efficiently converted to **1e** by PxaB, at comparable rates as for BraC (80–95%), thus revealing tight substrate-controlled product selectivity of PxaB with a particularly strong impact of the side-chain β-hydroxy function.

Importantly, irrespective of the employed substrate and utilized enzyme, the major product of all assays was the respective PA analog **2** (with an estimated product ratio of **1** versus **2** of <10 to >90% for all substrates **3a**–**e** and both enzymes). This raised the question whether a nonenzymatic process is involved in the formation of **2** from joint biosynthetic precursor **4**. Therefore, we exemplarily performed a PxaB assay with substrate **3c** and stopped it after 75 min by extraction with EtOAc. After evaporation of the solvent in vacuo, the remaining residue was redissolved in either MeOH or assay buffer (cf. Supporting Information, chapter 5.4). An immediate HPLC-MS/MS measurement showed large amounts of **4c** accompanied by the already formed **2c**. We then incubated these samples at 30 °C and monitored potential changes by LCMS after approximately 2, 5, 18, and 96 h.

While **4c** was observed to be reasonably stable in methanol, complete transformation to **2c** was detected in assay buffer after 5 h. This result indeed shows that the hydrolysis/decarboxylative ring contraction/dehydration sequence from **4c** to **2c** is a fast nonenzymatic process in aqueous solution. We further corroborated these results by additionally performing above-described analyses for substrate analogs **3a** and **3e**, leading to an identical outcome. Therefore, PAs **2** are shown to be formed as nonenzymatic products from **4**, irrespective of the enzyme employed.

In summary, we have shown that both enzymes transform all substrates **3a**–**e** into joint biosynthetic intermediates **4a**–**e**, which are nonenzymatically converted into the respective PA derivatives **2a**–**e**. The brabantamide biosynthetic enzyme BraC additionally generates significant amounts of expected pathway end products **1a**–**e** from all substrates **3a**–**e**. For PxaB, however, only hydroxylated substrate **3e**, which is identical to the native brabantamide A precursor molecule, is efficiently converted to brabantamide analog **1e** (Figure 2d). This indicates tight substrate-directed product selectivity for this enzyme. In the native producer strains, the precursor structure is controlled by the pathway-specific NRPSs, BraB and PxaA. As the latter incorporates only unsubstituted short-chain *N*-acyl groups up to C_8_,^18^ this NRPS serves as strict gate-keeper preventing brabantamide-type product formation.

Given the observed substrate-controlled product selectivity, we were interested to test whether the rhamnosylated brabantamide substrate analog, **3f**, would lead to an even more pronounced shift of the product spectrum. This compound corresponds to the theoretical direct precursor to brabantamide A (**1**). However, **3f** was not accepted by both enzymes, with no formation of products **4f**, **1f,** or **2f** observable (cf. Supporting Information, chapter 5.3.6). The glycosylated precursor **3f** is thus apparently too large to fit into the active sites of the BVMOs. This in turn also indicates that rhamnosylation is a late-stage tailoring reaction catalyzed by the glycosyl transferase BraA,^14^ taking place after BVMO catalysis.

**Scheme 6:**
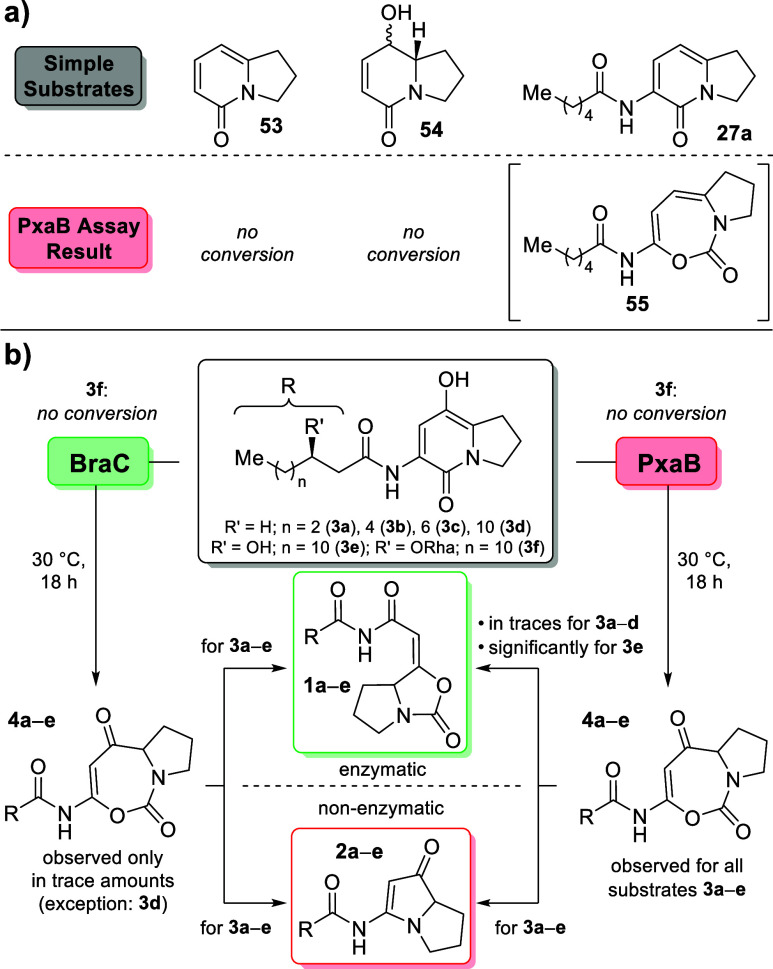
(a) Results of PxaB Assays with Simplified Substrates; (b) Summarized Results of Substrate-Product Relationship Studies on Bacterial BVMOs, BraC and PxaB

We next wanted to probe substrate structure requirements for an initial assessment of the potential general applicability of the studied BVMO biocatalysts. Using PxaB, assays with simplified substrates based on the indolizin-5(1*H*)-one carbon scaffold were performed. When applying unsubstituted 2,3-dihydroindolizin-5(1*H*)-one (**53**; synthesis shown in Supporting Information, chapter 3.3),^34^ no conversion was observed. The same result was obtained with alcohol **54** (synthesis described in Supporting Information, chapter 3.1.6), while assays with the nonhydroxylated C_6_ derivative **27a** only led to the formation of barely detectable amounts of the putative ring-expanded BVMO product **55** (Scheme 6a). These experiments strongly suggest that the enzyme requires the presence of both substituents at the 6-membered ring, the phenolic alcohol function and the *N*-acylated side-chain, for efficient substrate recognition.

## Conclusions

In this study, we investigated the product spectrum of the two BVMOs, BraC and PxaB, forming brabantamides and pyrrolizixenamides, respectively. This was enabled by the total synthesis of selected brabantamides (**1c**, **1d**), pyrrolizixenamides (**2a**, **2c**, **2d**), and putative joint biosynthetic precursors **3a**–**f** of both natural product families and the recombinant production of the two enzymes in *E. coli*. The substrate promiscuity and the product range of the BVMOs were analyzed in-depth using in vitro assays combined with detailed LC–MS/MS analysis. These investigations revealed that both enzymes produce the joint biosynthetic intermediate **4**, which is transformed into PA **2** by a nonenzymatic hydrolysis/decarboxylative ring contraction/dehydration sequence (Scheme 6b). While BraC alone is furthermore catalytically competent to generate significant amounts of the expected brabantamide-type products **1a**–**e** across substrates **3a**–**e**, PxaB performs only efficient brabantamide formation when using substrate analog **3e**, which is identical to the native brabantamide A precursor, thus indicating substrate-directed product selectivity. AlphaFold models of BraC in comparison to PxaB reveal the expected very high structural similarity of these enzymes (see Figure S7). Further insights into the molecular mechanism will require future comparative investigations on protein structures combined with in-depth substrate binding analyses.

The developed synthetic access to complex heterocyclic compounds **3**, which are central intermediates of various natural products, will allow for substrate screening of BVMOs involved in their biosyntheses. This also sets the stage for the development of chemo-enzymatic syntheses of these secondary metabolites. For example, our work paves the way for substrate-product selectivity studies on the highly related BVMO PymC from the recently described pyracrimycin A pathway.^35^ These endeavors are currently ongoing in our laboratories.

## Data Availability

The data that support the findings of this study are reported in the Supporting Information or available from the corresponding author upon request.

## Supporting Information Available

Chemical and biochemical methods, experimental procedures, additional figures including mechanistic considerations, UV spectra of selected compounds and MS/MS fragment analyses, HPLC/MS chromatograms including BPCs and EICs, ESI+ MS and CID MS/MS spectra, supplementary tables and NMR spectra (PDF)
