# Supplementary material for: Product Selectivity in Baeyer–Villiger Monooxygenase-Catalyzed Bacterial Alkaloid Core Structure Maturation
Source: J Am Chem Soc. 2024 Jun 3;146(23):16203–12. doi: 10.1021/jacs.4c04115 (PMC11177316; doi:10.1021/jacs.4c04115)
Supplement: Supplementary file 1 — ja4c04115_si_001.pdf [file ja4c04115_si_001.pdf]

Product Selectivity in Baeyer–Villiger Monooxygenase-Catalyzed Bacterial Alkaloid Core Structure Maturation

Manuel Einsiedler[a,b], Katharina Lamm[b], Jonas F. Ohlrogge[a,b], Sebastian Schuler[b], Ivana J. Richter[b], Tilo Lübken[c] and Tobias A. M. Gulder[a,b,*]

[a] Helmholtz Institute for Pharmaceutical Research Saarland (HIPS), Department of Natural Product Biotechnology, Helmholtz Centre for Infection Research (HZI) and Department of Pharmacy at Saarland University, Campus E8.1, 66123, Saarbrücken, Germany.
[b] Chair of Technical Biochemistry, Technische Universität Dresden, Bergstraße 66, 01069 Dresden, Germany.
[c] Chair of Organic Chemistry I, Technische Universität Dresden, Bergstraße 66, 01069 Dresden, Germany.
*E-mail: tobias.gulder@tu-dresden.de

Supporting Information

Contents

1. General Methods 4
1.1 Chemical and Analytical Methods 4
1.2 Biochemical Methods 5
1.2.1 BraC 6
1.2.2 PxaB 9
1.2.3 Enzyme production and purification 11
1.2.4 Bioinformatic comparison of both enzymes 13
1.2.5 *In vivo* production of [4.3.0]-bicyclic intermediates 3a,b 14
2. In-depth analysis of NRPS intermediates 16
3. Chemical Procedures 18
3.1 Synthesis of [4.3.0]-bicyclic intermediates 3 18
3.1.1 Enantioselective synthesis of β-hydroxy side chain 18
3.1.2 Acylation of methyl L-threoninate (14) 20
3.1.3 Elimination of alcohol 22
3.1.4 Saponification of methyl ester 24
3.1.5 Synthesis of vinylprolinol building block 25
3.1.6 Synthesis of unsubstituted core structure (metathesis test system) 28
3.1.7 Amide coupling (with OH-unprotected amine 22) 29
3.1.8 Amide coupling (with OTBS-protected amine 18) 30
3.1.9 TBS deprotection 32
3.1.10 Ring closing metathesis 34
3.1.11 'Swern elimination' 36

3.1.12 Halogenations 38
3.1.13 Hydroxylation 41
3.1.14 Synthesis of putative natural BraC substrate 3f 43
3.1.14.1 Synthesis of activated rhamnose building block **31** 43
3.1.14.2 Glycosylation, deprotection and hydroxylation 45
3.2 Synthesis of brabantamide/pyrrolizixenamide standards 51
3.2.1 Brabantamide synthesis[14] 51
3.2.1.1 Bicyclic enol core synthesis 51
3.2.1.2 Amide coupling 52
3.2.1.3 Amide oxidation[15] 53
3.2.2 Pyrrolizixenamide synthesis[16] 55
3.2.2.1 PA core synthesis 55
3.2.2.2 Acylation/deprotection sequence 56
3.3 Synthesis of simple substrate 53 57
4. Additional Figures 59
4.1 Stereochemical investigation of alcohols 26 59
4.2 Mechanistic investigation of 'Swern elimination' from 26 to 27 61
4.3 UV spectra of late-stage synthetic compounds 62
4.4 Detailed MS/MS fragment characterization of 4c and 1c 64
5. HPLC / MS Data 66
5.1 Chromatograms of product standards 66
5.1.1 Pyrrolizixenamide analogs 66
5.1.2 Brabantamide analogs 67
5.2 MS spectra of substrates and product standards 68
5.2.1 MS data 68
5.2.1.1 Substrates **3** 68
5.2.1.2 Brabantamide analogs **1c/d** 69
5.2.1.3 Pyrrolizixenamide analogs **2a/c/d** 70
5.2.2 MS/MS data 71
5.2.2.1 Substrates **3** 71
5.2.2.2 Brabantamide analogs **1c/d** 72
5.2.2.3 Pyrrolizixenamide analogs **2a/c/d** 73
5.3 Qualitative assay data 74
5.3.1 C_6-Substrate 3a 74
5.3.1.1 PxaB assay 74
5.3.1.2 BraC assay 76

5.3.2 C_8-Substrate 3b 78
5.3.2.1 PxaB assay 78
5.3.2.2 BraC assay 80
5.3.3 C_{10}-Substrate 3c 82
5.3.3.1 PxaB assay 82
5.3.3.2 BraC assay 84
5.3.4 C_{14}-Substrate 3d 86
5.3.4.1 PxaB assay 86
5.3.4.2 BraC assay 88
5.3.5 C_{14}OH-Substrate 3e 90
5.3.5.1 PxaB assay 90
5.3.5.2 BraC assay 92
5.3.6 C_{14}ORha substrate 3f 94
5.3.6.1 PxaB assay 94
5.3.6.2 BraC assay 96
5.3.7 PxaB assays with other substrates 98
5.3.7.1 Core structures without side chain 98
5.3.7.2 Non-hydroxylated C_6-substrate **27a** 102
5.4 Stability tracking of intermediate 4 104
5.4.1 C_6-Substrate 3a 104
5.4.2 C_{10}-Substrate 3c 106
5.4.3 C_{14}OH-Substrate 3e 108
5.5 Comparison of catalytic competence towards brabantamide analogs 110
6. NMR Spectra 112
6.1 2D NMR analysis of NRPS products (produced *in vivo*) 112
6.2 Synthetic substances 120
7. List of Abbreviations 199
8. Literature 200

1. General Methods

1.1 Chemical and Analytical Methods

Solvents and reagents

Solvents for HPLC and MS analysis, such as acetonitrile and methanol, were purchased from Fisher Scientific and VWR in a purity of over 99% (HPLC-grade). Water was purified and deionized using a TKA GenPure water treatment system. Dry solvents, such as acetonitrile, dichloromethane, methanol, ethanol and tetrahydrofuran, for procedures under inert atmosphere were prepared by distillation and dried over molecular sieves (3 Å or 4 Å). Commercial materials and other solvents were purchased at the highest commercial quality from the providers Acros Organics, Alfa Aesar, Carbolution, Carl Roth, Merck, Sigma Aldrich, VWR, TCI Chemicals and ThermoFisher Scientific. Grubbs-II catalyst was purchased from BLDPharm.

Chromatography

Thin-layer chromatography (TLC) was performed on precoated plates of silica gel F254 (Merck) with UV detection at 254 and 365 nm. Column chromatography was performed on silica gel 60 Geduran® Si 60 (40-60 µm) (Merck). High Performance Liquid Chromatography (HPLC) analysis was performed on a Azura® Knauer system consisting of an AS 6.1L sampler, a P 6.1L pump and a DAD 2.1L detector. The system was controlled by ClarityChrom software. A reversed phase column (100-3 C18A, 150 × 4.6 mm) with integrated precolumn manufactured by Knauer (with column oven at 25 °C) was used with the following solvents: A = H_2O + 0.05% TFA, B = ACN + 0.05% TFA. The separation method consisted of the following gradient system: 0–2 min: 95% A, 2–25 min: 95–5% A, 25–28 min: 5% A, 28–31 min: 95% A, with a flow rate of 1 mL/min. Eluting compounds were monitored at 220 nm. Purification of compounds was performed on a semi-preparative Medium Pressure Liquid Chromatography (MPLC) device (Reveleris X2) manufactured by Grace with reversed phase columns (C18, 40 g or 12 g). The system was controlled by the Reveleris Navigator software and the eluent system consisted of solvents A and B. The separation method used the following gradient system: 0–2 min: 95% A, 2–21 min: 95–5% A, 21–23 min: 5% A, 23–25 min: 95% A, with a flow rate of 36 or 28 mL/min. Preparative HPLC was performed on a Jasco system consisting of an UV-1575 Intelligent UV/VIS-Detector, two PU-2086 Plus Intelligent Prep pumps, MIKA 1000 Dynamic Mixing Chamber, 1000 µL injection port and a LC-NetII/ADC. The system was controlled by the Galaxie software. A reversed phase column (100-5 C18A, 250 × 16 mm) with precolumn manufactured by Knauer was used. The separation method consisted of the following gradient system: 0–2 min: 95% A, 2–25 min: 95–5% A, 25–26 min: 5% A, 26–28 min: 95% A. The used flow rate was 10 mL/min at 220 nm. For very unpolar compounds, the gradient was altered: 0–2 min: 95% A, 2–25 min: 95–5% A, 25–38 min: 5–0% A, 38–40 min: 95% A.

LC-MS

Liquid chromatography, coupled to mass spectrometry (LC-MS), was performed on a Azura® HPLC device manufactured by Knauer, consisting of the following components: AS 6.1L sampler, P 6.1L pump, DAD 2.1L detector. This device was coupled to an ESI mass spectrometer manufactured by Advion with single-quadrupole mass analyzer. The system was controlled by ClarityChrom software in combination with Advion Mass Express software. The same gradient as described above was used.

HR-MS

For direct high resolution mass spectrometry (HR-MS) a Waters Xevo G2-XS QTOF with electrospray ionization (ESI), as well as an Agilent mass spectrometer 6538 with ESI, with high resolution Q-TOF mass analyzer and microchannel plate detector were used.

LC-HRMS

For high resolution mass spectrometry and MS/MS measurements after HPLC separation, a Bruker UHPLC consisting of a Elute autosampler and a HPG 1300 pump was used. This was coupled to an impact II mass spectrometer with ESI source and Q-TOF mass analyzer manufactured by Bruker. The following parameters were used: solvents: A = H_2O + 0.05% formic acid (FA), B = ACN + 0.05% FA; separation method: 0–2 min: 95% A, 2–25 min: 95–5% A, 25–28 min: 5% A, 28–30 min: 95% A; flow rate: 0.3 mL/min; column: Intensity Solo 2 C18, 100 x 2.1 mm (in column oven: 40 °C). For MS/MS, auto-MS/MS mode with 20–50 eV collision energy (N_2) was used. The system was controlled by Bruker Compass® HyStar software; analysis was conducted with Bruker Compass® Data Analysis software.

NMR

Nuclear Magnetic Resonance (NMR) spectra were recorded on Bruker AVANCE II 300, ASCEND 400 and AVANCE III 600 spectrometers at ambient temperature. The chemical shifts are given in δ-values (ppm) relative to TMS (^{1}H, ^{13}C), $MeNO_2$ (^{15}N) or $CFCl_3$ (^{19}F). ^{1}H and ^{13}C spectra were referenced internally using the residual solvent resonances ($CDCl_3$: δ_H = 7.26 ppm, δ_C = 77.16 ppm; DMSO-d_6: δ_H = 2.50 ppm, δ_C = 39.52 ppm; CD_3OD: δ_H = 3.31 ppm, δ_C = 49.00 ppm). ^{19}F chemical shifts were internally referenced using residual TFA ($CDCl_3$: δ_F = –75.39 ppm)[1] or by unified chemical shift scale. ^{15}N chemical shifts were taken from ^{1}H–^{15}N-HMBC and HSQC experiments and were referenced using universal calibration (Ξ = 0.10136767)[2]. The coupling constants *J* are given in Hertz [Hz] and determined assuming first-order spin-spin coupling. The following abbreviations were used for the allocation of signal multiplicities: s – singlet, bs – broad singlet, vbs – very broad singlet ($\Delta\delta^{1H}$ > 1 ppm), d – doublet, bd – broad doublet, t – triplet, bt – broad triplet, q – quartet, qnt – quintet, sxt – sextet, spt – septet, m – multiplet, or any combination thereof.

Specific rotation

The specific rotation was measured with a PerkinElmer Model 341 LLC Polarimeter or a Krüss P3000 at 20 °C and 589 nm in methanol or chloroform. The concentration of the compounds during the measurements are given in units of g/100 mL.

1.2 Biochemical Methods

SDS-PAGE

12% acrylamide separation gels were used (electrophoresis settings: 35 mA/gel; 180 V). All samples (20 µL) were mixed with 5 µL loading buffer (333 mM TRIS, 400 mM, 280 mM SDS, 6 mM bromophenol blue, 4.4 M glycerol) and heated to 95 °C for five minutes. 9 µL sample or 6 µL marker were applied per bag. Staining was conducted by shaking in 1.5 mM Coomassie Brilliant Blue solution (45 vol-% H_2O, 45 vol-% MeOH, 10 vol-% AcOH); destaining was achieved by shaking in 60 vol-% H_2O, 30 vol-% MeOH, 10 vol-% AcOH. The marker (PageRuler™ broad range unstained protein ladder) was purchased from ThermoFisher Scientific.

Used primers

Table S1. Overview over used primers with sequences.

#	Name	Sequence 5'–3'	Use
P1	pET28bSUMO_BraC_fwd	AGAACAGATTGGTGGATCCACTAGTATGAC GGAACCGCGTAAA	Amplification of insert
P2	pET28bSUMO_BraC_rev	TGGTGGTGGTGCTCGAGTGCGGCCTTAGT CGTTGACATACGCCG	Amplification of insert
P3	BraC_screen_fwd	GTCATGACCGAAGGCTAT	BraC clone screening
P4	screen_f1 ori_rev	GAACCATCACCCTAATCAAG	BraC/PxaB clone screening, sequencing
P5	screen_T7_fwd	TAATACGACTCACTATAGGG	BraC/PxaB clone screening, sequencing
P6	Seq_PxaB_	CAAGTGATTATATCTGTTGG	PxaB sequencing

1.2.1 BraC

Cloning of pET28b-SUMO::*braC* vector

To linearize the pET28b-SUMO vector, it was digested with enzymes SpeI-HF and NotI-HF in a 50 µL assay, containing 1 µg of DNA (volume depending on concentration), 5 µL of CutSmart® buffer, 1 µL of each restriction enzyme and 43 µL water. After incubation at 37 °C for 1 hour, enzymes were deactivated at 80 °C for 20 minutes. The *braC* insert (57% GC, 1185 bp) was amplified from a pXst6-*braC* vector (codon-optimized for *E. coli*; received from Helge Bode, University of Frankfurt)[3] by PCR. The reaction was performed in 8 PCR assays (25 µL each) with Q5 Polymerase according to the manual of the supplier (0.63 µL of primers P1/P2, 0.25 µL dNTPs, 0.33 µL template DNA (~20 ng), 5.0 µL buffer, 2.5 µL GC enhancer, 0.13 µL Q5 polymerase and 15.53 µL water) with 8 different annealing temperatures from 64 °C to 54 °C. After the analysis by agarose gel (1%) electrophoresis proved the success for all tested temperatures, two PCR assays each were combined to four fractions in total (each 50 µL). After the PCR, the template DNA was shredded by a DpnI digest (50 µL PCR reaction mixture + 6.0 µL CutSmart® buffer + 0.5 µL DpnI, 37 °C for 18 hours, 80 °C for 20 minutes) and the insert as well as vector DNA were purified by a Monarch DNA gel extraction kit (from New England BioLabs; NEB) after electrophoresis. After determining the concentration (insert: 83.6 ng/µL, vector: 28.9 ng/mL) by NanoDrop, the two parts were assembled by sequence and ligation independent cloning (SLIC). Therefore, 0.1 pmol linearized vector were mixed with 0.02 pmol insert DNA in a 10 µL reaction volume containing 1 µL NEBuffer 2.1 and 0.5 µL T4 polymerase. After incubation for 90 s at room temperature and 10 minutes on ice, the reaction mixture was used for a transformation into chemo-competent *E. coli* DH5α cells: the SLIC assay was added to an aliquot of competent cells, incubated for 30 minutes on ice, followed by a heat-shock (42 °C, 90 s). After the cells have cooled down (2 min on ice) 900 µL of SOC medium was added and the cells were incubated at 37 °C for 1 hour at slight shaking (550 rpm). The cells were plated on agar plates containing kanamycin (50 µg/mL). After growing overnight at 37 °C, cultures were subjected to colony-screening PCR using OneTaq polymerase and primers P3/P4. Four positive clones were used for overnight cultures (5 mL each, 50 mg/L kanamycin-supplemented LB medium) at 37 °C overnight. Followed by plasmid isolation by Monarch plasmid miniprep kit, a control restriction digest by XmnI as well as Sanger sequencing (using primers P3/P5) confirmed the positive cloning. For protein expression, the plasmid was transformed into *E. coli* BL21 (DE3), *E. coli* BAP1 and *E. coli* Δmtn (DE3). For later expression, the cells were stored at –80 °C (containing 15 % glycerol).

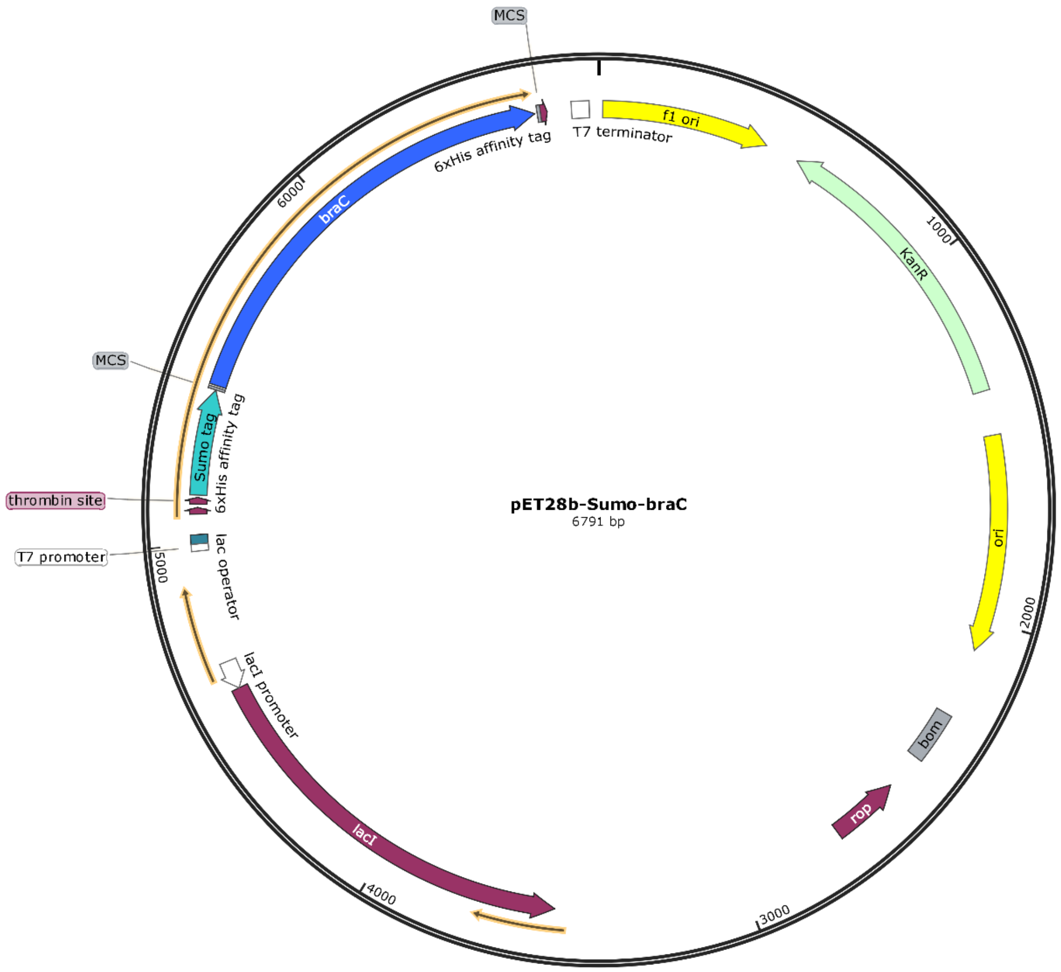

Figure S1. Plasmid map of pET28b-SUMO::*braC* vector.

Gene sequences

SUMO::*braC* – 1551 bp

5‘atgggcagcagccatcatcatcatcatcacagcagcggcctggtgccgcgcggcagccatatgtcggactcagaagtcaatcaagaagctaagccagaggt
caagccagaagtcaagcctgagactcacatcaatttaaaggtgtccgatggatcttcagagatcttcttcaagatcaaaaagaccactcctttaagaaggctgat
ggaagcgttcgctaaaagacagggtaaggaaatggactccttaagattcttgtacgacggtattagaattcaagctgatcagacccctgaagatttggacatgg
aggataacgatattattgaggctcacagagaacagattggtggatccactagtatgacggaaccgcgtaaagctatcgtggctggcgcaggtatcggtggtctg
acggccgcaatcgcactgcaacgtgcaggctggcaagtgaaagttttcgaagcagcacagaccctgcgtacgggcggtaccggtctgtcaattatggcaaacgc
tatggcagctctgcattcgatcgatgcgcacgtcccggtggaacaggcaggccaagctatccgtcagtttttctttaaaaaacataatggtcaaccgattacccgc
atgccgatccatgaagttggcgaacagctgggtcacccgaacgtcaatatccaacgtccgctgctgctgcgtgcactggcacagcaactggcaccggatagcct
gaccacgggtctgcgttgcgtgggttattctcaccgcccggatggcgttacggtccagttcgaagacggtagtacccaagaagcagatctgctgattggcgctga
cggtctgaactccgtgatccgtcagcaaatgctgggtgaaaccccgacgcgtccgtcaggttacattgcgtggctggcagttacgccgctgcgtcatccggtcatg
accgaaggctatgtggcccactactggggccgtggtaaacgctttggtctgtgcgatgtgggcgacggtcatgcatattggtggggcacgtgtaaccgtgcaaat
gcagcagatgacgccctgaatgtgtcgaaacaggaagttctgggcgcatacgctggttgggcgccggaagtggttgcagctattgaagcgaccccggaaagcg
ccctgctgaaaatgcatgcccgtgatcgccacccggttaaacagtgccgcgatggtcatgtcgtgctgctgggtgacgcagcacacccgatgctgccgtctctgg
gtcagggtgcagctcaagcgatcgaagatgccgttgtcctggcaaactgtctggctcagaccccggacctgggctcagcactggcacattatcaagcataccgtc
tgccgcgcgcgaacggcattgttaatgcggcccgttttatgagtggtatcgaacaggcggaatccacgttcgcatgttgggctcgtgaatggtattttcgcctgacc
ccgcagagctcttggcgtaagaaaaacctggatattctgtccttcaaaccgggtctgcaaccggtggcggcgtatgtcaacgactaa

braC – 1185 bp

5‘atgacggaaccgcgtaaagctatcgtggctggcgcaggtatcggtggtctgacggccgcaatcgcactgcaacgtgcaggctggcaagtgaaagttttcgaa
gcagcacagaccctgcgtacgggcggtaccggtctgtcaattatggcaaacgctatggcagctctgcattcgatcgatgcgcacgtcccggtggaacaggcagg

ccaagctatccgtcagtttttctttaaaaaacataatggtcaaccgattacccgcatgccgatccatgaagttggcgaacagctgggtcacccgaacgtcaatatc
caacgtccgctgctgctgcgtgcactggcacagcaactggcaccggatagcctgaccacgggtctgcgttgcgtgggttattctcaccgcccggatggcgttacg
gtccagttcgaagacggtagtacccaagaagcagatctgctgattggcgctgacggtctgaactccgtgatccgtcagcaaatgctgggtgaaaccccgacgcg
tccgtcaggttacattgcgtggctggcagttacgccgctgcgtcatccggtcatgaccgaaggctatgtggcccactactggggccgtggtaaacgctttggtctgt
gcgatgtgggcgacggtcatgcatattggtggggcacgtgtaaccgtgcaaatgcagcagatgacgccctgaatgtgtcgaaacaggaagttctgggcgcatac
gctggttgggcgccggaagtggttgcagctattgaagcgaccccggaaagcgccctgctgaaaatgcatgcccgtgatcgccacccggttaaacagtgccgcga
tggtcatgtcgtgctgctgggtgacgcagcacacccgatgctgccgtctctgggtcagggtgcagctcaagcgatcgaagatgccgttgtcctggcaaactgtctg
gctcagaccccggacctgggctcagcactggcacattatcaagcataccgtctgccgcgcgcgaacggcattgttaatgcggcccgttttatgagtggtatcgaa
caggcggaatccacgttcgcatgttgggctcgtgaatggtattttcgcctgaccccgcagagctcttggcgtaagaaaaacctggatattctgtccttcaaaccgg
gtctgcaaccggtggcggcgtatgtcaacgac

Amino acid sequences

SUMO-BraC – 516 AAs, 56.4 kDa

MGSSHHHHHHSSGLVPRGSHMSDSEVNQEAKPEVKPEVKPETHINLKVSDGSSEIFFKIKKTTPLRRLMEAFAKRQGKEMDSLR
FLYDGIRIQADQTPEDLDMEDNDIIEAHREQIGGSTSMTEPRKAIVAGAGIGGLTAAIALQRAGWQVKVFEAAQTLRTGGTGLSI
MANAMAALHSIDAHVPVEQAGQAIRQFFFKKHNGQPITRMPIHEVGEQLGHPNVNIQRPLLLRALAQQLAPDSLTTGLRCVG
YSHRPDGVTVQFEDGSTQEADLLIGADGLNSVIRQQMLGETPTRPSGYIAWLAVTPLRHPVMTEGYVAHYWGRGKRFGLCDV
GDGHAYWWGTCNRANAADDALNVSKQEVLGAYAGWAPEVVAAIEATPESALLKMHARDRHPVKQCRDGHVVLLGDAAHP
MLPSLGQGAAQAIEDAVVLANCLAQTPDLGSALAHYQAYRLPRANGIVNAARFMSGIEQAESTFACWAREWYFRLTPQSSWR
KKNLDILSFKPGLQPVAAYVND

BraC – 395 AAs, 42.7 kDa

MTEPRKAIVAGAGIGGLTAAIALQRAGWQVKVFEAAQTLRTGGTGLSIMANAMAALHSIDAHVPVEQAGQAIRQFFFKKHNG
QPITRMPIHEVGEQLGHPNVNIQRPLLLRALAQQLAPDSLTTGLRCVGYSHRPDGVTVQFEDGSTQEADLLIGADGLNSVIRQQ
MLGETPTRPSGYIAWLAVTPLRHPVMTEGYVAHYWGRGKRFGLCDVGDGHAYWWGTCNRANAADDALNVSKQEVLGAYA
GWAPEVVAAIEATPESALLKMHARDRHPVKQCRDGHVVLLGDAAHPMLPSLGQGAAQAIEDAVVLANCLAQTPDLGSALAH
YQAYRLPRANGIVNAARFMSGIEQAESTFACWAREWYFRLTPQSSWRKKNLDILSFKPGLQPVAAYVND

Test expression

Test expressions with pET28b-SUMO*::braC*-containing strains and different IPTG concentrations were conducted to identify the best expression conditions. After growing the cultures in kanamycin-supplemented (50 mg/L) TB medium (50 mL each) at 37 °C up to an OD_{600} of around 0.8, the cells were cooled and protein expression was induced by addition of IPTG at two different final concentrations (0.05 mᴍ, 0.1 mᴍ). The cultures were incubated at 16 °C overnight (180 rpm). Cell were harvested, resuspended in lysis buffer (10 mᴍ imidazole, 50 mᴍ NaH_2PO_4, 300 mᴍ NaCl, 10 vol-% glycerol, pH 8), and lysed by sonication (Branson Digital Sonifier 450-D; 35% amplitude, 1 s pulse, 1 s break, 2 minutes). After centrifugation (30 minutes, 16700 × *g*). the supernatants and the pellets were analyzed by SDS-PAGE showing ∆mtn cells at 0.1 mᴍ IPTG to be the best expression conditions.

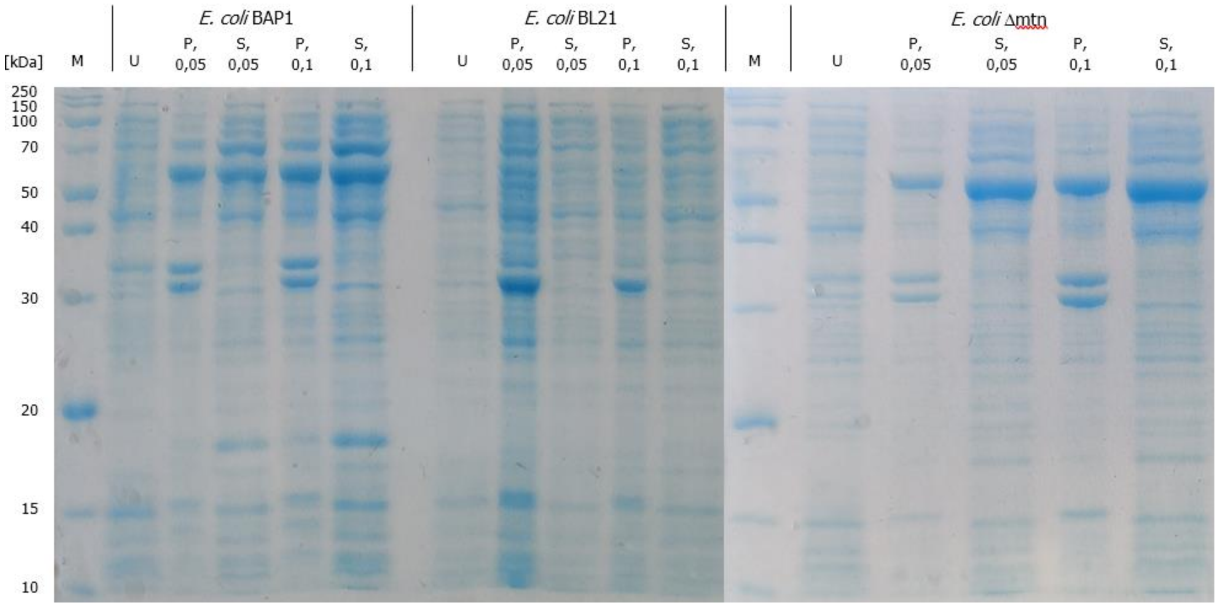

Figure S2. SDS-PAGE of test expressions for BraC. M – Marker, U – Uninduced culture, P – Pellet, S – Supernatant; IPTG concentrations given in mM. Size of desired enzyme: 56.4 kDa.

1.2.2 PxaB

Cloning of pET28bSUMOPxaB vector

The MOX gene *pxaB* originating in *Xenorhabdus stockiae* DSM17904 was amplified from plasmid DNA (pXst5, received from Helge Bode, University of Frankfurt)[3] as template. Cloning was performed similar as described above for *braC*, with transformation into *E. coli* BL21(DE3).

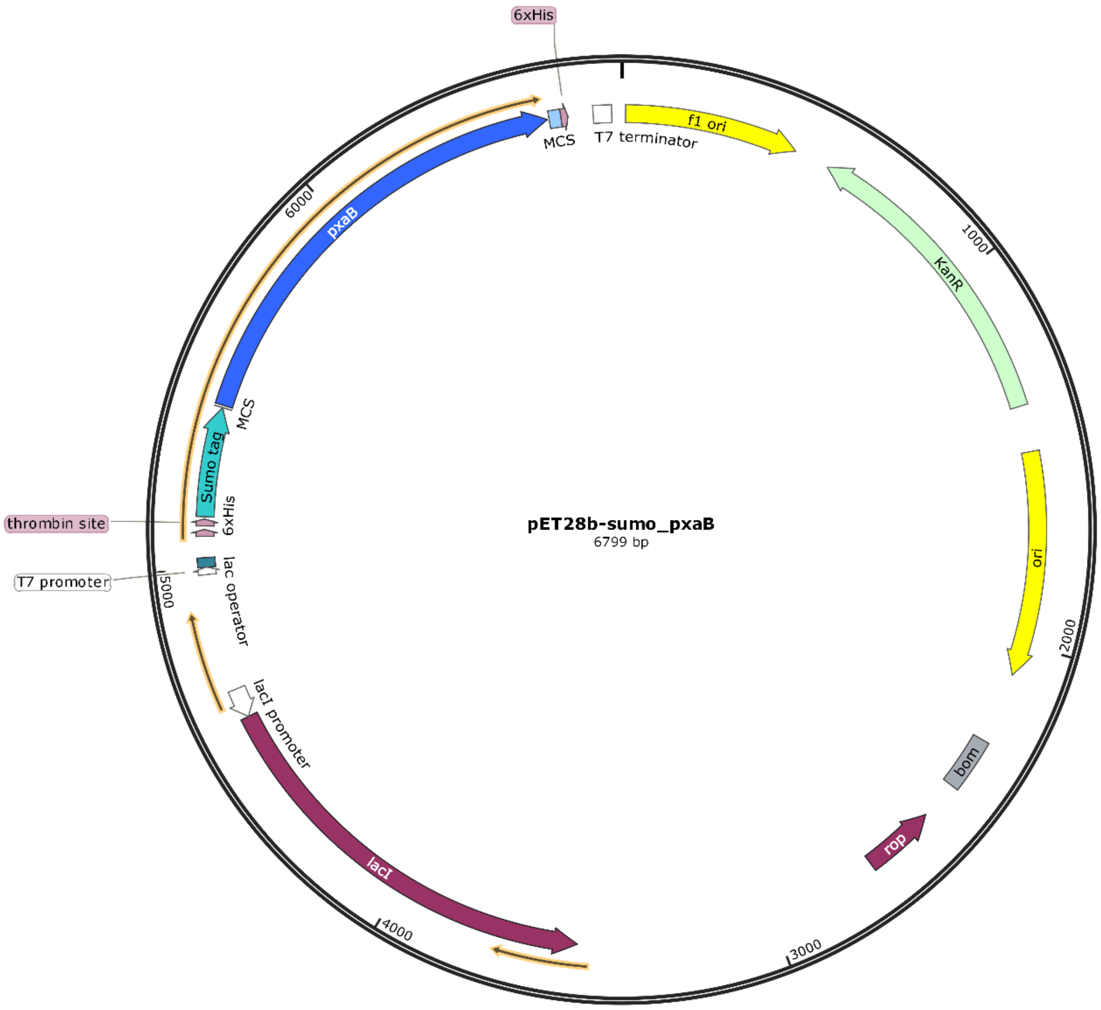

Figure S3. Plasmid map of pET28b-SUMO*::pxaB* vector.

Gene sequences

SUMO*::pxaB* – 1536 bp

5‘atgggcagcagccatcatcatcatcatcacagcagcggcctggtgccgcgcggcagccatatgtcggactcagaagtcaatcaagaagctaagccagaggt
caagccagaagtcaagcctgagactcacatcaatttaaaggtgtccgatggatcttcagagatcttcttcaagatcaaaaagaccactcctttaagaaggctgat
ggaagcgttcgctaaaagacagggtaaggaaatggactccttaagattcttgtacgacggtattagaattcaagctgatcagacccctgaagatttggacatgg
aggataacgatattattgaggctcacagagaacagattggtggatccatgagtcgcccgctcaaagtaattattgcgggcgcggggatcggcggactcacgacg
gcagtagtgctgcaacgtttaggttatgaagtagaagtctatgaacaggcgacgacgctccgcacagccggaagcggtctgtccgtaattaccaatgcggtagc
ggcattgtcctccatcggggtgaatttgaaactggaaaacttcggagccccagttaaaaatttcgaaatccgcaatgtgaaagaccagttgatccgtcgtatgcc
ggtgccggagatttccagcagcaatggtttcgattcggtgtgtctcagccgcaaagcgctccaggaagcactgctgcaacagttagaccagagcatcatccatgt
agatgcaaaggtcgatgaaatcattgagagcgataatgctgtcactgtccgtttcgcggatggccgtgaaacacagggcgatcttctcattggtgctgacggaat
ccactctgttgttcgtgagtatctgcaaggcaatcaaccactccgtgcaagtgattatatctgttggttggcgattacgcgttaccagcaccctcagattaccctg
ggtacgttgtacattacttgggccaaggcaaacgtgtaggcctgatcgatattgggggcggcgaagtgtattggtggggcacggccaatatgagcaacaaacag
gcatgtcactggcaagggagcaaccaggatgtgctggcatacttcgaagggtggccaacgattgtgtctgatattatttctcagaccccatccgaggatatcatta
gcgtctcggcgcaggatcggccgtttagcccgctttggggcaaagggcgtattactctcttaggagacgctgcgcatcctatgctgaccacgcttgggcagggcg
caggtatggcaatcgaggatgcggcggtcctgggtcatatgctgaaacagcagcctgatcctgtggctgcactgcgccagtacgaaaaagttcgtatcccgcgtg
ctgagctgtttgtgaacgaaagtaagatccagagcgatctgggccaagaagatggcatctttgattgtcaacaacgcgaactgacgctccagtccttgccggag
actgcgctgcgccaggattttgagcgcttcttacattttccggttggcgacttaaccttgcagactttataa

pxaB – 1179 bp

5‘atgagtcgcccgctcaaagtaattattgcgggcgcggggatcggcggactcacgacggcagtagtgctgcaacgtttaggttatgaagtagaagtctatgaac
aggcgacgacgctccgcacagccggaagcggtctgtccgtaattaccaatgcggtagcggcattgtcctccatcggggtgaatttgaaactggaaaacttcgga

gccccagttaaaaatttcgaaatccgcaatgtgaaagaccagttgatccgtcgtatgccggtgccggagatttccagcagcaatggtttcgattcggtgtgtctca
gccgcaaagcgctccaggaagcactgctgcaacagttagaccagagcatcatccatgtagatgcaaaggtcgatgaaatcattgagagcgataatgctgtcact
gtccgtttcgcggatggccgtgaaacacagggcgatcttctcattggtgctgacggaatccactctgttgttcgtgagtatctgcaaggcaatcaaccactccgtgc
aagtgattatatctgttggttggcgattacgcgttaccagcaccctcagattacccctgggtacgttgtacattacttgggccaaggcaaacgtgtaggcctgatcg
atattgggggcggcgaagtgtattggtggggcacggccaatatgagcaacaaacaggcatgtcactggcaagggagcaaccaggatgtgctggcatacttcga
agggtggccaacgattgtgtctgatattatttctcagaccccatccgaggatatcattagcgtctcggcgcaggatcggccgtttagcccgctttggggcaaaggg
cgtattactctcttaggagacgctgcgcatcctatgctgaccacgcttgggcagggcgcaggtatggcaatcgaggatgcggcggtcctgggtcatatgctgaaa
cagcagcctgatcctgtggctgcactgcgccagtacgaaaaagttcgtatcccgcgtgctgagctgtttgtgaacgaaagtaagatccagagcgatctgggcca
agaagatggcatctttgattgtcaacaacgcgaactgacgctccagtccttgccggagactgcgctgcgccaggattttgagcgcttcttacattttccggttggcg
acttaaccttgcagactttataa

Amino acid sequences

SUMO-PxaB – 511 AAs, 56.5 kDa

MGSSHHHHHHSSGLVPRGSHMSDSEVNQEAKPEVKPEVKPETHINLKVSDGSSEIFFKIKKTTPLRRLMEAFAKRQGKEMDSLR
FLYDGIRIQADQTPEDLDMEDNDIIEAHREQIGGSMSRPLKVIIAGAGIGGLTTAVVLQRLGYEVEVYEQATTLRTAGSGLSVITN
AVAALSSIGVNLKLENFGAPVKNFEIRNVKDQLIRRMPVPEISSSNGFDSVCLSRKALQEALLQQLDQSIIHVDAKVDEIIESDNAV
TVRFADGRETQGDLLIGADGIHSVVREYLQGNQPLRASDYICWLAITRYQHPQITPGYVVHYLGQGKRVGLIDIGGGEVYWWG
TANMSNKQACHWQGSNQDVLAYFEGWPTIVSDIISQTPSEDIISVSAQDRPFSPLWGKGRITLLGDAAHPMLTTLGQGAGMA
IEDAAVLGHMLKQQPDPVAALRQYEKVRIPRAELFVNESKIQSDLGQEDGIFDCQQRELTLQSLPETALRQDFERFLHFPVGDLT
LQTL

PxaB – 392 AAs, 43.0 kDa

MSRPLKVIIAGAGIGGLTTAVVLQRLGYEVEVYEQATTLRTAGSGLSVITNAVAALSSIGVNLKLENFGAPVKNFEIRNVKDQLIRR
MPVPEISSSNGFDSVCLSRKALQEALLQQLDQSIIHVDAKVDEIIESDNAVTVRFADGRETQGDLLIGADGIHSVVREYLQGNQPL
RASDYICWLAITRYQHPQITPGYVVHYLGQGKRVGLIDIGGGEVYWWGTANMSNKQACHWQGSNQDVLAYFEGWPTIVSDII
SQTPSEDIISVSAQDRPFSPLWGKGRITLLGDAAHPMLTTLGQGAGMAIEDAAVLGHMLKQQPDPVAALRQYEKVRIPRAELF
VNESKIQSDLGQEDGIFDCQQRELTLQSLPETALRQDFERFLHFPVGDLTLQTL

1.2.3 Enzyme production and purification

Cultivation and expression

The main cultures (2 L TB medium, 50 mg/L kanamycin-supplemented) were inoculated with 20 mL of overnight culture (LB medium, 50 mg/L kanamycin-supplemented) that were grown from the corresponding cryo-stock. After reaching an OD_{600} value of around 0.8, the cultures were cooled, and the expression was induced by addition of 200 µL of IPTG solution (1 M). Cultures were incubated at 16 °C overnight while shaking (180 rpm). Afterwards, cells were harvested by centrifugation (6000 × *g*, 10 minutes). The pellets were resuspended in 0.9% NaCl solution, transferred to falcon tubes, centrifuged as described above and stored at –80 °C.

Purification

Frozen bacterial cell mass (around 3.5 g) was thawed in 15 mL of His-tag lysis buffer and suspended by vigorous vortexing. After cell lysis by sonication (35%, 10 s pulse, 10 s break, 1:40 min), centrifugation (16700 × *g*, 4 °C, 30 min) removed insoluble cell debris. The supernatant was quickly transferred into a falcon tube and 2 mL of Ni-NTA resin was added. After incubation at 0 °C for 1 hour with slight shaking, the mixture was applied to a column and the resin was washed with buffer containing 40 mM to 50 mM imidazole (50 mM NaH_2PO_4, 300 mM NaCl, 10 vol-% glycerol, pH 7.5) to remove unspecifically bound enzymes. Elution was conducted with 5 mL of elution buffer containing 250 mM imidazole (50 mM NaH_2PO_4, 300 mM NaCl, 10 vol-% glycerol, pH 8). The eluate was concentrated in a VivaSpin column manufactured by *Sartorius* (30000 MWCO), followed by two washing steps (20 mL each) with BVMO storage buffer (50 mM TRIS HCl, 10 vol-% glycerol, pH 7.5). The final concentration was

determined on a *Implen* NanoDrop device, using the following data, as calculated by ExPASy ProtParam (in reduced form):

BraC: M_W = 56396.96 Da, ε_{280} = 65890 L·mol^{-1}·cm^{-1};

PxaB: M_w = 56512.98 Da, ε_{280} = 49390 L·mol^{-1}·cm^{-1}.

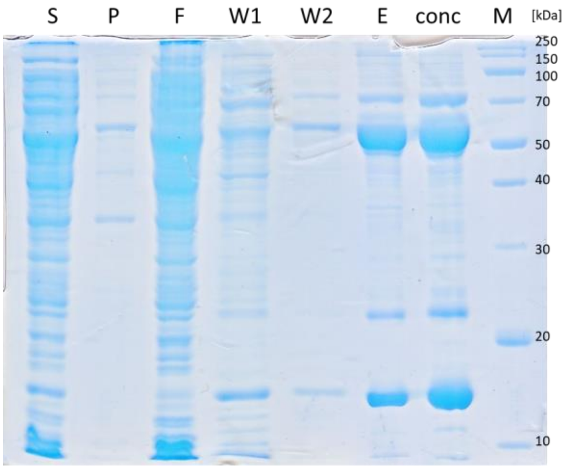

Figure S4. SDS-PAGE of BraC purification. S – Supernatant, P – Pellet, F – Flowthrough of Ni-NTA column, W1 – 1st washing with 40 mM imidazole, W2 – 2nd washing with 40 mM imidazole, E – Eluate. Size of desired enzyme: 56.4 kDa.

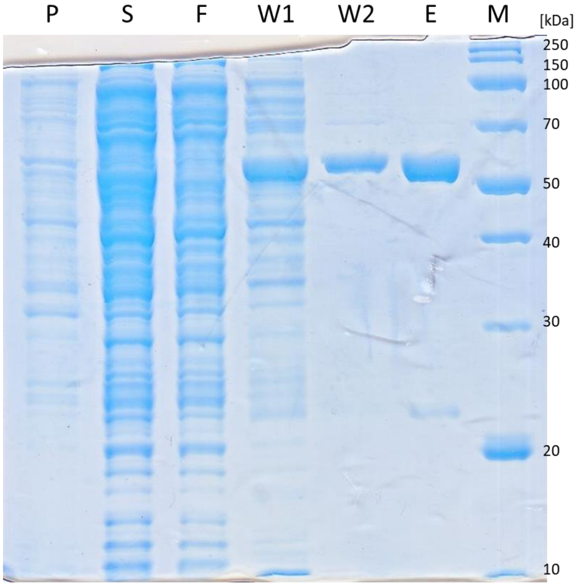

Figure S5. SDS-PAGE of PxaB purification. P – Pellet, S – Supernatant, F – Flowthrough of Ni-NTA column, W1 – washing with 40 mM imidazole, W2 – washing with 50 mM imidazole, E – Eluate. Size of desired enzyme: 56.5 kDa.

1.2.4 Bioinformatic comparison of both enzymes

Amino acid alignments

Blast search of the amino acid sequence of BraC/PxaB delivered HpxO as most similar enzyme in the PDB (identity: BraC: 32%; PxaB: 30%; similarity: BraC: 47%; PxaB: 48%). HpxO is a FAD-binding enzyme; its crystal structure (3RP8) has been determined in a study by Cole *et al.*[4] The following amino acids were described to be involved in FAD-binding:

Table S2. Amino acids involved in FAD-binding in HpxO.[4] HB – hydrogen bond.

AA	Function/ Interaction	AA	Function/ Interaction	AA	Function/ Interaction
I6	FAD-surrounding (adenine base)	G7	FAD-surrounding (ribose sugar)	I42	isoalloxazine ring binding pocket
V32		A31		P292	
L123		A153		G298	
R124		E30	2’-hydroxyl HB	C299	
V125		Q107	3’-hydroxyl HB	G9	adjacent residues
D154		G11	β-phosphate	I9	
G155		G297	*C-2* carbonyl HB	R103	
A159		S43	*N-3* HB	G284	
				D285	

```
HpxO_3RP8_1     ----MKAIVIGAGIGGLSAAVALKQSGIDCDVYEAVKEIKPVGAAISVWPNGVKCMAHLG    56
BraC            MTEPRKAIVAGAGIGGLTAAIALQRAGWQVKVFEAAQTLRTGGTGLSIMANAMAALHSID    60
PxaB            MSRPLKVIIAGAGIGGLTTAVVLQRLGYEVEVYEQATTLRTAGSGLSVITNAVAALSSIG    60
Similarity      :    *.*: *******::*:.*:: * : .*:* .  ::  *:.:*:  *.: .:  :.

HpxO_3RP8_1     MGDIMETFGGPLRRMAYRDFRSGENMTQFSLAPLIERTGSRPCPVSRAELQREMLDYWGR    116
BraC            AHVPVEQAGQAIRQFFFKKHN-GQPITRMPIHEVGEQLGHPNVNIQRPLLLRALAQQLAP    119
PxaB            VNLKLENFGAPVKNFEIRNVK-DQLIRRMPVPEISSSNGFDSVCLSRKALQEALLQQLDQ    119
Similarity          :*  *  ::.:  :. . .: : :: :  : .  *     :.*  * . : :

HpxO_3RP8_1     DSVQFGKRVTRCEEDADGVTVWFTDGSSASGDLLIAADGSHSALRPWVLGFTPQRRYAGY    176
BraC            DSLTTGLRCVGYSHRPDGVTVQFEDGSTQEADLLIGADGLNSVIRQQMLGETPTR-PSGY    178
PxaB            SIIHVDAKVDEIIESDNAVTVRFADGRETQGDLLIGADGIHSVVREYLQGNQPLR-ASDY    178
Similarity      . :  . :     .  :.*** * **   ..****.*** :*.:*  : *  * *  :.*

HpxO_3RP8_1     VNWNGLVEIDEALAPGDQWTTFVGEGKQVSLMPVSAGRFYFFFDVPLPAGLAEDRDTLRA    236
BraC            IAWLAVTPLRHPVMTEGYVAHYWGRGKRFGLCDVGDGHAYWWGTCNRANAADDALNVSKQ    238
PxaB            ICWLAITRYQHPQITPGYVVHYLGQGKRVGLIDIGGGEVYWWGTANMSNKQACHWQGSNQ    238
Similarity      : * .:.   .     .  . : *.**:..*  :. *. *::             :  .

HpxO_3RP8_1     DLSRYFAGWAPPVQKLIAALDPQTTNRIEIHDIEPFSRLVRGRVALLGDAGHSTTPDIGQ    296
BraC            EVLGAYAGWAPEVVAAIEATPESALLKMHARDRHPVKQCRDGHVVLLGDAAHPMLPSLGQ    298
PxaB            DVLAYFEGWPTIVSDIISQTPSEDIISVSAQDRPFSPLWGKGRITLLGDAAHPMLTTLGQ    298
Similarity      ::   : **   *   *     .    :  :*         *::.*****.*     :**

HpxO_3RP8_1     GGCAAMEDAVVLGAVFRQTRDIAAALREYEAQRCDRVRDLVLKARKRCDITHGKDMQ--L    354
BraC            GAAQAIEDAVVLANCLAQTPDLGSALAHYQAYRLPRANGIVNAARFMSGIEQAESTFACW    358
PxaB            GAGMAIEDAAVLGHMLKQQPDPVAALRQYEKVRIPRAELFVNESKIQSDLGQEDGIFDCQ    358
Similarity      *.  *:***.**.  : *  * :** .*:  *  *.. :*  ::  ..: : ..

HpxO_3RP8_1     TEAWYQELREETGERIINGMCDTILSGPLG---------- 384
BraC            AREWYFRLTPQSSWRK---KNLDILSFKPGLQPVAAYVND 395
PxaB            QRELTLQSLPETALRQ---DFERFLHFPVGDLTLQTL--- 392
Similarity       .    .   ::. *        :*    *
```

Figure S6. Multiple sequence alignment[5] of amino acid sequences of HpxO, BraC and PxaB. Amino acids involved in FAD-binding are labeled in yellow (see Table S2). Similarity legend: * – identical amino acids; : – very similar amino acids; . – similar amino acids.

AlphaFold2 models

Protein structures were predicted by AlphaFold2.ipynb and aligned by PyMOL (The PyMOL Molecular Graphics System, Version 2.5.5 Schrödinger, LLC).[6, 7]

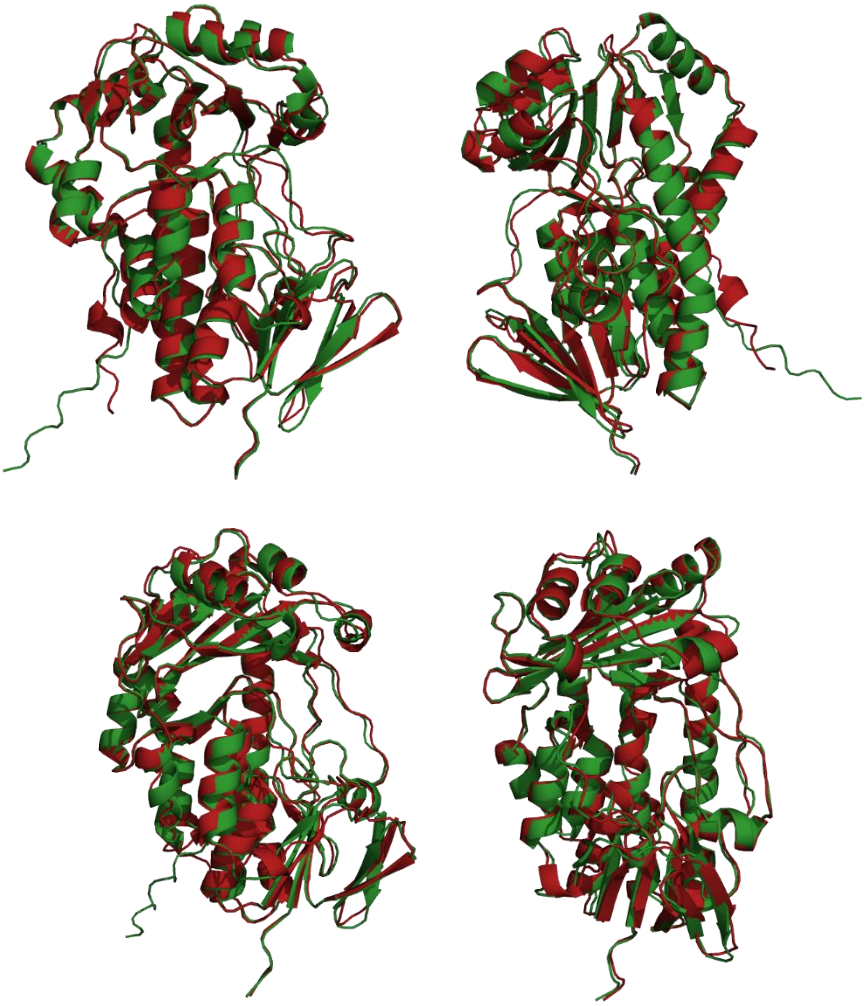

Figure S7. Alignment of the predicted three-dimensional structures of BraC (green) and PxaB (red) from different angles. The root mean square deviation (RMSD) of this alignment is 0.649 Å.

1.2.5 *In vivo* production of [4.3.0]-bicyclic intermediates 3a,b

In vivo production of NRPS intermediates was achieved by expression of the standalone PxaA enzyme in both *E. coli* BAP1 and DH10β cells harboring the functional NRPS gene *pxaA* from *Xenorhabdus stockiae* DSM17904 (pXst4, received from Helge Bode, University of Frankfurt)[3], thereby confirming successful priming of the NRPS PCP-domain upon co-expression with Sfp or MtaA PPTases. Heterologous expression was controlled by an L-arabinose inducible promoter.

Precultures of *E. coli* BAP1 cells harboring arabinose inducible constructs were incubated overnight at 37 °C and 200 rpm shaking in LB medium supplemented with kanamycin (50 mg/L). Grown cells were then used to inoculate main cultures at a 1:100 dilution in TB medium containing kanamycin (50 mg/L). Cultures were left shaking at 28 °C and 180 rpm for 19 h before gene cluster expression was induced with L-arabinose (0.1 mM final concentration). In addition, the basic building blocks L-serine, L-proline as well as a fatty acid of choice, e.g., lauric acid were fed at 100 mg/L. After 10 – 12 h incubation (28 °C, 180 rpm), 1 % (w/v) of washed Amberlites XAD-16 (Alfa Aesar) was added to the expression cultures for metabolite adsorption. Incubation was continued for further 12 h before the culture broth was

centrifuged (6000 × *g*, 10 min) to remove the medium. Cell pellet together with the polymeric adsorption material were subjected to extraction. The metabolites were released from Amberlites XAD-16 by extensive washing with MeOH for 2 h, including MeOH exchange in between. All MeOH phases were pooled, and the solvent was evaporated under reduced pressure. The residue was then dissolved in a 1:1 solvent mixture of H_2O and EtOAc and extracted with EtOAc (3×). Combined organic phases were dried over Na_2SO_4, filtered and the solvent removed under reduced pressure. The residue was subjected to preparative HPLC.

2. In-depth analysis of NRPS intermediates

Two bicyclic pathway intermediates differing only in chain length (**3a**: C_6 and **3b**: C_8) of the fatty acid moiety were isolated from the raw extract of expression cultures (cf. chapter 1.2.4).

N-(8-hydroxy-5-oxo-1,2,3,5-tetrahydroindolizin-6-yl)hexanamide (**3a**)

Purified by semi-preparative HPLC, yielding 9.8 mg of an off-white solid.

3a

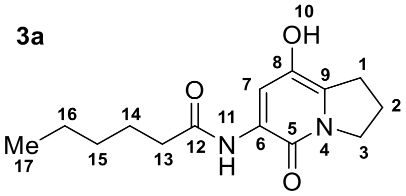

Table S3. Full NMR analysis of **3a** in DMSO-*d*6.

#	^{1}H NMR (δ, *J*)	^{13}C NMR (δ)	^{15}N NMR (δ)	COSY	^{1}H–^{13}C HMBC	^{1}H–^{15}N HMBC
**1**	2.93 (t, 7.6 Hz)	27.72	–	2	2,3,7 (weak),8,9	4
**2**	2.08 (qnt, 7.5 Hz)	21.47	–	1,3	1,3,9	4
**3**	3.99 (bt, 7.5 Hz)	49.14	–	2	1,2,9	4
**4**	–	–	–206.4 (from HMBC)	–	–	–
**5**	–	152.87	–	–	–	–
**6**	–	126.27	–	–	–	–
**7**	8.18 (s)	117.85	–	–	1 (weak),5,8,9	4 (weak), 11
**8**	–	133.45	–	–	–	–
**9**	–	126.05	–	–	–	–
**10**	8.63 (bs)	–	–	–	–	–
**11**	8.99 (s)	–	–255.7 (from HSQC)	–	5,7,12	–
**12**	–	171.95	–	–	–	–
**13**	2.40 (t, 7.5 Hz)	36.18	–	14	12,14,15	11
**14**	1.54 (qnt, 7.5 Hz)	24.97	–	13,15	12 (weak),13,15,16	–
**15**	1.26–1.21 (m)	30.86	–	14,16	16,17 (weak)	–
**16**	1.31–1.26 (m)	21.94	–	15,17	15,17	–
**17**	0.85 (t, 7.0 Hz)	13.92	–	16	15,16	–

HR-MS (ESI+)**:** *m/z* calcd. for $C_{14}H_{20}N_2O_3$ $[M+H]^+$: 265.1547, found: 265.1548; calcd. for $[M+Na]^+$: 287.1366, found: 287.1369. (ESI–): calcd. for $[M-H]^-$: 263.1401, found: 263.1399.

N-(8-hydroxy-5-oxo-1,2,3,5-tetrahydroindolizin-6-yl)octanamide (**3b**)

Purified by semi-preparative HPLC, yielding 5.4 mg of an off-white solid.

3b

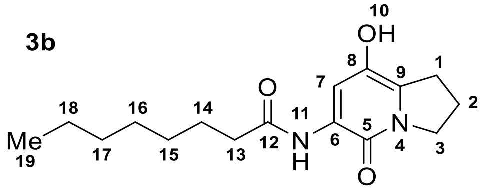

Table S4. Full NMR analysis of **3b** in DMSO-d_6.

#	^{1}H NMR (δ, *J*)	^{13}C NMR (δ)	^{15}N NMR (δ)	COSY	^{1}H–^{13}C HMBC	^{1}H–^{15}N HMBC
**1**	2.93 (t, 7.6 Hz)	27.71	–	2	2,3,7 (weak),8,9	4
**2**	2.08 (qnt, 7.5 Hz)	21.46	–	1,3	1,3,9	4
**3**	4.01–3.97 (m)	49.12	–	2	1,2,9	4
**4**	–	–	−206.7 (from HMBC)	–	–	–
**5**	–	152.86	–	–	–	–
**6**	–	126.26	–	–	–	–
**7**	8.17 (s)	117.83	–	–	1 (weak),5,8,9	4 (weak), 11
**8**	–	133.42	–	–	–	–
**9**	–	126.03	–	–	–	–
**10**	8.62 (s)	–	–	–	–	–
**11**	8.99 (bs)	–	−256.1 (from HMBC)	–	5,7,12	–
**12**	–	171.94	–	–	–	–
**13**	2.40 (t, 7.4 Hz)	36.20	–	14	12,14,15,16	11
**14**	1.54 (qnt, 7.3 Hz)	25.28	–	13	12 (weak),13,15,16	–
**15**	1.29–1.20 (m)	28.59, 28.49	–	Interconvertible due to overlay		–
**16**			–			–
**17**		31.20	–			–
**18**		22.10	–			–
**19**	0.85 (t, 7.0 Hz)	13.99	–	18	17,18	–

HR-MS (ESI+)**:** *m/z* calcd. for $C_{16}H_{24}N_2O_3$ $[M+H]^+$: 293.1860, found: 293.1868; calcd. for $[M+Na]^+$: 315.1680, found: 315.1685.

3. Chemical Procedures

3.1 Synthesis of [4.3.0]-bicyclic intermediates 3

3.1.1 Enantioselective synthesis of β-hydroxy side chain

5-(1-hydroxydodecylidene)-2,2-dimethyl-1,3-dioxane-4,6-dione (**9**)[8]

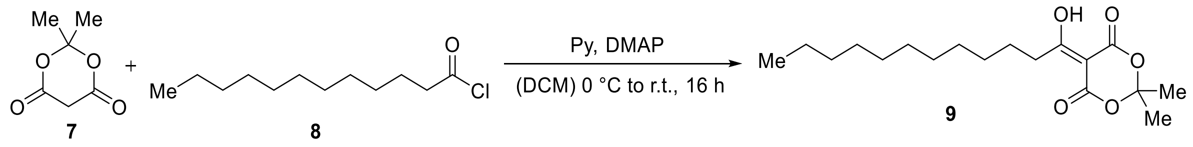

In a 250 mL flame-dried round-bottom Schlenk flask, 4.0 g Meldrum's acid (27.4 mmol, 1.0 eq.), 0.7 g DMAP (5.5 mmol, 0.2 eq.) and 6.3 mL lauroyl chloride (6.0 g, 27.4 mmol, 1.0 eq.) were dissolved in DCM (2 mL/mmol). The obtained solution was cooled to 0 °C, afterwards 4.4 mL pyridine (4.3 g, 54.8 mmol, 2.0 eq.) were added dropwise. The mixture was warmed to room temperature and stirred for 16 hours, before it was washed with H_2O (20 mL) and 0.1 M aqueous HCl (20 mL). The organic phase was dried over Na_2SO_4, filtered and the solvent was removed under reduced pressure, yielding an orange oil which was used in the next step without further purification. A small amount was purified by MPLC. The product was obtained as a mixture of keto–enol tautomers with a ratio of ~ 1:9.

^{1}H-NMR (300 MHz, $CDCl_3$)**:** δ [ppm] = 15.29 (s, 1 H), 3.06 (dd, *J* = 8.3, 7.0 Hz, 2 H), 1.73 (s, 6 H), 1.70–1.63 (m, 2 H), 1.44–1.22 (m, 16 H), 0.91–0.84 (m, 3 H).

^{13}C {^{1}H}-NMR (75.5 MHz, $CDCl_3$)**:** δ [ppm] = 198.5, 170.7, 160.3, 104.9, 91.4, 35.9, 32.0, 29.69 (3C), 29.53, 29.48, 29.43, 29.35, 26.9, 26.3, 22.8, 14.2.

Only NMR-signals of the predominant enol tautomer are given.

HR-MS (ESI+)**:** *m/z* calcd. for $C_{18}H_{30}O_5$ $[M+Na]^+$: 349.1985, found: 349.1976.

methyl 3-oxotetradecanoate (**10**)

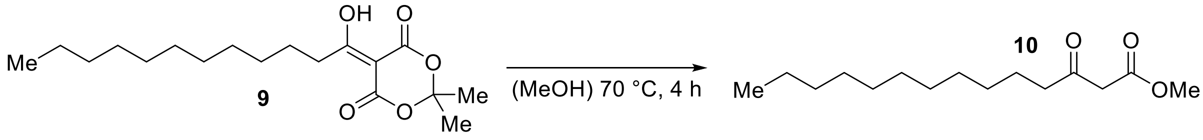

In a 50 mL round-bottom flask, the crude acylated Meldrum's acid (1.0 eq.) was dissolved in MeOH (4 mL/mmol), and the mixture heated to 70 °C for 4 hours. Afterwards, the solvent was removed under reduced pressure and the residue purified by flash chromatography over silica gel with pentane/EtOAc = 50/1, yielding 4.59 g of a yellowish oil, which solidified upon standing (17.9 mmol, 65% over two steps). The product was obtained as a mixture of keto–enol tautomers with a ratio of ~9:1.

^{1}H-NMR (300 MHz, $CDCl_3$)**:** δ [ppm] = 3.73 (s, 3 H), 3.44 (s, 2 H), 2.52 (t, *J* = 7.4 Hz, 2 H), 1.62–1.53 (m, 2 H), 1.33–1.24 (m, 16 H), 0.87 (t, *J* = 6.6 Hz, 3 H).

***R*$_f$** (pentane/EtOAc = 50/1) = 0.30.

Only NMR-signals of the predominant keto tautomer are given.

Known compound, CAS 22348-97-6.

methyl (*R*)-3-hydroxytetradecanoate (**11**)[9]

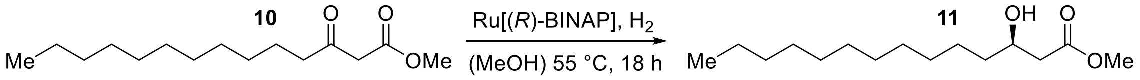

In a flame-dried 50 mL Schlenk flask, Ru(methylallyl)$_2$(COD) (24.7 mg, 77.4 µmol, 2 mol-%) and (*R*)-BINAP (58.1 mg, 92.3 µmol, 2.4 mol-%) were dissolved in 5 mL of dry acetone. The mixture was purged

with argon and methanolic HBr (20 µL of 48% HBr solution in 1 mL of dry MeOH) was added, before being stirred at room temperature for 45 minutes. The solvents were removed under reduced pressure, then a solution of methyl 3-oxotetradecanoate (1.0 g, 3.87 mmol, 1.0 eq.) in 10 mL of dry, degassed MeOH was added to the catalyst. The mixture was purged with hydrogen and afterwards stirred under an atmosphere of hydrogen at 55 °C for 18 hours. After filtration, the solvents were removed *in vacuo* and the residue purified by flash chromatography over silica gel (pentane/EtOAc = 5/1) to yield 0.92 g of a yellowish solid (3.55 mmol, 92%).

^{1}H-NMR (300 MHz, $CDCl_3$)**:** δ [ppm] = 4.04–3.93 (m, 1 H), 3.71 (s, 3 H), 2.52 (bs, 1 H), 2.51 (dd, *J* = 16.4, 3.3 Hz, 1 H), 2.40 (dd, *J* = 16.4, 8.8 Hz, 1 H), 1.52–1.41 (m, 2 H), 1.39–1.20 (m, 18 H), 0.91–0.83 (m, 3 H).

^{13}C {^{1}H}-NMR (75.5 MHz, $CDCl_3$)**:** δ [ppm] = 173.7, 68.2, 51.9, 41.3, 36.7, 32.1, 29.8 (2C), 29.7 (3C), 29.5, 25.6, 22.8, 14.2.

HR-MS (ESI+)**:** *m/z* calcd. for $C_{15}H_{30}O_3$ $[M+Na]^+$: 281.2087, found: 281.2082.

***R*f** (pentane/EtOAc = 4/1) = 0.70.

Specific rotation: $[\alpha]_D = -13.9\ ^{\circ}\cdot mL/_{dm\cdot g}$ (ρ = 0.90; $CHCl_3$).

***ee*:** >99%

Determination of enantiomeric excess:

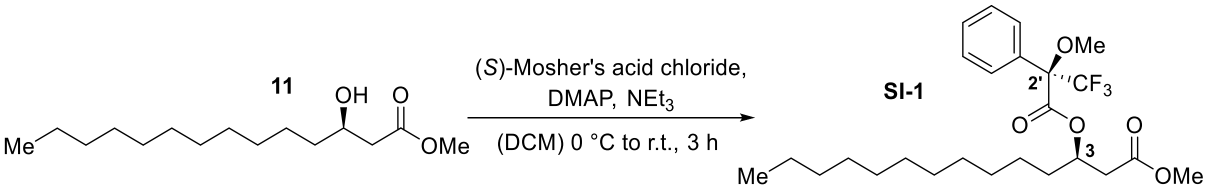

In a flame-dried 10 mL Schlenk flask, methyl (*R*)-3-hydroxytetradecanoate (20.0 mg, 77.4 µmol, 1.0 eq.) was dissolved in 1.5 mL dry DCM, before addition of NEt_3 (42.3 µL, 31.3 mg, 310 µmol, 4.0 eq.), DMAP (47.2 mg, 387 µmol, 5.0 eq.) and (*S*)-Mosher's acid chloride (29.0 µL, 39.1 mg, 155 µmol, 2.0 eq.). The mixture was stirred at room temperature for 2 hours, then poured on 10 mL 0.1 M HCl_{aq}. The aqueous phase was extracted with Et_2O (2 × 15 mL). Combined organic phases were washed with brine, dried over Na_2SO_4, filtered and the solvents removed under reduced pressure. Peak ratio of (3*R*-2'*R*)- and (3*S*-2'*R*)-diastereomers in RP-HPLC showed an enantiomeric ratio of >99:1. The residue was purified by preparative HPLC, yielding 24.9 mg of a colorless oil (52.5 µmol, 68%): methyl (*R*)-3-(((*R*)-3,3,3-trifluoro-2-methoxy-2-phenylpropanoyl)oxy)tetradecanoate (**SI-1**).

^{1}H-NMR (300 MHz, $CDCl_3$)**:** δ [ppm] = 7.58–7.49 (m, 2 H), 7.42–7.33 (m, 3 H), 5.47 (tt, *J* = 6.5, 5.1 Hz, 1 H), 3.66 (s, 3 H), 3.54 (q, *J* = 1.4 Hz, 3 H), 2.70 (dd, *J* = 15.9, 8.1 Hz, 1 H), 2.60 (dd, *J* = 15.9,4.8 Hz, 1 H), 1.70–1.54 (m, 2 H), 1.32–1.15 (m, 18 H), 0.92–0.83 (m, 3 H).

^{13}C {^{1}H}-NMR (75.5 MHz, $CDCl_3$)**:** δ [ppm] = 170.7, 166.1, 132.5, 129.7, 128.5 (2C), 127.5 (2C, q, *J* = 1.3 Hz), 123.4 (q, *J* = 289 Hz), 84.7 (q, *J* = 27.4 Hz), 73.5, 55.6 (q, *J* = 1.6 Hz), 52.0, 38.7, 33.8, 32.1, 29.7 (2C), 29.6, 29.5 (2C), 29.3, 24.8, 22.8, 14.3.

^{19}F {^{1}H}-NMR (282 MHz, $CDCl_3$)**:** δ [ppm] = –71.2.

HR-MS (ESI+)**:** *m/z* calcd. for $C_{25}H_{37}F_3O_5$ $[M+Na]^+$: 497.2485, found: 497.2476.

Specific rotation: $[\alpha]_D = +28.0\ ^{\circ}\cdot mL/_{dm\cdot g}$ (ρ = 1.46; $CHCl_3$).

methyl (*R*)-3-(methoxymethoxy)tetradecanoate (**12**)

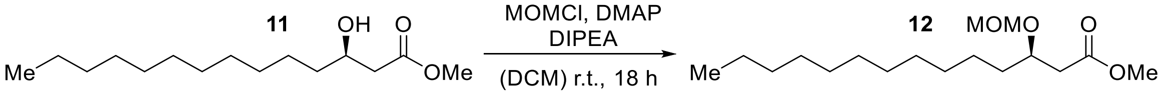

In a flame-dried 50 mL Schlenk flask, methyl (*R*)-3-hydroxytetradecanoate (0.50 g, 1.93 mmol, 1.0 eq.) was dissolved in 20 mL of dry DCM. DMAP (23.0 mg, 0.19 mmol, 0.1 eq.) and DIPEA (1.01 mL, 0.75 g,

5.80 mmol, 3.0 eq.) were added, before dropwise addition of freshly distilled MOM chloride (0.44 mL, 0.47 g, 5.80 mmol, 3.0 eq.). The mixture was stirred at room temperature for 18 hours, before being quenched by addition of 15 mL saturated $NaHCO_3$ solution and vigorous stirring for 30 minutes. The aqueous phase was extracted with DCM (15 mL) and combined organic phases were washed with 0.1 M HCl_{aq} (20 mL) and brine (20 mL), dried over Na_2SO_4, filtered and the volatiles removed under reduced pressure. The residue was purified by flash column chromatography (pentane/EtOAc = 40/1 to 20/1), yielding 0.54 g of a colorless oil (1.80 mmol, 93%).

^{1}H-NMR (300 MHz, $CDCl_3$)**:** δ [ppm] = 4.69–4.61 (m, 2 H), 4.03–3.93 (m, 1 H), 3.67 (s, 3 H), 3.34 (s, 3 H), 2.56 (dd, *J* = 15.1, 7.3 Hz, 1 H), 2.46 (dd, *J* = 15.2, 5.3 Hz, 1 H), 1.64–1.45 (m, 2 H), 1.37–1.20 (m, 18 H), 0.91–0.81 (m, 3 H).

^{13}C {^{1}H}-NMR (75.5 MHz, $CDCl_3$)**:** δ [ppm] = 172.2, 96.1, 74.8, 55.7, 51.7, 40.3, 35.0, 32.0, 29.8 (2C), 29.7 (3C), 29.5, 25.3, 22.8, 14.2.

HR-MS (ESI+)**:** *m/z* calcd. for $C_{17}H_{34}O_4$ $[M+Na]^+$: 325.2349, found: 325.2347.

***R*$_f$** (pentane/EtOAc = 40/1) = 0.19.

Specific rotation: $[\alpha]_D = -7.4\ ^{\circ \cdot mL}/_{dm \cdot g}$ (ρ = 0.99; $CHCl_3$).

(*R*)-3-(methoxymethoxy)tetradecanoic acid (**6e**)

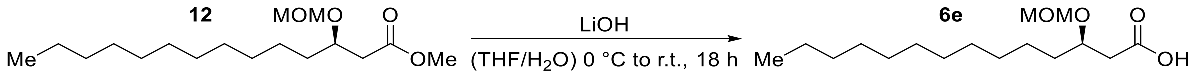

In a 50 mL round-bottomed flask, methyl (*R*)-3-(methoxymethoxy)tetradecanoate (2.18 g, 7.21 mmol, 1.0 eq.) was dissolved in a mixture of THF and water (4/1, 20 mL/mmol) and cooled to 0 °C. LiOH (0.52 g, 21.6 mmol, 3.0 eq.) was added, and the mixture allowed to warm to room temperature. After stirring for 18 hours, the solution was diluted with water (20 mL) and Et_2O (20 mL) and the organic phase was extracted with water (20 mL). Combined aqueous phases were acidified by addition of 1 M HCl_{aq} to a pH of around 3 and extracted with EtOAc (3 × 30 mL). Combined organic extracts were washed with brine (20 mL), dried over Na_2SO_4, filtered and concentrated under reduced pressure to yield the product as a colorless oil (1.89 g, 6.55 mmol, 91%).

^{1}H-NMR (300 MHz, $CDCl_3$)**:** δ [ppm] = 11.07 (bs, 1 H), 4.68–4.57 (m, 2 H), 4.01–3.89 (m, 1 H), 3.32 (s, 3 H), 2.60–2.42 (m, 2 H), 1.61–1.43 (m, 2 H), 1.36–1.16 (m, 18 H), 0.87–0.79 (m, 3 H).

^{13}C {^{1}H}-NMR (75.5 MHz, $CDCl_3$)**:** δ [ppm] = 177.5, 95.9, 74.6, 55.5, 40.1, 34.8, 32.0, 29.7 (2C), 29.6 (3C), 29.4, 25.2, 22.7, 14.1.

HR-MS (ESI+)**:** *m/z* calcd. for $C_{16}H_{32}O_4$ $[M+Na]^+$: 311.2193, found: 311.2190.

***R*$_f$** (pentane/EtOAc = 3/1) = 0.31.

Specific rotation: $[\alpha]_D = -8.9\ ^{\circ \cdot mL}/_{dm \cdot g}$ (ρ = 0.93; $CHCl_3$).

3.1.2 Acylation of methyl L-threoninate (14)

Procedure A:

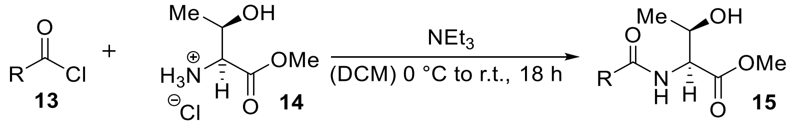

Methyl L-threoninate hydrochloride (1.10 eq.) was dissolved in dry DCM (2 mL/mmol) and NEt_3 (3.0 eq.) was added at 0 °C. The corresponding acid chloride (1.0 eq.) dissolved in dry DCM (1 mL/mmol) was added dropwise and the mixture allowed to warm to room temperature and stirred for 18 hours. The mixture was diluted with EtOAc (30 mL) and washed with 1 M HCl_{aq} (30 mL), saturated $NaHCO_3$ (30 mL) and brine (30 mL). The organic phase was dried over Na_2SO_4, filtered and the solvents

removed under reduced pressure. The residue was purified by column chromatography on silica gel (pentane/EtOAc = 2/3).

Procedure B:

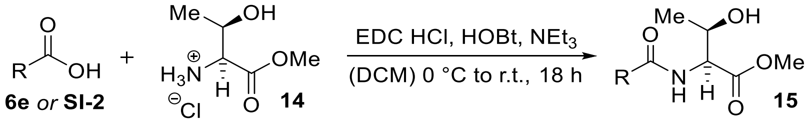

In a flame-dried 50 mL Schlenk flask, methyl L-threoninate hydrochloride (1.1 eq.) was dissolved in dry DCM (2 mL/mmol) and cooled to 0 °C, before NEt_3 (2.0 eq.) was added. After stirring for 10 minutes, the corresponding acid (1.0 eq.), EDC hydrochloride (2.0 eq.) and HOBt (2.0 eq.) were added. The mixture was stirred at room temperature for 18 hours. After completion of the reaction monitored by TLC, the solution was diluted with DCM (20 mL), and successively washed with 0.1 M HCl_{aq} (15 mL) and brine (30 mL), before being dried over Na_2SO_4, filtered and concentrated under reduced pressure. The residue was purified by flash column chromatography.

methyl hexanoyl-L-threoninate (**15a**)

15a

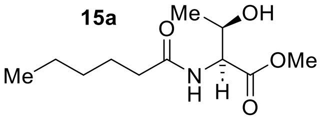

This compound was synthesized from hexanoic acid following procedure B, yielding 0.33 g (1.43 mmol, 68%) of a yellowish solid.

^{1}H-NMR (300 MHz, $CDCl_3$): δ [ppm] = 6.23 (d, *J* = 8.9 Hz, 1 H), 4.62 (dd, *J* = 8.8, 2.5 Hz, 1 H), 4.35 (dq, *J* = 6.4, 2.5 Hz, 1 H), 3.77 (s, 3 H), 2.32–2.24 (m, 2 H), 2.16 (bs, 1 H), 1.72–1.60 (m, 2 H), 1.38–1.28 (m, 4 H), 1.22 (d, *J* = 6.4 Hz, 3 H), 0.93–0.87 (m, 3 H).

***R*$_f$** (pentane/EtOAc = 1/2) = 0.40.

NMR data matched those reported in literature.[10]

methyl decanoyl-L-threoninate (**15c**)

15c

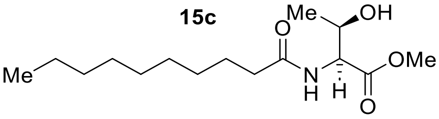

This compound was synthesized from decanoic acid following procedure B, yielding 6.94 g (24.2 mmol, 84%) of a white solid.

^{1}H-NMR (300 MHz, $CDCl_3$): δ [ppm] = 6.20 (bs, 1 H), 4.63 (dd, *J* = 8.6, 2.0 Hz, 1 H), 4.36 (dq, *J* = 6.4, 2.0 Hz, 1 H), 3.77 (s, 3 H), 2.29 (t, *J* = 7.5 Hz, 2 H), 2.24 (bs, 1 H), 1.65 (qnt, *J* = 7.5 Hz, 2 H), 1.39–1.24 (m, 12 H), 1.22 (d, *J* = 6.3 Hz, 3 H), 0.86 (t, *J* = 6.9 Hz, 3 H).

^{13}C {^{1}H}-NMR (75.5 MHz, $CDCl_3$)**:** δ [ppm] = 174.0, 171.9, 68.2, 57.1, 52.8, 36.8, 32.0, 29.6, 29.5, 29.4 (2C), 25.8, 22.8, 20.1, 14.3.

***R*$_f$** (pentane/EtOAc = 3/2) = 0.51.

HR-MS (ESI+)**:** *m/z* calcd. for $C_{15}H_{29}NO_4$ $[M+H]^+$: 288.2169, found: 288.2164.

methyl tetradecanoyl-L-threoninate (**15d**)

15d

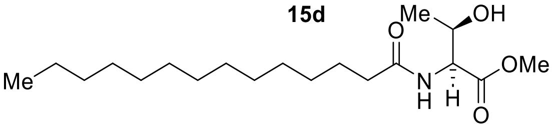

This compound was synthesized from myristoyl chloride following procedure A, yielding 2.26 g (6.6 mmol, 66%) of a white solid.
^{1}H-NMR (300 MHz, $CDCl_3$)**:** δ [ppm] = 6.16 (d, *J* = 8.8 Hz, 1 H), 4.63 (dd, *J* = 8.8, 2.5 Hz, 1 H), 4.35 (dq, *J* = 6.4, 2.5 Hz, 1 H), 3.77 (s, 3 H), 2.33–2.23 (m, 2 H), 1.72–1.58 (m, 2 H), 1.35–1.20 (m, 23 H), 0.91–0.84 (m, 3 H).
***R*$_f$** (pentane/EtOAc = 3/2) = 0.31.
NMR data matched those reported in literature.[10]

methyl ((*R*)-3-(methoxymethoxy)tetradecanoyl)-L-threoninate (**15e**)

15e

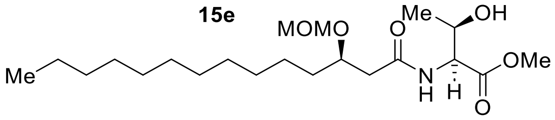

This compound was synthesized from (*R*)-3-(methoxymethoxy)tetradecanoic acid following procedure B, yielding 0.44 g of a white solid (1.1 mmol, 89%).
^{1}H-NMR (300 MHz, $CDCl_3$)**:** δ [ppm] = 6.89 (d, *J* = 8.9 Hz, 1 H), 4.70–4.58 (m, 2 H), 4.54 (dd, *J* = 8.9, 2.8 Hz, 1 H), 4.26 (dq, *J* = 6.4, 2.8 Hz, 1 H), 3.93 (qnt, *J* = 6.1 Hz, 1 H), 3.70 (s, 3 H), 3.32 (s, 3 H), 3.19 (bs, 1 H), 2.44 (d, *J* = 5.9 Hz, 2 H), 1.63–1.46 (m, 2 H), 1.34–1.17 (m, 18 H), 1.15 (d, *J* = 6.3 Hz, 3 H), 0.86–0.77 (m, 3 H).
^{13}C {^{1}H}-NMR (75.5 MHz, $CDCl_3$)**:** δ [ppm] = 171.7, 171.5, 96.1, 75.5, 67.9, 57.4, 55.7, 52.4, 41.9, 34.6, 31.9, 29.7 (2C), 29.6 (3C), 29.4, 25.3, 22.7, 20.0, 14.1.
***R*$_f$** (pentane/EtOAc = 2/3) = 0.25.
HR-MS (ESI+)**:** *m/z* calcd. for $C_{21}H_{41}NO_6$ $[M+Na]^+$: 426.2826, found: 426.2821.

3.1.3 Elimination of alcohol

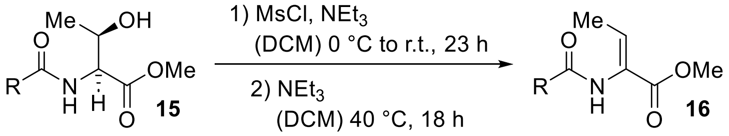

In a flame-dried 100 mL Schlenk flask, the corresponding alcohol (1.0 eq.) was dissolved in dry DCM (17 mL/mmol) and the solution cooled to 0 °C. Mesyl chloride (1.5 eq.) and NEt_3 (3.0 eq.) were added and the mixture stirred at room temperature for 18 hours. After addition of an additional portion of MsCl (0.75 eq.), the mixture was stirred for 5 hours, then diluted with DCM (50 mL) and quenched with water (50 mL). The organic phase was separated and washed with 1 M HCl_{aq} (50 mL) and 5% $NaHCO_3$ solution (50 mL), before being dried over Na_2SO_4, filtered and concentrated under reduced pressure. The residue was dissolved in DCM (17 mL/mmol), and NEt_3 (1.5 eq.) was added. The solution was heated to reflux for 18 hours to convert residual mesylate. After work-up (as described for the first step), the residue was purified by MPLC or column chromatography.

methyl (*Z*)-2-hexanamidobut-2-enoate (**16a**)

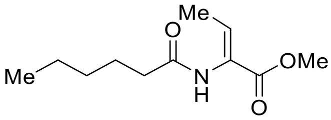

This compound was synthesized from methyl hexanoyl-L-threoninate, yielding 2.62 g (12.3 mmol, 67%) of a yellow oil after purification by column chromatography (pentane/EtOAc = 2/1).
^{1}H-NMR (300 MHz, $CDCl_3$)**:** δ [ppm] = 6.80 (bs, 1 H), 6.80 (q, *J* = 7.2 Hz, 1 H), 3.76 (s, 3 H), 2.33 (t, *J* = 7.5 Hz, 2 H), 1.77 (d, *J* = 7.1 Hz, 3 H), 1.74–1.63 (m, 2 H), 1.39–1.29 (m, 4 H), 0.94–0.86 (m, 3 H).

^{13}C {^{1}H}-NMR (75.5 MHz, $CDCl_3$)**:** δ [ppm] = 171.4, 165.3, 133.9, 126.0, 52.5, 37.0, 31.5, 25.5, 22.5, 15.1, 14.1.
***R*f** (pentane/EtOAc = 2/1) = 0.45.
HR-MS (ESI+)**:** *m/z* calcd. for $C_{11}H_{19}NO_3$ $[M+Na]^+$: 236.1257, found: 236.1251.

methyl (*Z*)-2-decanamidobut-2-enoate (**16c**)

16c

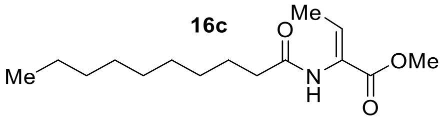

This compound was synthesized from methyl decanoyl-L-threoninate, yielding 4.25 g (15.8 mmol, 68%) of a white solid after purification by column chromatography (pentane/EtOAc = 2/1).
^{1}H-NMR (600 MHz, $CDCl_3$): δ [ppm] = 6.82 (bs, 1 H), 6.80 (q, *J* = 7.2 Hz, 1 H), 3.76 (s, 3 H), 2.32 (t, *J* = 7.6 Hz, 2 H), 1.76 (d, *J* = 7.2 Hz, 3 H), 1.68 (qnt, *J* = 7.5 Hz, 2 H), 1.38–1.32 (m, 2 H), 1.32–1.21 (m, 10 H), 0.87 (t, *J* = 7.0 Hz, 3 H).
^{13}C {^{1}H}-NMR (151 MHz, $CDCl_3$): δ [ppm] = 171.4, 165.3, 133.9, 125.9, 52.5, 37.0, 32.0, 29.6, 29.5, 29.4, 29.3, 25.8, 22.8, 15.1, 14.2.
***R*f** (pentane/EtOAc = 2/1) = 0.68.
HR-MS (ESI+)**:** *m/z* calcd. for $C_{15}H_{27}NO_3$ $[M+Na]^+$: 292.1883, found: 292.1879.

methyl (*Z*)-2-tetradecanamidobut-2-enoate (**16d**)

16d

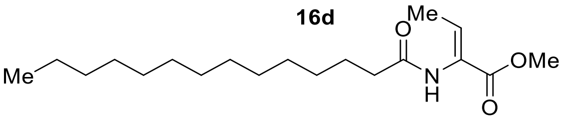

This compound was synthesized from methyl tetradecanoyl-L-threoninate, yielding 2.60 g (8.0 mmol, 71%) of a white solid after purification by MPLC. *E*/*Z* configuration was determined by a 2D NOESY-NMR experiment.
^{1}H-NMR (300 MHz, $CDCl_3$)**:** δ [ppm] = 6.81 (q, *J* = 7.1 Hz, 1 H), 6.77 (bs, 1 H), 3.77 (s, 3 H), 2.33 (t, *J* = 7.5 Hz, 2 H), 1.77 (d, *J* = 7.2 Hz, 3 H), 1.72–1.64 (m, 2 H), 1.35–1.23 (m, 20 H), 0.90–0.84 (m, 3 H).
^{13}C {^{1}H}-NMR (75.5 MHz, $CDCl_3$)**:** δ [ppm] = 171.5, 165.3, 134.0, 126.0, 52.5, 37.0, 32.0, 29.8, 29.8 (2C), 29.7, 29.6, 29.5 (2C), 29.4, 25.8, 22.8, 15.1, 14.3.
Important NOE contact (^{1}H-NMR shifts given in [ppm]):

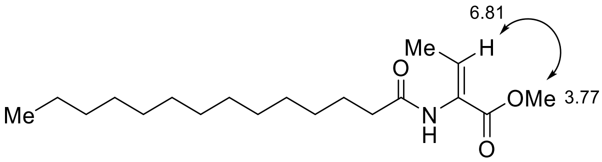

***R*f** (pentane/EtOAc = 3/1) = 0.58.
HR-MS (ESI+)**:** *m/z* calcd. for $C_{19}H_{35}NO_3$ $[M+Na]^+$: 348.2509, found: 348.2503.

methyl (*R*,*Z*)-2-(3-(methoxymethoxy)tetradecanamido)but-2-enoate (**16e**)

16e

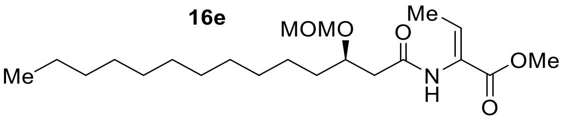

This compound was synthesized from methyl ((*R*)-3-(methoxymethoxy)tetradecanoyl)-L-threoninate, yielding 0.36 g of a white solid (0.93 mmol, 87%).
^{1}H-NMR (600 MHz, $CDCl_3$)**:** δ [ppm] = 7.54 (bs, 1 H), 6.77 (q, *J* = 7.2 Hz, 1 H), 4.75–4.68 (m, 2 H), 3.95 (dq, *J* = 6.3, 4.2 Hz, 1 H), 3,74 (s, 3 H), 3.38 (s, 3 H), 2.61 (dd, *J* = 15.0, 4.3 Hz, 1 H), 2.50 (dd, *J* = 15.0,

6.4 Hz, 1 H), 1.76 (d, J = 7.2 Hz, 3 H), 1.72–1.54 (m, 2 H), 1.37–1.31 (m, 2 H), 1.29–1.20 (m, 16 H), 0.86 (t, J = 7.0 Hz, 3 H).
^{13}C {^{1}H}-NMR (151 MHz, $CDCl_3$)**:** δ [ppm] = 169.3, 165.1, 133.7, 126.3, 96.3, 75.5, 55.9, 53.4, 42.1, 34.5, 32.0, 29.8, 29.7 (4C), 29.5, 25.5, 22.8, 14.8, 14.2.
Important NOE contact (^{1}H-NMR shifts given in [ppm]):

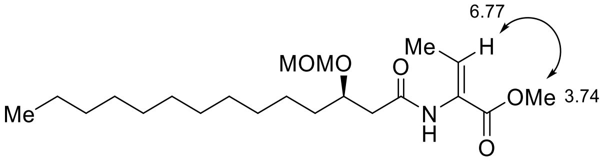

***R*f** (pentane/EtOAc = 1/1) = 0.55.
HR-MS (ESI+)**:** *m/z* calcd. for $C_{21}H_{39}NO_5$ $[M+Na]^+$: 408.2720, found: 408.2718.
Specific rotation: $[\alpha]_D = -1.9\ ^{\circ}\cdot{}^{mL}/_{dm\cdot g}$ (ρ = 0.75; $CHCl_3$).

3.1.4 Saponification of methyl ester

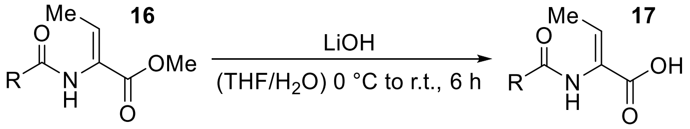

In a 100 mL round-bottomed flask, the corresponding ester (1.0 eq.) was dissolved in a mixture of THF and water (4/1, 10 mL/mmol). The solution was cooled to 0 °C and LiOH (2.5 eq.) was added. The mixture was stirred at 0 °C for 5 hours, then allowed to warm to room temperature and stirred for another hour. After dilution with 20 mL of water and 25 mL of Et_2O, the aqueous phase was separated and the organic phase extracted with water once. Combined aqueous phases were treated with EtOAc (25 mL), before being acidified by 1 M HCl_{aq} to a pH of around 3 under vigorous stirring. After extraction with EtOAc (3 × 25 mL), the combined organic extracts were washed with brine (50 mL), before being dried over Na_2SO_4, filtered and concentrated under reduced pressure. Unless stated otherwise, the product was used without further purification.

(*Z*)-2-hexanamidobut-2-enoic acid (**17a**)

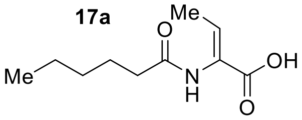

This compound was synthesized from methyl (*Z*)-2-hexanamidobut-2-enoate, yielding 1.97 g of a white solid (9.9 mmol, 90%).
^{1}H-NMR (300 MHz, DMSO-d_6)**:** δ [ppm] = 12.33 (bs, 1 H), 8.96 (s, 1 H), 6.48 (q, J = 7.0 Hz, 1 H), 2.19 (t, J = 7.4 Hz, 2 H), 1.63 (d, J = 7.0 Hz, 3 H), 1.51 (qnt, J = 7.3 Hz, 2 H), 1.33–1.21 (m, 4 H), 0.85 (t, J = 6.9 Hz, 3 H).
^{13}C {^{1}H}-NMR (75.5 MHz, DMSO-d_6)**:** δ [ppm] = 171.1, 165.7, 131.1, 128.9, 35.1, 30.8, 24.9, 21.9, 13.9, 13.5.
HR-MS (ESI+): *m/z* calcd. for $C_{10}H_{17}NO_3$ $[M+Na]^+$: 222.1101, found: 222.1099.

(*Z*)-2-decanamidobut-2-enoic acid (**17c**)

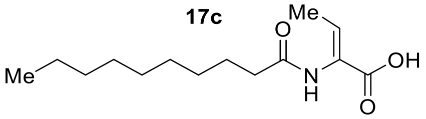

This compound was synthesized from methyl (*Z*)-2-decanamidobut-2-enoate, yielding 3.55 g of a white solid (13.9 mmol, 88%).
^{1}H-NMR (300 MHz, DMSO-*d*$_6$)**:** δ [ppm] = 12.35 (bs, 1 H), 8.95 (s, 1 H), 6.48 (q, *J* = 7.0 Hz, 1 H), 2.19 (t, *J* = 7.3 Hz, 2 H), 1.63 (d, *J* = 7.0 Hz, 3 H), 1.51 (qnt, *J* = 7.0 Hz, 2 H), 1.30–1.20 (m, 12 H), 0.84 (t, *J* = 7.0 Hz).
^{13}C {^{1}H}-NMR (75.5 MHz, DMSO-*d*$_6$)**:** δ [ppm] = 171.1, 165.7, 131.1, 128.9, 35.1, 31.3, 28.9, 28.8, 28.7, 28.6, 25.2, 22.1, 14.0, 13.5.
HR-MS (ESI+)**:** *m/z* calcd. for $C_{14}H_{25}NO_3$ [M+H]$^+$: 256.1907, found: 256.1903.

(*Z*)-2-tetradecanamidobut-2-enoic acid (**17d**)

17d

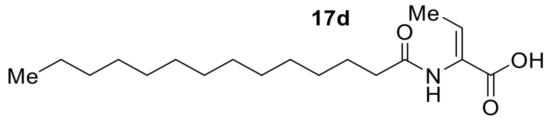

This compound was synthesized from methyl (*Z*)-2-tetradecanamidobut-2-enoate and purified by MPLC, yielding 0.38 g of a white solid (1.21 mmol, 79%).
^{1}H-NMR (300 MHz, DMSO-*d*$_6$)**:** δ [ppm] = 12.34 (bs, 1 H), 8.95 (s, 1 H), 6.48 (q, *J* = 6.9 Hz, 1 H), 2.19 (t, *J* = 7.3 Hz, 2 H), 1.63 (d, *J* = 7.0 Hz, 3 H), 1.56–1.45 (m, 2 H), 1.29–1.17 (m, 20 H), 0.88–0.80 (m, 3 H).
^{13}C {^{1}H}-NMR (75.5 MHz, DMSO-*d*$_6$)**:** δ [ppm] = 171.1, 165.7, 131.1, 128.8, 35.1, 31.3, 29.08, 29.06, 29.03 (2C), 28.98, 28.8, 28.7, 28.6, 25.2, 22.1, 14.0, 13.5.
HR-MS (ESI+)**:** *m/z* calcd. for $C_{18}H_{33}NO_3$ [M+Na]$^+$: 334.2353, found: 334.2347.

(*R*,*Z*)-2-(3-(methoxymethoxy)tetradecanamido)but-2-enoic acid (**17e**)

17e

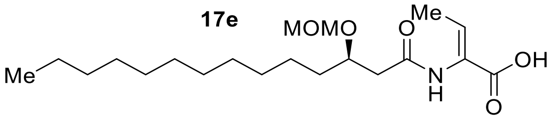

This compound was synthesized from methyl (*R*,*Z*)-2-(3-(methoxymethoxy)tetradecanamido)but-2-enoate, yielding 0.29 g of a white solid (0.79 mmol, 90%).
^{1}H-NMR (300 MHz, $CDCl_3$)**:** δ [ppm] = 9.33 (bs, 1 H), 7.67 (s, 1 H), 6.90 (q, *J* = 7.2 Hz, 1 H), 4.78–4.66 (m, 2 H), 4.05–3.91 (m, 1 H), 3.38 (s, 3 H), 2.65–2.48 (m, 2 H), 1.78 (d, *J* = 7.1 Hz, 3 H), 1.72–1.51 (m, 2 H), 1.38–1.19 (m, 18 H), 0.90–0.82 (m, 3 H).
^{13}C {^{1}H}-NMR (75.5 MHz, $CDCl_3$)**:** δ [ppm] = 169.8, 168.6, 136.1, 126.0, 96.2, 75.6, 55.9, 42.0, 34.5, 32.0, 29.8 (2C), 29.7 (3C), 29.5, 25.4, 22.8, 15.1, 14.2.
HR-MS (ESI+)**:** *m/z* calcd. for $C_{20}H_{37}NO_5$ [M+Na]$^+$: 394.2564, found: 394.2559.
Specific rotation: $[\alpha]_D = -5.3\ ^{\circ}\cdot mL/_{dm\cdot g}$ (ρ = 1.09; $CHCl_3$).

3.1.5 Synthesis of vinylprolinol building block

N-Boc-L-prolinal (**20**)

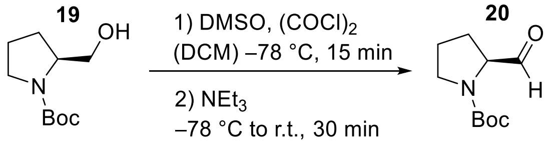

In a flame-dried 50 mL Schlenk flask, 0.78 mL (0.85 g, 10.9 mmol, 2.2 eq.) of dry DMSO was dissolved in 12 mL of dry DCM and cooled to –78 °C. 0.51 mL (0.78 g, 5.96 mmol, 1.2 eq.) oxalyl chloride was added slowly and the resulting solution was stirred for 5 minutes, before slow addition (15 minutes) of a solution of 1.0 g (4.97 mmol, 1.0 eq.) *N*-Boc-L-prolinol in 12 mL of dry DCM. The mixture was stirred for 15 minutes, before being treated with 2.75 mL (2.01 g, 19.8 mmol, 4.0 eq.) NEt_3. The solution was

allowed to warm to room temperature over 30 minutes. 30 mL of H_2O were added, and the aqueous phase was extracted with DCM (1 × 20 mL). Combined organic phases were washed with saturated $NaHCO_3$ solution (1 × 20 mL), 0.1 M HCl_{aq} (1 × 20 mL) and brine (1 × 20 mL), dried over Na_2SO_4, filtered and the solvents removed under reduced pressure. After drying under high vacuum, the product was obtained as a slight yellow oil (0.95 g, 4.77 mmol, 95%) and used without further purification.

^{1}H-NMR (300 MHz, $CDCl_3$)**:** δ [ppm] = 9.55 (d, *J* = 3.0 Hz, 0.4 H), 9.45 (d, *J* = 3.0 Hz, 0.6 H), 4.26–4.14 (m, 0.4 H), 4.10–4.00 (m, 0.6 H), 3.62–3.37 (m, 2 H), 2.17–1.81 (m, 4 H), 1.47 (s, 3.4 H), 1.42 (s, 5.6 H).

NMR spectrum showed a mixture of conformers.

Known compound, CAS 69610-41-9.

tert-butyl (*S*)-2-(1-hydroxyallyl)pyrrolidine-1-carboxylate (**21**)

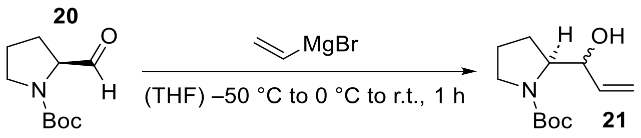

In a flame-dried 50 mL Schlenk flask, 0.50 g of *N*-Boc-L-prolinal (2.5 mmol, 1.0 eq.) was dissolved in 14 mL of dry THF and cooled to –50 °C. Then, 4.5 mL of a 1 M vinyl magnesium bromide solution in THF (4.5 mmol, 1.8 eq.) was added over 5 minutes and stirred for another 10 minutes at –50 °C. Afterwards, the mixture was stirred at 0 °C for 30 minutes and room temperature for 15 minutes. The reaction was quenched by addition of saturated NH_4Cl solution (30 mL) and the aqueous phase extracted with EtOAc (2 × 30 mL). Combined organic phases were washed with saturated $NaHCO_3$ solution (30 mL) and brine (30 mL), dried over Na_2SO_4, filtered and the solvents removed *in vacuo* to yield the final product as 0.54 g of a slight yellow oil (2.38 mmol, 95%) which was used without further purification.

^{1}H-NMR (300 MHz, $CDCl_3$)**:** δ [ppm] = 5.81 (dddd, *J* = 16.8, 10.4, 6.5, 2.6 Hz, 1 H), 5.36–5.26 (m, 1 H), 5.23–5.15 (m, 1 H), 4.23–3.79 (m, 2 H), 3.69 (bs, 1 H), 3.55–3.43 (m, 1 H), 3.36–3.15 (m, 1 H), 2.08–1.67 (m, 4 H), 1.47 (s, 4.4 H), 1.46 (s, 4.6 H).

NMR spectrum showed a mixture of isomers.

Known compound, CAS 220329-69-1.

(*S*)-1-(pyrrolidin-2-yl)prop-2-en-1-ol hydrochloride (**22**)

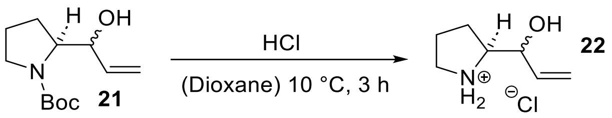

In a 25 mL flask, 0.54 g *tert*-butyl (*S*)-2-(1-hydroxyallyl)pyrrolidine-1-carboxylate (2.38 mmol, 1.0 eq.) was dissolved in 9 mL dioxane and cooled to 10 °C. Afterwards, the solution was treated with 3 mL of a 4 M HCl solution in dioxane (12 mmol, 5.0 eq.) and stirred for 2 hours. The mixture was diluted with 5 mL H_2O, the volatiles were removed under reduced pressure and the product was isolated by freeze drying as HCl salt as a brown solid (0.39 g, quant.).

^{1}H-NMR (300 MHz, DMSO-*d*$_6$)**:** δ [ppm] = 9.77 (bs, 0.5 H), 9.54 (bs, 0.4 H), 8.71 (bs, 0.9 H), 5.85 (dddd, *J* = 19.2, 10.4, 5.5, 3.2 Hz, 1 H), 5.41–5.30 (m, 1 H), 5.23–5.16 (m, 1 H), 4.42–4.36 (m, 0.5 H), 4.17–4.09 (m, 0.5 H), 3.59–3.46 (m, 1 H), 3.36–3.29 (m, 0.3 H), 3.17–3.03 (m, 2 H), 1.96–1.60 (m, 4 H).

^{13}C {^{1}H}-NMR (75.5 MHz, DMSO-*d*$_6$)**:** δ [ppm] = 137.5, 116.3, 69.2, 62.3, 45.2, 23.8, 23.6.

HR-MS (ESI+)**:** *m/z* calcd. for $C_7H_{13}NO$ $[M+H]^+$: 128.1070, found: 128.1067.

NMR spectra showed a mixture of isomers. Only ^{13}C signals of the major isomer are given.

tert-butyl (*S*)-2-(1-((*tert*-butyldimethylsilyl)oxy)allyl)pyrrolidine-1-carboxylate (**23**)

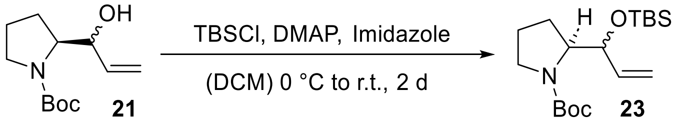

In a flame-dried 250 mL Schlenk flask, 2.90 g of *tert*-butyl (*S*)-2-(1-hydroxyallyl)pyrrolidine-1-carboxylate (12.8 mmol, 1.0 eq.) were dissolved in 80 mL of dry DCM, and 0.31 g DMAP (2.6 mmol, 0.2 eq.) and 0.97 g imidazole (14.2 mmol, 1.11 eq.) were added at 0 °C. After stirring the mixture for 15 minutes, 2.14 g TBSCl (14.2 mmol, 1.11 eq.) were added, and the mixture allowed to warm to room temperature. After 15 hours of stirring under argon, another 0.97 g imidazole (14.2 mmol, 1.11 eq.) and 1.07 g TBSCl (7.1 mmol, 0.55 eq.) were added, and the mixture stirred for 23 hours. The reaction was quenched by addition of 40 mL H_2O. The organic phase was separated, and the aqueous phase was extracted with DCM (3 × 30 mL), combined organic extracts washed with brine (40 mL), dried over Na_2SO_4, filtered and the volatiles removed under reduced pressure. The residue was purified by flash column chromatography (pentane/EtOAc = 40/1), yielding 3.91 g of a clear, yellowish oil (11.4 mmol, 89%). The product was obtained as a complex mixture of isomers, as observed by NMR spectroscopy.

^{1}H-NMR (300 MHz, $CDCl_3$)**:** δ [ppm] = 5.83–5.66 (m, 1 H), 5.31–5.04 (m, 2 H), 4.81–4.51 (m, 1 H), 3.95–3.59 (m, 1 H), 3.53–3.10 (m, 2 H), 2.04–1.59 (m, 4 H), 1.52–1.38 (m, 9 H), 0.92–0.82 (m, 9 H), 0.11–(-0.05) (m, 6 H).

^{13}C {^{1}H}-NMR (151 MHz, $CDCl_3$)**:** δ [ppm] = 154.1, 138.8, 114.4, 78.4, 72.2, 61.4, 47.1, 28.3 (3C), 25.6 (3C), 24.3, 23.8, 17.8, –5.2 (2C).

Only ^{13}C signals of the major isomer are given.

***R*$_f$** (pentane/EtOAc = 40/1) = 0.34.

HR-MS (ESI+)**:** *m/z* calcd. for $C_{18}H_{35}NO_3Si$ $[M+H]^+$: 342.2459, found: 342.2453.

(*S*)-2-(1-((tert-butyldimethylsilyl)oxy)allyl)pyrrolidine (**18**)[11]

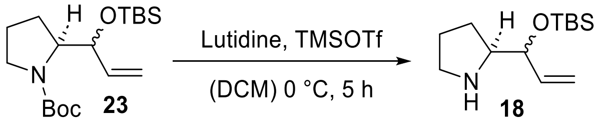

In a flame-dried 100 mL Schlenk flask, 0.70 g *tert*-butyl (*S*)-2-(1-((*tert*-butyldimethylsilyl)oxy)allyl)pyrrolidine-1-carboxylate (2.05 mmol, 1.0 eq.) was dissolved in 20 mL dry DCM and 1.91 mL lutidine (1.75 g, 16.4 mmol, 8.0 eq.) were added at 0 °C. Afterwards, 2.20 mL TMSOTf (2.71 g, 12.2 mmol, 6.0 eq.) were added to the reaction mixture and the solution was vigorously stirred at 0 °C under argon for 5 hours. The reaction was quenched by slow addition of saturated $NaHCO_3$ solution (30 mL). The organic phase was separated, and the aqueous phase was extracted with 25 mL DCM once. Combined organic phases were washed with brine (2 × 30 mL), dried over Na_2SO_4, filtered and the volatiles removed under reduced pressure. The residue was purified by flash column chromatography (DCM/MeOH = 10/1; 1 vol-% NEt_3) to yield the desired product as 0.48 g of an orange oil (1.96 mmol, 96%). The product was obtained as a complex mixture of isomers, as observed by NMR spectroscopy.

^{1}H-NMR (300 MHz, $CDCl_3$)**:** δ [ppm] = 6.03–5.71 (m, 1 H), 5.50–5.24 (m, 2 H), 4.52 (dd, *J* = 7.5, 5.1 Hz, 0.5 H), 4.38–4.32 (m, 0.4 H), 3.71–3.56 (m, 1 H), 3.49–3.24 (m, 2 H), 2.14–1.82 (m, 4 H), 0.94–0.85 (m, 9 H), 0.16–(-0.02) (m, 6 H).

^{13}C {^{1}H}-NMR (151 MHz, $CDCl_3$)**:** δ [ppm] = 136.0, 120.1, 73.1, 64.6, 47.1, 25.9 (3C), 25.3, 24.0, 18.1, –3.9, –4.9.

Only ^{13}C signals of the major isomer are given.

R_f (DCM/MeOH = 10/1) = 0.36.
HR-MS (ESI+): *m/z* calcd. for $C_{13}H_{27}NOSi$ $[M+H]^+$: 242.1935, found: 242.1931.

3.1.6 Synthesis of unsubstituted core structure (metathesis test system)

(*S*)-1-(2-(1-hydroxyallyl)pyrrolidin-1-yl)prop-2-en-1-one (**SI-3**)

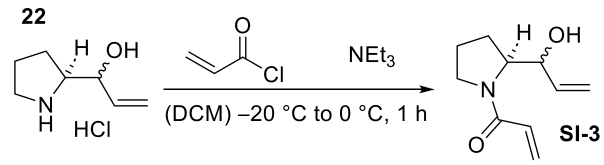

In a 100 mL round-bottomed flask, 0.30 g (*S*)-1-(pyrrolidin-2-yl)prop-2-en-1-ol hydrochloride (1.83 mmol, 1.05 eq.) was dissolved in dry DCM (9 mL/mmol), and 0.51 mL NEt_3 (0.37 g, 3.67 mmol, 2.0 eq.) was added. The mixture was cooled to –20 °C and 0.14 mL freshly distilled acryloyl chloride (0.16 g, 1.74 mmol, 1.0 eq.) was added slowly. The solution was stirred at 0 °C for one hour, before being quenched with saturated $NaHCO_3$ solution (30 mL). The aqueous phase was extracted with EtOAc (2 × 20 mL) and combined organic extracts were washed with brine (20 mL), dried over Na_2SO_4, filtered and concentrated under reduced pressure. The residue was purified by column chromatography (pentane/EtOAc = 1/2), yielding 0.16 g of a slight yellow oil (0.90 mmol, 52%). The product was obtained as a mixture of conformers/diastereomers.

^{1}H-NMR (300 MHz, $CDCl_3$): δ [ppm] = 6.74–5.96 (m, 2 H), 5.89–5.49 (m, 2 H), 5.43–4.78 (m, 3 H), 4.34–3.34 (m, 4 H), 2.13–1.64 (m, 4 H).

^{13}C {^{1}H}-NMR (75.5 MHz, $CDCl_3$): δ [ppm] = 166.6, 136.4, 128.6, 128.5, 116.8, 75.2, 63.9, 48.8, 27.6, 24.3.

Only ^{13}C signals of the major isomer are given.

R_f (pentane/EtOAc = 1/2) = 0.40.

HR-MS (ESI+): *m/z* calcd. for $C_{10}H_{15}NO_2$ $[M+Na]^+$: 204.0995, found: 204.0992.

(*S*)-8-hydroxy-2,3,8,8a-tetrahydroindolizin-5(1*H*)-one (**54**)[12]

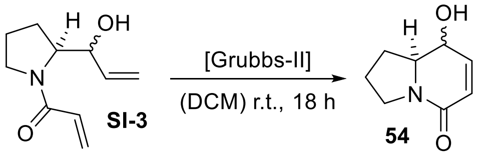

In a flame-dried 25 mL Schlenk flask, (*S*)-1-(2-(1-hydroxyallyl)pyrrolidin-1-yl)prop-2-en-1-one (164 mg, 0.91 mmol, 1.0 eq.) was dissolved in dry DCM (20 mL/mmol) and the solution flushed with argon for 15 minutes. Grubbs-II-catalyst (38.5 mg, 0.045 mmol, 5 mol-%) was added, and the mixture stirred at room temperature for 18 hours. Volatiles were removed under reduced pressure and the residue purified by MPLC, yielding 66.0 mg (0.43 mmol, 48%) of a white solid. The product was obtained as a mixture of diastereomers.

^{1}H-NMR (300 MHz, CD_3OD): δ [ppm] = 6.80 (dd, *J* = 9.7, 5.9 Hz, 0.4 H), 6.53 (dd, *J* = 10.0, 1.7 Hz, 0.6 H), 6.01 (d, *J* = 9.7 Hz, 0.4 H), 5.78 (dd, *J* = 9.9, 2.5 Hz, 0.6 H), 4.32 (ddd, *J* = 11.8, 2.4, 1.7 Hz, 0.6 H), 4.12 (dd, *J* = 5.9, 3.7 Hz, 0.4 H), 3.74–3.51 (m, 2 H), 3.47–3.34 (m, 1 H), 2.41–2.32 (m, 0.5 H), 2.20–1.98 (m, 1.9 H), 1.95–1.67 (m, 1.7 H).

^{13}C {^{1}H}-NMR (75.5 MHz, CD_3OD): δ [ppm] = 165.0, 147.8, 124.3, 72.2, 34.1, 45.7, 32.9, 23.6.

Only ^{13}C signals of the major isomer are given.

HR-MS (ESI+): *m/z* calcd. for $C_8H_{11}NO_2$ $[M+Na]^+$: 176.0682, found: 176.0676.

3.1.7 Amide coupling (with OH-unprotected amine 22)

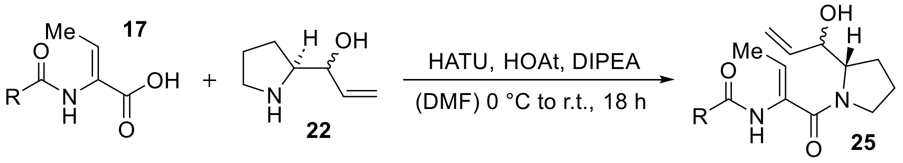

In a 50 mL Schlenk flask, the corresponding acid (1.0 eq.) was dissolved in dry DMF. The solution was cooled to 0 °C, before addition of HATU (2.0 eq.), HOAt (2.0 eq.) and DIPEA (4.0 eq.). In another 10 mL Schlenk flask, (*S*)-1-(pyrrolidin-2-yl)prop-2-en-1-ol hydrochloride (1.2 eq.) was dissolved in dry DMF. Then, the amine solution was added dropwise to the acid solution and the mixture stirred at room temperature under argon for 18 hours. The solution was diluted with EtOAc (20 mL), and successively washed with saturated $NaHCO_3$ solution (30 mL), 40% citric acid (30 mL) and brine (30 mL), before being dried over Na_2SO_4, filtered and concentrated under reduced pressure. The residue was purified by flash column chromatography or MPLC.

(*S*,*Z*)-*N*-(1-(2-(1-hydroxyallyl)pyrrolidin-1-yl)-1-oxobut-2-en-2-yl)tetradecanamide (**25d**)

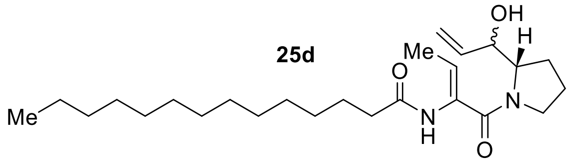

This compound was synthesized from (*Z*)-2-tetradecanamidobut-2-enoic acid and purified by column chromatography, yielding 142 mg (0.39 mmol, 28%) of a colorless oil. The product was obtained as mixture of isomers.

^{1}H-NMR (300 MHz, $CDCl_3$)**:** δ [ppm] = 8.87 (bs, 1 H), 5.78 (ddd, *J* = 16.6, 8.9, 3.7 Hz, 1 H), 5.40–5.09 (m, 3 H), 4.65 (d, *J* = 5.3 Hz, 0.3 H), 4.46 (d, *J* = 5.3 Hz, 0.6 H), 4.31–3.99 (m, 1 H), 3.91–3.65 (m, 1 H), 2.32–1.68 (m, 6 H), 1.61 (d, *J* = 7.0 Hz, 3 H), 1.58–1.47 (m, 2 H), 1.29–1.15 (m, 20 H), 0.86–0.78 (m, 3 H).

^{13}C {^{1}H}-NMR (75.5 MHz, $CDCl_3$)**:** δ [ppm] = 172.9, 169.4, 136.3, 132.0, 118.9, 116.2, 73.2, 63.0, 51.7, 35.7, 31.9, 29.7 (4C), 29.5, 29.4 (3C), 27.2, 25.4, 24.7, 22.7, 14.1, 12.2.

Only ^{13}C signals of the major isomer are given.

***R*$_f$** (pentane/EtOAc = 1/2) = 0.31.

HR-MS (ESI+)**:** *m/z* calcd. for $C_{25}H_{44}N_2O_3$ $[M+Na]^+$: 443.3244, found: 443.3240.

(*R*)-*N*-((*Z*)-1-((*S*)-2-(1-hydroxyallyl)pyrrolidin-1-yl)-1-oxobut-2-en-2-yl)-3-(methoxymethoxy)tetradecanamide (**25e**)

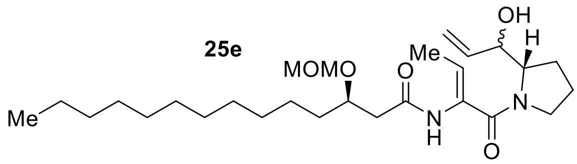

This compound was synthesized from (*R*,*Z*)-2-(3-(methoxymethoxy)tetradecanamido)but-2-enoic acid and purified by MPLC, yielding 80 mg (166 µmol, 29% brsm) of a colorless oil. The product was obtained as mixture of isomers.

^{1}H-NMR (300 MHz, $CDCl_3$)**:** δ [ppm] = 8.32 (bs, 1 H), 5.87–5.71 (m, 1 H), 5.48 (q, *J* = 7.2 Hz, 1 H), 5.40–5.29 (m, 1 H), 5.24–5.16 (m, 1 H), 4.76–4.61 (m, 3 H), 4.30–4.17 (m, 1 H), 3.99–3.70 (m, 2 H), 3.41–3.29 (m, 4 H), 2.59 (dd, *J* = 15.2, 4.0 Hz, 1 H), 2.48 (dd, *J* = 15.2, 7.0 Hz, 1 H), 2.08–1.82 (m, 3 H), 1.80–1.67 (m, 4 H), 1.65–1.46 (m, 2 H), 1.31–1.21 (m, 18 H), 0.90–0.82 (m, 3 H).

^{13}C {^{1}H}-NMR (75.5 MHz, $CDCl_3$)**:** δ [ppm] = 170.8, 168.4, 135.8, 130.8, 119.5, 116.8, 96.0, 75.2, 72.0, 63.2, 55.8, 51.5, 41.3, 34.4, 32.0, 29.7 (3C), 29.6, 29.4 (2C), 26.2, 25.3, 24.7, 22.8, 14.2, 12.0.
Only ^{13}C signals of the major isomer are given.
HR-MS (ESI+)**:** *m/z* calcd. for $C_{27}H_{48}N_2O_5$ $[M+Na]^+$: 503.3455, found: 503.3455.

3.1.8 Amide coupling (with OTBS-protected amine 18)

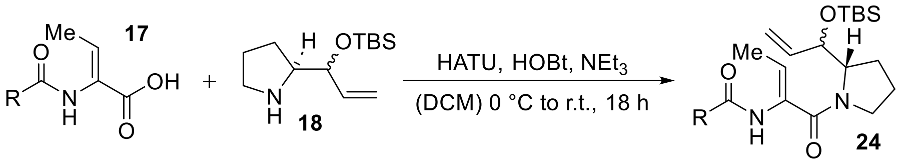

In a flame-dried Schlenk flask, (*S*)-2-(1-((tert-butyldimethylsilyl)oxy)allyl)pyrrolidine (1.1 eq.) was dissolved in dry DCM (10 mL/mmol acid), cooled to 0 °C, and NEt_3 (2.0 eq.) was added. After stirring for 10 minutes, the corresponding acid (1.0 eq.), HATU (1.2 eq.) and HOBt (1.2 eq.) were added, the mixture allowed to warm to room temperature and stirred under argon for 15 hours. After addition of an additional portion of HATU (0.6 eq.) and stirring for another 3 hours, the reaction was quenched by addition of H_2O (15 mL). The organic phase was separated, and the aqueous phase was extracted with DCM once (20 mL). Combined organic phases were washed with saturated NH_4Cl solution, saturated $NaHCO_3$ solution and brine (20 mL each), dried over Na_2SO_4, filtered and the volatiles removed under reduced pressure. The residue was purified by flash column chromatography to yield the desired amide.

(*S*,*Z*)-*N*-(1-(2-(1-((tert-butyldimethylsilyl)oxy)allyl)pyrrolidin-1-yl)-1-oxobut-2-en-2-yl)hexanamide (**24a**)

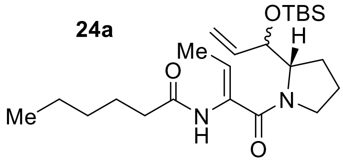

This compound was synthesized from (*Z*)-2-hexanamidobut-2-enoic acid, yielding 1.09 g (2.58 mmol, 37%) of a yellowish oil. The product was obtained as a mixture of diastereomers.
^{1}H-NMR (300 MHz, $CDCl_3$)**:** δ [ppm] = 8.50 (bs, 0.2 H), 8.04 (bs, 0.4 H), 5.88–5.68 (m, 1 H), 5.53 (q, *J* = 7.0 Hz, 0.5 H), 5.38 (q, *J* = 7.0 Hz, 0.4 H), 5.31–5.19 (m, 1 H), 5.15 (dt, *J* = 10.5, 1.9 Hz, 0.4 H), 5.09 (dt, *J* = 10.5, 1.8 Hz, 0.6 H), 4.81–4.67 (m, 0.9 H), 4.36–4.25 (m, 0.4 H), 4.12 (td, *J* = 7.4, 2.6 Hz, 0.6 H), 4.00–3.84 (m, 0.9 H), 3.38–3.15 (m, 1 H), 2.36–2.15 (m, 2 H), 2.13–1.98 (m, 1 H), 1.96–1.73 (m, 3.5 H), 1.72–1.59 (m, 5 H), 1.36–1.24 (m, 4 H), 0.94–0.83 (m, 12 H), 0.12–0.03 (m, 2.6 H), –0.02 (s, 1.7 H) –0.03 (s, 1.7 H).
^{13}C {^{1}H}-NMR (75.5 MHz, $CDCl_3$)**:** δ [ppm] = 172.2, 168.0, 139.1, 131.7, 121.6, 115.3, 72.5, 62.1, 51.6, 36.3, 31.6, 26.0 (3C), 25.4*, 25.0*, 23.6*, 22.5, 18.2, 14.1, 13.0, –4.2, –4.7.
Only ^{13}C signals of the major isomer are given.
*: Signals may result from minor isomer.
R_f (pentane/EtOAc = 3/1) = 0.13–0.26.
HR-MS (ESI+)**:** *m/z* calcd. for $C_{23}H_{42}N_2O_3Si$ $[M+H]^+$: 423.3038, found: 423.3032.

(*S*,*Z*)-*N*-(1-(2-(1-((tert-butyldimethylsilyl)oxy)allyl)pyrrolidin-1-yl)-1-oxobut-2-en-2-yl)decanamide (**24c**)

24c

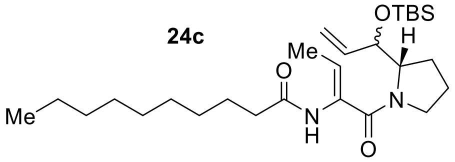

This compound was synthesized from (*Z*)-2-hexanamidobut-2-enoic acid, yielding 1.28 g (2.68 mmol, 44%) of a yellowish oil. The product was obtained as a mixture of diastereomers.

^{1}H-NMR (600 MHz, $CDCl_3$)**:** δ [ppm] = 8.49 (bs, 0.3 H), 8.02 (bs, 0.6 H), 5.84–5.71 (m, 1 H), 5.53 (q, *J* = 6.9 Hz, 0.6 H), 5.39 (q, *J* = 6.9 Hz, 0.4 H), 5.29–5.22 (m, 1 H), 5.16 (dt, *J* = 10.7, 1.8 Hz, 0.4 H), 5.09 (dt, *J* = 10.6, 1.5 Hz, 0.6 H), 4.78–4.70 (m, 1 H), 4.32 (td, *J* = 7.6, 4.7 Hz, 0.4 H), 4.13 (td, *J* = 7.8, 2.5 Hz, 0.6 H), 3.97–3.88 (m, 1 H), 3.31 (td, *J* = 9.9, 6.3 Hz, 0.6 H), 3.23 (td, *J* = 9.9, 6.7 Hz, 0.4 H), 2.34–2.21 (m, 2 H), 2.11–1.70 (m, 4 H), 1.69–1.57 (m, 5 H), 1.34–1.21 (m, 12 H), 0.90 (s, 9 H), 0.86 (t, *J* = 7.0 Hz, 3 H), 0.10 (s, 1.1 H), 0.05 (s, 1.1 H), –0.02 (s, 1.9 H), –0.03 (s, 1.9 H).

^{13}C {^{1}H}-NMR (151 MHz, $CDCl_3$)**:** δ [ppm] = 172.2, 168.0, 139.1, 131.6, 121.7, 115.3, 72.4, 62.1, 51.6, 36.4, 32.0, 29.6, 29.5 (2C), 29.4, 26.0 (3C), 25.7, 25.3, 23.6, 22.8, 18.1, 14.2, 13.1, –4.2, –4.7.

Only ^{13}C signals of the major isomer are given.

***R*$_f$** (pentane/EtOAc = 3/1) = 0.16–0.44.

HR-MS (ESI+)**:** *m/z* calcd. for $C_{27}H_{50}N_2O_3Si$ $[M+H]^+$: 479.3664, found: 479.3662.

(*S*,*Z*)-*N*-(1-(2-(1-((*tert*-butyldimethylsilyl)oxy)allyl)pyrrolidin-1-yl)-1-oxobut-2-en-2-yl)tetradecanamide (**24d**)

24d

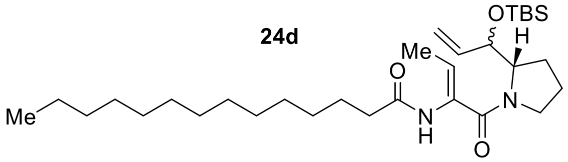

This compound was synthesized from (*Z*)-2-tetradecanamidobut-2-enoic acid, yielding 635 mg (1.19 mmol, 72%) of a yellowish oil. The product was obtained as a mixture of diastereomers.

^{1}H-NMR (300 MHz, $CDCl_3$)**:** δ [ppm] = 9.31 (bs, 0.2 H), 8.88 (bs, 0.5 H), 5.86–5.64 (m, 1 H), 5.38 (q, *J* = 6.8 Hz, 0.6 H), 5.31–5.17 (m, 1.3 H), 5.13 (dt, *J* = 10.5, 1.9 Hz, 0.4 H), 5.06 (dt, *J* = 10.4, 1.6 Hz, 0.6 H), 4.78–4.70 (m, 0.4 H), 4.69–4.62 (m, 0.5 H), 4.30 (td, *J* = 7.4, 4.6 Hz, 0.4 H), 4.13 (dt, *J* = 7.8, 3.8 Hz, 0.6 H), 4.02–3.86 (m, 1 H), 3.35–3.11 (m, 1 H), 2.37–2.11 (m, 2 H), 2.09–1.93 (m, 1 H), 1.91–1.71 (m, 3 H), 1.68–1.52 (m, 5 H), 1.32–1.15 (m, 20 H), 0.90–0.80 (m, 12 H), 0.07 (s, 1 H), 0.03 (s, 1 H), –0.04 (s, 2 H), –0.05 (s, 2 H).

^{13}C {^{1}H}-NMR (75.5 MHz, $CDCl_3$)**:** δ [ppm] = 172.8, 168.6, 139.1, 132.2, 120.5, 115.2, 72.7, 61.9, 51.5, 36.0, 32.0, 29.8 (3C), 29.7 (3C), 29.4 (2C), 25.9 (3C), 25.7, 25.1, 23.6, 22.7, 18.1, 14.2, 12.6, –4.2, –4.7.

Only ^{13}C signals of the major isomer are given.

***R*$_f$** (pentane/EtOAc = 3/1) = 0.37.

HR-MS (ESI+)**:** *m/z* calcd. for $C_{31}H_{58}N_2O_3Si$ $[M+H]^+$: 535.4289, found: 535.4280.

(*R*)-*N*-((*Z*)-1-((*S*)-2-(1-((*tert*-butyldimethylsilyl)oxy)allyl)pyrrolidin-1-yl)-1-oxobut-2-en-2-yl)-3-(methoxymethoxy)tetradecanamide (**24e**)

24e

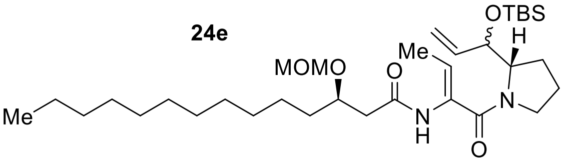

This compound was synthesized from (*R*,*Z*)-2-(3-(methoxymethoxy)tetradecanamido)but-2-enoic acid, yielding 1.65 g of a yellowish oil (2.77 mmol, 85%). The product was obtained as a mixture of diastereomers.

^{1}H-NMR (600 MHz, $CDCl_3$)**:** δ [ppm] = 9.10 (bs, 0.2 H), 8.88 (bs, 0.5 H), 5.77–5.68 (m, 1 H), 5.33 (q, *J* = 6.9 Hz, 0.6 H), 5.26–5.16 (m, 1.3 H), 5.08 (dt, *J* = 10.5, 1.9 Hz, 0.3 H), 5.02 (dt, *J* = 10.6, 1.7 Hz, 0.7 H), 4.74–4.70 (m, 0.3 H), 4.67–4.55 (m, 2.5 H), 4.25 (td, *J* = 7.6, 4.8 Hz, 0.3 H), 4.06 (dt, *J* = 7.7, 3.6 Hz, 0.6 H), 3.97–3.79 (m, 1.9 H), 3.28 (s, 1.9 H), 3.27 (s, 1.1 H), 3.26–3.12 (m, 1 H), 2.49 (dd, *J* = 14.6, 7.7 Hz, 0.9 H), 2.36 (dd, *J* = 14.6, 4.2 Hz, 0.9 H), 2.04–1.97 (m, 0.6 H), 1.91–1.84 (m, 0.4 H), 1.83–1.75 (m, 1 H), 1.75–1.66 (m, 2 H), 1.65–1.50 (m, 4 H), 1.50–1.43 (m, 1 H), 0.87–0.81 (m, 9 H), 0.80 (t, *J* = 7.0 Hz, 3 H), 0.04 (s, 1 H), –0.01 (s, 1 H), –0.05–(–0.11) (m, 4 H).

^{13}C {^{1}H}-NMR (151 MHz, $CDCl_3$)**:** δ [ppm] = 170.1, 168.1, 139.0, 132.2, 119.7, 115.0, 95.8, 75.0, 72.5, 61.8, 55.5, 51.4, 41.5, 34.7, 31.9, 29.6 (5C), 29.3, 25.8 (3C), 25.4, 25.0, 23.5, 22.7, 18.0, 14.1, 12.4, –4.3, –4.8.

***R*$_f$** (pentane/EtOAc = 5/2) = 0.24.

HR-MS (ESI+)**:** *m/z* calcd. for $C_{33}H_{62}N_2O_5Si$ $[M+H]^+$: 595.4501, found: 595.4496.

3.1.9 TBS deprotection

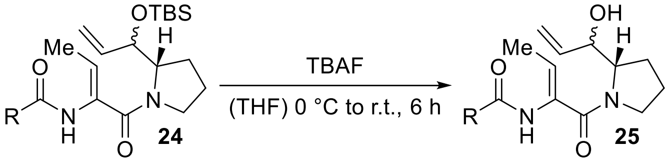

In a round-bottomed flask, TBS-protected alcohol (1.0 eq.) was dissolved in THF (so that all material was dissolved) and the solution cooled to 0 °C. Tetra-*n*-butylammonium fluoride (TBAF; 1 M in THF, 4.0 eq.) was added, and the solution stirred at room temperature. When the reaction was completed according to TLC, the mixture was diluted with DCM (20 mL) and quenched with water (10 mL). The organic phase was separated, and the aqueous phase was extracted with DCM once (20 mL). Combined organic phases were washed with brine (15 mL), dried over Na_2SO_4, filtered and the volatiles removed under reduced pressure. The residue was purified by column chromatography to yield the desired alcohol.

(*S*,*Z*)-*N*-(1-(2-(1-hydroxyallyl)pyrrolidin-1-yl)-1-oxobut-2-en-2-yl)hexanamide (**25a**)

25a

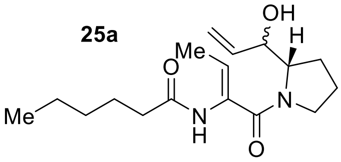

This compound was synthesized from (*S*,*Z*)-*N*-(1-(2-(1-((*tert*-butyldimethylsilyl)oxy)allyl)pyrrolidin-1-yl)-1-oxobut-2-en-2-yl)hexanamide, yielding 527 mg of a yellowish oil (1.71 mmol, 74%). The product was obtained as a mixture of diastereomers.

^{1}H-NMR (600 MHz, $CDCl_3$)**:** δ [ppm] = 8.29 (bs, 0.3 H), 8.21 (bs, 0.4 H), 5.88–5.77 (m, 1 H), 5.48–5.39 (m, 1 H), 5.38–5.31 (m, 1 H), 5.19 (dt, *J* = 10.5, 1.8 Hz, 1 H), 4.52 (bd, *J* = 5.4 Hz, 0.5 H), 4.30 (td, *J* = 8.0, 2.0 Hz, 0.5 H), 4.25 (q, *J* = 7.6 Hz, 0.4 H), 4.06–4.00 (m, 0.8 H), 3.88–3.83 (m, 0.5 H), 3.41–3.35 (m, 0.4 H), 3.34–3.27 (m, 0.6 H), 2.32–2.19 (m, 2 H), 2.09–1.99 (m, 1 H), 1.91–1.74 (m, 2.6 H), 1.68 (bd, *J* = 6.9 Hz, 3 H), 1.66–1.59 (m, 2.4 H), 1.34–1.27 (m, 4 H), 0.90–0.86 (m, 3 H).
^{13}C {^{1}H}-NMR (151 MHz, $CDCl_3$)**:** δ [ppm] = 172.6, 169.0, 136.3, 131.5, 120.4, 116.4, 78.2, 63.3, 51.8, 36.0, 31.5, 28.8, 25.2, 24.8, 22.5, 14.1, 12.6.
Only ^{13}C signals of the major isomer are given.
***R*$_f$** (pentane/EtOAc = 1/3) = 0.18–0.28.
HR-MS (ESI+)**:** *m/z* calcd. for $C_{17}H_{28}N_2O_3$ $[M+H]^+$: 309.2173, found: 309.2166.

(*S*,*Z*)-*N*-(1-(2-(1-hydroxyallyl)pyrrolidin-1-yl)-1-oxobut-2-en-2-yl)decanamide (**25c**)

25c

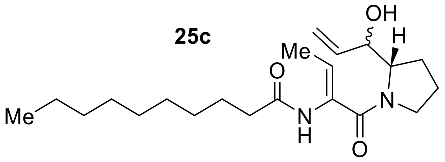

This compound was synthesized from (*S*,*Z*)-*N*-(1-(2-(1-((*tert*-butyldimethylsilyl)oxy)allyl)pyrrolidin-1-yl)-1-oxobut-2-en-2-yl)decanamide, yielding 697 mg of a colorless oil (1.91 mmol, 83%). The product was obtained as a mixture of diastereomers.
^{1}H-NMR (600 MHz, $CDCl_3$)**:** δ [ppm] = 8.17–7.85 (bs, 0.8 H), 5.87–5.76 (m, 1 H), 5.50 (bq, *J* = 7.1 Hz, 1 H), 5.40–5.31 (m, 1 H), 5.20 (bdt, *J* = 10.4, 1.8 Hz, 1 H), 4.58 (bd, *J* = 3.7 Hz, 0.6 H), 4.32–4.21 (m, 1 H), 4.07–3.98 (m, 0.7 H), 3.84–3.78 (0.5 H), 3.39 (dt, *J* = 10.3, 7.2 Hz, 0.4 H), 3.32 (dt, *J* = 10.4, 6.1 Hz, 0.6 H), 2.33–2.24 (m, 2 H), 2.07–1.99 (m, 1 H), 1.93–1.72 (m, 3 H), 1.72–1.68 (m, 2.7 H), 1.67–1.59 (m, 2.3 H), 1.34–1.20 (m, 12 H), 0.87 (t, *J* = 7.1 Hz, 3 H).
^{13}C {^{1}H}-NMR (151 MHz, $CDCl_3$)**:** δ [ppm] = 172.8, 169.3, 136.3, 131.9, 120.2, 116.4, 73.4, 63.2, 51.8, 35.9, 32.0, 29.6, 29.5, 29.4 (2C), 27.3, 25.5, 24.8, 22.8, 14.2, 12.3.
Only ^{13}C signals of the major isomer are given.
***R*$_f$** (pentane/EtOAc = 1/3) = 0.15–0.30.
HR-MS (ESI+)**:** *m/z* calcd. for $C_{21}H_{36}N_2O_3$ $[M+H]^+$: 365.2799, found: 365.2790.

(*S*,*Z*)-*N*-(1-(2-(1-hydroxyallyl)pyrrolidin-1-yl)-1-oxobut-2-en-2-yl)tetradecanamide (**25d**)

25d

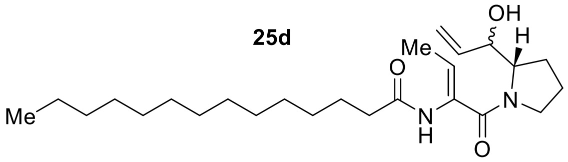

This compound was synthesized from (*S*,*Z*)-*N*-(1-(2-(1-((*tert*-butyldimethylsilyl)oxy)allyl)pyrrolidin-1-yl)-1-oxobut-2-en-2-yl)tetradecanamide, yielding 115 mg of a yellowish oil (273 µmol, 75%).
***R*$_f$** (pentane/EtOAc = 1/3) = 0.27.
Analytical data were in agreement with those of previously produced **25d** (chapter 3.1.7).

(*R*)-*N*-((*Z*)-1-((*S*)-2-(1-hydroxyallyl)pyrrolidin-1-yl)-1-oxobut-2-en-2-yl)-3-(methoxymethoxy)tetradecanamide (**25e**)

25e

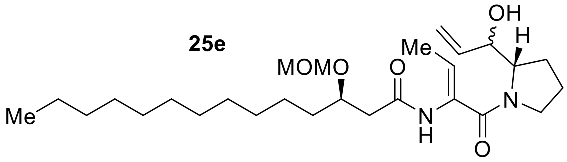

This compound was synthesized from (*R*)-*N*-((*Z*)-1-((*S*)-2-(1-((*tert*-butyl-dimethylsilyl)oxy)allyl) pyrrolidin-1-yl)-1-oxobut-2-en-2-yl)-3-(methoxymethoxy)tetradecanamide, yielding 1.23 g of a yellowish oil (2.55 mmol, 91%).

R

$\boldsymbol{R_f}$ (EtOAc) = 0.29.

Analytical data were in agreement with those of previously produced **25e** (chapter 3.1.7).

3.1.10 Ring closing metathesis

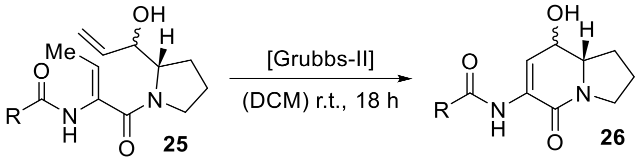

In a flame-dried 25 mL Schlenk flask, the corresponding alcohol (1.0 eq.) was dissolved in DCM (50 mL/mmol) and the solution purged with argon for 15 minutes. Afterwards, Grubbs 2nd generation catalyst (5 mol-%) was added and the mixture stirred under argon for 18 hours at room temperature. In case of incomplete conversion, as monitored by HPLC, another 2 mol-% of catalyst were added and stirred for 2 additional hours. The mixture was diluted with DCM (10 mL) and treated with 0.1 M HCl_{aq} (10 mL). The organic phase was separated, and the aqueous phase was extracted with DCM once (10 mL). Combined organic phases were washed with brine (20 mL), dried over Na_2SO_4, filtered, the volatiles removed under reduced pressure and the residue purified by column chromatography.

(*S*)-*N*-(8-hydroxy-5-oxo-1,2,3,5,8,8a-hexahydroindolizin-6-yl)hexanamide (**26a**)

26a

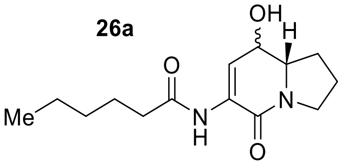

This compound was synthesized from (*S*,*Z*)-*N*-(1-(2-(1-hydroxyallyl)pyrrolidin-1-yl)-1-oxobut-2-en-2-yl)hexanamide, yielding 258 mg of a slightly brown solid (0.97 mmol, 58%). The product was obtained as mixture of diastereomers.

^{1}H-NMR (600 MHz, $CDCl_3$)**:** δ [ppm] = 8.17 (bs, 0.4 H), 8.05 (bs, 0.5 H), 7.45 (d, *J* = 6.5 Hz, 0.4 H), 7.17 (d, *J* = 1.9 Hz, 0.4 H), 4.48 (dd, *J* = 11.5, 1.9 Hz, 0.5 H), 4.27 (dd, *J* = 6.5, 3.5 Hz, 0.4 H), 3.75–3.39 (m, 3 H), 2.48–2.27 (m, 3 H)*, 2.26 –2.04 (m, 3 H)*, 2.03–1.95 (m, 1 H)*, 1.90–1.60 (m, 4 H)*, 1.38–1.27 (m, 4 H), 0.93–0.84 (m, 3 H).

*Due to overlapping signals of a small amount of compound **27a**, theoretical integrals are given.

^{13}C {^{1}H}-NMR (75.5 MHz, $CDCl_3$)**:** δ [ppm] = 172.5, 159.9, 127.3, 121.4, 71.2, 62.9, 45.2, 37.8, 31.9, 31.4, 25.3, 23.0, 22.5, 14.0.

In the ^{13}C-NMR spectrum, the signals of dehydrated compound **27a** are already visible due to elimination of water in the NMR tube. See chapter 4 for details. Signals were determined by comparison with spectrum of pure **27a**.

$\boldsymbol{R_f}$ (pentane/EtOAc = 1/2) = 0.35–0.41.

HR-MS (ESI+)**:** *m/z* calcd. for $C_{14}H_{22}N_2O_3$ $[M+H]^+$: 267.1704, found: 267.1699.

(*S*)-*N*-(8-hydroxy-5-oxo-1,2,3,5,8,8a-hexahydroindolizin-6-yl)decanamide (**26c**)

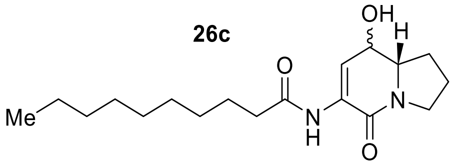

This compound was synthesized from (*S*,*Z*)-*N*-(1-(2-(1-hydroxyallyl)pyrrolidin-1-yl)-1-oxobut-2-en-2-yl)decanamide, yielding 269 mg of a slightly brown solid (0.83 mmol, 49%). The product was obtained as mixture of diastereomers.

^{1}H-NMR (300 MHz, $CDCl_3$)**:** δ [ppm] = 8.16 (bs, 0.4 H), 8.04 (bs, 0.5 H), 7.45 (d, *J* = 6.6 Hz, 0.4 H), 7.17 (d, *J* = 2.1 Hz, 0.5 H), 4.48 (dd, *J* = 11.5, 2.0 Hz, 0.5 H), 4.27 (dd, *J* = 6.6, 3.4 Hz, 0.4 H), 3.76–3.39 (m, 3 H), 2.47–2.26 (m, 3 H)*, 2.26–2.15 (m, 1 H)*, 2.14–1.96 (m, 3 H)*, 1.92–1.71 (m, 2 H)*, 1.71–1.59 (m, 2 H)*, 1.36–1.21 (m, 12 H), 0.91–0.83 (m, 3 H).

*Due to overlapping signals of a small amount of compound **27c**, theoretical integrals are given.

^{13}C {^{1}H}-NMR (75.5 MHz, $CDCl_3$)**:** δ [ppm] = 172.5, 159.8, 127.3, 121.3, 71.3, 62.9, 45.2, 37.8, 31.9, 29.6, 29.5, 29.4 (2C), 29.3, 25.6, 23.0, 22.8, 14.2.

In the ^{13}C NMR, the signals of dehydrated compound **27c** are already visible due to elimination of water in the NMR tube. See chapter 4 for details. Signals were determined by comparison with spectrum of pure **27c**.

***R*$_f$** (pentane/EtOAc = 1/2) = 0.42–0.50.

HR-MS (ESI+)**:** *m/z* calcd. for $C_{18}H_{30}N_2O_3$ $[M+H]^+$: 323.2330, found: 323.2322.

(*S*)-*N*-(8-hydroxy-5-oxo-1,2,3,5,8,8a-hexahydroindolizin-6-yl)tetradecanamide (**26d**)

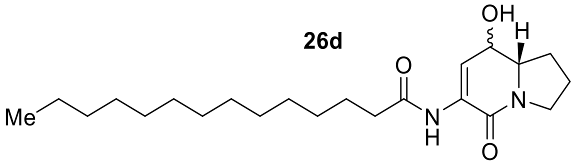

This compound was synthesized from (*S*,*Z*)-*N*-(1-(2-(1-hydroxyallyl)pyrrolidin-1-yl)-1-oxobut-2-en-2-yl)tetradecanamide, yielding 127 mg of a slightly brown solid (335 µmol, 67%). The product was obtained as mixture of diastereomers.

^{1}H-NMR (300 MHz, $CDCl_3$)**:** δ [ppm] = 8.15 (s, 0.1 H), 8.07 (s, 0.9 H), 7.43 (d, *J* = 6.5 Hz, 0.1 H), 7.22 (d, *J* = 1.8 Hz, 0.9 H), 4.46 (dd, *J* = 11.6, 1.9 Hz, 0.9 H), 4.24 (dd, *J* = 6.6, 3.4 Hz, 0.1 H), 4.04–3.40 (m, 4 H), 2.46–2.35 (m, 1 H), 2.30 (t, *J* = 7.6 Hz, 2 H), 2.10–2.00 (m, 1 H), 1.86–1.68 (m, 2 H), 1.68–1.57 (m, 2 H), 1.31–1.20 (m, 20 H), 0.90–0.82 (m, 3 H).

^{13}C {^{1}H}-NMR (75.5 MHz, $CDCl_3$)**:** δ [ppm] = 172.7, 159.8, 126.9, 122.0, 71.0, 62.8, 45.2, 37.8, 32.0 (2C), 29.8, 29.7 (3C), 29.6, 29.4 (2C), 29.3, 25.6, 22.9, 22.8, 14.2.

NMR spectra showed a mixture of isomers. Only ^{13}C signals of the major isomer are given.

***R*$_f$** (pentane/EtOAc = 2/3) = 0.50.

HR-MS (ESI+)**:** *m/z* calcd. for $C_{22}H_{38}N_2O_3$ $[M+Na]^+$: 401.2775, found: 401.2769.

(*R*)-*N*-((*S*)-8-hydroxy-5-oxo-1,2,3,5,8,8a-hexahydroindolizin-6-yl)-3-(methoxymethoxy)tetradecanamide (**26e**)

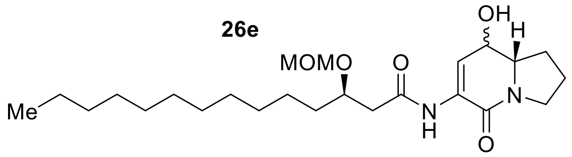

This compound was synthesized from (*R*)-*N*-((*Z*)-1-((*S*)-2-(1-hydroxyallyl)pyrrolidin-1-yl)-1-oxobut-2-en-2-yl)-3-(methoxymethoxy)tetradecanamide, yielding 1.20 g of a beige solid (2.74 mmol, 82%). The product was obtained as mixture of diastereomers.

^{1}H-NMR (600 MHz, $CDCl_3$)**:** δ [ppm] = 8.80 (bs, 0.1 H), 8.60 (bs, 0.8 H), 7.18 (d, *J* = 1.9 Hz, 0.9 H), 4.73 (s, 0.2 H), 4.70 (s, 1.8 H), 4.48 (dd, *J* = 11.5, 2.0 Hz, 0.9 H), 4.18 (dd, *J* = 8.0, 6.6 Hz, 0.1 H), 4.03–3.98 (m, 0.1 H), 3.95 (dq, *J* = 6.4, 4.2 Hz, 0.9 H), 3.66 (ddd, *J* = 11.9, 9.0, 2.6 Hz, 0.9 H), 3.59–3.54 (m, 0.9 H), 3.52–3.46 (m, 1 H), 3.37 (s, 0.4 H), 3.37 (s, 2.6 H), 2.64 (dd, *J* = 14.8, 4.3 Hz, 0.1 H), 2.60–2.55 (m, 1 H), 2.49 (dd, *J* = 14.9, 6.7 Hz, 0.9 H), 2.44–2.37 (m, 0.9 H), 2.11–2.04 (m, 1 H), 1.85–1.79 (m, 1 H), 1.78–1.71 (m, 1 H), 1.67–1.60 (m, 1 H), 1.57–1.50 (m, 1 H), 1.38–1.30 (m, 2 H), 1.30–1.23 (m, 16 H), 0.87 (t, *J* = 7.0 Hz, 3 H).

^{13}C {^{1}H}-NMR (151 MHz, $CDCl_3$)**:** δ [ppm] = 170.3, 159.8, 127.6, 121.3, 96.1, 75.1, 71.3, 62.9, 55.9, 45.2, 43.2, 34.6, 32.1, 31.9, 29.8 (2C), 29.7 (3C), 29.5, 25.5, 23.0, 22.8, 14.3.

Only ^{13}C shifts of the major isomer are given.

HR-MS (ESI+)**:** *m/z* calcd. for $C_{24}H_{42}N_2O_5$ $[M+Na]^+$: 461.2986, found: 461.2981.

3.1.11 'Swern elimination'

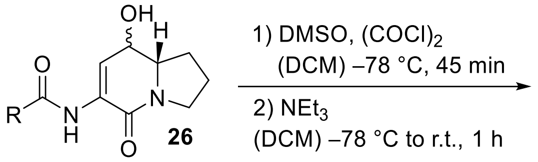

In a flame-dried 25 mL Schlenk flask, DMSO (3.0 eq.) was dissolved in dry DCM and the mixture cooled to –78 °C. Oxalyl chloride (2.2 eq.) was added slowly, and the mixture stirred for 10 minutes at –78 °C. Afterwards, a solution of the alcohol (1.0 eq.) in dry DCM was added slowly, and the mixture stirred at –78 °C for 45 minutes. NEt_3 (4.0 eq.) was added, and the cooling bath removed to allow the mixture to warm to room temperature. After stirring for 1 hour, the solution was poured onto water (15 mL) and the aqueous phase was extracted with DCM (2 × 10 mL). Combined organic extracts were washed with 0.1 M HCl_{aq}, saturated $NaHCO_3$ solution and brine (10 mL each), dried over Na_2SO_4, filtered and the solvents removed under reduced pressure. The residue was purified by column chromatography to yield the desired product.

N-(5-oxo-1,2,3,5-tetrahydroindolizin-6-yl)hexanamide (**27a**)

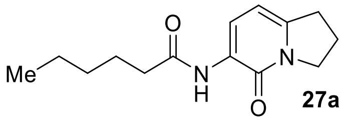

This compound was synthesized from (*S*)-*N*-(8-hydroxy-5-oxo-1,2,3,5,8,8a-hexahydroindolizin-6-yl)hexanamide, yielding 141 mg (0.57 mmol, 62%) of a slightly brown solid.

^{1}H-NMR (400 MHz, $CDCl_3$)**:** δ [ppm] = 8.39 (d, *J* = 7.5 Hz, 1 H), 8.24 (bs, 1 H), 6.16 (dt, *J* = 7.5, 1.3 Hz, 1 H), 4.17 (bt, *J* = 7.3 Hz, 2 H), 3.06 (td, *J* = 7.7, 1.3 Hz, 2 H), 2.38 (t, *J* = 7.6 Hz, 2 H), 2.21 (qnt, *J* = 7.6 Hz, 2 H), 1.76–1.67 (m, 2 H), 1.38–1.30 (m, 4 H), 0.94–0.87 (m, 3 H).

^{13}C {^{1}H}-NMR (151 MHz, $CDCl_3$)**:** δ [ppm] = 172.4, 156.6, 142.6, 126.9, 123.7, 101.8, 49.3, 37.9, 31.5, 31.3, 25.4, 22.6, 22.0, 14.1.

$\boldsymbol{R_f}$ (pentane/EtOAc = 1/4) = 0.30.

HR-MS (ESI+)**:** *m/z* calcd. for $C_{14}H_{20}N_2O_2$ $[M+Na]^+$: calcd. 271.1417, found: 271.1415.

N-(5-oxo-1,2,3,5-tetrahydroindolizin-6-yl)decanamide (**27c**)

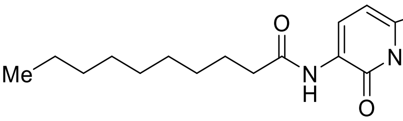

27c

This compound was synthesized from (*S*)-*N*-(8-hydroxy-5-oxo-1,2,3,5,8,8a-hexahydroindolizin-6-yl)decanamide, yielding 196 mg (0.64 mmol, 77%) of an off-white solid.

^{1}H-NMR (300 MHz, $CDCl_3$)**:** δ [ppm] = 8.41 (d, *J* = 7.6 Hz, 1 H), 8.40 (bs, 1 H), 6.18 (dt, *J* = 7.6, 1.7 Hz, 1 H), 4.21–4.14 (m, 2 H), 3.07 (bt, *J* = 7.5 Hz, 2 H), 2.39 (bt, *J* = 7.6 Hz, 2 H), 2.21 (qnt, *J* = 7.7 Hz, 2 H), 1.70 (qnt, *J* = 7.4 Hz, 2 H), 1.40 –1.19 (m, 12 H), 0.90–0.82 (m, 3 H).

^{13}C {^{1}H}-NMR (75.5 MHz, $CDCl_3$)**:** δ [ppm] = 172.4, 156.5, 142.6, 126.9, 123.7, 101.8, 49.3, 37.9, 32.0, 31.3, 29.6, 29.5, 29.4 (2C), 25.7, 22.8, 22.0, 14.2.

***R*$_f$** (pentane/EtOAc = 2/3) = 0.29.

HR-MS (ESI+)**:** *m/z* calcd. for $C_{18}H_{28}N_2O_2$ $[M+Na]^+$: calcd. 327.2043, found: 327.2043.

N-(5-oxo-1,2,3,5-tetrahydroindolizin-6-yl)tetradecanamide (**27d**)

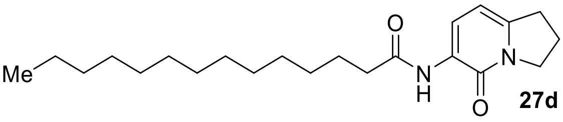

27d

This compound was synthesized from (*S*)-*N*-(8-hydroxy-5-oxo-1,2,3,5,8,8a-hexahydroindolizin-6-yl)tetradecanamide, yielding 118 mg (348 µmol, 80%) of an off-white solid.

^{1}H-NMR (600 MHz, $CDCl_3$)**:** δ [ppm] = 8.40 (bs, 1 H), 8.40 (d, *J* = 7.6 Hz, 1 H), 6.17 (dt, *J* = 7.5, 1.5 Hz, 1 H), 4.18–4.15 (m, 2 H), 3.06 (td, *J* = 7.7, 1.3 Hz, 2 H), 2.38 (t, *J* = 7.6 Hz, 2 H), 2.20 (qnt, *J* = 7.6 Hz, 2 H), 1.68 (qnt, *J* = 7.6 Hz, 2 H), 1.36–1.31 (m, 2 H), 1.30–1.21 (m, 18 H), 0.86 (t, *J* = 7.0 Hz, 3 H).

^{13}C {^{1}H}-NMR (151 MHz, $CDCl_3$)**:** δ [ppm] = 172.5, 156.4, 142.7, 126.8, 124.1, 102.1, 49.4, 37.8, 32.0, 31.2, 29.8, 29.7 (3C), 29.6, 29.5 (2C), 29.3, 25.7, 22.8, 22.0, 14.2.

***R*$_f$** (pentane/EtOAc = 1/3) = 0.34.

HR-MS (ESI+)**:** *m/z* calcd. for $C_{22}H_{36}N_2O_2$ $[M+H]^+$: 361.2850, found: 361.2842.

(*R*)-3-(methoxymethoxy)-*N*-(5-oxo-1,2,3,5-tetrahydroindolizin-6-yl)tetradecanamide (**27e**)

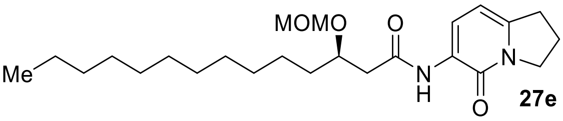

27e

This compound was synthesized from (*R*)-*N*-((*S*)-8-hydroxy-5-oxo-1,2,3,5,8,8a-hexahydroindolizin-6-yl)-3-(methoxymethoxy)tetradecanamide, yielding 633 mg (1.49 mmol, 91%) of a yellowish oil.

^{1}H-NMR (600 MHz, $CDCl_3$)**:** δ [ppm] = 8.75 (bs, 1 H), 8.36 (d, *J* = 7.5 Hz, 1 H), 6.11 (dt, *J* = 7.5, 1.4 Hz, 1 H), 4.70 (s, 2 H), 4.15–4.12 (m, 2 H), 4.00–3.95 (m, 1 H), 3.34 (s, 3 H), 3.03 (td, *J* = 7.7, 1.4 Hz, 2 H), 2.61 (dd, *J* = 14.8, 4.8 Hz, 1 H), 2.54 (dd, *J* = 14.8, 6.8 Hz, 1 H), 2.17 (qnt, *J* = 7.6 Hz, 2 H), 1.66–1.59 (m, 1 H), 1.57–1.50 (m, 1 H), 1.37–1.30 (m, 2 H), 1.27–1.19 (m, 16 H), 0.84 (t, *J* = 7.4 Hz, 3 H).

^{13}C {^{1}H}-NMR (151 MHz, $CDCl_3$)**:** δ [ppm] = 169.9, 156.7, 142.5, 126.9, 123.3, 101.2, 96.0, 75.1, 55.8, 49.1, 43.3, 34.6, 32.0, 31.2, 29.7 (3C), 29.6 (2C), 29.4, 25.4, 22.8, 22.0, 14.2.

***R*$_f$** (pentane/EtOAc = 1/2) = 0.22.

HR-MS (ESI+)**:** *m/z* calcd. for $C_{24}H_{40}N_2O_4$ $[M+H]^+$: 421.3061, found: 421.3052.

Specific rotation: $[\alpha]_D = -3.8\ ^{\circ}\cdot {}^{mL}/_{dm\cdot g}$ (ρ = 1.47; $CHCl_3$).

3.1.12 Halogenations

Brominations

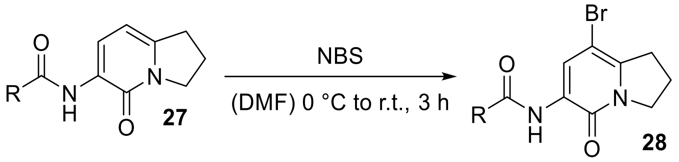

In a flame-dried 10 mL Schlenk tube, the corresponding tetrahydroindolizin (1.0 eq.) was dissolved in dry DMF (20 mL/mmol) and the mixture cooled to 0 °C. *N*-Bromosuccinimide (1.4 eq.) was added in one portion and the solution allowed to warm to room temperature. After stirring under argon for 3 hours, the reaction mixture was diluted with DCM (10 mL) and quenched by addition of 10 mL of H_2O. The organic phase was separated, and the aqueous phase was extracted once with DCM (20 mL). Combined organic extracts were washed with brine (20 mL), dried over Na_2SO_4, filtered and the solvents removed under reduced pressure (DCM) and high vacuum (DMF). The residue was purified by flash column chromatography to yield the desired brominated product.

N-(8-bromo-5-oxo-1,2,3,5-tetrahydroindolizin-6-yl)tetradecanamide (**28d**)

28d

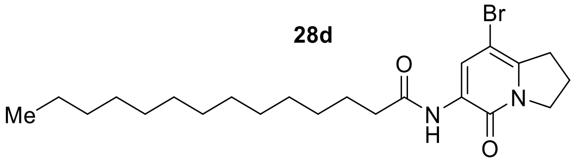

This compound was synthesized from *N*-(5-oxo-1,2,3,5-tetrahydroindolizin-6-yl)tetradecanamide, yielding 24 mg of a beige solid (54.6 µmol, 79%).

^{1}H-NMR (300 MHz, $CDCl_3$)**:** δ [ppm] = 8.55 (s, 1 H), 8.23 (bs, 1 H), 4.25 (bt, *J* = 7.4 Hz, 2 H), 3.09 (t, *J* = 7.8 Hz, 2 H), 2.38 (t, *J* = 7.5 Hz, 2 H), 2.24 (qnt, *J* = 7.7 Hz, 2 H), 1.69 (qnt, *J* = 7.3 Hz, 2 H), 1.38–1.21 (m, 20 H), 0.86 (t, *J* = 7.0 Hz, 3 H).

^{13}C {^{1}H}-NMR (151 MHz, $CDCl_3$)**:** δ [ppm] = 172.4, 155.6, 140.8, 127.8, 126.0, 95.3, 50.8, 37.8, 32.7, 32.1, 29.8 (3C), 29.7, 29.6, 29.5 (2C), 29.3, 25.6, 22.8, 21.2, 14.3.

***R*$_f$** (pentane/EtOAc = 1/1) = 0.32.

HR-MS (ESI+)**:** *m/z* calcd. for $C_{22}H_{35}BrN_2O_2$ $[M+H]^+$: 439.1955, 441.1935; found: 439.1943, 441.1955.

(*R*)-*N*-(8-bromo-5-oxo-1,2,3,5-tetrahydroindolizin-6-yl)-3-(methoxymethoxy)tetradecanamide (**28e**)

28e

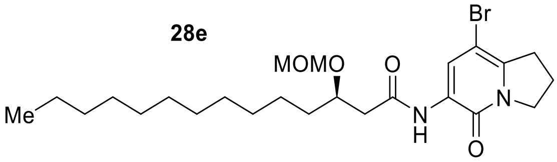

This compound was synthesized from (*R*)-3-(methoxymethoxy)-*N*-(5-oxo-1,2,3,5-tetrahydroindolizin-6-yl)tetradecanamide, yielding 44 mg of an orange oil (88.1 µmol, 84%).

^{1}H-NMR (300 MHz, $CDCl_3$)**:** δ [ppm] = 8.78 (bs, 1 H), 8.53 (s, 1 H), 4.72 (s, 2 H), 4.26–4.19 (m, 2 H), 4.02–3.93 (m, 1 H), 3.36 (s, 3 H), 3.07 (t, *J* = 7.7 Hz, 2 H), 2.65 (dd, *J* = 14.9, 4.4 Hz, 1 H), 2.56 (dd, *J* = 14.9, 6.6 Hz, 1 H), 2.23 (p, *J* = 7.7 Hz, 2 H), 1.69–1.47 (m, 2 H), 1.38–1.29 (m, 2 H), 1.29–1.19 (m, 16 H), 0.86 (t, *J* = 7.0 Hz, 3 H).

^{13}C {^{1}H}-NMR (75.5 MHz, $CDCl_3$)**:** δ [ppm] = 170.2, 155.6, 140.8, 128.0, 126.0, 96.1, 95.0, 75.1, 55.9, 50.7, 43.2, 34.6, 32.7, 32.0, 29.7 (5C), 29.5, 25.5, 22.8, 21.2, 14.2.

***R*$_f$** (pentane/EtOAc = 2/3) = 0.26.

HR-MS (ESI+)**:** *m/z* calcd. for $C_{24}H_{39}BrN_2O_4$ $[M+H]^+$: 499.2166, 501.2146, found: 499.2151, 501.2136.

Specific rotation: $[\alpha]_D = -7.2\ ^\circ\cdot{}^{mL}/_{dm\cdot g}$ (ρ = 0.84; $CHCl_3$).

Iodinations

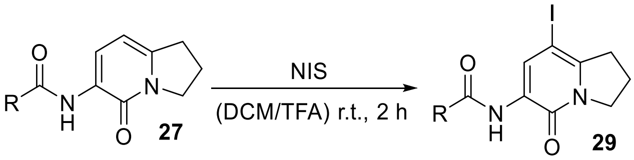

In a flame-dried 10 mL Schlenk tube, the *N*-(5-oxo-1,2,3,5-tetrahydroindolizin-6-yl)amide (1.0 eq.) was dissolved in dry DCM and TFA (3/1). *N*-Iodosuccinimide (1.2 eq.) was added, and the mixture stirred at room temperature under argon for 2 hours. Afterwards, the pink solution was diluted with DCM (10 mL) and quenched by addition of saturated $NaHCO_3$ solution (15 mL). The organic phase was separated, and the aqueous phase was extracted once with DCM (10 mL). Combined organic phases were washed with half-saturated $Na_2S_2O_3$ solution (10 mL) and brine (10 mL), dried over Na_2SO_4, filtered and the volatiles removed under reduced pressure. The residue was purified by flash column chromatography.

N-(8-iodo-5-oxo-1,2,3,5-tetrahydroindolizin-6-yl)hexanamide (**29a**)

29a

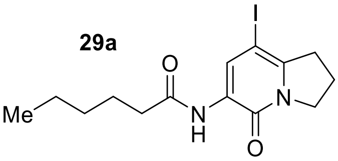

This compound was synthesized from *N*-(5-oxo-1,2,3,5-tetrahydroindolizin-6-yl)hexanamide, yielding 154 mg of a yellowish solid (412 µmol, 77%).

^{1}H-NMR (300 MHz, $CDCl_3$)**:** δ [ppm] = 8.63 (s, 1 H), 8.22 (bs, 1 H), 4.27 (bt, *J* = 7.4 Hz, 2 H), 3.05 (t, *J* = 7.8 Hz, 2 H), 2.36 (t, *J* = 7.5 Hz, 2 H), 2.21 (qnt, *J* = 7.8 Hz, 2 H), 1.71–1.64 (m, 2 H), 1.35–1.28 (m, 4 H), 0.89–0.85 (m, 3 H).

^{13}C {^{1}H}-NMR (75.5 MHz, $CDCl_3$)**:** δ [ppm] = 172.2, 155.9, 144.2, 130.1, 127.8, 63.1, 51.1, 37.7, 25.9, 31.4, 25.2, 22.5, 20.9, 14.0.

R_f (pentane/EtOAc = 1/1) = 0.31.

HR-MS (ESI+)**:** *m/z* calcd. for $C_{14}H_{19}IN_2O_2$ $[M+H]^+$: 375.0564, found: 375.0562.

N-(8-iodo-5-oxo-1,2,3,5-tetrahydroindolizin-6-yl)decanamide (**29c**)

29c

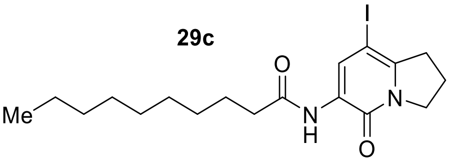

This compound was synthesized from *N*-(5-oxo-1,2,3,5-tetrahydroindolizin-6-yl)decanamide, yielding 238 mg of a yellowish solid (553 µmol, 89%).

^{1}H-NMR (300 MHz, $CDCl_3$)**:** δ [ppm] = 8.65 (s, 1 H), 8.20 (bs, 1 H), 4.33–4.26 (m, 2 H), 3.07 (t, *J* = 7.8 Hz, 2 H), 2.38 (t, *J* = 7.5 Hz, 2 H), 2.23 (qnt, *J* = 7.7 Hz, 2 H), 1.69 (qnt, *J* = 7.4 Hz, 2 H), 1.38–1.22 (m, 12 H), 0.90–0.84 (m, 3 H).

^{13}C {^{1}H}-NMR (75.5 MHz, $CDCl_3$)**:** δ [ppm] = 172.2, 156.0, 144.3, 130.1, 127.9, 63.1, 51.2, 37.9, 36.0, 32.0, 29.5 (2C), 29.4, 29.3, 25.6, 22.8, 21.0, 14.2.

R_f (pentane/EtOAc = 1/1) = 0.45.

HR-MS (ESI+)**:** *m/z* calcd. for $C_{18}H_{27}IN_2O_2$ $[M+H]^+$: 431.1190, found: 431.1187.

N-(8-iodo-5-oxo-1,2,3,5-tetrahydroindolizin-6-yl)tetradecanamide (**29d**)

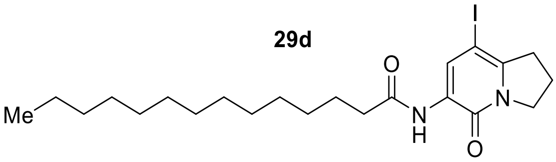

This compound was synthesized from *N*-(5-oxo-1,2,3,5-tetrahydroindolizin-6-yl)tetradecanamide, yielding 58 mg of a beige solid (119 µmol, 86%).

^{1}H-NMR (600 MHz, $CDCl_3$)**:** δ [ppm] = 8.64 (s, 1 H), 8.22 (bs, 1 H), 4.30–4.26 (m, 2 H), 3.05 (t, *J* = 7.8 Hz, 2 H), 2.37 (t, *J* = 7.6 Hz, 2 H), 2.22 (qnt, *J* = 7.7 Hz, 2 H), 1.67 (qnt, *J* = 7.5 Hz, 2 H), 1.35–1.30 (m, 2 H), 1.30–1.21 (m, 18 H), 0.86 (t, *J* = 7.0 Hz, 3 H).

^{13}C {^{1}H}-NMR (151 MHz, $CDCl_3$)**:** δ [ppm] = 172.2, 155.9, 144.2, 130.1, 127.8, 63.1, 51.1, 37.8, 36.0, 32.0, 29.8, 29.7 (3C), 29.6, 29.5 (2C), 29.3, 25.6, 22.8, 21.0, 14.2.

***R*$_f$** (pentane/EtOAc = 1/1) = 0.38.

HR-MS (ESI+)**:** *m/z* calcd. for $C_{22}H_{35}IN_2O_2$ $[M+H]^+$: 487.1816, 488.1850; found: 487.1804, 488.1837.

(*R*)-1-((8-iodo-5-oxo-1,2,3,5-tetrahydroindolizin-6-yl)amino)-1-oxotetradecan-3-yl 2,2,2-trifluoroacetate (**30**)

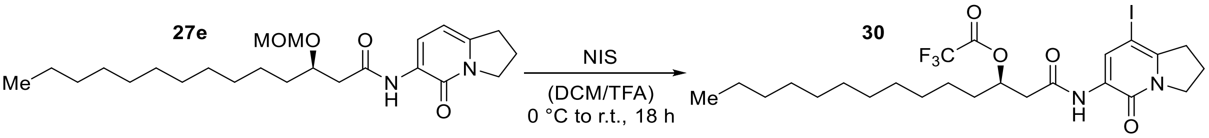

In a flame-dried 10 mL Schlenk tube, 40 mg (*R*)-3-(methoxymethoxy)-*N*-(5-oxo-1,2,3,5-tetrahydro-indolizin-6-yl)tetradecanamide (95.1 µmol, 1.0 eq.) was dissolved in 3 mL of dry DCM. After cooling to 0 °C, 1 mL TFA and 32.1 mg NIS (143 µmol, 1.5 eq.) were added. The solution was allowed to warm to room temperature and stirred under argon for 18 hours. The pink solution was diluted with DCM (10 mL) and quenched by addition of saturated $NaHCO_3$ solution (15 mL). The organic phase was separated, and the aqueous phase was extracted once with DCM (10 mL). Combined organic phases were washed with half-saturated $Na_2S_2O_3$ solution (10 mL) and brine (10 mL), dried over Na_2SO_4, filtered and the volatiles removed under reduced pressure. The residue was purified by flash column chromatography, yielding 41 mg of a beige solid (68.5 µmol, 58%).

^{1}H-NMR (600 MHz, $CDCl_3$)**:** δ [ppm] = 8.59 (s, 1 H), 8.38 (bs, 1 H), 5.54 (tt, *J* = 7.4, 5.2 Hz, 1 H), 4.31–4.26 (m, 2 H), 3.07 (t, *J* = 7.8 Hz, 2 H), 2.82 (dd, *J* = 15.6, 7.8 Hz, 1 H), 2.75 (dd, *J* = 15.6, 4.9 Hz, 1 H), 2.23 (p, *J* = 7.7 Hz, 2 H), 1.78–1.71 (m, 2 H), 1.36–1.31 (m, 2 H), 1.29–1.21 (m, 16 H), 0.87 (t, *J* = 7.0 Hz, 3 H).

^{13}C {^{1}H}-NMR (151 MHz, $CDCl_3$)**:** δ [ppm] = 167.4, 157.0 (q, *J* = 42.0 Hz) 155.8, 145.0, 130.8, 127.4, 114.6 (q, *J* = 286 Hz), 75.8, 62.9, 51.2, 41.4, 36.1, 33.8, 32.0, 29.7 (2C), 29.6, 29.5, 29.4, 29.3, 25.0, 22.8, 20.9, 14.3.

^{19}F {^{1}H}-NMR (282 MHz, $CDCl_3$): δ [ppm] = –74.5.

***R*$_f$** (pentane/EtOAc = 1/1) = 0.45.

HR-MS (ESI+)**:** *m/z* calcd. for $C_{24}H_{34}F_3IN_2O_4$ $[M+Na]^+$: 621.1408, found: 621.1409.

Specific rotation: $[\alpha]_D$ = +4.4 $^{\circ}\cdot {}^{mL}/_{dm\cdot g}$ (ρ = 2.05; $CHCl_3$).

(*R*)-3-hydroxy-*N*-(8-iodo-5-oxo-1,2,3,5-tetrahydroindolizin-6-yl)tetradecanamide (**29e**)

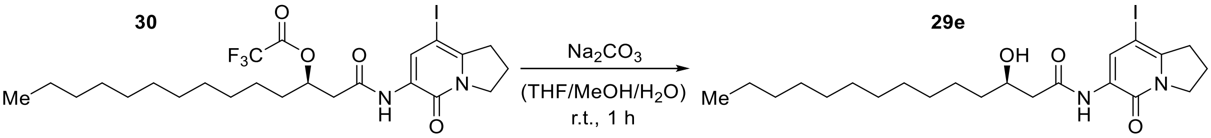

In a flame-dried 10 mL Schlenk tube, 41 mg (*R*)-1-((8-iodo-5-oxo-1,2,3,5-tetrahydroindolizin-6-yl)amino)-1-oxotetradecan-3-yl 2,2,2-trifluoroacetate (68.5 µmol, 1.0 eq.) was dissolved in a mixture of THF, MeOH and H_2O (1.6 mL / 0.2 mL / 0.2 mL) and 29 mg sodium carbonate (274 µmol, 4.0 eq.) was added. The mixture was stirred at room temperature for 1 hour and afterwards separated between EtOAc and H_2O (5 mL each). The organic phase was separated, and the aqueous phase extracted with EtOAc (3 × 10 mL). Combined organic extracts were washed with brine (15 mL), dried over Na_2SO_4, filtered and the solvents removed under reduced pressure. The residue was purified by column chromatography to yield 24.4 mg of an orange wax (48.6 µmol, 71%).

^{1}H-NMR (600 MHz, $CDCl_3$)**:** δ [ppm] = 8.69 (bs, 1 H), 8.59 (s, 1 H), 4.32–4.23 (m, 2 H), 4.10–4.05 (m, 1 H), 3.15 (bs, 1 H), 3.12–3.02 (m, 2 H), 2.56 (dd, *J* = 15.5, 2.8 Hz, 1 H), 2.49 (dd, *J* = 15.5, 9.0 Hz, 1 H), 2.26–2.20 (m, 2 H), 1.59–1.51 (m, 1 H), 1.50–1.41 (m, 1 H), 1.36–1.30 (m, 2 H), 1.30–1.22 (m, 16 H), 0.87 (t, *J* = 7.0 Hz, 3 H).

^{13}C {^{1}H}-NMR (151 MHz, $CDCl_3$)**:** δ [ppm] = 171.5, 155.8, 144.7, 130.6, 127.6, 68.7, 63.0, 51.2, 44.1, 37.1, 36.0, 32.0, 29.8 (2C), 29.7 (3C), 29.5, 25.6, 22.8, 21.0, 14.3.

***R*$_f$** (pentane/EtOAc = 1/2) = 0.26.

HR-MS (ESI+)**:** *m/z* calcd. for $C_{22}H_{35}IN_2O_3$ $[M+H]^+$: 503.1765, found: 503.1751.

Specific rotation: $[\alpha]_D = -8.4\ ^{\circ\cdot mL}/_{dm\cdot g}$ (ρ = 1.22; $CHCl_3$).

3.1.13 Hydroxylation

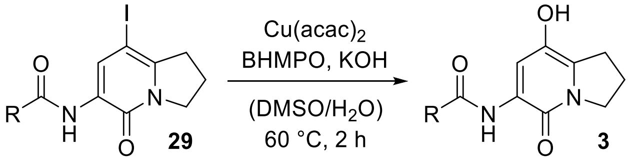

In an oven-dried 10 mL Schlenk tube, the corresponding iodide (1.0 eq.) was dissolved/suspended in DMSO and degassed H_2O (7/1), and the mixture degassed by bubbling with argon for 15 minutes. Afterwards, $Cu(acac)_2$ (20 mol-%), *N^1*,*N^2*-bis(4-hydroxy-2,6-dimethylphenyl)oxalamide (BHMPO, 20 mol-%) and KOH (3.0 eq.) were added quickly, and the resulting mixture heated to 60 °C under argon while being vigorously stirred. Progress of the reaction was tracked by HPLC. After 2 hours, the mixture was cooled and acidified by 0.1 M HCl_{aq}. The slurry was diluted with DCM (10 mL) and the organic phase was separated. The aqueous phase was extracted with DCM (2 × 10 mL). Combined organic extracts were washed with brine (10 mL), dried over Na_2SO_4, filtered and the volatiles removed under reduced pressure. The residue was purified *via* preparative HPLC.

N-(8-hydroxy-5-oxo-1,2,3,5-tetrahydroindolizin-6-yl)hexanamide (**3a**)

3a

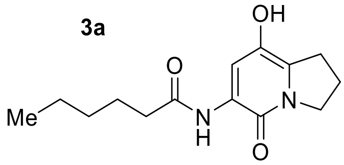

This compound was synthesized from *N*-(8-iodo-5-oxo-1,2,3,5-tetrahydroindolizin-6-yl)hexanamide, yielding 9.4 mg of a white solid (35.6 µmol, 51%).

^{1}H-NMR (600 MHz, DMSO-*d*$_6$)**:** δ [ppm] = 8.99 (bs, 1 H), 8.62 (s, 1 H), 8.18 (s, 1 H), 4.01–3.97 (m, 2 H), 2.93 (t, *J* = 7.6 Hz, 2 H), 2.40 (t, *J* = 7.5 Hz, 2 H), 2.08 (qnt, *J* = 7.6 Hz, 2 H), 1.54 (qnt, *J* = 7.5 Hz, 2 H), 1.32–1.21 (m, 4 H), 0.86 (t, *J* = 7.1 Hz, 3 H).

^{13}C {^{1}H}-NMR (151 MHz, DMSO-*d*$_6$)**:** δ [ppm] = 171.9, 152.9, 133.4, 126.3, 126.0, 117.8, 49.1, 36.2, 30.9, 27.7, 25.0, 21.9, 21.5, 13.9.

HR-MS (ESI+)**:** *m/z* calcd. for $C_{14}H_{20}N_2O_3$ calcd. for $[M+Na]^+$: 287.1367, found: 287.1367.

N-(8-hydroxy-5-oxo-1,2,3,5-tetrahydroindolizin-6-yl)decanamide (**3c**)

3c

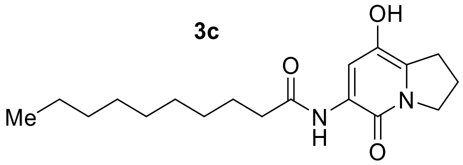

This compound was synthesized from *N*-(8-iodo-5-oxo-1,2,3,5-tetrahydroindolizin-6-yl)decanamide, yielding 10.0 mg of a white solid (31.2 µmol, 54%).

^{1}H-NMR (600 MHz, DMSO-d_6)**:** δ [ppm] = 8.98 (s, 1 H), 8.62 (s, 1 H), 8.17 (s, 1 H), 3.99 (dd, *J* = 8.0, 6.6 Hz, 2 H), 2.93 (t, *J* = 7.6 Hz, 2 H), 2.40 (t, *J* = 7.4 Hz, 2 H), 2.08 (qnt, *J* = 7.5 Hz, 2 H), 1.53 (qnt, *J* = 6.8 Hz, 2 H), 1.28–1.21 (m, 12 H), 0.85 (t, *J* = 7.0 Hz, 3 H).

^{13}C {^{1}H}-NMR (151 MHz, DMSO-d_6)**:** δ [ppm] = 171.9, 152.9, 133.4, 126.3, 126.0, 117.8, 49.1, 36.2, 31.3, 28.9, 28.8, 28.7, 28.6, 27.7, 25.3, 22.1, 21.5, 14.0.

HR-MS (ESI+)**:** *m/z* calcd. for $C_{18}H_{28}N_2O_3$ $[M+Na]^+$: 343.1993, found: 343.1991.

N-(8-hydroxy-5-oxo-1,2,3,5-tetrahydroindolizin-6-yl)tetradecanamide (**3d**)

3d

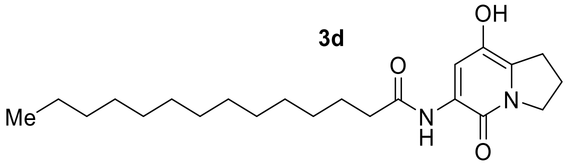

This compound was synthesized from *N*-(8-iodo-5-oxo-1,2,3,5-tetrahydroindolizin-6-yl)tetradecane-amide, yielding 9.2 mg of a white solid (24.4 µmol, 49%).

^{1}H-NMR (600 MHz, DMSO-d_6)**:** δ [ppm] = 8.99 (s, 1 H), 8.62 (bs, 1 H), 8.17 (s, 1 H), 4.01–3.97 (m, 2 H), 2.93 (t, *J* = 7.6 Hz, 2 H), 2.40 (t, *J* = 7.4 Hz, 2 H), 2.08 (qnt, *J* = 7.5 Hz, 2 H), 1.52 (bqnt, *J* = 7.0 Hz, 2 H), 1.28–1.25 (m, 2 H), 1.25–1.22 (m, 18 H), 0.85 (t, *J* = 6.9 Hz, 3 H).

^{13}C {^{1}H}-NMR (151 MHz, DMSO-d_6)**:** δ [ppm] = 171.9, 152.9, 133.4, 126.3, 126.0, 117.8, 49.1, 36.2, 31.3, 29.10, 29.06, 29.04 (2C), 28.96, 28.81, 28.75, 28.6, 27.7, 25.3, 22.1, 21.5, 14.0.

HR-MS (ESI+)**:** *m/z* calcd. for $C_{22}H_{36}N_2O_3$ $[M+H]^+$: 377.2799, found: 377.2802.

(*R*)-3-hydroxy-*N*-(8-hydroxy-5-oxo-1,2,3,5-tetrahydroindolizin-6-yl)tetradecanamide (**3e**)

3e

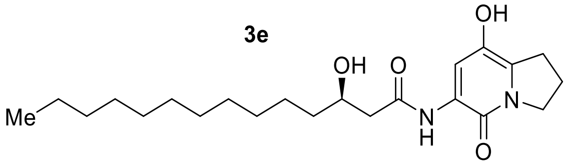

This compound was synthesized from (*R*)-3-hydroxy-*N*-(8-iodo-5-oxo-1,2,3,5-tetrahydroindolizin-6-yl)tetradecanamide, yielding 9.0 mg of a white solid (22.9 µmol, 40%).

^{1}H-NMR (600 MHz, DMSO-d_6)**:** δ [ppm] = 9.28 (s, 1 H), 8.62 (s, 1 H), 8.20 (s, 1 H), 4.93 (d, *J* = 5.4 Hz, 1 H), 4.00–3.97 (m, 2 H), 3.83–3.77 (m, 1 H), 2.93 (t, *J* = 7.6 Hz, 2 H), 2.44 (dd, *J* = 14.5, 4.5 Hz, 1 H), 2.41 (dd, *J* = 14.5, 7.5 Hz, 1 H), 2.08 (qnt, *J* = 7.7 Hz, 2 H), 1.38–1.33 (m, 2 H), 1.29–1.25 (m, 2 H), 1.25–1.22 (m, 16 H), 0.85 (t, *J* = 6.9 Hz, 3 H).

^{13}C {^{1}H}-NMR (151 MHz, DMSO-d_6)**:** δ [ppm] = 170.4, 152.8, 133.5, 126.4, 125.9, 117.4, 67.4, 49.1, 44.7, 37.1, 31.3, 29.1 (3C), 29.0 (2C), 28.8, 27.7, 25.1, 22.1, 21.5, 14.0.

HR-MS (ESI+)**:** *m/z* calcd. for $C_{22}H_{36}N_2O_4$ $[M+Na]^+$: 415.2567, found: 415.2575.

Specific rotation: $[\alpha]_D = -7.7\ ^{\circ}\cdot {}^{mL}/_{dm\cdot g}$ (ρ = 0.39; MeOH).

3.1.14 Synthesis of putative natural BraC substrate 3f

3.1.14.1 Synthesis of activated rhamnose building block **31**

(3*R*,4*R*,5*S*,6*S*)-6-methyltetrahydro-2*H*-pyran-2,3,4,5-tetrayl tetrabenzoate (**33**)[13]

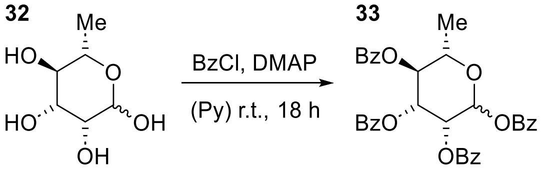

In a flame-dried 250 mL Schlenk flask, 3.0 g L-rhamnose (16.5 mmol, 1.0 eq.) was dissolved in 30 mL dry pyridine. 86 mg of DMAP (0.70 mmol, 5 mol-%) were added, followed by dropwise addition of benzoyl chloride (9.64 mL, 11.6 g, 82.5 mmol, 5.0 eq.). The resulting mixture was stirred at room temperature for 18 hours and quenched by addition of 30 mL water. After extraction with DCM (2 × 40 mL); combined organic phases were washed with saturated NH_4Cl solution (30 mL) and brine (2 × 30 mL), dried over Na_2SO_4, filtered and the solvents removed under reduced pressure. The residue was purified by flash column chromatography (pentane/EtOAc = 8/1) to yield the product as a white foam (7.84 g, 13.5 mmol, 82%) as a mixture of anomers (ratio ~11:4).

^{1}H-NMR (300 MHz, $CDCl_3$)**:** δ [ppm] = 8.22–8.12 (m, 3.6 H), 8.01–7.92 (m, 2.4 H), 7.86–7.82 (m, 1.9 H), 7.70–7.62 (m, 1.9 H), 7.58–7.49 (m, 4.9 H), 7.47–7.43 (m, 1 H), 7.42–7.38 (m, 2 H), 7.37–7.33 (m, 0.5 H), 7.31–7.26 (m, 1.9 H), 6.55 (d, *J* = 1.9 Hz, 0.7 H), 6.35 (d, *J* = 1.2 Hz, 0.3 H), 6.08–6.06 (m, 0.3 H), 5.99 (dd, *J* = 10.2, 3.5 Hz, 0.7 H), 5.86 (dd, *J* = 3.5, 2.0 Hz, 0.3 H), 5.80 (t, *J* = 10.0 Hz, 0.7 H), 5.74–5.70 (m, 0.5 H), 4.38–4.32 (m, 0.7 H), 4.14–4.06 (m, 0.3 H), 1.48 (d, *J* = 6.2 Hz, 0.8 H), 1.41 (d, *J* = 6.2 H, 2.2 H).

***R*$_f$** (pentane/EtOAc = 8/1) = 0.52.

NMR data matched literature values.[13]

(3*R*,4*R*,5*S*,6*S*)-2-hydroxy-6-methyltetrahydro-2*H*-pyran-3,4,5-triyl tribenzoate (**34**)[13]

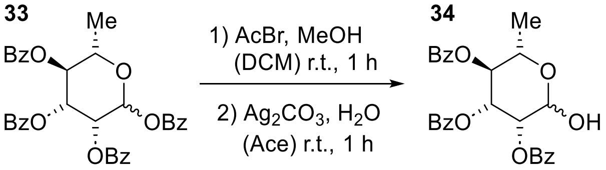

In a flame-dried 100 mL Schlenk flask, 3.0 g tetrabenzoyl-L-rhamnose (5.17 mmol, 1.0 eq.) was dissolved in 30 mL dry DCM. 1.88 mL dry MeOH (1.50 g, 46.6 mmol, 9.0 eq.) and 3.82 mL acetyl bromide (6.34 g, 51.7 mmol, 10 eq.) were added slowly, and the mixture stirred at room temperature for 1 hour. The mixture was treated with 20 mL water and the aqueous phase extracted with Et_2O (2 × 20 mL). Combined organic phases were dried over Na_2SO_4, filtered and the solvents removed under reduced pressure to yield the crude bromide which was used without further purification. This residue was dissolved in 16 mL of acetone and 0.40 mL of water was added. After addition of 7.12 g Ag_2CO_3 (25.8 mmol, 5.0 eq.), the mixture was stirred at room temperature for 1 hour. The slurry was filtered and the solid washed with acetone. The volatiles were removed under reduced pressure and the residue purified by MPLC to yield the desired product as a white solid (1.51 g, 3.17 mmol, 61%) as a mixture of anomers (ratio ~13:2; determined by ^{1}H-NMR).

^{1}H-NMR (300 MHz, $CDCl_3$)**:** δ [ppm] = 8.15–8.07 (m, 2 H), 8.02–7.92 (m, 2 H), 7.87–7.77 (m, 2 H), 7.65–7.57 (m, 1 H), 7.56–7.35 (m, 6 H), 7.31–7.22 (m, 2 H)*, 5.96–5.86 (m, 1 H), 5.74–5.55 (m, 2 H), 5.46 (bs, 0.9 H), 5.23 (bs, 0.1 H), 4.46 (dq, *J* = 9.7, 6.2 Hz, 0.9 H), 3.96–3.86 (m, 0.1 H), 3.71 (bs, 0.1 H), 3.14 (bs, 0.9 H), 1.44 (d, *J* = 6.2 Hz, 0.4 H), 1.37 (d, *J* = 6.2 Hz, 2.6 H).

^{13}C {^{1}H}-NMR (151 MHz, $CDCl_3$)**:** δ [ppm] = 166.0, 165.8, 165.7, 133.6, 133.5, 133.3, 130.1 (2C), 129.88 (2C), 129.85 (2C), 129.5, 129.4, 129.3, 128.7 (2C), 128.6 (2C), 128.4 (2C), 92.4, 72.0, 71.3, 69.8, 66.9, 17.9.
*: Signal overlaps with solvent residual peak.
Only ^{13}C signals of the main anomer are given.
***R*f** (pentane/EtOAc = 8/1) = 0.36.
NMR data matched literature values.[13]

(2*S*,3*S*,4*R*,5*R*,6*S*)-2-methyl-6-(2,2,2-trichloro-1-iminoethoxy)tetrahydro-2*H*-pyran-3,4,5-triyl tribenzoate (**31**)

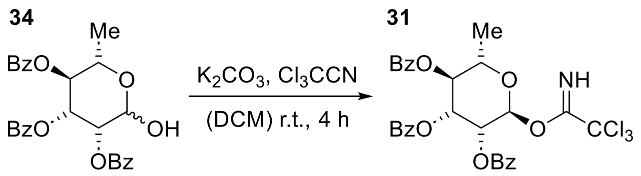

In a flame-dried 50 mL Schlenk flask, 272 mg (3*R*,4*R*,5*S*,6*S*)-2-hydroxy-6-methyltetrahydro-2*H*-pyran-3,4,5-triyl tribenzoate (0.57 mmol, 1.0 eq.) was dissolved in DCM (8 mL). 473 mg K_2CO_3 (3.42 mmol, 6.0 eq.) were added, and after stirring for 5 minutes, 343 µL of Cl_3CCN (494 mg, 3.42 mmol, 6.0 eq.) were added dropwise. The resulting mixture was stirred at room temperature for 2 hours, when another 114 µL (164 mg, 1.14 mmol, 2.0 eq.) Cl_3CCN were added. After stirring for another 2 hours, the mixture was filtered through a pad of Na_2SO_4 and the solvent removed under reduced pressure, furnishing 350 mg of a white foam (0.55 mmol, 96%), which was used without further purification (anomeric purity determined by ^{1}H-NMR: α/β = ~96/4).
A small amount of the residue was chromatographed on neutral Al_2O_3 (pentane/EtOAc = 6/1) to yield an analytically pure sample.
^{1}H-NMR (600 MHz, $CDCl_3$)**:** δ [ppm] = 8.82 (s, 1 H), 8.12–8.09 (m, 2 H), 8.00–7.96 (m, 2 H), 7.84–7.80 (m, 2 H), 7.64–7.60 (m, 1 H), 7.54–7.48 (m, 3 H), 7.45–7.41 (m, 1 H), 7.40–7.37 (m, 2 H), 7.28–7.24 (m, 2 H),* 6.49 (d, *J* = 1.7 Hz, 1 H), 5.91–5.87 (m, 3 H), 5.80–5.75 (m, 1 H), 4.40 (dq, *J* = 9.8, 6.2 Hz, 1 H), 1.42 (d, *J* = 6.2 Hz, 3 H).
^{13}C {^{1}H}-NMR (151 MHz, $CDCl_3$)**:** δ [ppm] = 165.8, 165.6, 165.4, 160.2, 133.8, 133.6, 133.4, 130.1 (2C), 129.9 (2C), 129.8 (2C), 129.19, 129.15, 129.1, 128.8 (2C), 128.6 (2C), 128.5 (2C), 94.9, 90.9, 71.2, 69.81, 69.79, 69.3, 17.9.
*: Signal overlaps with solvent residual peak.
Determination of α/β by 2D NOESY-NMR:

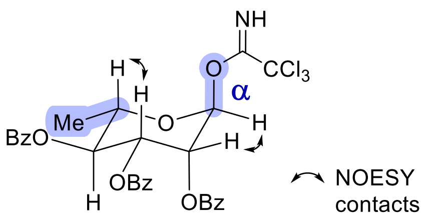

HR-MS (ESI+)**:** *m/z* calcd. for $C_{29}H_{24}Cl_3NO_8$ $[M+Na]^+$: 642.0460, found: 642.0465.
Predominant MS signal due to in-source elimination of trichloroacetamide during ESI (*m/z* calcd. for $C_{27}H_{22}O_7$ [M+H]+: 459.1439, found: 459.1446).
NMR data matched literature values.[13]

3.1.14.2 Glycosylation, deprotection and hydroxylation

(2*R*,3*R*,4*R*,5*S*,6*S*)-2-(((*R*)-1-((8-iodo-5-oxo-1,2,3,5-tetrahydroindolizin-6-yl)amino)-1-oxotetradecan-3-yl)oxy)-6-methyltetrahydro-2*H*-pyran-3,4,5-triyl tribenzoate (**35**)

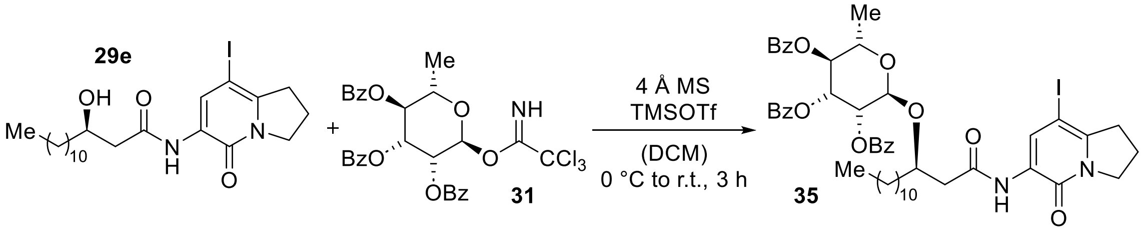

In a flame-dried 10 mL Schlenk tube, 15.0 mg (*R*)-3-hydroxy-*N*-(8-iodo-5-oxo-1,2,3,5-tetrahydro-indolizin-6-yl)tetradecanamide (29.8 µmol, 1.0 eq.) was dissolved in 2 mL DCM. Activated molecular sieve (4 Å) was added, and a solution of 19.7 mg (2*S*,3*S*,4*R*,5*R*,6*R*)-2-methyl-6-(2,2,2-trichloro-1-iminoethoxy)tetrahydro-2H-pyran-3,4,5-triyl tribenzoate (31.7 µmol, 1.06 eq.) in DCM (0.5 mL) was added to the mixture at 0 °C. After stirring for 10 minutes, a solution of 2 µL TMSOTf (2.5 mg, 11.1 µmol, 0.37 eq.) in DCM (200 µL) was added slowly. The mixture was stirred at 0 °C for 15 minutes, and another 3 hours at room temperature. The mixture was filtered and poured onto 10 mL H_2O. The aqueous phase was extracted with DCM (2 × 10 mL), combined organic phases were dried over Na_2SO_4, filtered, and the solvents removed *in vacuo*. The residue was purified by column chromatography (pentane/EtOAc = 1/1), yielding 15.5 mg of the desired product as a colorless oil (16.1 µmol, 54%; anomeric purity determined by ^{1}H-NMR: α/β = ~98/2).

^{1}H-NMR (600 MHz, $CDCl_3$)**:** δ [ppm] = 8.67 (s, 1 H), 8.51 (bs, 1 H), 8.12–8.07 (m, 2 H), 7.96–7.91 (m, 2 H), 7.82–7.77 (m, 2 H), 7.62–7.58 (m, 1 H), 7.52–7.46 (m, 3 H), 7.43–7.39 (m, 1 H), 7.39–7.35 (m, 2 H), 7.26–7.22 (m, 2 H), 5.76 (dd, *J* = 10.1, 3.4 Hz, 1 H), 5.61 (dd, *J* = 10.1, 9.8 Hz, 1 H), 5.61 (dd, *J* = 3.4, 1.8 Hz, 1 H), 5.16 (d, *J* = 1.8 Hz, 1 H), 4.32–4.24 (m, 3 H), 4.21 (dq, *J* = 9.8, 6.2 Hz, 1 H), 3.08–2.96 (m, 2 H), 2.75 (dd, *J* = 14.7, 7.5 Hz, 1 H), 2.70 (dd, *J* = 14.7, 4.7 Hz, 1 H), 2.34–2.11 (m, 2 H), 1.77–1.70 (m, 1 H), 1.67–1.60 (m, 1 H), 1.46–1.38 (m, 2 H), 1.36–1.29 (m, 16 H), 1.30 (d, *J* = 6.3 Hz, 3 H), 0.86 (t, *J* = 7.0 Hz, 3 H).

^{13}C {^{1}H}-NMR (151 MHz, $CDCl_3$)**:** δ [ppm] = 169.6, 166.0, 165.8, 165.5, 156.0, 144.6, 133.5, 133.3, 133.1, 130.3, 130.1 (2C), 129.9 (2C), 129.8 (2C), 129.62, 129.60, 129.5, 128.7 (2C), 128.5 (2C), 128.4 (2C), 127.8, 96.4, 75.7, 72.1, 71.4, 70.2, 67.3, 62.7, 51.2, 43.4, 36.0, 33.6, 32.1, 29.79, 29.77 (2C), 29.73, 29.66, 29.5, 25.2, 22.8, 21.0, 17.8, 14.3.

Determination of α/β by 2D NOESY-NMR:

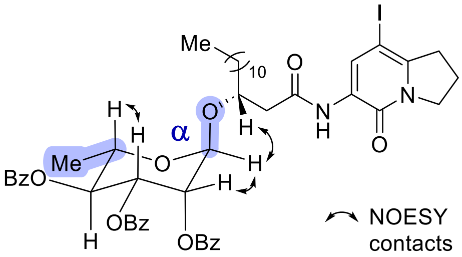

HR-MS (ESI+)**:** *m/z* calcd. for $C_{49}H_{57}IN_2O_{10}$ $[M+H]^+$: 961.3131, found: 961.3121.

***R*f** (pentane/EtOAc = 1/1) = 0.56.

Specific rotation: $[\alpha]_D$ = +29.0 $^{\circ}\cdot{}^{mL}/_{dm\cdot g}$ (ρ = 1.03; $CHCl_3$).

(*R*)-*N*-(8-iodo-5-oxo-1,2,3,5-tetrahydroindolizin-6-yl)-3-(((2*R*,3*R*,4*R*,5*R*,6*S*)-3,4,5-trihydroxy-6-methyltetrahydro-2*H*-pyran-2-yl)oxy)tetradecanamide (**36**)

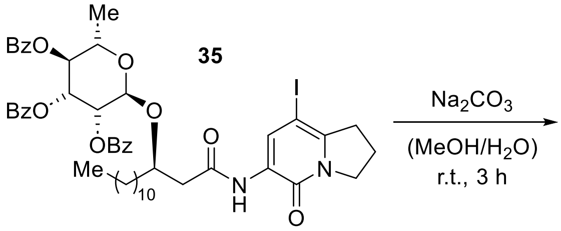

In a 5 mL glass vial, 14.6 mg (2*R*,3*R*,4*R*,5*S*,6*S*)-2-(((*R*)-1-((8-iodo-5-oxo-1,2,3,5-tetrahydroindolizin-6-yl)amino)-1-oxotetradecan-3-yl)oxy)-6-methyltetrahydro-2*H*-pyran-3,4,5-triyl tribenzoate (15.2 µmol, 1.0 eq.) were dissolved in MeOH (1.5 mL), and 0.15 mL of saturated Na_2CO_3 solution (corresponds to ~33 mg, 307 µmol, 20 eq.) was added. The mixture was stirred at room temperature for 3 hours, before being poured onto 5 mL of saturated NH_4Cl solution. After extraction with $CHCl_3$/EtOH (2/1, 3 × 10 mL), the combined phases were dried over Na_2SO_4, filtered, and the solvents removed under reduced pressure. The residue was purified by preparative HPLC to yield 6.8 mg of a white solid (10.5 µmol, 69%).

^{1}H-NMR (600 MHz, DMSO-*d*$_6$)**:** δ [ppm] = 9.25 (s, 1 H), 8.44 (s, 1 H), 4.67 (d, *J* = 4.2 Hz, 1 H), 4.62 (d, *J* = 1.6 Hz, 1 H), 4.59 (d, *J* = 5.1 Hz, 1 H), 4.44 (d, *J* = 6.0 Hz, 1 H), 4.15 (dd, *J* = 8.2, 6.7 Hz, 2 H), 4.00–3.94 (m, 1 H), 3.56 (td, *J* = 3.7, 1.6 Hz, 1 H), 3.39–3.34 (m, 2 H)*, 3.13 (td, *J* = 9.4, 4.9 Hz, 1 H), 2.99 (t, *J* = 7.7 Hz, 2 H), 2.67 (dd, *J* = 14.5, 7.5 Hz, 1 H), 2.56 (dd, *J* = 14.5, 5.1 Hz, 1 H), 2.12 (qnt, *J* = 7.7 Hz, 2 H), 1.49–1.40 (m, 2 H), 1.30–1.26 (m, 2 H), 1.26–1.21 (m, 16 H), 1.02 (d, *J* = 6.2 Hz, 3 H), 0.85 (t, *J* = 7.0 Hz, 3 H).

*Signals overlap with water peak.

^{13}C {^{1}H}-NMR (151 MHz, DMSO-*d*$_6$)**:** δ [ppm] = 170.2, 155.2, 145.4, 130.2, 127.6, 98.8, 73.7, 71.9, 70.9, 70.6, 68.7, 61.8, 50.9, 42.1, 35.4, 32.9, 31.3, 29.1 (2C), 29.03, 29.00 (2C), 28.8, 24.3, 22.1, 20.4, 17.8, 14.0.

HR-MS (ESI+)**:** *m/z* calcd. for $C_{28}H_{45}IN_2O_7$ $[M+Na]^+$: 671.2164, found: 671.2164.

Specific rotation: $[\alpha]_D = -33.1\ ^\circ\cdot mL/_{dm\cdot g}$ (ρ = 0.45; MeOH).

(*R*)-*N*-(8-hydroxy-5-oxo-1,2,3,5-tetrahydroindolizin-6-yl)-3-(((2*R*,3*R*,4*R*,5*R*,6*S*)-3,4,5-trihydroxy-6-methyltetrahydro-2*H*-pyran-2-yl)oxy)tetradecanamide (**3f**)

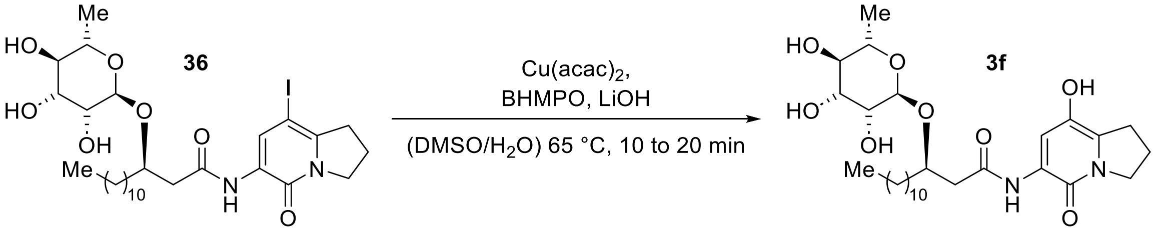

In a typical reaction, 2.2 mg (*R*)-*N*-(8-iodo-5-oxo-1,2,3,5-tetrahydroindolizin-6-yl)-3-(((2*R*,3*R*,4*R*,5*R*,6*S*)-3,4,5-trihydroxy-6-methyltetrahydro-2*H*-pyran-2-yl)oxy)tetradecanamide (3.39 µmol, 1.0 eq.), 2.1 mg $Cu(acac)_2$ (8.02 µmol, 2.4 eq.), 2.6 mg BHMPO (7.92 µmol, 2.3 eq.) and 3.5 mg LiOH (146 µmol, 43 eq.) were placed in a 10 mL Schlenk tube. After evacuating and flushing with argon three times, DMSO (0.3 mL) and degassed H_2O (75 µL) were added and the mixture immediately heated to 65 °C under vigorous stirring, forming a dark brown color. After 10 to 20 minutes (progress monitored by HPLC), the reaction tube was placed on liquid nitrogen to rapidly cool it. After thawing, it was poured onto MeOH (0.5 mL) with TFA (10 µL) and immediately purified by preparative HPLC, yielding a beige solid. Combining the products of three assays (7.7 mg starting material in total; 11.9 µmol) resulted in 1.5 mg of a beige solid (2.78 µmol, 23%).

^{1}H-NMR (600 MHz, DMSO-d_6)**:** δ [ppm] = 9.05 (s, 1 H), 8.62 (s, 1 H), 8.17 (s, 1 H), 4.78–4.33 (bs, 3 H), 4.64 (d, *J* = 1.6 Hz, 1 H), 4.01–3.94 (m, 3 H), 3.57 (dd, *J* = 3.4, 1.7 Hz, 1 H), 3.40 (dq, *J* = 9.4, 6.2 Hz, 1 H)*, 3.39 (dd, *J* = 9.4, 3.4 Hz, 1 H)*, 3.12 (dd, *J* = 9.4, 9.4 Hz, 1 H), 2.93 (t, *J* = 7.6 Hz, 2 H), 2.65 (dd, *J* = 14.6, 7.2 Hz, 1 H), 2.58 (dd, 14.5, 5.1 Hz, 1 H), 2.08 (qnt, *J* = 7.2 Hz, 2 H), 1.49–1.42 (m, 2 H), 1.31–1.20 (m, 18 H), 1.02 (d, *J* = 6.2 Hz, 3 H), 0.85 (t, *J* = 7.0 Hz, 3 H).
*Signals overlap with water peak.
^{13}C {^{1}H}-NMR (126 MHz, DMSO-d_6)**:** δ [ppm] = 169.6, 152.8, 133.5, 126.2, 126.1, 118.0, 98.8, 73.5, 71.9, 70.8, 70.6, 68.7, 49.1, 42.3, 32.8, 31.3, 29.11, 29.06 (2C), 29.01 (3C), 28.7, 24.3, 22.1, 21.4, 17.8, 14.0.
NMR spectra showed fast decomposition to various compounds (see below); hence for overlapping ^{1}H-signals, theoretical integrals are given. Assignments were determined by 2D NMR.
HR-MS (ESI+)**:** *m/z* calcd. for $C_{28}H_{46}N_2O_8$ [M+Na]$^+$: 561.3147, found: 561.3149.
Specific rotation: $[\alpha]_D = -24.0\ ^{\circ}\cdot{}^{mL}/_{dm\cdot g}$ (ρ = 0.13; MeOH).

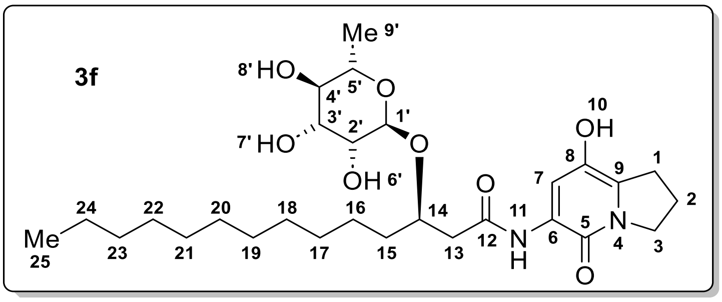

Table S5. NMR analysis of **3f** in DMSO-d_6. Assignment by HSQC and HMBC experiments.

#	^{1}H-NMR (δ, *J*) @ 600 MHz	^{13}C-NMR (δ) @ 126 MHz (BBO(F) CryoProbe)	COSY @ 500 MHz	^{1}H–^{13}C HMBC @ 600/151 MHz
**1**	2.93 (t, 7.6 Hz)	27.70	2	2,3,7 (weak),8,9
**2**	2.08 (qnt, 7.5 Hz)	21.42	1,3	1,3,9
**3**	3.99 (bt, 7.5 Hz)	49.11	2	1,2,9
**5**	–	152.83	–	–
**6**	–	126.21	–	–
**7**	8.17 (s)	117.96	–	5,8,9
**8**	–	133.46	–	–
**9**	–	126.10	–	–
**10**	8.62 (s)	–	–	–
**11**	9.05 (s)	–	–	5,7,12
**12**	–	169.58	–	–
**13**	2.65 (dd, 14.5, 7.2 Hz), 2.58 (dd, 14.5, 5.1 Hz)	42.30	14	12,14
**14**	4.00–3.94 (m)	73.47	13,15	13,1’
**15**	1.49–1.42 (m)	32.76	14	–
**16**	1.31–1.20 (m)	24.29	overl.	overlap
**17** **18** **19** **20** **21** **22**	1.31–1.20 (m)	29.11, 29.06, 29.01, 28.72	overl.	overlap
**23**	1.31–1.20 (m)	31.29	overl.	overlap
**24**	1.31–1.20 (m)	22.09	overl.	overlap
**25**	0.85 (t, 7.0 Hz)	13.96	overl.	23,24
**1’**	4.64 (d, 1.6 Hz)	98.75	overl.	14,3’,5’
**2’**	3.57 (dd, 3.4, 1.7 Hz)	70.84	6’	3’,4’
**3’**	3.39 (dd, 9.4, 3.4 Hz)	70.57	overl.	4’
**4’**	3.12 (dd, 9.4, 9.4 Hz)	71.92	8’	3’,5’,9’
**5’**	3.40 (dq, 9.4, 6.2 Hz)	68.72	9’	4’
**6’**	4.68 (d, 4.2 Hz)	–	2’	(* 1’,2’,3’)
**7’**	4.45 (d, 5.9 Hz)	–	3’	(* 2’,3’,4’)
**8’**	4.57 (d, 5.1 Hz)	–	4’	(* 3’,4’,5’)
**9’**	1.02 (d, 6.2 Hz)	17.82	5’	4’,5’

* Not observed due to broad OH-signals in HMBC measurement; correlations taken from HMBC of precursor iodide **36** (Fig. S262).

Commonly observed side products of hydroxylation reactions

By **hydro-de-iodination**:

(*R*)-*N*-(5-oxo-1,2,3,5-tetrahydroindolizin-6-yl)-3-(((2*R*,3*R*,4*R*,5*R*,6*S*)-3,4,5-trihydroxy-6-methyltetrahydro-2*H*-pyran-2-yl)oxy)tetradecanamide (**37**)

37

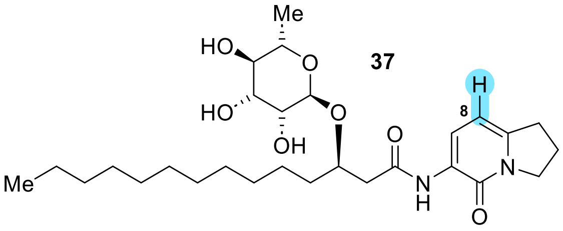

^{1}H-NMR (600 MHz, DMSO-d_6)**:** δ [ppm] = 9.08 (s, 1 H), 8.17 (d, *J* = 7.5 Hz, 1 H), 6.17 (dt, *J* = 7.5, 1.3 Hz, 1 H), 4.69 (d, *J* = 4.2 Hz, 1 H), 4.63 (d, *J* = 1.6 Hz, 1 H), 4.59 (d, *J* = 5.3 Hz, 1 H), 4.46 (d, *J* = 6.0 Hz, 1 H), 4.02 (bt, *J* = 7.3 Hz, 2 H), 3.99–3.95 (m, 1 H), 3.58–3.55 (m, 1 H), 3.43–3.36 (m, 2 H)*, 3.12 (td, *J* = 9.4, 5.1 Hz, 1 H), 3.02 (bt, *J* = 7.7 Hz, 2 H), 2.64 (dd, *J* = 14.2, 7.0 Hz, 1 H), 2.56 (dd, *J* = 14.2, 5.3 Hz, 1 H), 2.10 (qnt, *J* = 7.6 Hz, 2 H), 1.49–1.41 (m, 2 H), 1.32–1.18 (m, 18 H), 1.01 (d, *J* = 6.2 Hz, 3 H), 0.85 (t, *J* = 7.0 Hz, 3 H).

*Signals overlap with water peak.

HR-MS (ESI+)**:** *m/z* calcd. for $C_{28}H_{46}N_2O_7$ $[M+Na]^+$: 545.3198, found: 545.3188.

Isolation of **37**/**37-D** after hydroxylation reaction in DMSO-d_6/D_2O:

HR-MS (ESI+)**:** *m/z* calcd. for $C_{28}H_{46}N_2O_7$ $[M+Na]^+$: 545.3198, found: 545.3201.

HR-MS (ESI+)**:** *m/z* calcd. for $C_{28}H_{45}DN_2O_7$ $[M+Na]^+$: 546.3260, found: 546.3259.

Ratio of 8-protonated **37** to 8-deuterated **37-D**, as determined by mass counts: ~ 43/57

By **hydro-de-iodination** and **rhamnose elimination**:

(*E*)-*N*-(5-oxo-1,2,3,5-tetrahydroindolizin-6-yl)tetradec-2-enamide (**38**)

38

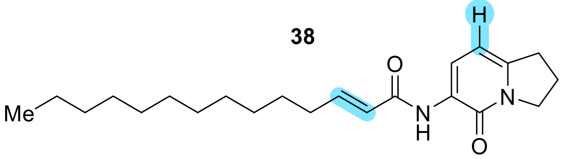

^{1}H-NMR (600 MHz, DMSO-d_6)**:** δ [ppm] = 9.16 (s, 1 H), 8.27 (d, *J* = 7.4 Hz, 1 H), 6.73 (dt, *J* = 15.2, 6.8 Hz, 1 H), 6.46 (dt, *J* = 15.2, 1.4 Hz, 1 H), 6.19 (dt, *J* = 7.6, 1.3 Hz, 1 H), 4.05–4.01 (m, 2 H), 3.05–3.01 (m, 2 H), 2.18–2.13 (m, 2 H), 2.11 (bqnt, *J* = 7.3 Hz, 2 H), 1.44–1.38 (m, 2 H), 1.30–1.20 (m, 18 H), 0.84 (t, *J* = 7.0 Hz, 3 H).

HR-MS (ESI+)**:** *m/z* calcd. for $C_{22}H_{34}N_2O_2$ $[M+Na]^+$: 381.2513, found: 381.2512.

Isolated oxidized degradation products

(3*R*)-*N*-(8a-hydroxy-5,8-dioxo-1,2,3,5,8,8a-hexahydroindolizin-6-yl)-3-(((2*R*,3*R*,4*R*,5*R*,6*S*)-3,4,5-trihydroxy-6-methyltetrahydro-2*H*-pyran-2-yl)oxy)tetradecanamide (**39**, 2 stereoisomers)

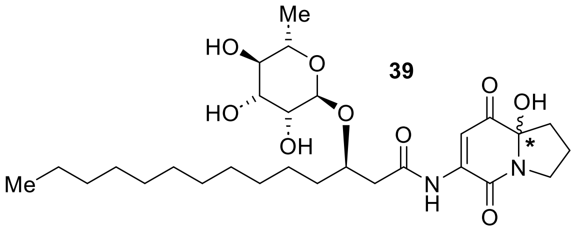

Isomer 1:

^{1}H-NMR (600 MHz, DMSO-*d*$_6$)**:** δ [ppm] = 9.77 (s, 1 H), 7.13 (s, 1 H), 6.75 (bs, 1 H), 4.68 (d, *J* = 4.2 Hz, 1 H), 4.65 (d, *J* = 5.0 Hz, 1 H), 4.63 (d, *J* = 1.6 Hz, 1 H), 4.42 (d, *J* = 6.0 Hz, 1 H), 4.00–3.95 (m, 1 H), 3.61 (dd, *J* = 9.4, 5.3 Hz, 2 H), 3.56–3.54 (m, 1 H), 3.34–3.29 (m, 2 H)*, 3.13 (td, *J* = 9.4, 4.9 Hz, 1 H), 2.88 (dd, *J* = 14.9, 8.0 Hz, 1 H), 2.61 (dd, *J* = 14.9, 4.5 Hz, 1 H), 2.08–1.99 (m, 2 H), 1.92–1.84 (m, 2 H), 1.50–1.42 (m, 2 H), 1.29–1.21 (m, 18 H)**, 1.03 (d, *J* = 6.1 Hz, 3 H), 0.85 (t, *J* = 6.8 Hz, 3 H)**.

^{13}C {^{1}H} from HSQC/HMBC (DMSO-*d*$_6$)**:** δ [ppm] = 172.0$_{HMBC}$, 156.8$_{HMBC}$, 141.5$_{HMBC}$, 109.7 (δ^{1H} = 7.13), 98.5 (δ^{1H} = 4.63), 87.3$_{HMBC}$, 73.0 (δ^{1H} = 3.98), 71.8 (δ^{1H} = 3.13), 70.8 (δ^{1H} = 3.55), 70.6 (δ^{1H} = 3.33), 68.7 (δ^{1H} = 3.31), 45.5 (δ^{1H} = 3.60), 42.3 (δ^{1H} = 2.88, 2.61), 34.4 (δ^{1H} = 2.06, 1.87), 32.7 (δ^{1H} = 1.46), 31.4 (δ^{1H} = 1.24), 29.0 (δ^{1H} = 1.24), 24.3 (δ^{1H} = 1.26), 22.1 (δ^{1H} = 1.26), 19.2 (δ^{1H} = 2.02, 1.90), 17.8 (δ^{1H} = 1.03), 13.9 (δ^{1H} = 0.85).

HR-MS (ESI+)**:** *m/z* calcd. for $C_{28}H_{46}N_2O_9$ $[M+H]^+$: 555.3277, found: 555.3283.

Predominant MS signal due to in-source elimination of water during ESI (*m/z* calcd. for $[M+H–H_2O]^+$: 537.3171, found: 537.3179).

Isomer 2:

^{1}H-NMR (600 MHz, DMSO-*d*$_6$)**:** δ [ppm] = 9.83 (s, 1 H), 7.13 (s, 1 H), 6.74 (bs, 1 H), 4.71 (d, *J* = 5.1 Hz, 1 H), 4.70 (d, *J* = 4.2 Hz, 1 H), 4.64 (d, *J* = 1.6 Hz, 1 H), 4.47 (d, *J* = 6.0 Hz, 1 H), 4.01–3.95 (m, 1 H), 3.64–3.57 (m, 2 H), 3.57–3.55 (m, 1 H), 3.38–3.34 (m, 2 H)*, 3.15 (td, *J* = 9.3, 5.1 Hz, 1 H), 2.77 (dd, *J* = 15.3, 5.4 Hz, 1 H), 2.73 (dd, *J* = 15.3, 7.3 Hz, 1 H), 2.09–1.99 (m, 2 H), 1.94–1.83 (m, 2 H), 1.49–1.40 (m, 2 H)**, 1.30–1.21 (m, 18 H)**, 1.04 (d, *J* = 6.2 Hz, 3 H), 0.85 (t, *J* = 6.9 Hz, 3 H)**.

^{13}C {^{1}H} from HSQC/HMBC (DMSO-*d*$_6$)**:** δ [ppm] = 171.8$_{HMBC}$, 156.7$_{HMBC}$, 141.6$_{HMBC}$, 109.6 (δ^{1H} = 7.13), 98.7 (δ^{1H} = 4.64), 87.3$_{HMBC}$, 72.9 (δ^{1H} = 3.98), 71.9 (δ^{1H} = 3.15), 70.8 (δ^{1H} = 3.56), 70.5 (δ^{1H} = 3.36), 68.8 (δ^{1H} = 3.37), 45.5 (δ^{1H} = 2.75), 42.5 (δ^{1H} = 4.64), 34.4 (δ^{1H} = 2.07, 1.85), 32.7 (δ^{1H} = 1.45), 31.4 (δ^{1H} = 1.24), 29.1 (δ^{1H} = 1.24), 24.2 (δ^{1H} = 1.26), 22.1 (δ^{1H} = 1.26), 19.1 (δ^{1H} = 2.02, 1.92), 17.8 (δ^{1H} = 1.04), 14.0 (δ^{1H} = 0.85).

HR-MS (ESI+)**:** *m/z* calcd. for $C_{28}H_{46}N_2O_9$ $[M+Na]^+$: 577.3096, found: 577.3037.

Predominant MS signal due to in-source elimination of water during ESI (*m/z* calcd. for $[M+H–H_2O]^+$: 537.3171, found: 537.3117).

*Overlaps with water signal.

**For overlapping signals (from impurities/isomers), theoretical integrals are given.

Double oxidation product (**SI-4**)

HR-MS (ESI+)**:** *m/z* calcd. for $C_{28}H_{46}N_2O_{10}$ $[M+H]^+$: 571.3226, found: 571.3218.

Predominant MS signal due to in-source de-rhamnosylation during ESI (*m/z* calcd. for $C_{22}H_{36}N_2O_6$ $[M+H]^+$: 425.2647, found: 425.2642).

3.2 Synthesis of brabantamide/pyrrolizixenamide standards

3.2.1 Brabantamide synthesis[14]

3.2.1.1 Bicyclic enol core synthesis

tert-butyl (*R*)-2-(2,2-dimethyl-4,6-dioxo-1,3-dioxane-5-carbonyl)pyrrolidine-1-carboxylate (**41**)

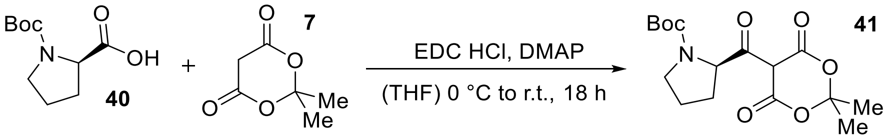

In a flame-dried 100 mL Schlenk flask, 1.9 g *N*-Boc-D-proline (8.8 mmol, 1.0 eq.) was dissolved in anhydrous THF (23 mL), the solution was cooled to 0 °C and 1.86 g 1-ethyl-3-(3-dimethylaminopropyl)carbodiimide hydrochloride (EDC HCl) (9.7 mmol, 1.1 eq.) was added. After stirring for 2 minutes, 1.61 g DMAP (13.2 mmol, 1.5 eq.) and 1.53 g Meldrum's acid (10.6 mmol, 1.2 eq.) were added. The reaction mixture was stirred for 18 hours at room temperature. Next, the suspension was filtered, and the filtrate was concentrated under reduced pressure. The residue was dissolved in DCM (50 mL), washed with 1 M HCl (2 × 15 mL) and water (30 mL). The organic layer was dried over Na_2SO_4, filtered and the solvent was removed under reduced pressure. The obtained residue (3.05 g of a yellow oil) was directly used in the next step.

tert-butyl (*R*)-2-(3-oxo-3-(2-(trimethylsilyl)ethoxy)propanoyl)pyrrolidine-1-carboxylate (**43**)

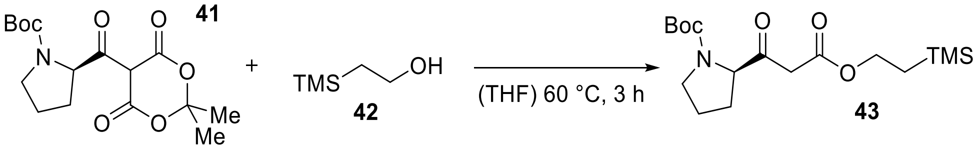

In a 100 mL round-bottomed flask, the Meldrum's acid intermediate was dissolved in dry THF (24 mL), 1.26 g 2-(trimethylsilyl)ethanol (1.52 mL, 10.6 mmol, 1.2 eq.) was added, and the reaction mixture was stirred at 60 °C for 3.5 hours. The reaction mixture was concentrated under reduced pressure and the residue was purified by column chromatography (pentane/EtOAc = 7/1), yielding 2.02 g of a colourless oil (5.65 mmol, 64% over two steps).

^{1}H-NMR (300 MHz, $CDCl_3$)**:** δ [ppm] = 12.09 (s, 0.1 H), 5.00 (s, 0.1 H), 4.39 (dd, *J* = 8.1, 5.1 Hz, 0.4 H), 4.31–4.16 (m, 2.6 H), 3.63–3.38 (m, 3.8 H), 2.28–1.80 (m, 4 H), 1.45 (s, 3.6 H), 1.43 (s, 4.8 H), 1.40 (s, 0.6 H), 1.05–0.96 (m, 2 H), 0.04 (s, 9 H).

^{13}C {^{1}H}-NMR (75.5 MHz, $CDCl_3$)**:** δ [ppm] = 203.3, 203.0, 167.6, 167.3, 155.0, 153.9, 80.9, 80.3, 65.7, 65.2, 63.9, 63.8, 47.1, 46.9, 46.8, 45.6, 29.9, 28.7, 28.6, 28.4, 24.6, 23.9, 17.5, –1.4.

R_f (pentane/EtOAc = 7/1) = 0.19.

NMR spectra showed a mixture of rotamers (~55:45) and tautomers (~9:1). The data matched those reported in literature.[14]

2-(trimethylsilyl)ethyl (*R*,*E*)-2-(3-oxotetrahydro-1*H*,3*H*-pyrrolo[1,2-*c*]oxazol-1-ylidene)acetate (**44**)

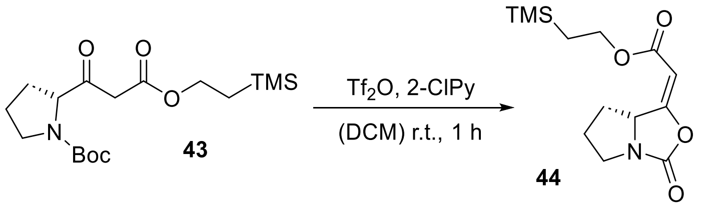

In a flame-dried 100 mL Schlenk flask, to a stirred solution of 1.94 g *tert*-butyl (*R*)-2-(3-oxo-3-(2-(trimethylsilyl)ethoxy)propanoyl)pyrrolidine-1-carboxylate (5.41 mmol, 1.0 eq.) in DCM (35 mL), 1.57 mL 2-chloropyridine (1.91 g, 11.2 mmol, 3.1 eq.), followed by 1.73 g trifluoromethanesulfonic

anhydride (1.04 mL, 6.15 mmol, 1.1 eq.) were added, and the mixture stirred at room temperature for 1 hour. The mixture was filtered over a pad of silica (DCM/EtOAc = 95/5) to remove the dark brown color, and the filtrate concentrated under reduced pressure. Residual 2-chloropyridine was removed by applying high vacuum overnight. The residue was purified by column chromatography (100% DCM), yielding 1.01 g of a colorless oil (3.78 mmol, 70%).

^{1}H-NMR (600 MHz, $CDCl_3$)**:** δ [ppm] = 5.63 (d, *J* = 2.0 Hz, 1 H), 4.91 (ddd, *J* = 9.1, 6.6, 2.0 Hz, 1 H), 4.25–4.16 (m, 2 H), 3.68 (dt, *J* = 11.4, 7.9 Hz, 1 H), 3.29 (ddd, *J* = 11.3, 8.9, 4.5 Hz, 1 H), 2.67–2.60 (m, 1 H), 2.16–2.02 (m, 2 H), 1.60 (dq, *J* = 12.7, 9.6 Hz, 1 H), 1.04–0.98 (m, 2 H), 0.05 (s, 9 H).

^{13}C {^{1}H}-NMR (151 MHz, $CDCl_3$)**:** δ [ppm] = 166.5, 165.5, 157.0, 96.1, 64.4, 62.8, 46.1, 30.5, 26.4, 17.5, –1.4.

R_f (DCM) = 0.36.

NMR data matched those reported in literature.[14]

(*R*,*E*)-2-(3-oxotetrahydro-1*H*,3*H*-pyrrolo[1,2-*c*]oxazol-1-ylidene)acetic acid (**45**)

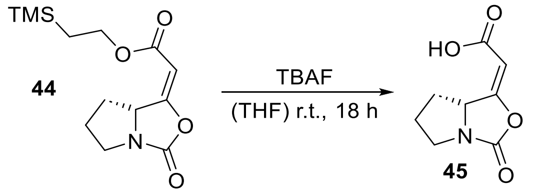

In a flame-dried 50 mL Schlenk flask, 0.98 g 2-(trimethylsilyl)ethyl (*R*,*E*)-2-(3-oxotetrahydro-1*H*,3*H*-pyrrolo[1,2-*c*]oxazol-1-ylidene)acetate (3.66 mmol, 1.0 eq.) was dissolved in THF (12 mL) and 7.3 mL of a solution of TBAF (1 M in THF, 7.3 mmol, 2.0 eq.) was added. After stirring at room temperature for 18 hours, the reaction mixture was diluted with water (10 mL), acidified with 1 M HCl to pH 3 and extracted with EtOAc (3 × 20 mL). The combined organic layers were dried over Na_2SO_4, filtered and the solvents removed under reduced pressure. The residue was purified by MPLC, yielding 352 mg of a white solid (1.92 mmol, 53%).

^{1}H-NMR (600 MHz, $CDCl_3$)**:** δ [ppm] = 10.28 (vbs, 1 H), 5.67 (bs, 1 H), 4.92 (bt, *J* = 8.1 Hz, 1 H), 3.69 (bq, *J* = 8.3 Hz, 1 H), 3.35–3.26 (m, 1 H), 2.67–2.56 (m, 1 H), 2.19–2.03 (m, 2 H), 1.67–1.57 (m, 1 H).

^{1}H-NMR spectrum showed generally broad signals.

^{13}C {^{1}H}-NMR (151 MHz, $CDCl_3$)**:** δ [ppm] = 171.9, 168.0, 156.5, 95.2, 64.5, 46.1, 30.4, 26.4.

NMR data matched those reported in literature.[14]

3.2.1.2 Amide coupling

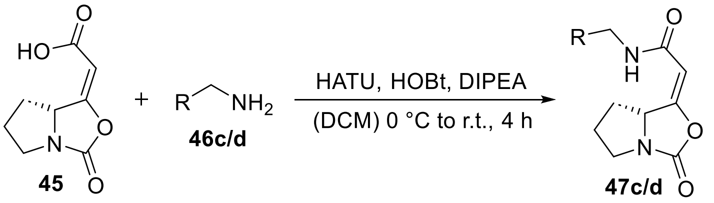

In a flame-dried 25 mL Schlenk flask, (*E*)-2-(3-oxotetrahydro-1*H*,3*H*-pyrrolo[1,2-*c*]oxazol-1-ylidene)acetic acid (1.0 eq.) was dissolved in DCM (5 mL) and cooled to 0 °C. HATU (1.5 eq.), HOBt (1.5 eq.) and DIPEA (2.4 eq.) were added, and the solution stirred for 10 minutes. Afterwards, the corresponding amine (1.5 eq.) was added, and the mixture was stirred at room temperature for 4 hours. The reaction was quenched by addition of 1 M HCl_{aq}, and the aqueous phase was extracted with EtOAc (3 × 30 mL). Combined organic phases were washed with brine (20 mL), dried over Na_2SO_4,

filtered and the solvents removed under reduced pressure. The residue was purified by column chromatography to give the desired amide.

(*R*,*E*)-*N*-decyl-2-(3-oxotetrahydro-1*H*,3*H*-pyrrolo[1,2-*c*]oxazol-1-ylidene)acetamide (**47c**)

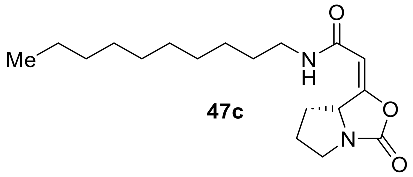

This compound was synthesized from (*E*)-2-(3-oxotetrahydro-1*H*,3*H*-pyrrolo[1,2-*c*]oxazol-1-ylidene)acetic acid and decylamine, yielding 109 mg of a colorless oil (338 µmol, 95%).

^{1}H-NMR (300 MHz, $CDCl_3$)**:** δ [ppm] = 6.01 (t, *J* = 5.8 Hz, 1 H), 5.58 (d, *J* = 2.1 Hz, 1 H), 4.95 (ddd, *J* = 9.0, 6.5, 2.0 Hz, 1 H), 3.61 (dt, *J* = 11.3, 7.9 Hz, 1 H), 3.31–3.12 (m, 3 H), 2.63 (dddd, *J* = 13.0, 6.7, 6.3, 3.5 Hz, 1 H), 2.01–1.93 (m, 2 H), 1.63–1.40 (m, 3 H), 1.31–1.17 (m, 14 H), 0.87–0.79 (m, 3 H).

^{13}C {^{1}H}-NMR (75.5 MHz, $CDCl_3$)**:** δ [ppm] = 165.1, 161.9, 157.3, 97.9, 64.2, 45.9, 39.6, 31.9, 30.9, 29.7, 29.59, 29.57, 29.3 (2C), 27.0, 26.2, 22.7, 14.1.

***R*$_f$** (pentane/EtOAc = 5/2) = 0.24.

HR-MS (ESI+)**:** *m/z* calcd. for $C_{18}H_{30}N_2O_3$ $[M+H]^+$: 323.2330, found: 323.2336.

Specific rotation: $[\alpha]_D$ = +162.9 $^{\circ}\cdot {}^{mL}/_{dm\cdot g}$ (ρ = 1.75; $CHCl_3$).

(*R*,*E*)-2-(3-oxotetrahydro-1*H*,3*H*-pyrrolo[1,2-*c*]oxazol-1-ylidene)-*N*-tetradecylacetamide (**47d**)

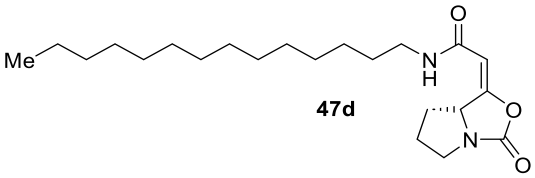

This compound was synthesized from (*E*)-2-(3-oxotetrahydro-1*H*,3*H*-pyrrolo[1,2-*c*]oxazol-1-ylidene)acetic acid and tetradecylamine, yielding 128 mg of a yellowish oil (338 µmol, 94%), which solidified upon standing.

^{1}H-NMR (600 MHz, $CDCl_3$)**:** δ [ppm] = 6.11 (t, *J* = 5.7 Hz, 1 H), 5.59 (d, *J* = 2.0 Hz, 1 H), 4.95 (ddd, *J* = 9.0, 6.5, 2.1 Hz, 1 H), 3.60 (dt, *J* = 11.3, 7.9 Hz, 1 H), 3.27–3.14 (m, 3 H), 2.62 (dddd, *J* = 13.2, 6.9, 6.5, 3.1 Hz, 1 H), 2.08–1.95 (m, 2 H), 1.59–1.51 (m, 1 H), 1.46 (qnt, *J* = 7.2 Hz, 2 H), 1.31–1.15 (m, 22 H), 0.83 (t, *J* = 7.1 Hz, 3 H).

^{13}C {^{1}H}-NMR (75.5 MHz, $CDCl_3$)**:** δ [ppm] = 165.1, 161.9, 157.3, 97.9, 64.2, 45.9, 39.6, 32.0, 30.9, 29.72, 29.71, 29.69 (2C), 29.65, 29.63, 29.61, 29.39, 29.35, 27.0, 26.2, 22.7, 14.2.

***R*$_f$** (pentane/EtOAc = 5/2) = 0.27.

HR-MS (ESI+)**:** *m/z* calcd. for $C_{22}H_{38}N_2O_3$ $[M+H]^+$: 379.2956, found: 379.2958.

Specific rotation: $[\alpha]_D$ = +134.3 $^{\circ}\cdot {}^{mL}/_{dm\cdot g}$ (ρ = 1.15; MeOH).

3.2.1.3 Amide oxidation[15]

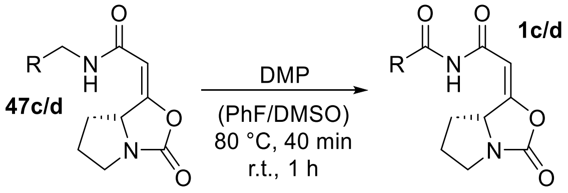

In a flame-dried 10 mL Schlenk tube, the corresponding amide (1.0 eq.) was dissolved in fluorobenzene (150 mM), and three drops of DMSO were added. After addition of DMP (2.0 eq.), the mixture was heated to 80 °C for 40 minutes and then stirred at room temperature for 1 hour. 2 mL of saturated $Na_2S_2O_3$ solution were added, and the mixture diluted with Et_2O (20 mL). The organic phase was separated and washed with a 50:50 mixture of saturated $Na_2S_2O_3$/$NaHCO_3$ solution (2 × 20 mL) and brine (20 mL), dried over Na_2SO_4, filtered and the volatiles were removed under reduced pressure. The residue was purified *via* preparative HPLC to yield the corresponding imide.

(*R*,*E*)-*N*-(2-(3-oxotetrahydro-1*H*,3*H*-pyrrolo[1,2-*c*]oxazol-1-ylidene)acetyl)decanamide (**1c**)

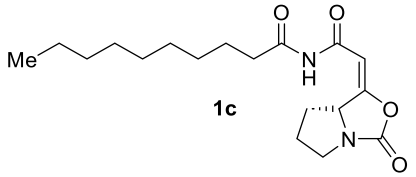

1c

This compound was synthesized from (*R*,*E*)-*N*-decyl-2-(3-oxotetrahydro-1*H*,3*H*-pyrrolo[1,2-*c*]oxazol-1-ylidene)acetamide, yielding 6.8 mg of a yellowish solid (20.2 µmol, 19%).

^{1}H-NMR (600 MHz, $CDCl_3$)**:** δ [ppm] = 8.26 (bs, 1 H), 6.46 (bs, 1 H), 4.93 (ddd, *J* = 9.1, 6.7, 2.0 Hz, 1 H), 3.69 (dt, *J* = 11.4, 7.9 Hz, 1 H), 3.31 (ddd, *J* = 11.4, 7.9 Hz, 4.5 Hz, 1 H), 2.70–2.64 (m, 1 H), 2.55 (t, *J* = 7.5 Hz, 2 H), 2.17–2.06 (m, 2 H), 1.66 (qnt, *J* = 7.5 Hz, 2 H), 1.59 (dt, *J* = 12.8, 9.3 Hz, 1 H), 1.37–1.32 (m, 2 H), 1.32–1.22 (m, 10 H), 0.87 (t, *J* = 7.0 Hz, 3 H).

^{13}C {^{1}H}-NMR (151 MHz, $CDCl_3$)**:** δ [ppm] = 173.9, 167.8, 165.2, 156.6, 97.1, 65.1, 46.2, 37.8, 32.0, 30.2, 29.6, 29.5, 29.4, 29.2, 26.5, 24.6, 22.8, 14.3.

HR-MS (ESI+)**:** *m/z* calcd. for $C_{18}H_{28}N_2O_4$ $[M+Na]^+$: 359.1942, found: 359.1947.

Specific rotation: $[\alpha]_D$ = +147.5 $^{\circ}\cdot{}^{mL}/_{dm\cdot g}$ (ρ = 0.40; MeOH).

(*R*,*E*)-*N*-(2-(3-oxotetrahydro-1*H*,3*H*-pyrrolo[1,2-*c*]oxazol-1-ylidene)acetyl)tetradecanamide (**1d**)

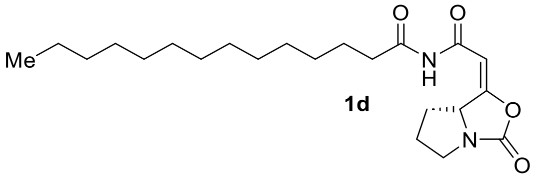

1d

This compound was synthesized from (*R*,*E*)-2-(3-oxotetrahydro-1*H*,3*H*-pyrrolo[1,2-*c*]oxazol-1-ylidene)-*N*-tetradecylacetamide, yielding 6.2 mg of a white solid (15.8 µmol, 20%).

^{1}H-NMR (600 MHz, $CDCl_3$)**:** δ [ppm] = 8.30 (bs, 1 H), 6.47 (bs, 1 H), 4.93 (ddd, *J* = 9.1, 6.6, 2.0 Hz, 1 H), 3.69 (dt, *J* = 11.4, 7.9 Hz, 1 H), 3.31 (ddd, *J* = 11.4, 8.8, 4.6 Hz, 1 H), 2.69–2.64 (m, 1 H), 2.55 (t, *J* = 7.5 Hz, 2 H), 2.17–2.06 (m, 2 H), 1.65 (qnt, *J* = 7.6 Hz, 2 H), 1.63–1.56 (m, 2 H), 1.37–1.32 (m, 2 H), 1.32–1.24 (m, 18 H), 0.88 (t, *J* = 7.0 Hz, 3 H).

^{13}C {^{1}H}-NMR (151 MHz, $CDCl_3$)**:** δ [ppm] = 173.9, 167.8, 165.2, 156.6, 97.1, 65.1, 46.2, 37.8, 32.1, 30.2, 29.82, 29.79 (2C), 29.7, 29.6, 29.50, 29.48, 29.2, 26.5, 24.6, 22.8, 14.3.

HR-MS (ESI+)**:** *m/z* calcd. for $C_{22}H_{36}N_2O_4$ $[M+Na]^+$: 415.2568, found: 415.2572.

Specific rotation: $[\alpha]_D$ = +147.1 $^{\circ}\cdot{}^{mL}/_{dm\cdot g}$ (ρ = 0.39; MeOH).

3.2.2 Pyrrolizixenamide synthesis[16]

3.2.2.1 PA core synthesis

tert-butyl (*S*)-3-amino-1-oxo-5,6,7,7a-tetrahydro-1*H*-pyrrolizine-2-carboxylate (**51**)

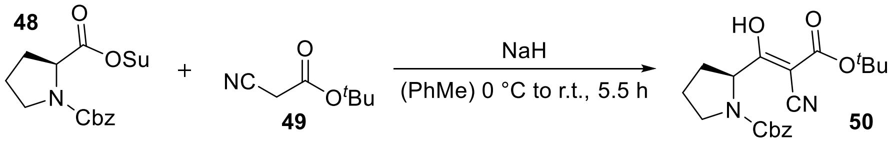

In a flame-dried 50 mL Schlenk flask, 0.29 g NaH (7.22 mmol, 2.5 eq.) were suspended in 18 mL of toluene, then 1.24 mL *tert*-butylcyanoacetate (1.22 g, 8.66 mmol, 3.0 eq.) were added slowly. After stirring for 2 hours at room temperature, a solution of 1.00 g Cbz-L-Pro-OSu (2.89 mmol, 1.0 eq.) in 5 mL of toluene was added. The mixture was stirred for another 3.5 hours, quenched by addition of water (30 mL) and extracted with pentane (50 mL). The aqueous phase was acidified by addition of 4 M HCl_{aq} to pH 3, then extracted with DCM (3 × 20 mL). Combined organic extracts were washed with brine (20 mL), dried over Na_2SO_4, filtered and the solvents removed under reduced pressure, yielding 1.41 g of an oily white solid, which was used directly in the next reaction.

tert-butyl (*S*)-3-amino-1-oxo-5,6,7,7a-tetrahydro-1*H*-pyrrolizine-2-carboxylate (**51**)

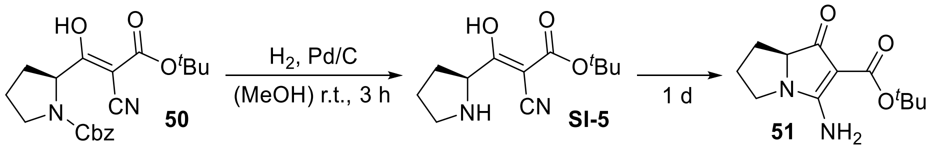

Crude *tert*-butyl (*S*)-3-amino-1-oxo-5,6,7,7a-tetrahydro-1*H*-pyrrolizine-2-carboxylate was dissolved in 50 mL of methanol and 0.39 g of activated palladium on charcoal (10%) were added. The mixture was vigorously stirred at room temperature under a H_2 atmosphere for 3 hours, before being filtered through a pad of celite. The filtrate was concentrated under reduced pressure. The residue was allowed to stand at room temperature for one day. In this time a yellowish solid formed, which was purified by column chromatography (EtOAc/MeOH = 95/5) to yield 0.24 g of a yellowish solid (1.01 mmol, 35% over 2 steps). A small amount was again purified *via* preparative HPLC for NMR measurements, yielding the TFA salt (from HPLC solvents).

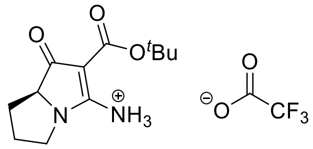

^{1}H-NMR (600 MHz, $CDCl_3$)**:** δ [ppm] = 8.15–7.65 (bs, 2.5 H), 6.87 (bs, 1 H), 4.21 (dd, *J* = 10.3, 6.8 Hz, 1 H), 3.49 (ddd, *J* = 10.9, 8.4, 2.7 Hz, 1 H), 3.35–3.29 (m, 1 H), 3.34–2.24 (m, 2 H), 2.24–2.14 (m, 1 H), 1.58–1.46 (m, 1 H), 1.54 (s, 9 H).**^{13}C {^{1}H}-NMR** (151 MHz, $CDCl_3$)**:** δ [ppm] = 192.0, 172.3, 164.5, 160.1 (q, *J* = 38.7 Hz), 115.6, (q, *J* = 288 Hz), 92.2, 82.1, 69.7, 46.2, 28.5 (3 C), 28.3, 27.0.

^{19}F {^{1}H}-NMR (282 MHz, $CDCl_3$)**:** δ [ppm] = –75.92 (bs).

***R*$_f$** (EtOAc/MeOH = 95/5) = 0.16.

HR-MS (ESI–): *m/z* calcd. for $[M–H]^-$: 237.1244, found: 237.1246.

Specific rotation: $[\alpha]_D$ = +7.4 $^{\circ}\cdot mL/_{dm \cdot g}$ (ρ = 0.41; MeOH).

3.2.2.2 Acylation/deprotection sequence

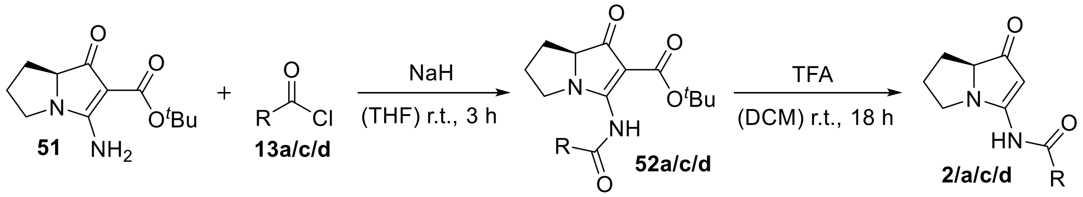

In a flame-dried 10 mL Schlenk flask, *tert*-butyl (*S*)-3-amino-1-oxo-5,6,7,7a-tetrahydro-1*H*-pyrrolizine-2-carboxylate (1.0 eq.) was dissolved in THF. NaH (3.0 eq.) was added and the mixture stirred at room temperature for 10 minutes. Next, the corresponding acid chloride (1.2 eq.) was added slowly, and the mixture stirred for 3 hours. The reaction was quenched by addition of water (10 mL) and the aqueous phase extracted with DCM (2 × 10 mL). Combined organic extracts were washed with brine (15 mL), dried over Na_2SO_4, filtered and the solvents removed under reduced pressure. The oily residue was dissolved in a mixture of DCM and TFA (9/1) and stirred at room temperature for 18 hours. The solution was poured on saturated $NaHCO_3$ solution (15 mL), and the aqueous phase extracted with DCM (2 × 10 mL). Combined organic extracts were washed with brine (15 mL), dried over Na_2SO_4, filtered and the solvents removed under reduced pressure. The residue was purified *via* preparative HPLC.

(*S*)-*N*-(1-oxo-5,6,7,7a-tetrahydro-1*H*-pyrrolizin-3-yl)hexanamide / (*S*)-pyrrolizixenamide A (**2a**)

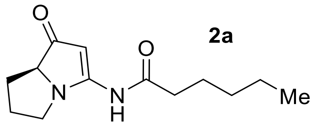

2a

This compound was synthesized from *tert*-butyl (*S*)-3-amino-1-oxo-5,6,7,7a-tetrahydro-1*H*-pyrrolizine-2-carboxylate and hexanoyl chloride, yielding 11.5 mg of a yellowish oil (TFA salt from HPLC solvents; 48.7 µmol, 39% over 2 steps).

^{1}H-NMR (600 MHz, $CDCl_3$)**:** δ [ppm] = 11.28 (bs, 1 H), 8.59 (bs, 1 H), 5.97 (s, 1 H), 4.28 (dd, *J* = 10.6, 6.7 Hz, 1 H), 3.67–3.60 (m, 1 H), 3.31–3.23 (m, 1 H), 2.52 (t, *J* = 7.5 Hz, 2 H), 2.39–2.23 (m, 3 H), 1.64 (qnt, *J* = 7.4 Hz, 2 H), 1.53–1.44 (m, 1 H), 1.35–1.27 (m, 4 H), 0.88 (t, *J* = 6.9 Hz, 3 H).

^{13}C {^{1}H}-NMR (151 MHz, $CDCl_3$)**:** δ [ppm] = 193.5, 173.0, 170.2, 162.0 (q, *J* = 37.0 Hz), 116.3 (q, *J* = 291 Hz), 93.7, 69.9, 47.4, 37.0, 31.2, 29.2, 26.8, 24.3, 22.5, 14.0.

^{19}F {^{1}H}-NMR (282 MHz, $CDCl_3$)**:** δ [ppm] = –75.96 (bs).

HR-MS (ESI+)**:** *m/z* calcd. for $C_{13}H_{20}N_2O_2$ $[M+H]^+$: 237.1598, found: 237.1603.

Specific rotation: $[\alpha]_D \sim 0$ (ρ = 0.48; $CHCl_3$); cf. Lit.: +0.9 (ρ = 0.57; MeOH).[3]

(*S*)-*N*-(1-oxo-5,6,7,7a-tetrahydro-1*H*-pyrrolizin-3-yl)decanamide (**2c**)

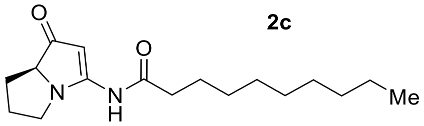

2c

This compound was synthesized from *tert*-butyl (*S*)-3-amino-1-oxo-5,6,7,7a-tetrahydro-1*H*-pyrrolizine-2-carboxylate and decanoyl chloride, yielding 19.0 mg of a white solid (65.0 µmol, 39% over 2 steps).

^{1}H-NMR (600 MHz, $CDCl_3$)**:** δ [ppm] = 10.68 (bs, 1 H), 5.74 (s, 1 H), 3.97 (bt, *J* = 8.5 Hz, 1 H), 3.46 (ddd, *J* = 11.1, 7.6, 3.5 Hz, 1 H), 3.19 (dt, 11.0, 7.6 Hz, 1 H), 2.53–2.44 (m, 2 H), 2.23–2.13 (m, 2 H), 2.13–2.05 (m, 1 H), 1.65 (qnt, 7.6 Hz, 2 H), 1.58–1.50 (m, 1 H), 1.34–1.21 (m, 12 H), 0.86 (t, *J* = 7.0 Hz, 3 H).

^{13}C {^{1}H}-NMR (151 MHz, $CDCl_3$)**:** δ [ppm] = 200.5, 172.9, 171.1, 93.1, 69.5, 48.2, 37.1, 32.0, 29.6, 29.5, 29.4, 29.3, 28.4, 26.6, 25.0, 22.8, 14.2.

HR-MS (ESI+)**:** *m/z* calcd. for $C_{17}H_{28}N_2O_2$ $[M+H]^+$: 293.2224, found: 293.2231.

Specific rotation: $[\alpha]_D = -1.0\ ^{\circ}\cdot {}^{mL}/_{dm\cdot g}$ (ρ = 1.0; $CHCl_3$).

(*S*)-*N*-(1-oxo-5,6,7,7a-tetrahydro-1*H*-pyrrolizin-3-yl)tetradecanamide (**2d**)

2d

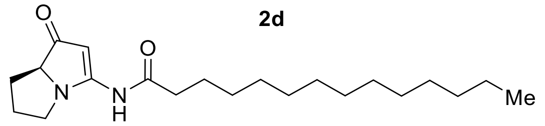

This compound was synthesized from *tert*-butyl (*S*)-3-amino-1-oxo-5,6,7,7a-tetrahydro-1*H*-pyrrolizine-2-carboxylate and tetradecanoyl chloride, yielding 16.5 mg of a white solid (9/1 mixture of free amine/TFA salt from HPLC solvents; 45.8 µmol, 36%).

^{1}H-NMR (600 MHz, $CDCl_3$)**:** δ [ppm] = 10.42 (s, 0.1 H), 10.07 (s, 0.8 H), 5.70 (s, 0.8 H), 4.31 (dd, *J* = 10.9, 6.9 Hz, 0.1 H), 3.92 (bt, *J* = 8.4 Hz, 0.9 H), 3.41 (ddd, *J* = 11.1, 7.5, 3.9 Hz, 0.9 H), 3.23–3.11 (m, 1 H), 2.55–2.51 (m, 0.3 H), 2.50–2.43 (m, 1.7 H), 2.38–2.26 (m, 0.5 H), 2.20–2.02 (m, 2.5 H), 1.75–1.54 (m, 3 H), 1.37–1.22 (m, 20 H), 0.87 (t, *J* = 7.0 Hz, 3 H).

^{13}C {^{1}H}-NMR (151 MHz, $CDCl_3$)**:** δ [ppm] = 201.6, 172.5, 170.1, 93.3, 69.5, 48.4, 37.2, 32.1, 29.8 (4C), 29.6, 29.5 (2C), 29.3, 28.1, 26.6, 25.0, 22.8, 14.3.

Only ^{13}C shifts of the free amine are given.

HR-MS (ESI+)**:** *m/z* calcd. for $C_{21}H_{36}N_2O_2$ $[M+H]^+$: 349.2850, found: 349.2853.

Specific rotation: $[\alpha]_D = -0.6\ ^{\circ}\cdot mL/_{dm\cdot g}$ (ρ = 0.89; MeOH).

3.3 Synthesis of simple substrate 53

2-methoxy-6-(3-((tetrahydro-2*H*-pyran-2-yl)oxy)propyl)pyridine (**SI-7**)[17]

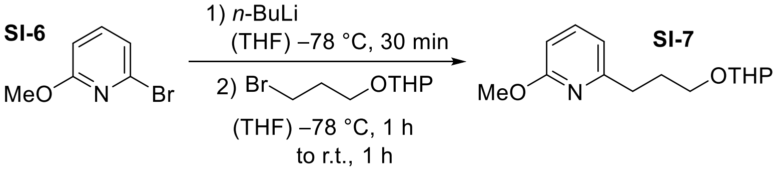

In a flame-dried 100 mL Schlenk flask, 0.98 mL 2-bromo-6-methoxypyridine (1.50 g, 8.0 mmol, 1.42 eq.) was dissolved in 15 mL THF and the mixture cooled to –78 °C. 3.2 mL *n*-BuLi (2.5 M in hexane; 0.51 g, 8.0 mmol, 1.42 eq.) was added dropwise and the solution stirred at –78 °C for 30 minutes. Next, 0.95 mL 2-(3-bromopropoxy)tetrahydro-2*H*-pyran (1.25 g, 5.62 mmol, 1.0 eq.) was added slowly and the mixture stirred at –78 °C for 30 minutes. Afterwards, the solution was allowed to warm to room temperature over 1 hour. The reaction was quenched by pouring it on ice water (15 mL). After extraction with EtOAc (3 × 20 mL), combined organic extracts were dried over Na_2SO_4, filtered and the solvent removed under reduced pressure. The residue was purified by flash column chromatography (hexane/EtOAc = 20/1 to 10/1) to yield 587 mg of a yellow oil (2.34 mmol, 42%; 61% brsm).

^{1}H-NMR (400 MHz, $CDCl_3$)**:** δ [ppm] = 7.45 (d, *J* = 8.2, 7.2 Hz, 1 H), 6.71 (d, *J* = 7.2 Hz, 1 H), 6.53 (d, *J* = 8.3 Hz, 1 H), 4.59 (dd, *J* = 4.4, 2.8 Hz, 1 H), 3.91 (s, 3 H), 3.90–3.77 (m, 2 H), 3.53–3.41 (m, 2 H), 2.85–2.70 (m, 2 H), 2.10–2.00 (m, 2 H), 1.90–1.78 (m, 1 H), 1.76–1.67 (m, 1 H), 1.63–1.43 (m, 4 H).

^{13}C {^{1}H}-NMR (100.6 MHz, $CDCl_3$)**:** δ [ppm] = 163.8, 159.7, 138.8, 115.3, 107.5, 99.0, 67.2, 62.4, 53.3, 34.5, 30.9, 29.3, 25.7, 19.8.

***R*$_f$** (hexane/EtOAc = 10/1) = 0.25.

HR-MS (ESI+)**:** *m/z* calcd. for $C_{14}H_{21}NO_3$ $[M+H]^+$: 252.1595, found: 252.1600; main signal resulting from acetal cleavage: *m/z* calcd. for $C_9H_{14}NO_2$ $[M+H]^+$: 168.1020, found: 168.1030.

NMR data matched those reported in literature.[17]

2,3-dihydroindolizin-5(1*H*)-one (**53**)[17]

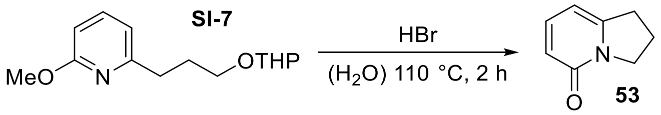

In a 10 mL round-bottomed flask, 108 mg 2-methoxy-6-(3-((tetrahydro-2*H*-pyran-2-yl)oxy)propyl)pyridine (0.43 mmol, 1.0 eq.) was dissolved in 2.45 mL 48% HBr_{aq} (1.74 g, 21.5 mmol, 50 eq.) and the mixture stirred at 110 °C for 2 hours. After cooling to room temperature, the solution was neutralized with 6 mL 4 M $NaOH_{aq}$ and extracted with DCM (2 × 20 mL). Combined extracts were dried over Na_2SO_4, filtered and the solvent removed under reduced pressure, yielding 52 mg of a brownish oil (0.38 mmol, 90%), which showed small (~5 w-%) contamination with starting material. A small amount was purified by preparative HPLC, yielding a white solid, which was used for enzyme assays.

^{1}H-NMR (600 MHz, $CDCl_3$)**:** δ [ppm] = 7.30 (dd, *J* = 9.0, 6.8 Hz, 1 H), 6.38 (ddt, *J* = 9.1, 1.0, 0.9 Hz, 1 H), 6.10 (ddt, *J* = 6.8, 1.2, 1.2 Hz, 1 H), 4.16–4.12 (m, 2 H), 3.09–3.05 (m, 2 H), 2.21–2.15 (m, 2 H).

^{13}C {^{1}H}-NMR (100.6 MHz, $CDCl_3$)**:** δ [ppm] = 162.4, 150.6, 140.0, 116.8, 101.2, 48.6, 31.7, 21.1.

HR-MS (ESI+)**:** *m/z* calcd. for C_8H_9NO $[M+H]^+$: 136.0757, found: 136.0766.

NMR data matched those reported in literature.[18]

4. Additional Figures

4.1 Stereochemical investigation of alcohols 26

Exemplarily shown for C_6 compound **26a**

- Estimated dihedral angles θ after energy optimization (by MMFF, with R = H) and corresponding $^3J_{H-H}$ coupling constants, estimated by Bothner-By eq. ($^3J_{H-H} = 7 - \cos\theta + 5\cos(2\theta)$)[19, 20]

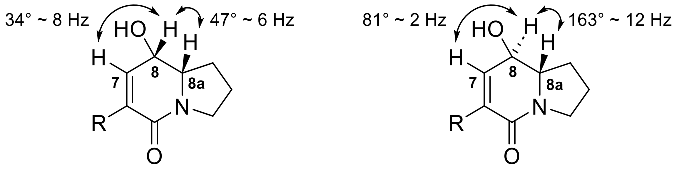

(8*S*,8a*S*)-**26a** (8*R*,8a*S*)-**26a**

- Excerpt from the ^{1}H-NMR spectrum of **26a**, directly after purification (R = –NHCOC$_5$H$_{13}$)

N-((8aS)-8-hydroxy-5-oxo-1,2,3,5,8,8a-hexahydroindolizin-6-yl)hexanamide

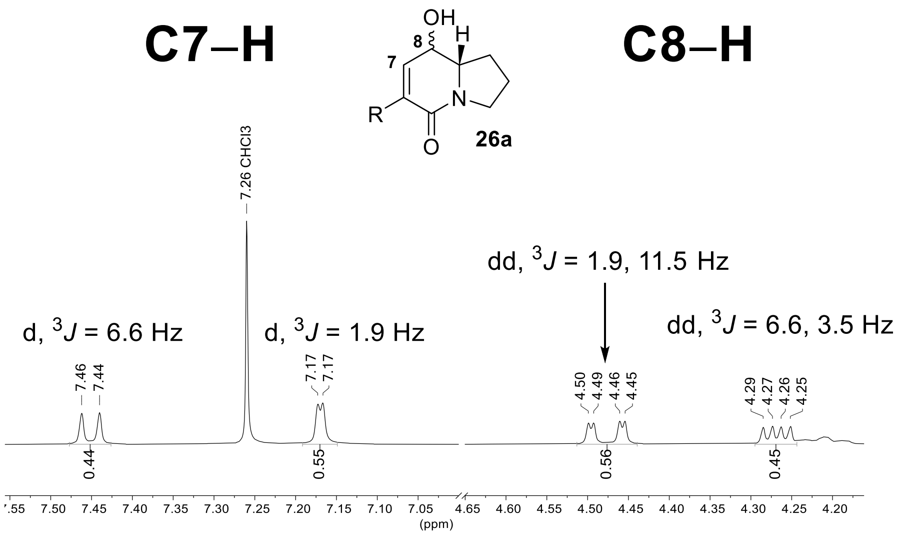

Figure S8. Excerpt from ^{1}H-NMR spectrum of **26a**.

- Fitting of predicted to observed data (R = –NHCOC$_5$H$_{13}$)

N-((8aS)-8-hydroxy-5-oxo-1,2,3,5,8,8a-hexahydroindolizin-6-yl)hexanamide

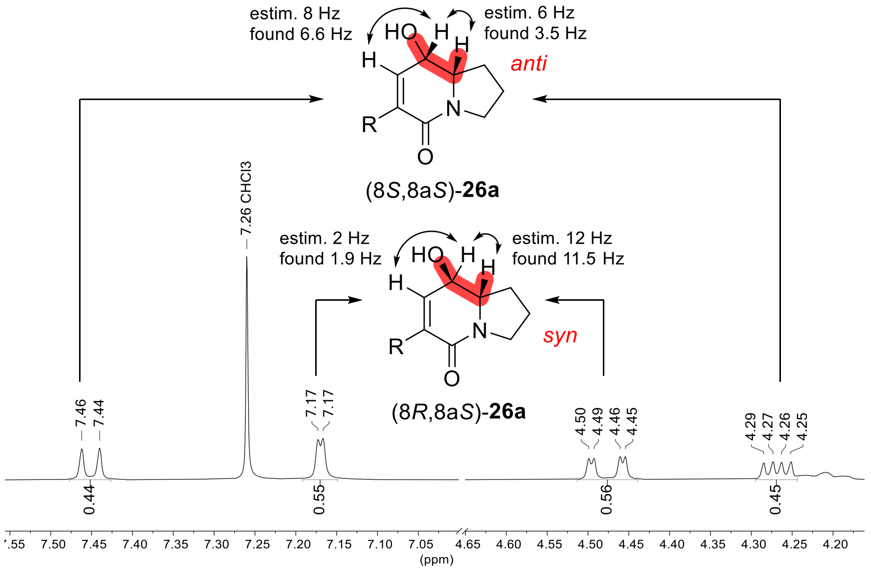

Figure S9. Excerpt from ^{1}H-NMR spectrum of **26a** with fitting of estimated to observed data.

- Comparison of ^{1}H-NMR spectra of **26a**, directly after purification and after standing in $CDCl_3$ for one day (R = $–NHCOC_5H_{13}$)

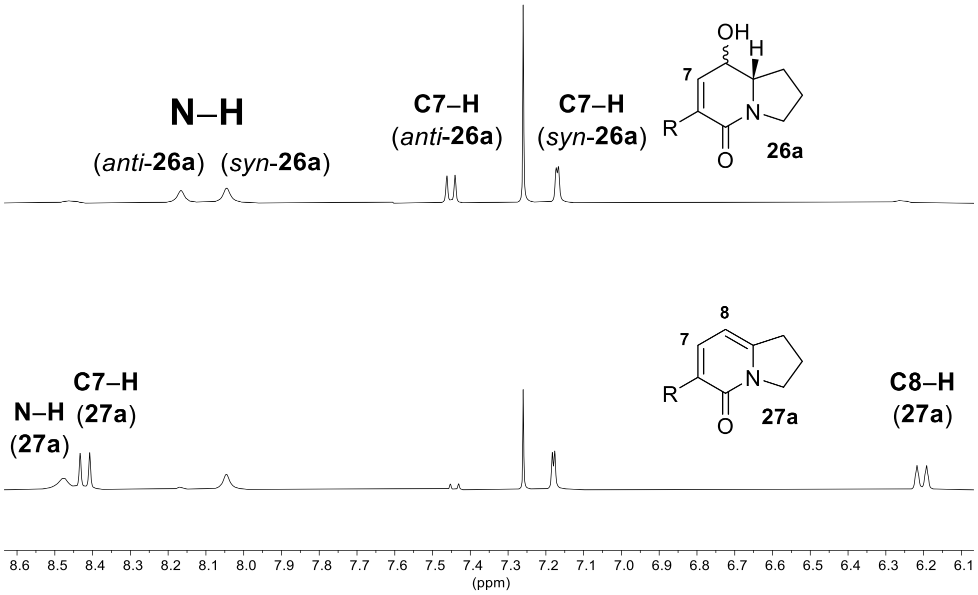

Figure S10. Excerpts from ^{1}H-NMR spectra of **26a**. Top: directly after purification; bottom: after standing in $CDCl_3$ for one day.

These investigations support the assumption that the labile compound is (*anti*)-**26a**, which readily eliminates water in an E2 mechanism to form **27a**:

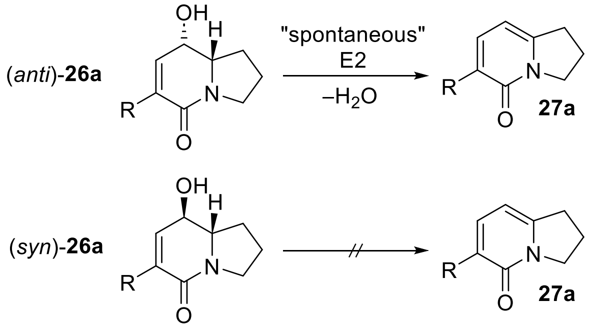

Figure S11. Explanation of the observed spontaneous elimination of one isomer of alcohol **26a** by molecular geometry, as investigated by ^{1}H-NMR.

4.2 Mechanistic investigation of 'Swern elimination' from 26 to 27

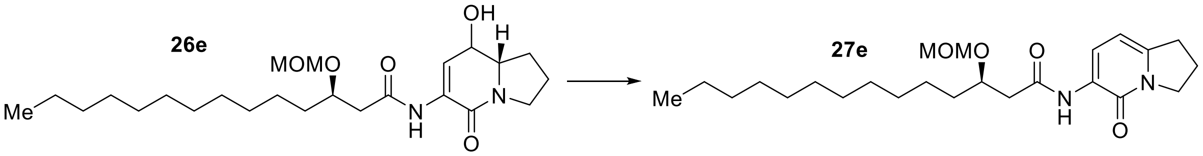

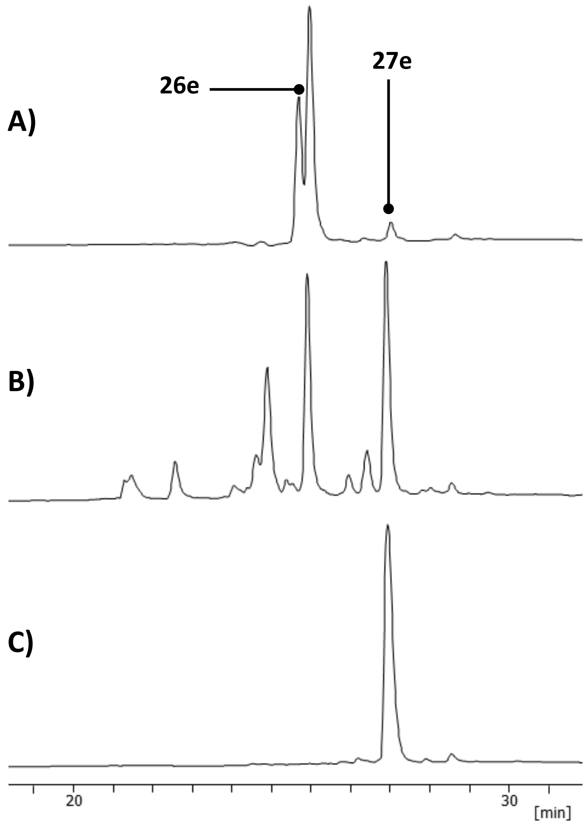

Figure S12. Investigation of transformation of **26e** to **27e** under different conditions by HPLC. A) After refluxing with 3.0 eq. NEt_3 in DCM for 2.5 hours: only minute amounts of **27e** observed. B) After stirring at 0 °C with 2.0 eq. HCl in dioxane for 2 hours: elimination of one isomer of **26e** with formation of **27e**, with observation of several side products. C) After Swern conditions (DMSO (3.0 eq.), $(COCl)_2$ (2.2 eq.), –78 °C, 30 min) and aqueous workup, but *before* addition of NEt_3: complete and clean transformation of both isomers of **26e** to **27e**.

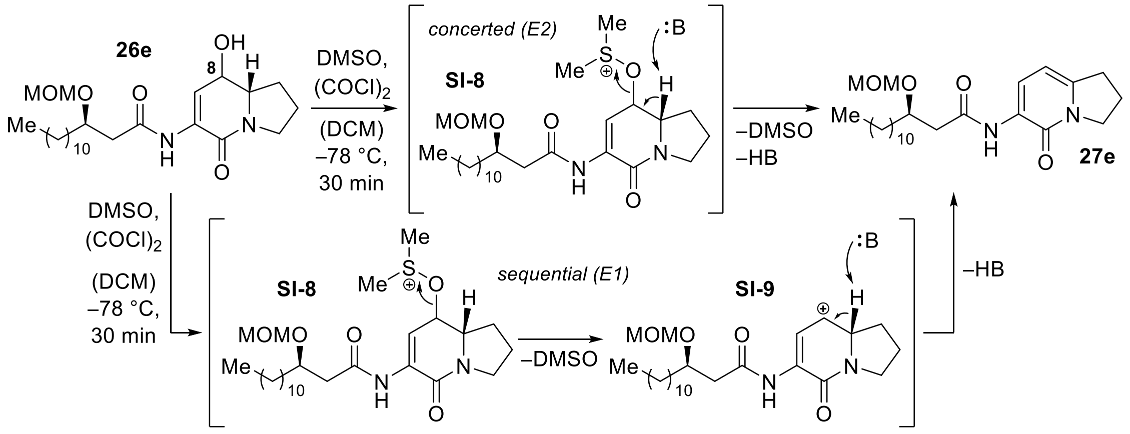

Figure S13. Proposed mechanism for the 'Swern elimination': After addition of **26e** to the preformed chloro(dimethyl)sulfonium chloride, the intermediate alkoxysulfonium ion **SI-8** directly eliminates DMSO with concomitant (E2, top) or subsequent (E1, bottom; *via* carbocation **SI-9**) proton abstraction to form the olefin **27e**. A combination of both mechanisms is assumed, with a preference dependent on the stereochemical configuration of *C*-8 in **26** (cf. chapter 4.1). Additionally, the stability of **SI-9** (allylic carbocation) must be considered, rather indicating an E1 mechanism. B – (weak) base, e.g., Cl^- or H_2O.

4.3 UV spectra of late-stage synthetic compounds
UV spectra have been recorded by DAD-HPLC analysis (gradient and solvents as described in chapter 1.1). Absorption maxima are given in [nm].

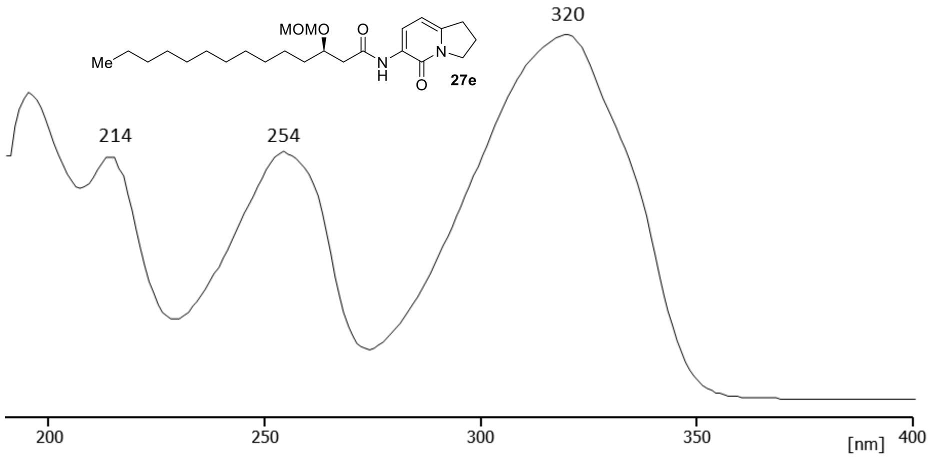

Figure S14. UV-spectrum of compound **27e**.

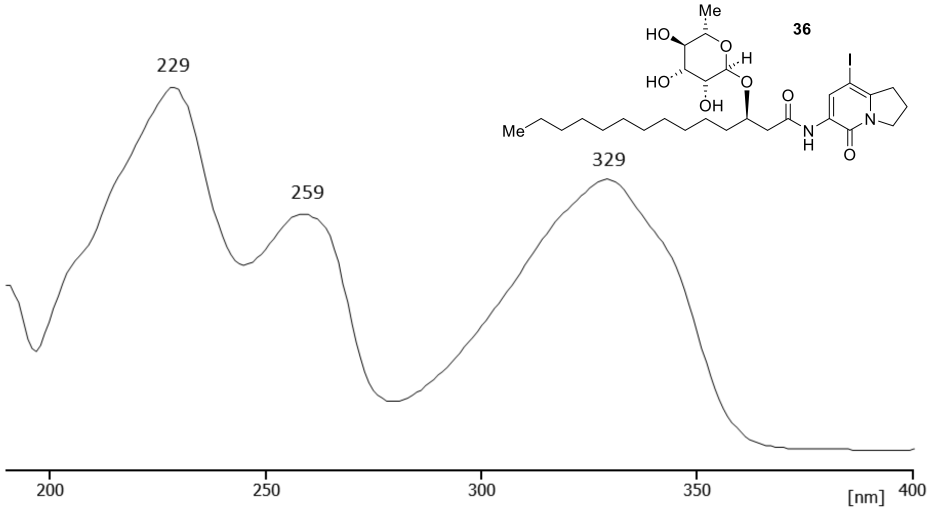

Figure S15. UV-spectrum of compound **36**.

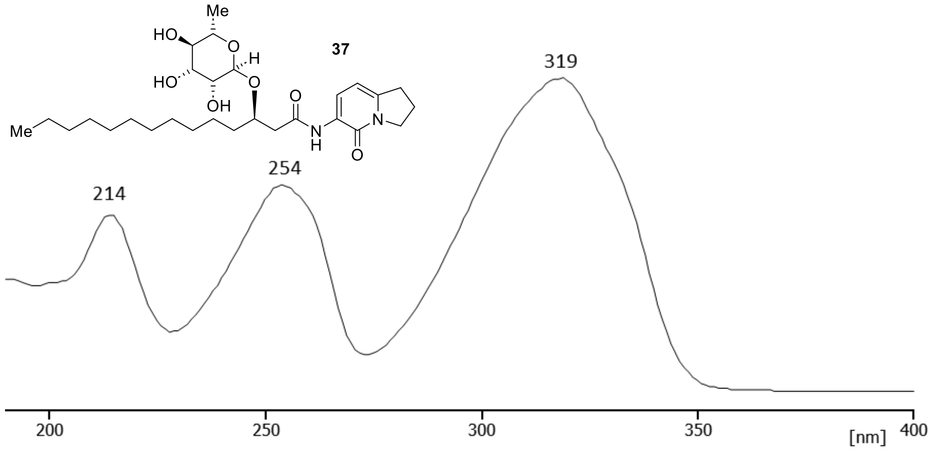

Figure S16. UV-spectrum of compound **37**.

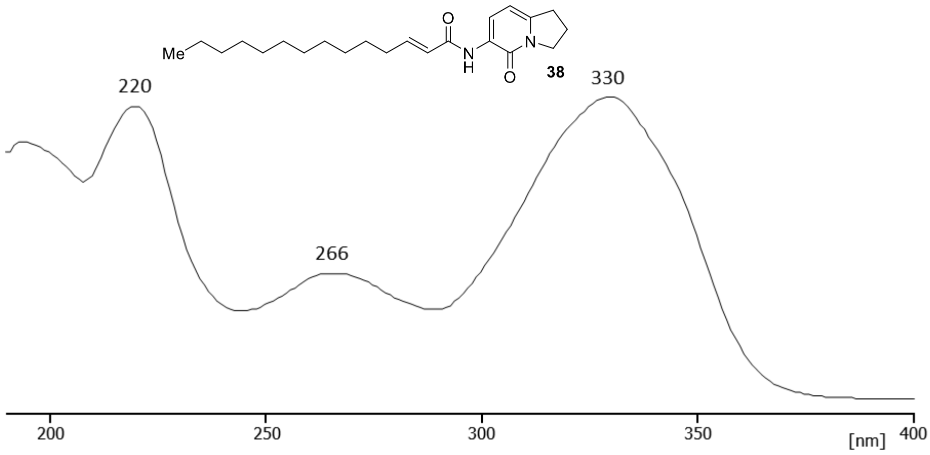

Figure S17. UV-spectrum of compound **38**.

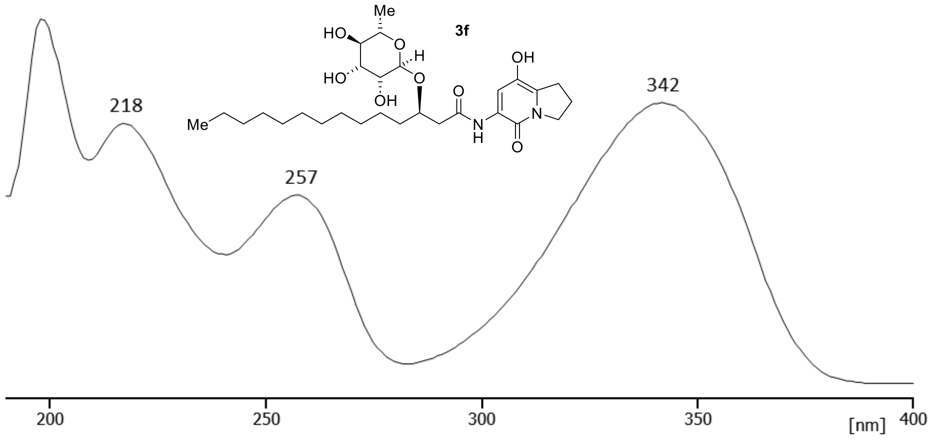

Figure S18. UV-spectrum of compound **3f**.

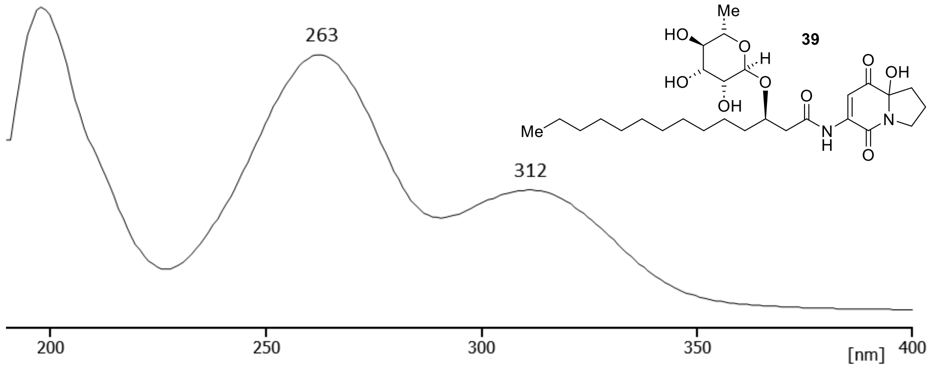

Figure S19. UV-spectrum of compound **39**.

4.4 Detailed MS/MS fragment characterization of 4c and 1c

After close investigation of the MS/MS spectra of the product pairs **4** / **1**, small, but decisive differences in the fragmentation patterns were observed. Most prominently, we detected the presence *vs*. absence of a fragmentation signal in accordance with decarboxylation in **4** *vs.* **1**, respectively. Additionally, an abundant fragment at *m/z* 139 *vs*. *m/z* 138 was observed in the MS/MS spectra of **4** *vs.* **1**, respectively (MS/MS spectra of **4c** (from **3c** PxaB assay) and **1c** (from **3c** BraC assay) exemplarily shown in Fig. S20 and S21). Analysis of the MS/MS spectra of synthetic brabantamide product standard **1c** (Fig. S22) revealed the absence of a signal corresponding to decarboxylation and the presence of a signal at *m/z* 138.

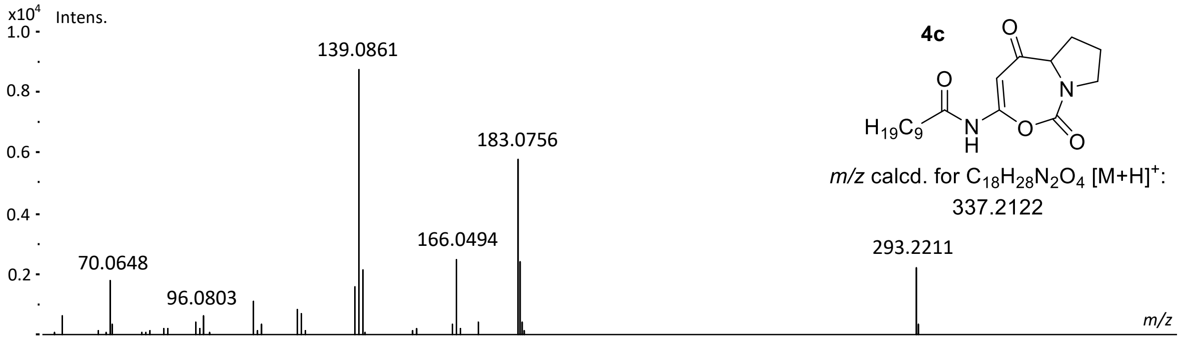

Figure S20. CID MS/MS spectrum (*m/z* 337.2109) from PxaB assay with substrate **3c**, retention time 19.4 minutes, thus identifying **4c**.

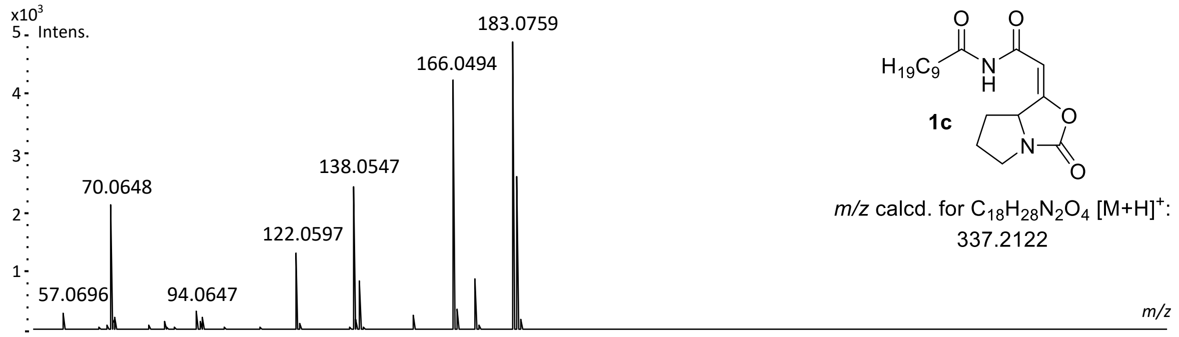

Figure S21. CID MS/MS spectrum (*m/z* 337.2109) from BraC assay with substrate **3c**, retention time 20.3 minutes, thus identifying **1c**.

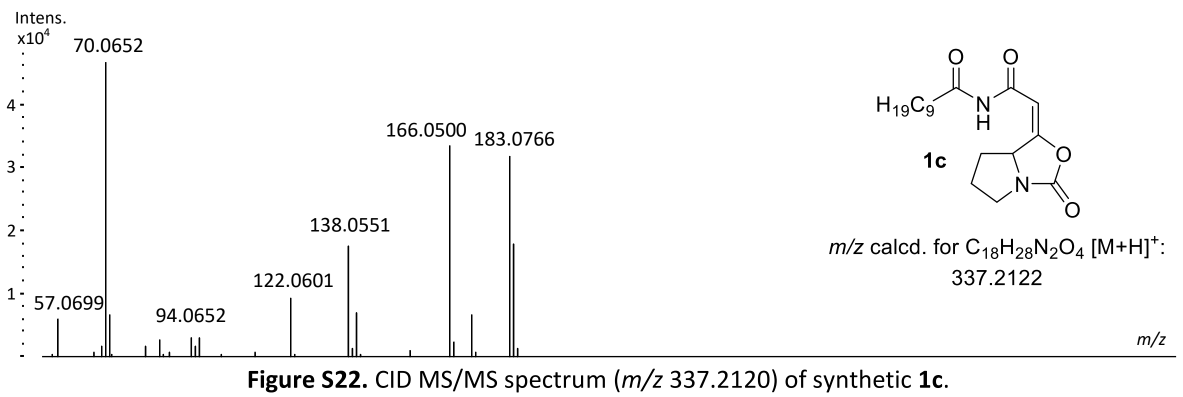

Figure S22. CID MS/MS spectrum (*m/z* 337.2120) of synthetic **1c**.

We next compared the expected MS/MS fragments of **4c** and **1c** (cf. Fig. S23) to those observed in obtained MS/MS spectra of **4c** (Fig. S20) and **1c** (Fig. S21/S22). Fragments observed in both spectra at *m/z* 70, 166, and 183 can be explained by identical (**F1**) or structurally different (**F3**/**F9**, **F4**/**F10**) product ions. The decisive signals at *m/z* 293 (**F5**, formal decarboxylation) and 139 (**F2**, formal decarboxylation and cleavage of amide bond) only present in MS/MS spectra of **4c** can be assigned to specific fragments

(Fig. S23, blue boxes). This fragment **F2** is also very abundant in MS/MS spectra of compounds **2** (see chapter 5.2.2.3). The diagnostic signal at *m/z* 138 can be attributed to **F8** of brabantamide **1c** (Fig. S23, orange box). These assignments hence allowed for unambiguous identification of the structures of products **4c** and **1c**, respectively (and analogously for all other tested substrate derivatives).

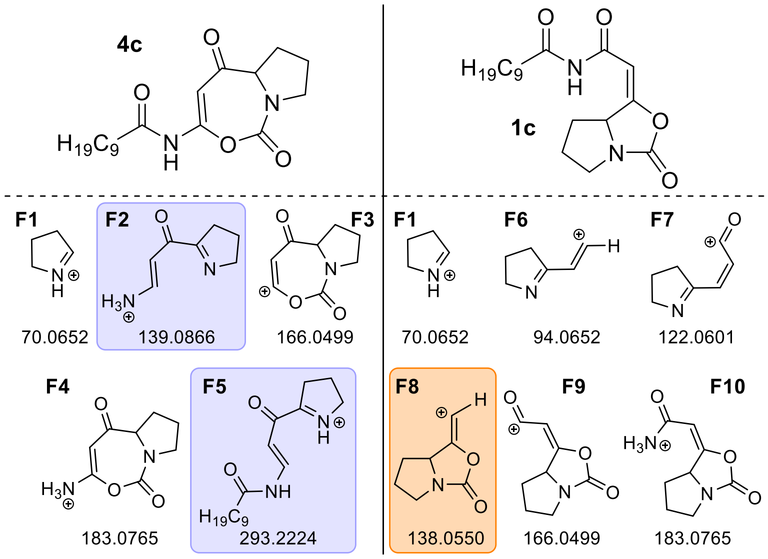

Figure S23. Theoretical MS/MS fragments with calculated *m/z* ratios for assignment of most abundant observed mass fragments, shown for **4c** and **1c**. Fragments allowing product discrimination highlighted in blue (**4c**) and orange (**1c**) boxes.

5. HPLC / MS Data

5.1 Chromatograms of product standards

5.1.1 Pyrrolizixenamide analogs

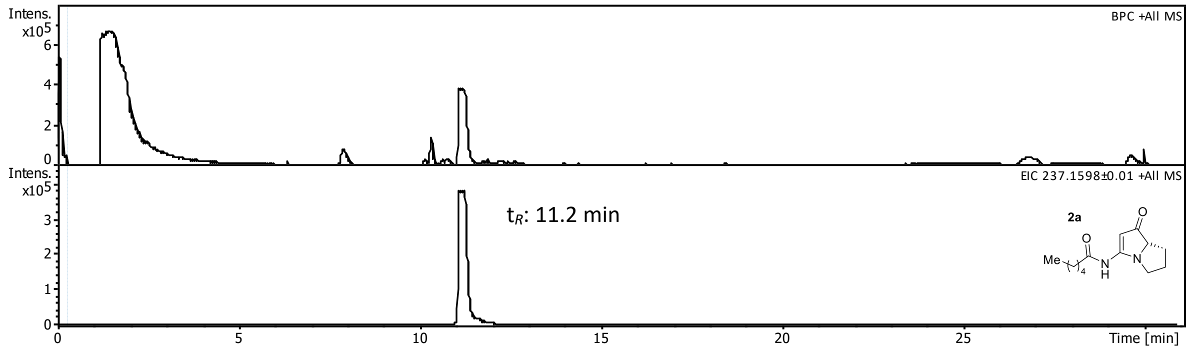

Figure S24. Base peak chromatogram (BPC) and extracted ion chromatogram (EIC) of C_6-PA-standard **2a**.

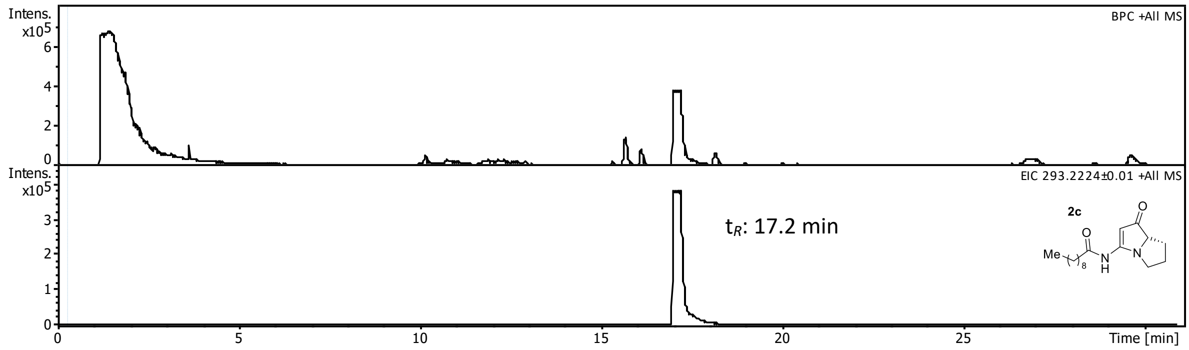

Figure S25. Base peak chromatogram (BPC) and extracted ion chromatogram (EIC) of C_{10}-PA-standard **2c**.

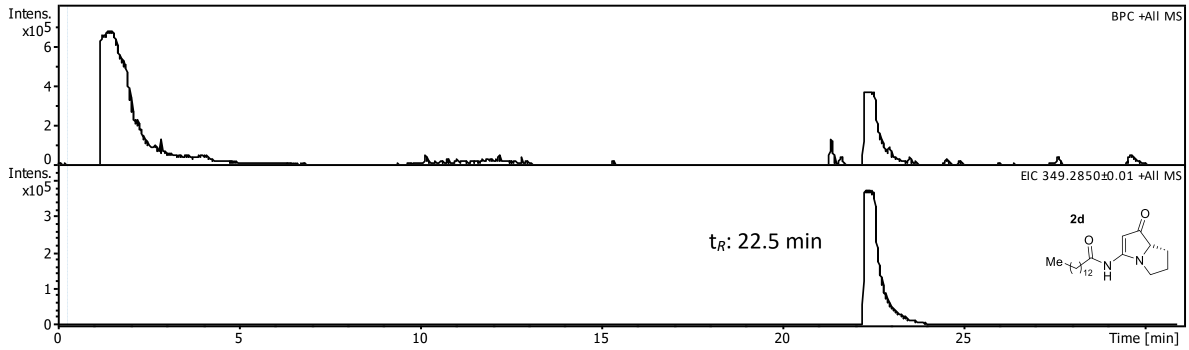

Figure S26. Base peak chromatogram (BPC) and extracted ion chromatogram (EIC) of C_{14}-PA-standard **2d**.

5.1.2 Brabantamide analogs

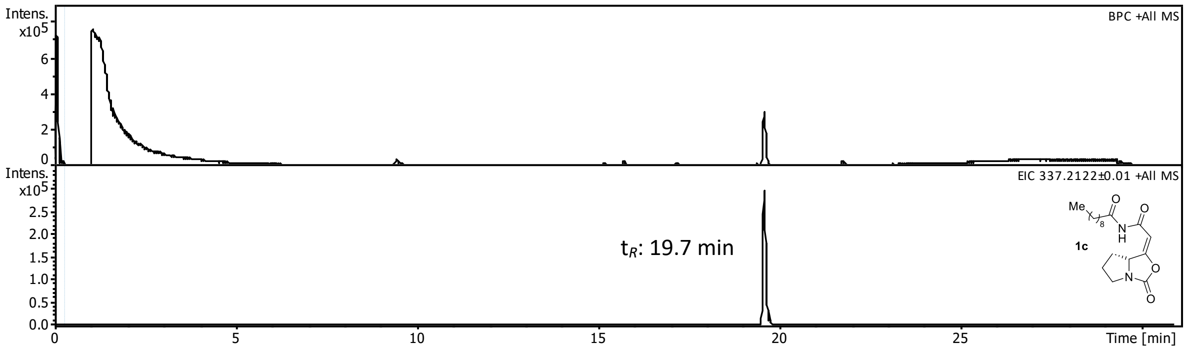

Figure S27. Base peak chromatogram (BPC) and extracted ion chromatogram (EIC) of C_{10}-brabantamide-standard **1c**.

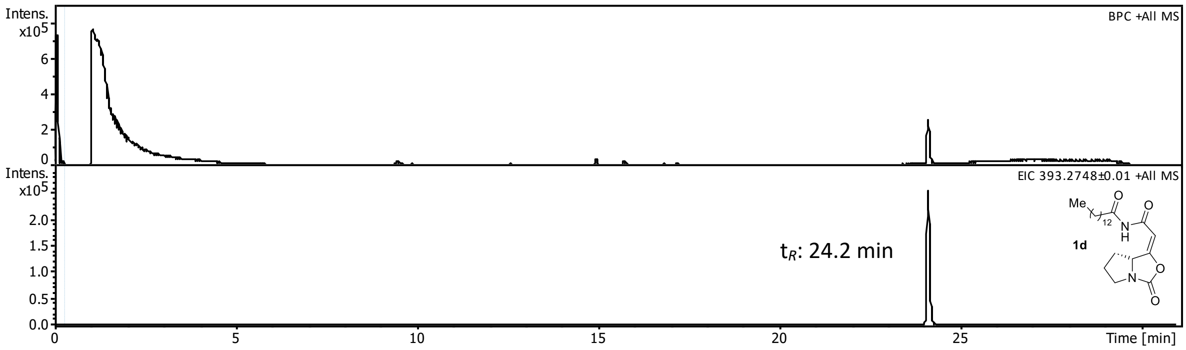

Figure S28. Base peak chromatogram (BPC) and extracted ion chromatogram (EIC) of C_{14}-brabantamide-standard **1d**.

5.2 MS spectra of substrates and product standards

All investigated compounds showed to be relatively easy to ionize by ESI(+). With collision-induced dissociation (CID) in auto-MS/MS mode (collision energy 20‑50 eV), characteristic MS/MS spectra were obtained, which were suitable for unambiguous product identification.

5.2.1 MS data

5.2.1.1 Substrates **3**

Even with the relatively mild ESI method, in-source fragmentation takes place. Thus, in addition to the $[M+H]^+$ ions, Y-type ions (*m/z* 167) could be generated by alpha-cleavage of the amide bond.

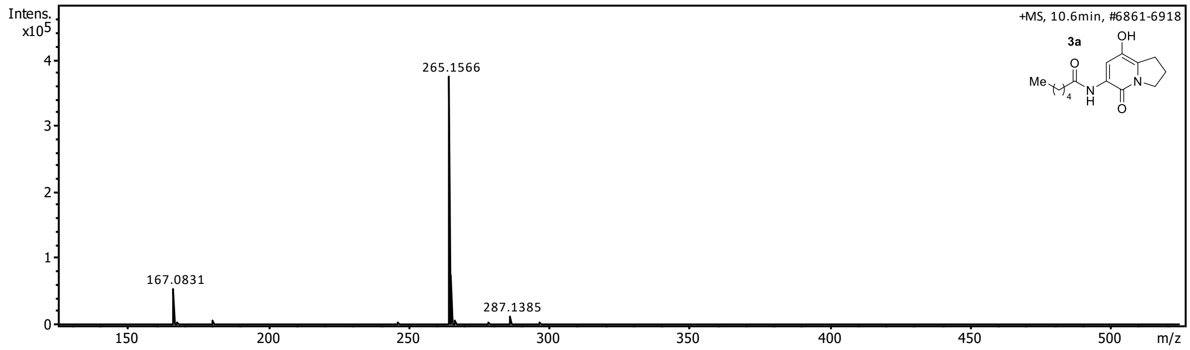

Figure S29. ESI+ mass spectrum of C_6-substrate **3a** from HPLC.

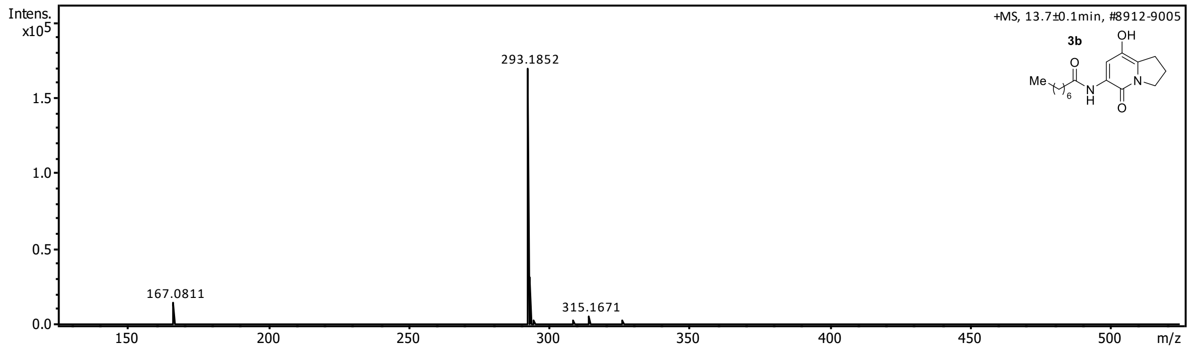

Figure S30. ESI+ mass spectrum of C_8-substrate **3b** from HPLC.

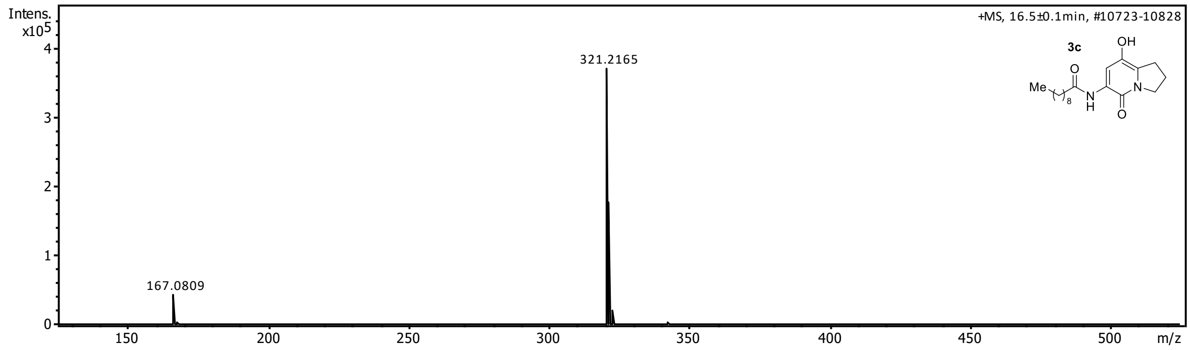

Figure S31. ESI+ mass spectrum of C_{10}-substrate **3c** from HPLC.

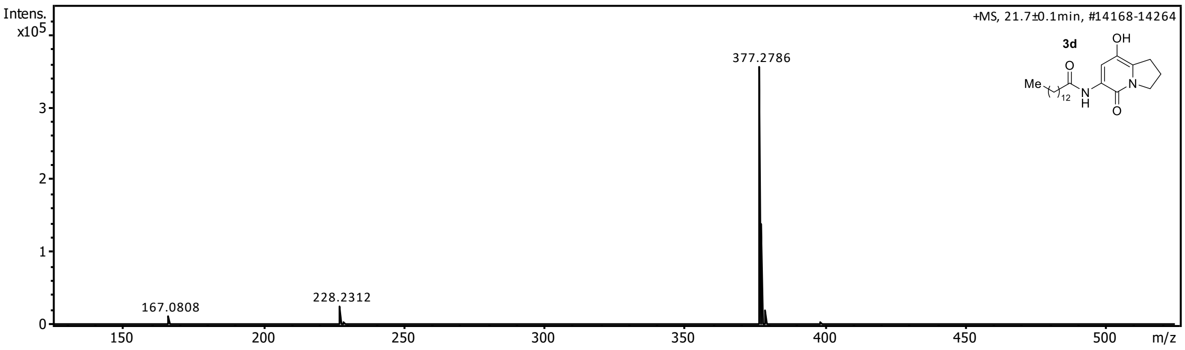

Figure S32. ESI+ mass spectrum of C_{14}-substrate **3d** from HPLC.

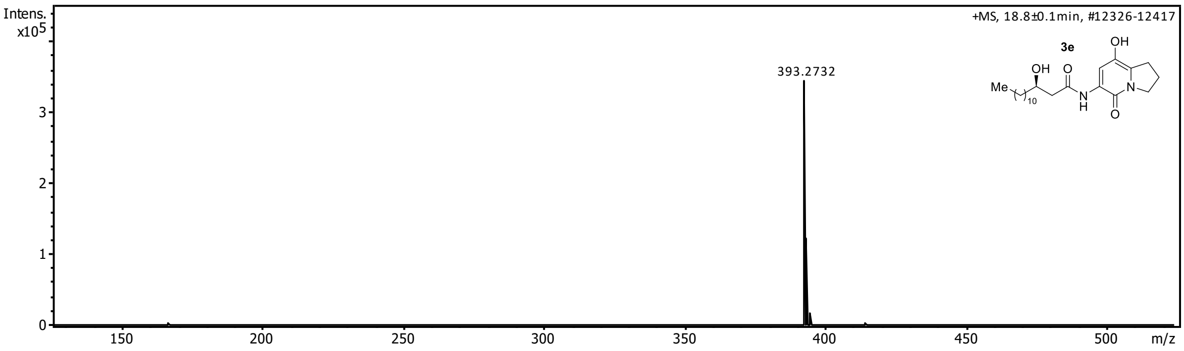

Figure S33. ESI+ mass spectrum of C_{14}OH-substrate **3e** from HPLC.

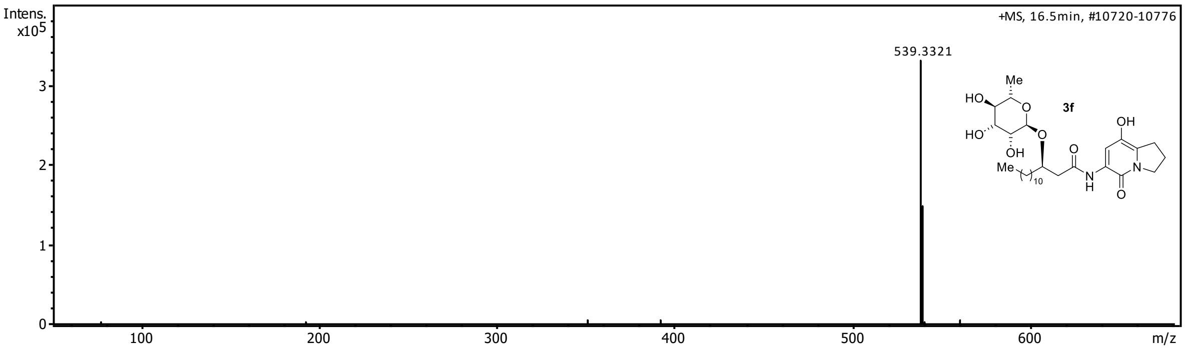

Figure S34. ESI+ mass spectrum of C_{14}ORha-substrate **3f** from HPLC.

5.2.1.2 Brabantamide analogs 1c/d

Caused by in-source fragmentation, alpha-cleavage of the imide bond leads to additional ions (*m/z* 183) next to the $[M+H]^+$ ions.

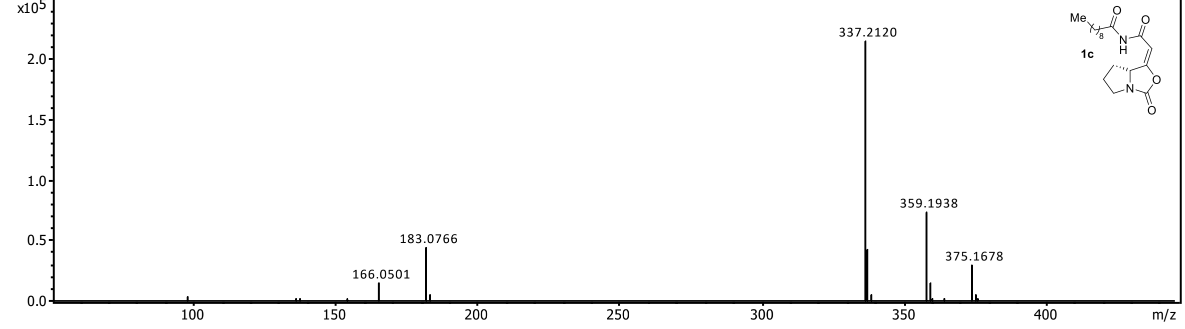

Figure S35. ESI+ mass spectrum of C_{10}-brabantamide-standard **1c** from HPLC.

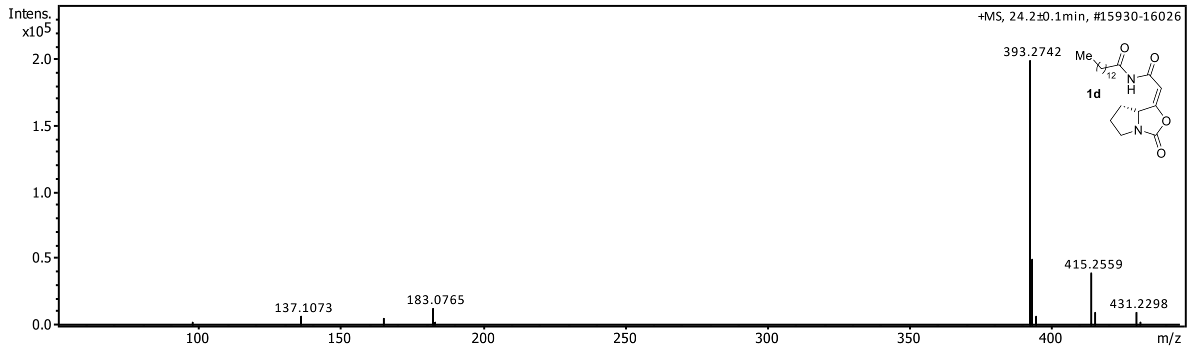

Figure S36. ESI+ mass spectrum of C_{14}-brabantamide-standard **1d** from HPLC.

5.2.1.3 Pyrrolizixenamide analogs **2a/c/d**

Caused by in-source fragmentation, alpha-cleavage of the amide bond leads to additional ions (*m/z* 139) next to the $[M+H]^+$ ions.

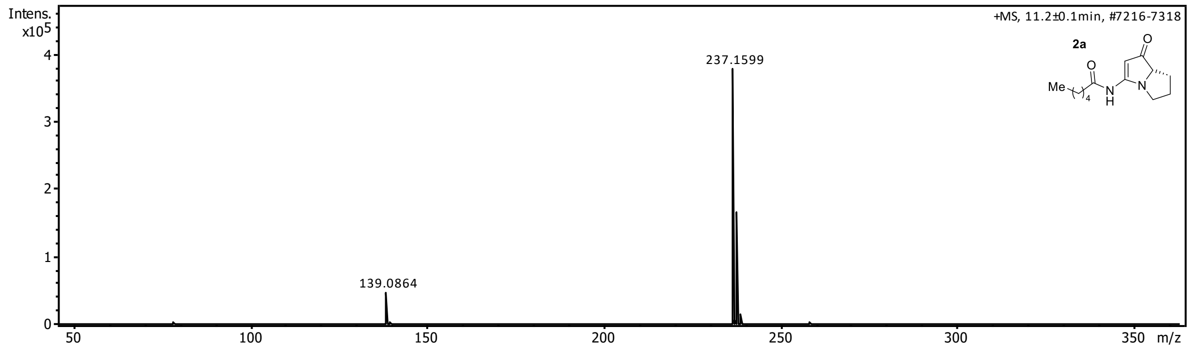

Figure S37. ESI+ mass spectrum of C_6-pyrrolizixenamide-standard **2a** from HPLC.

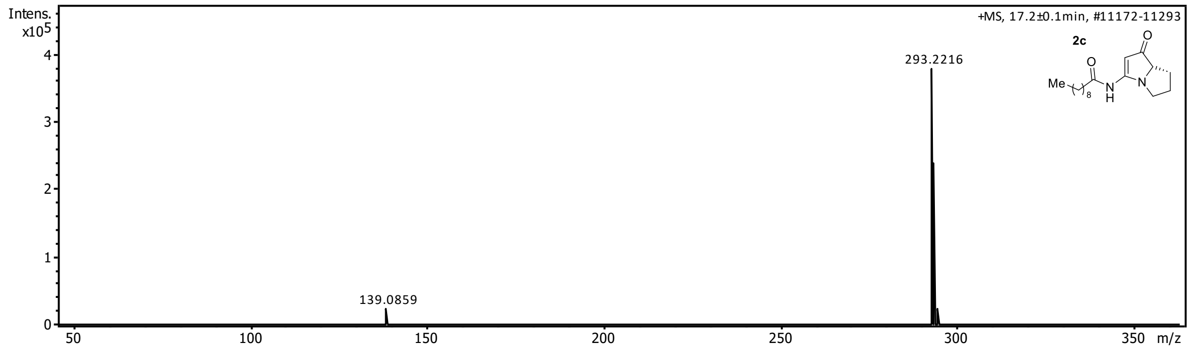

Figure S38. ESI+ mass spectrum of C_{10}-pyrrolizixenamide-standard **2c** from HPLC.

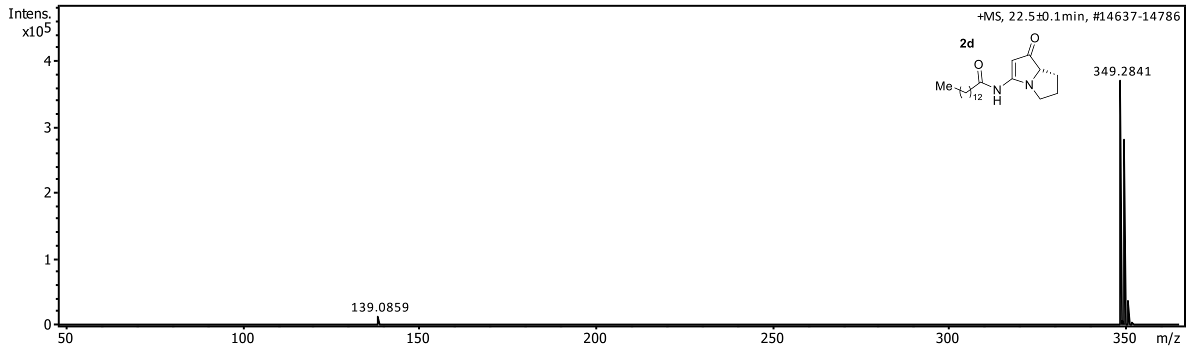

Figure S39. ESI+ mass spectrum of C_{14}-pyrrolizixenamide-standard **2d** from HPLC.

5.2.2 MS/MS data

5.2.2.1 Substrates **3**

MS/MS spectra of the substrates are dominated by a very intense peak caused by cleavage of the amide bond (*m/z* 167). A cleavage of ethylene leads to an ion with *m/z* 139. The cleavage of CO produces an ion with *m/z* 111. In addition, for **3e** a water cleavage from the $[M+H]^+$ ion can be shown. For **3f**, cleavage of the glycosidic bond can be observed $[M+H–146]^+$.

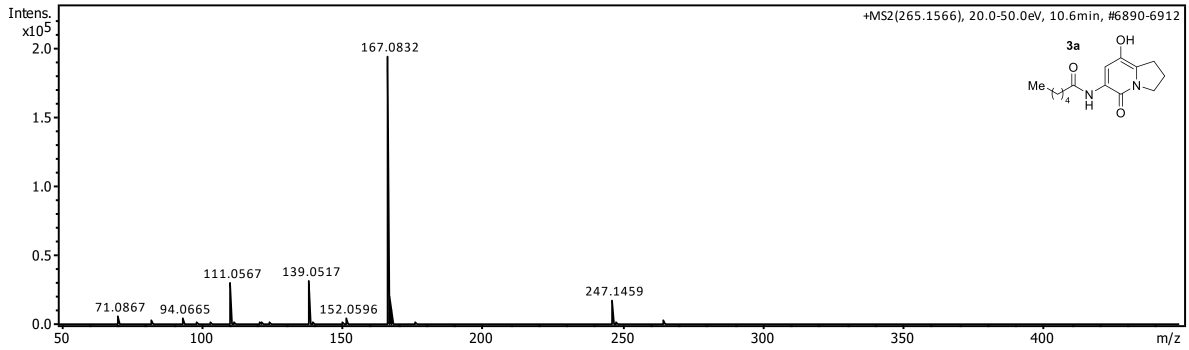

Figure S40. ESI+ CID MS/MS spectrum of C_6-substrate **3a** from HPLC.

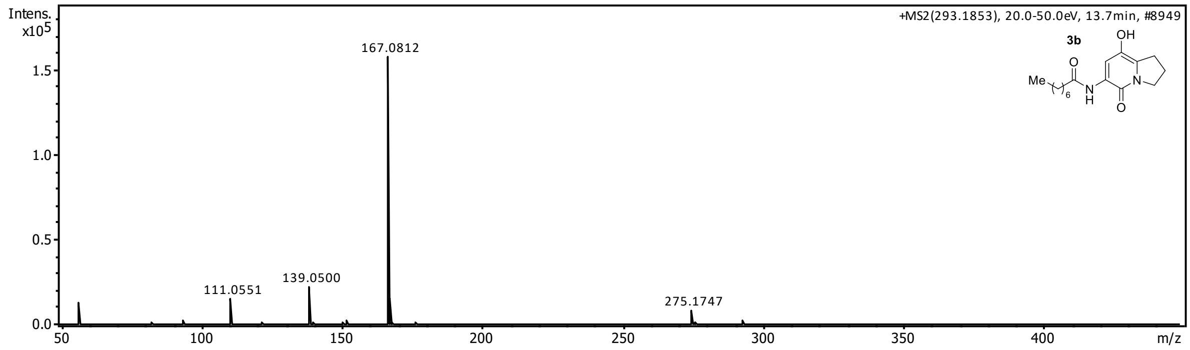

Figure S41. ESI+ CID MS/MS spectrum of C_8-substrate **3b** from HPLC.

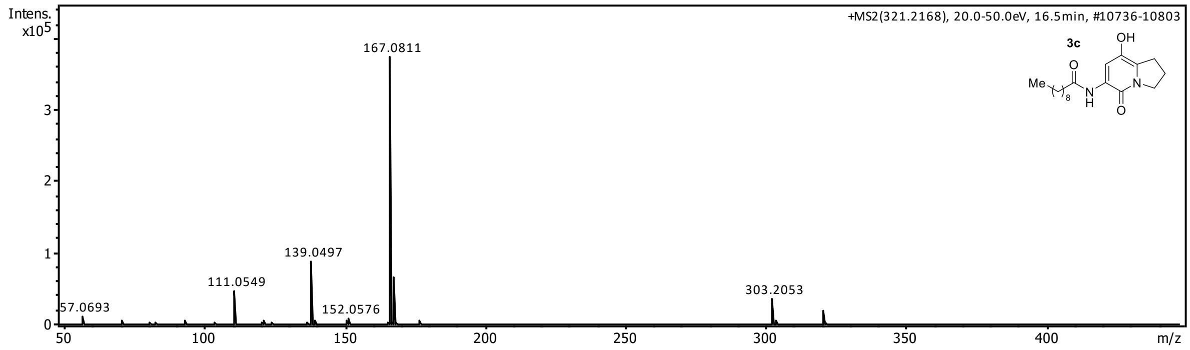

Figure S42. ESI+ CID MS/MS spectrum of C_{10}-substrate **3c** from HPLC.

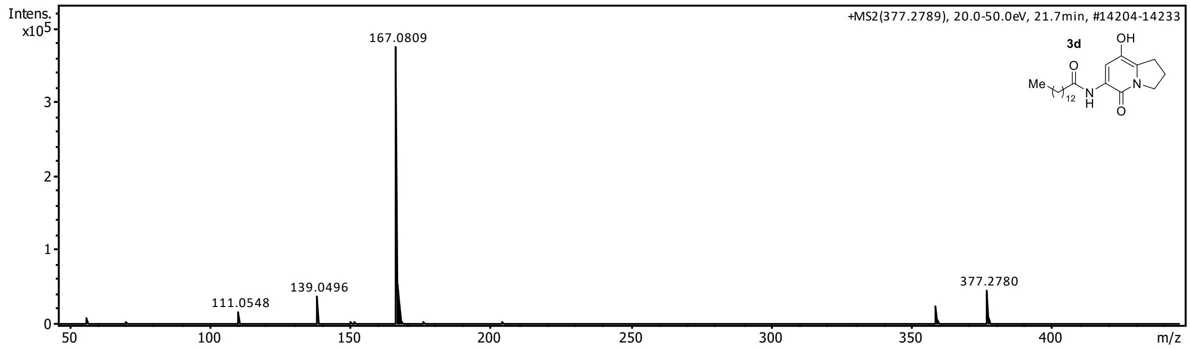

Figure S43. ESI+ CID MS/MS spectrum of C_{14}-substrate **3d** from HPLC.

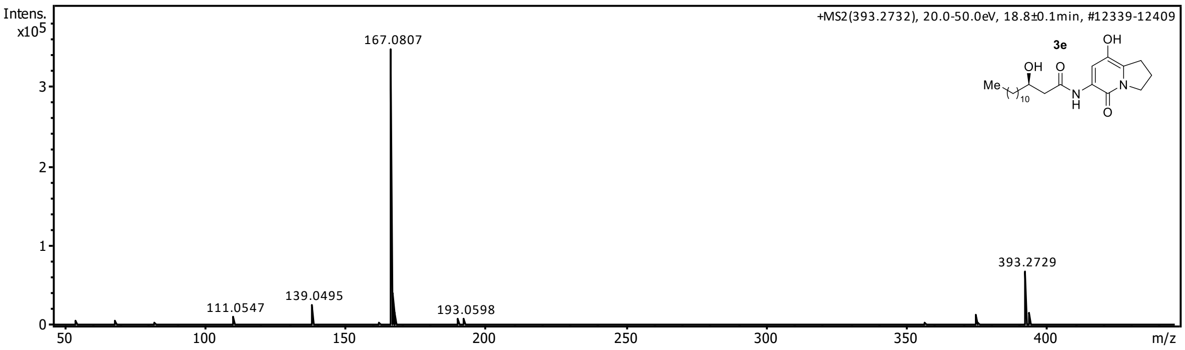

Figure S44. ESI+ CID MS/MS spectrum of C_{14}OH-substrate **3e** from HPLC.

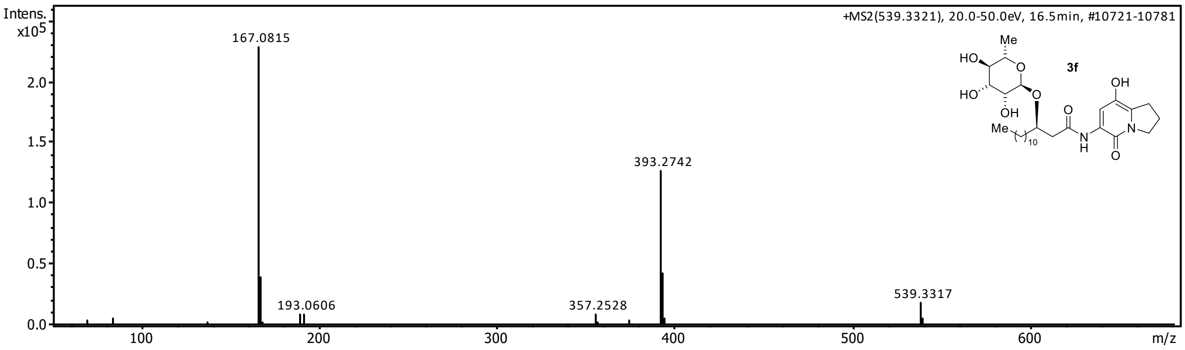

Figure S45. ESI+ CID MS/MS spectrum of C_{14}ORha-substrate **3f** from HPLC.

5.2.2.2 Brabantamide analogs 1c/d

The dihydropyrrolium ion (*m/z* 70) is dominating the MS/MS spectra of the brabantamide analogs. In addition, expected cleavage product ions (*m/z* 138, 166, 183) are seen, which further cleave CO_2 (*m/z* 94, 122, 139). An interesting peak can be detected at *m/z* 184, which can only be formed by rearrangement of the imide group to an imidic anhydride and subsequent (C=NH)–O cleavage. This ion also splits off CO_2 (*m/z* 140). Ions resulting from the cleaved side chains are also observable (e.g., *m/z* 172, 228).

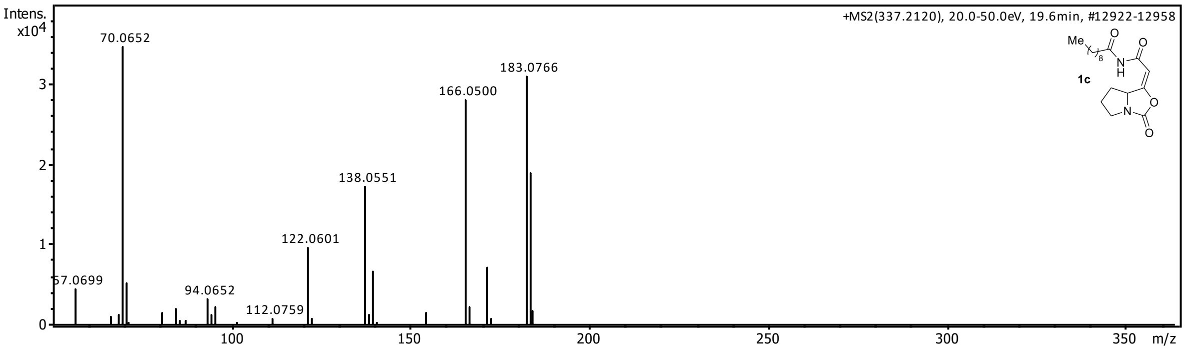

Figure S46. ESI+ CID MS/MS spectrum of C_{10}-brabantamide-standard **1c** from HPLC.

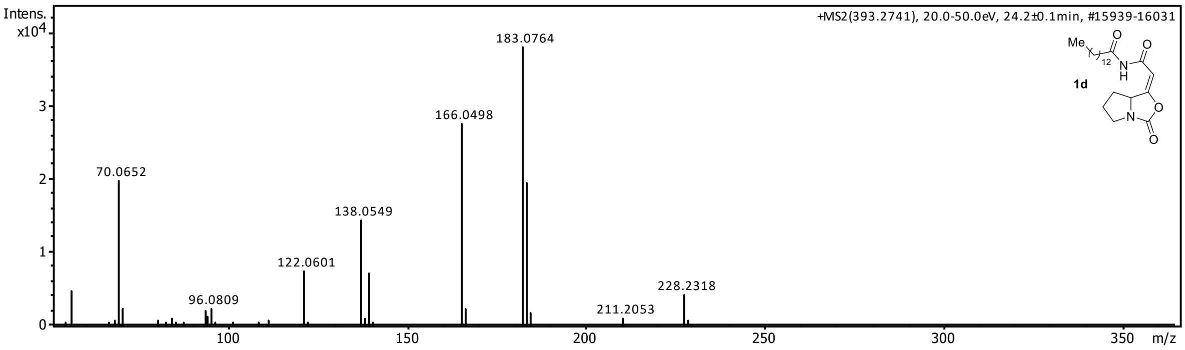

Figure S47. ESI+ CID MS/MS spectrum of C_{14}-brabantamide-standard **1d** from HPLC.

5.2.2.3 Pyrrolizixenamide analogs **2a/c/d**

The MS/MS spectra of pyrrolizixenamides show a very intense peak caused by cleavage of the amide bond (*m/z* 139) and a dominant peak at *m/z* 110 ($C_6H_8NO^+$). Another characteristic feature is the cleavage of a dihydropyrrolium ion (*m/z* 70).

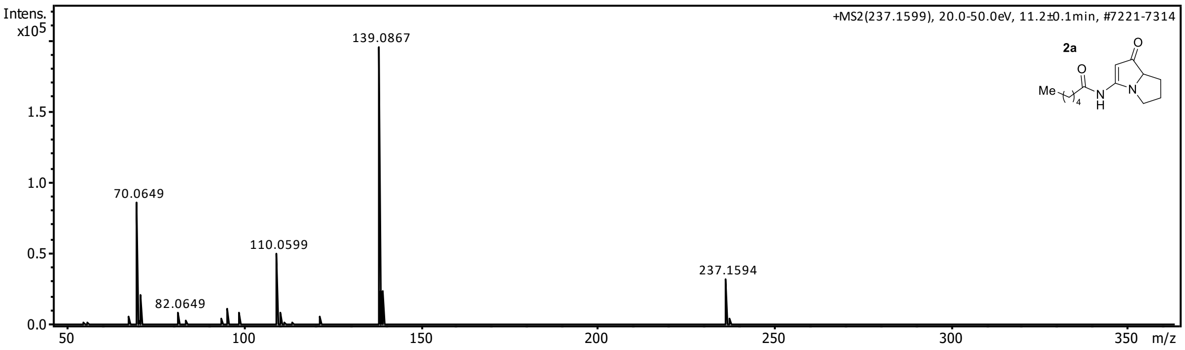

Figure S48. ESI+ CID MS/MS spectrum of C_6-pyrrolizixenamide-standard **2a** from HPLC.

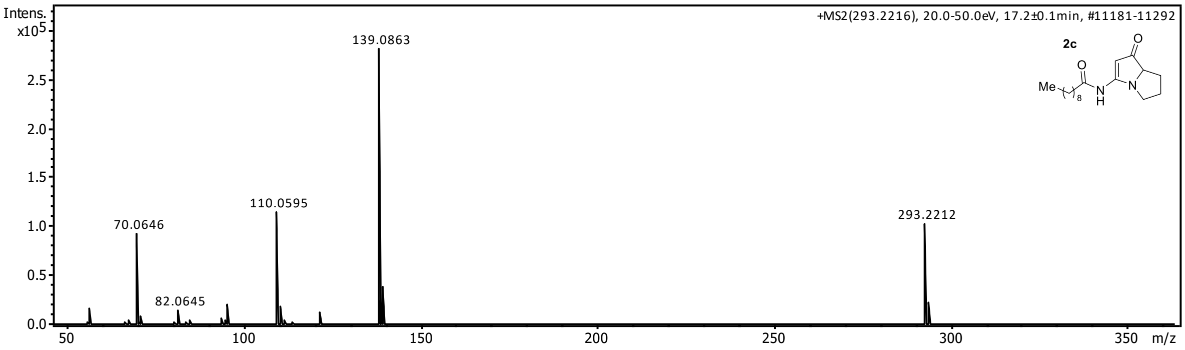

Figure S49. ESI+ CID MS/MS spectrum of C_{10}-pyrrolizixenamide-standard **2c** from HPLC.

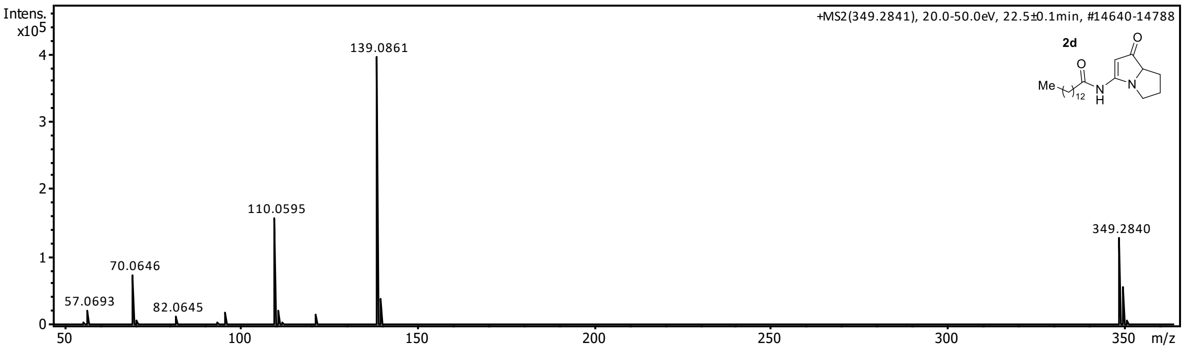

Figure S50. ESI+ CID MS/MS spectrum of C_{14}-pyrrolizixenamide-standard **2d** from HPLC.

5.3 Qualitative assay data

Typical assays were performed in a total volume of 200 µL, containing 300 µM substrate (10 mM in DMSO; 6 µL), 100 µM NADPH (20 µL), 50 µM FAD (10 µL), 30 µM of purified BVMO (10 mol-% corresponding to substrate; amount depending on enzyme concentration) and additional 7 vol-% DMSO (14 µL) in 50 mM TRIS HCl buffer (pH 7.5, 10 vol-% glycerol). For negative controls, buffer instead of enzyme was used. After incubation at 30 °C at 300 rpm for approx. 18 hours, assays were quenched by extraction with EtOAc (3 × 200 µL). Combined extracts were dried in a SpeedVac concentrator, and the residues dissolved in 120 µL MeOH before being analyzed by RP-LC-HRMS (injection volumes: negative control 5 µL; assay: 10 µL).

5.3.1 C_6-Substrate 3a

5.3.1.1 PxaB assay

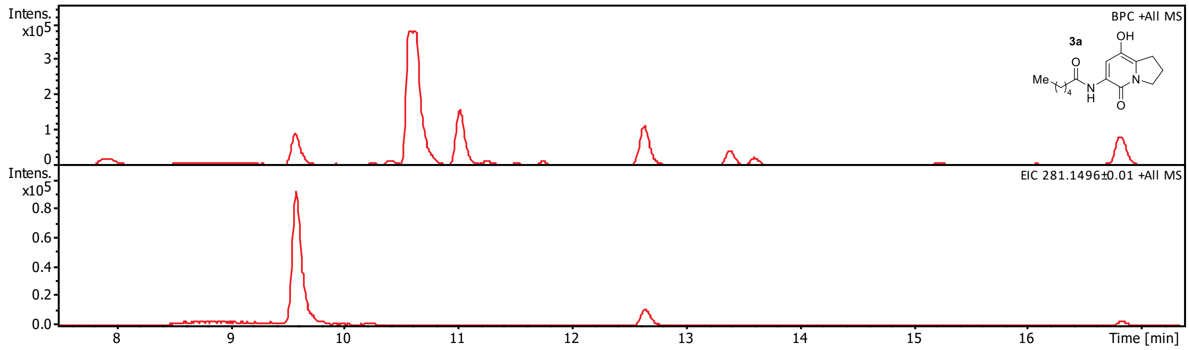

Figure S51. BPC (top) and EIC for oxidized degradation products (bottom) of negative control for substrate **3a**.

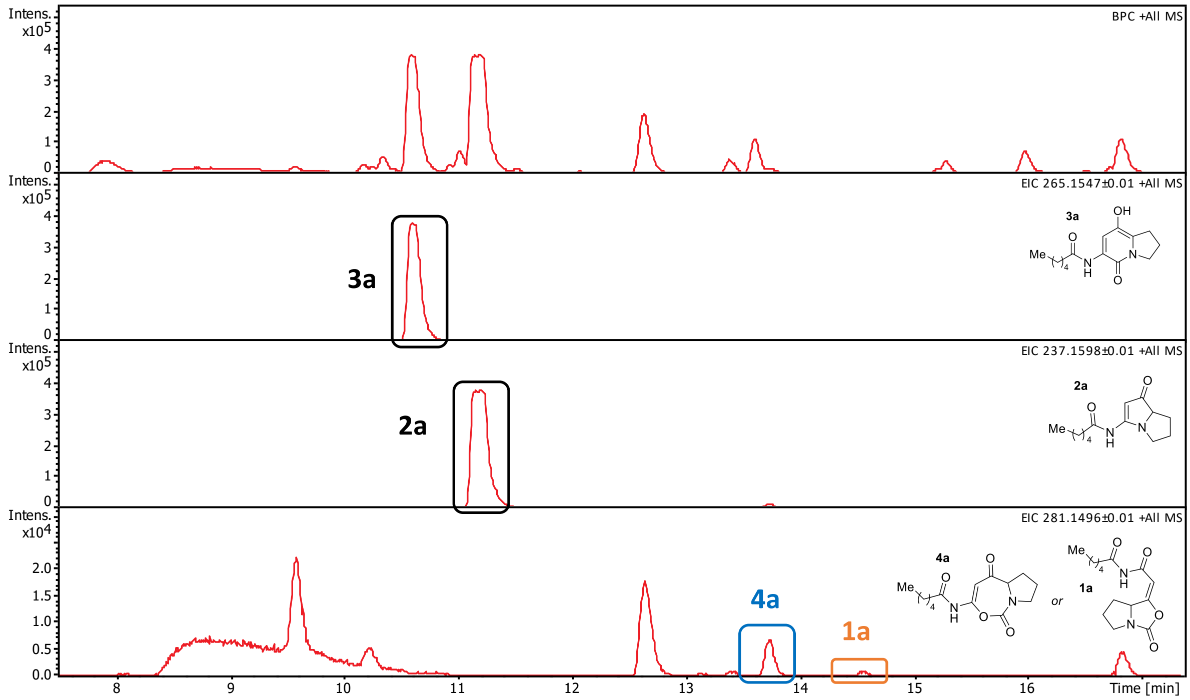

Figure S52. BPC (line one) and EICs for substrate **3a**, PA derivative **2a**, and oxidized products **4a** or **1a** (from line two to four) of the enzyme assay employing PxaB and substrate **3a**.

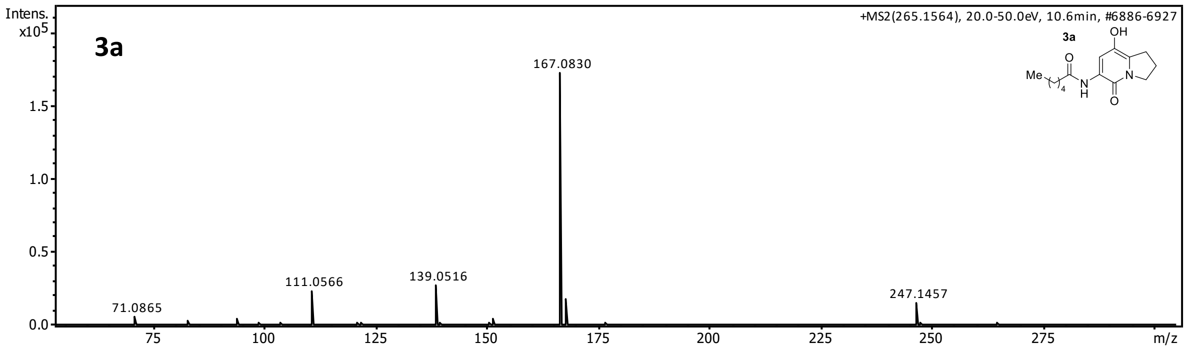

Figure S53. CID MS/MS spectrum from PxaB assay with substrate **3a**, retention time 10.6 minutes.

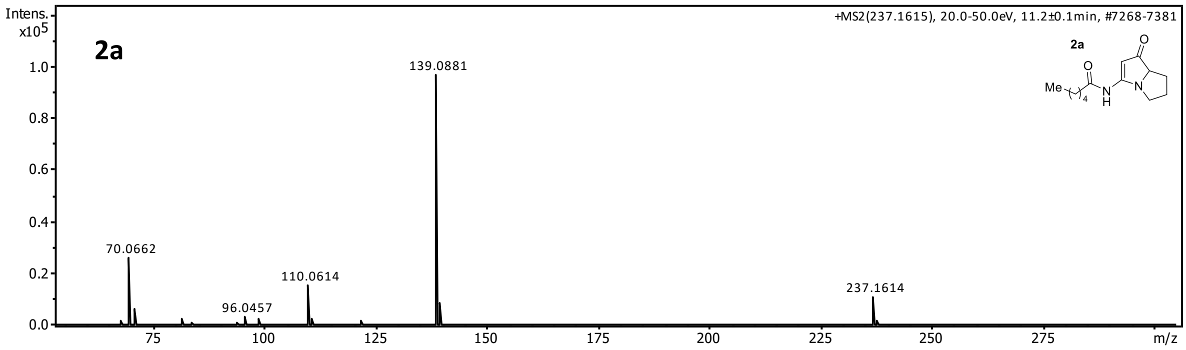

Figure S54. CID MS/MS spectrum from PxaB assay with substrate **3a**, retention time 11.2 minutes.

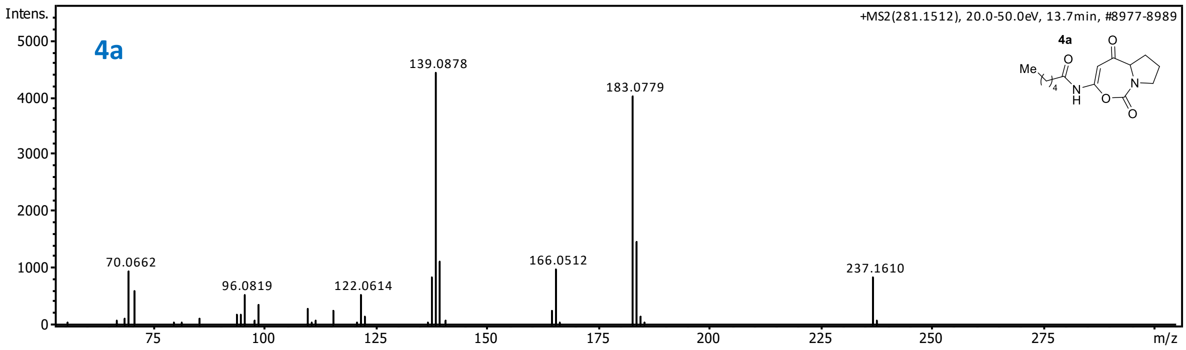

Figure S55. CID MS/MS spectrum from PxaB assay with substrate **3a**, retention time 13.7 minutes, thus identifying **4a**.

Peak 1a: Not enough counts for MS/MS spectrum.

5.3.1.2 BraC assay

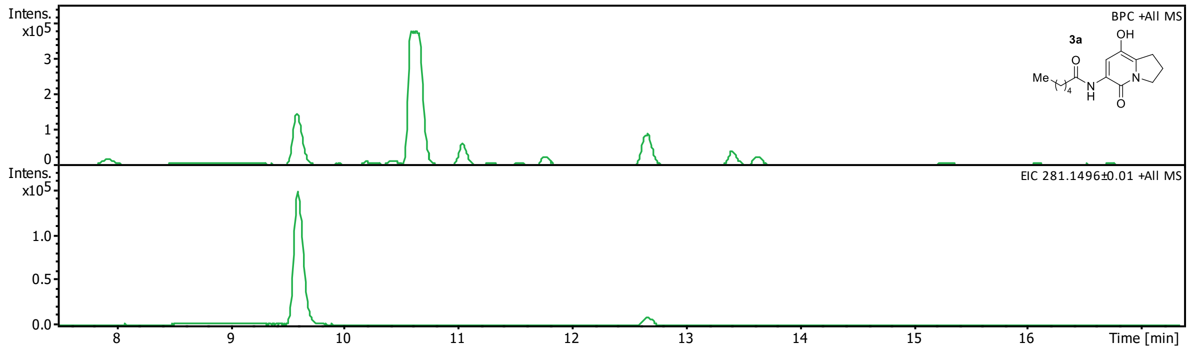

Figure S56. BPC (top) and EIC for oxidized degradation products (bottom) of negative control for substrate **3a**.

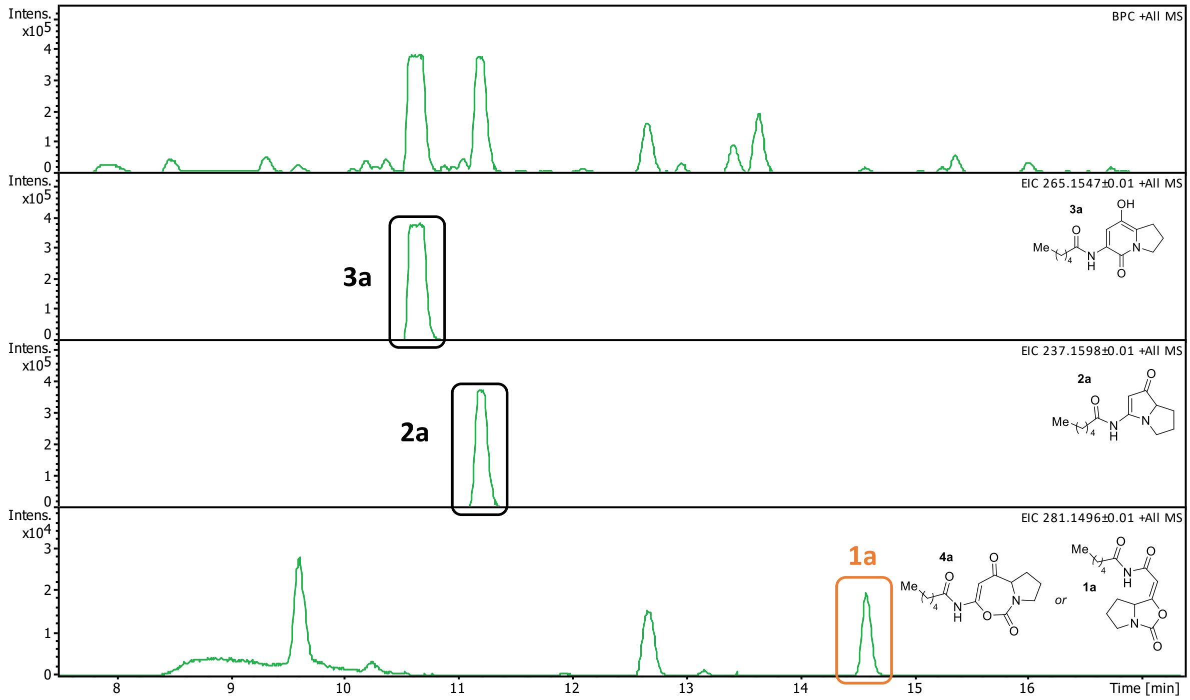

Figure S57. BPC (line one) and EICs for substrate **3a**, PA derivative **2a**, and oxidized products **4a** or **1a** (from line two to four) of the enzyme assay employing BraC and substrate **3a**.

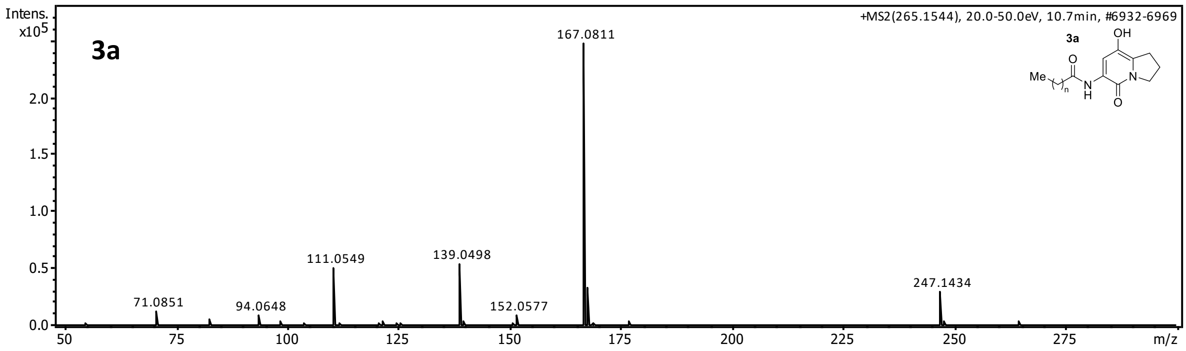

Figure S58. CID MS/MS spectrum from BraC assay with substrate **3a**, retention time 10.7 minutes.

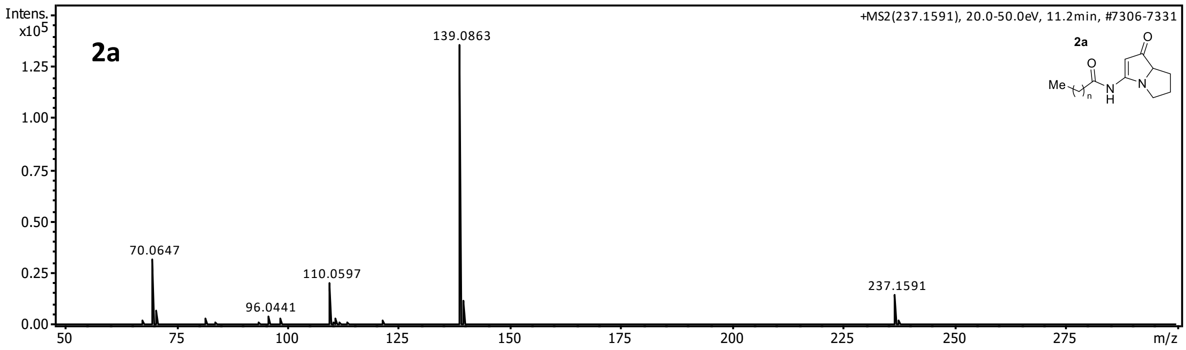

Figure S59. CID MS/MS spectrum from BraC assay with substrate **3a**, retention time 11.2 minutes.

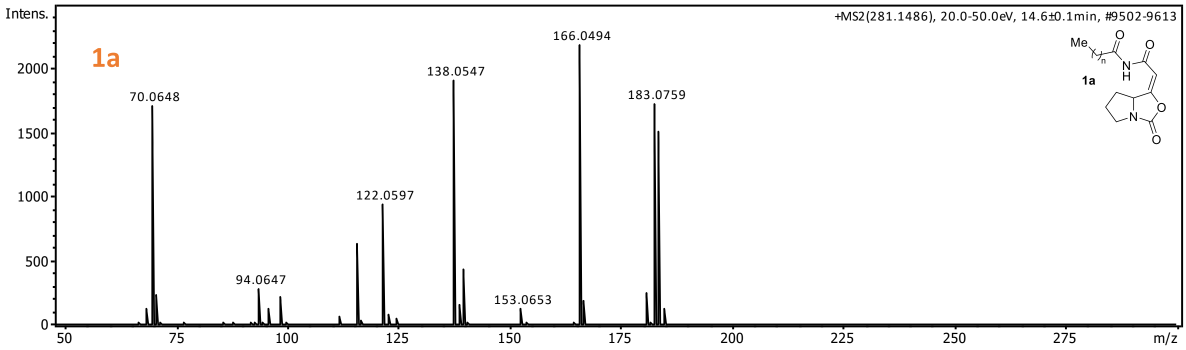

Figure S60. CID MS/MS spectrum from BraC assay with substrate **3a**, retention time 14.6 minutes, thus identifying **1a**.

5.3.2 C_8-Substrate 3b

5.3.2.1 PxaB assay

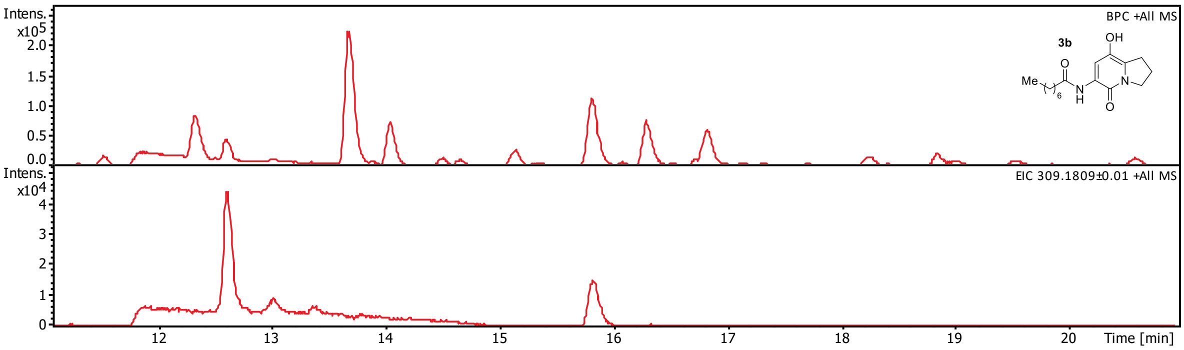

Figure S61. BPC (top) and EIC for oxidized degradation products (bottom) of negative control for substrate **3b**.

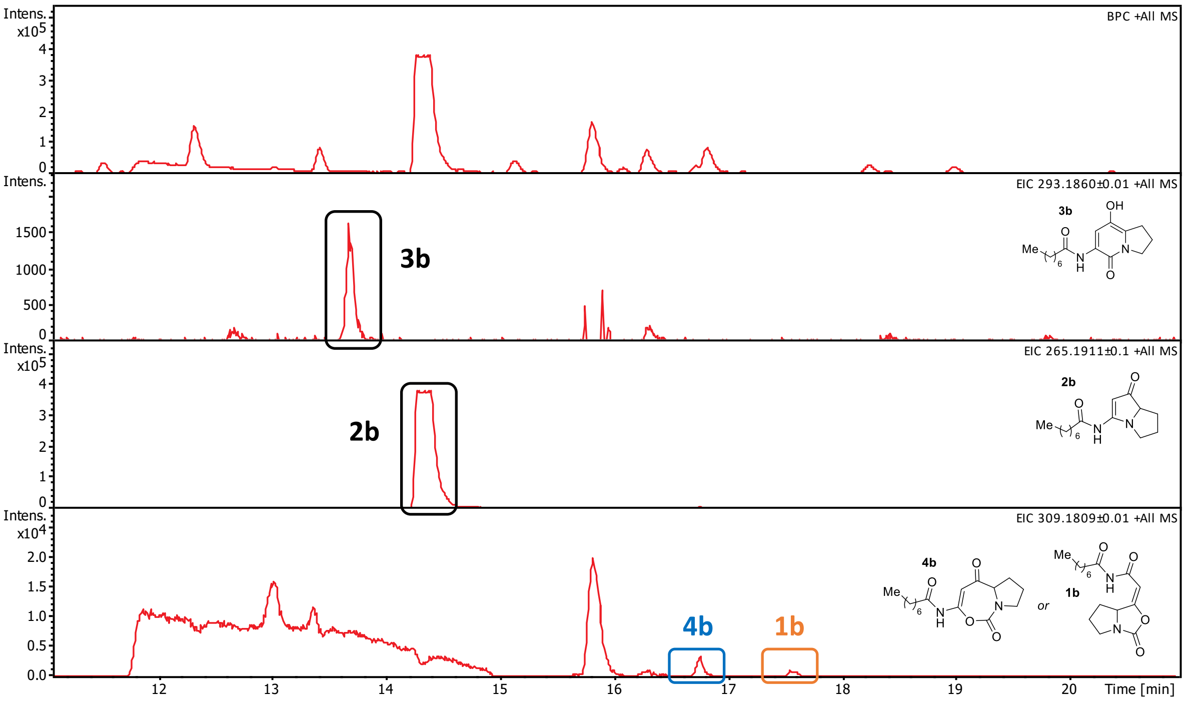

Figure S62. BPC (line one) and EICs for substrate **3b**, PA derivative **2b**, and oxidized products **4b** or **1b** (from line two to four) of the enzyme assay employing PxaB and substrate **3b**.

Peak **3b**: Not enough counts for MS/MS spectrum.

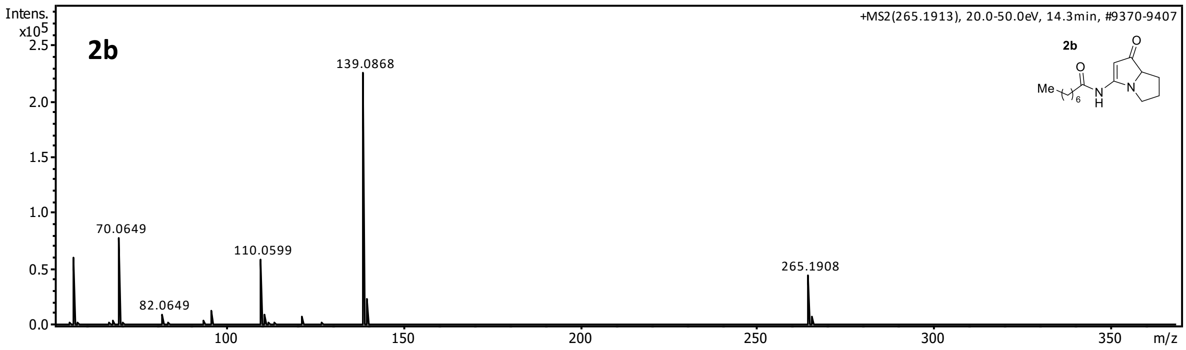

Figure S63. CID MS/MS spectrum from PxaB assay with substrate **3b**, retention time 14.3 minutes.

Peak **4b**: Not enough counts for MS/MS spectrum.

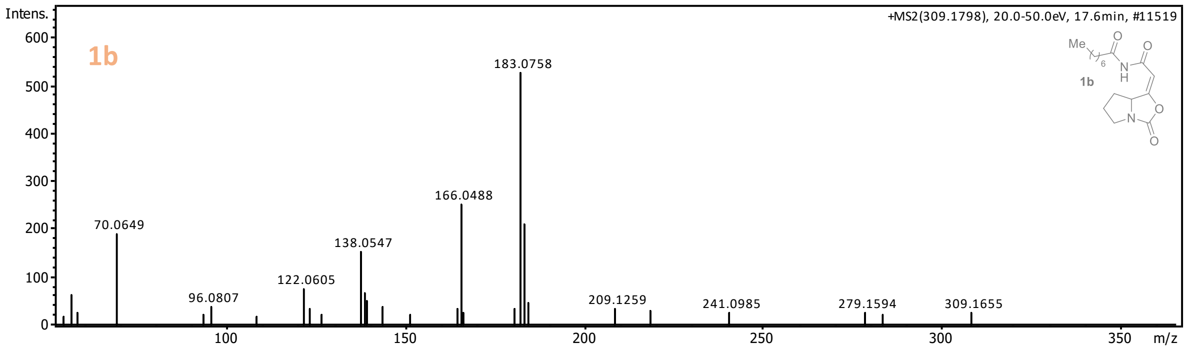

Figure S64. CID MS/MS spectrum from PxaB assay with substrate **3b**, retention time 17.6 minutes (low number of counts; presumably traces of **1b**).

5.3.2.2 BraC assay

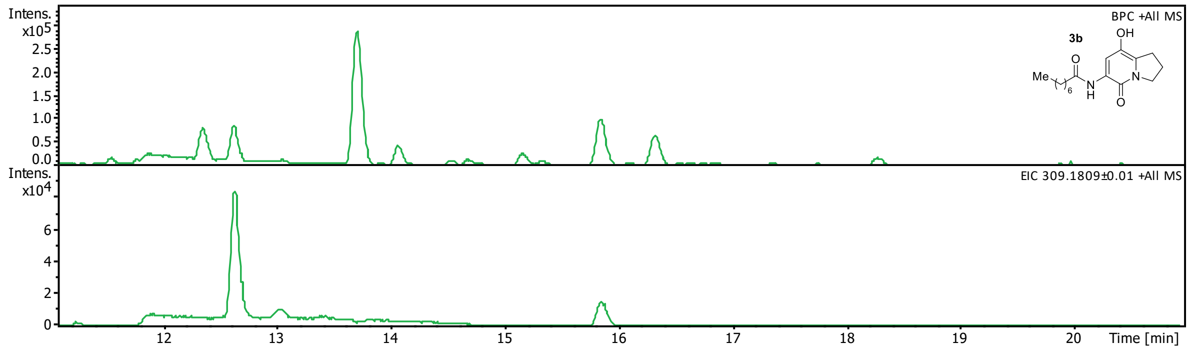

Figure S65. BPC (top) and EIC for oxidized degradation products (bottom) of negative control for substrate **3b**.

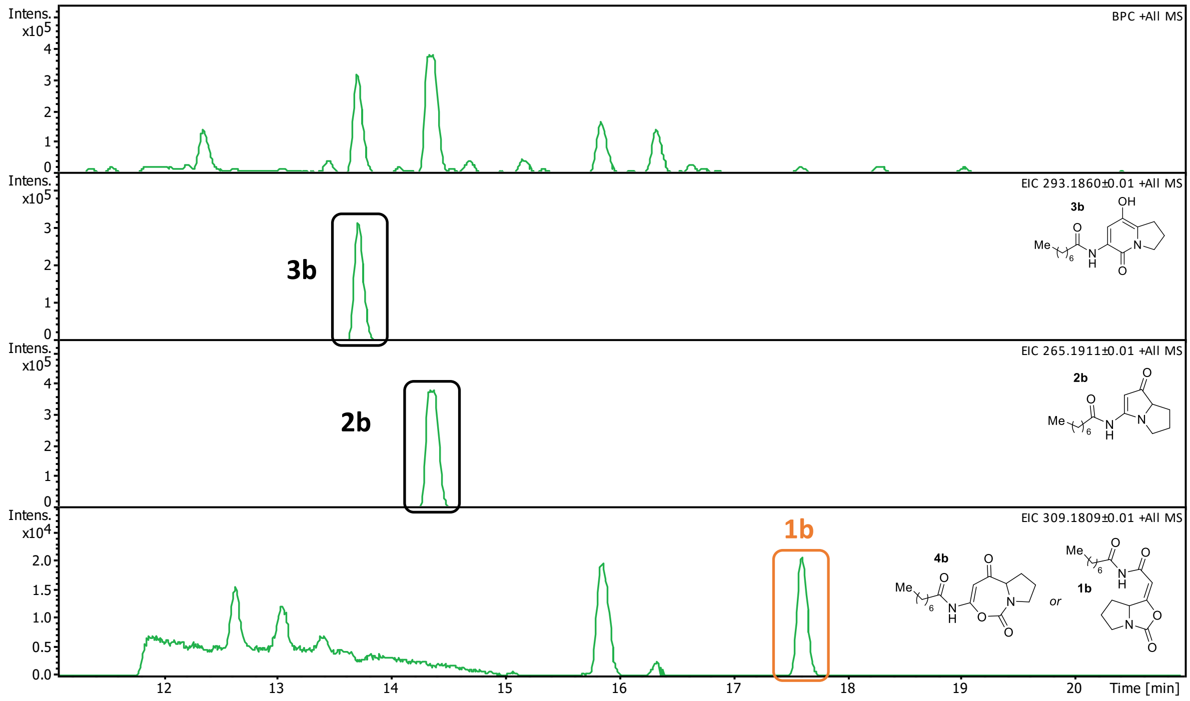

Figure S66. BPC (line one) and EICs for substrate **3b**, PA derivative **2b**, and oxidized products **4b** or **1b** (from line two to four) of the enzyme assay employing BraC and substrate **3b**.

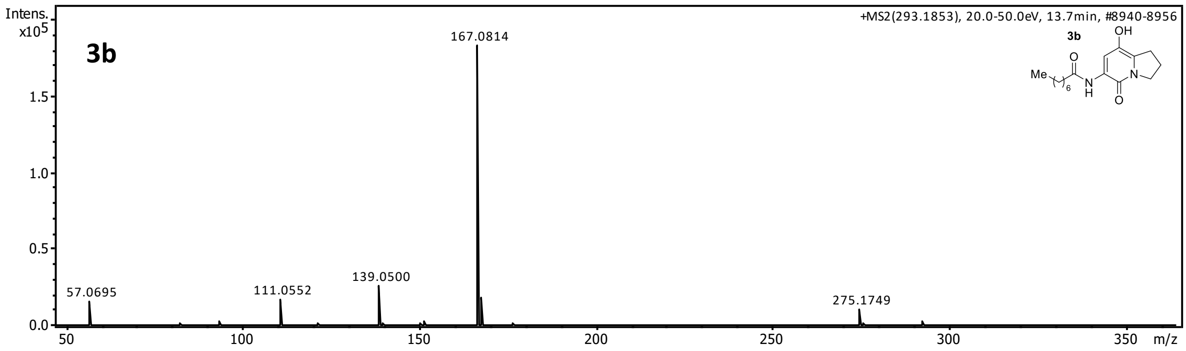

Figure S67. CID MS/MS spectrum from BraC assay with substrate **3b**, retention time 13.7 minutes.

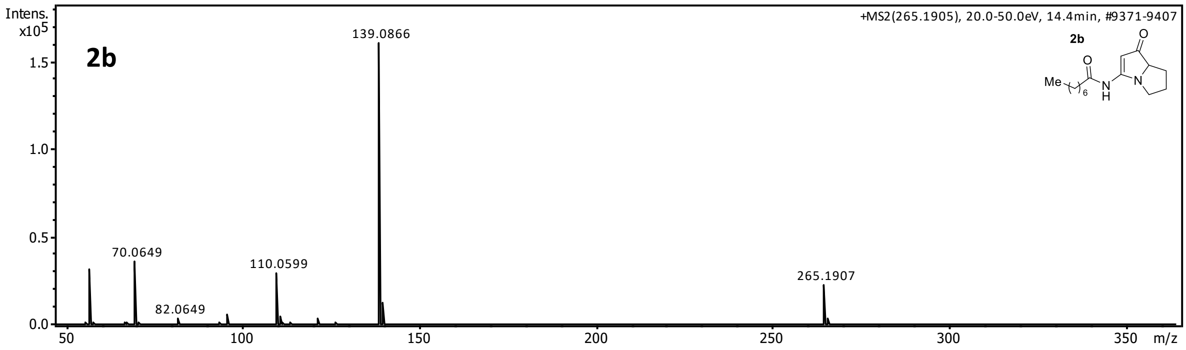

Figure S68. CID MS/MS spectrum from BraC assay with substrate **3b**, retention time 14.4 minutes.

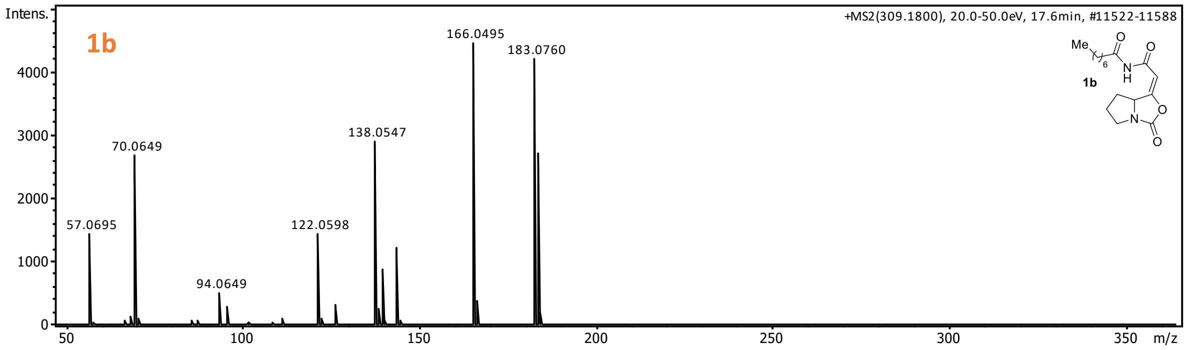

Figure S69. CID MS/MS spectrum from BraC assay with substrate **3b**, retention time 17.6 minutes, thus identifying **1b**.

5.3.3 C_{10}-Substrate 3c

5.3.3.1 PxaB assay

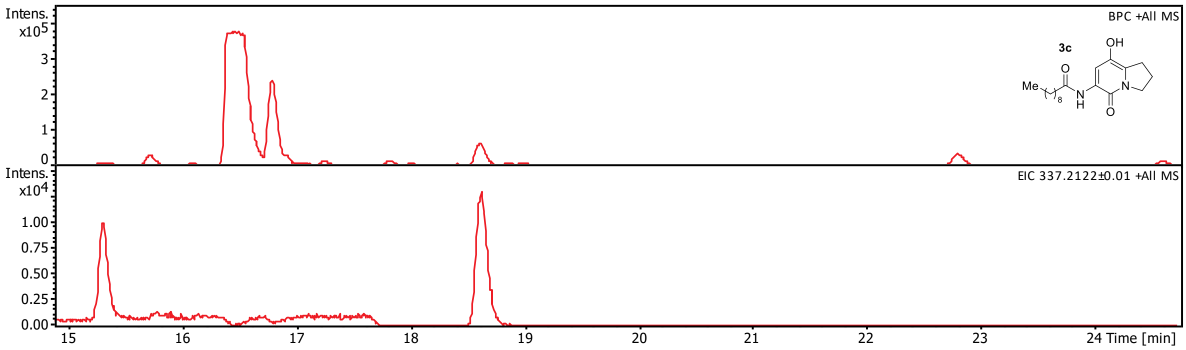

Figure S70. BPC (top) and EIC for oxidized degradation products (bottom) of negative control for substrate **3c**.

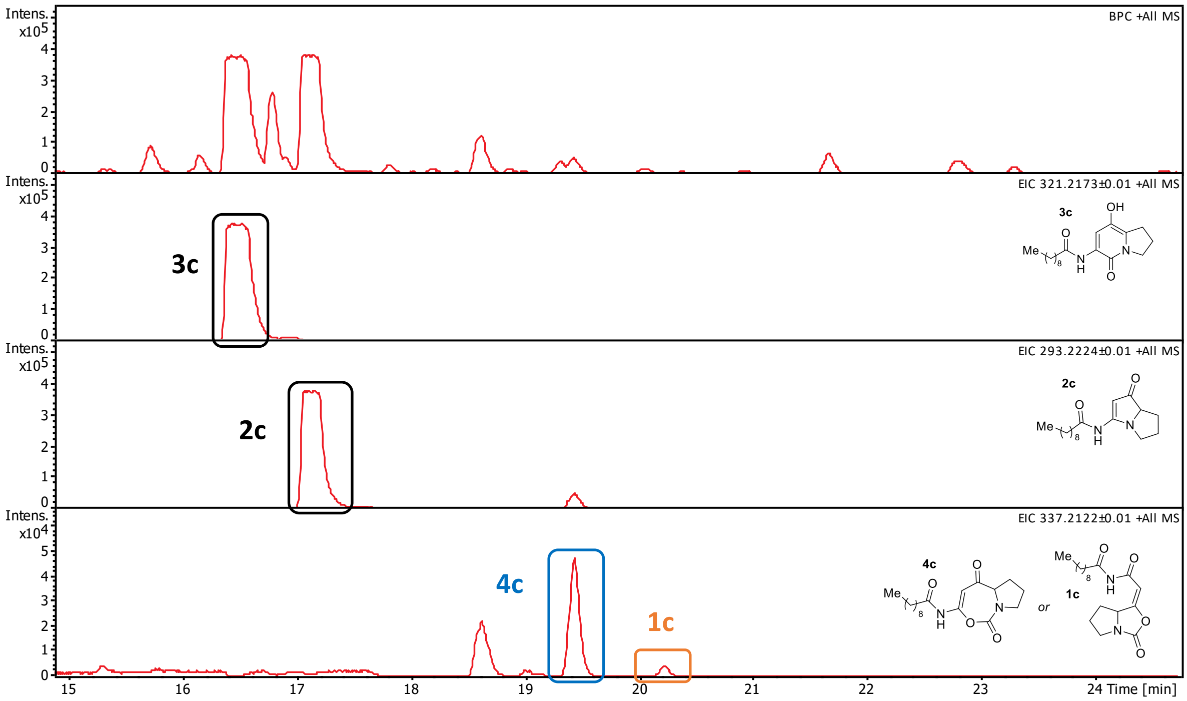

Figure S71. BPC (line one) and EICs for substrate **3c**, PA derivative **2c**, and oxidized products **4c** or **1c** (from line two to four) of the enzyme assay employing PxaB and substrate **3c**.

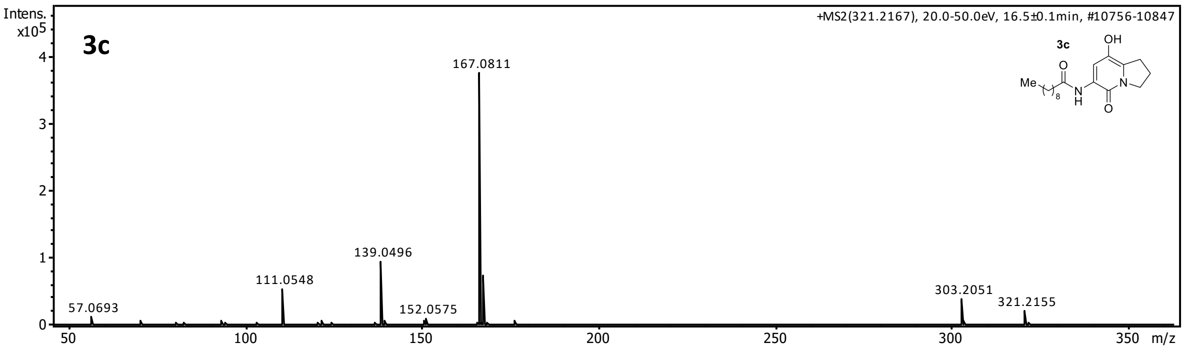

Figure S72. CID MS/MS spectrum from PxaB assay with substrate **3c**, retention time 16.5 minutes.

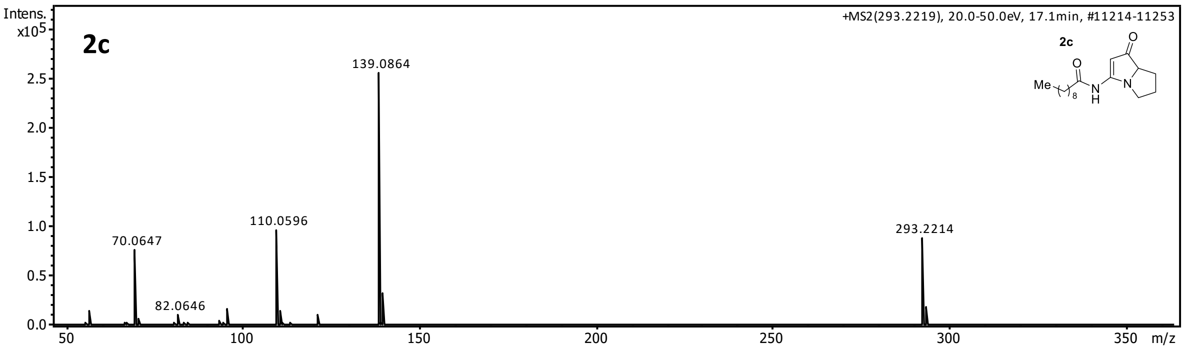

Figure S73. CID MS/MS spectrum from PxaB assay with substrate **3c**, retention time 17.1 minutes.

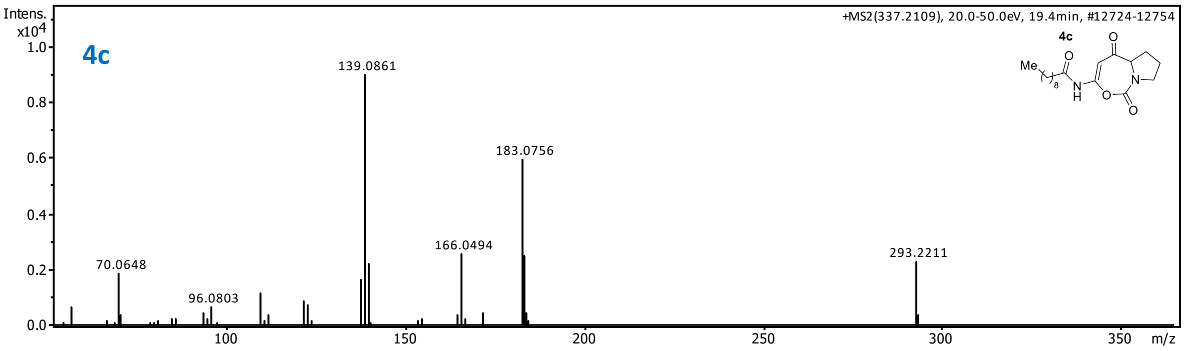

Figure S74. CID MS/MS spectrum from PxaB assay with substrate **3c**, retention time 19.4 minutes, thus identifying **4c**.

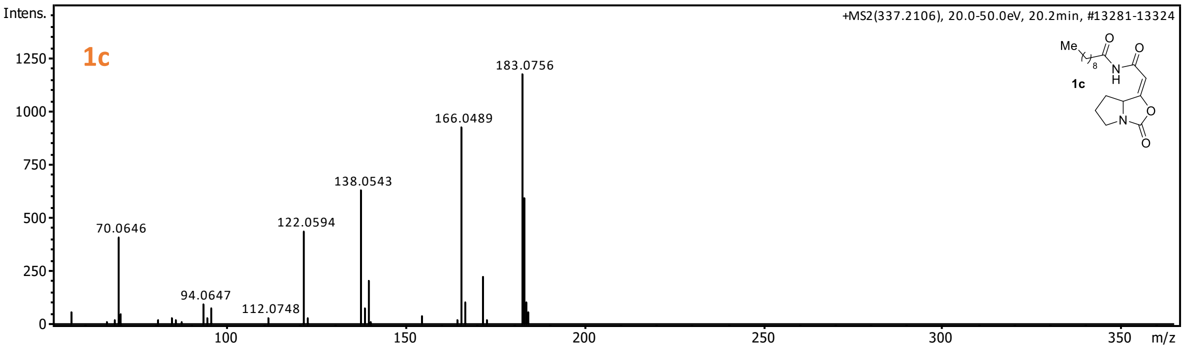

Figure S75. CID MS/MS spectrum from PxaB assay with substrate **3c**, retention time 20.2 minutes, thus identifying **1c**.

5.3.3.2 BraC assay

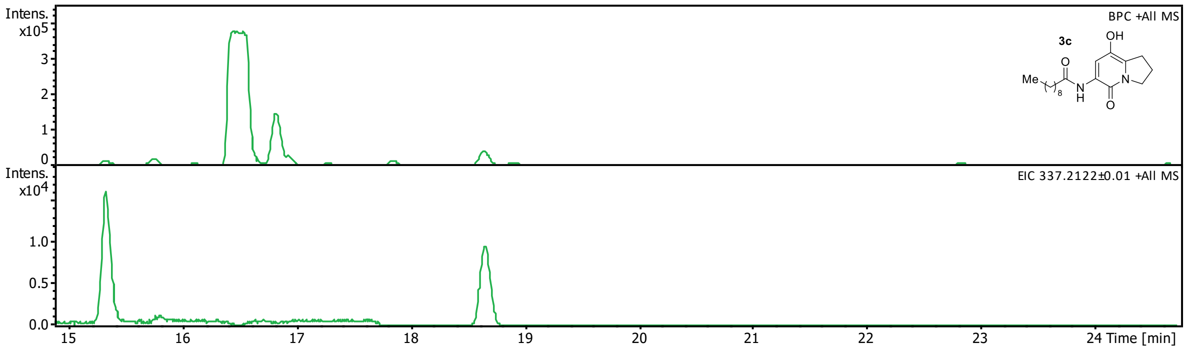

Figure S76. BPC (top) and EIC for oxidized degradation products (bottom) of negative control for substrate **3c**.

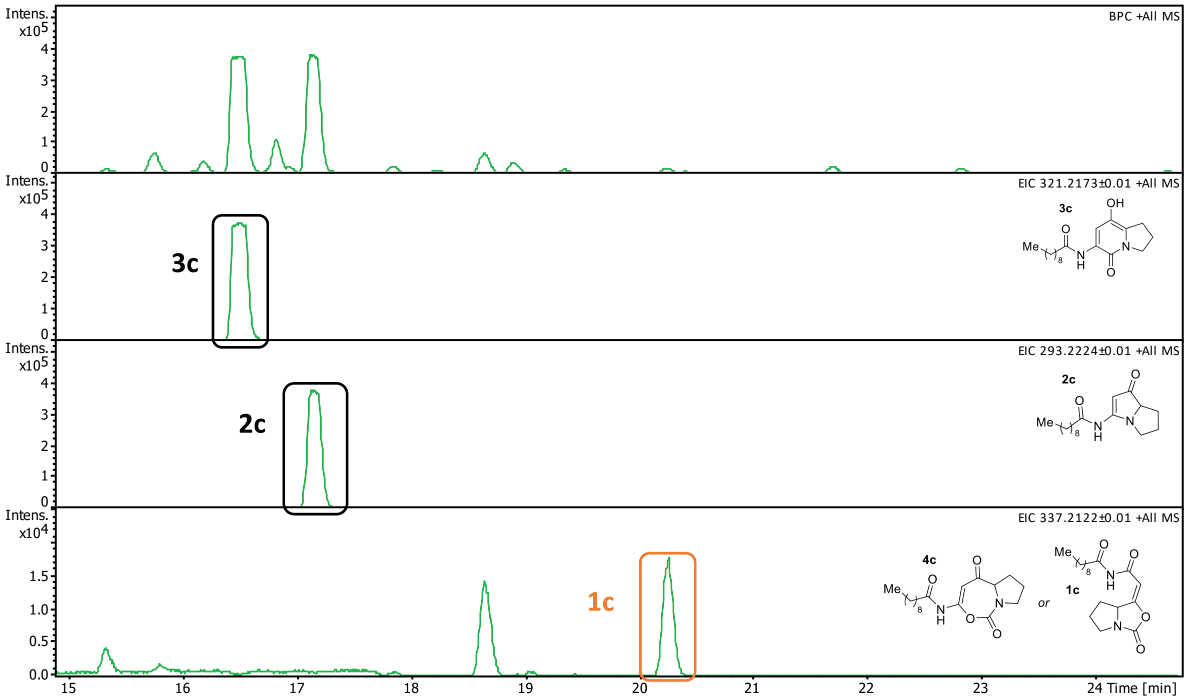

Figure S77. BPC (line one) and EICs for substrate **3c**, PA derivative **2c**, and oxidized products **4c** or **1c** (from line two to four) of the enzyme assay employing BraC and substrate **3c**.

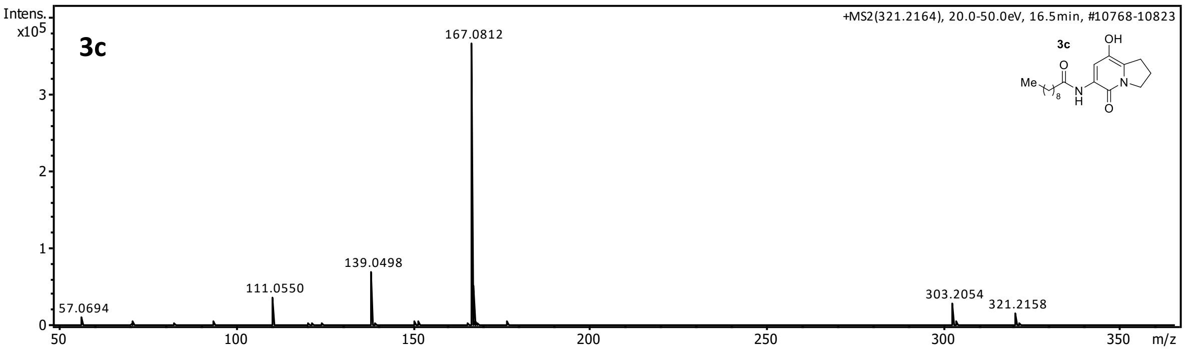

Figure S78. CID MS/MS spectrum from BraC assay with substrate **3c**, retention time 16.5 minutes.

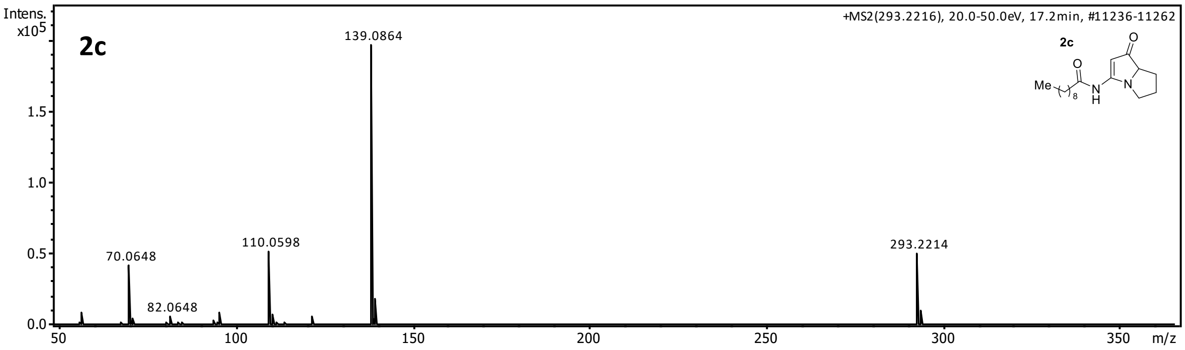

Figure S79. CID MS/MS spectrum from BraC assay with substrate **3c**, retention time 17.2 minutes.

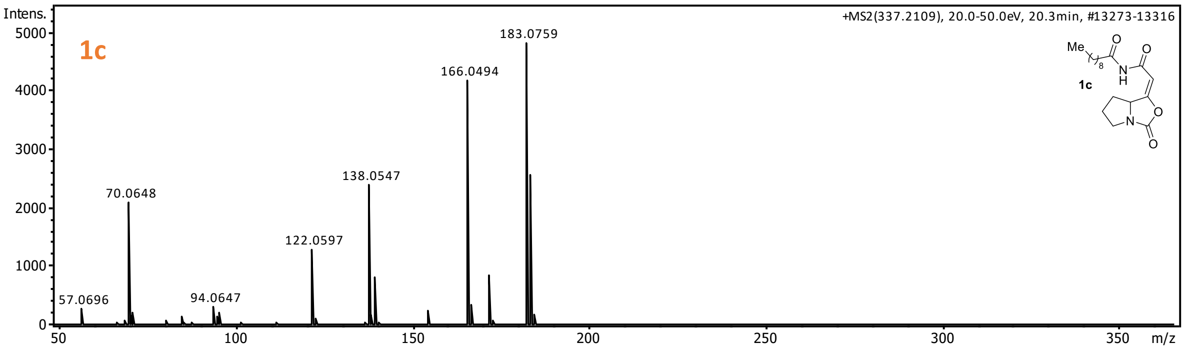

Figure S80. CID MS/MS spectrum from BraC assay with substrate **3c**, retention time 20.3 minutes, thus identifying **1c**.

5.3.4 C_{14}-Substrate 3d

5.3.4.1 PxaB assay

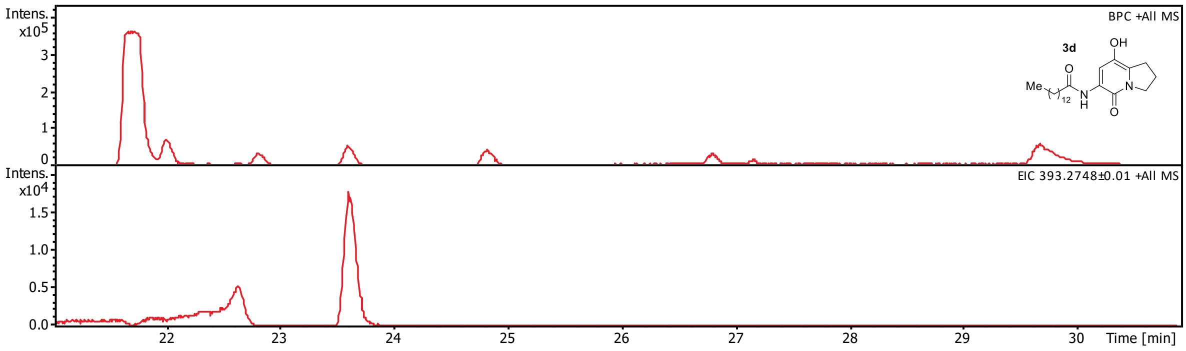

Figure S81. BPC (top) and EIC for oxidized degradation products (bottom) of negative control for substrate **3d**.

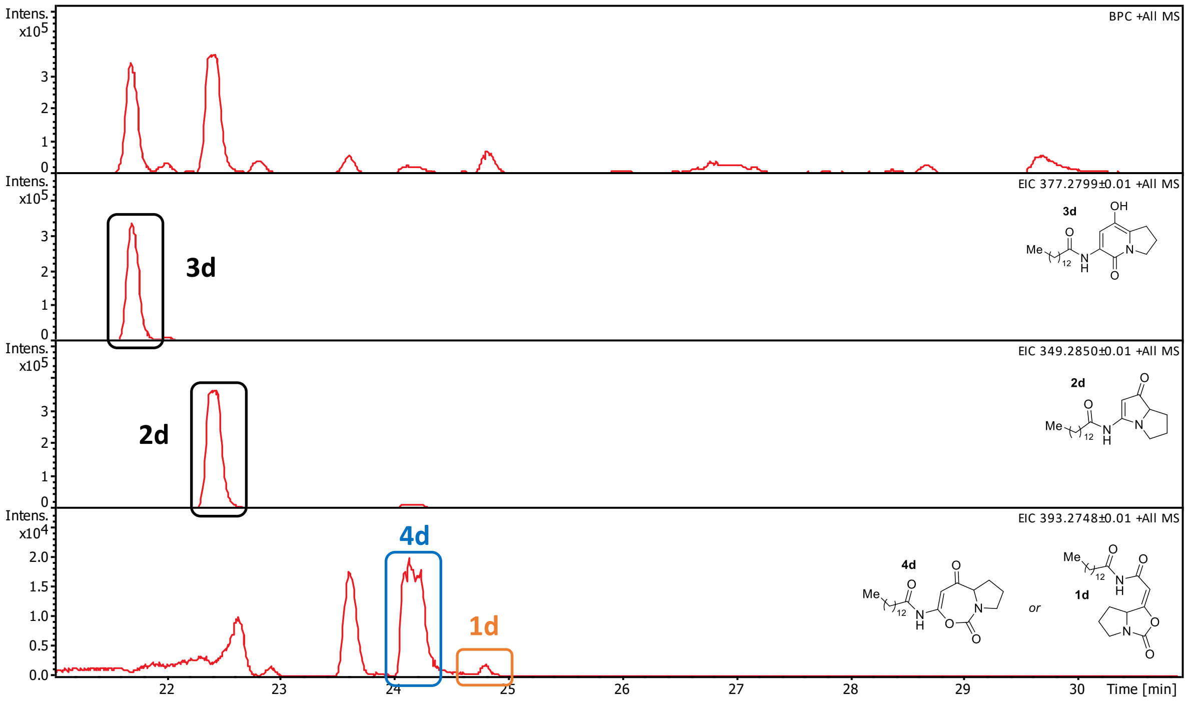

Figure S82. BPC (line one) and EICs for substrate **3d**, PA derivative **2d**, and oxidized products **4d** or **1d** (from line two to four) of the enzyme assay employing PxaB and substrate **3d**.

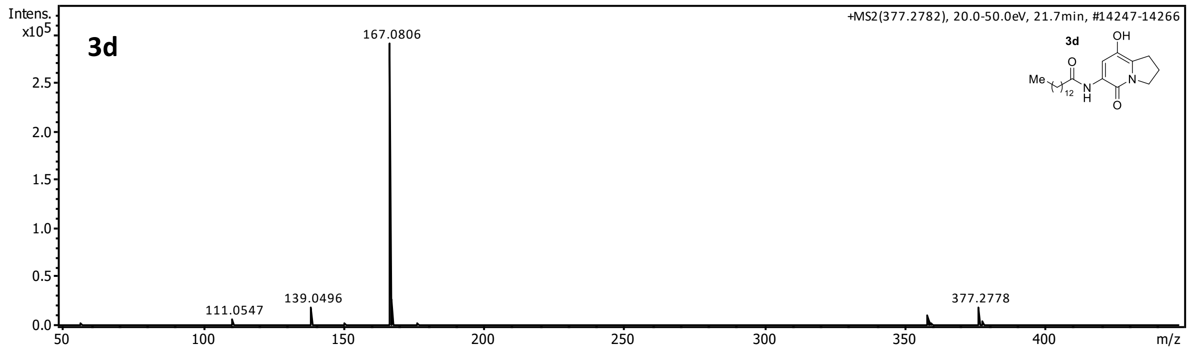

Figure S83. CID MS/MS spectrum from PxaB assay with substrate **3d**, retention time 21.7 minutes.

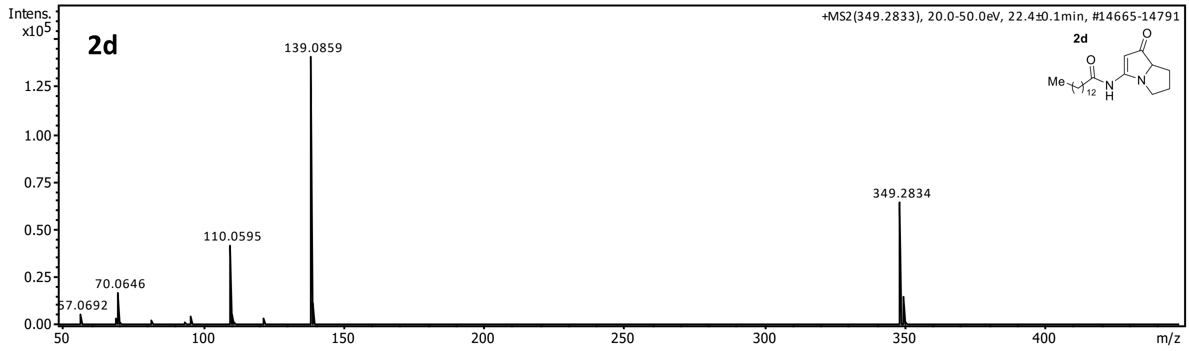

Figure S84. CID MS/MS spectrum from PxaB assay with substrate **3d**, retention time 22.4 minutes.

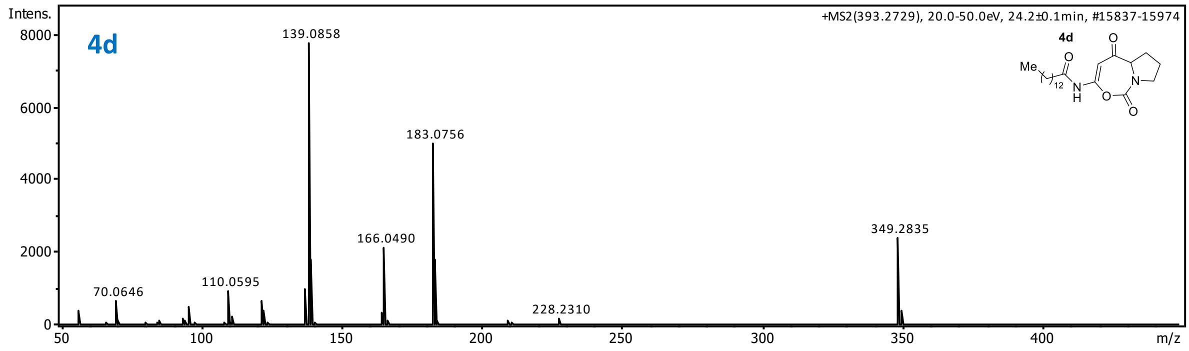

Figure S85. CID MS/MS spectrum from PxaB assay with substrate **3d**, retention time 24.2 minutes, thus identifying **4d**.

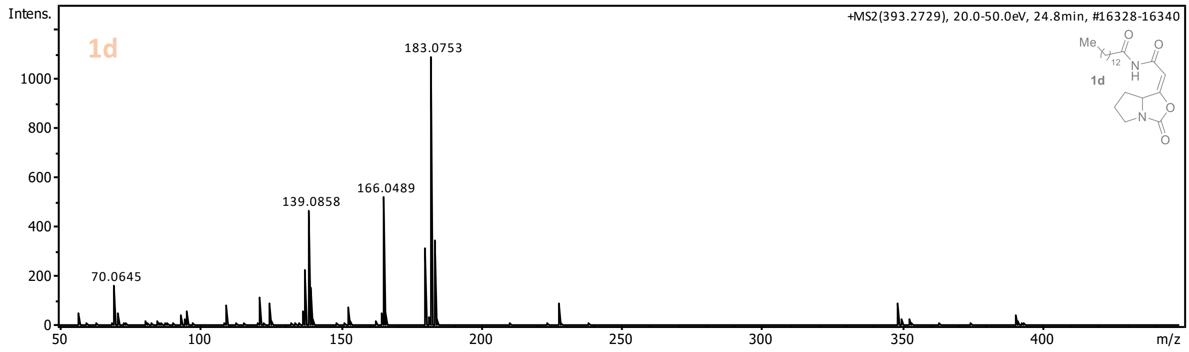

Figure S86. CID MS/MS spectrum from PxaB assay with substrate **3d**, retention time 24.8 minutes (low number of counts, peak not completely resolved; presumably traces of **1d**).

5.3.4.2 BraC assay

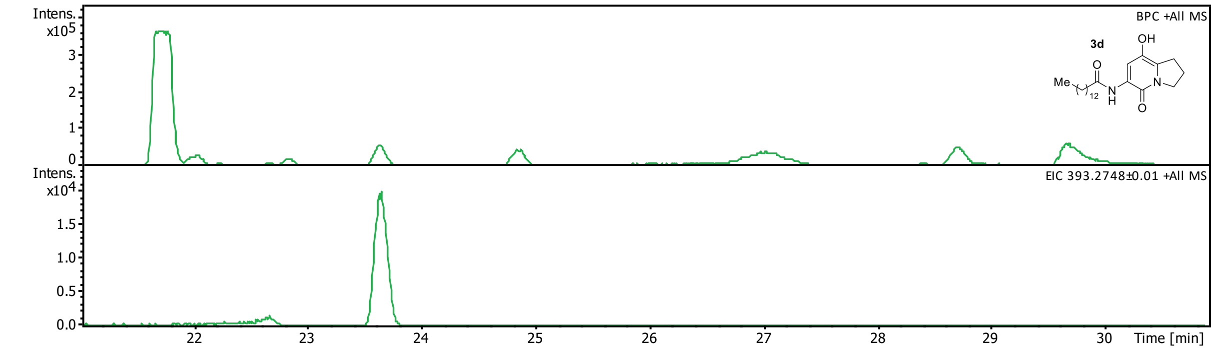

Figure S87. BPC (top) and EIC for oxidized degradation products (bottom) of negative control for substrate **3d**.

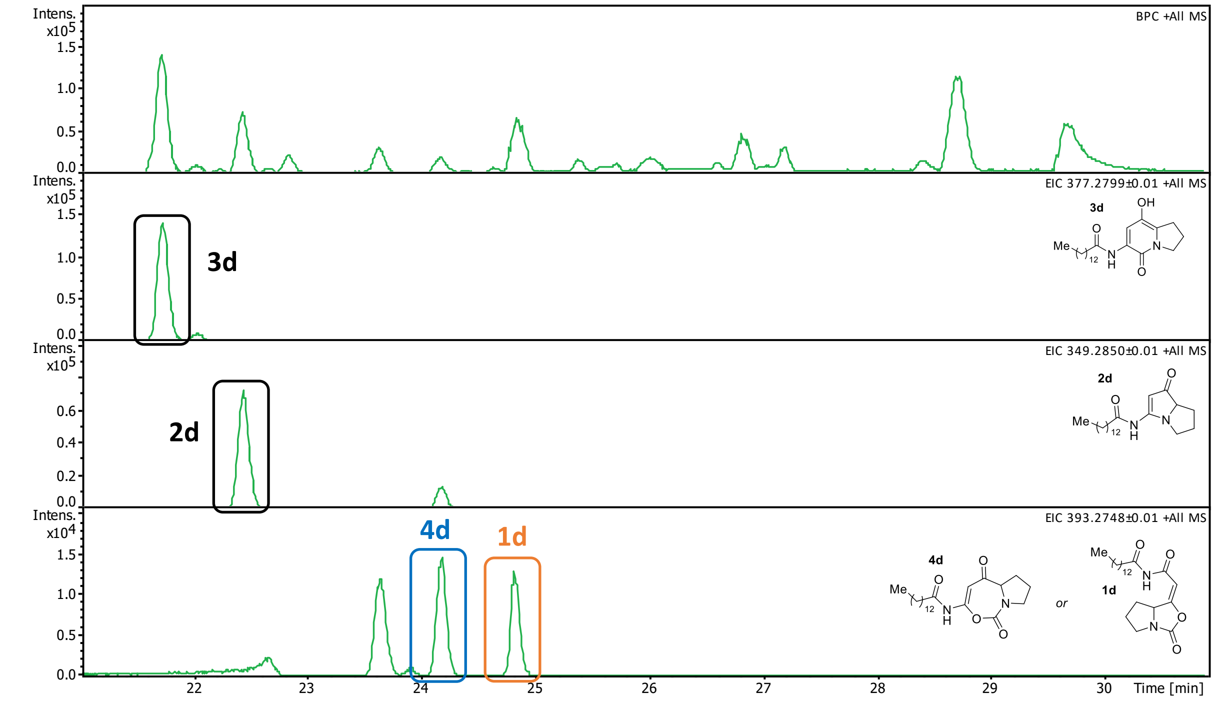

Figure S88. BPC (line one) and EICs for substrate **3d**, PA derivative **2d**, and oxidized products **4d** or **1d** (from line two to four) of the enzyme assay employing BraC and substrate **3d**.

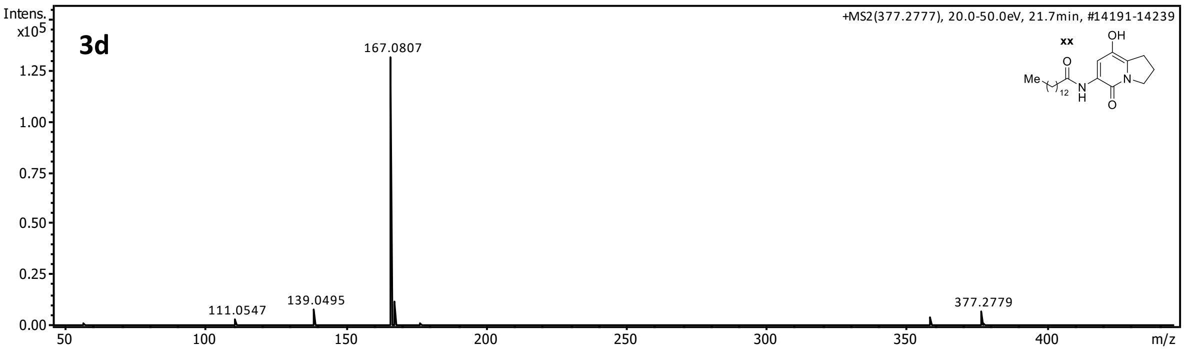

Figure S89. CID MS/MS spectrum from BraC assay with substrate **3d**, retention time 21.7 minutes.

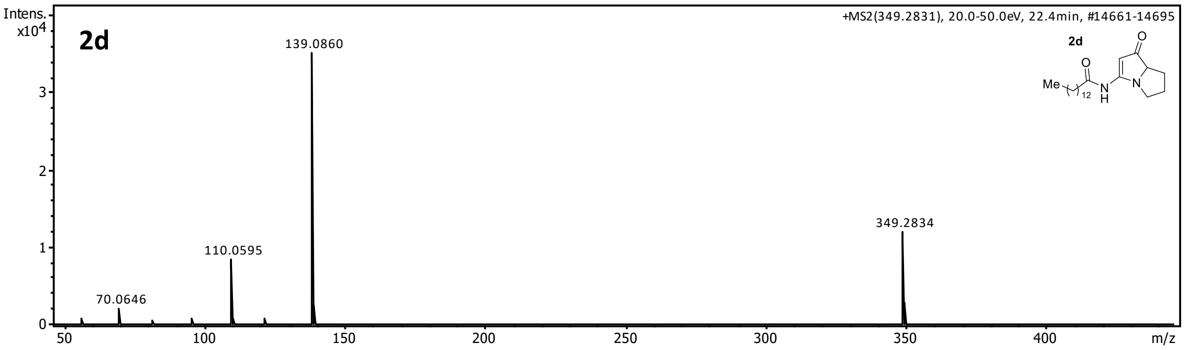

Figure S90. CID MS/MS spectrum from BraC assay with substrate **3d**, retention time 22.4 minutes.

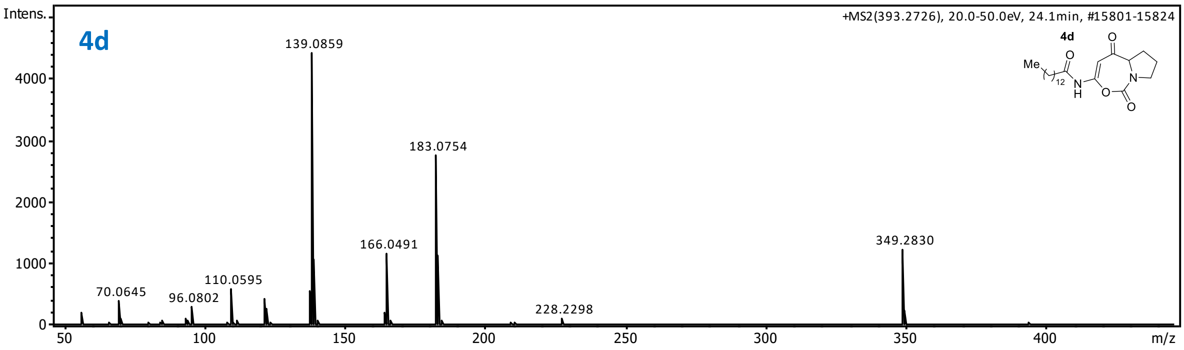

Figure S91. CID MS/MS spectrum from BraC assay with substrate **3d**, retention time 24.1 minutes, thus identifying **4d**.

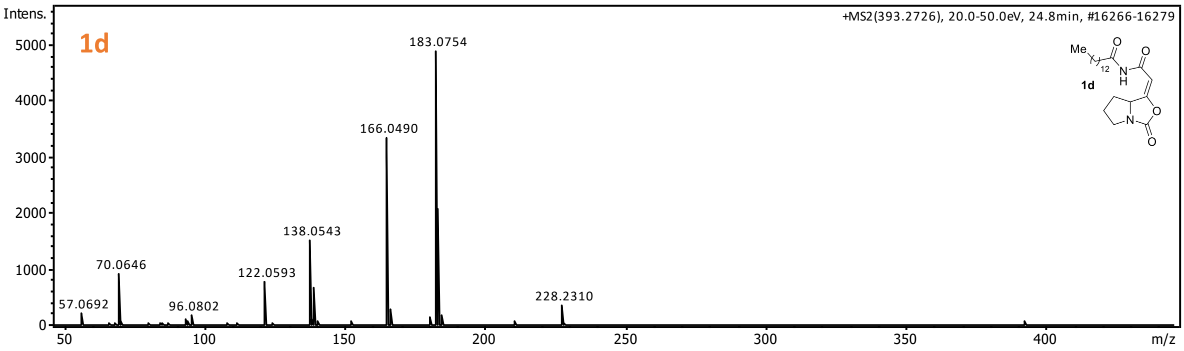

Figure S92. CID MS/MS spectrum from BraC assay with substrate **3d**, retention time 24.8 minutes, thus identifying **1d**.

5.3.5 C_{14}OH-Substrate 3e

5.3.5.1 PxaB assay

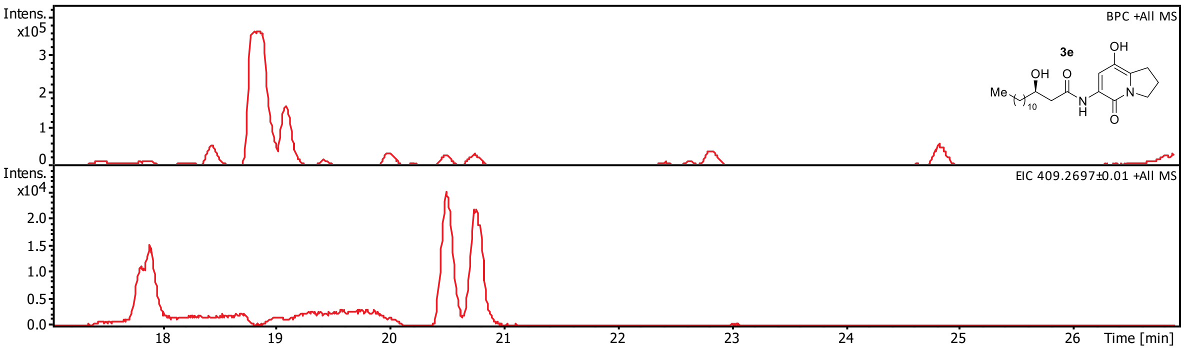

Figure S93. BPC (top) and EIC for oxidized degradation products (bottom) of negative control for substrate **3e**.

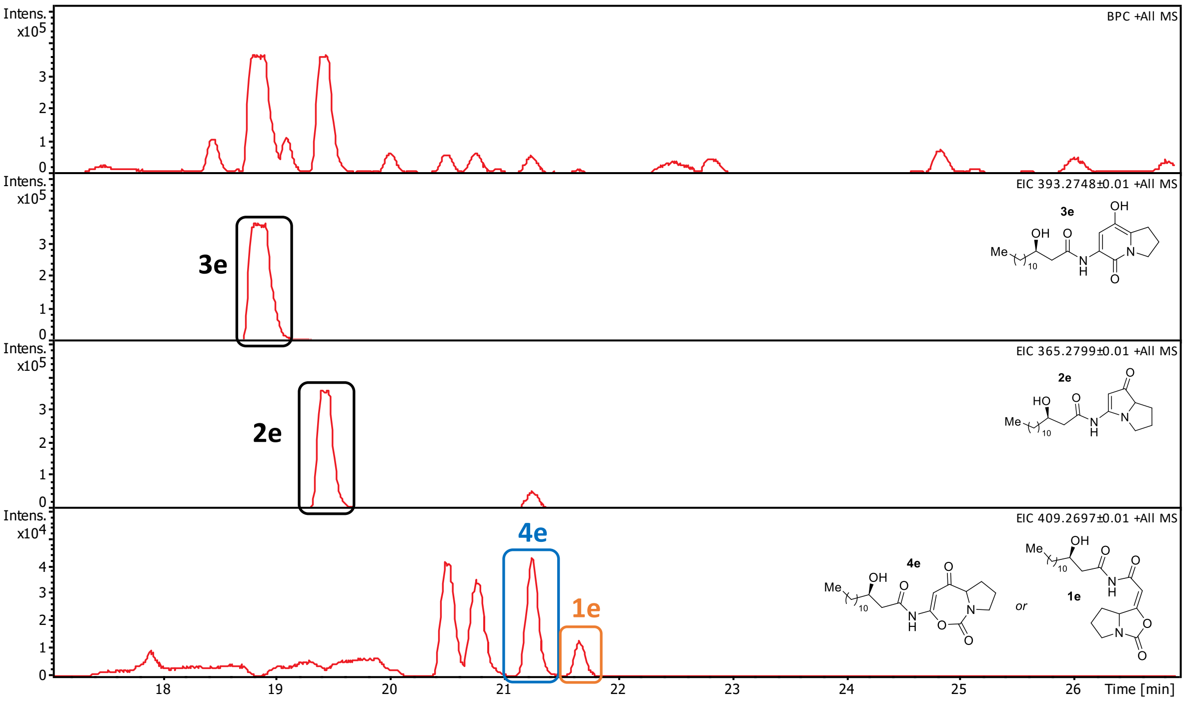

Figure S94. BPC (line one) and EICs for substrate **3e**, PA derivative **2e**, and oxidized products **4e** or **1e** (from line two to four) of the enzyme assay employing PxaB and substrate **3e**.

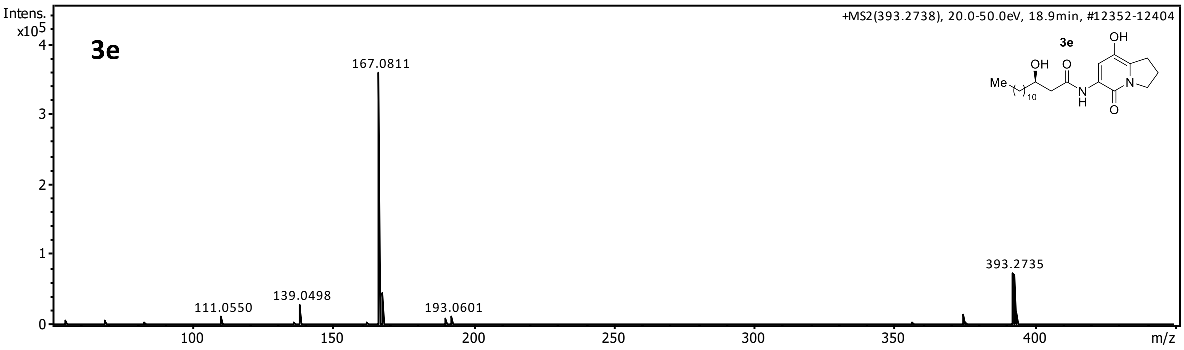

Figure S95. CID MS/MS spectrum from PxaB assay with substrate **3e**, retention time 18.9 minutes.

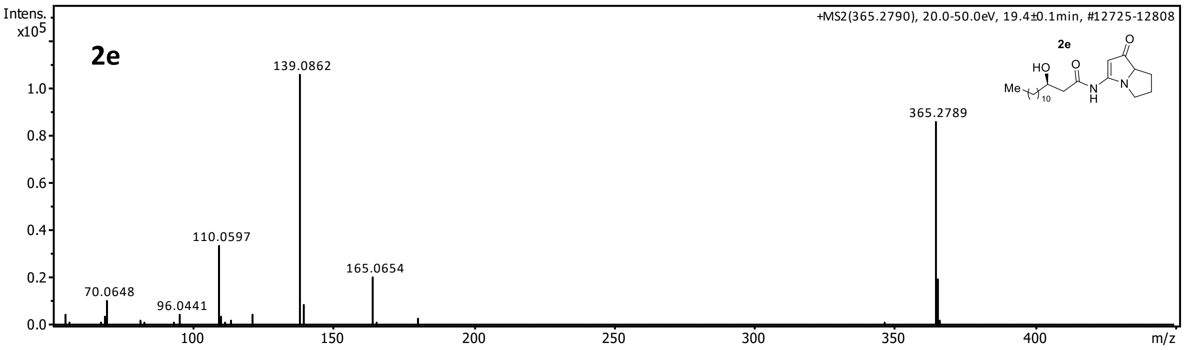

Figure S96. CID MS/MS spectrum from PxaB assay with substrate **3e**, retention time 19.4 minutes.

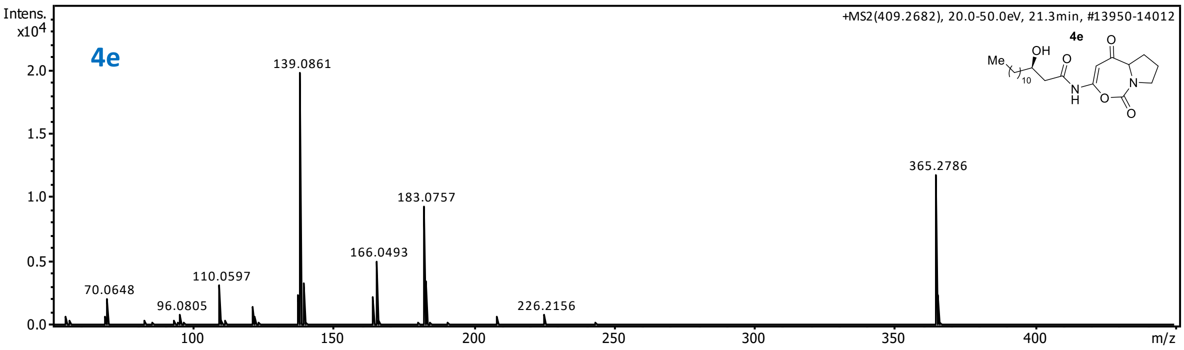

Figure S97. CID MS/MS spectrum from PxaB assay with substrate **3e**, retention time 21.3 minutes, thus identifying **4e**.

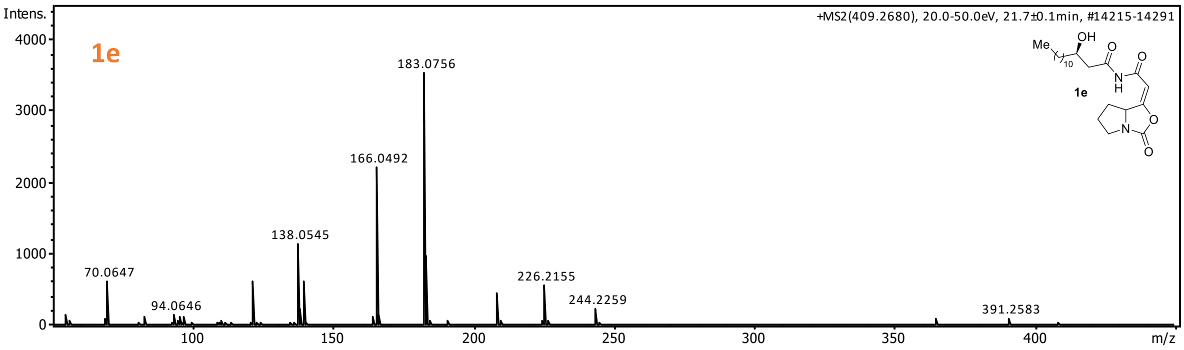

Figure S98. CID MS/MS spectrum from PxaB assay with substrate **3e**, retention time 21.7 minutes, thus identifying **1e**.

5.3.5.2 BraC assay

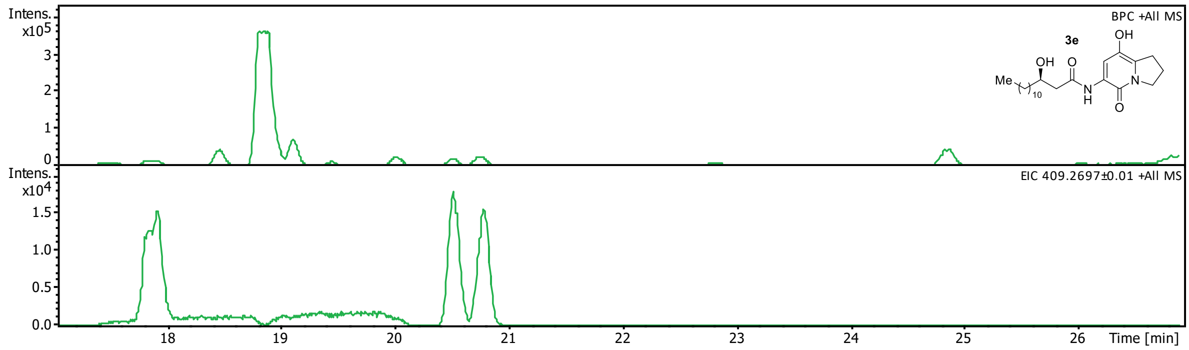

Figure S99. BPC (top) and EIC for oxidized degradation products (bottom) of negative control for substrate **3e**.

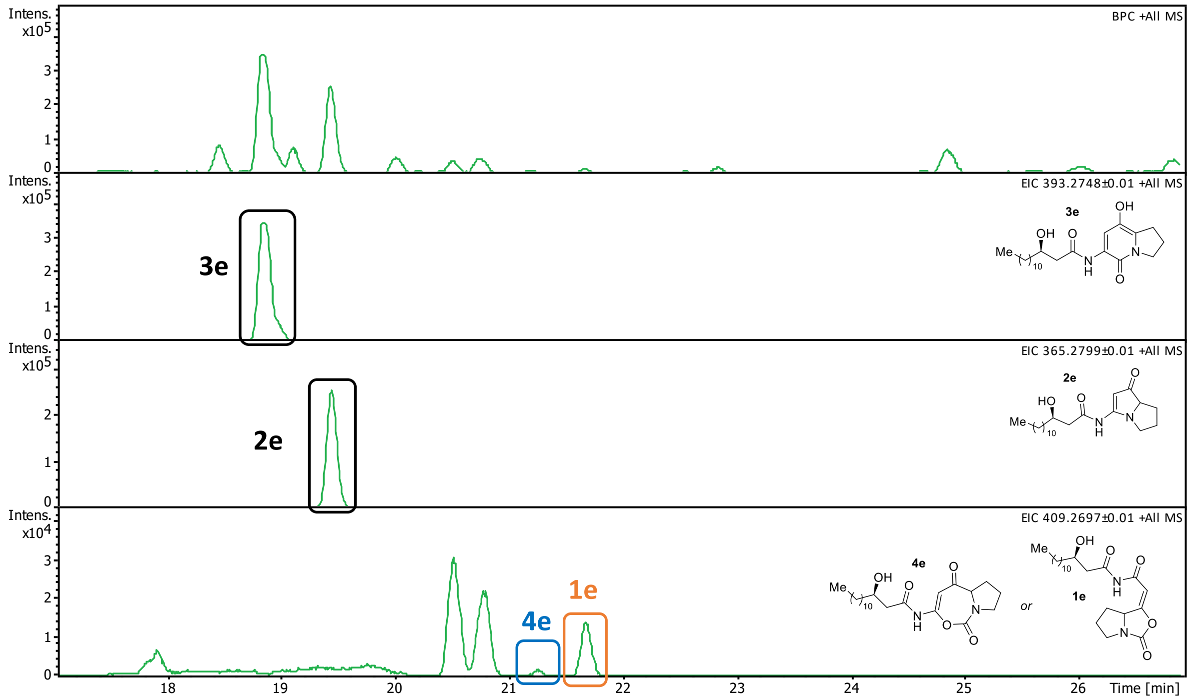

Figure S100. BPC (line one) and EICs for substrate **3e**, PA derivative **2e**, and oxidized products **4e** or **1e** (from line two to four) of the enzyme assay employing BraC and substrate **3e**.

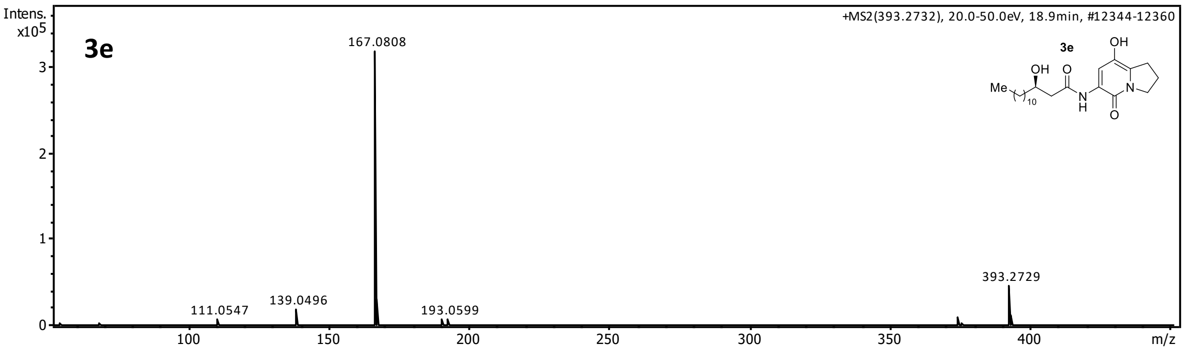

Figure S101. CID MS/MS spectrum from BraC assay with substrate **3e**, retention time 18.9 minutes.

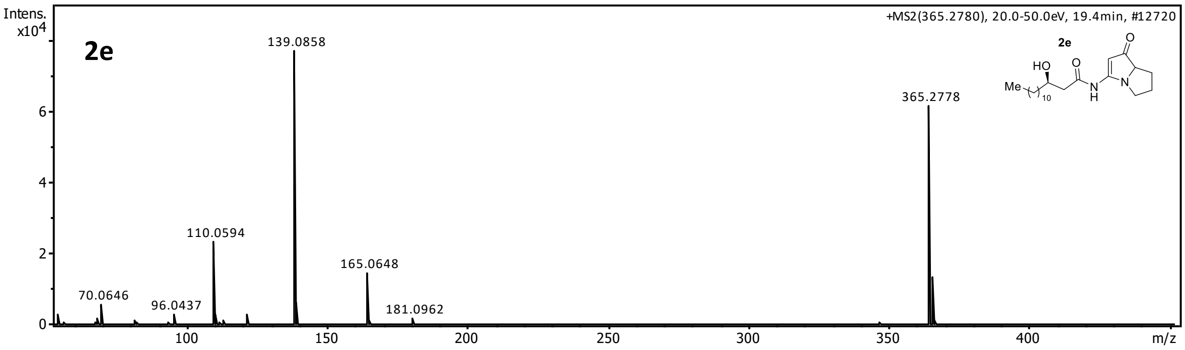

Figure S102. CID MS/MS spectrum from BraC assay with substrate **3e**, retention time 19.4 minutes.

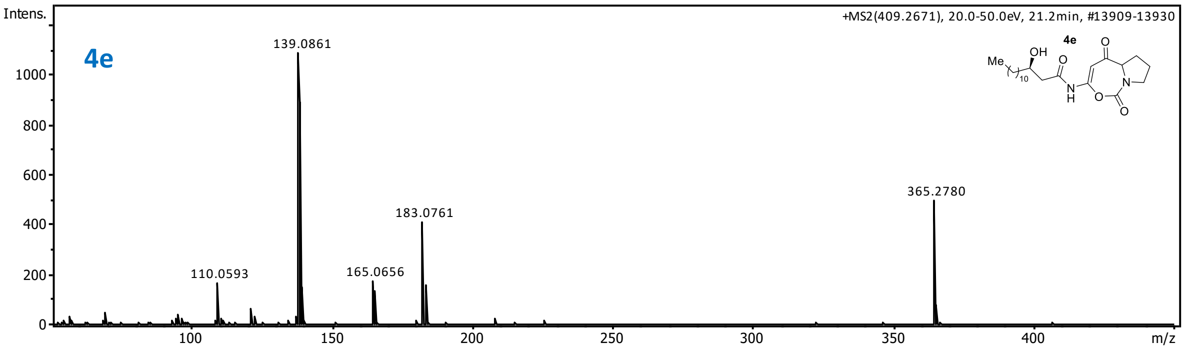

Figure S103. CID MS/MS spectrum from BraC assay with substrate **3e**, retention time 21.2 minutes, thus identifying **4e**.

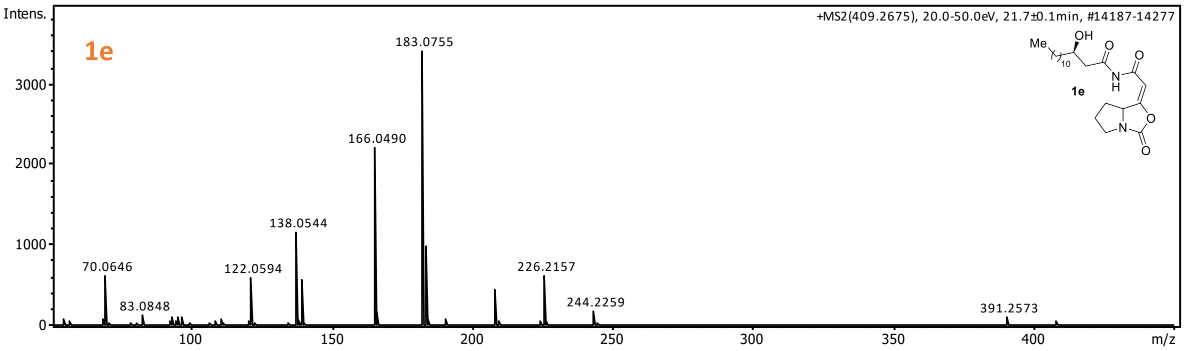

Figure S104. CID MS/MS spectrum from BraC assay with substrate **3e**, retention time 21.7 minutes, thus identifying **1e**.

5.3.6 C_{14}ORha substrate 3f

5.3.6.1 PxaB assay

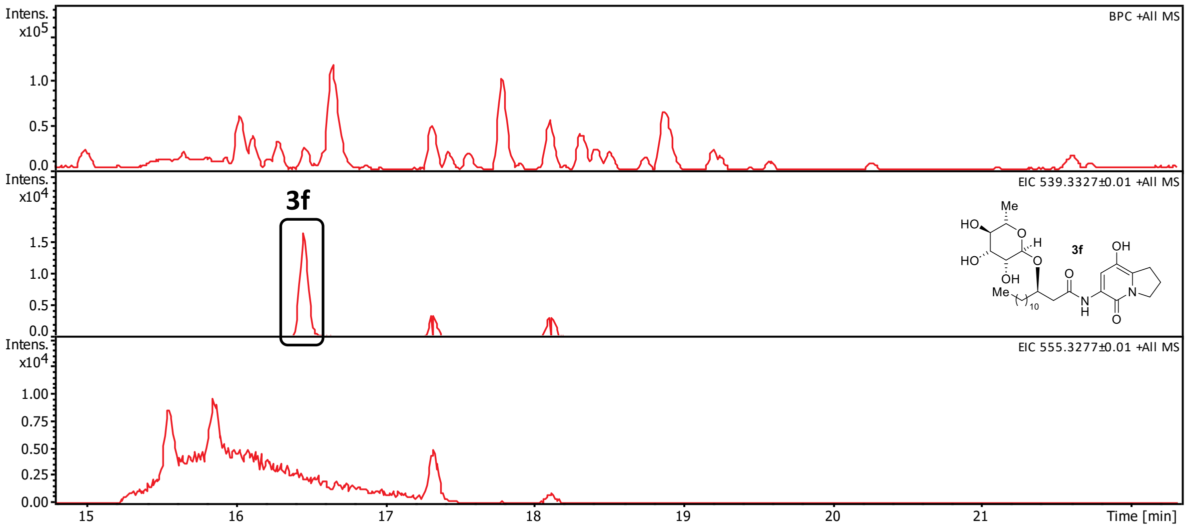

Figure S105. BPC (top) and EICs for substrate **3f** (middle) and oxidized degradation products (bottom) of negative control for substrate **3f**.

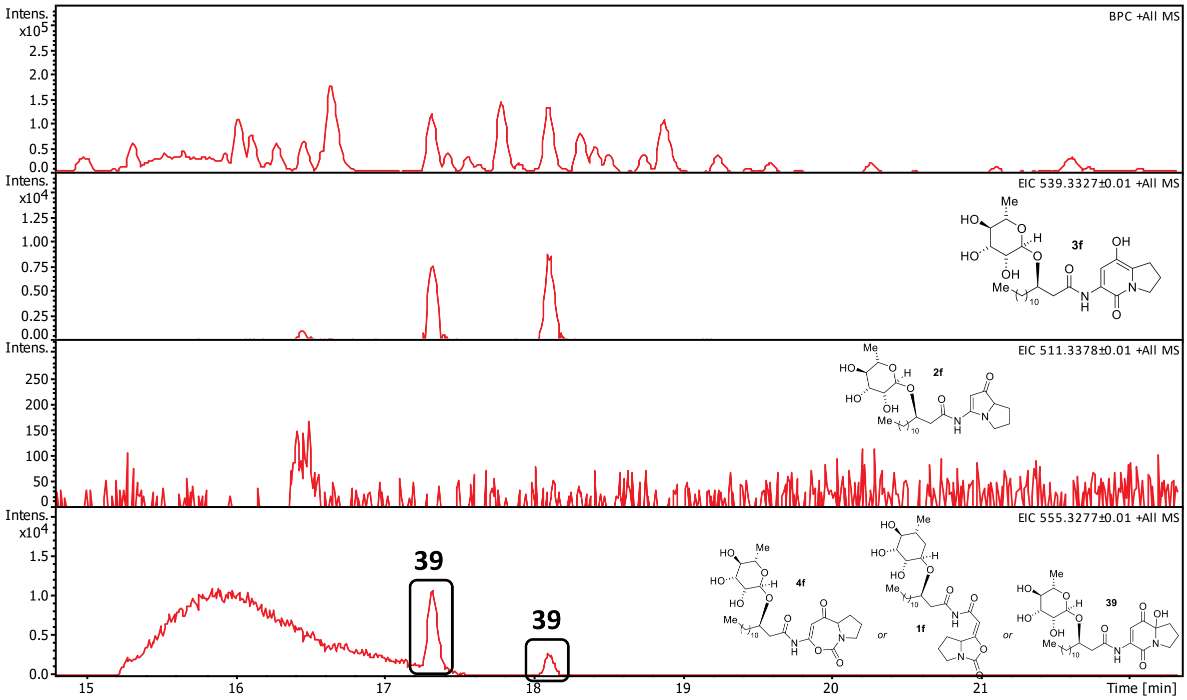

Figure S106. BPC (line one) and EICs for substrate **3f**, PA derivative **2f**, and oxidized products **4f** or **1f** or **39** (from line two to four) of the enzyme assay employing PxaB and substrate **3f**.

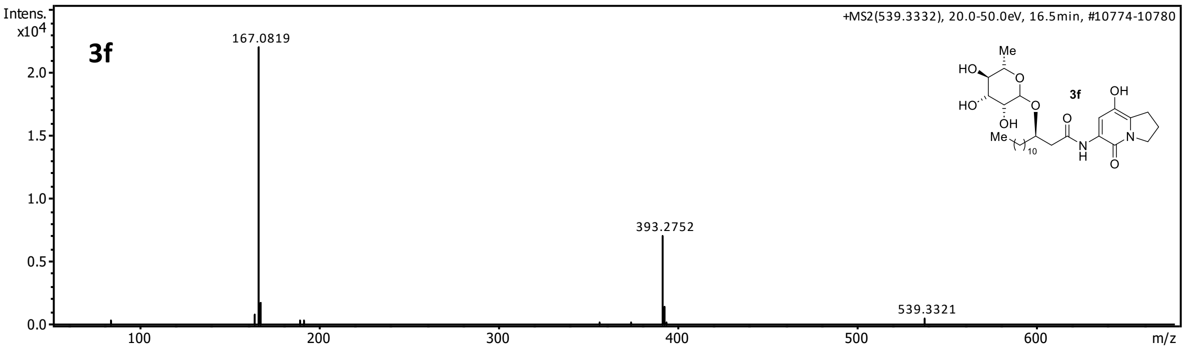

Figure S107. CID MS/MS spectrum from negative control with substrate **3f**, retention time 16.5 minutes.

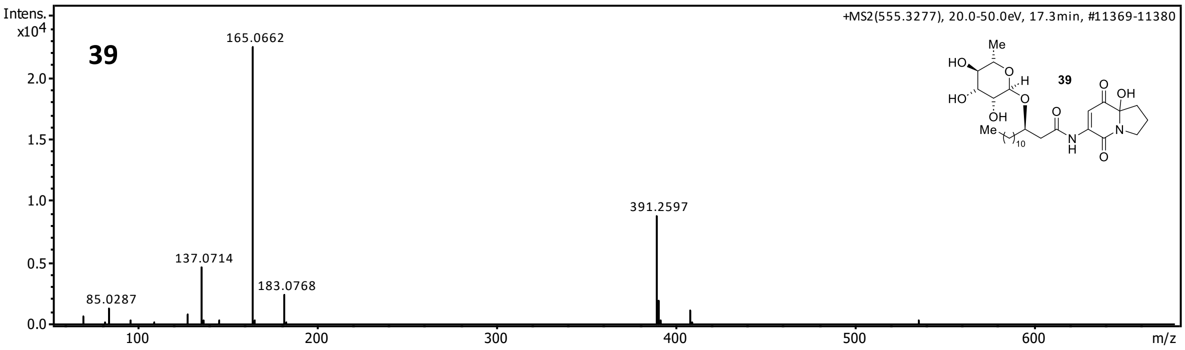

Figure S108. CID MS/MS spectrum from PxaB assay with substrate **3f**, retention time 17.3 minutes, thus identifying more polar isomer of **39**.

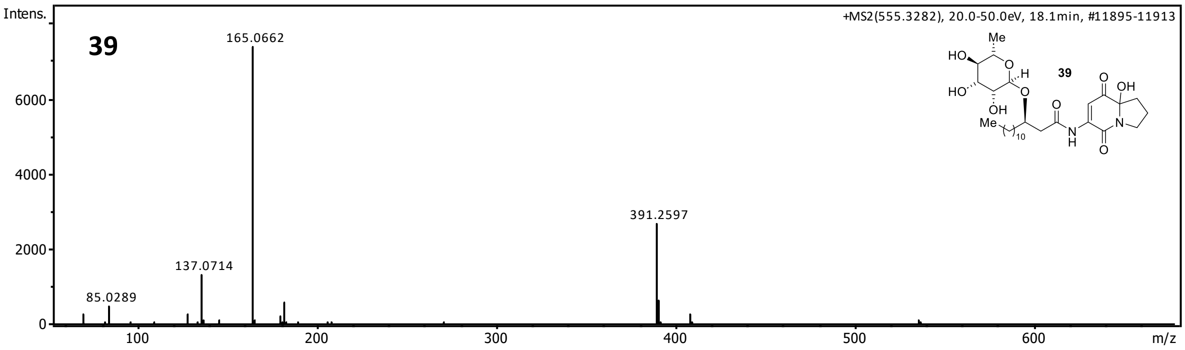

Figure S109. CID MS/MS spectrum from PxaB assay with substrate **3f**, retention time 18.1 minutes, thus identifying less polar isomer of **39**.

5.3.6.2 BraC assay

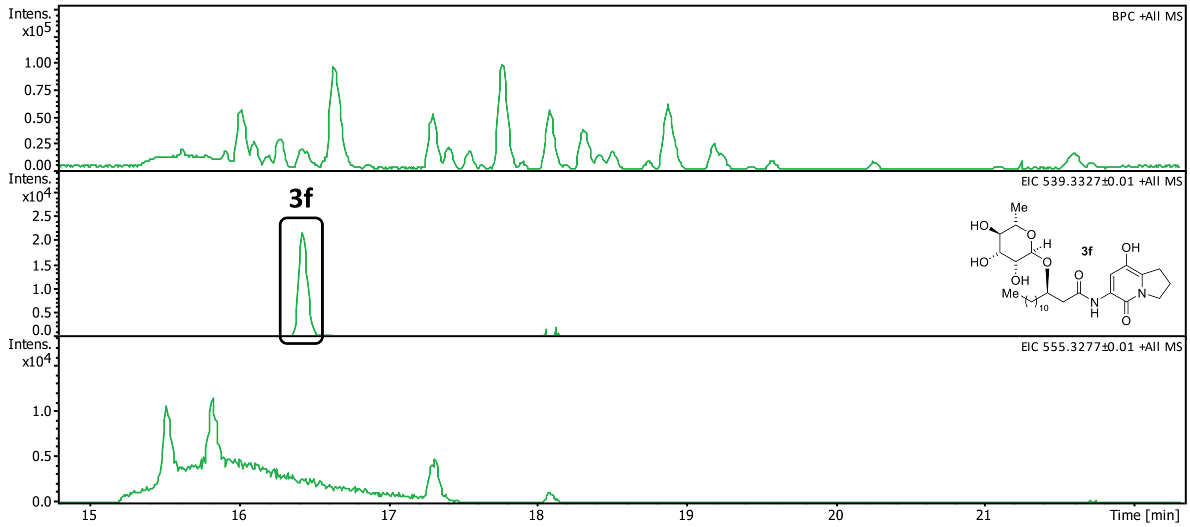

Figure S110. BPC (top) and EIC for substrate **3f** (middle) and oxidized degradation products (bottom) of negative control for substrate **3f**.

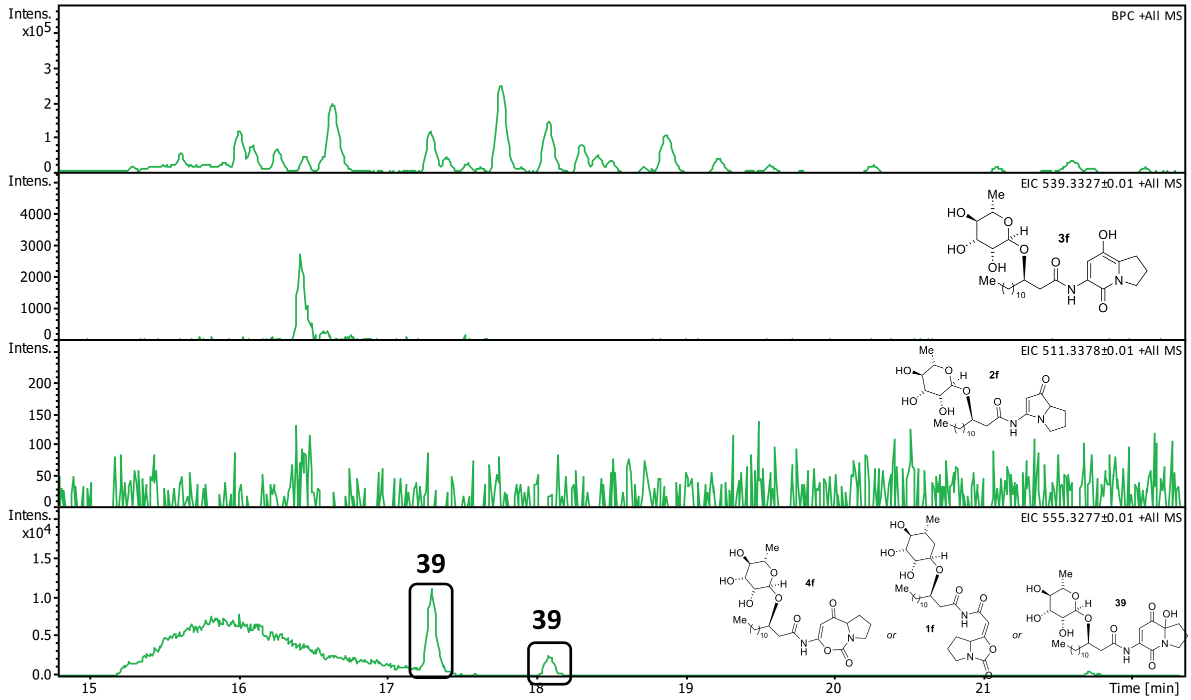

Figure S111. BPC (line one) and EICs for substrate **3f**, PA derivative **2f**, and oxidized products **4f** or **1f** or **39** (from line two to four) of the enzyme assay employing BraC and substrate **3f**.

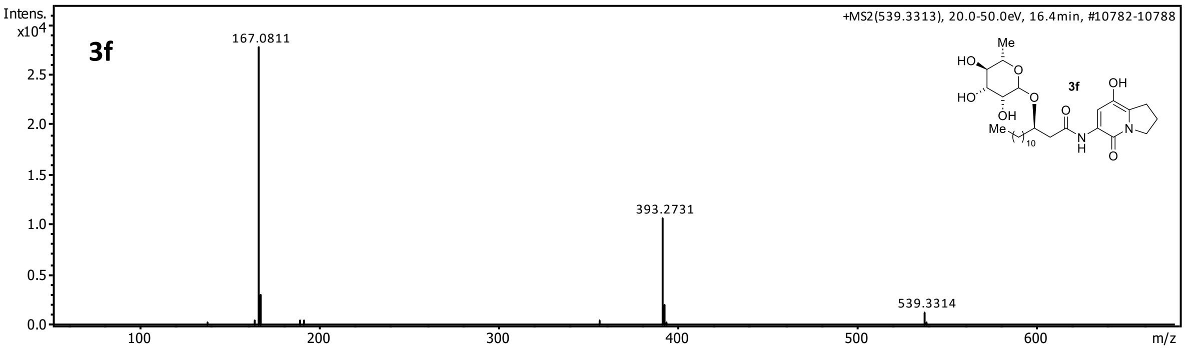

Figure S112. CID MS/MS spectrum from negative control with substrate **3f**, retention time 16.4 minutes.

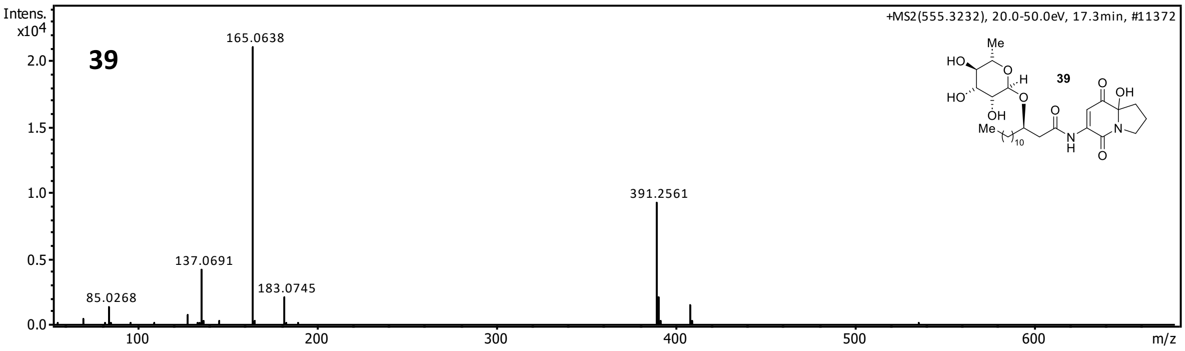

Figure S113. CID MS/MS spectrum from BraC assay with substrate **3f**, retention time 17.3 minutes, thus identifying **39**.

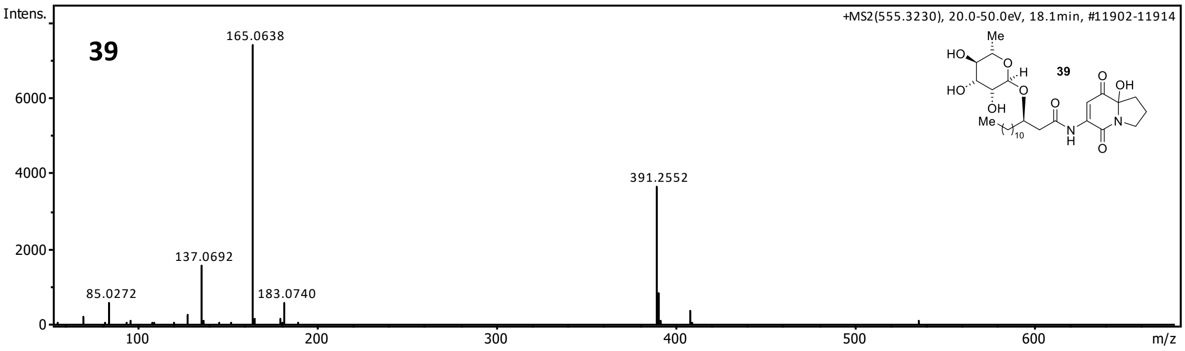

Figure S114. CID MS/MS spectrum from BraC assay with substrate **3f**, retention time 18.1 minutes, thus identifying **39**.

5.3.7 PxaB assays with other substrates

5.3.7.1 Core structures without side chain

2,3-dihydroindolizin-5(1*H*)-one (**53**)

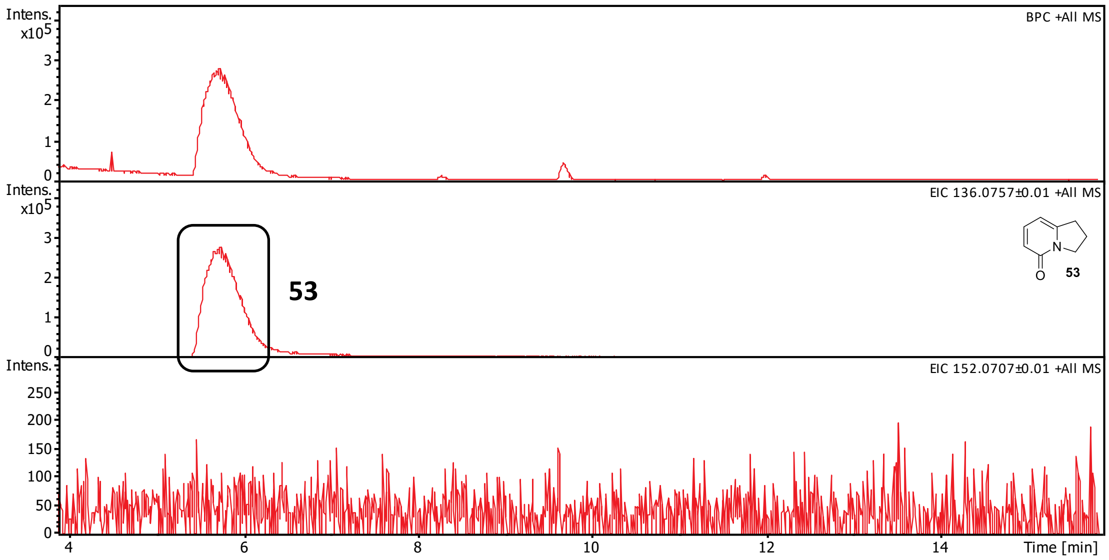

Figure S115. BPC (top) and EIC for substrate **53** (middle) and oxidized degradation products (bottom) of negative control for substrate **53**.

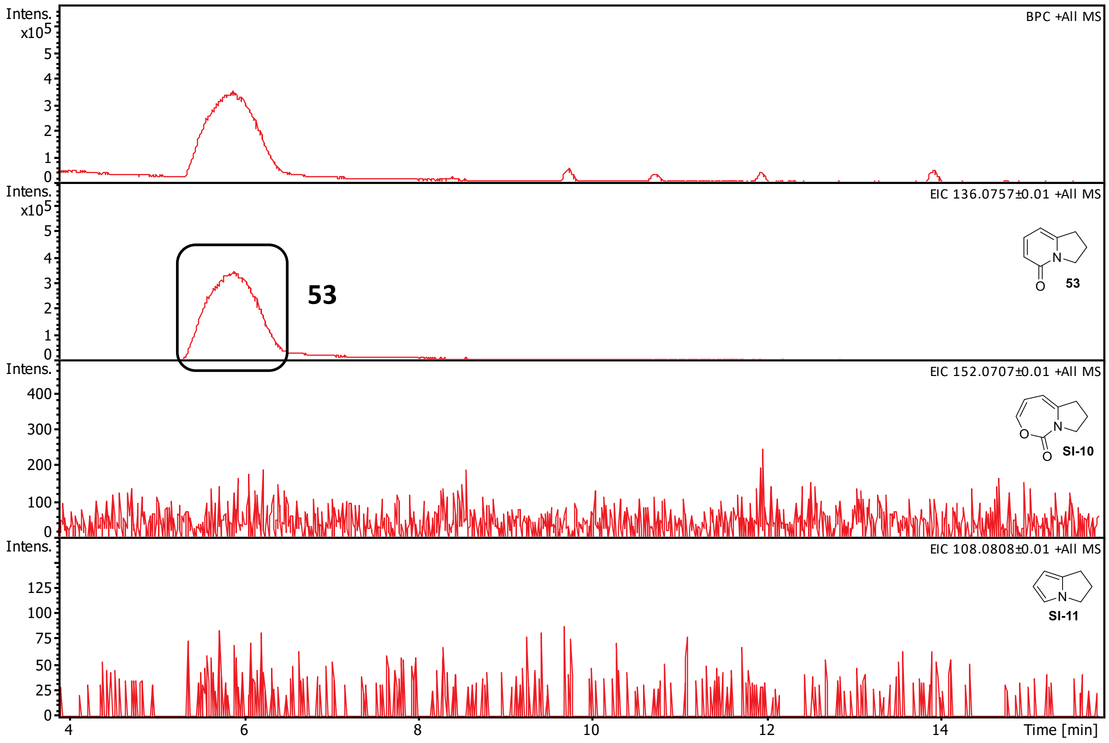

Figure S116. BPC (line one) and EICs for substrate **53**, hypothetical oxidized product **SI-10**, and hypothetical PA derivative **SI-11**, (from line two to four) of the enzyme assay employing PxaB and substrate **53**.

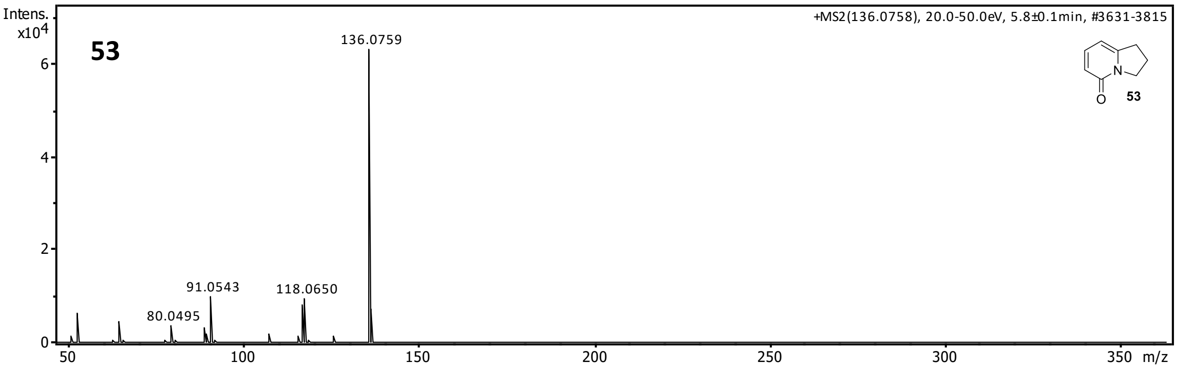

Figure S117. CID MS/MS spectrum from negative control with substrate **53**, retention time 5.8 minutes.

Alcohol **54**

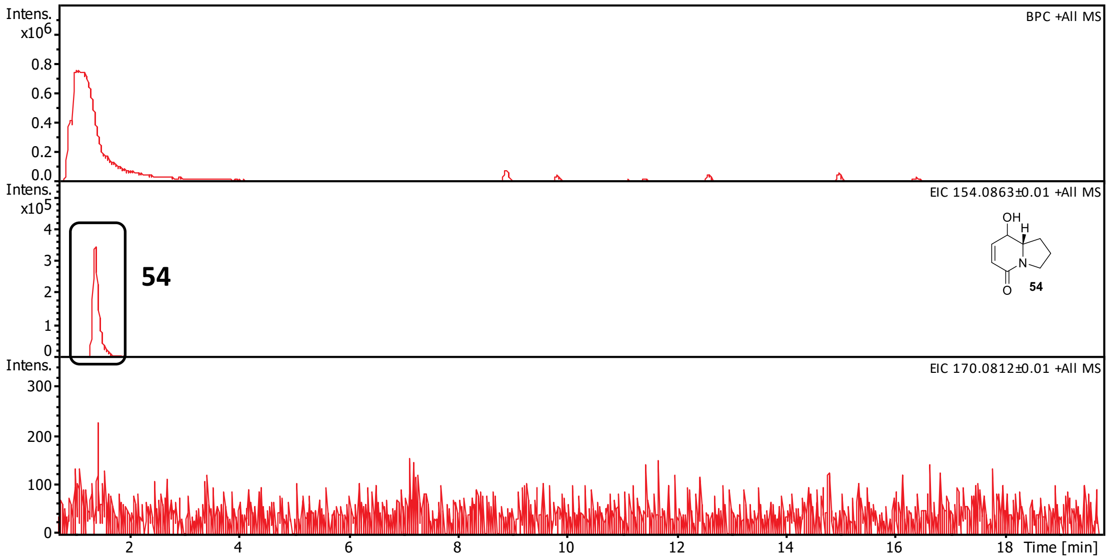

Figure S118. BPC (top) and EIC for substrate **54** (middle) and oxidized degradation products (bottom) of negative control for substrate **54**.

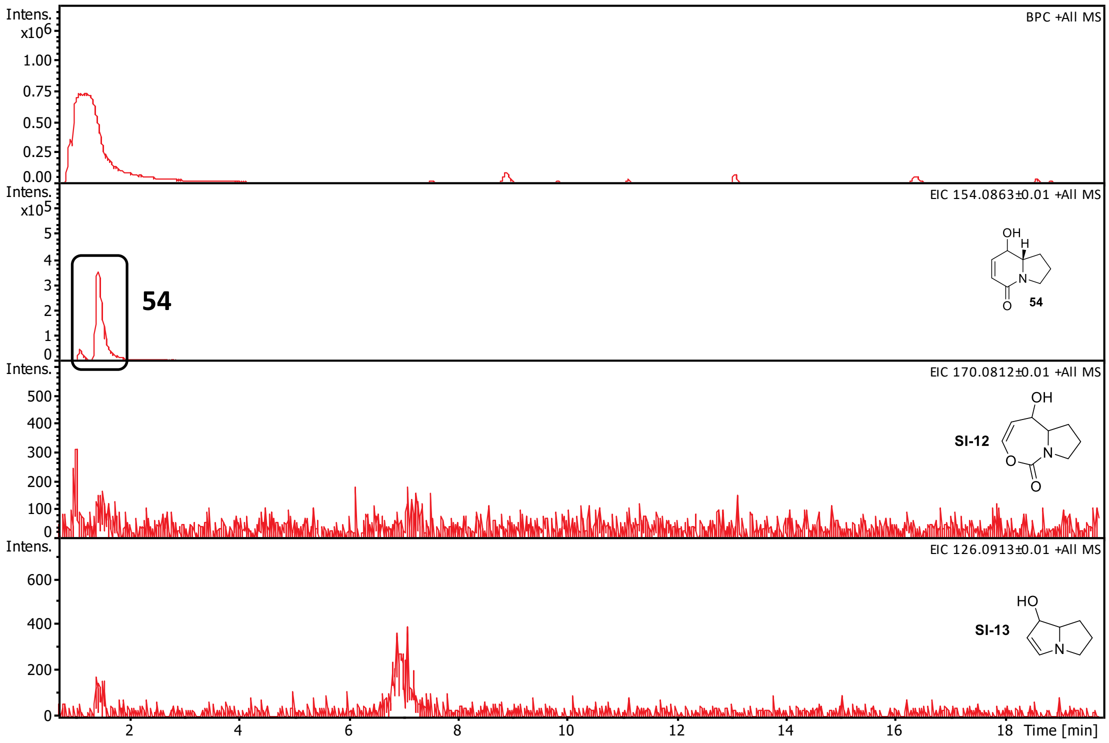

Figure S119. BPC (line one) and EICs for substrate **54**, hypothetical oxidized product **SI-12**, and hypothetical PA derivative **SI-13**, (from line two to four) of the enzyme assay employing PxaB and substrate **54**.

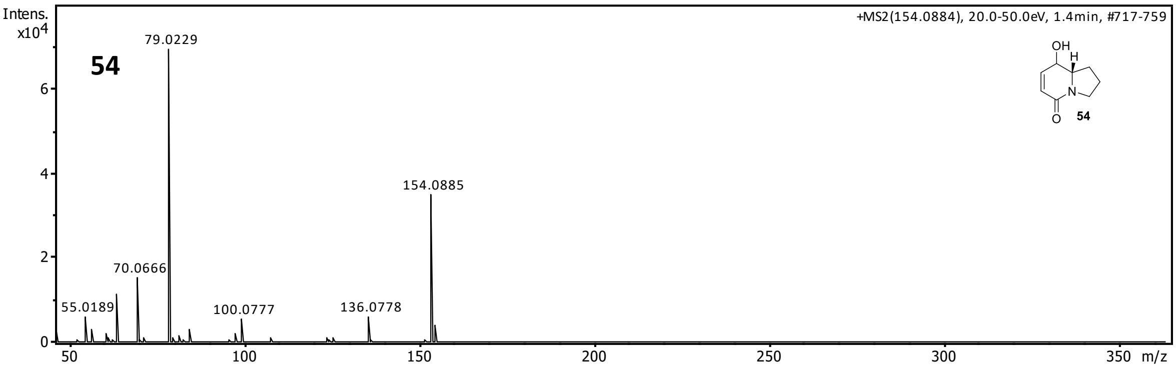

Figure S120. CID MS/MS spectrum from negative control with substrate **54**, retention time 1.4 minutes.

5.3.7.2 Non-hydroxylated C_6-substrate **27a**

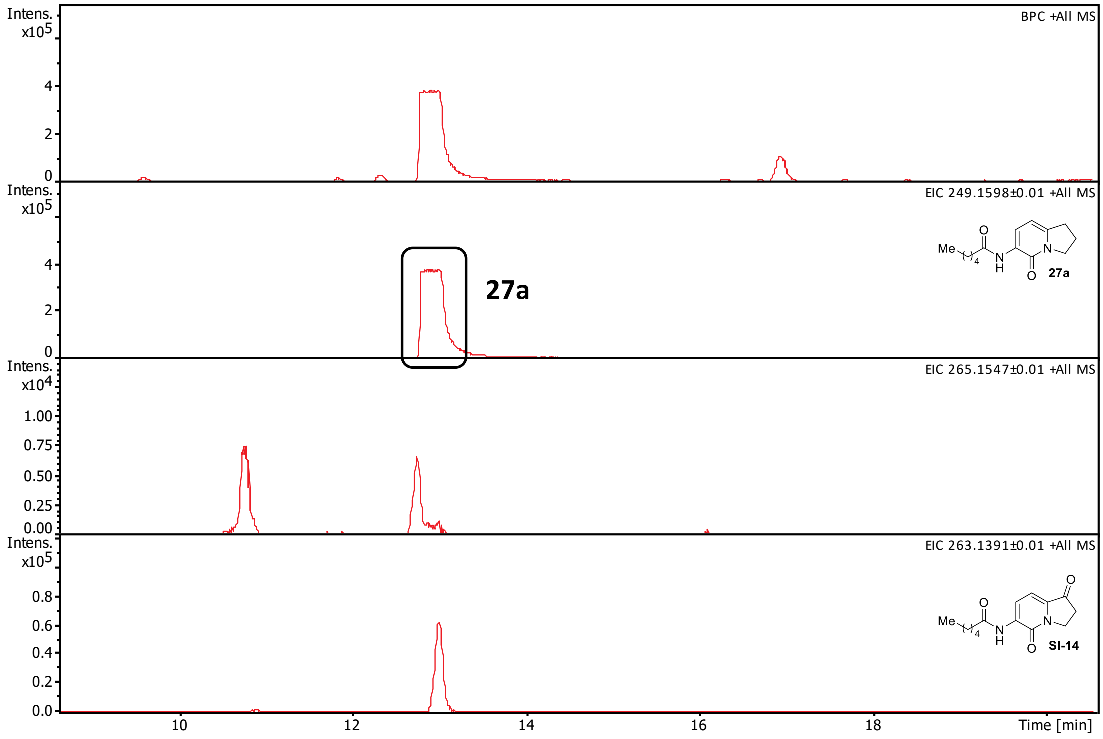

Figure S121. BPC (line one) and EIC for substrate **27a** (line two) and oxidized degradation products (line three) of negative control for substrate **27a**. Note: Substrate **27a** was contaminated with approx. 5% of inseparable **SI-14** (EIC in line four; amount and structure elucidated by NMR; presumably resulting from allylic air oxidation during prolonged standing of compound **27a** at room temperature before use for enzyme assay).

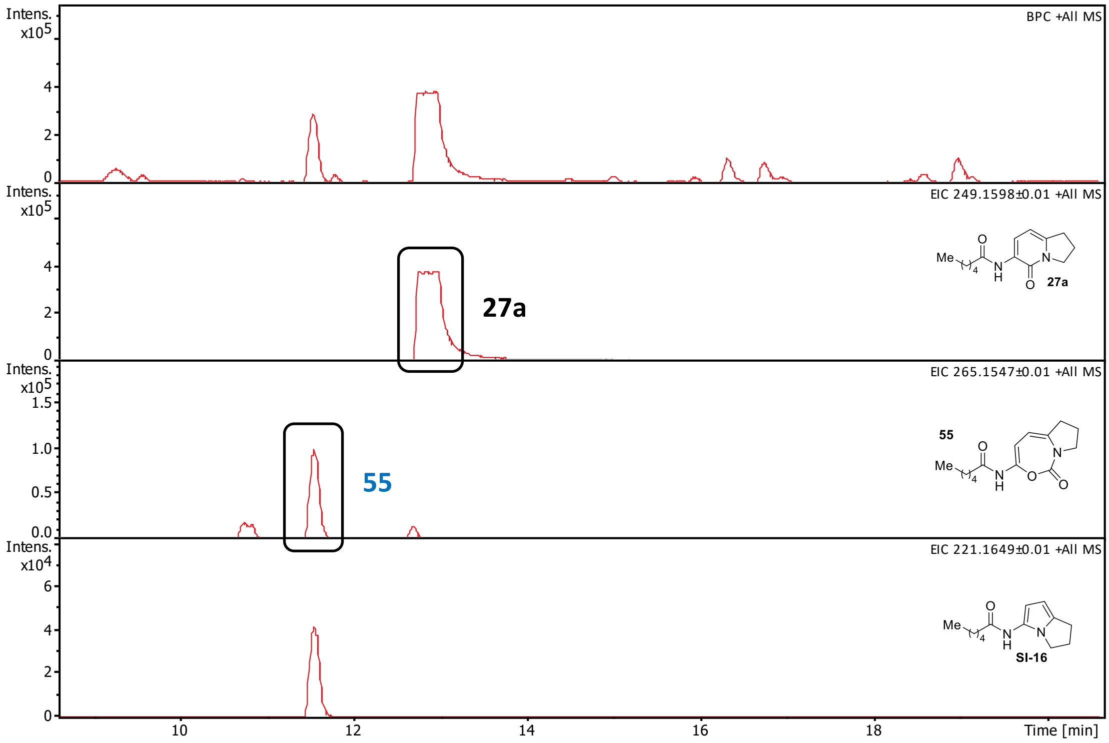

Figure S122. BPC (line one) and EICs for substrate **27a**, hypothetical oxidized product **55**, and hypothetical PA derivative **SI-15**, (from line two to four) of the enzyme assay employing PxaB and substrate **27a**. Peak for mass of **SI-15** in line four results from in-source decarboxylation of **56**.

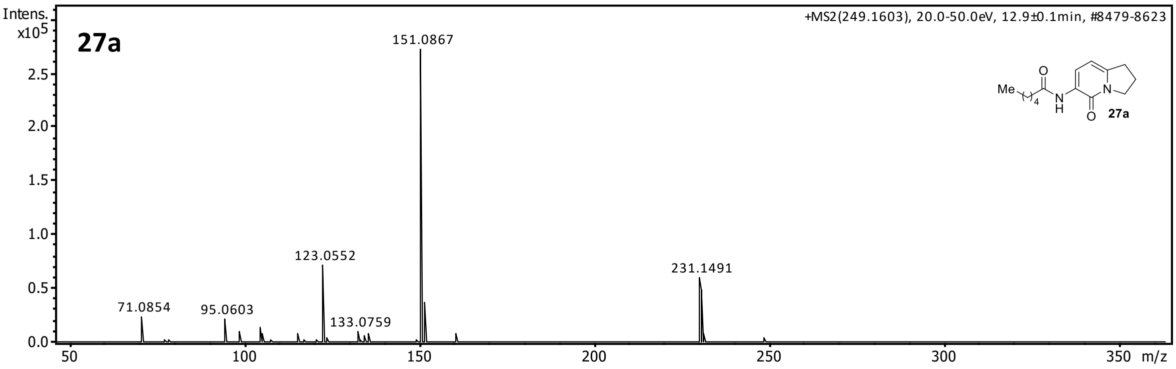

Figure S123. CID MS/MS spectrum from negative control with substrate **27a**, retention time 12.9 minutes.

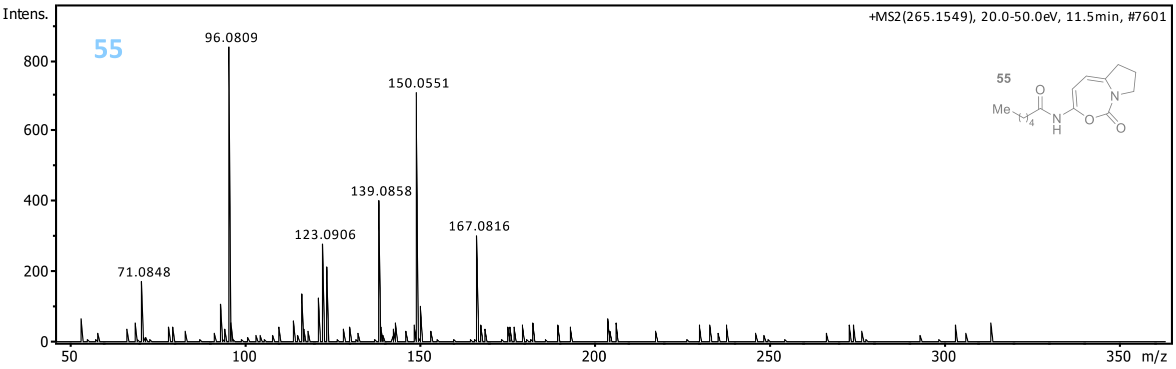

Figure S124. CID MS/MS spectrum from PxaB assay with substrate **27a**, retention time 11.5 minutes, thus presumably identifying **55** (low number of counts).

5.4 Stability tracking of intermediate 4

Two to three 200 µL assays as described in chapter 5.3 (employing **3a/c/e** and PxaB) were extracted with EtOAc (3 × 200 µL) after 75 minutes at 30 °C, and the solvent of combined organic phases was removed by Speedvac evaporation. The residues were dissolved in 120 µL MeOH or assay buffer (with 10% DMSO) each and combined, followed by LC-HRMS analysis (10 µL injection volume) at different times, while being incubated at 33 °C. Injection volume for NCs was 5 µL.

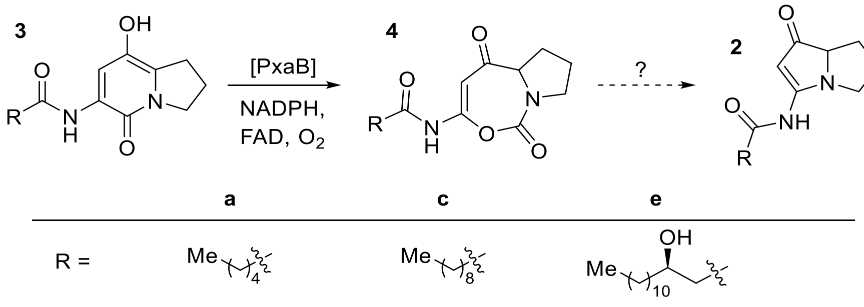

Figure S125. Substrates **3** used for tracking of stability of enzymatic intermediates **4**.

5.4.1 C_6-Substrate 3a

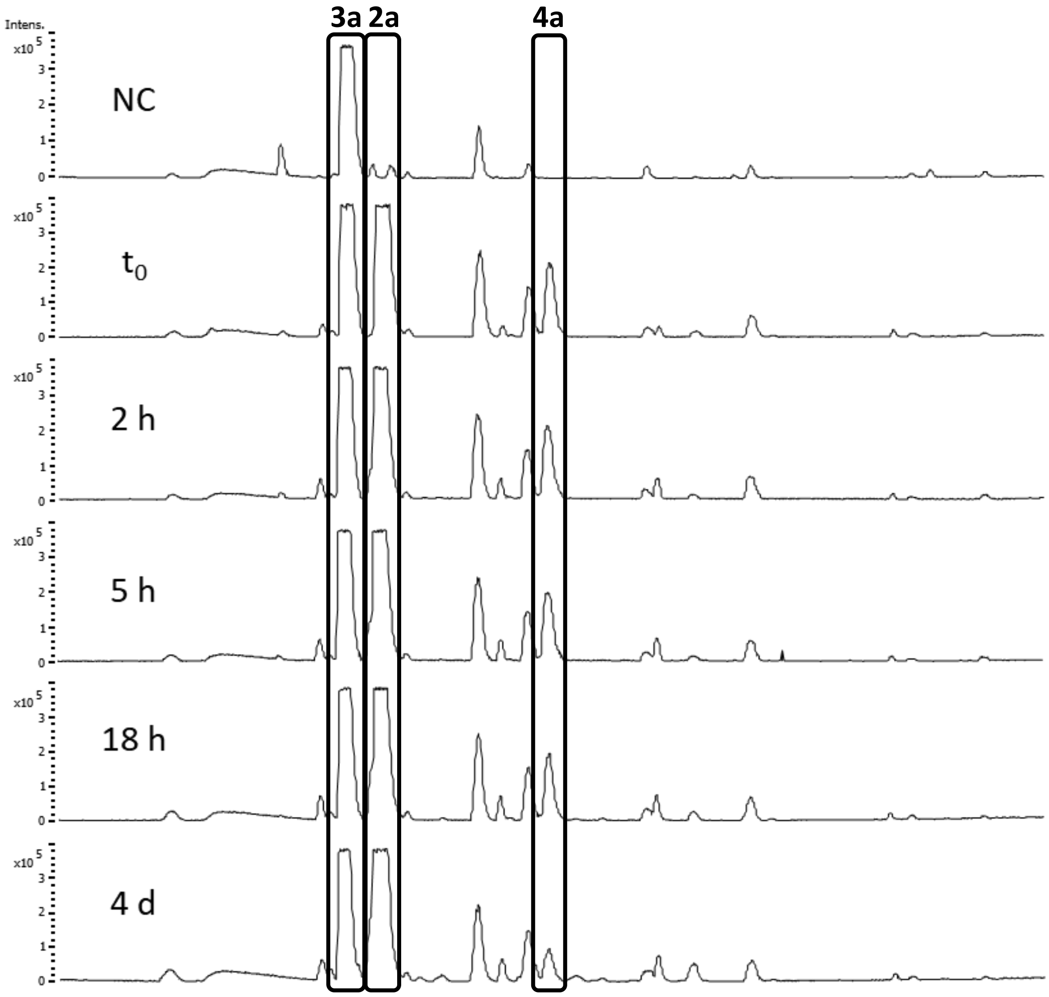

Figure S126. BPCs of PxaB assays with substrate **3a** after enzyme quenching by extraction with EtOAc, evaporation and redissolution in MeOH. NC – negative control (without enzyme), t_0 – immediate measurement after dissolution.

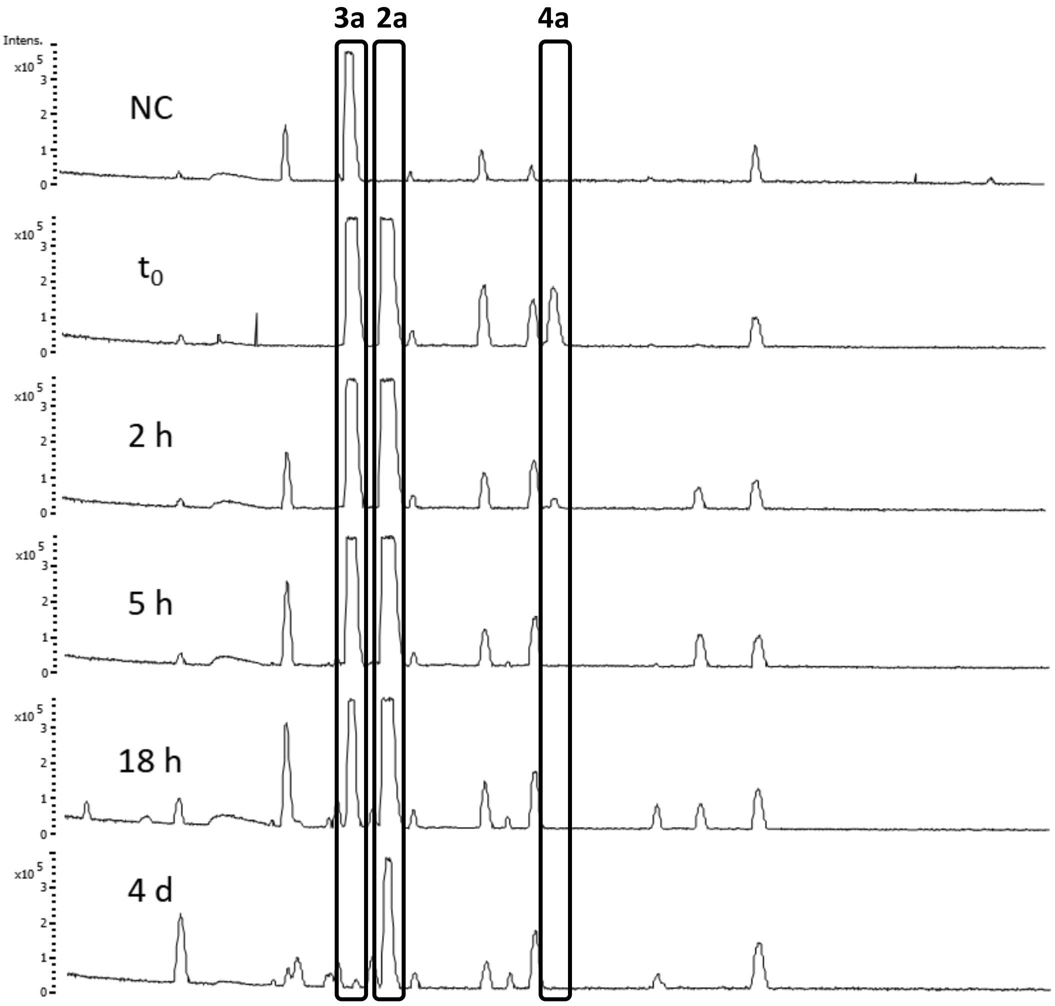

Figure S127. BPCs of PxaB assays with substrate **3a** after enzyme quenching by extraction with EtOAc, evaporation and redissolution in MeOH. NC – negative control (without enzyme), t_0 – immediate measurement after dissolution.

5.4.2 C_{10}-Substrate 3c

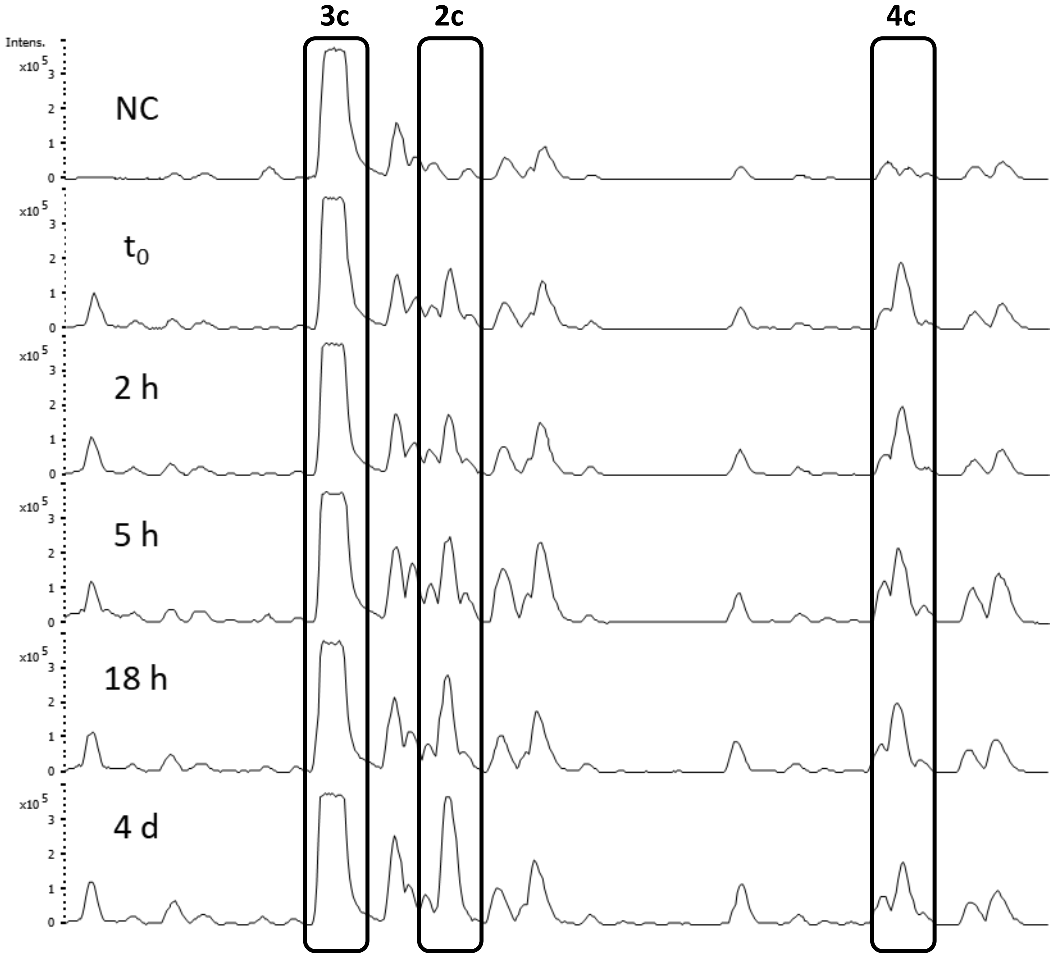

Figure S128. BPCs of PxaB assays with substrate **3c** after enzyme quenching by extraction with EtOAc, evaporation and redissolution in MeOH. NC – negative control (without enzyme), t_0 – immediate measurement after dissolution.

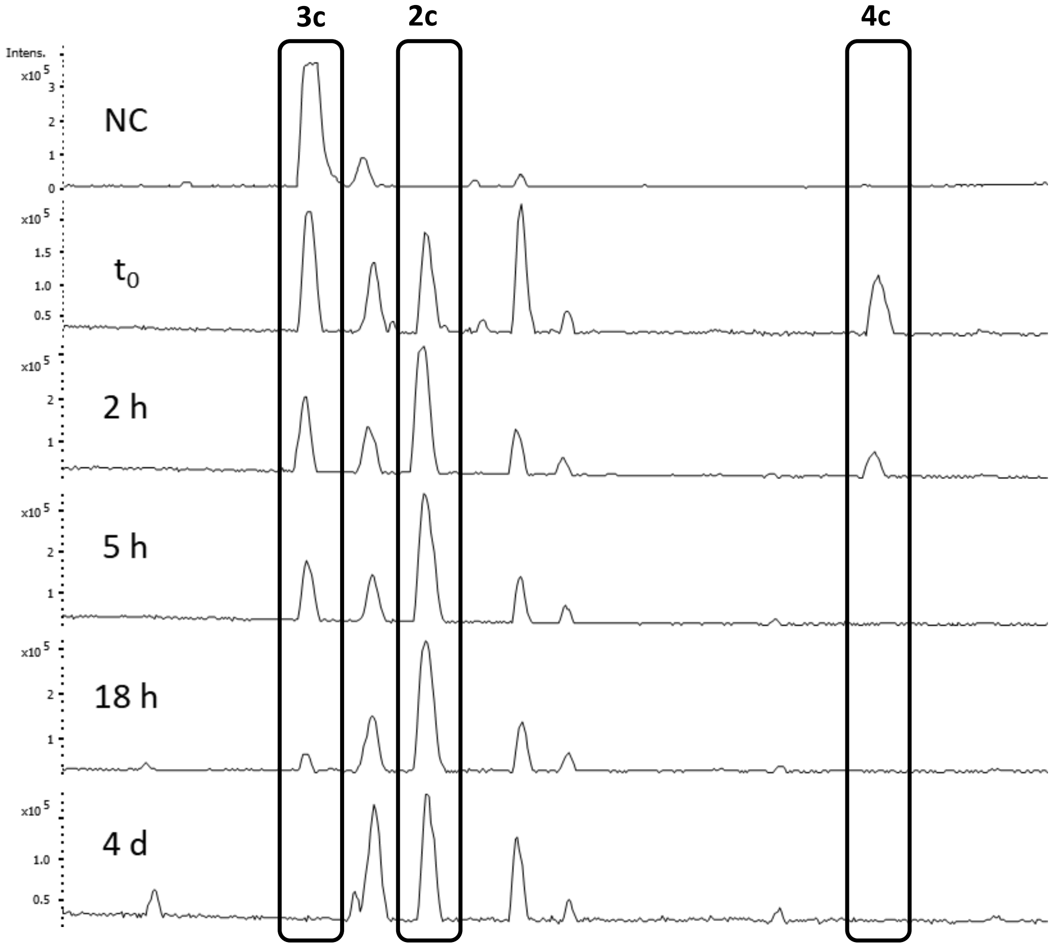

Figure S129. BPCs of PxaB assays with substrate **3c** after enzyme quenching by extraction with EtOAc, evaporation and redissolution in reaction buffer (with 10 vol-% DMSO). NC – negative control (without enzyme), t_0 – directly after dissolution.

5.4.3 C_{14}OH-Substrate 3e

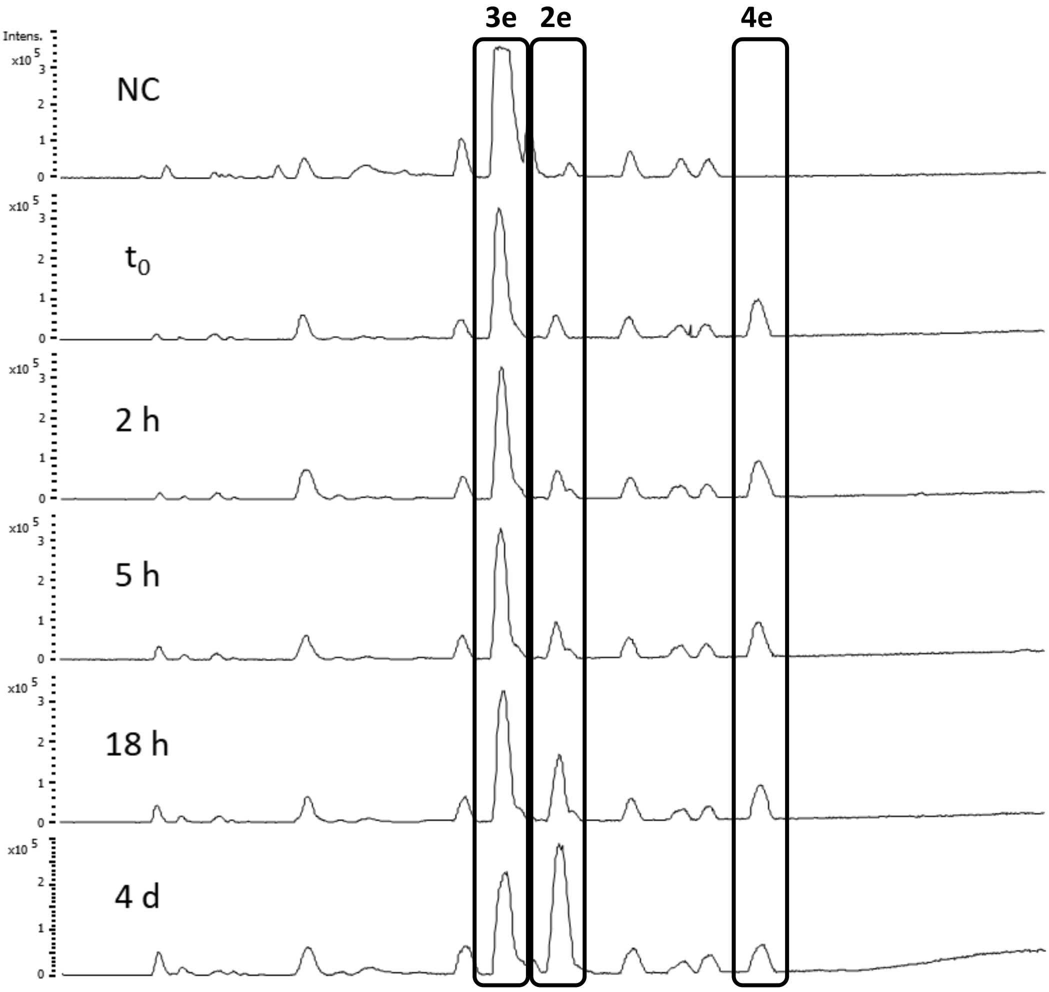

Figure S130. BPCs of PxaB assays with substrate **3e** after enzyme quenching by extraction with EtOAc, evaporation and redissolution in MeOH. NC – negative control (without enzyme), t_0 – immediate measurement after dissolution.

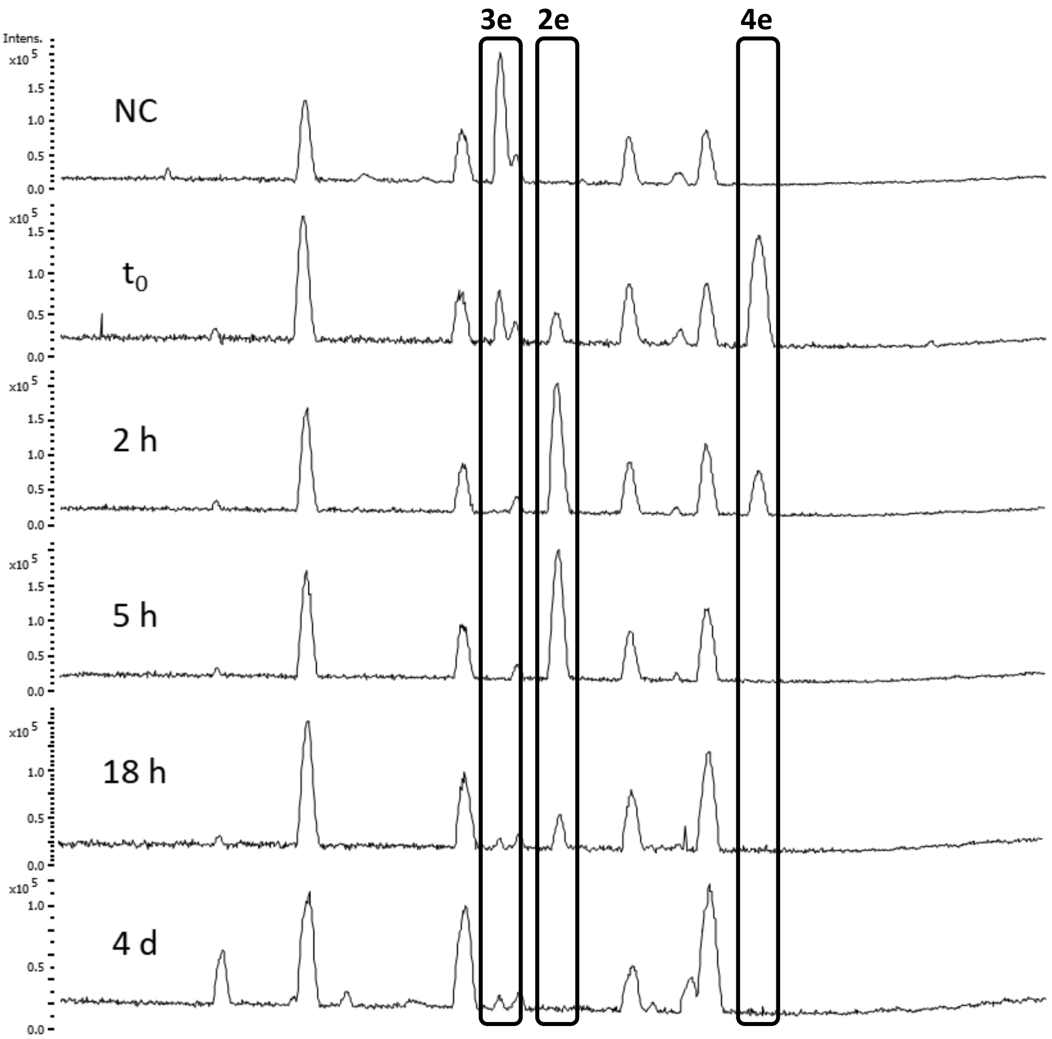

Figure S131. BPCs of PxaB assays with substrate **3c** after enzyme quenching by extraction with EtOAc, evaporation and redissolution in reaction buffer (with 10 vol-% DMSO). NC – negative control (without enzyme), t_0 – directly after dissolution.

5.5 Comparison of catalytic competence towards brabantamide analogs

Colors indicate the taken values (red PxaB; green BraC). BraC value was set to 100%.

Table S6. Comparison of **1a** production by both enzymes by EIC peak integration.

**3a**	PxaB					BraC	
	Peak Area of **1a**	Peak Area Average	Normalized to BraC [%]	Normalized – Average [%]	Standard Deviation [%]	Peak Area of **1a**	Peak Area Average
Entry 1	5679		4,952			113340	
Entry 2	5559,6	5452	4,848	4,755	0,211	115999	114669,5
Entry 3	5117,4		4,463			-	

Table S7. Comparison of **1b** production by both enzymes by EIC peak integration.

**3b**	PxaB					BraC	
	Peak Area of **1b**	Peak Area Average	Normalized to BraC [%]	Normalized – Average [%]	Standard Deviation [%]	Peak Area of **1b**	Peak Area Average
Entry 1	5706,9		4,571			125312	
Entry 2	5781,1	5983,6	4,631	4,793	0,273	124367	124839,5
Entry 3	6462,8		5,177			-	

Table S8. Comparison of **1c** production by both enzymes by EIC peak integration.

**3c**	PxaB					BraC	
	Peak Area of **1c**	Peak Area Average	Normalized to BraC [%]	Normalized – Average [%]	Standard Deviation [%]	Peak Area of **1c**	Peak Area Average
Entry 1	24392		21,603			113317	
Entry 2	24822	24466	21,984	21,669	0,235	112499	112908
Entry 3	24184		21,419			-	

Table S9. Comparison of **1d** production by both enzymes by EIC peak integration.

**3d**	PxaB					BraC	
	Peak Area of **1d**	Peak Area Average	Normalized to BraC [%]	Normalized – Average [%]	Standard Deviation [%]	Peak Area of **1d**	Peak Area Average
Entry 1	10094		14,067			71759	
Entry 2	11539	10833,333	16,080	15,097	0,823	37810	54784,5
Entry 3	10867		15,144			-	

Table S10. Comparison of **1e** production by both enzymes by EIC peak integration.

**3e**	PxaB					BraC	
	Peak Area of **1e**	Peak Area Average	Normalized to BraC [%]	Normalized – Average [%]	Standard Deviation [%]	Peak Area of **1e**	Peak Area Average
Entry 1	81438		94,504			86174	
Entry 2	74494	74914,666	86,446	86,934	5,992	65603	75888,5
Entry 3	68812		79,852			-	

6. NMR Spectra

6.1 2D NMR analysis of NRPS products (produced *in vivo*)

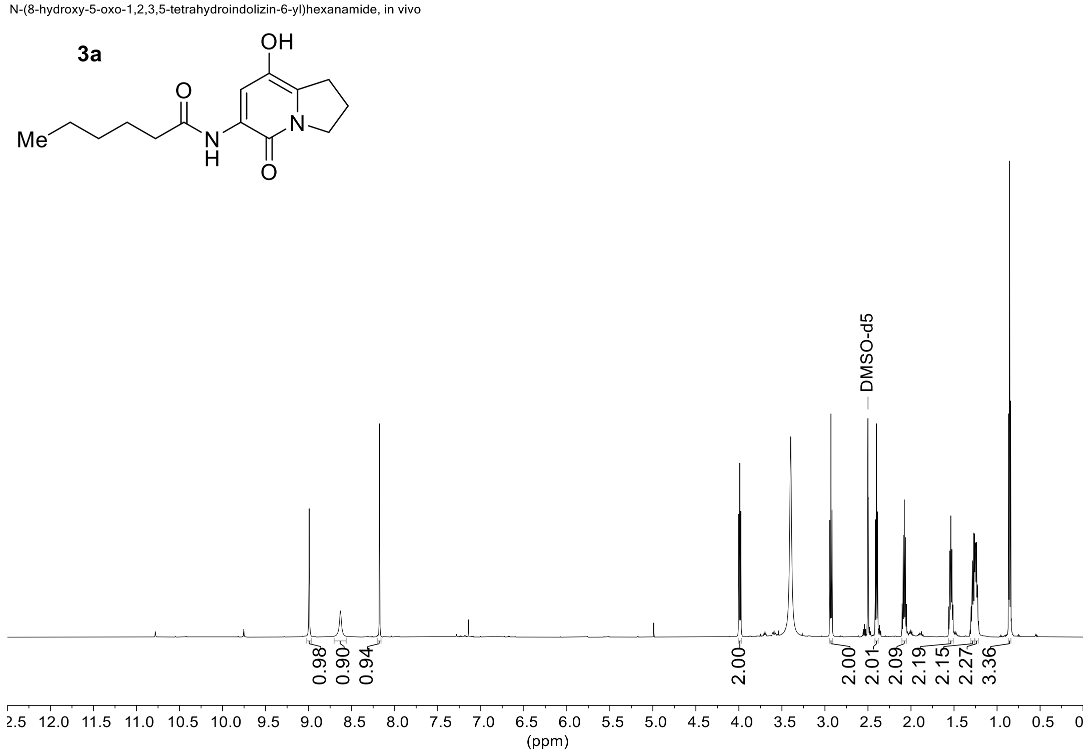

Figure S132. ^{1}H-NMR spectrum (600 MHz) of *N*-(8-hydroxy-5-oxo-1,2,3,5-tetrahydroindolizin-6-yl)hexanamide (**3a**).

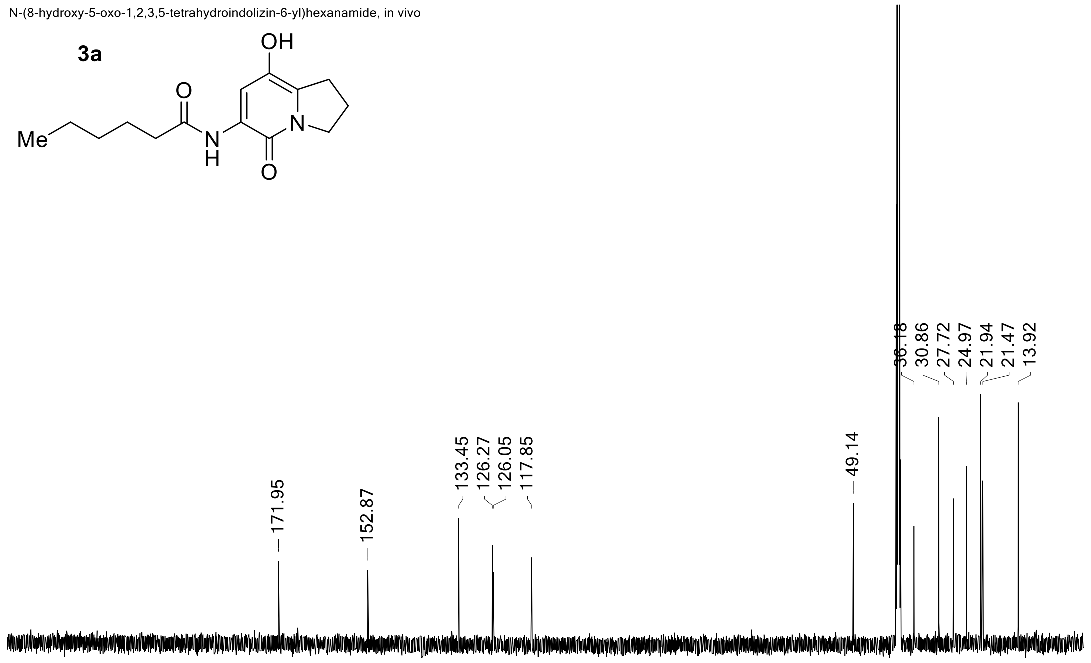

Figure S133. ^{13}C {^{1}H}-NMR spectrum (151 MHz) of *N*-(8-hydroxy-5-oxo-1,2,3,5-tetrahydroindolizin-6-yl)hexanamide (**3a**).

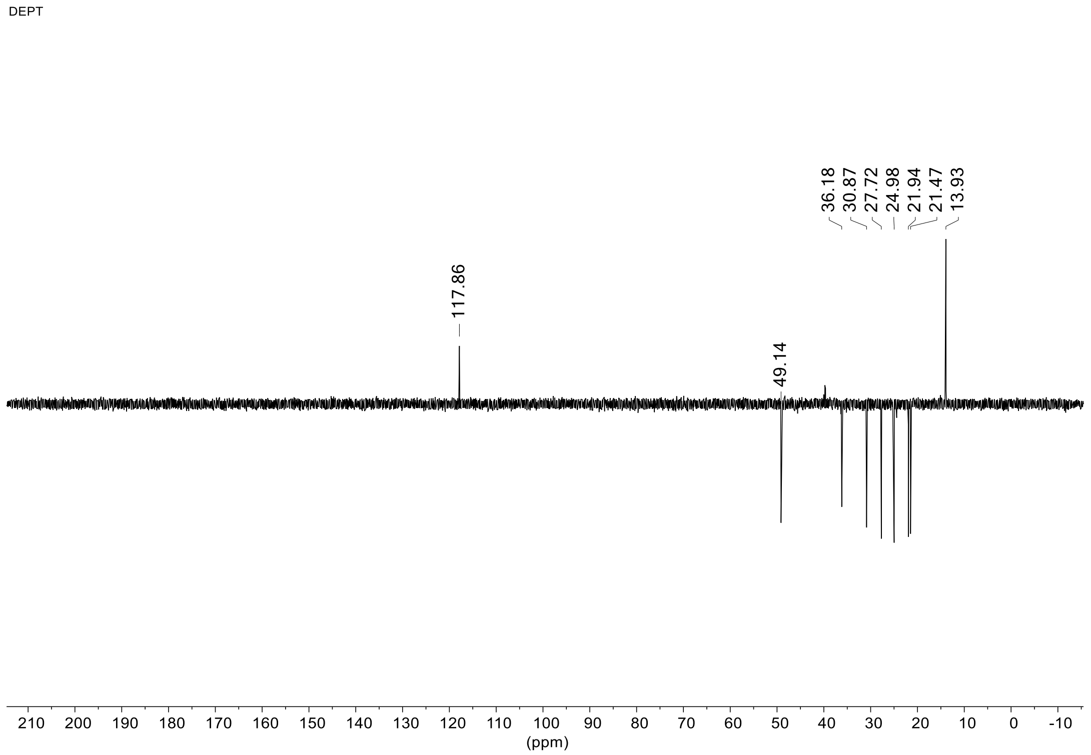

Figure S134. DEPT[135]-NMR spectrum of *N*-(8-hydroxy-5-oxo-1,2,3,5-tetrahydroindolizin-6-yl)hexanamide (**3a**).

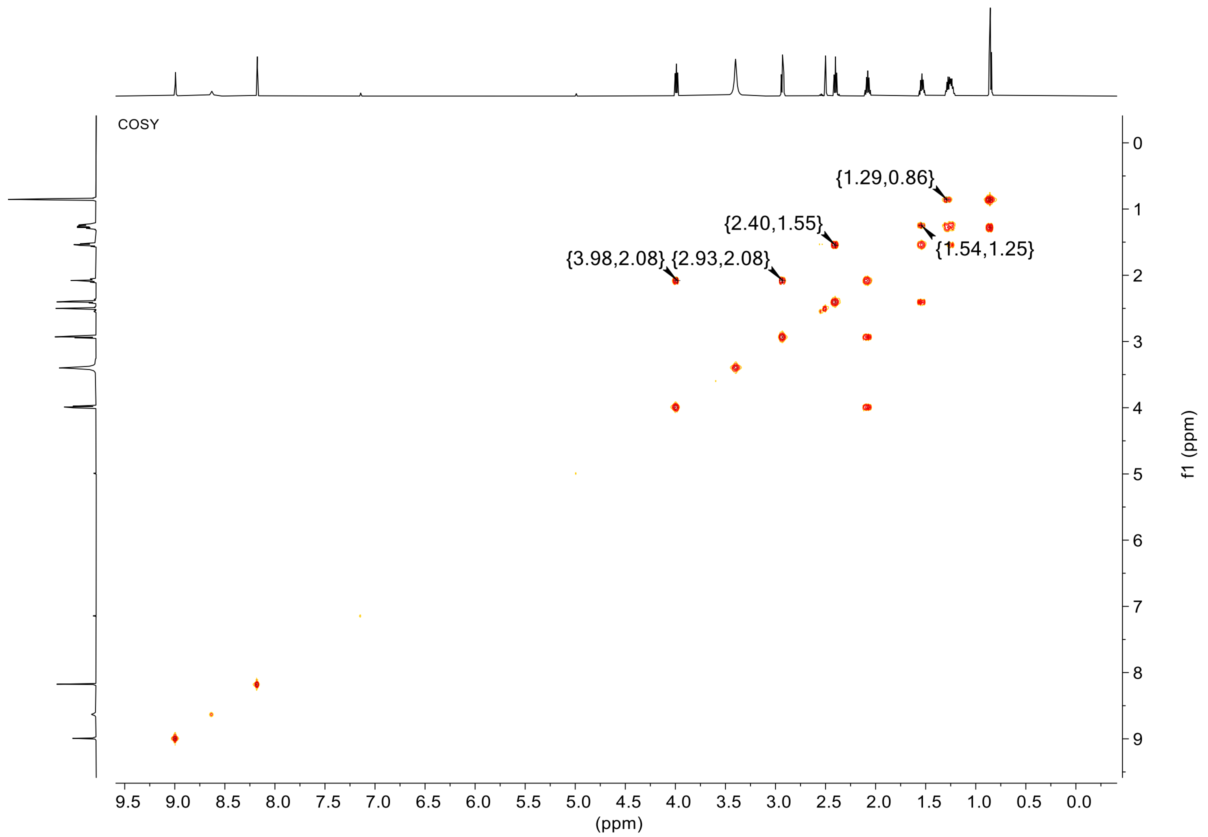

Figure S135. COSY-NMR spectrum of *N*-(8-hydroxy-5-oxo-1,2,3,5-tetrahydroindolizin-6-yl)hexanamide (**3a**).

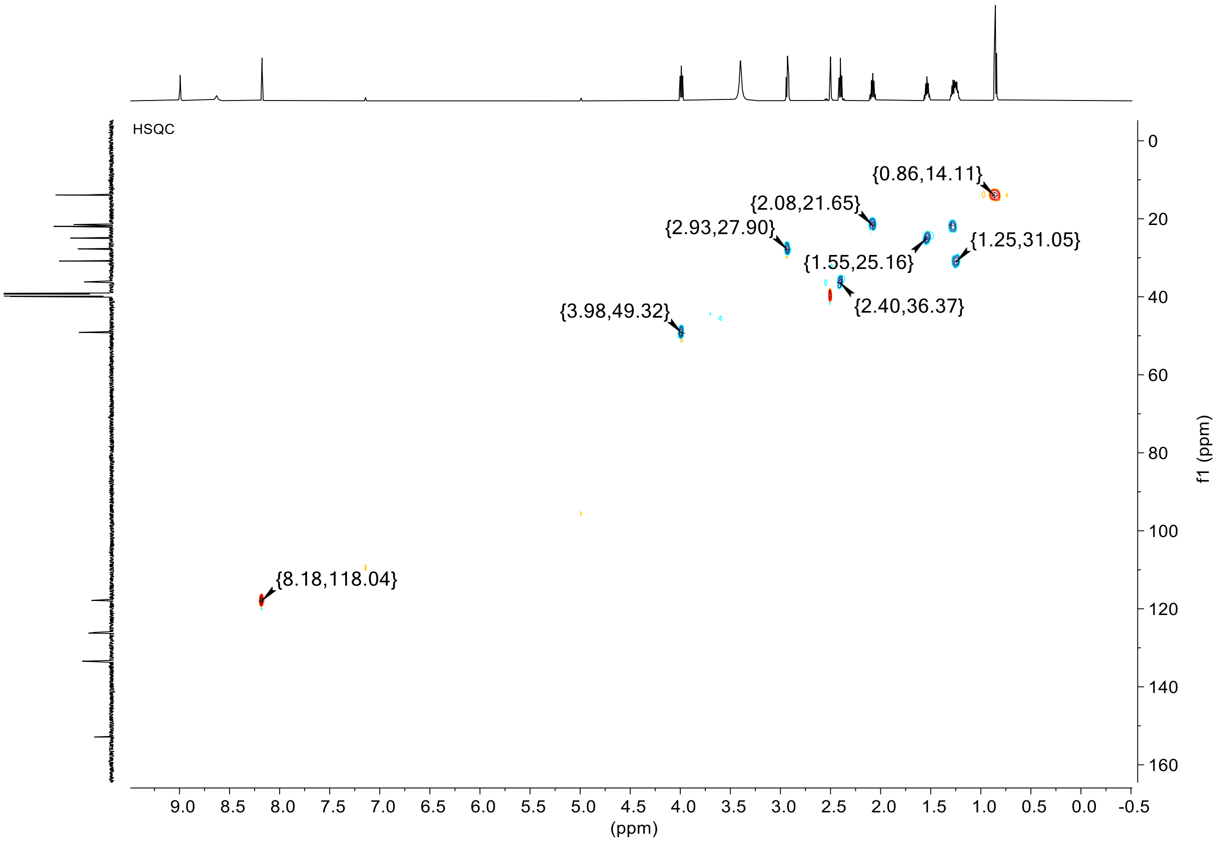

Figure S136. ^{1}H–^{13}C-HSQC-NMR spectrum of *N*-(8-hydroxy-5-oxo-1,2,3,5-tetrahydroindolizin-6-yl)hexanamide (**3a**).

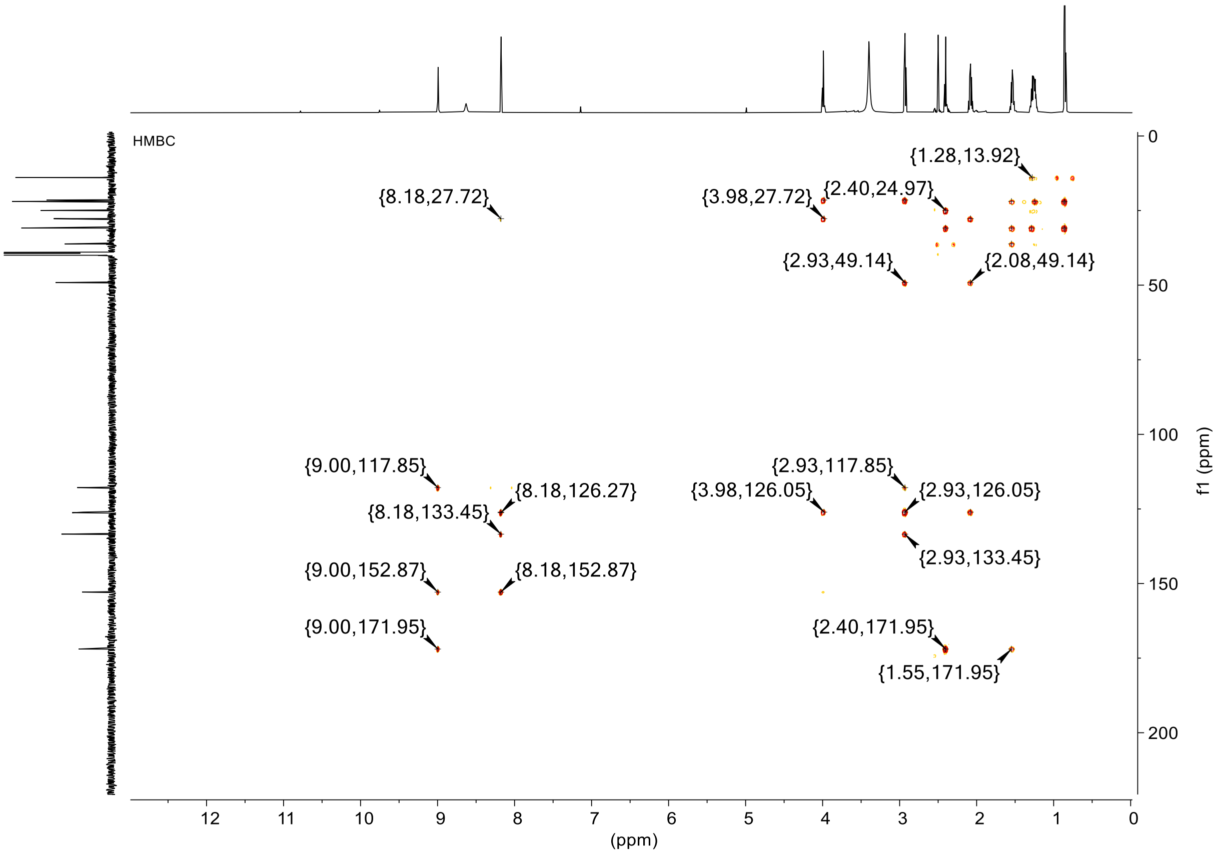

Figure S137. ^{1}H–^{13}C-HMBC-NMR of *N*-(8-hydroxy-5-oxo-1,2,3,5-tetrahydroindolizin-6-yl)hexanamide (**3a**).

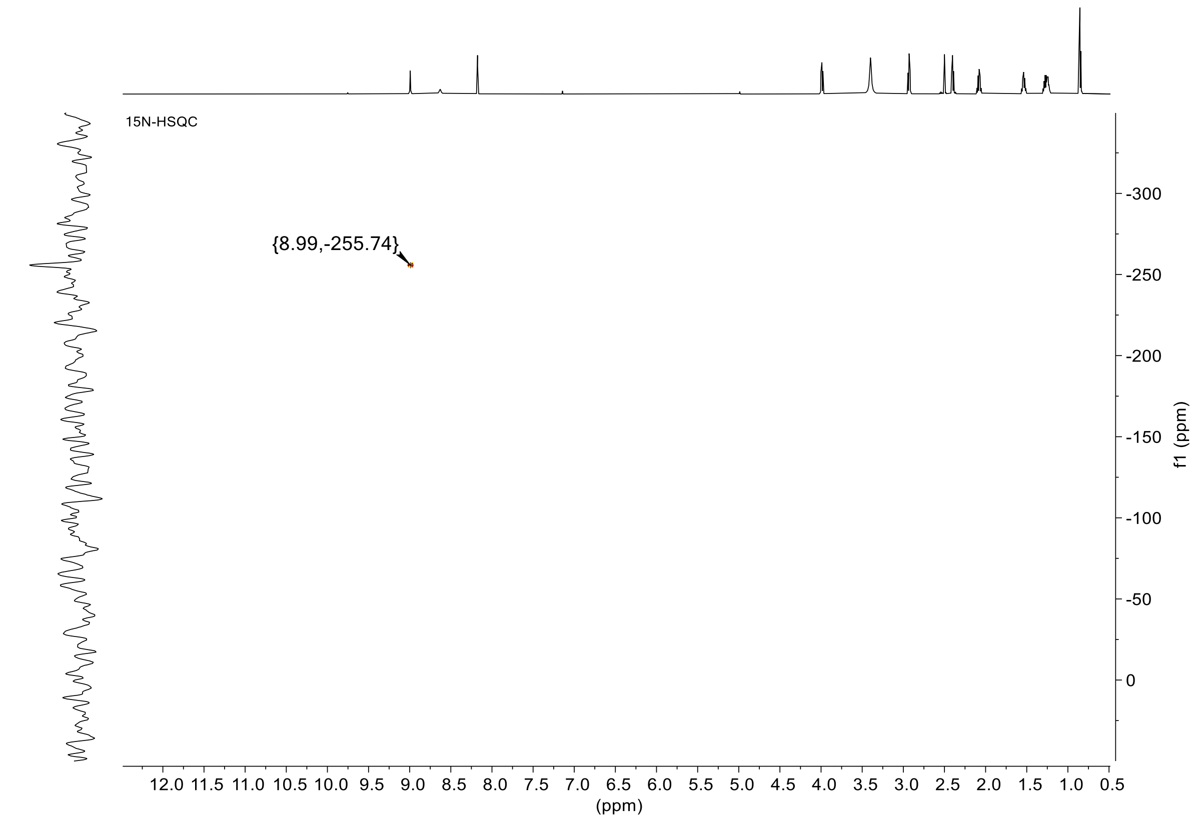

Figure S138. ^{1}H–^{15}N-HSQC-NMR spectrum of *N*-(8-hydroxy-5-oxo-1,2,3,5-tetrahydroindolizin-6-yl)hexanamide (**3a**).

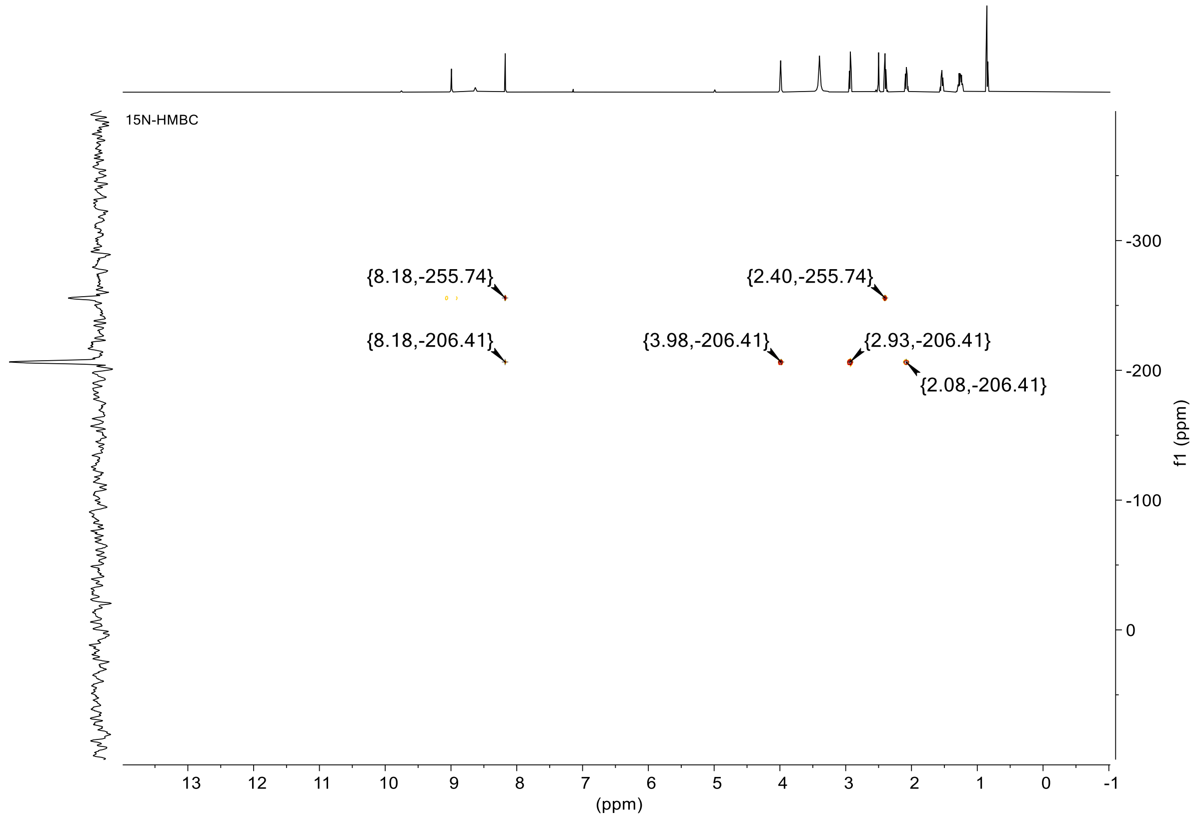

Figure S139. ^{1}H–^{15}N-HMBC-NMR spectrum of *N*-(8-hydroxy-5-oxo-1,2,3,5-tetrahydroindolizin-6-yl)hexanamide (**3a**).

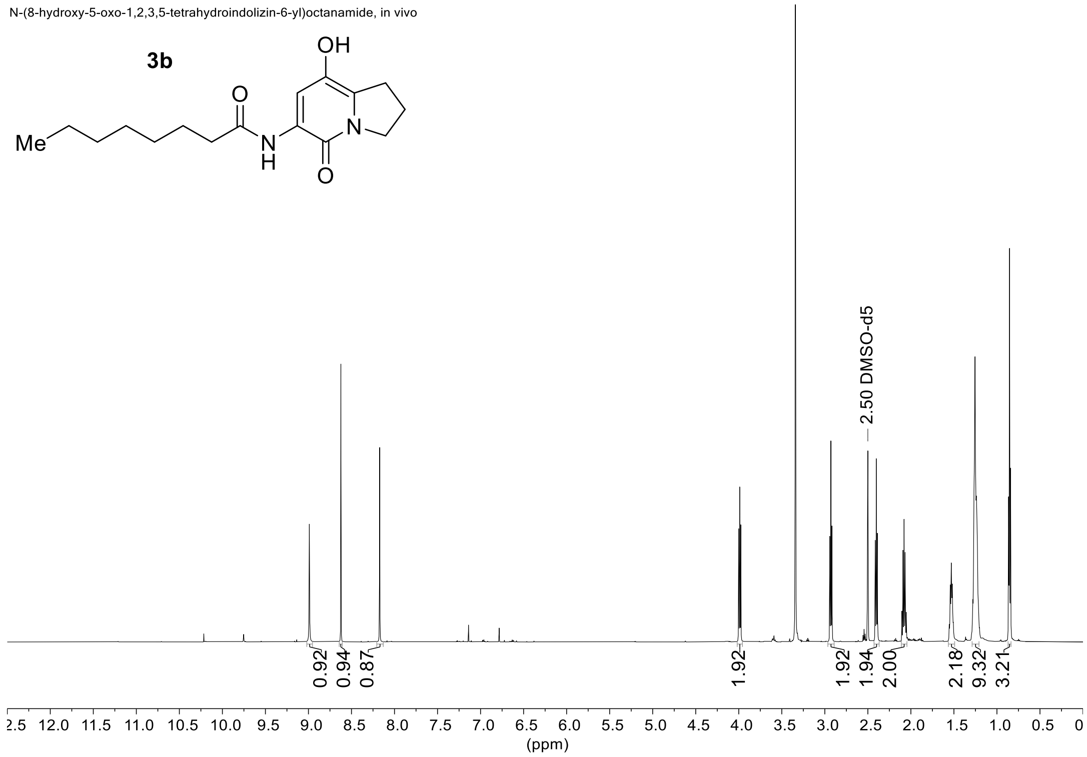

Figure S140. ^{1}H-NMR spectrum (600 MHz) of *N*-(8-hydroxy-5-oxo-1,2,3,5-tetrahydroindolizin-6-yl)octanamide (**3b**).

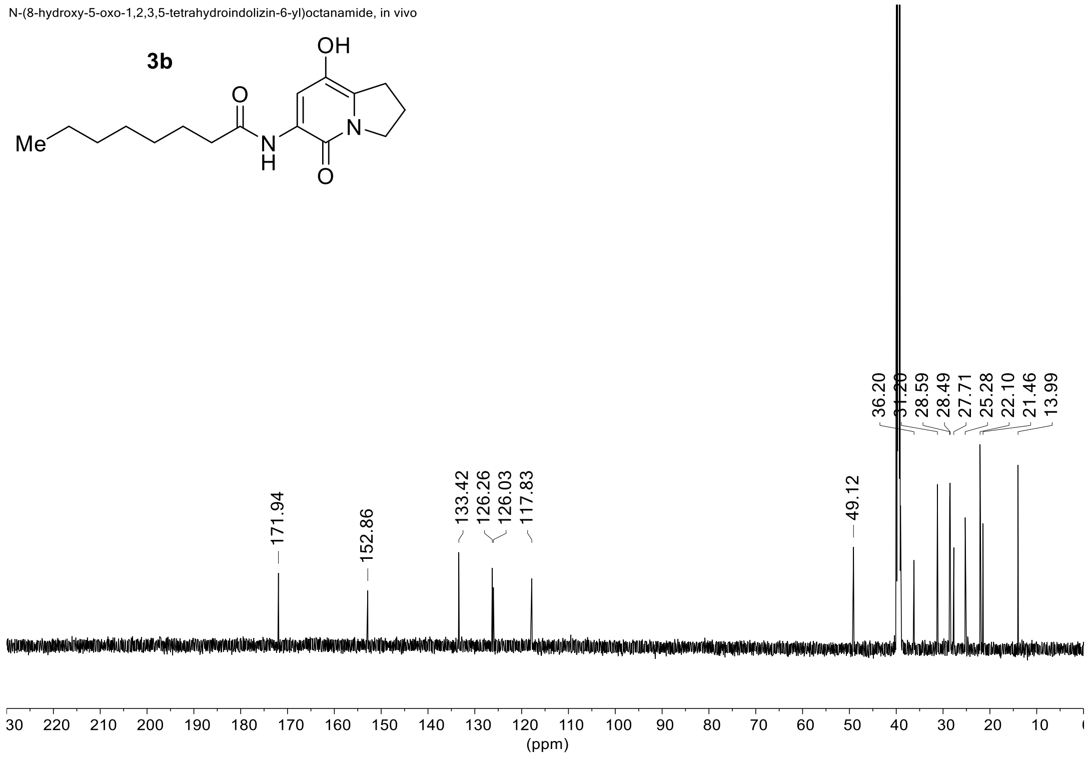

Figure S141. ^{13}C {^{1}H}-NMR spectrum (151 MHz) of *N*-(8-hydroxy-5-oxo-1,2,3,5-tetrahydroindolizin-6-yl)octanamide (**3b**).

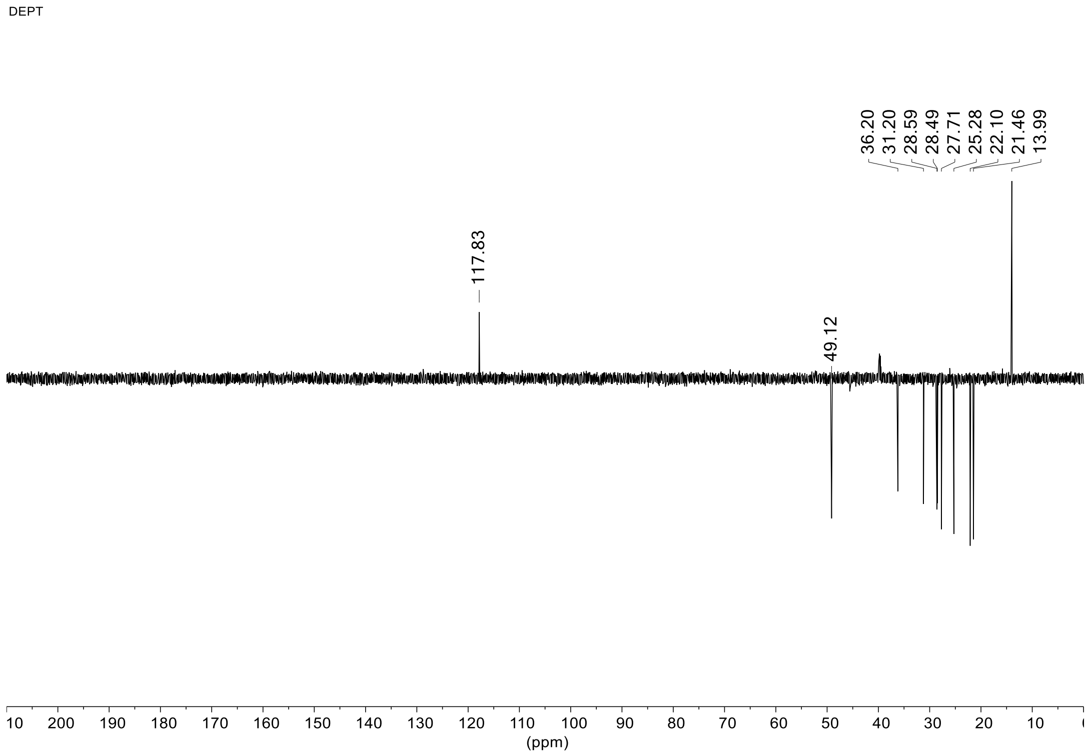

Figure S142. DEPT[135]-NMR spectrum of *N*-(8-hydroxy-5-oxo-1,2,3,5-tetrahydroindolizin-6-yl)octanamide (**3b**).

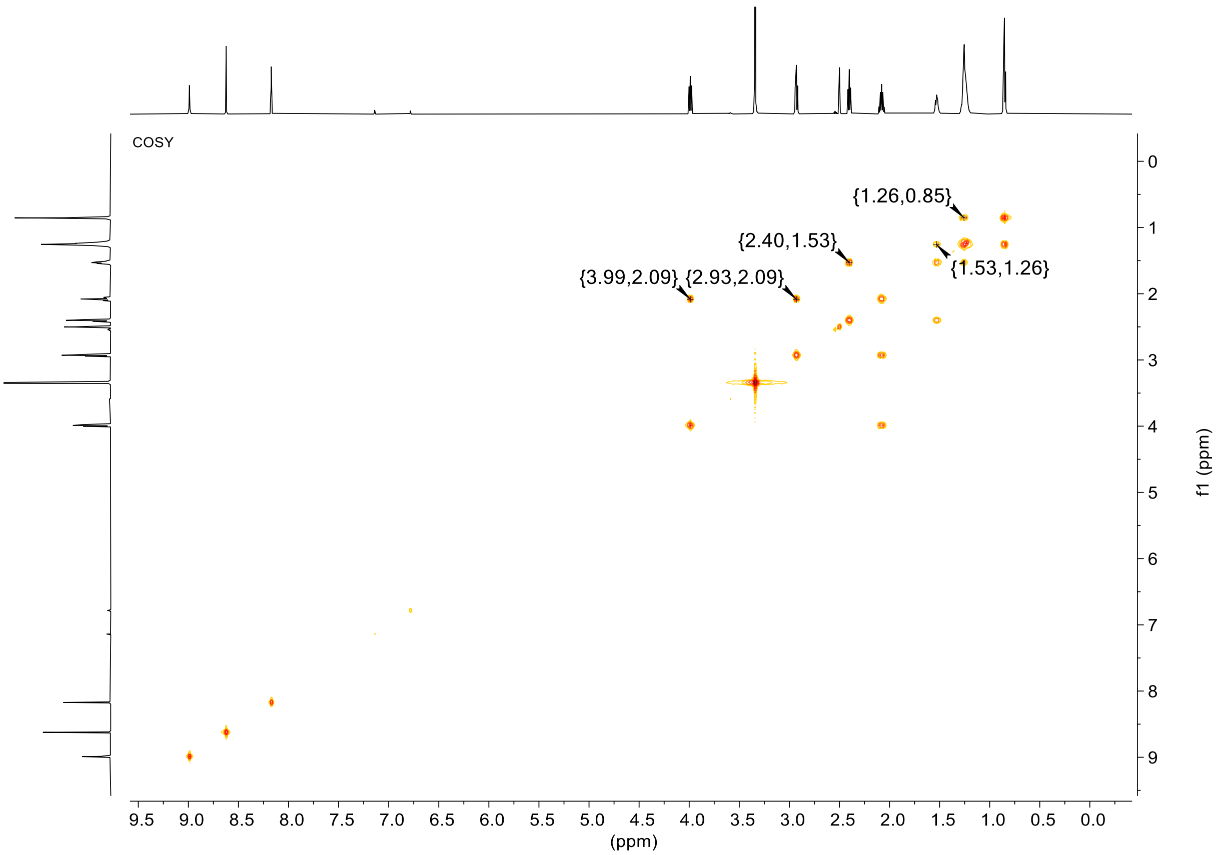

Figure S143. COSY-NMR spectrum of *N*-(8-hydroxy-5-oxo-1,2,3,5-tetrahydroindolizin-6-yl)octanamide (**3b**).

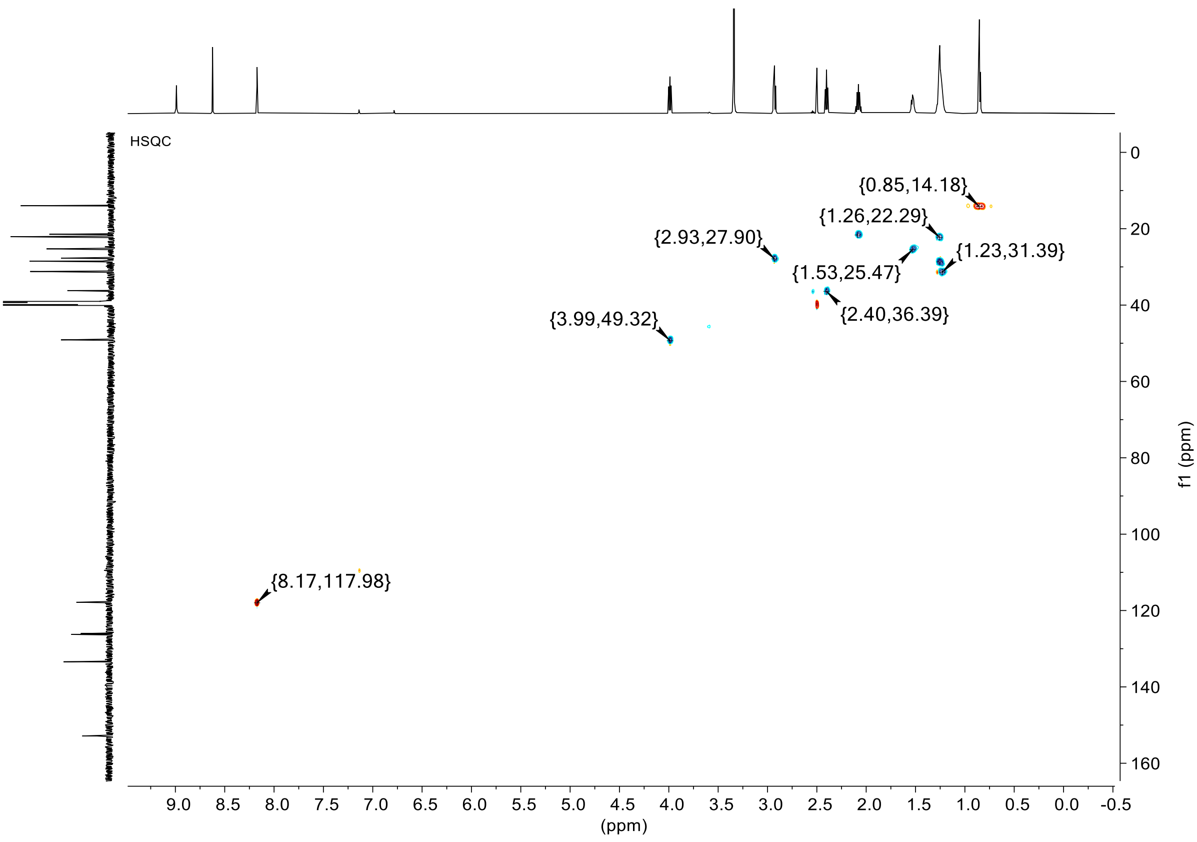

Figure S144. ^{1}H–^{13}C-HSQC-NMR spectrum of *N*-(8-hydroxy-5-oxo-1,2,3,5-tetrahydroindolizin-6-yl)octanamide (**3b**).

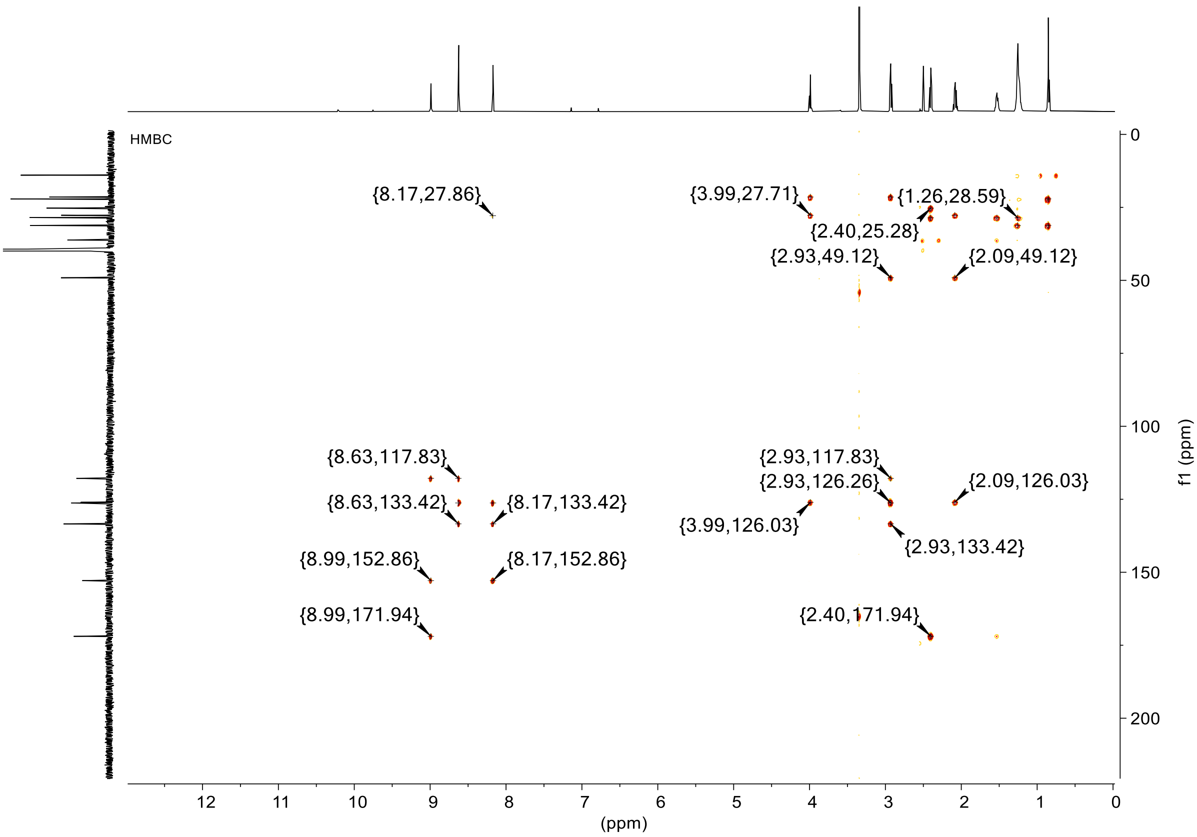

Figure S145. ^{1}H–^{13}C-HMBC-NMR spectrum of *N*-(8-hydroxy-5-oxo-1,2,3,5-tetrahydroindolizin-6-yl)octanamide (**3b**).

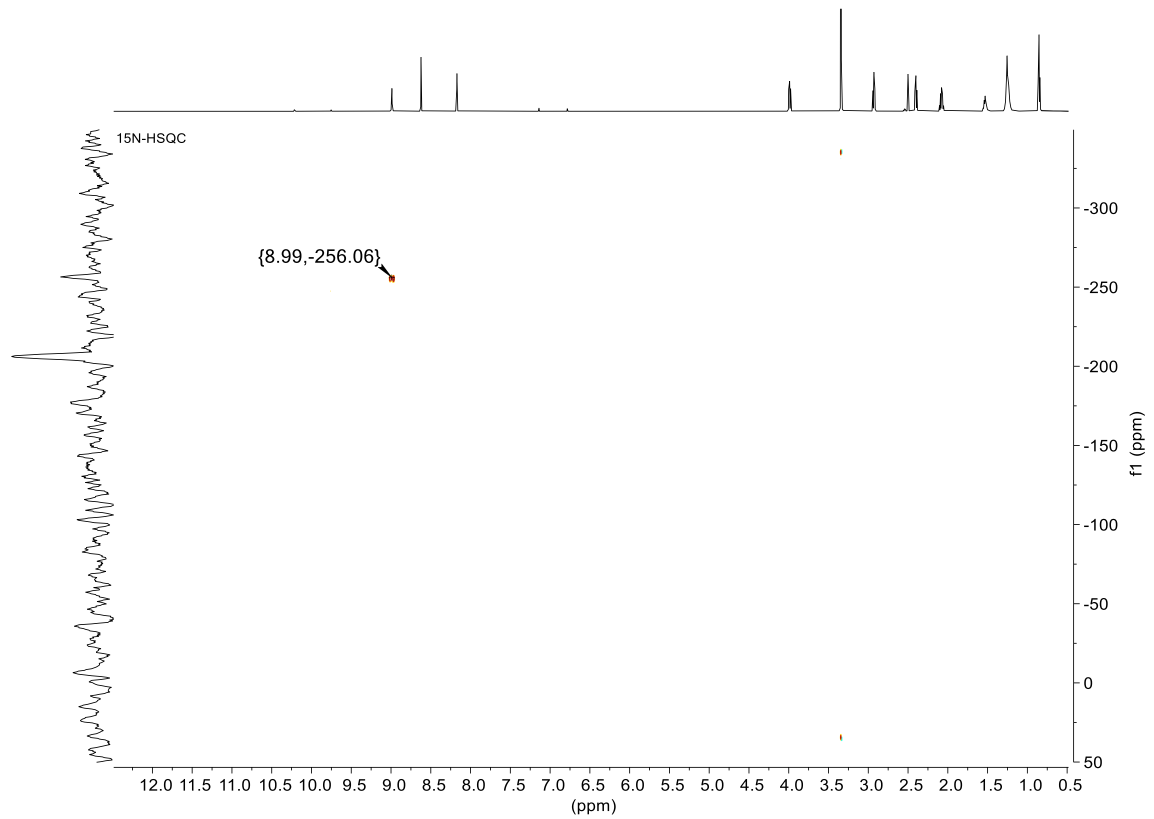

Figure S146. ^{1}H–^{15}N-HSQC-NMR spectrum of *N*-(8-hydroxy-5-oxo-1,2,3,5-tetrahydroindolizin-6-yl)octanamide (**3b**).

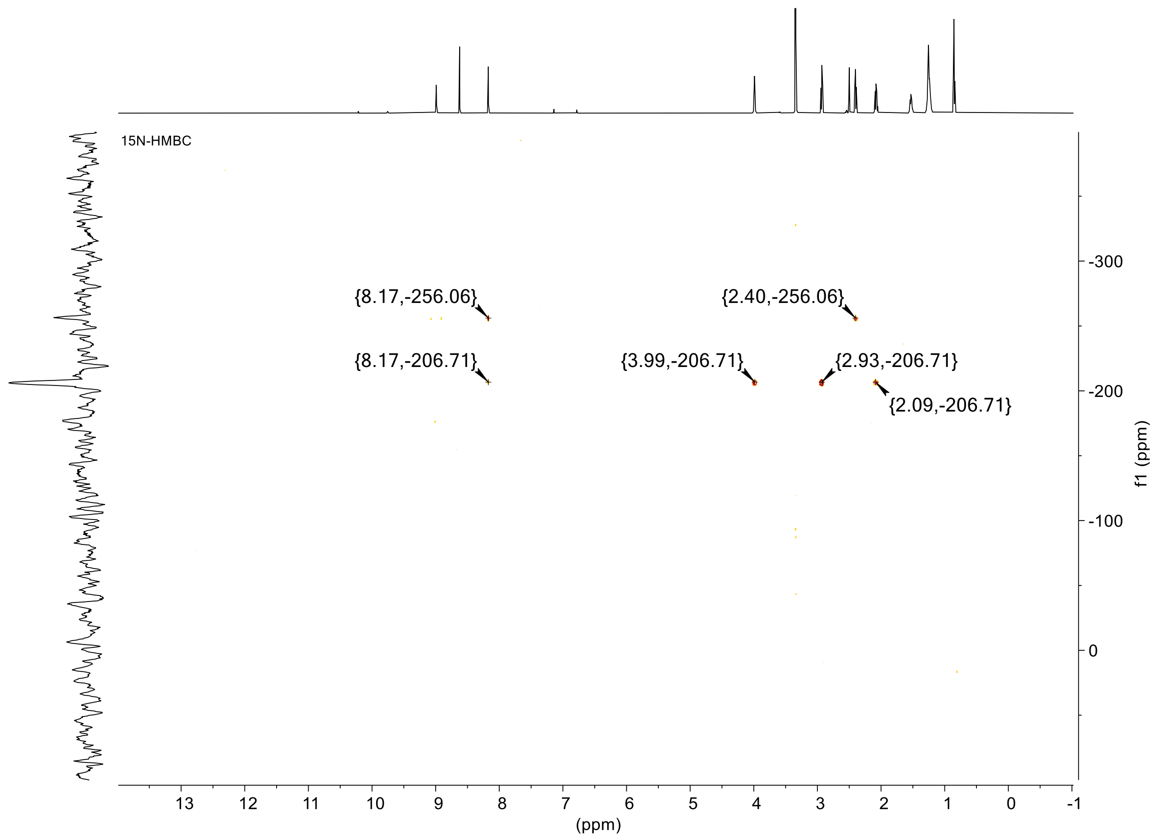

Figure S147. ^{1}H–^{15}N-HMBC-NMR spectrum of *N*-(8-hydroxy-5-oxo-1,2,3,5-tetrahydroindolizin-6-yl)octanamide (**3b**).

6.2 Synthetic substances

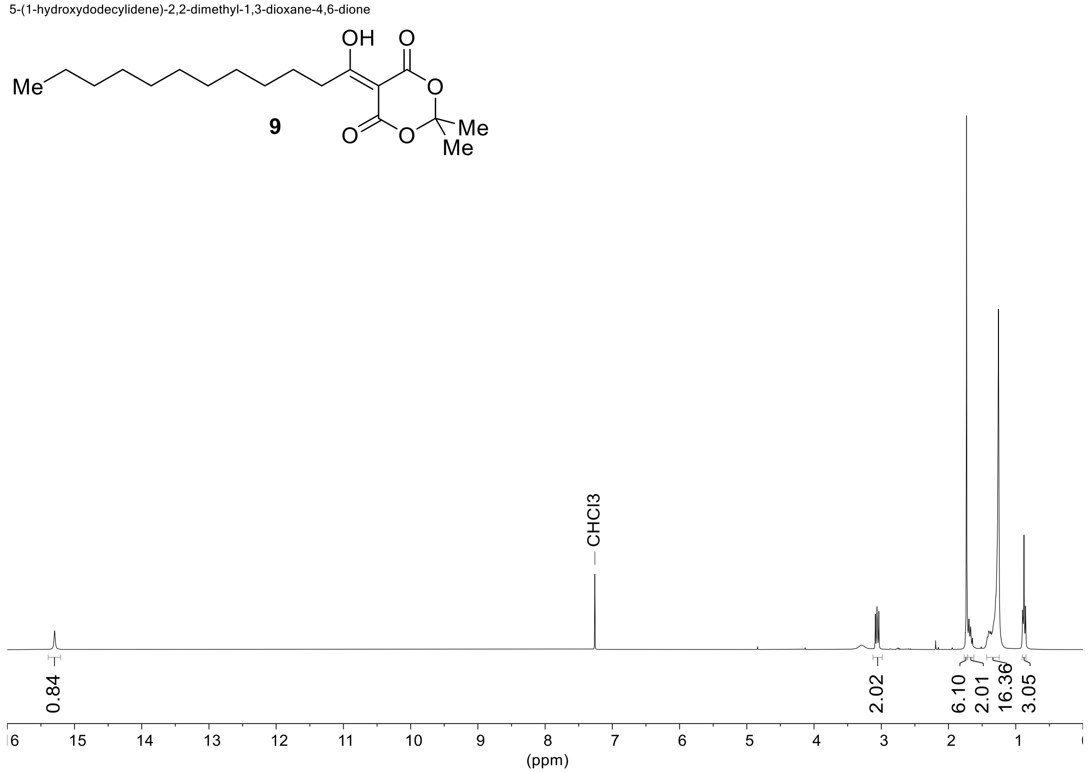

Figure S148. ^{1}H-NMR spectrum (300 MHz) of 5-(1-hydroxydodecylidene)-2,2-dimethyl-1,3-dioxane-4,6-dione (**9**).

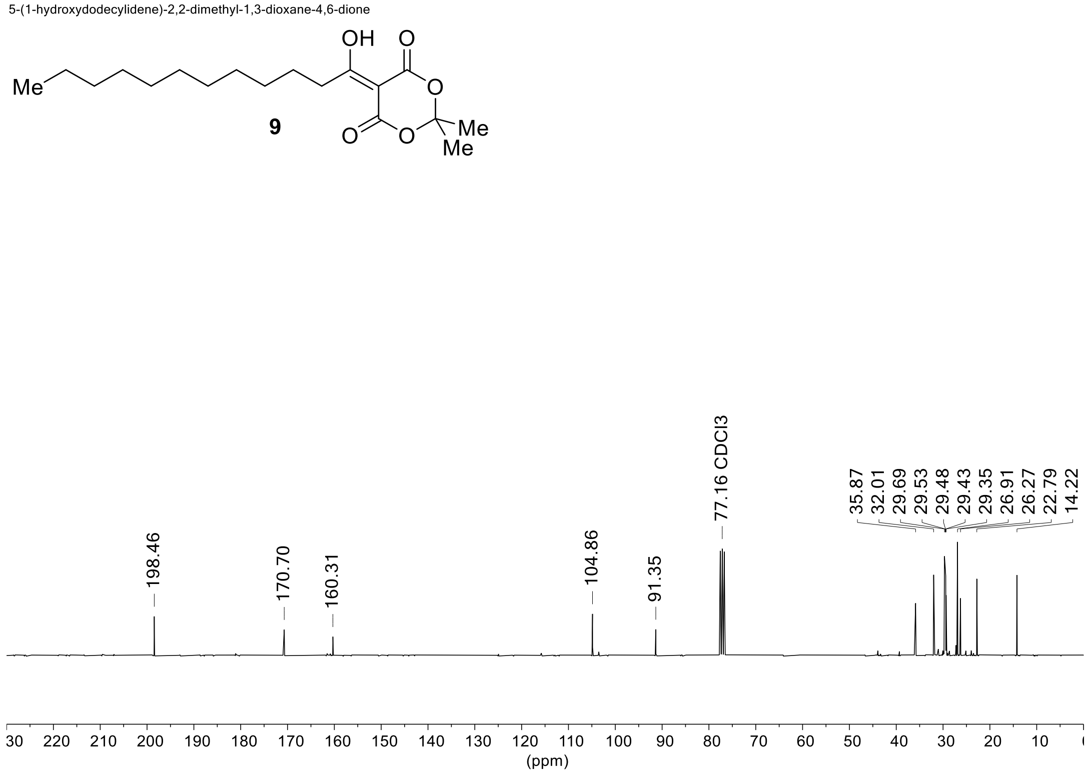

Figure S149. ^{13}C-NMR spectrum (75.5 MHz) of 5-(1-hydroxydodecylidene)-2,2-dimethyl-1,3-dioxane-4,6-dione (**9**).

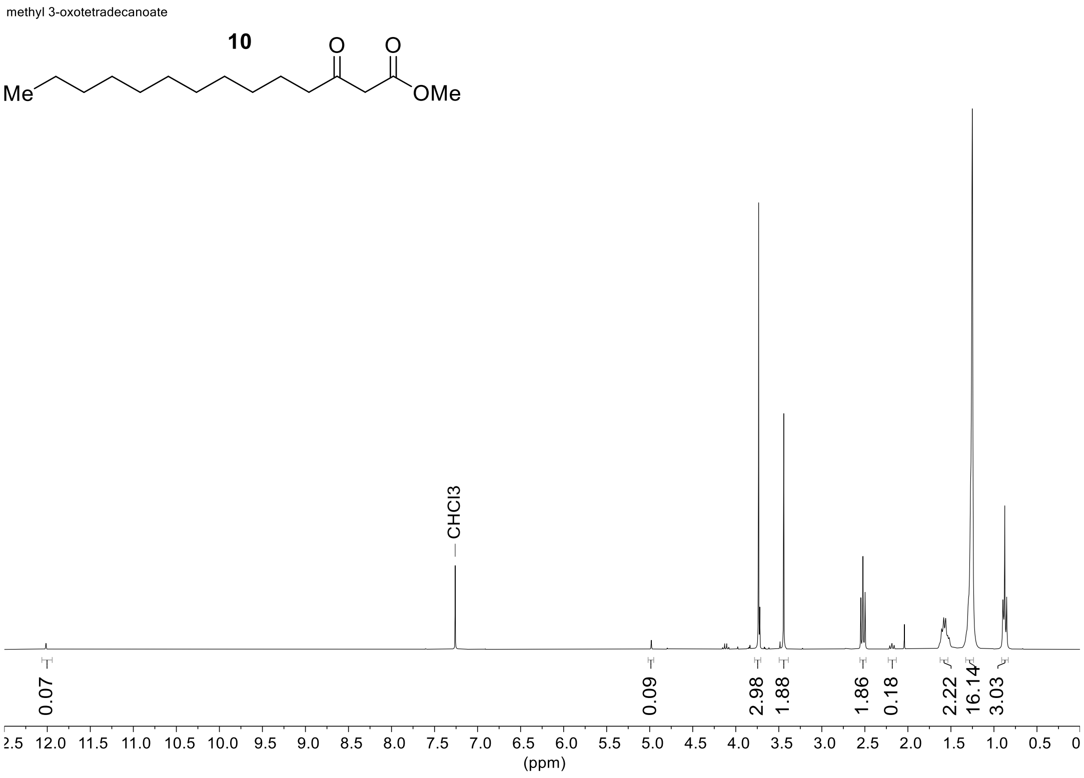

Figure S150. ^{1}H-NMR spectrum (300 MHz) of methyl 3-oxotetradecanoate (**10**).

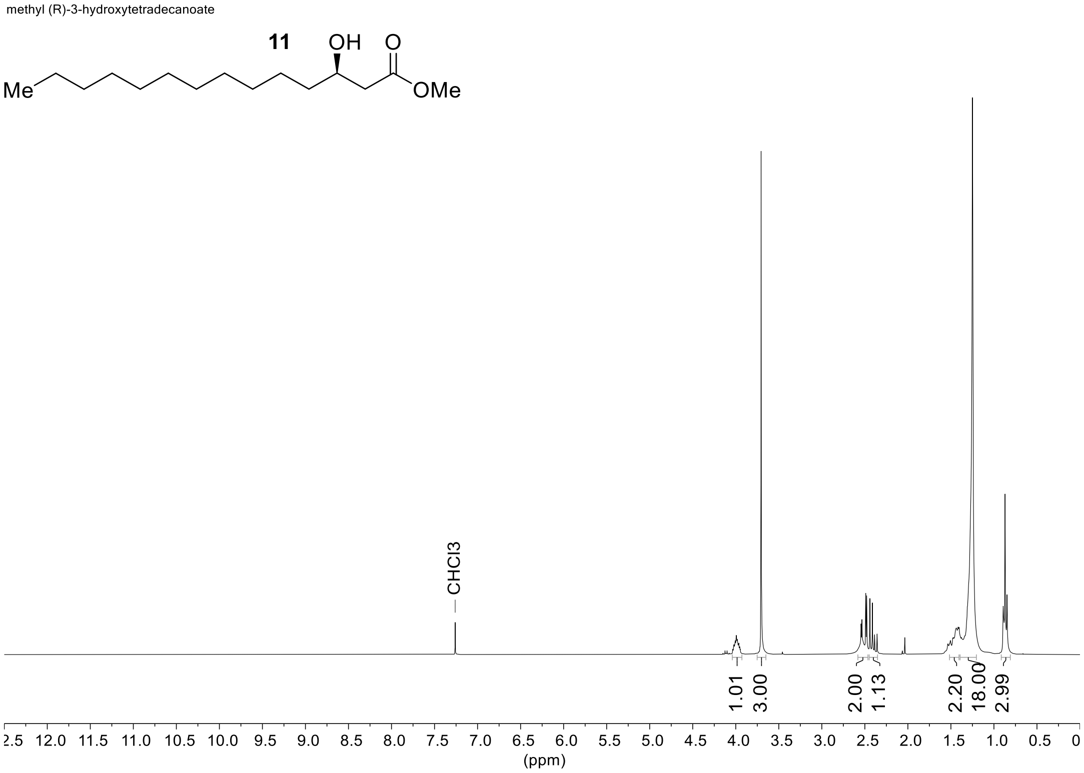

Figure S151. ^{1}H-NMR spectrum (300 MHz) of methyl (*R*)-3-hydroxytetradecanoate (**11**).

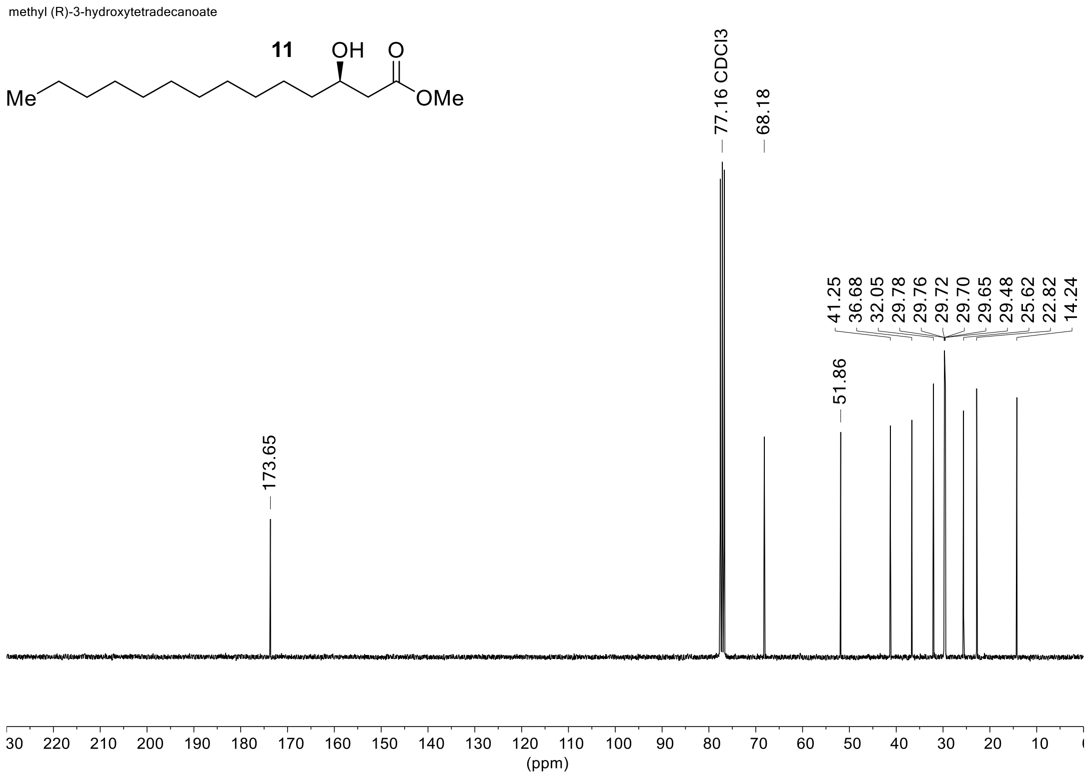

Figure S152. $^{13}C\ \{^{1}H\}$-NMR spectrum (75.5 MHz) of methyl (*R*)-3-hydroxytetradecanoate (**11**).

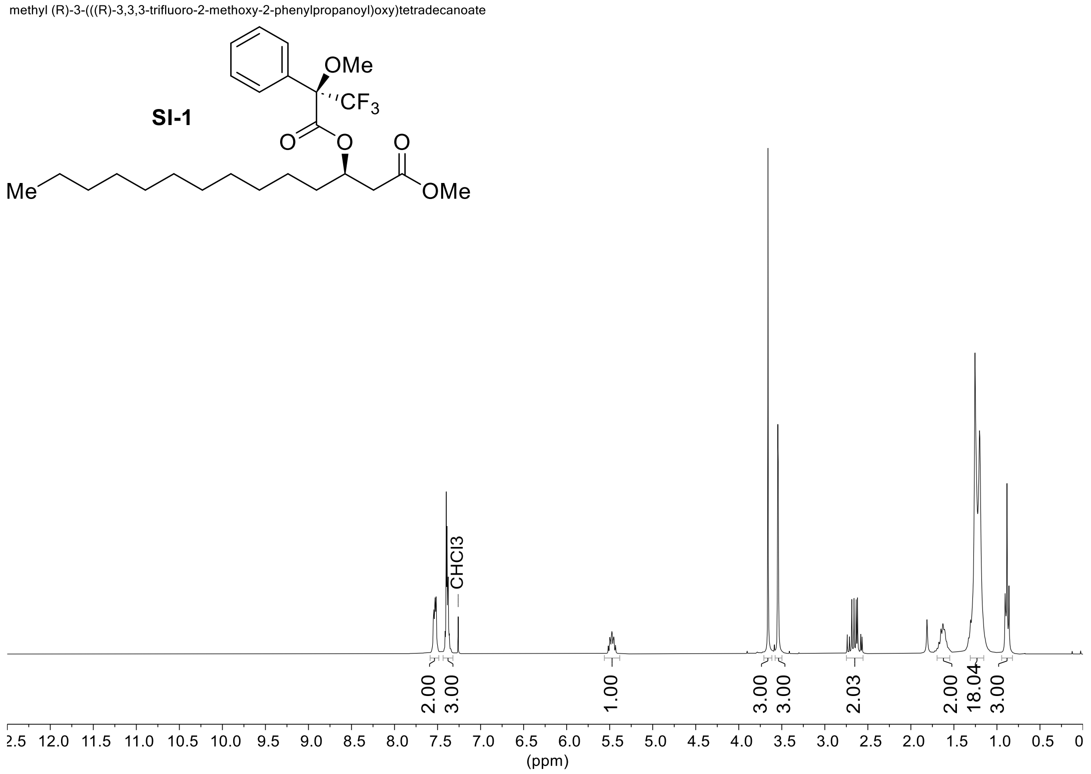

Figure S153. ^{1}H-NMR spectrum (300 MHz) of methyl (*R*)-3-(((*R*)-3,3,3-trifluoro-2-methoxy-2-phenylpropanoyl)oxy)tetradecanoate (**SI-1**).

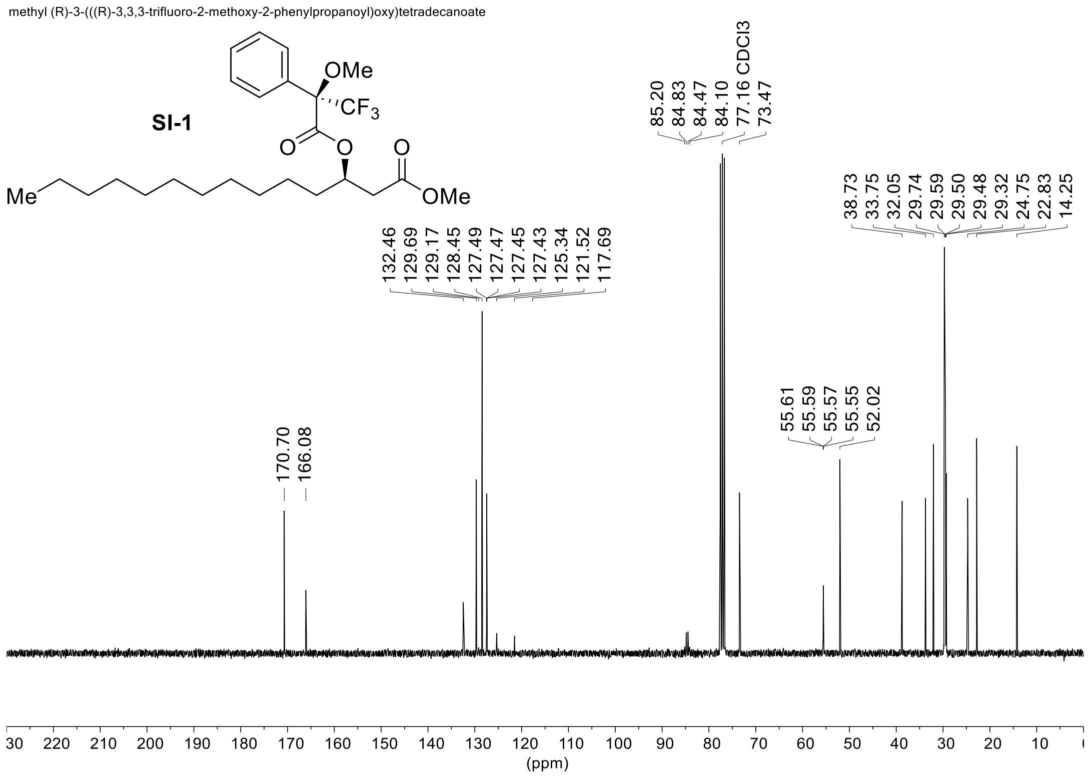

Figure S154. ^{13}C {^{1}H}-NMR spectrum (75.5 MHz) of methyl (*R*)-3-(((*R*)-3,3,3-trifluoro-2-methoxy-2-phenylpropanoyl)oxy)tetradecanoate (**SI-1**).

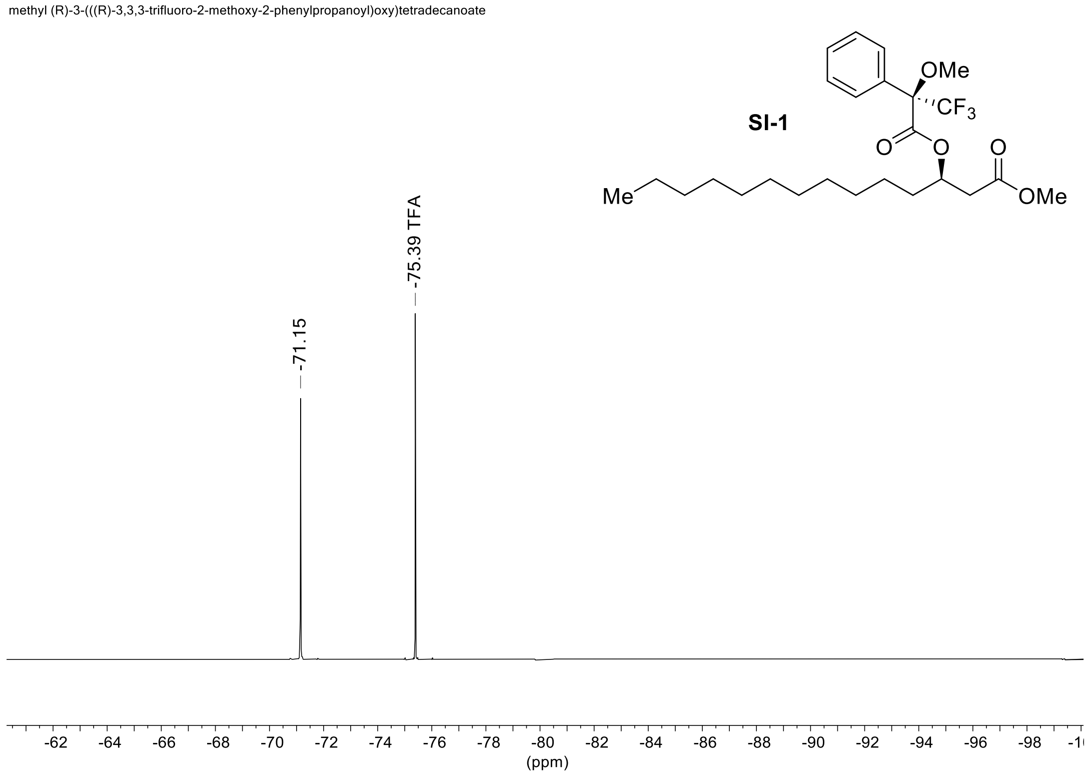

Figure S155. ^{19}F {^{1}H}-NMR spectrum (282 MHz) of methyl (*R*)-3-(((*R*)-3,3,3-trifluoro-2-methoxy-2-phenylpropanoyl)oxy)tetradecanoate (**SI-1**).

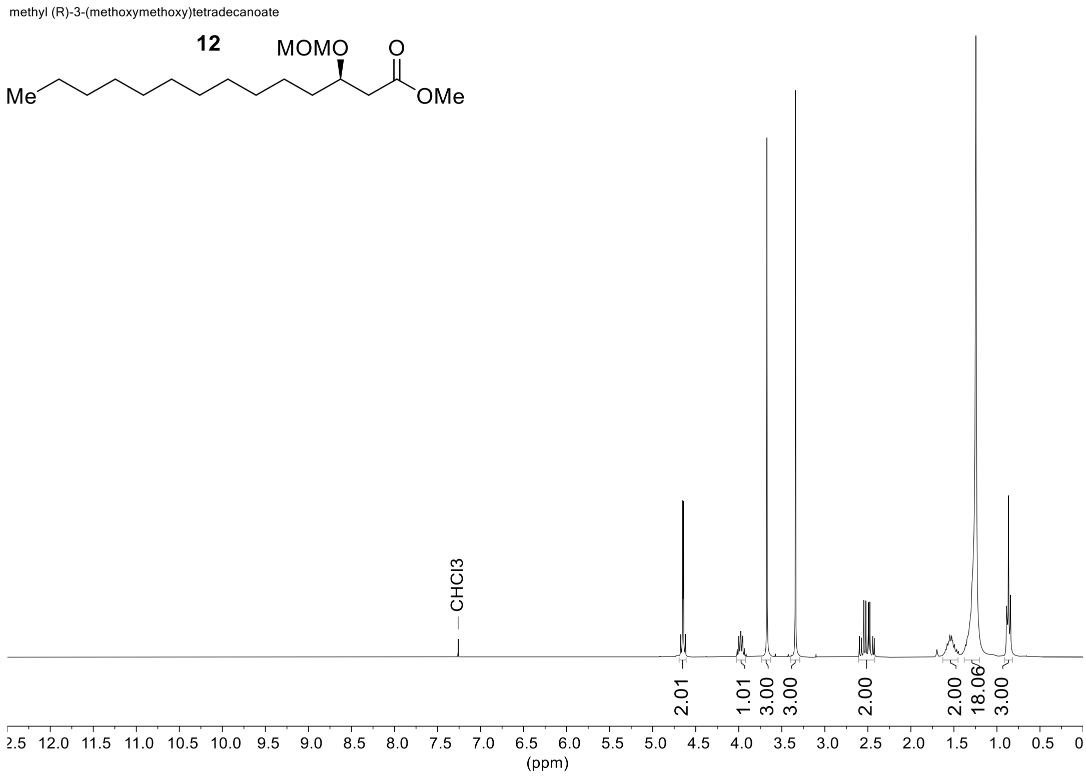

Figure S156. ^{1}H-NMR spectrum (300 MHz) of methyl (*R*)-3-(methoxymethoxy)tetradecanoate (**12**).

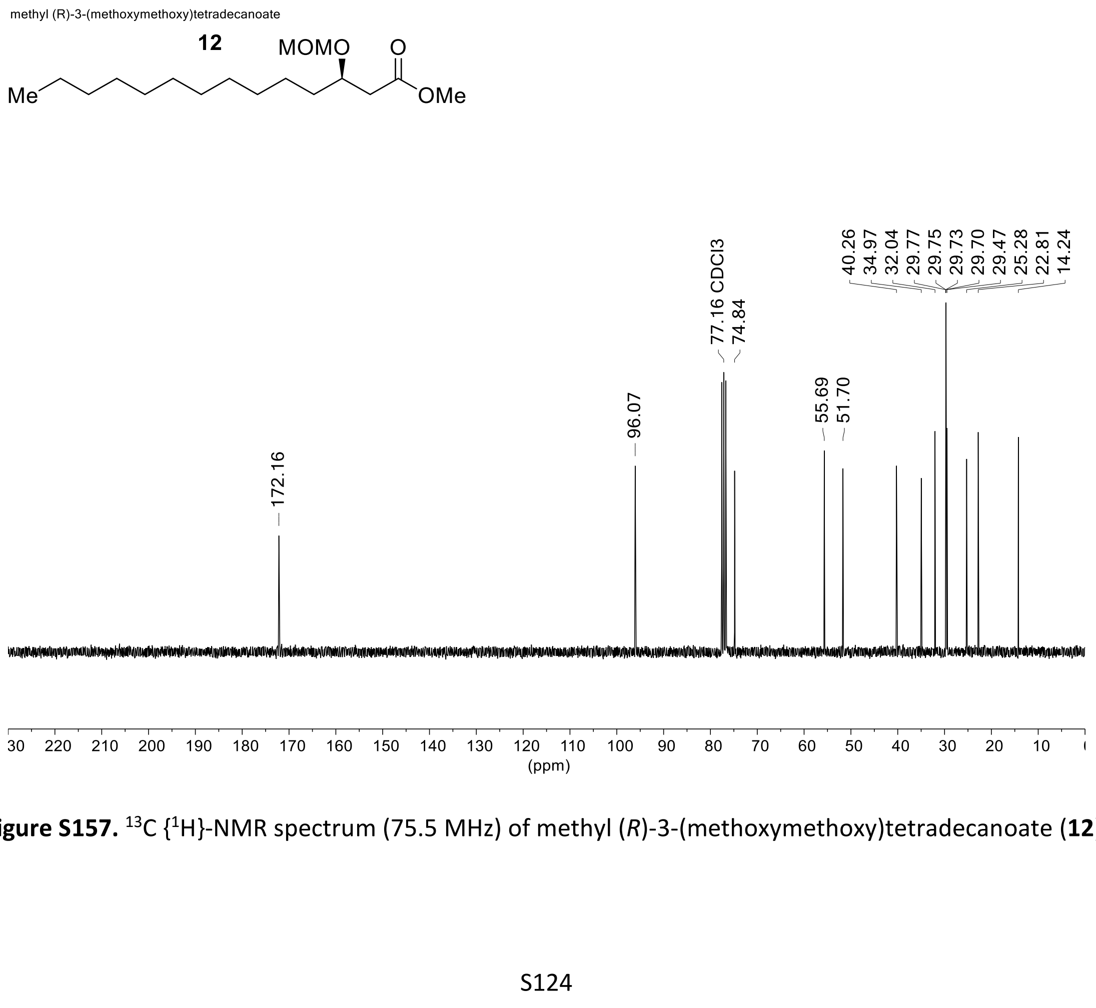

Figure S157. ^{13}C {^{1}H}-NMR spectrum (75.5 MHz) of methyl (*R*)-3-(methoxymethoxy)tetradecanoate (**12**).

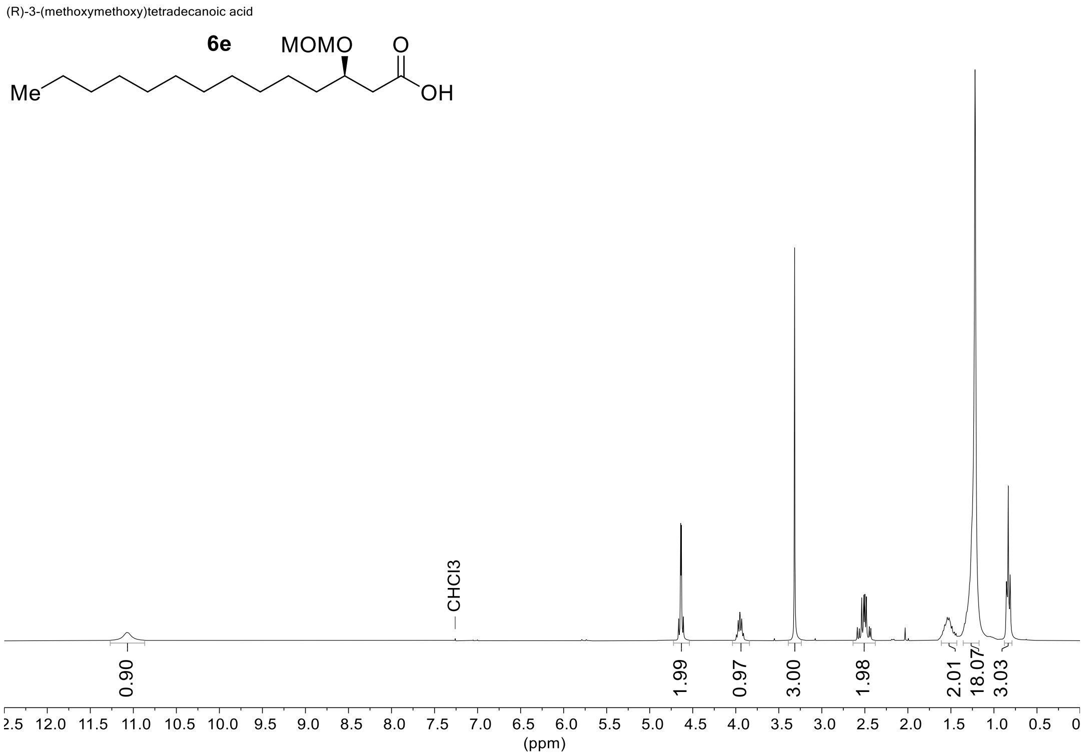

Figure S158. ^{1}H-NMR spectrum (300 MHz) of (*R*)-3-(methoxymethoxy)tetradecanoic acid (**6e**).

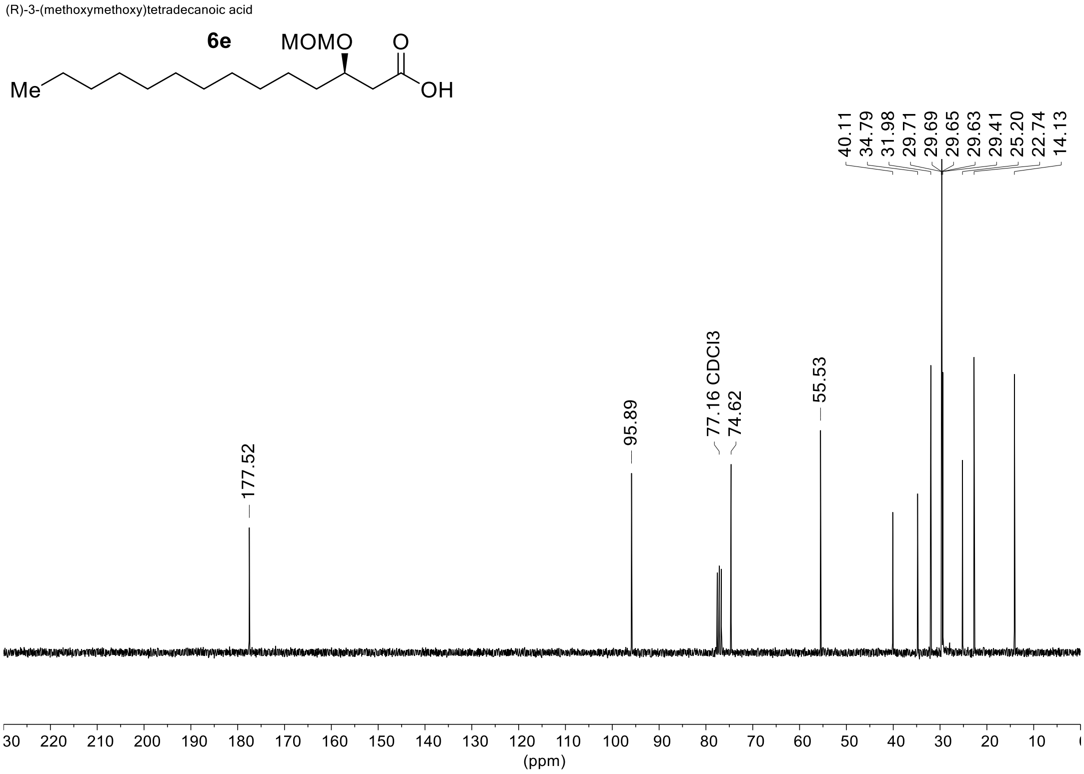

Figure S159. ^{13}C {^{1}H}-NMR spectrum (75.5 MHz) of (*R*)-3-(methoxymethoxy)tetradecanoic acid (**6e**).

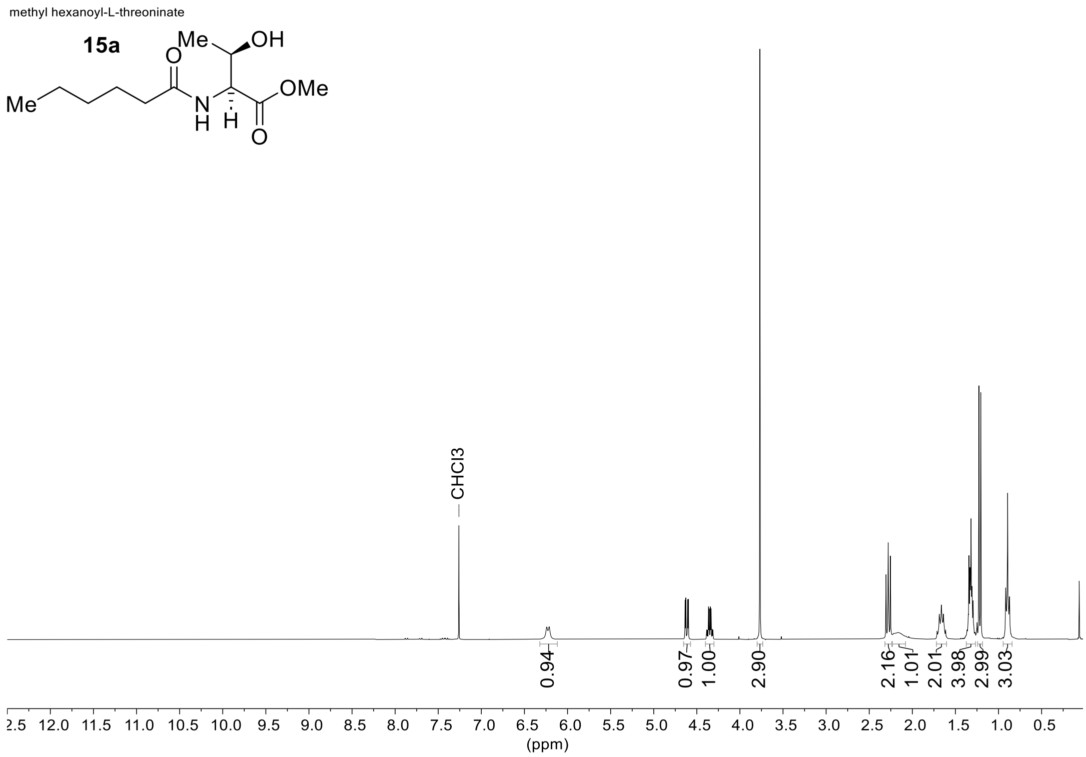

Figure S160. ^{1}H-NMR spectrum (300 MHz) of methyl hexanoyl-L-threoninate (**15a**).

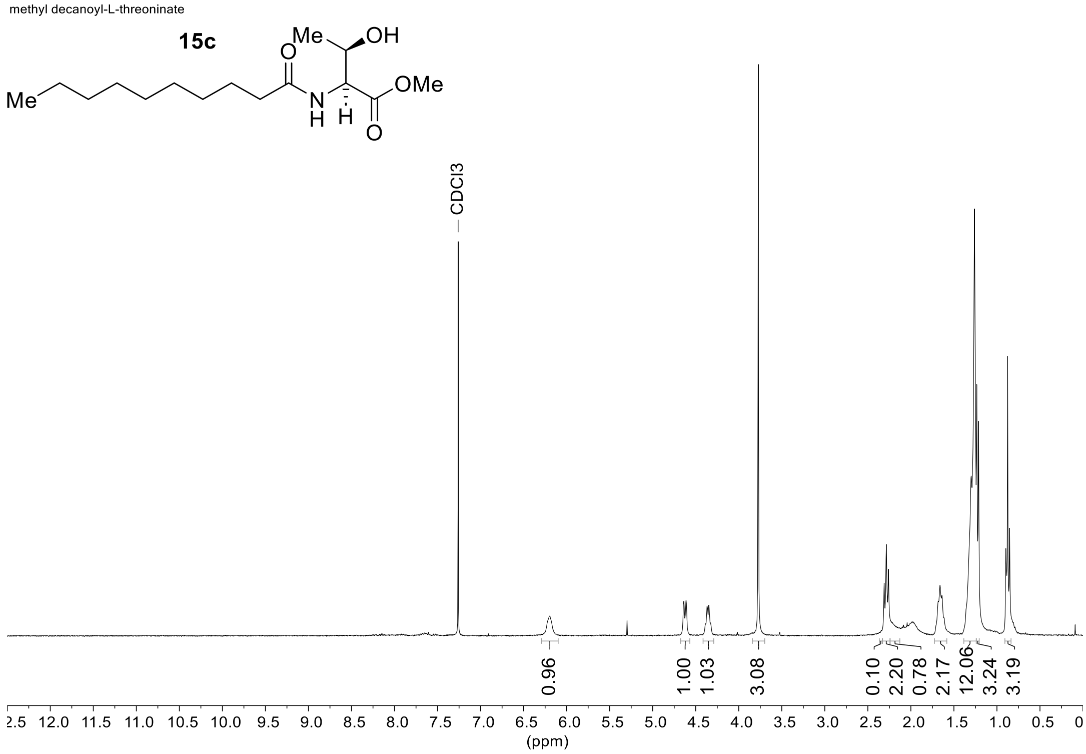

Figure S161. ^{1}H-NMR spectrum (300 MHz) of methyl decanoyl-L-threoninate (**15c**).

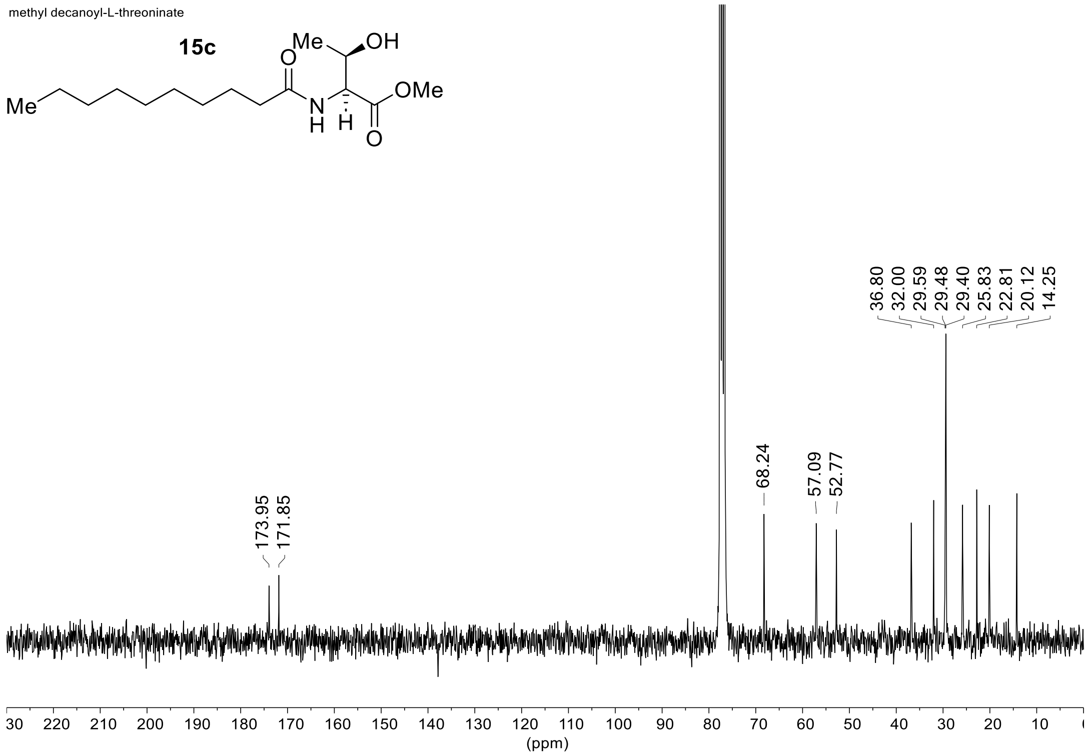

Figure S162. $^{13}C\{^1H\}$-NMR spectrum (75.5 MHz) of methyl decanoyl-L-threoninate (**15c**).

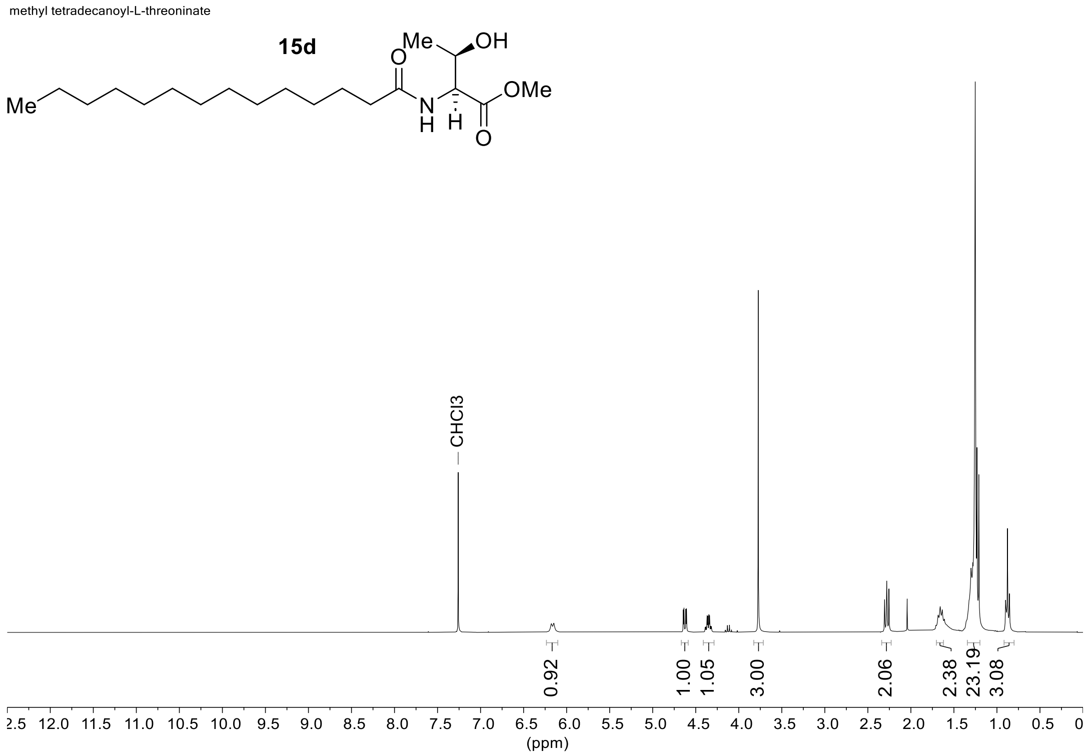

Figure S163. ^1H-NMR spectrum (300 MHz) of methyl tetradecanoyl-L-threoninate (**15d**).

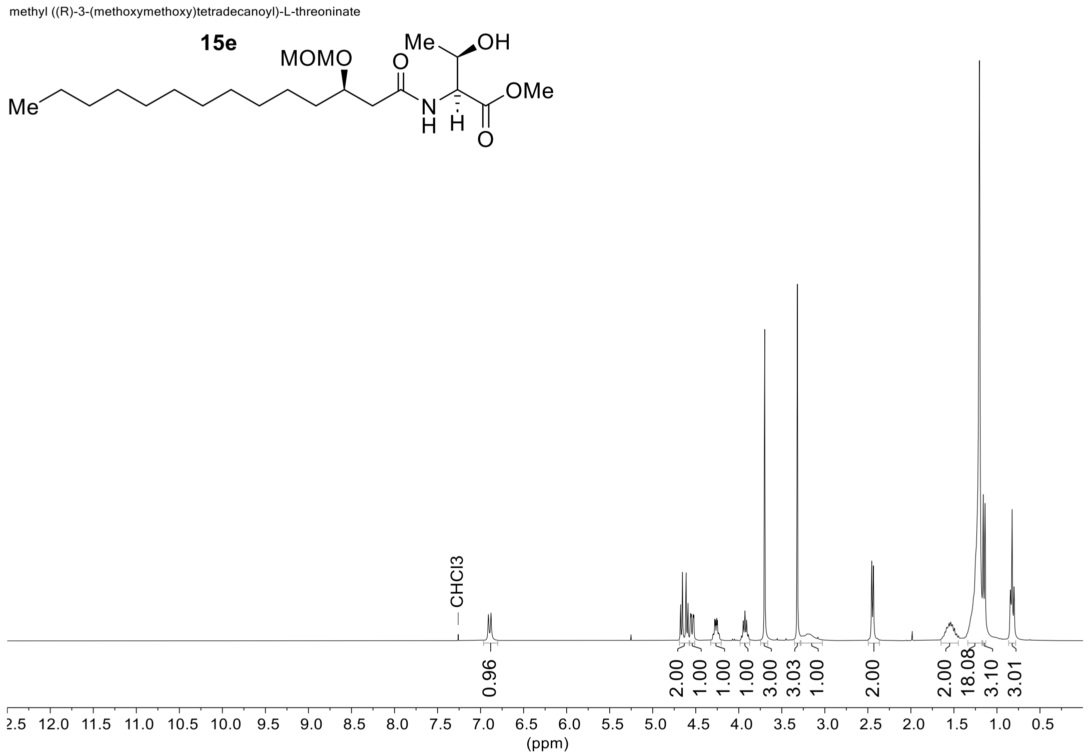

Figure S164. ^{1}H-NMR spectrum (300 MHz) of methyl ((*R*)-3-(methoxymethoxy)tetradecanoyl-L-threoninate (**15e**).

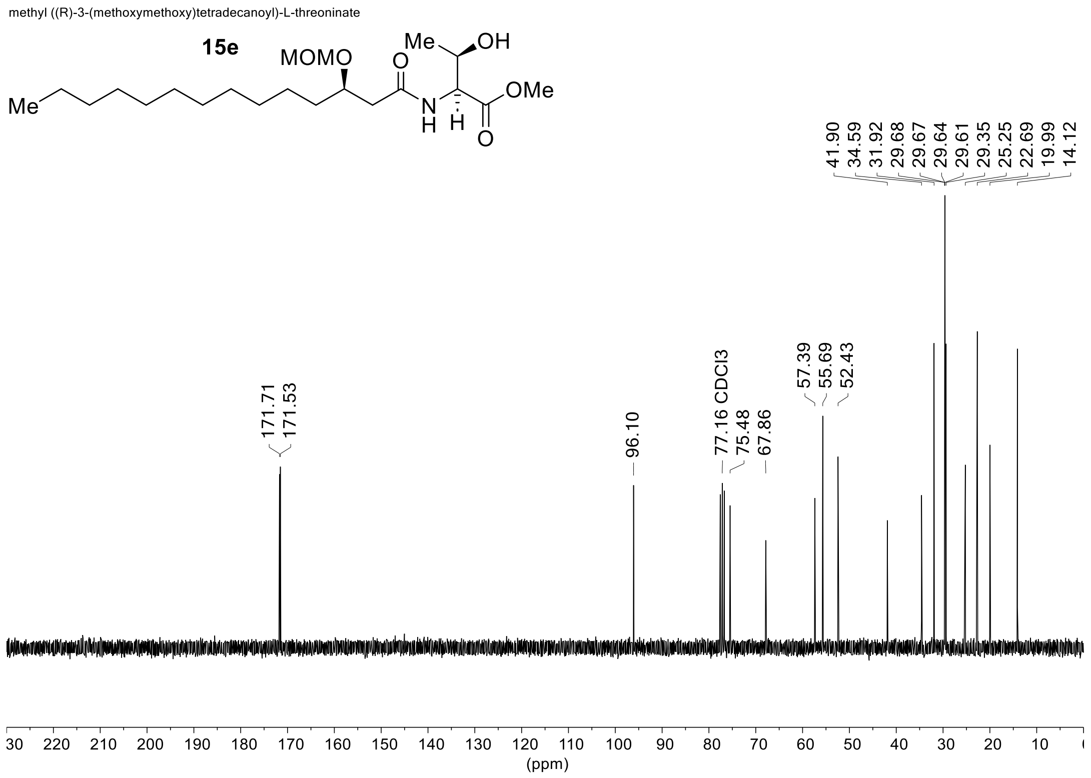

Figure S165. ^{13}C {^{1}H}-NMR spectrum of methyl ((*R*)-3-(methoxymethoxy)tetradecanoyl-L-threoninate (**15e**).

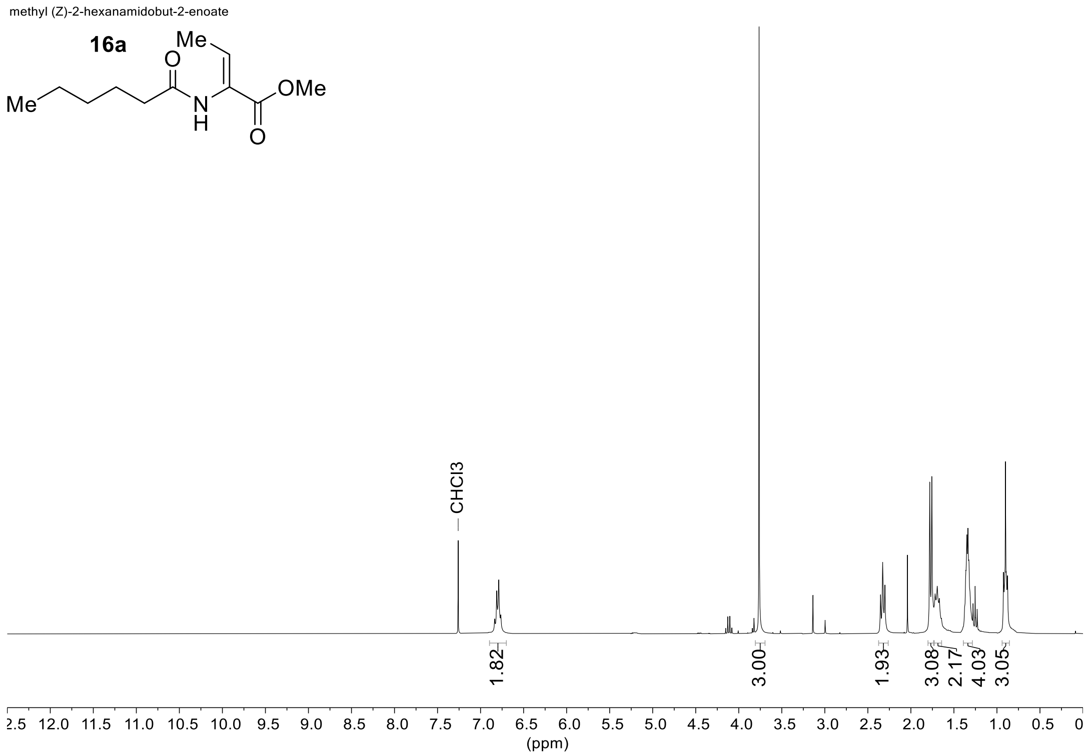

Figure S166. ^{1}H-NMR spectrum (300 MHz) of methyl (*Z*)-2-hexanamidobut-2-enoate (**16a**).

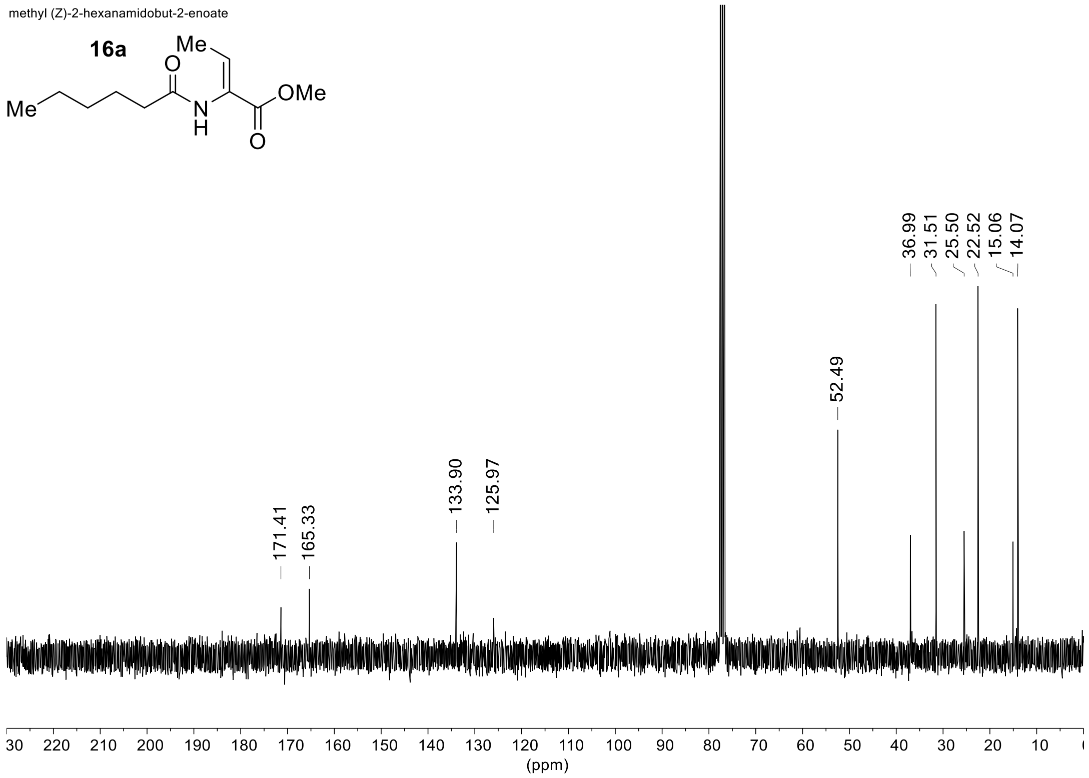

Figure S167. ^{13}C {^{1}H}-NMR spectrum (75.5 MHz) of methyl (*Z*)-2-hexanamidobut-2-enoate (**16a**).

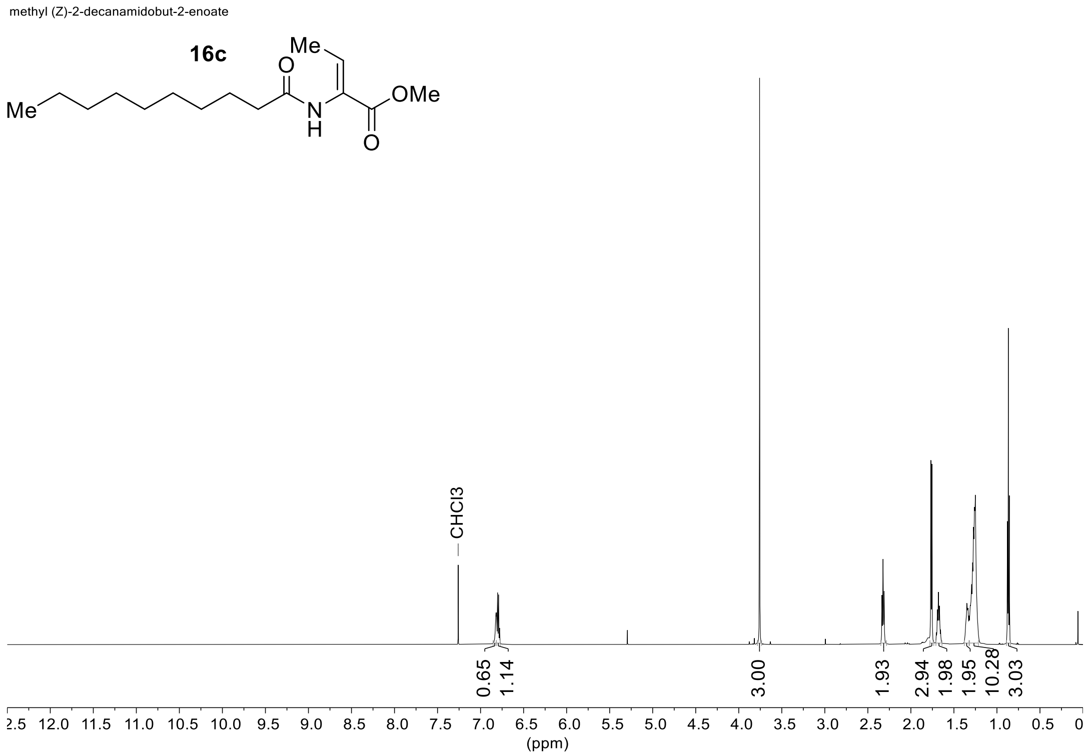

Figure S168. ^{1}H-NMR spectrum (600 MHz) of methyl (*Z*)-2-decanamidobut-2-enoate (**16c**).

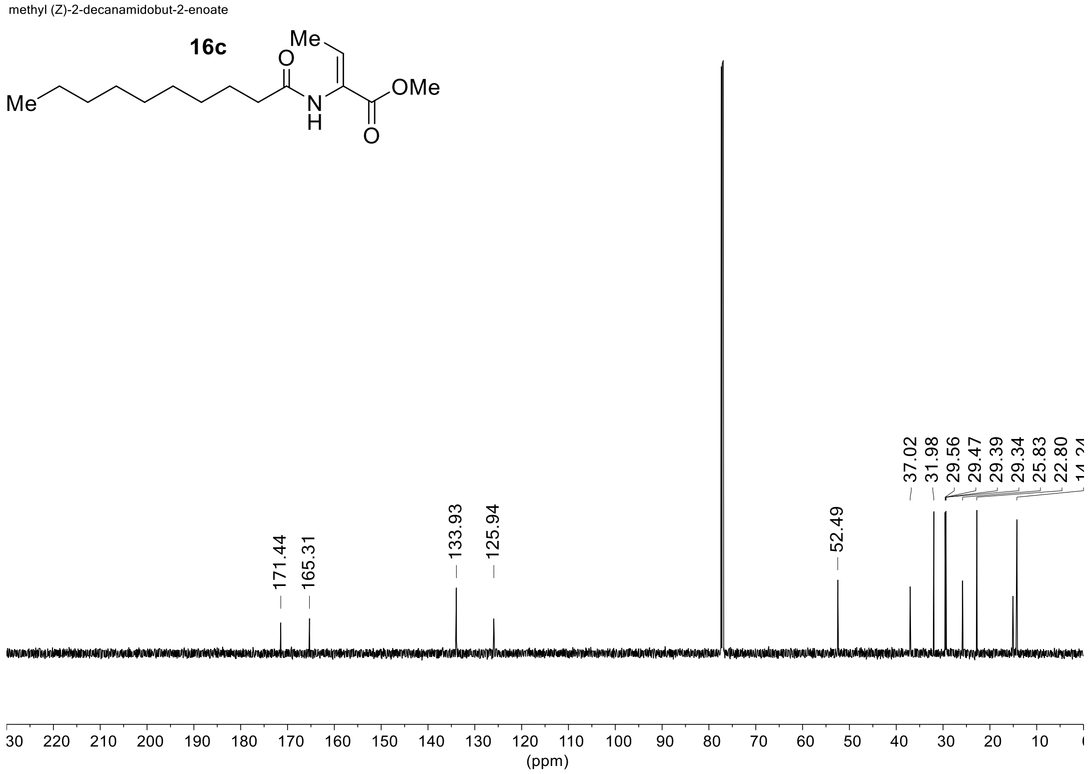

Figure S169. ^{13}C {^{1}H}-NMR spectrum (151 MHz) of methyl (*Z*)-2-decanamidobut-2-enoate (**16c**).

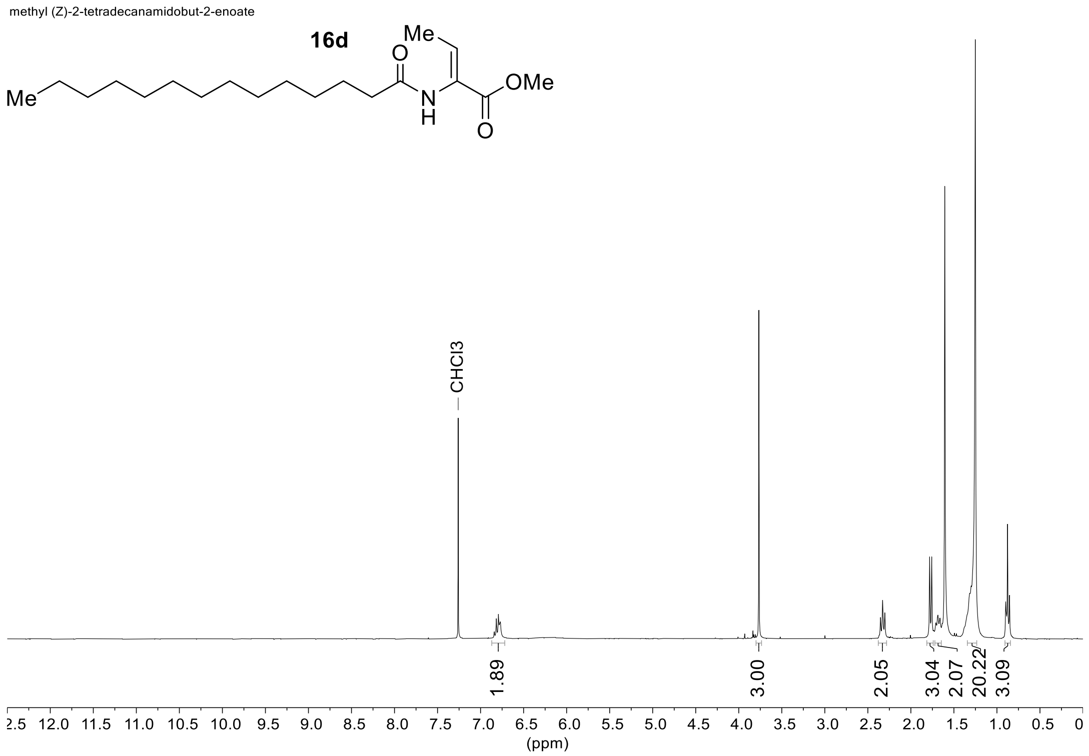

Figure S170. ^{1}H-NMR spectrum (300 MHz) of methyl (*Z*)-2-tetradecanamidobut-2-enoate (**16d**).

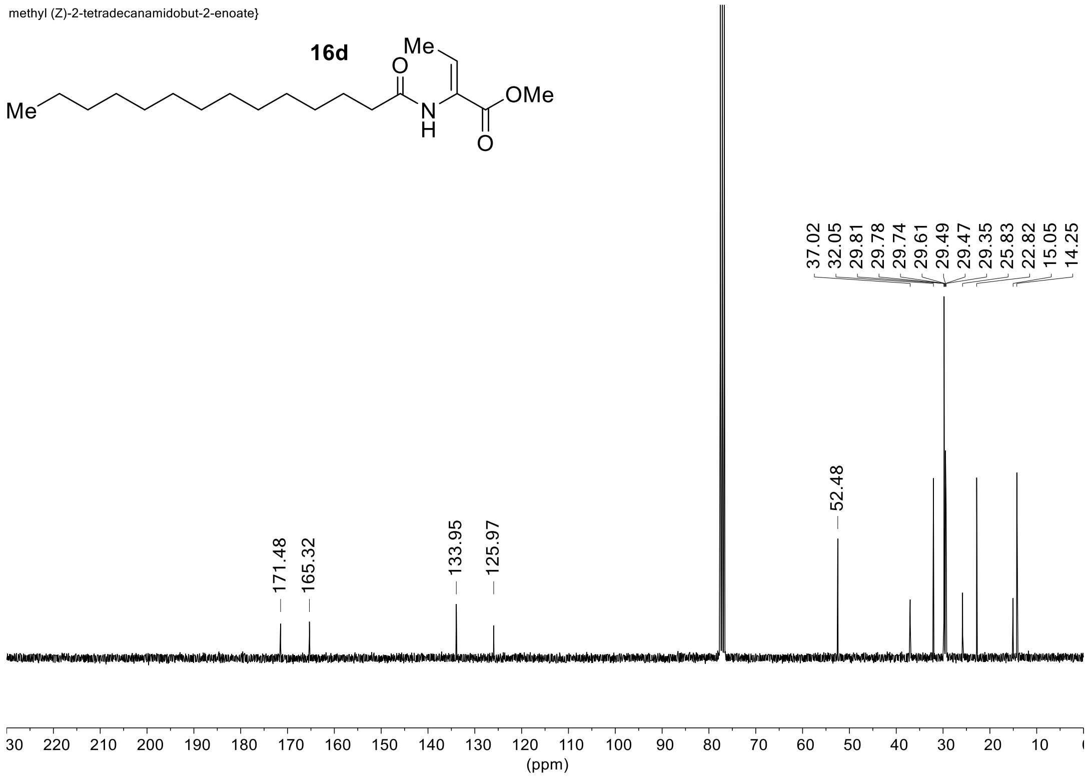

Figure S171. ^{13}C {^{1}H}-NMR spectrum (75.5 MHz) of methyl (*Z*)-2-tetradecanamidobut-2-enoate (**16d**).

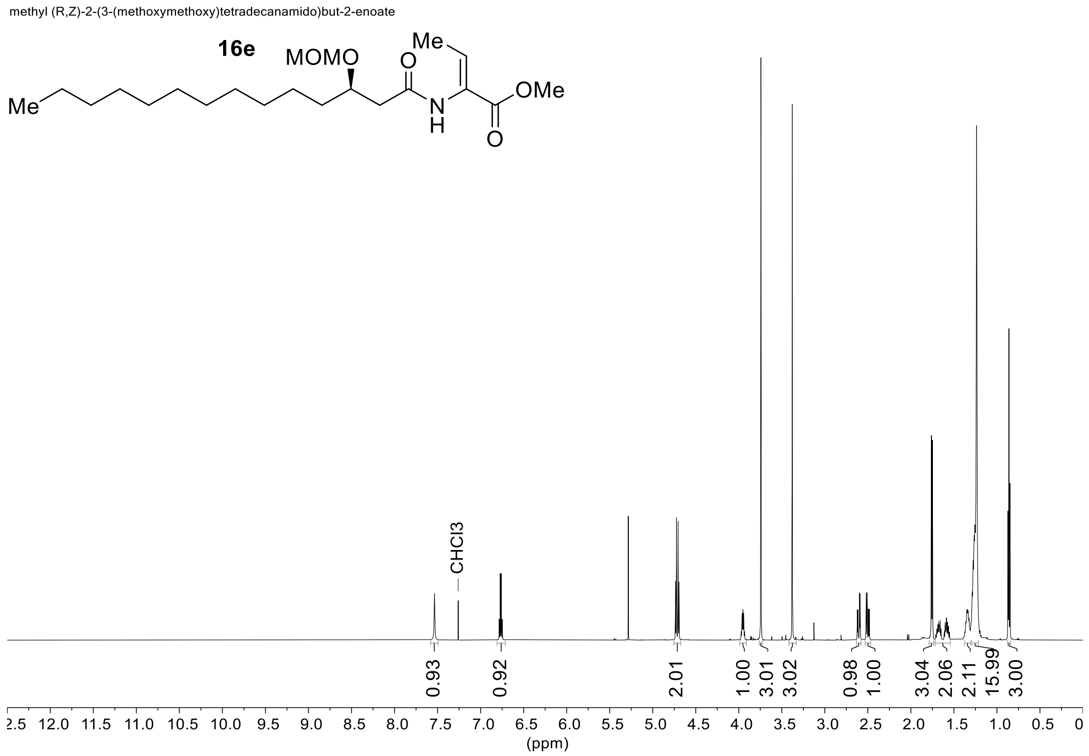

Figure S172. ^{1}H-NMR spectrum (600 MHz) of methyl (*R*,*Z*)-2-(3-(methoxymethoxy)tetradecanamido)but-2-enoate (**16e**).

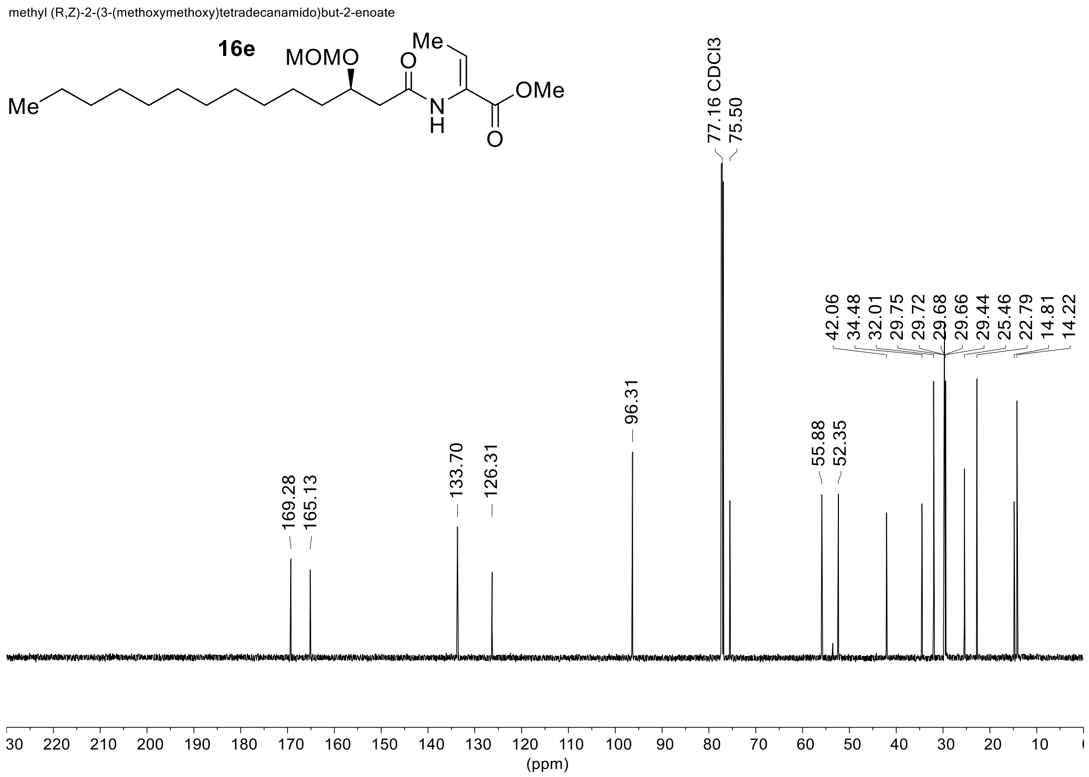

Figure S173. ^{13}C {^{1}H}-NMR spectrum (151 MHz) of methyl (*R*,*Z*)-2-(3-(methoxymethoxy)tetradecanamido)but-2-enoate (**16e**).

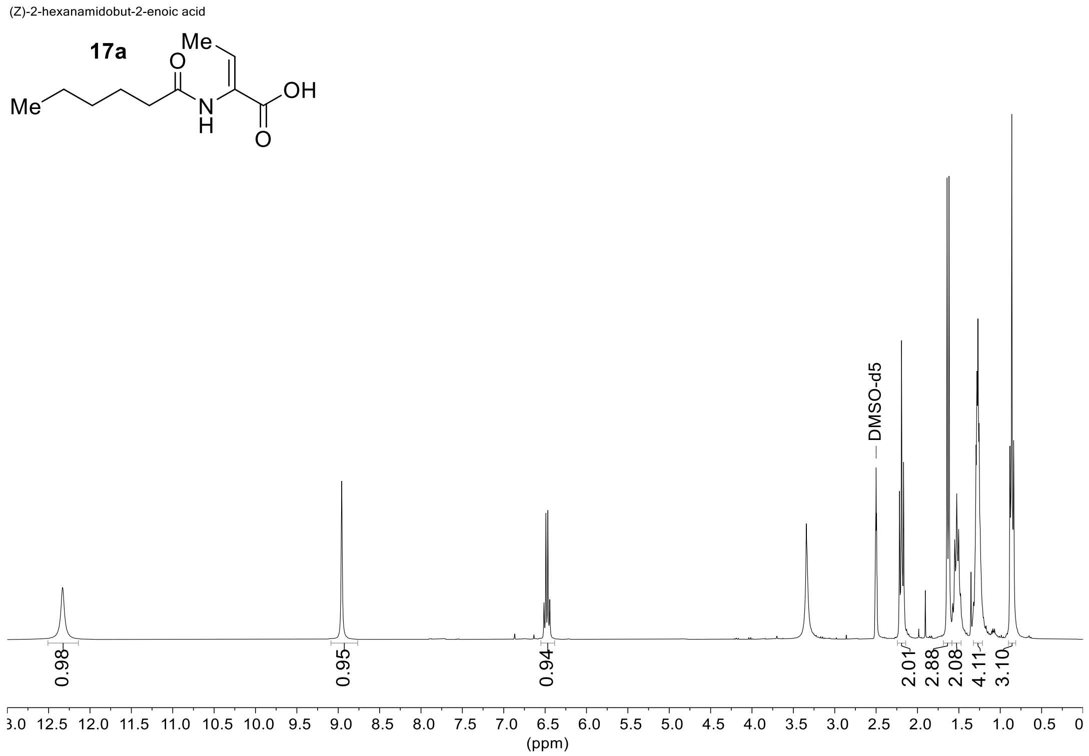

Figure S174. ^{1}H-NMR spectrum (300 MHz) of (*Z*)-2-hexanamidobut-2-enoic acid (**17a**).

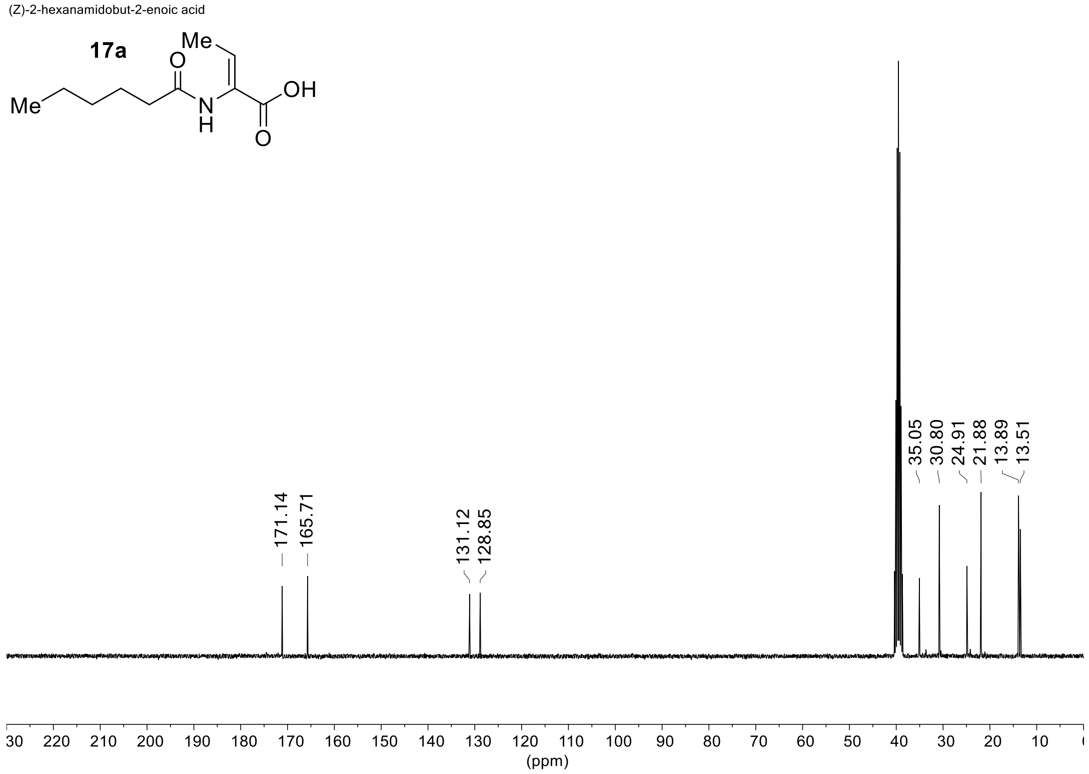

Figure S175. ^{13}C {^{1}H}-NMR spectrum (75.5 MHz) of (*Z*)-2-hexanamidobut-2-enoic acid (**17a**).

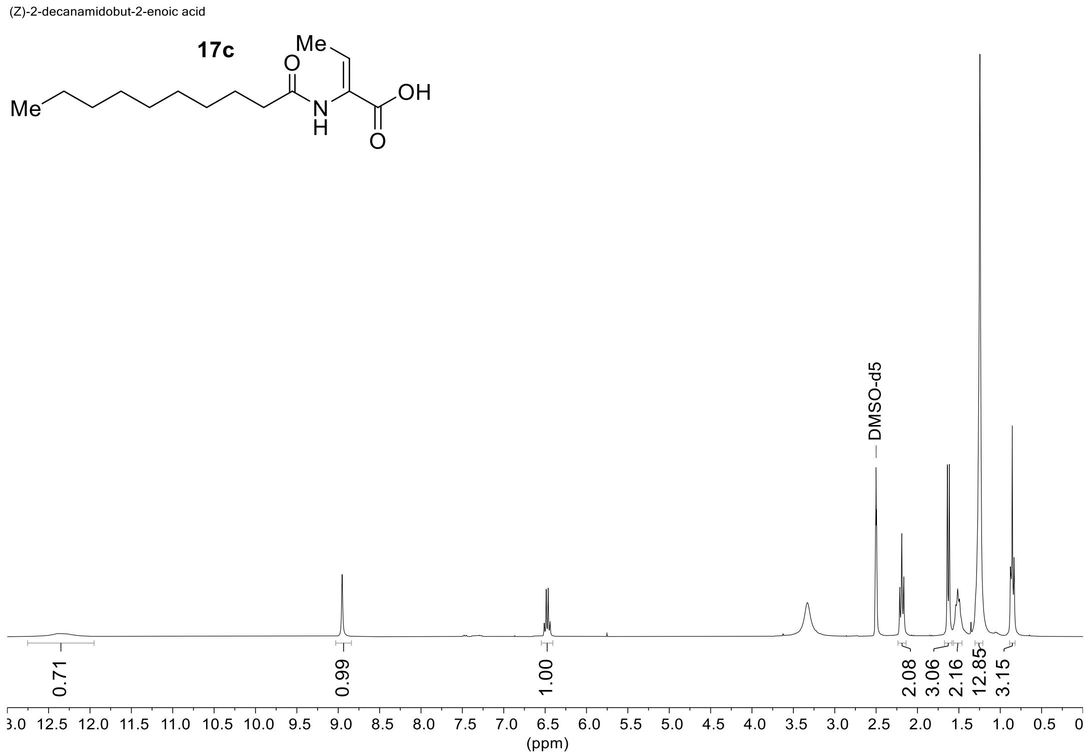

Figure S176. ^{1}H-NMR spectrum (300 MHz) of (*Z*)-2-decanamidobut-2-enoic acid (**17c**).

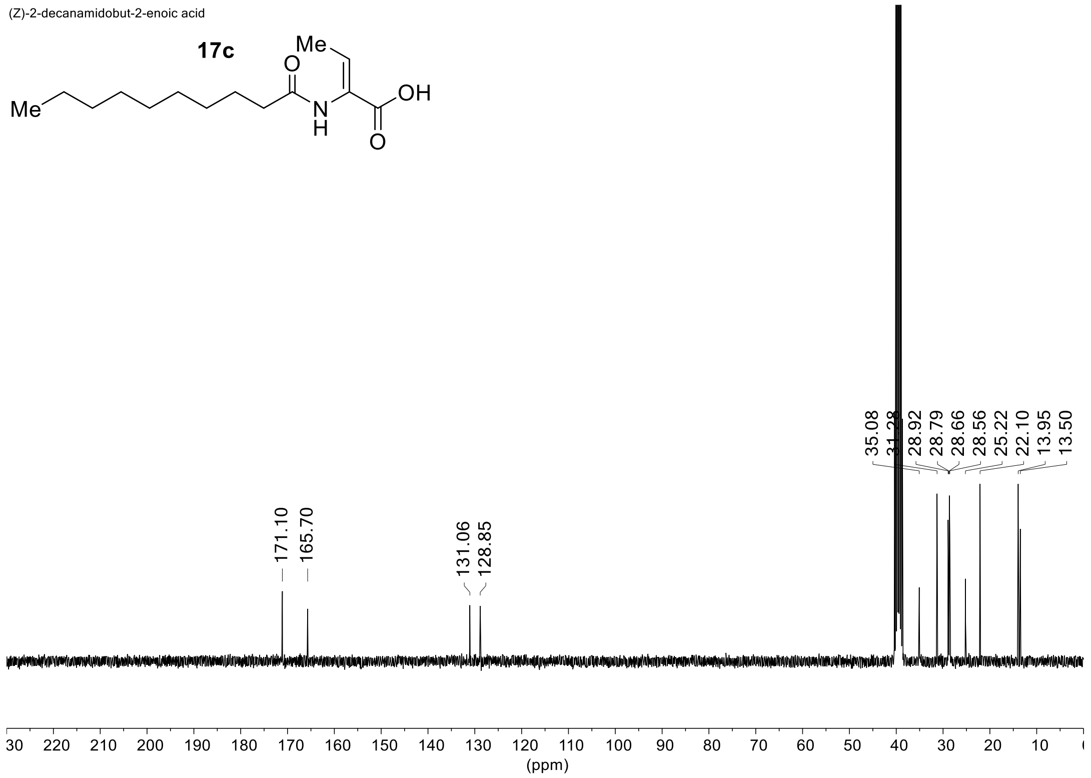

Figure S177. ^{13}C {^{1}H}-NMR spectrum (75.5 MHz) of (*Z*)-2-decanamidobut-2-enoic acid (**17c**).

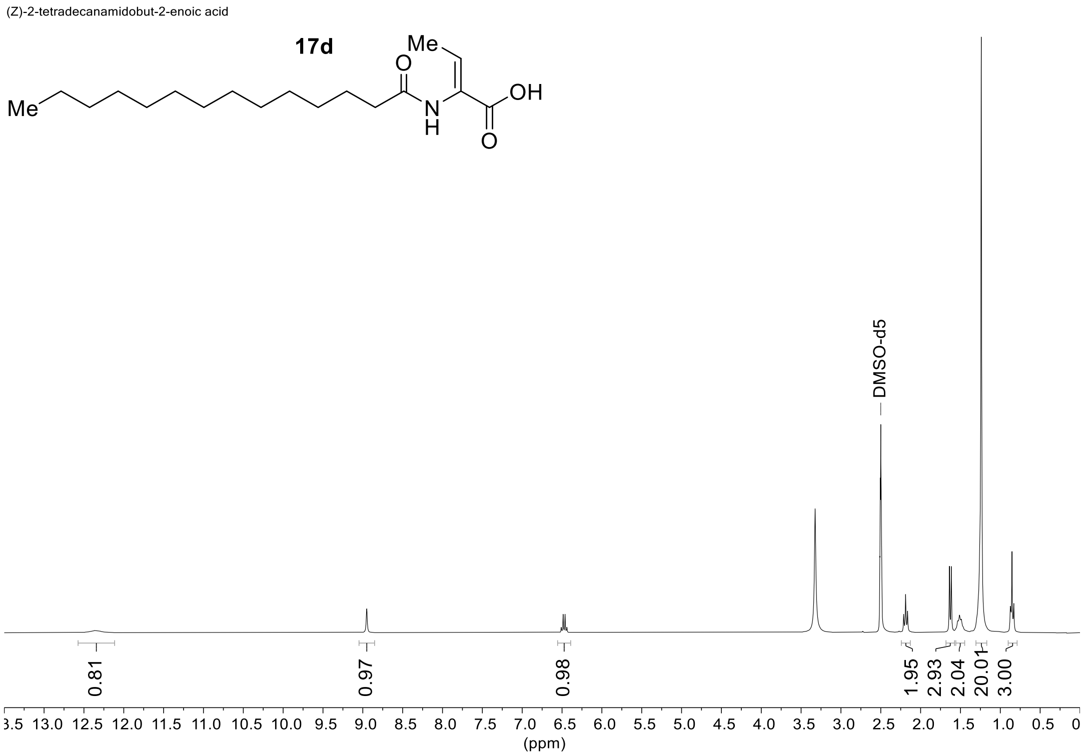

Figure S178. ^{1}H-NMR spectrum (300 MHz) of (*Z*)-2-tetradecanamidobut-2-enoic acid (**17d**).

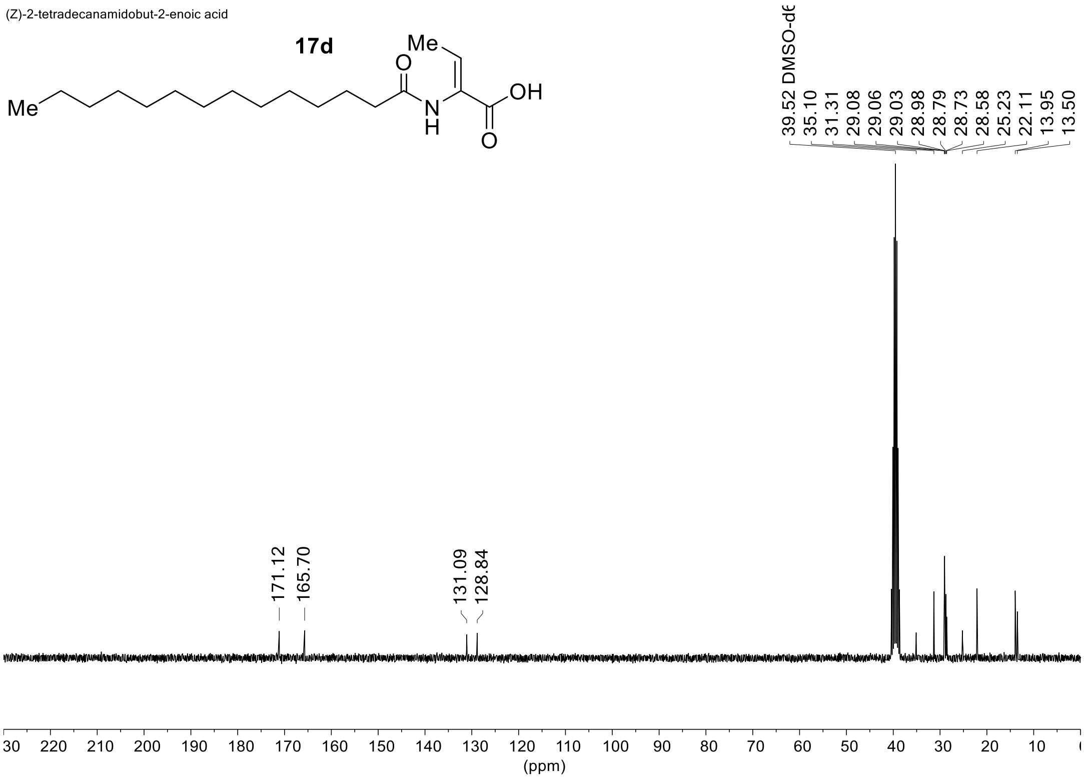

Figure S179. ^{13}C {^{1}H}-NMR spectrum (75.5 MHz) of (*Z*)-2-tetradecanamidobut-2-enoic acid (**17d**).

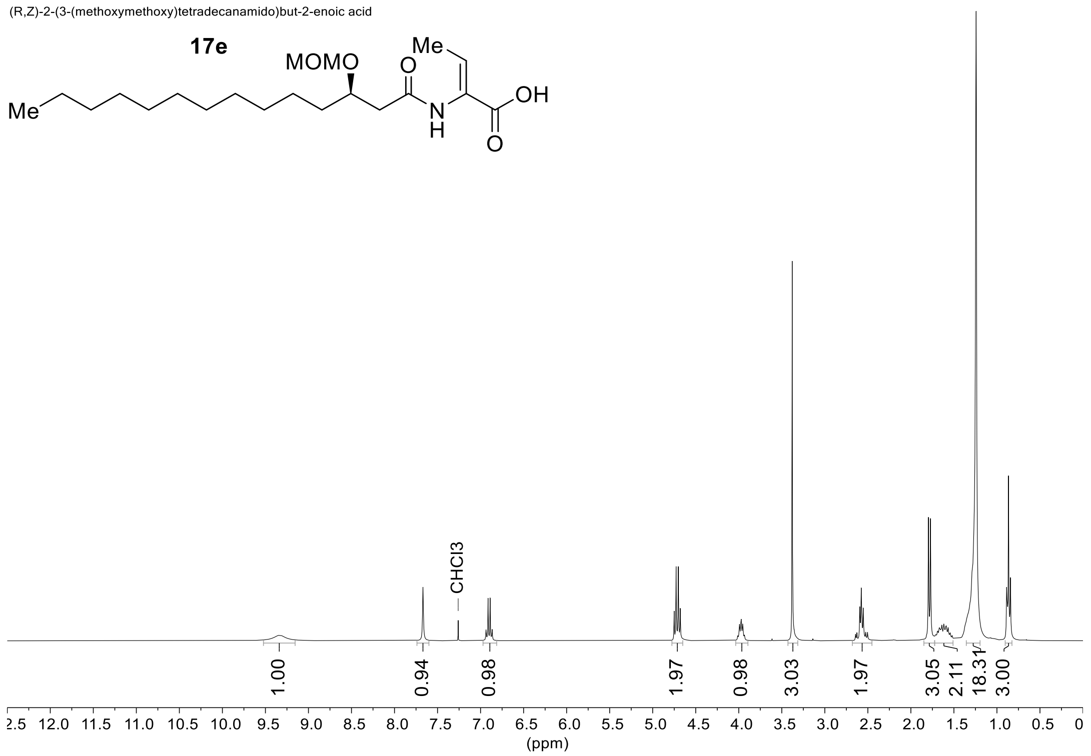

Figure S180. ^{1}H-NMR spectrum (300 MHz) of (*R*,*Z*)-2-(3-(methoxymethoxy)tetradecanamido)but-2-enoic acid (**17e**).

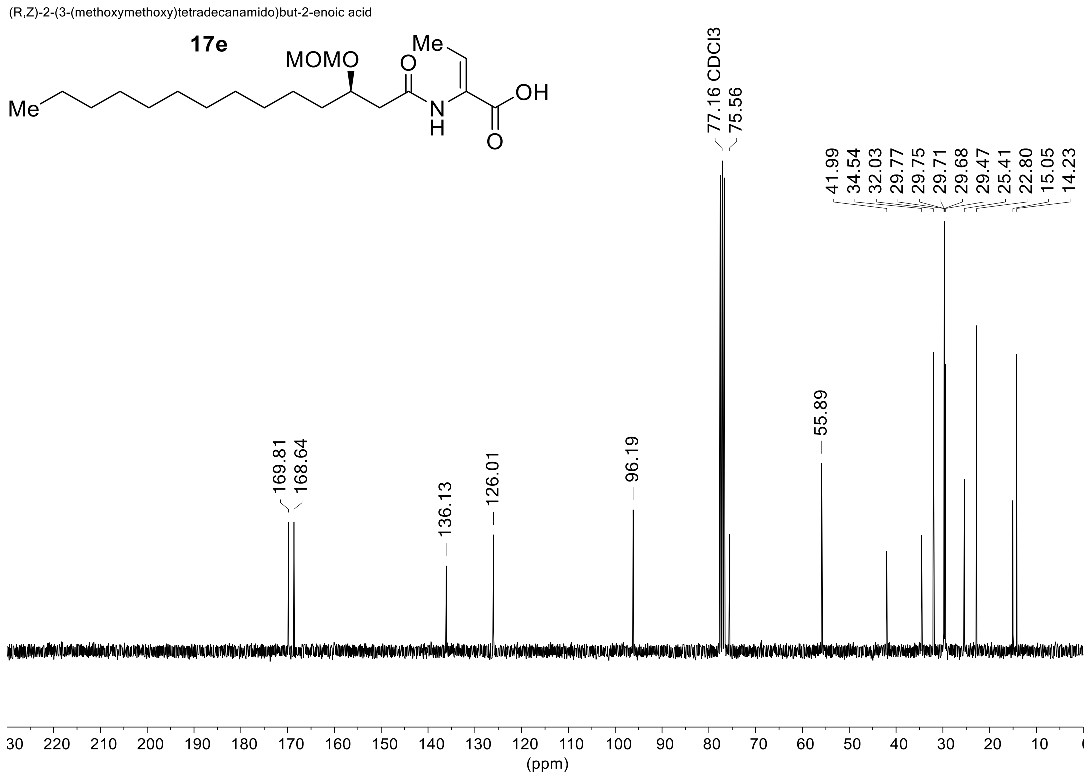

Figure S181. ^{13}C {^{1}H}-NMR spectrum (75.5 MHz) of (*R*,*Z*)-2-(3-(methoxymethoxy)tetradecanamido)but-2-enoic acid (**17e**).

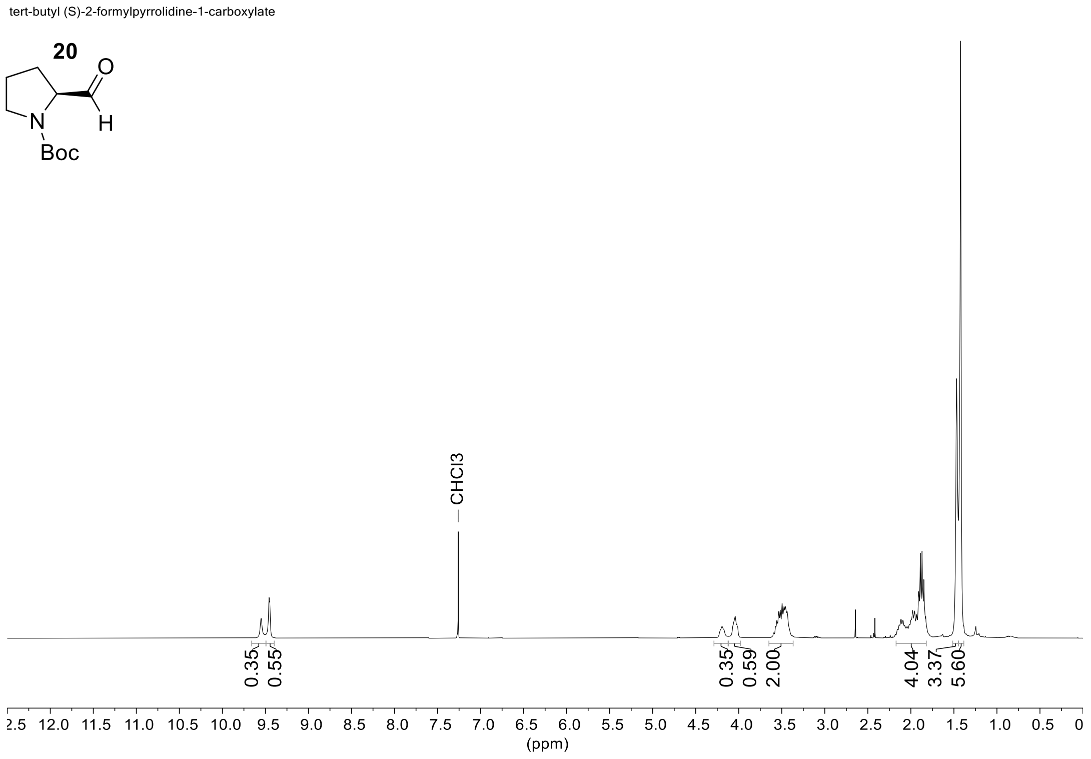

Figure S182. ^{1}H-NMR spectrum (300 MHz) of *N*-Boc-L-prolinal (**20**).

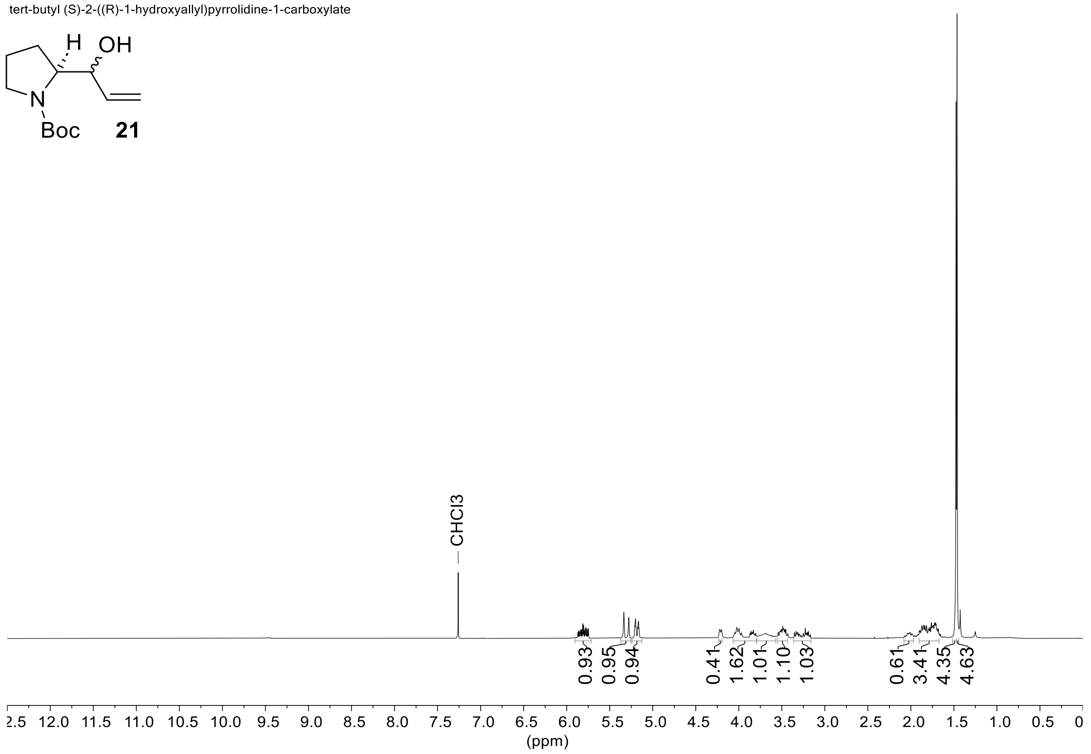

Figure S183. ^{1}H-NMR spectrum (300 MHz) of *tert*-butyl (*S*)-2-(1-hydroxyallyl)pyrrolidine-1-carboxylate (**21**).

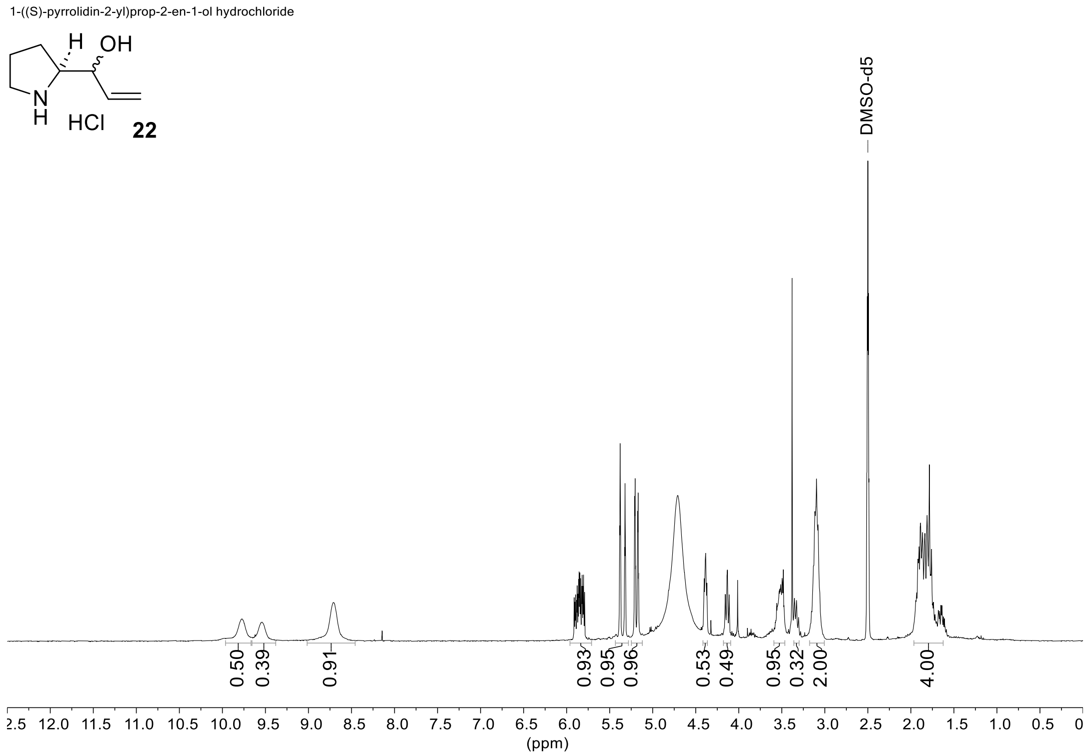

Figure S184. ^{1}H-NMR spectrum (300 MHz) of (*S*)-1-(pyrrolidin-2-yl)prop-2-en-1-ol hydrochloride (**22**).

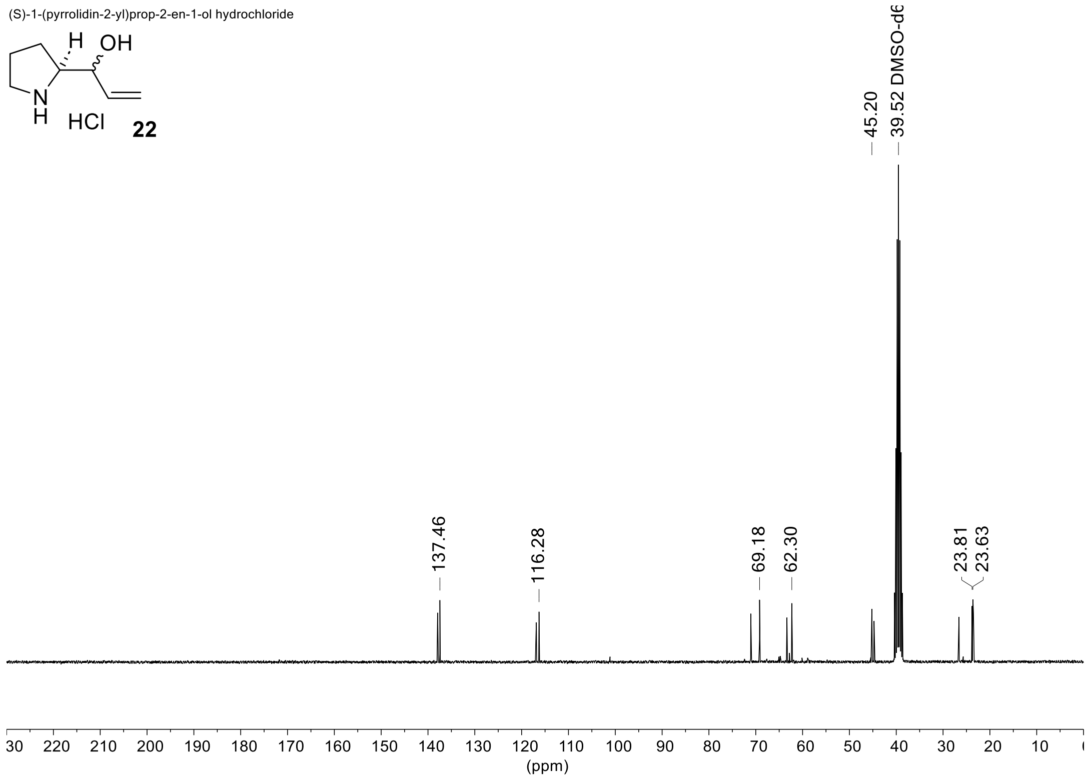

Figure S185. ^{13}C {^{1}H}-NMR spectrum (75.5 MHz) of (*S*)-1-(pyrrolidin-2-yl)prop-2-en-1-ol hydrochloride (**22**).

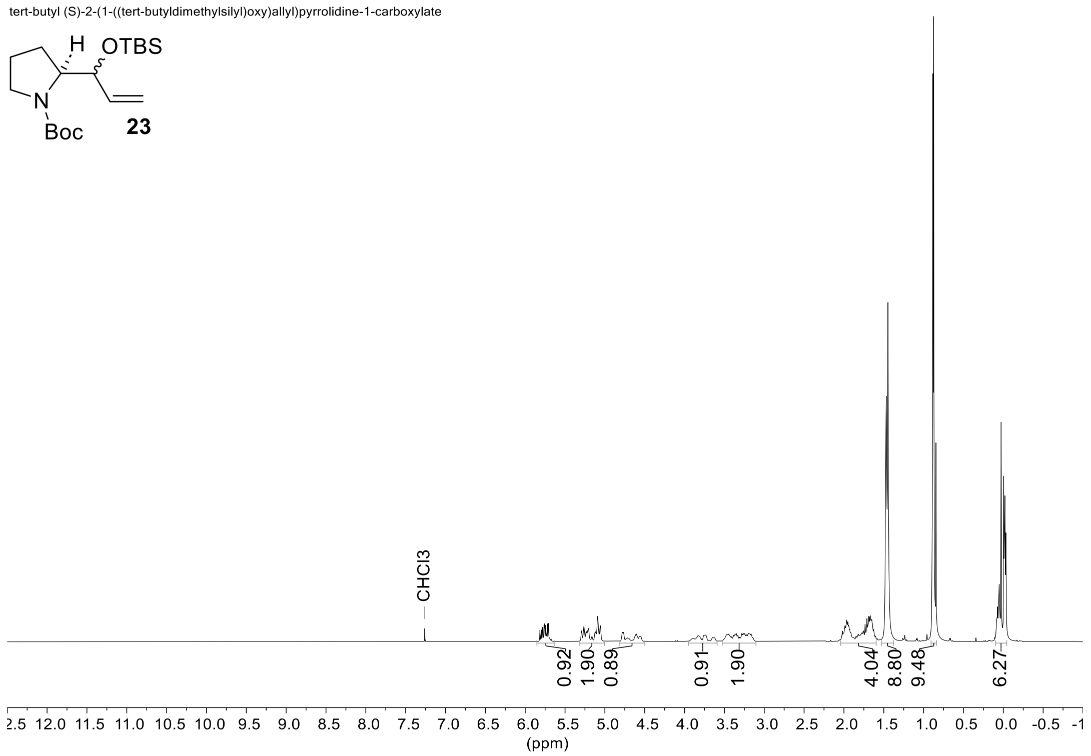

Figure S186. ^{1}H-NMR spectrum (300 MHz) of *tert*-butyl (*S*)-2-(1-((*tert*-butyldimethylsilyl)oxy)allyl)pyrrolidine-1-carboxylate (**23**).

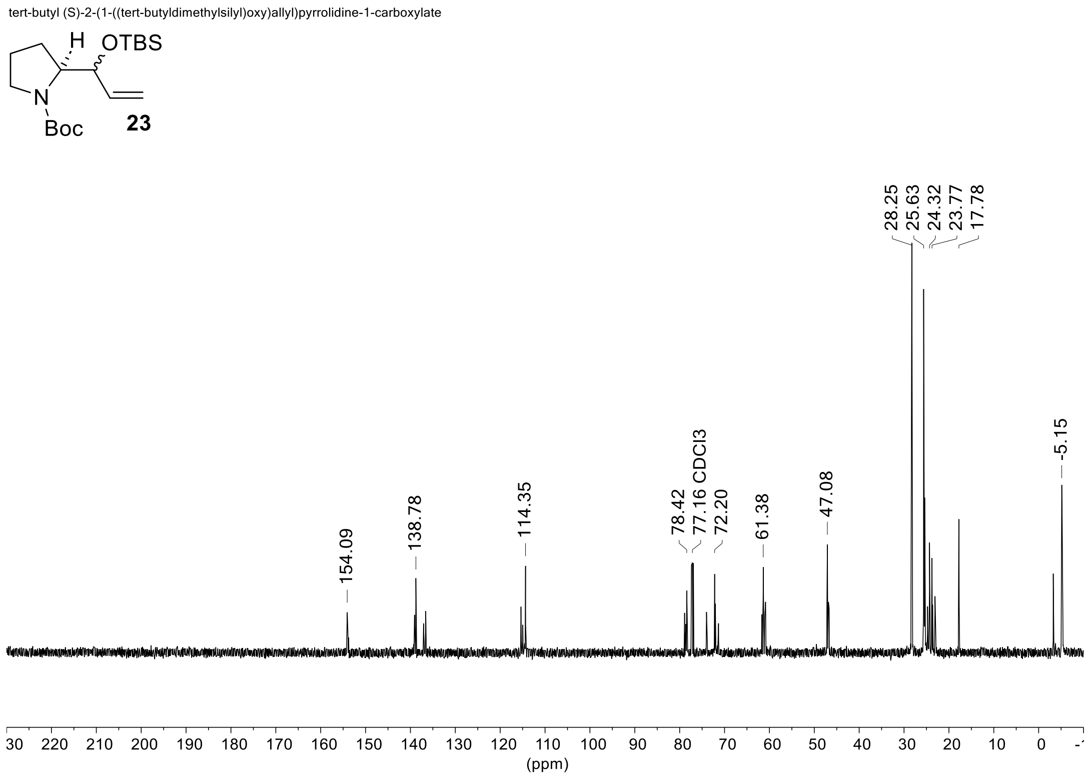

Figure S187. ^{13}C {^{1}H}-NMR spectrum (151 MHz) of *tert*-butyl (*S*)-2-(1-((*tert*-butyldimethylsilyl)oxy)allyl)pyrrolidine-1-carboxylate (**23**).

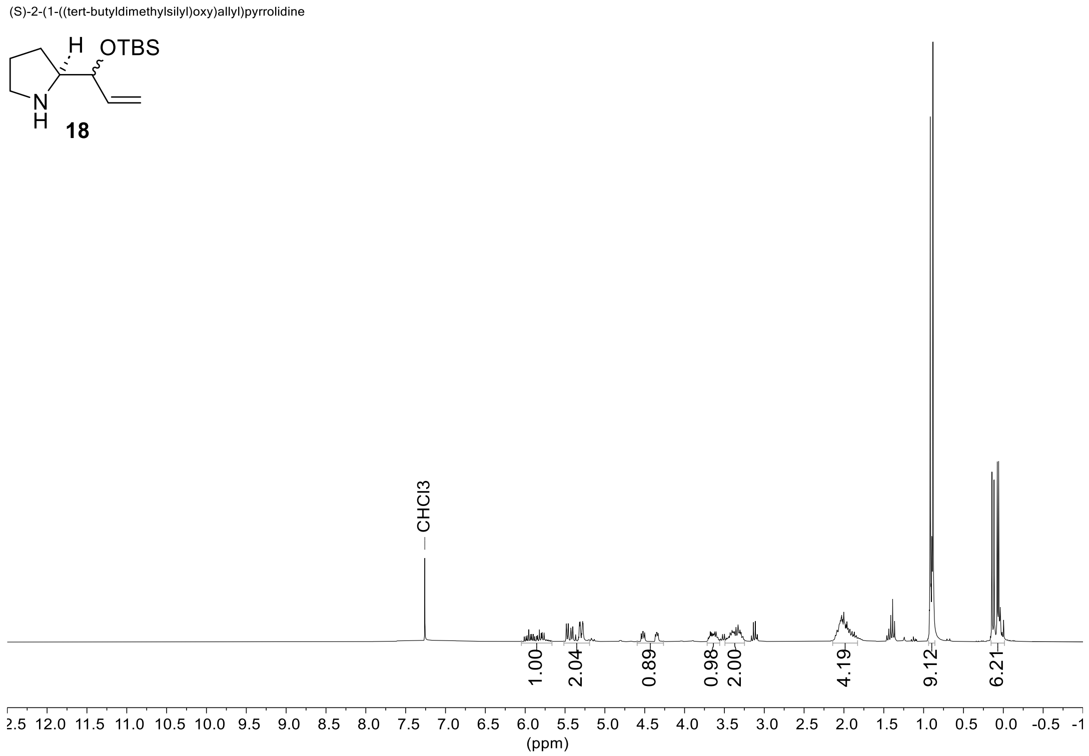

Figure S188. ^{1}H-NMR spectrum (300 MHz) of (*S*)-2-(1-((*tert*-butyldimethylsilyl)oxy)allyl)pyrrolidine (**18**).

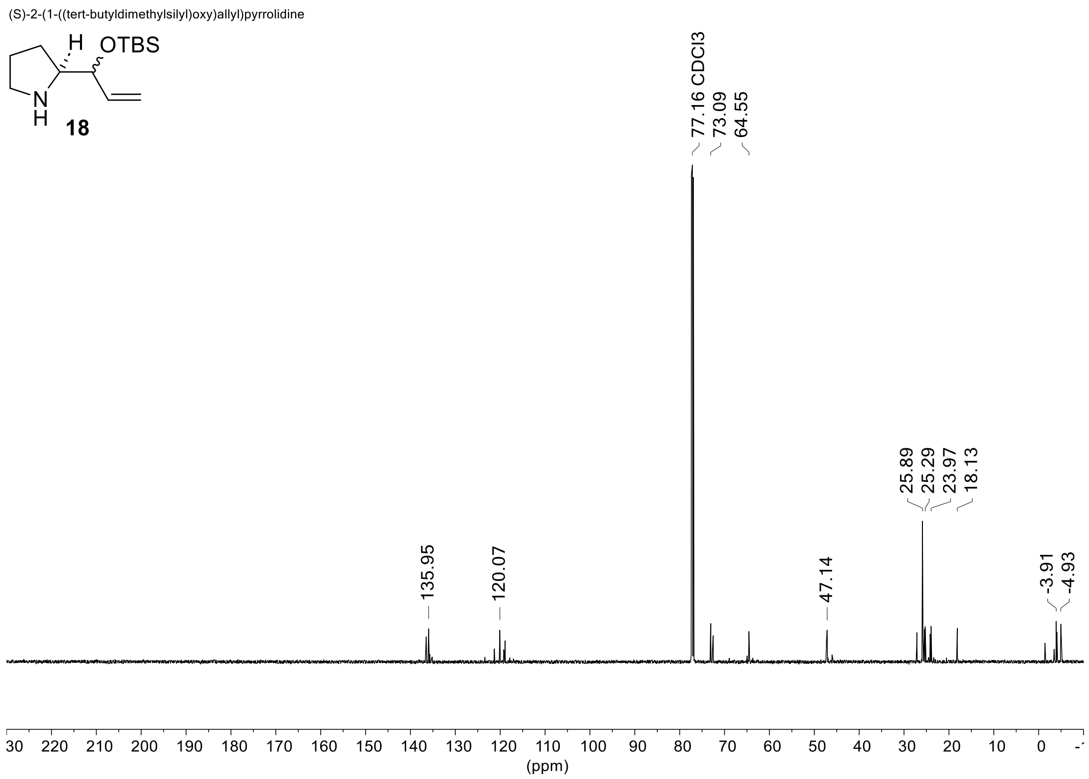

Figure S189. ^{13}C {^{1}H}-NMR spectrum (151 MHz) of (*S*)-2-(1-((*tert*-butyldimethylsilyl)oxy)allyl)pyrrolidine (**18**).

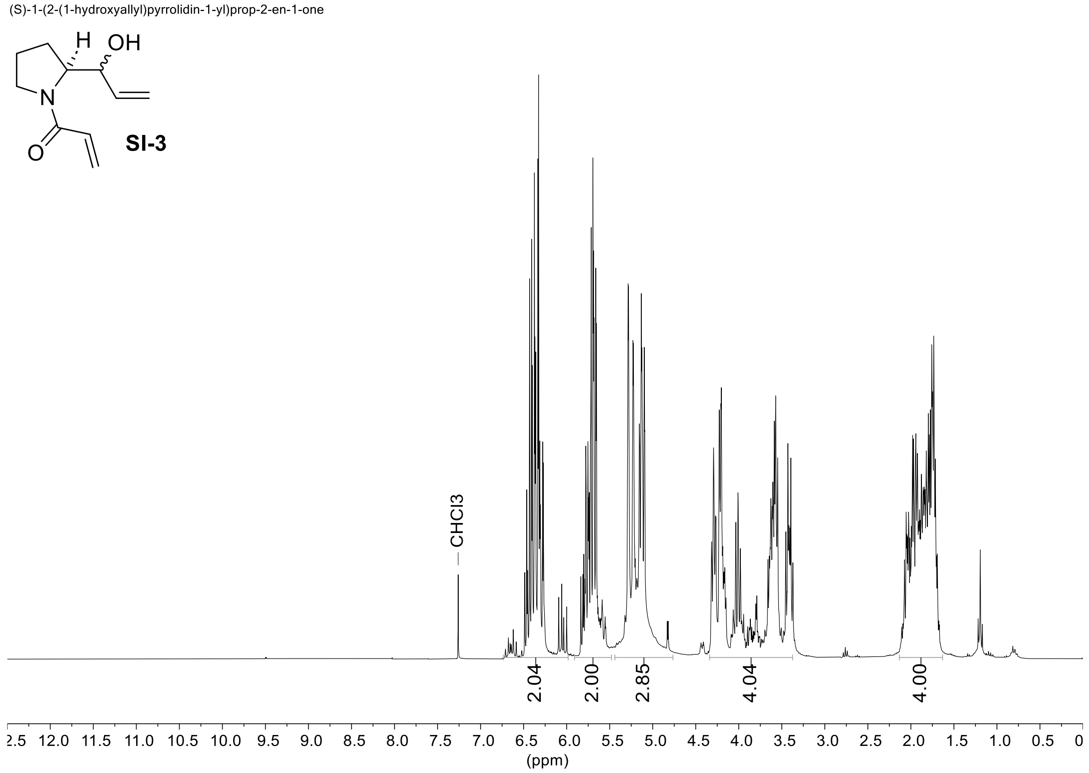

Figure S190. ^{1}H-NMR spectrum (300 MHz) of (*S*)-1-(2-(1-hydroxyallyl)pyrrolidin-1-yl)prop-2-en-1-one (**SI-3**).

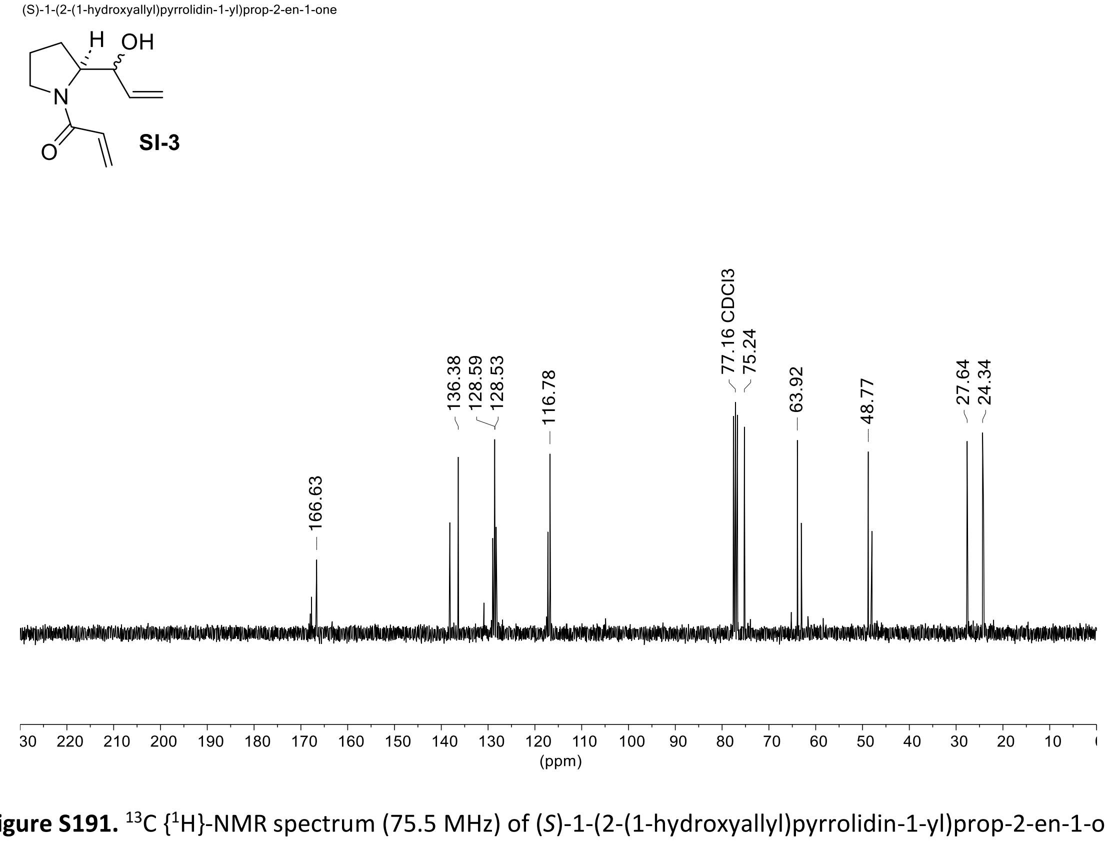

Figure S191. ^{13}C {^{1}H}-NMR spectrum (75.5 MHz) of (*S*)-1-(2-(1-hydroxyallyl)pyrrolidin-1-yl)prop-2-en-1-one (**SI-3**).

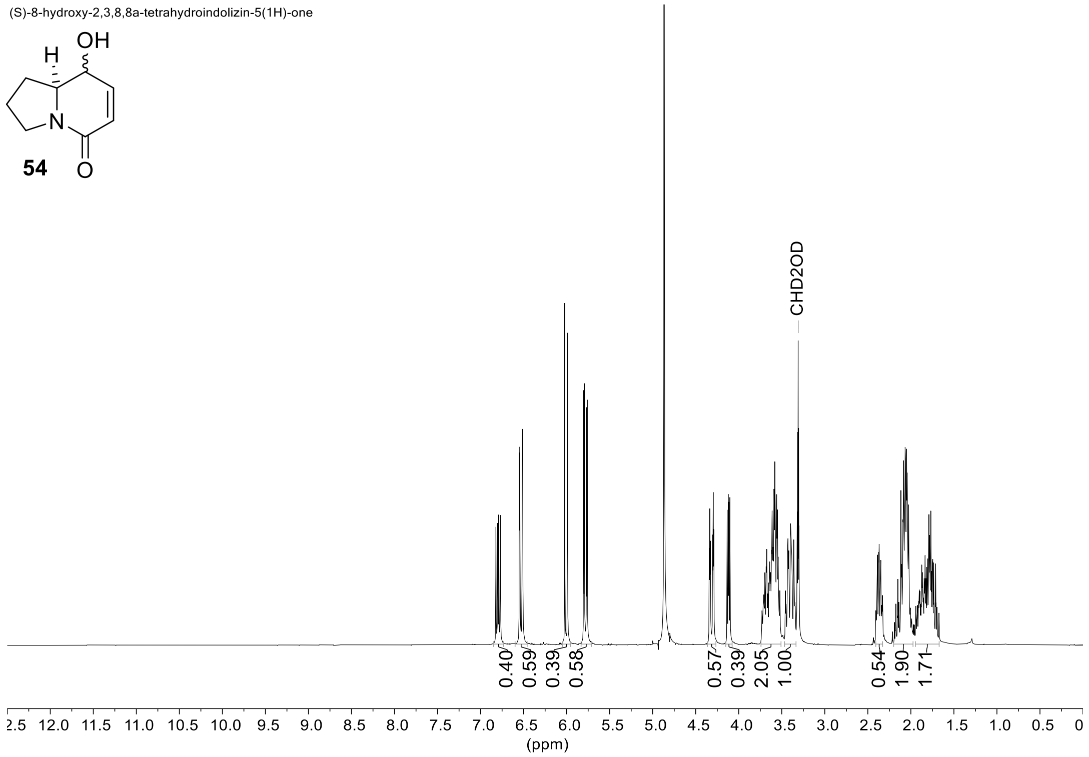

Figure S192. ^{1}H-NMR spectrum (300 MHz) of (*S*)-8-hydroxy-2,3,8,8a-tetrahydroindolizin-5(1*H*)-one (**54**).

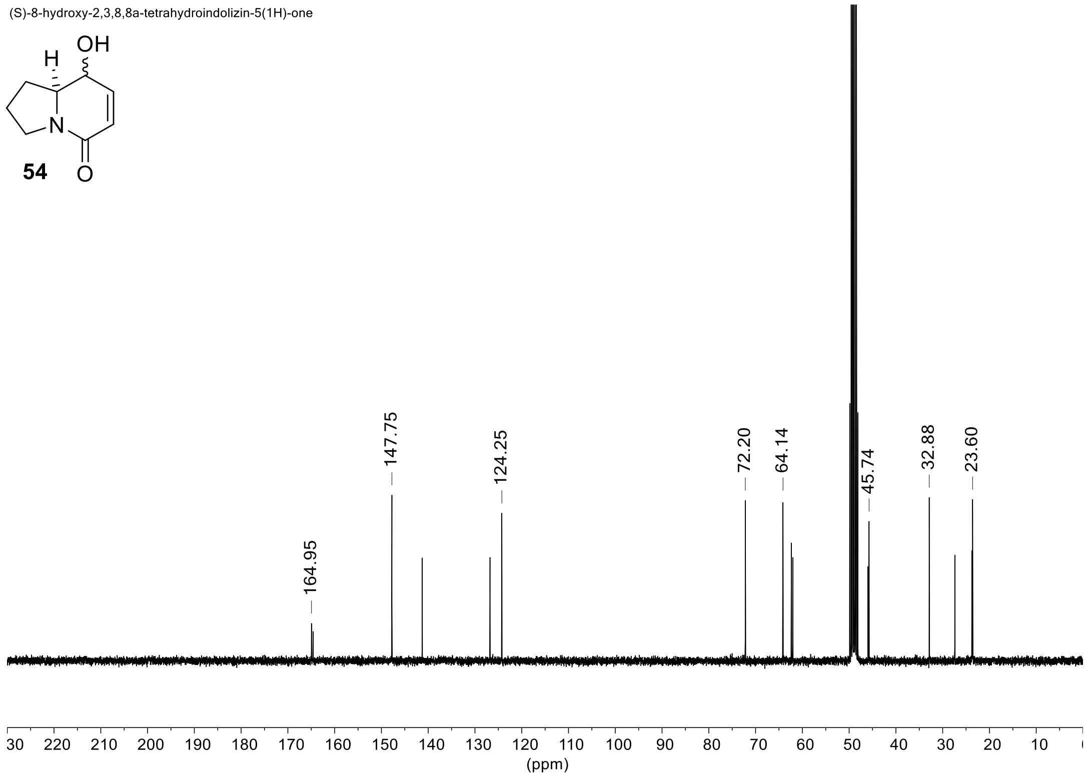

Figure S193. ^{13}C {^{1}H}-NMR spectrum (75.5 MHz) of (*S*)-8-hydroxy-2,3,8,8a-tetrahydroindolizin-5(1*H*)-one (**54**).

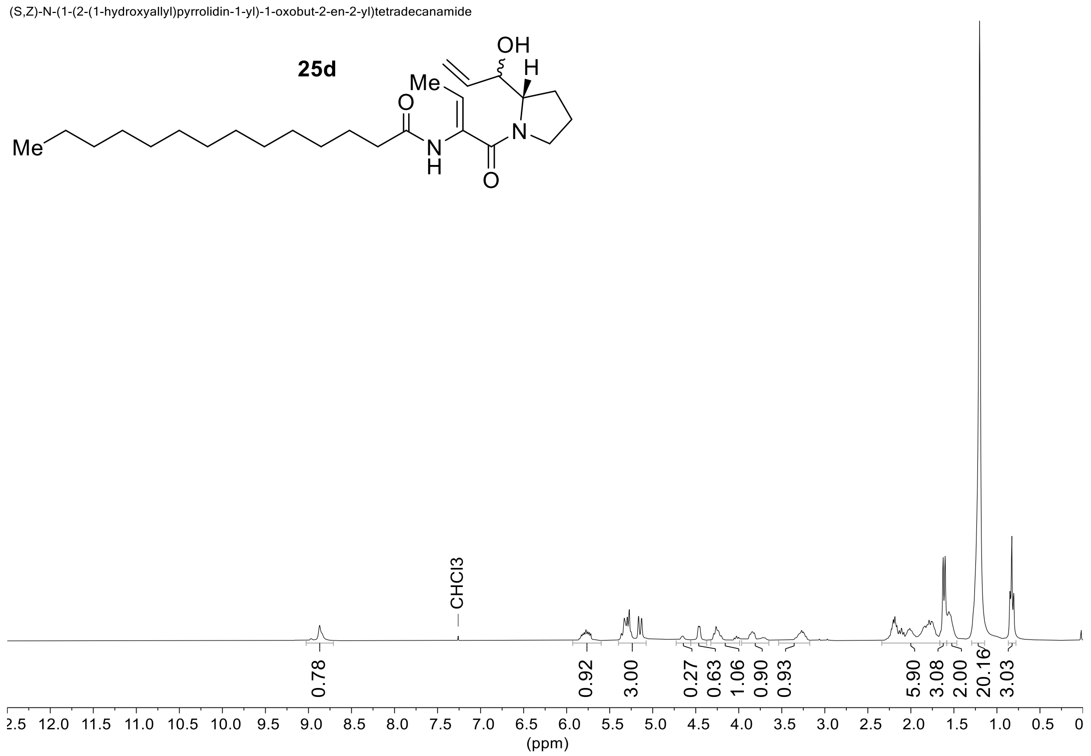

Figure S194. ^{1}H-NMR spectrum (300 MHz) of (*S*,*Z*)-*N*-(1-(2-(1-hydroxyallyl)pyrrolidin-1-yl)-1-oxobut-2-en-2-yl)tetradecanamide (**25d**).

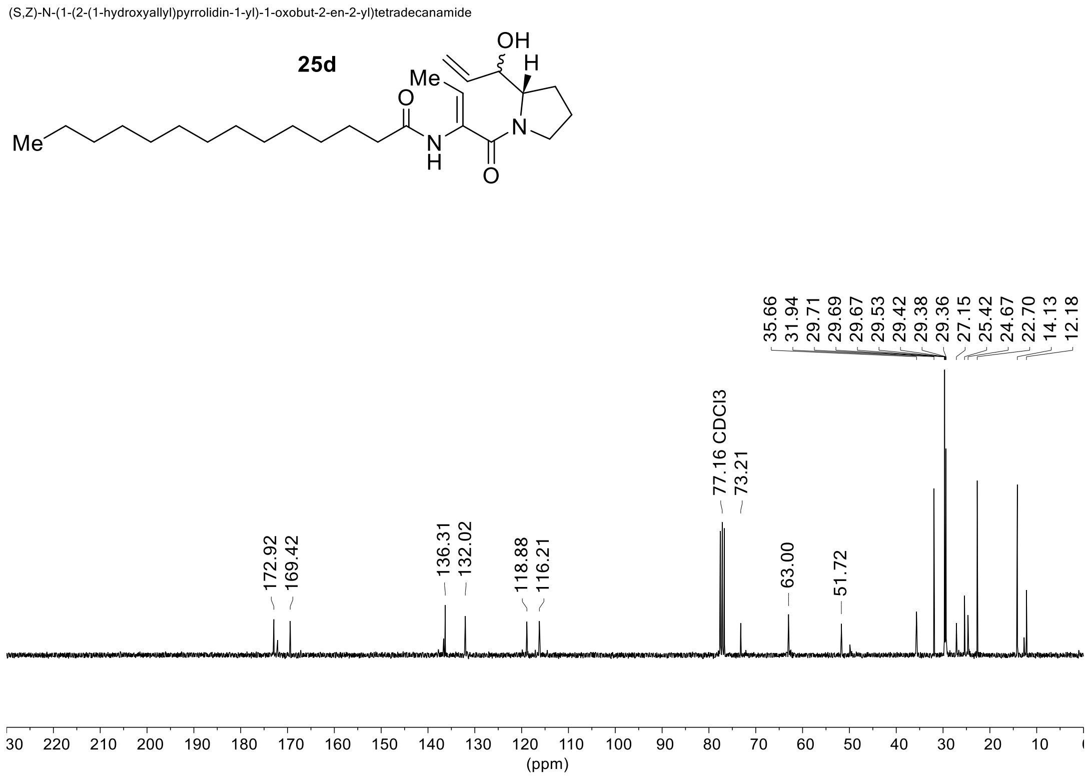

Figure S195. ^{13}C {^{1}H}-NMR spectrum (75.5 MHz) of (*S*,*Z*)-*N*-(1-(2-(1-hydroxyallyl)pyrrolidin-1-yl)-1-oxobut-2-en-2-yl)tetradecanamide (**25d**).

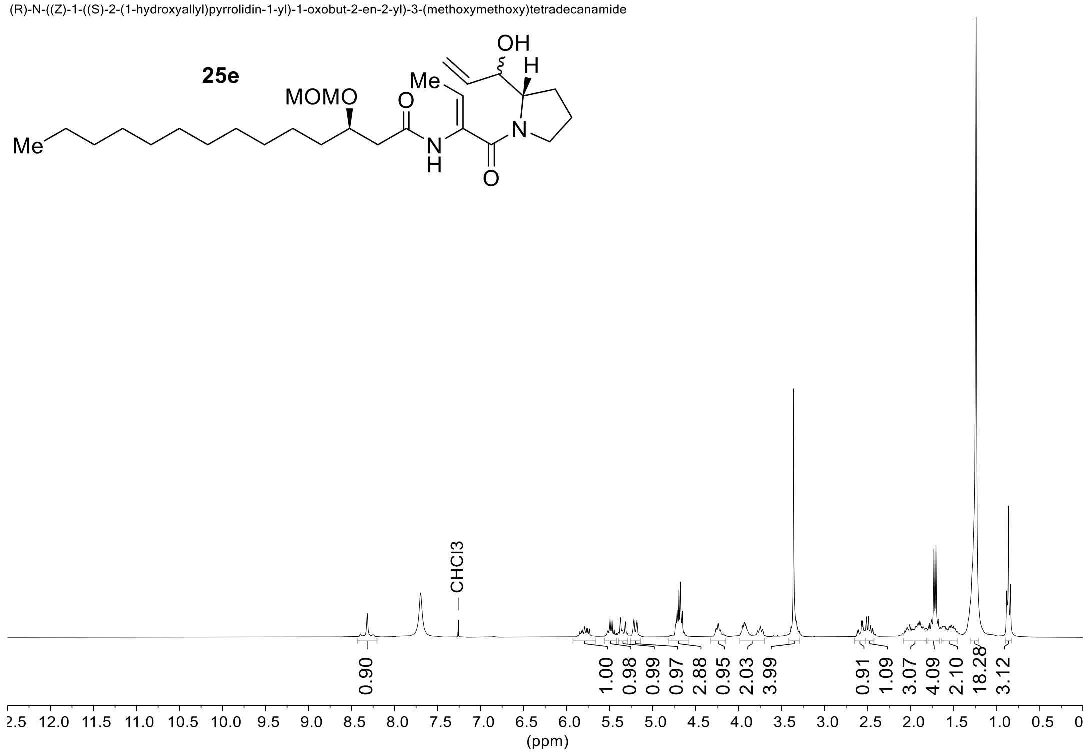

Figure S196. ^{1}H-NMR spectrum (300 MHz) of (*R*)-*N*-((*Z*)-1-((*S*)-2-(1-hydroxyallyl)pyrrolidin-1-yl)-1-oxobut-2-en-2-yl)-3-(methoxymethoxy)tetradecanamide (**25e**).

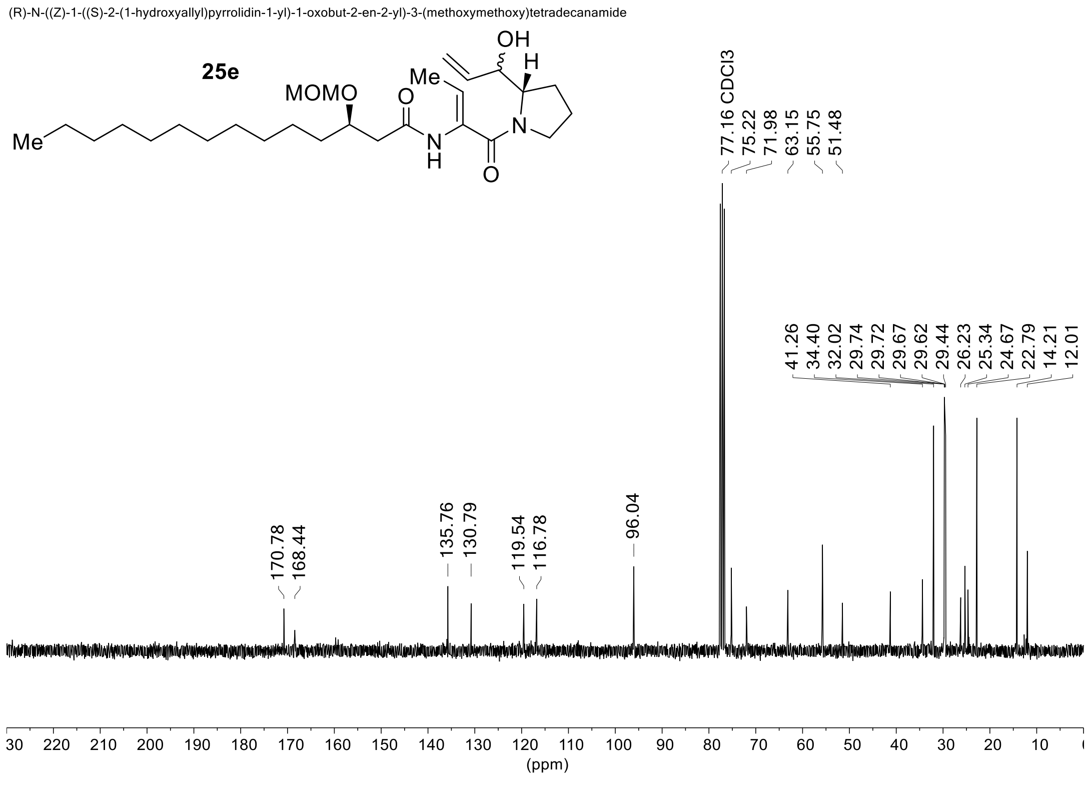

Figure S197. ^{13}C {^{1}H}-NMR spectrum (75.5 MHz) of (*R*)-*N*-((*Z*)-1-((*S*)-2-(1-hydroxyallyl)pyrrolidin-1-yl)-1-oxobut-2-en-2-yl)-3-(methoxymethoxy)tetradecanamide (**25e**).

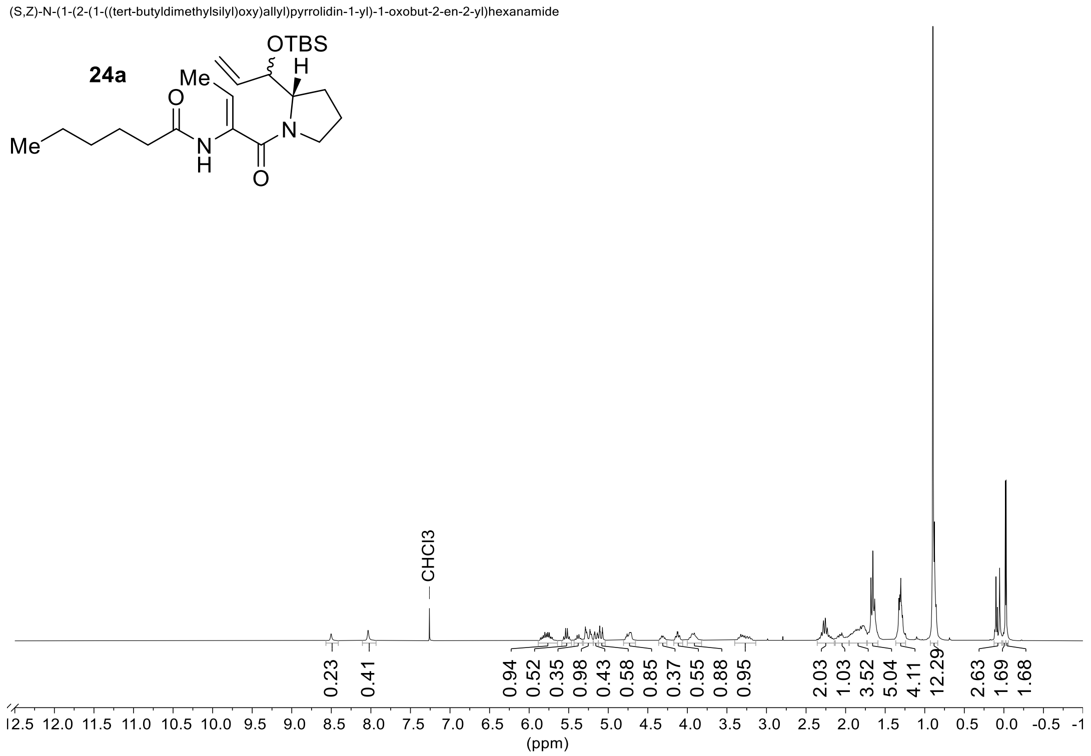

Figure S198. ^{1}H-NMR spectrum (300 MHz) of (*S,Z*)-*N*-(1-(2-(1-((tert-butyldimethylsilyl)oxy)allyl)pyrrolidin-1-yl)-1-oxobut-2-en-2-yl)hexanamide (**24a**).

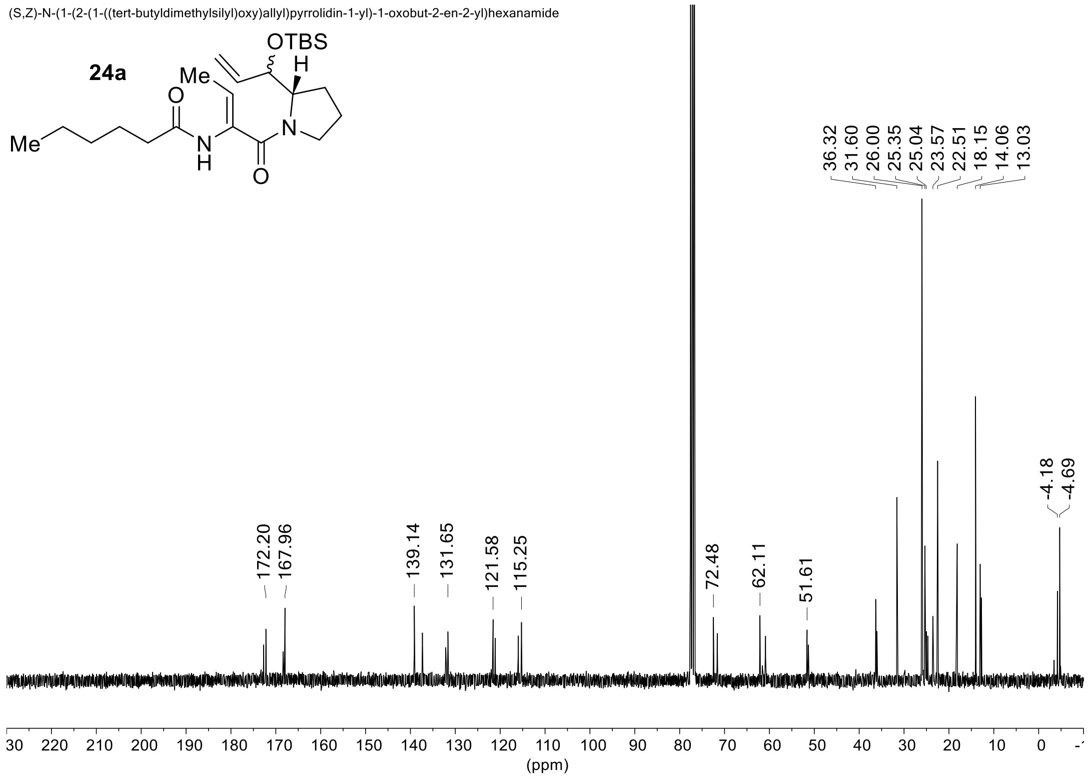

Figure S199. ^{13}C {^{1}H}-NMR spectrum (75.5 MHz) of (*S,Z*)-*N*-(1-(2-(1-((tert-butyldimethylsilyl)oxy)allyl)pyrrolidin-1-yl)-1-oxobut-2-en-2-yl)hexanamide (**24a**).

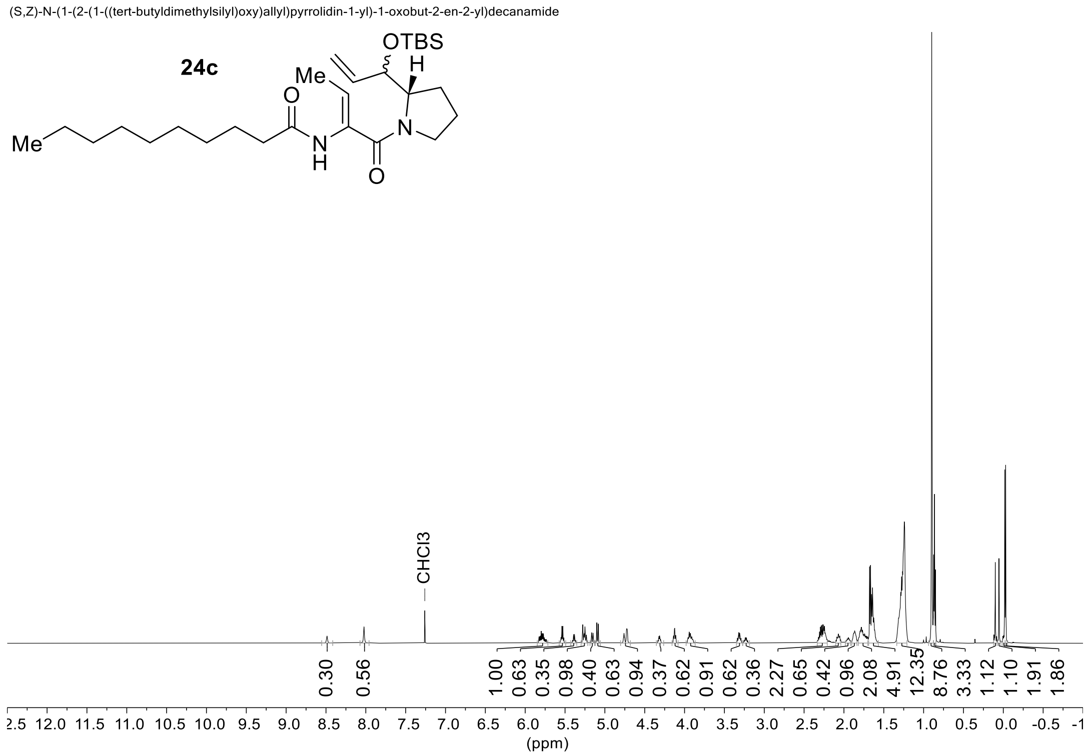

Figure S200. ^{1}H-NMR spectrum (600 MHz) of (*S*,*Z*)-*N*-(1-(2-(1-((tert-butyldimethylsilyl)oxy)allyl)pyrrolidin-1-yl)-1-oxobut-2-en-2-yl)decanamide (**24c**).

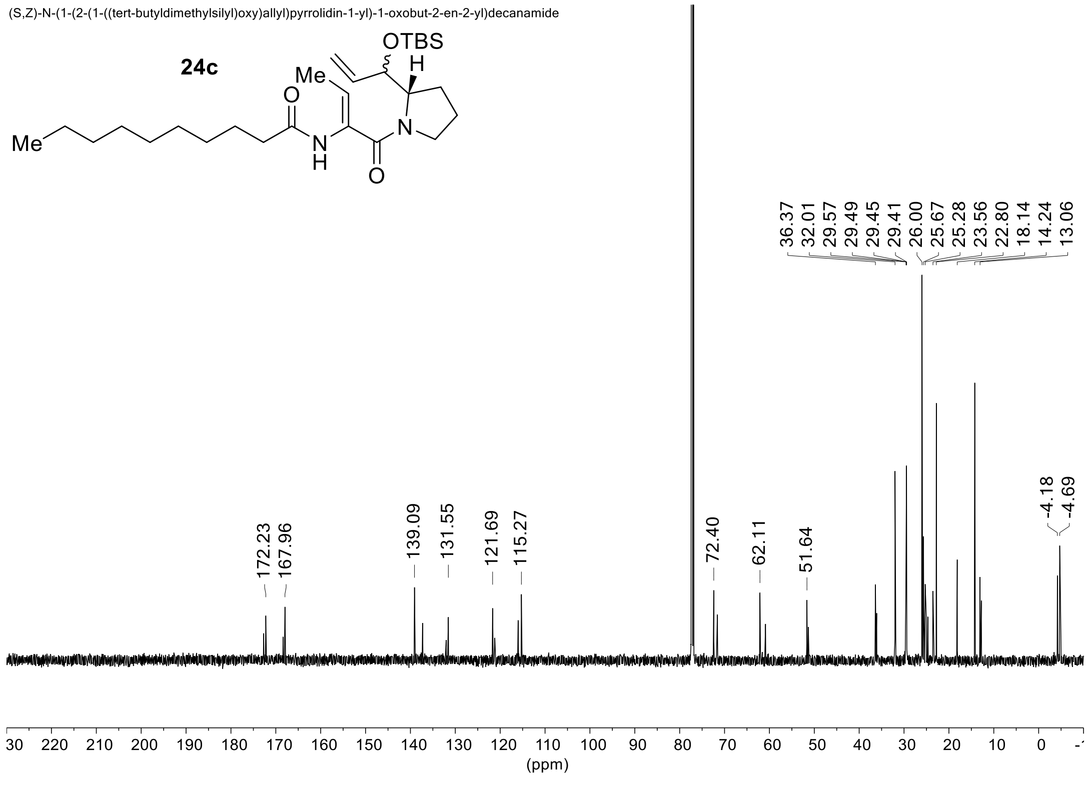

Figure S201. ^{13}C {^{1}H}-NMR spectrum (151 MHz) of (*S*,*Z*)-*N*-(1-(2-(1-((tert-butyldimethylsilyl)oxy)allyl)pyrrolidin-1-yl)-1-oxobut-2-en-2-yl)decanamide (**24c**).

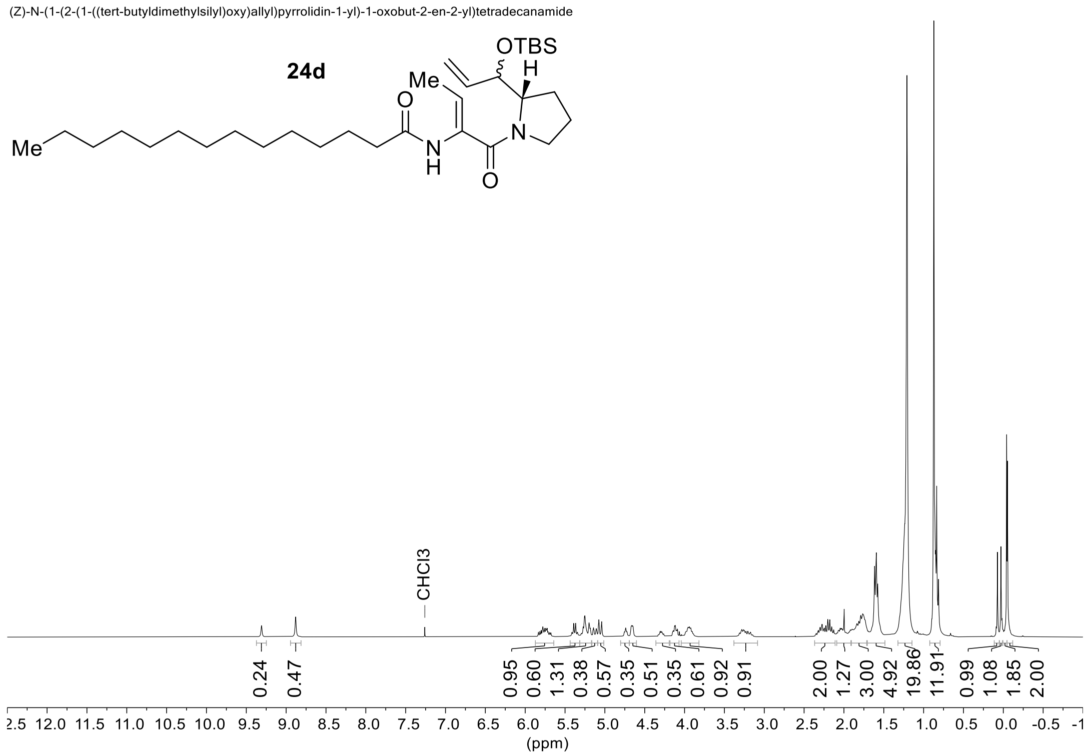

Figure S202. ^{1}H-NMR spectrum (300 MHz) of (*S*,*Z*)-*N*-(1-(2-(1-((tert-butyldimethylsilyl)oxy)allyl)pyrrolidin-1-yl)-1-oxobut-2-en-2-yl)tetradecanamide (**24d**).

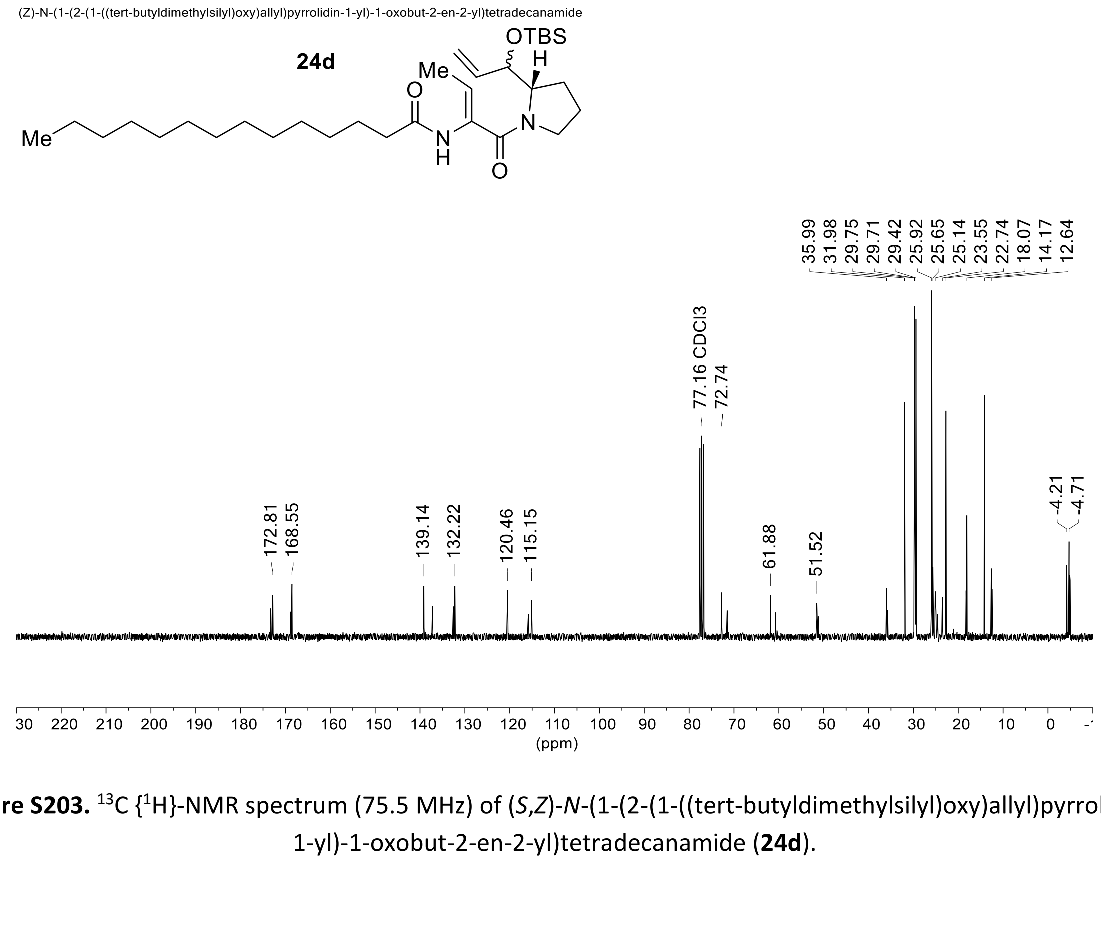

Figure S203. ^{13}C {^{1}H}-NMR spectrum (75.5 MHz) of (*S*,*Z*)-*N*-(1-(2-(1-((tert-butyldimethylsilyl)oxy)allyl)pyrrolidin-1-yl)-1-oxobut-2-en-2-yl)tetradecanamide (**24d**).

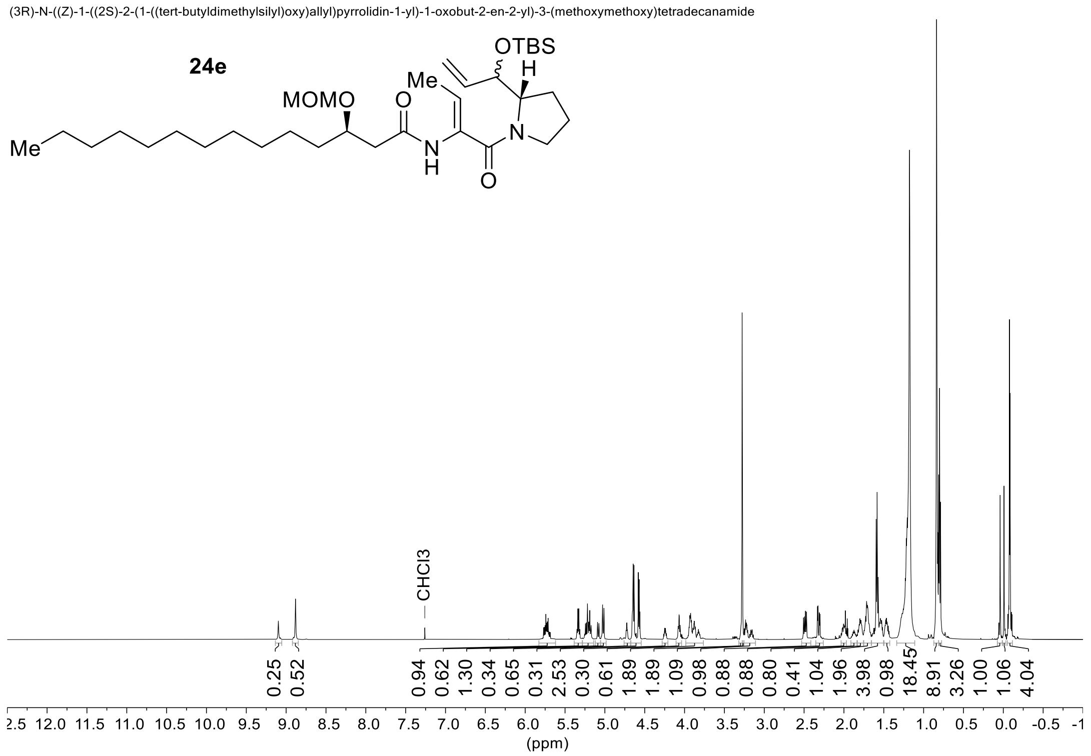

Figure S204. ^{1}H-NMR spectrum (600 MHz) of (*R*)-*N*-((*Z*)-1-((S)-2-(1-((tert-butyldimethylsilyl)oxy)allyl)pyrrolidin-1-yl)-1-oxobut-2-en-2-yl)-3-(methoxymethoxy)tetradecanamide (**24e**).

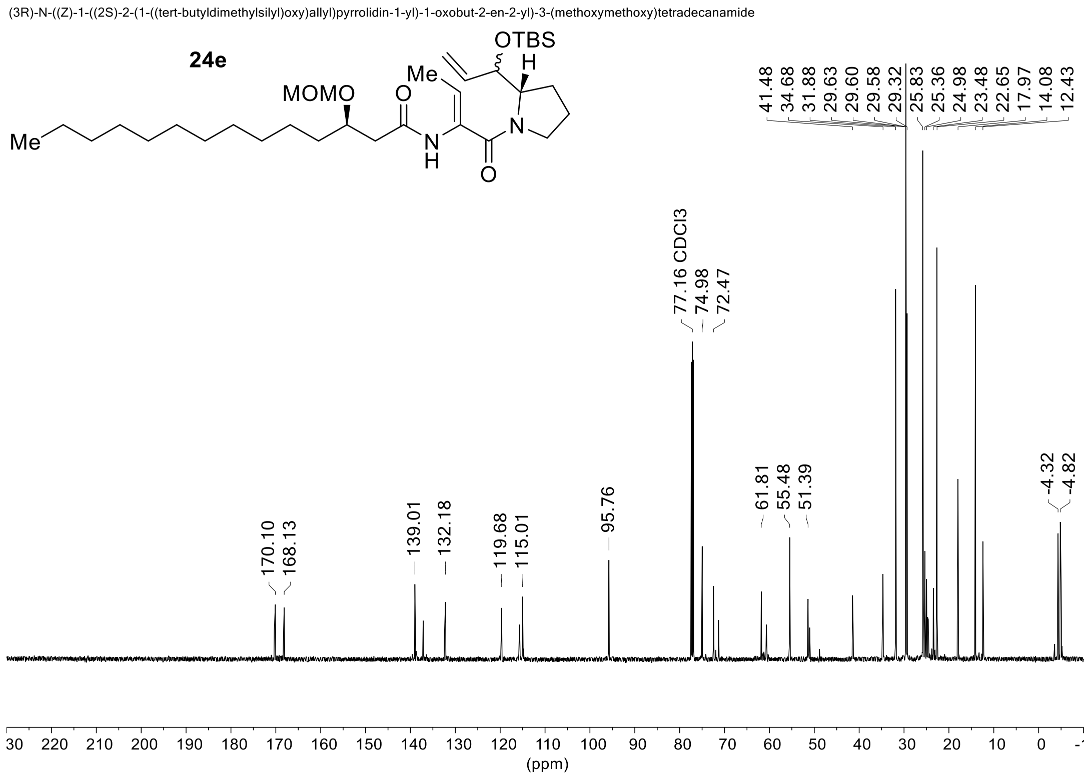

Figure S205. ^{13}C {^{1}H}-NMR spectrum (151 MHz) of (*R*)-*N*-((*Z*)-1-((S)-2-(1-((tert-butyldimethylsilyl)oxy)allyl)pyrrolidin-1-yl)-1-oxobut-2-en-2-yl)-3-(methoxymethoxy)tetradecanamide (**24e**).

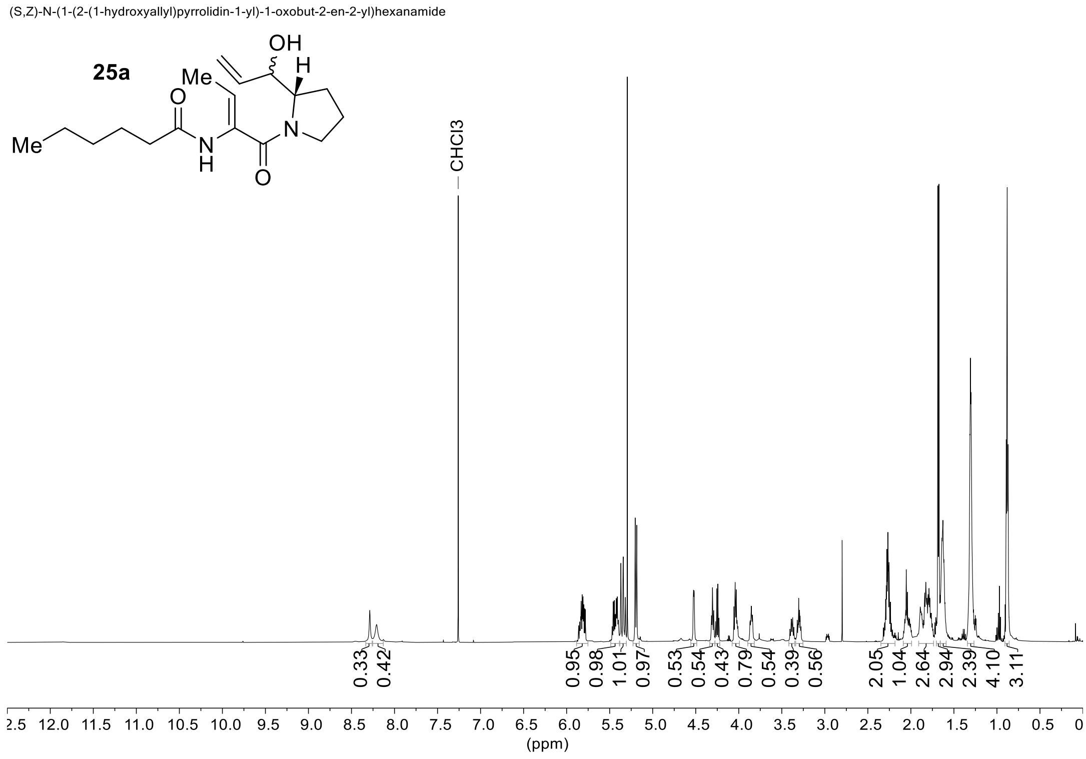

Figure S206. ^{1}H-NMR spectrum (600 MHz) of (*S*,*Z*)-*N*-(1-(2-(1-hydroxyallyl)pyrrolidin-1-yl)-1-oxobut-2-en-2-yl)hexanamide (**25a**).

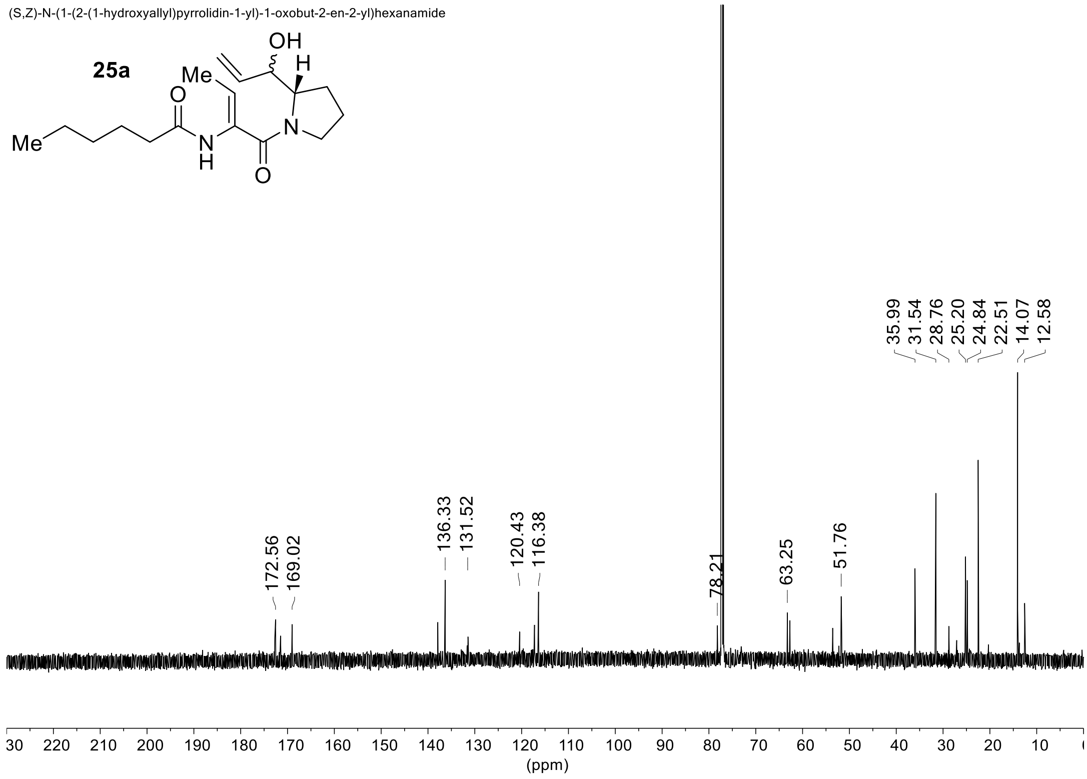

Figure S207. ^{13}C {^{1}H}-NMR spectrum (151 MHz) of (*S*,*Z*)-*N*-(1-(2-(1-hydroxyallyl)pyrrolidin-1-yl)-1-oxobut-2-en-2-yl)hexanamide (**25a**).

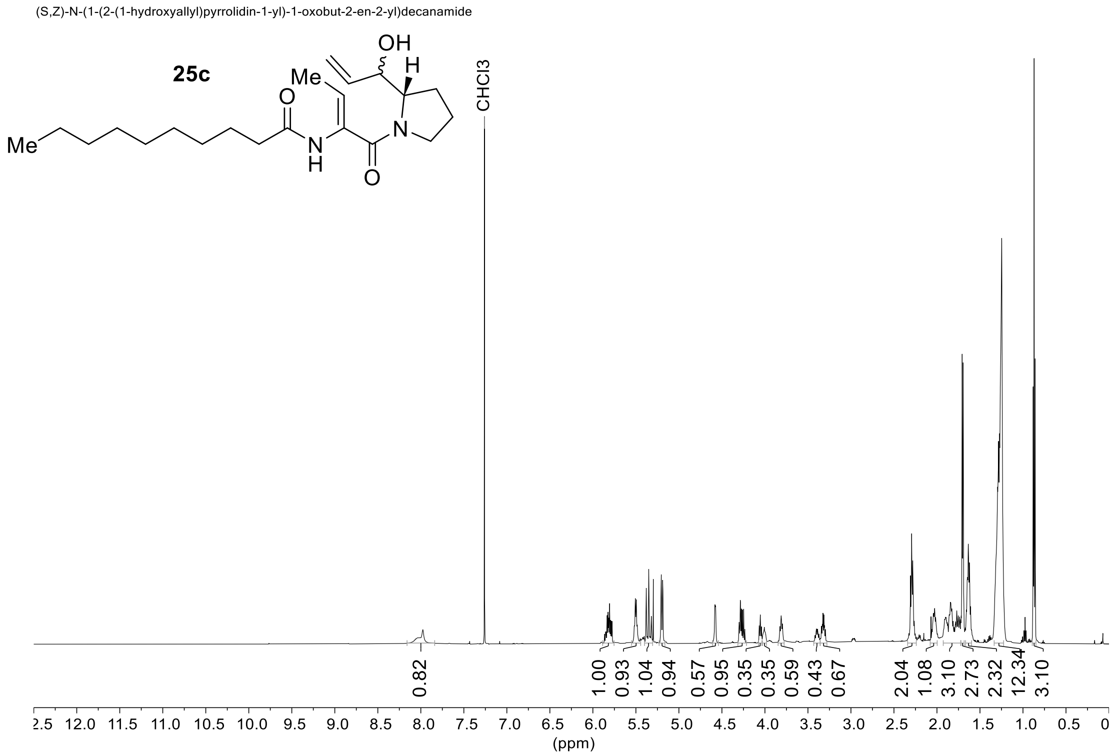

Figure S208. ^{1}H-NMR spectrum (600 MHz) of (*S*,*Z*)-*N*-(1-(2-(1-hydroxyallyl)pyrrolidin-1-yl)-1-oxobut-2-en-2-yl)decanamide (**25c**).

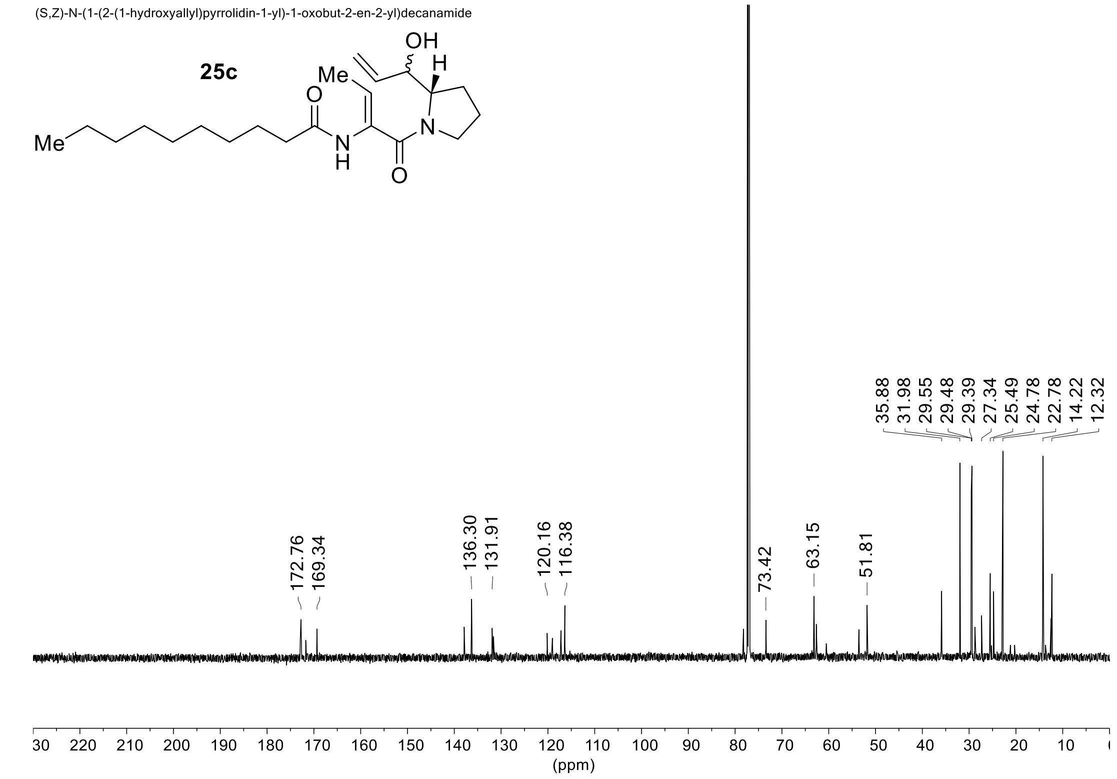

Figure S209. ^{13}C {^{1}H}-NMR spectrum (151 MHz) of (*S*,*Z*)-*N*-(1-(2-(1-hydroxyallyl)pyrrolidin-1-yl)-1-oxobut-2-en-2-yl)decanamide (**25c**).

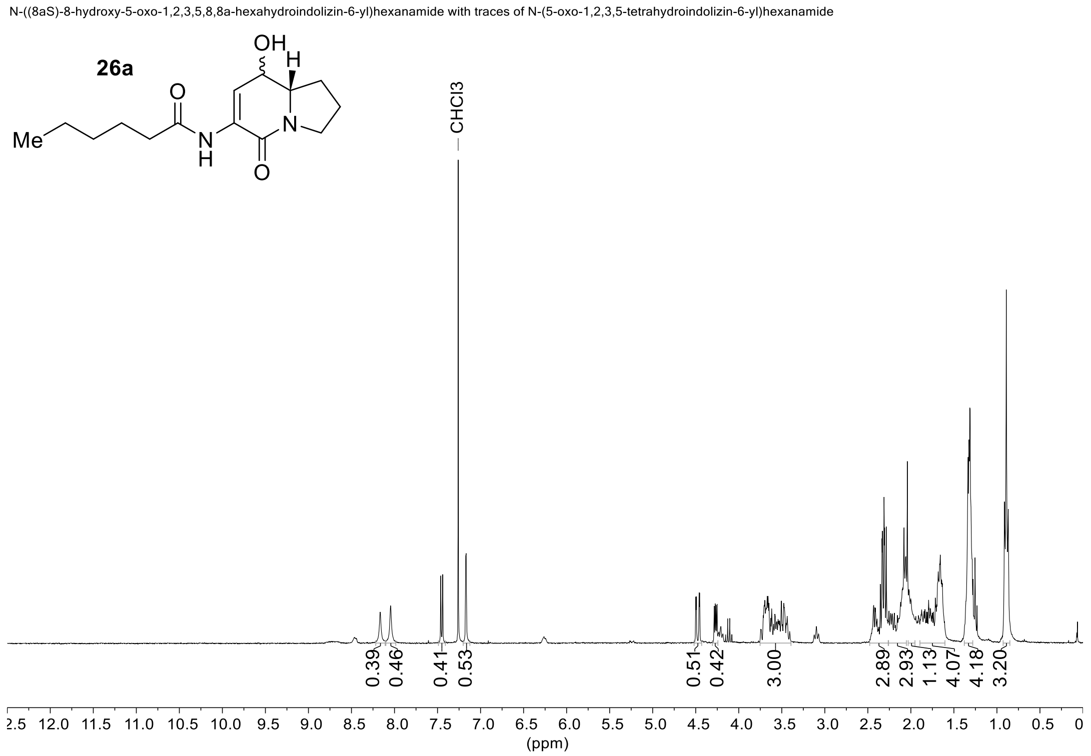

Figure S210. ^{1}H-NMR spectrum (600 MHz) of (*S*)-*N*-(8-hydroxy-5-oxo-1,2,3,5,8,8a-hexahydroindolizin-6-yl)hexanamide (**26a**) with traces of *N*-(5-oxo-1,2,3,5-tetrahydroindolizin-6-yl)hexanamide (**27a**).

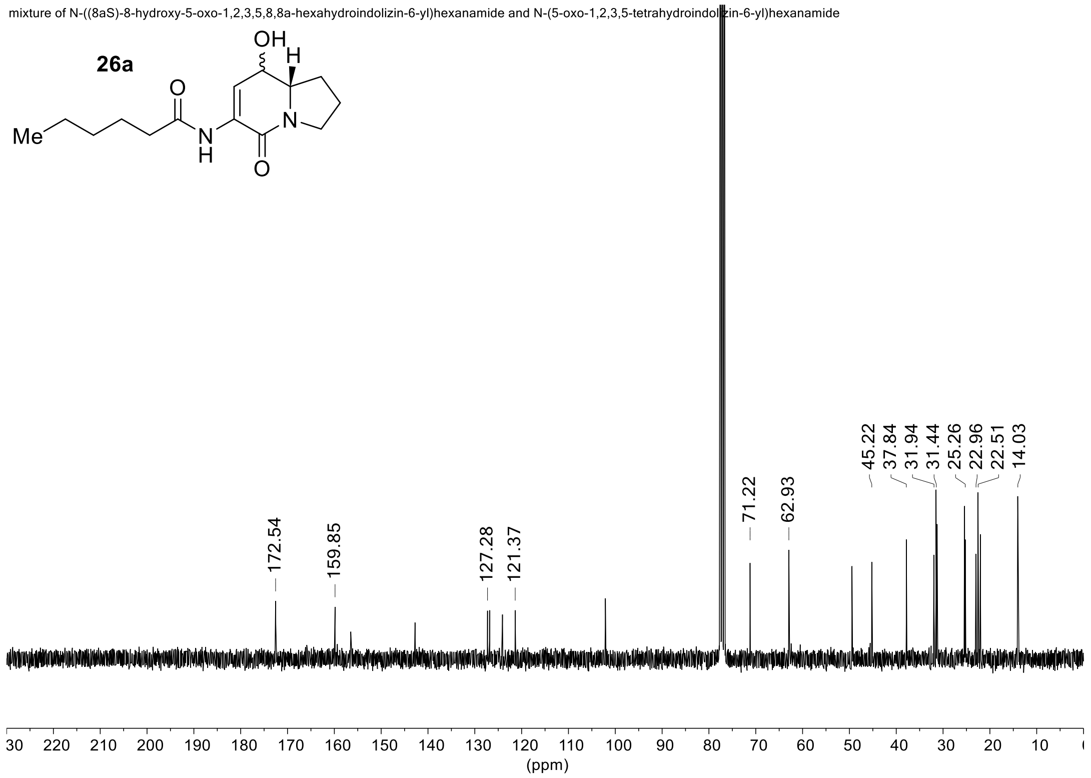

Figure S211. ^{13}C {^{1}H}-NMR spectrum (151 MHz) of (*S*)-*N*-(8-hydroxy-5-oxo-1,2,3,5,8,8a-hexahydroindolizin-6-yl)hexanamide (**26a**) and *N*-(5-oxo-1,2,3,5-tetrahydroindolizin-6-yl)hexanamide (**27a**), resulting from water elimination while standing in $CDCl_3$. Only the signals of the still present, stable isomer of **26a** are labeled.

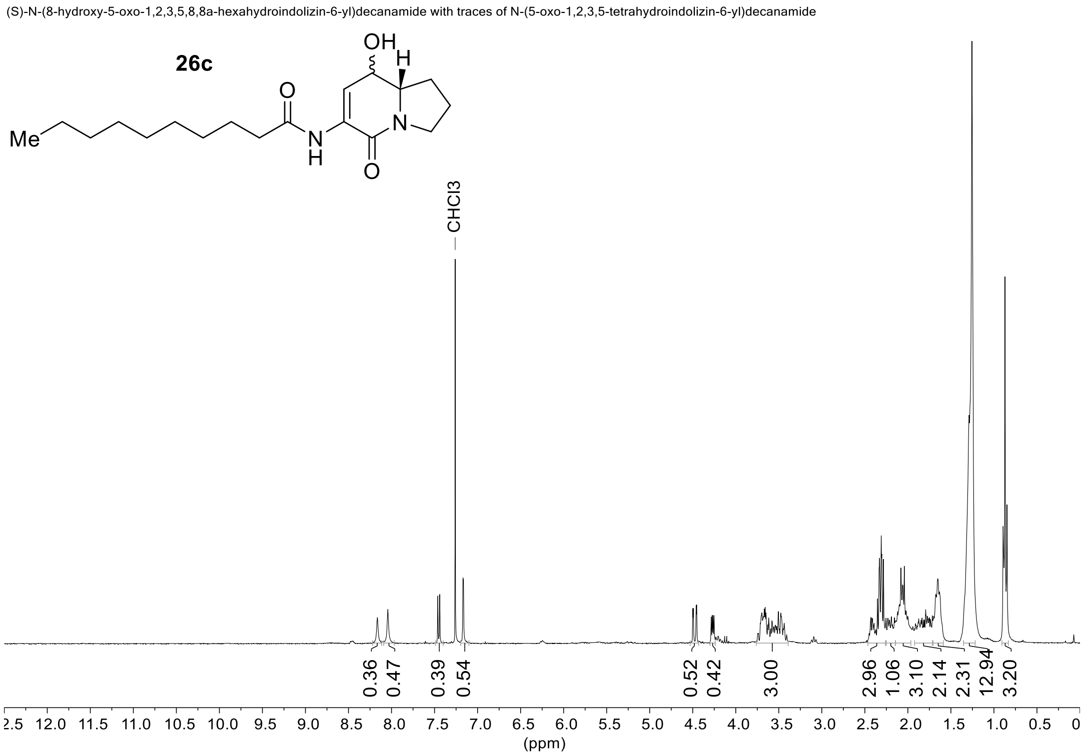

Figure S212. ^{1}H-NMR spectrum (300 MHz) of (*S*)-*N*-(8-hydroxy-5-oxo-1,2,3,5,8,8a-hexahydroindolizin-6-yl)decanamide (**26c**) with traces of *N*-(5-oxo-1,2,3,5-tetrahydroindolizin-6-yl)decanamide (**27c**).

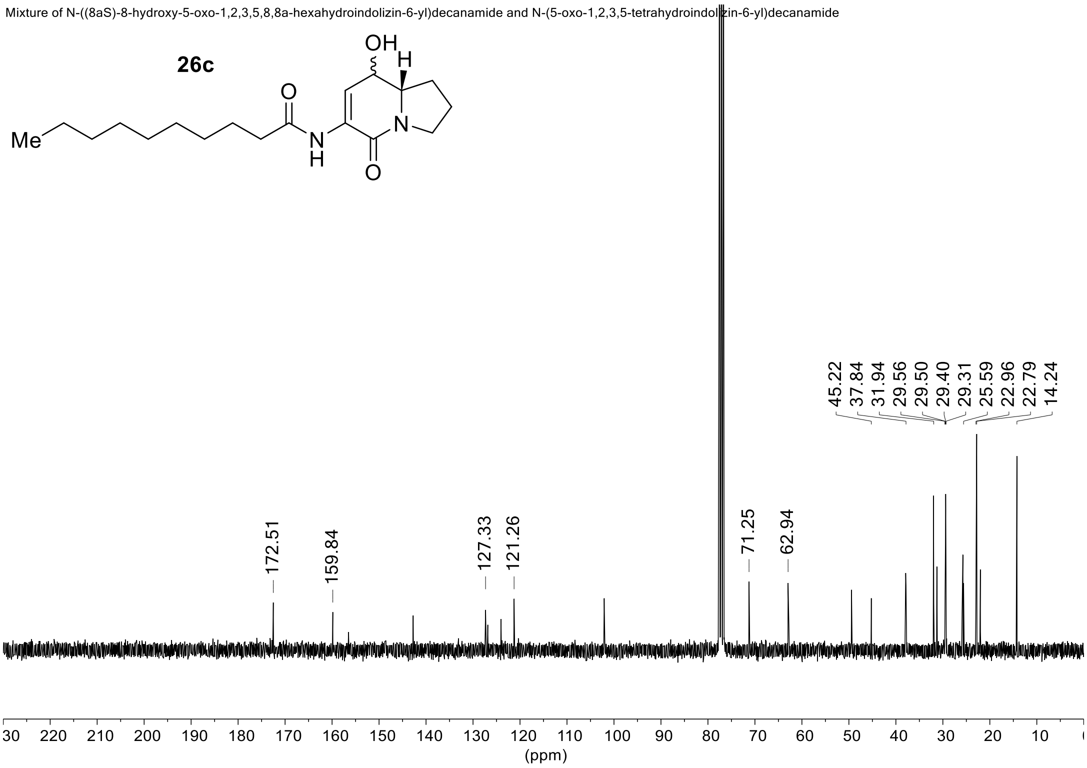

Figure S213. ^{13}C {^{1}H}-NMR spectrum (75.5 MHz) of (*S*)-*N*-(8-hydroxy-5-oxo-1,2,3,5,8,8a-hexahydroindolizin-6-yl)decanamide (**26c**) and *N*-(5-oxo-1,2,3,5-tetrahydroindolizin-6-yl)decanamide (**27c**), resulting from water elimination while standing in $CDCl_3$. Only the signals of the still present, stable isomer of **26c** are labeled.

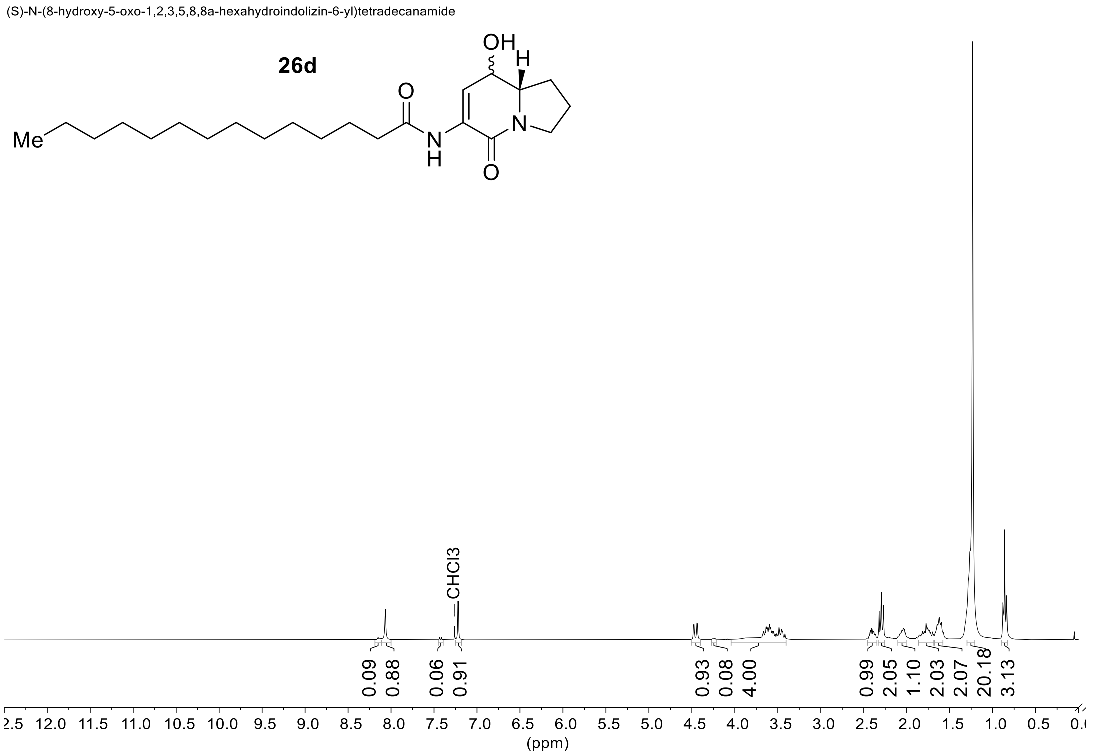

Figure S214. ^{1}H-NMR spectrum (300 MHz) of (*S*)-*N*-(8-hydroxy-5-oxo-1,2,3,5,8,8a-hexahydroindolizin-6-yl)tetradecanamide (**26d**).

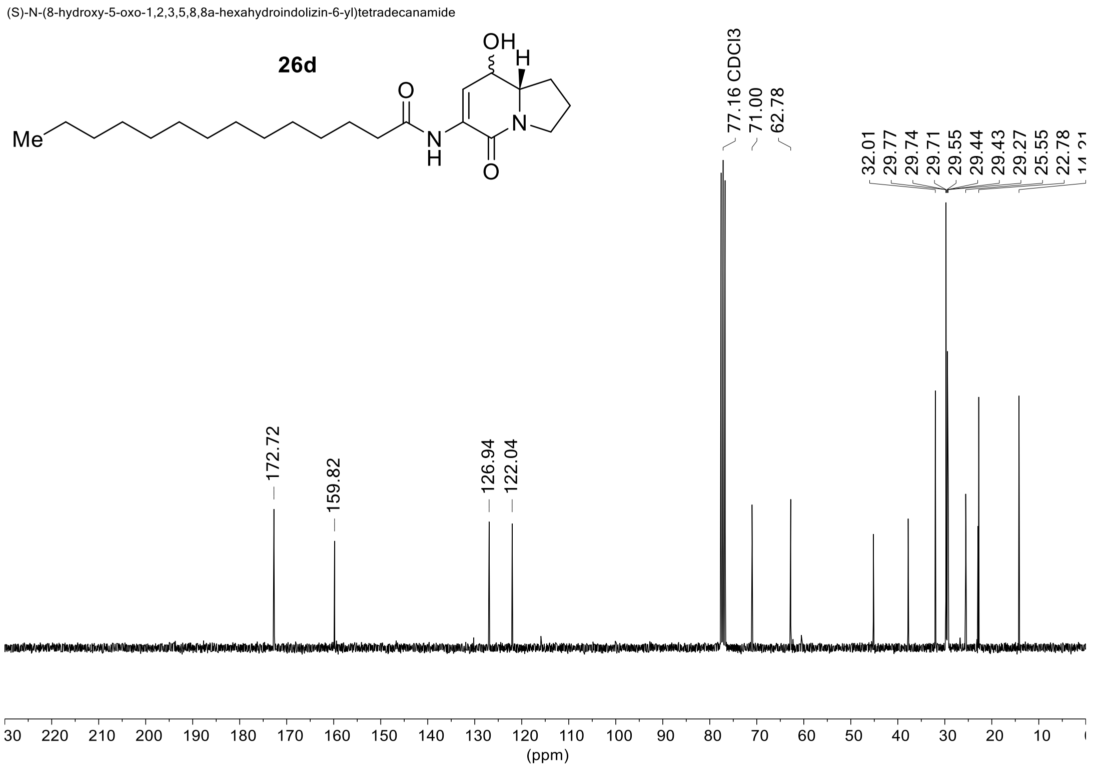

Figure S215. ^{13}C {^{1}H}-NMR spectrum (75.5 MHz) of (*S*)-*N*-(8-hydroxy-5-oxo-1,2,3,5,8,8a-hexahydroindolizin-6-yl)tetradecanamide (**26d**). Only the signals of the still present, stabile isomer of **26d** are labeled.

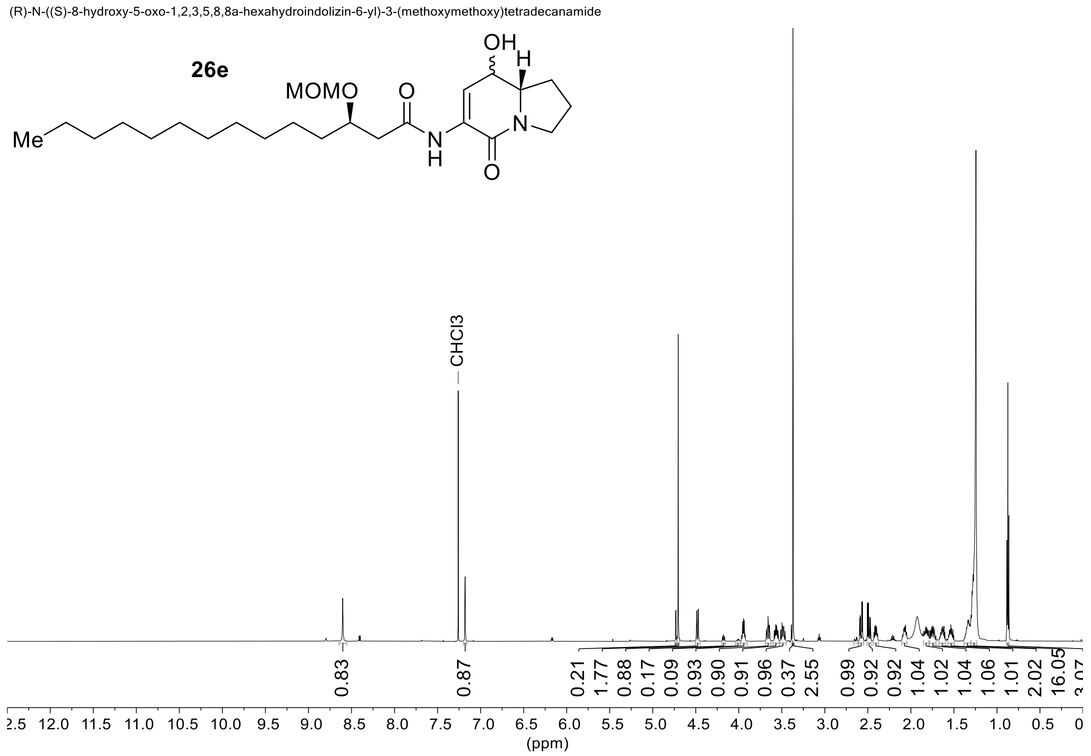

Figure S216. ^{1}H-NMR spectrum (600 MHz) of (*R*)-*N*-((*S*)-8-hydroxy-5-oxo-1,2,3,5,8,8a-hexahydroindolizin-6-yl)-3-(methoxymethoxy)tetradecanamide (**26e**).

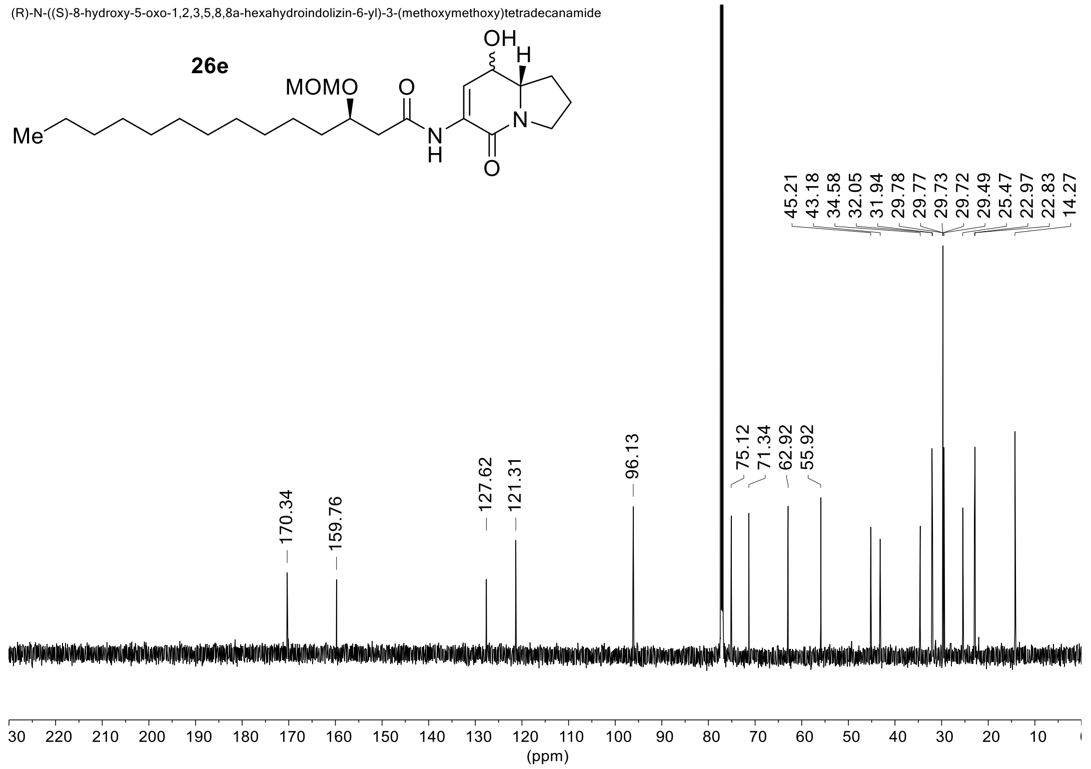

Figure S217. ^{13}C {^{1}H}-NMR spectrum (151 MHz) of (*R*)-*N*-((*S*)-8-hydroxy-5-oxo-1,2,3,5,8,8a-hexahydroindolizin-6-yl)-3-(methoxymethoxy)tetradecanamide (**26e**).

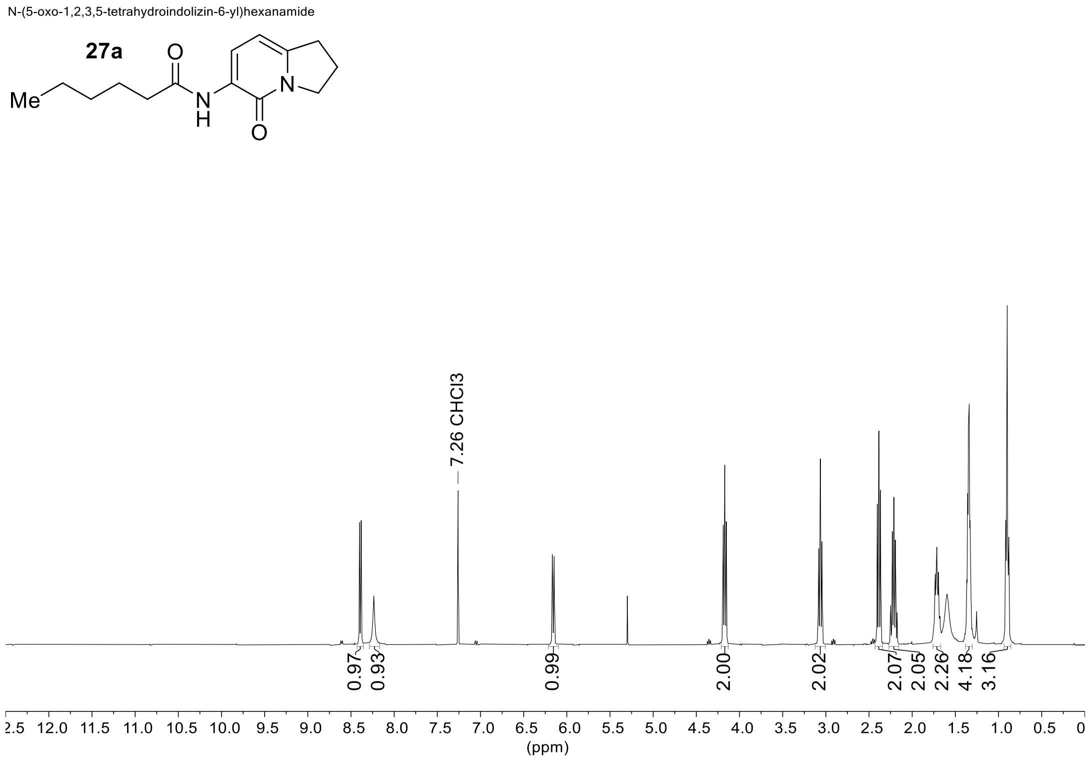

Figure S218. ^{1}H-NMR spectrum (400 MHz) of *N*-(5-oxo-1,2,3,5-tetrahydroindolizin-6-yl)hexanamide (**27a**).

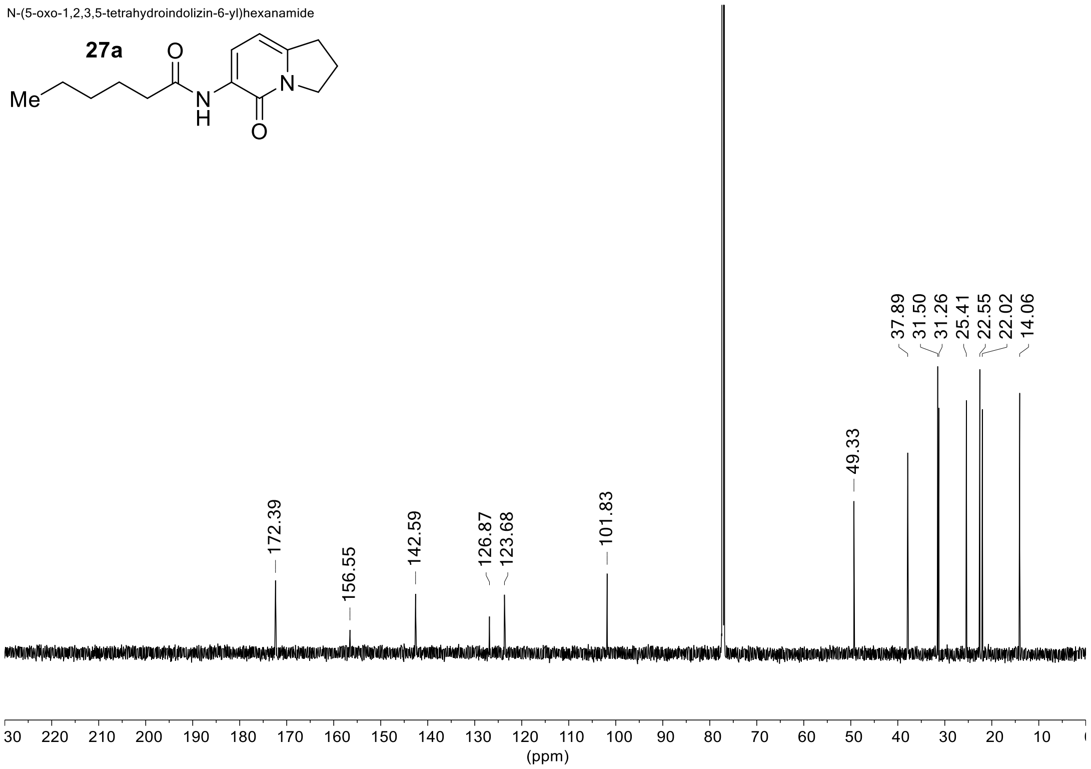

Figure S219. ^{13}C {^{1}H}-NMR spectrum (151 MHz) of *N*-(5-oxo-1,2,3,5-tetrahydroindolizin-6-yl)hexanamide (**27a**).

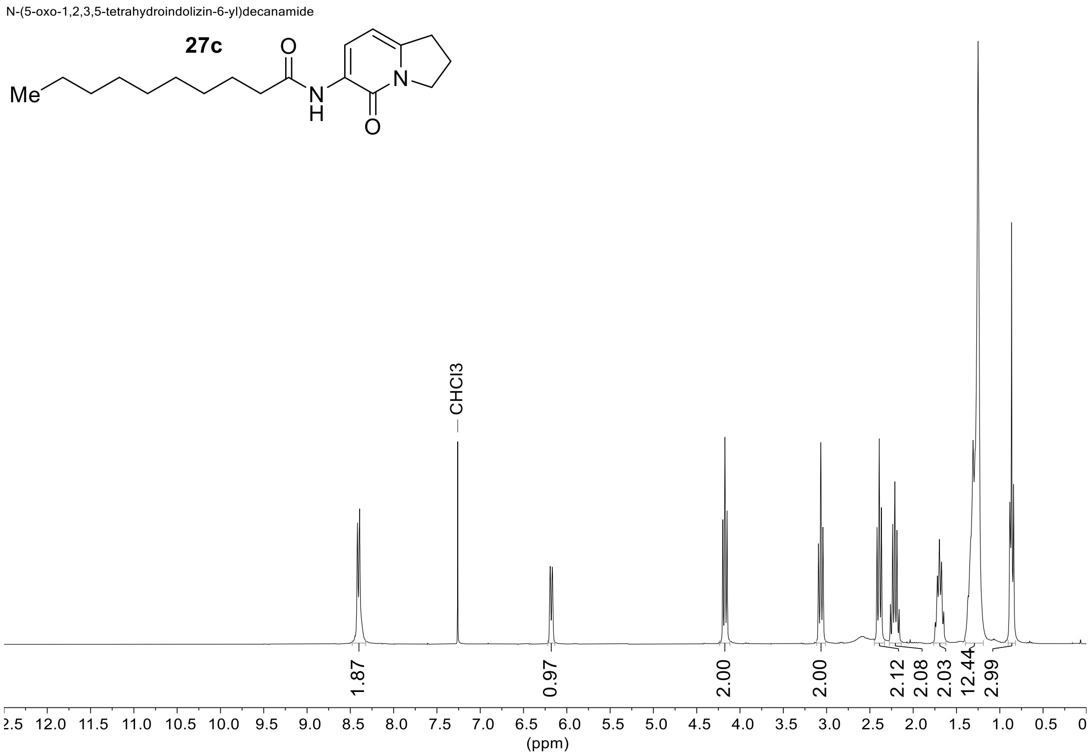

Figure S220. ^{1}H-NMR spectrum (300 MHz) of *N*-(5-oxo-1,2,3,5-tetrahydroindolizin-6-yl)decanamide (**27c**).

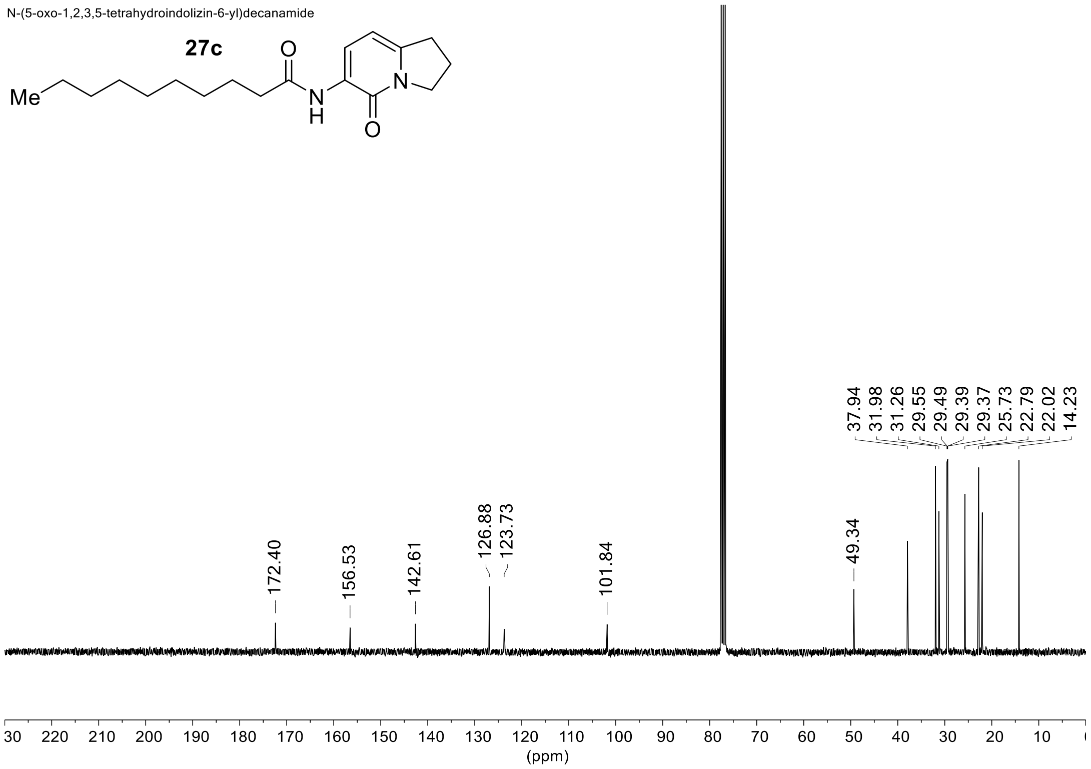

Figure S221. ^{13}C {^{1}H}-NMR spectrum (75.5 MHz) of *N*-(5-oxo-1,2,3,5-tetrahydroindolizin-6-yl)decanamide (**27c**).

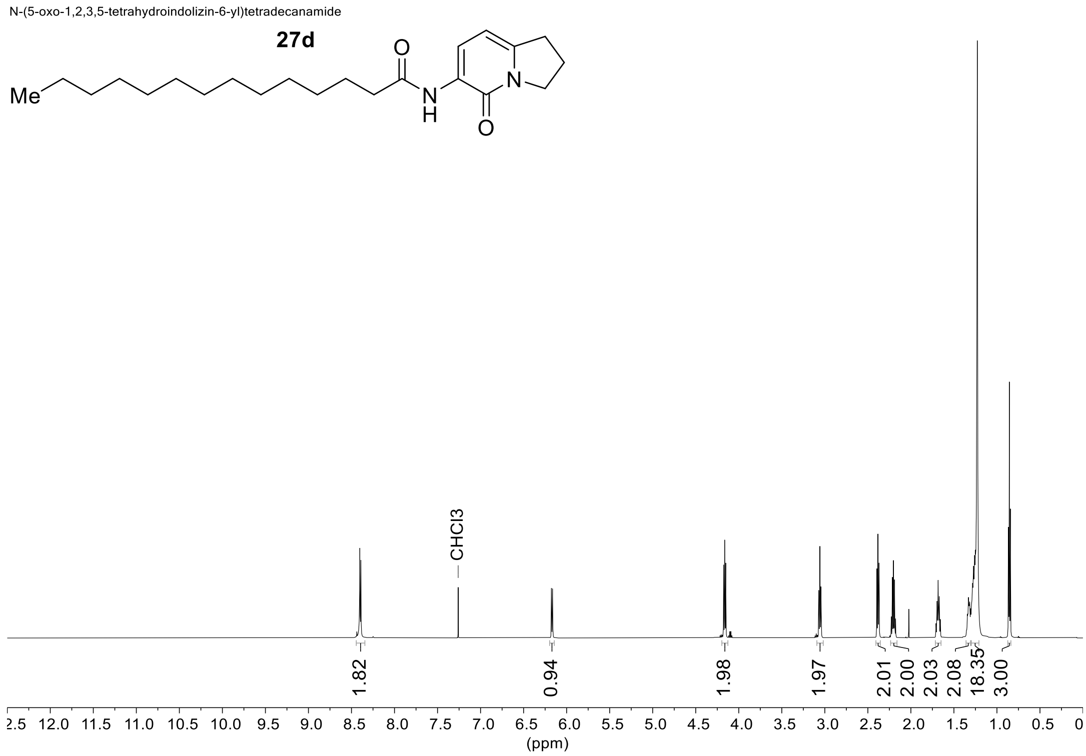

Figure S222. ^{1}H-NMR spectrum (600 MHz) of *N*-(5-oxo-1,2,3,5-tetrahydroindolizin-6-yl)tetradecanamide (**27d**).

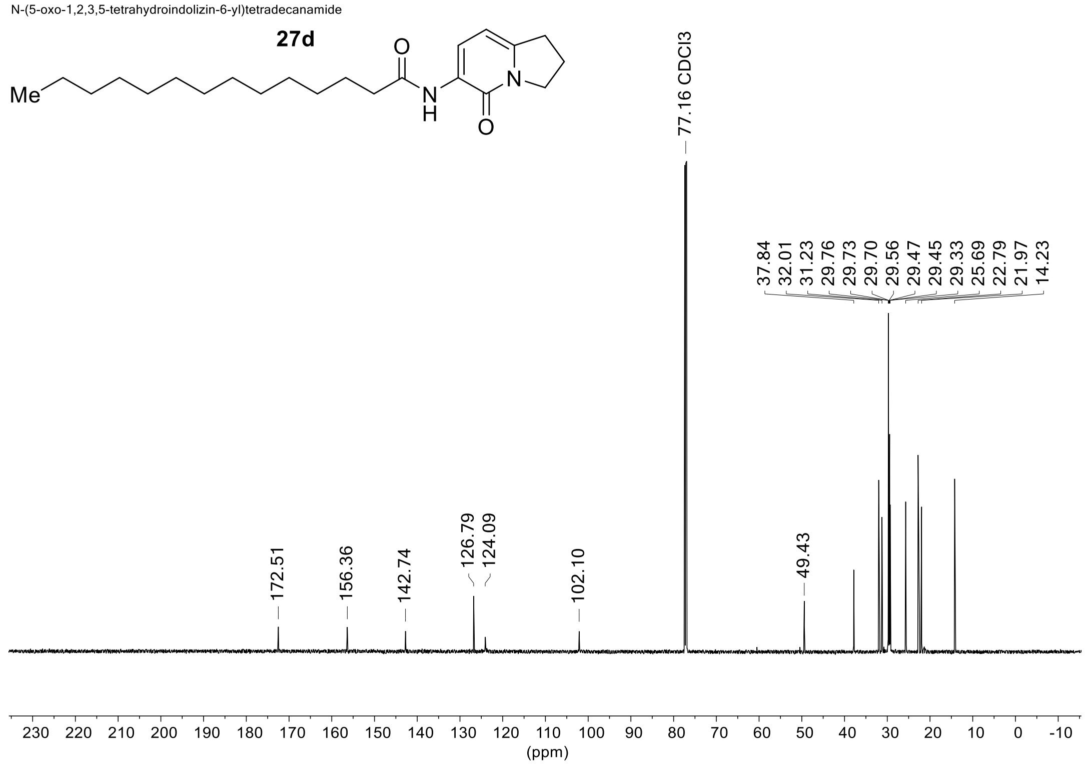

Figure S223. ^{13}C {^{1}H}-NMR spectrum (151 MHz) of *N*-(5-oxo-1,2,3,5-tetrahydroindolizin-6-yl)tetradecanamide (**27d**).

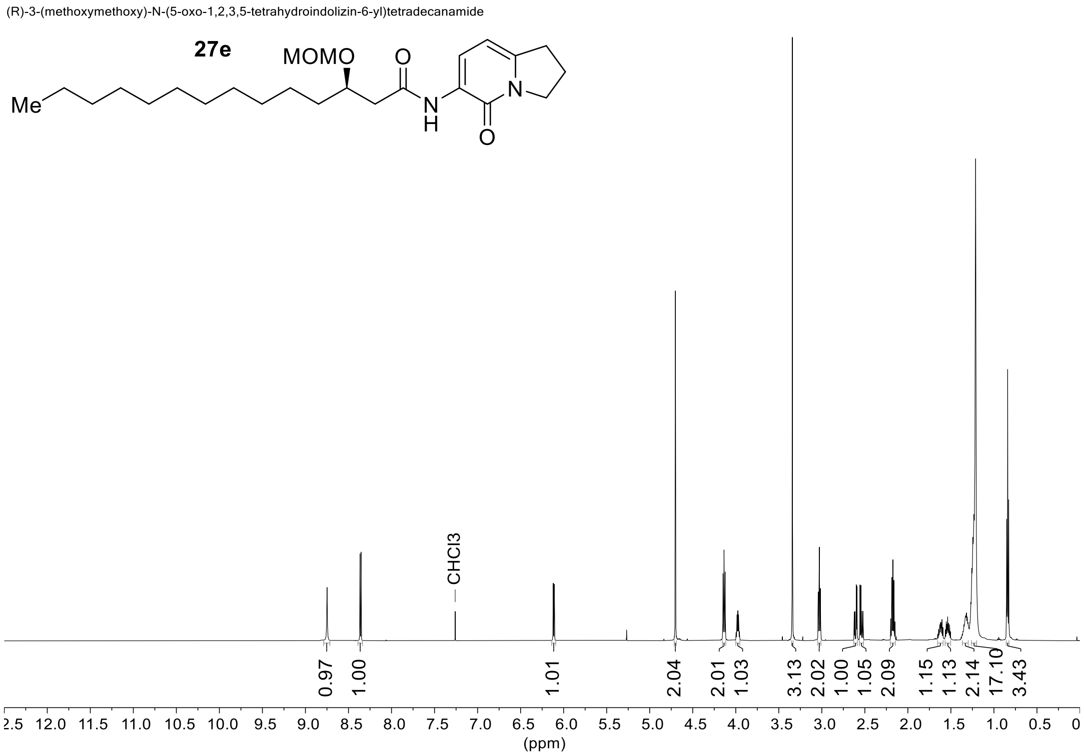

Figure S224. ^{1}H-NMR spectrum (600 MHz) of (*R*)-3-(methoxymethoxy)-*N*-(5-oxo-1,2,3,5-tetrahydroindolizin-6-yl)tetradecanamide (**27e**).

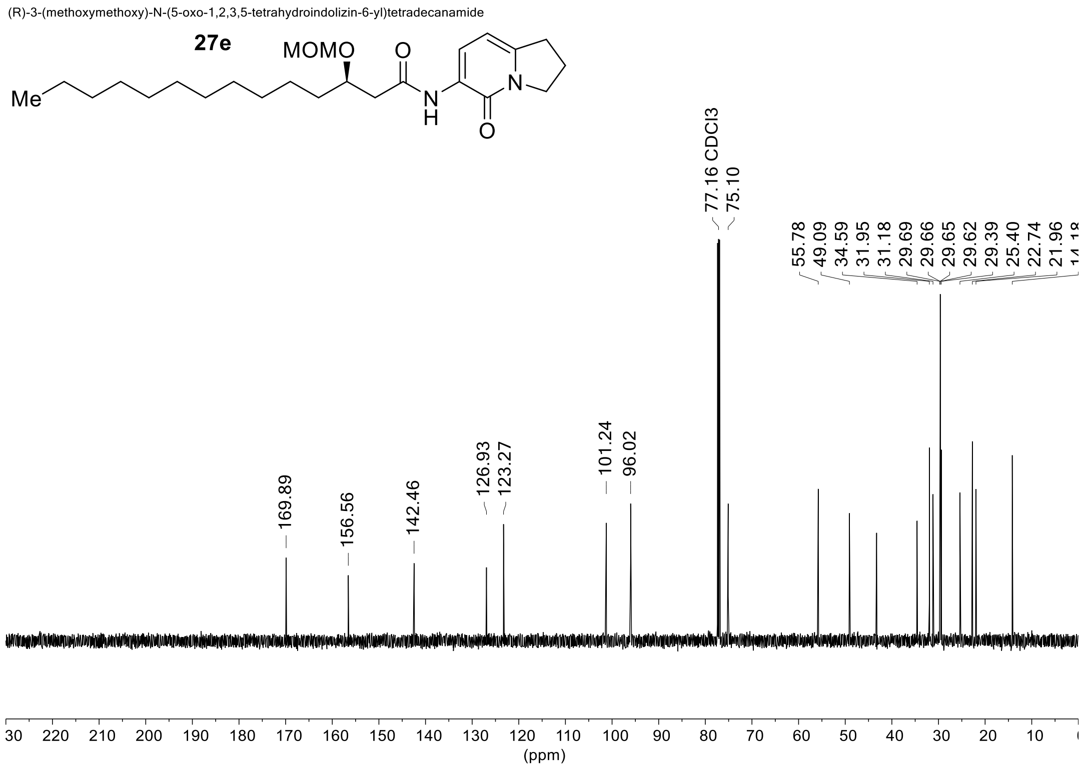

Figure S225. ^{13}C {^{1}H}-NMR spectrum (151 MHz) of (*R*)-3-(methoxymethoxy)-*N*-(5-oxo-1,2,3,5-tetrahydroindolizin-6-yl)tetradecanamide (**27e**).

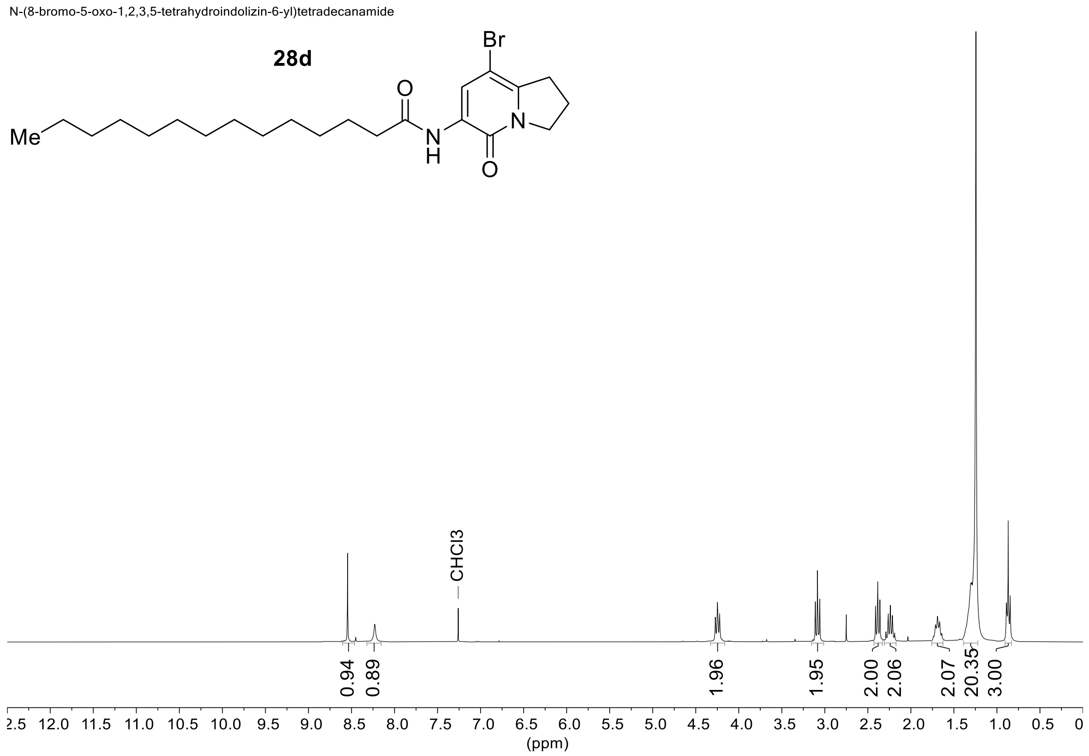

Figure S226. ^{1}H-NMR spectrum (300 MHz) of *N*-(8-bromo-5-oxo-1,2,3,15-tetrahydroindolizin-6-yl)tetradecanamide (**28d**).

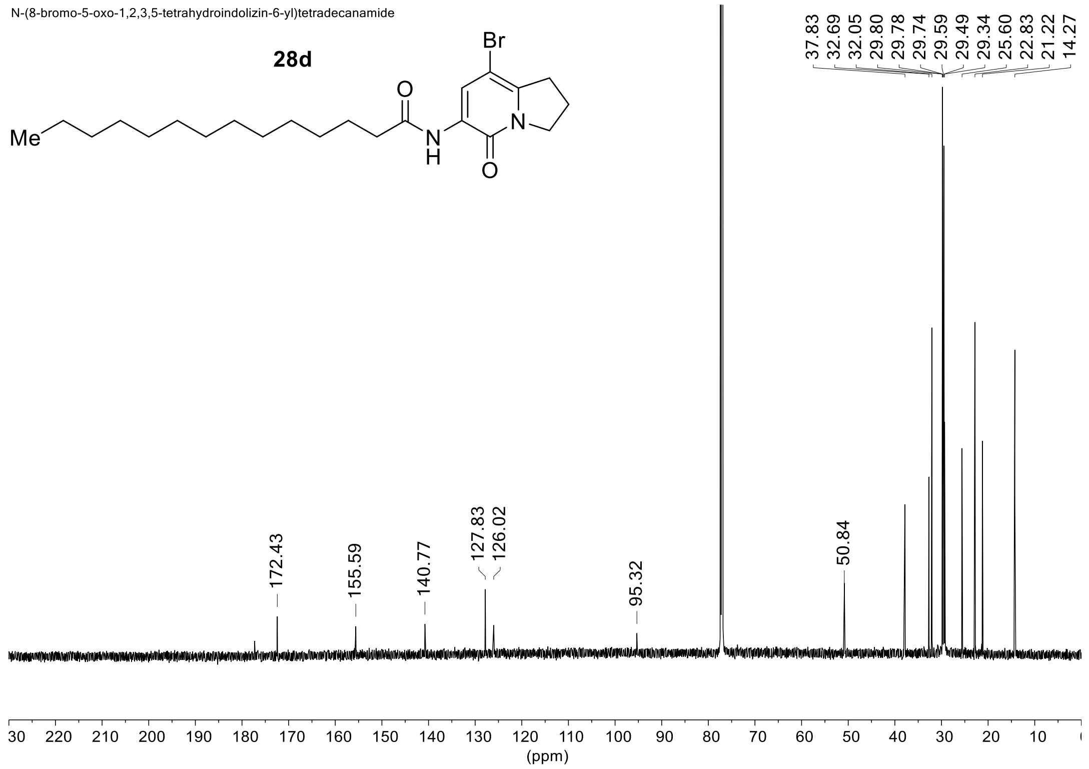

Figure S227. ^{13}C {^{1}H}-NMR spectrum (75.5 MHz) of *N*-(8-bromo-5-oxo-1,2,3,5-tetrahydroindolizin-6-yl)tetradecanamide (**28d**).

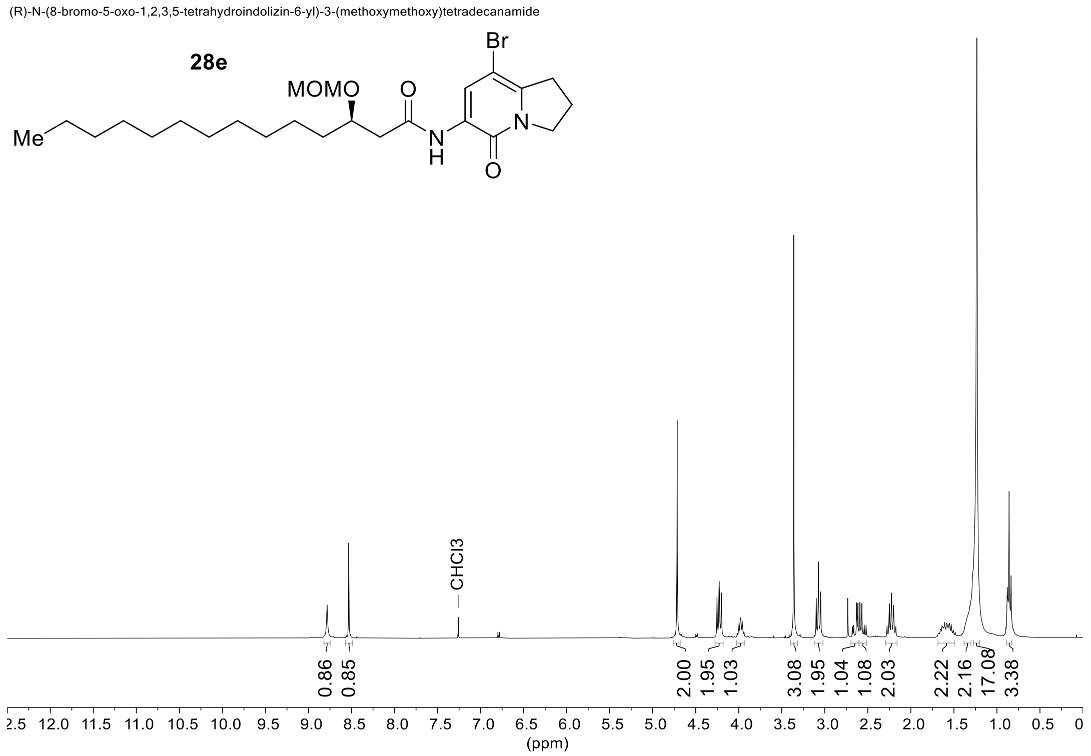

Figure S228. ^{1}H-NMR spectrum (300 MHz) of (*R*)-*N*-(8-bromo-5-oxo-1,2,3,5-tetrahydroindolizin-6-yl)-3-(methoxymethoxy)tetradecanamide (**28e**).

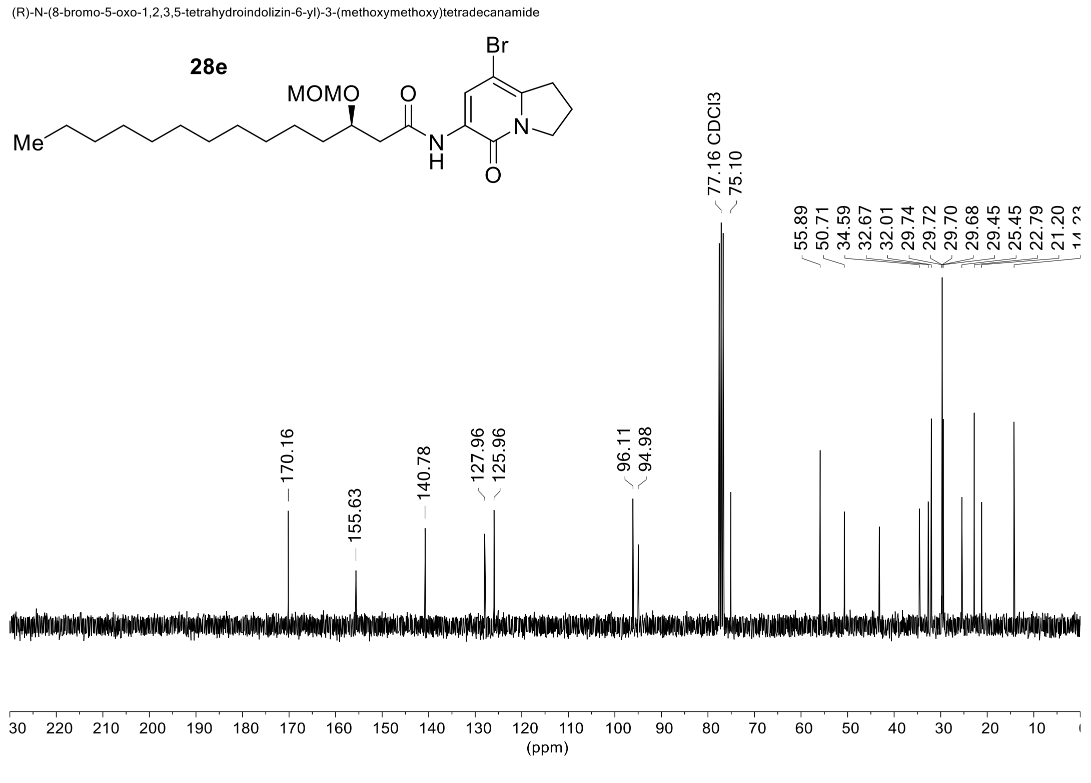

Figure S229. ^{13}C {^{1}H}-NMR spectrum (75.5 MHz) of (*R*)-*N*-(8-bromo-5-oxo-1,2,3,5-tetrahydroindolizin-6-yl)-3-(methoxymethoxy)tetradecanamide (**28e**).

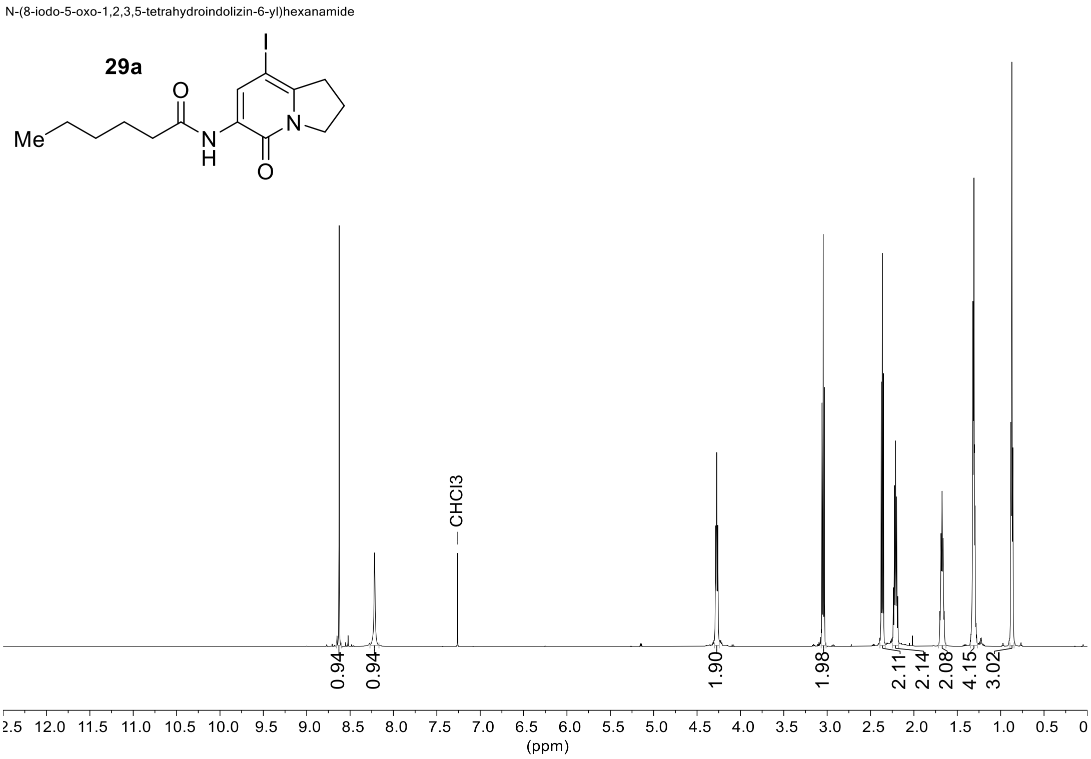

Figure S230. ^{1}H-NMR spectrum (300 MHz) of *N*-(8-iodo-5-oxo-1,2,3,5-tetrahydroindolizin-6-yl)hexanamide (**29a**).

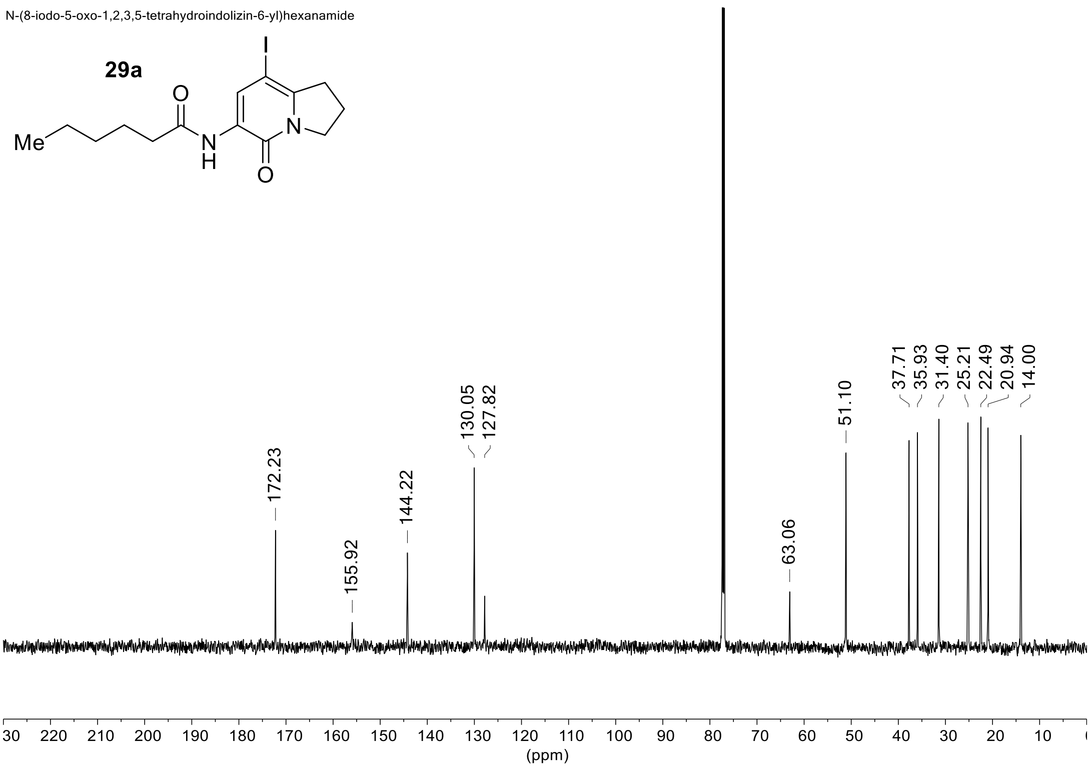

Figure S231. ^{13}C {^{1}H}-NMR spectrum (75.5 MHz) of *N*-(8-iodo-5-oxo-1,2,3,5-tetrahydroindolizin-6-yl)tetradecanamide (**29a**).

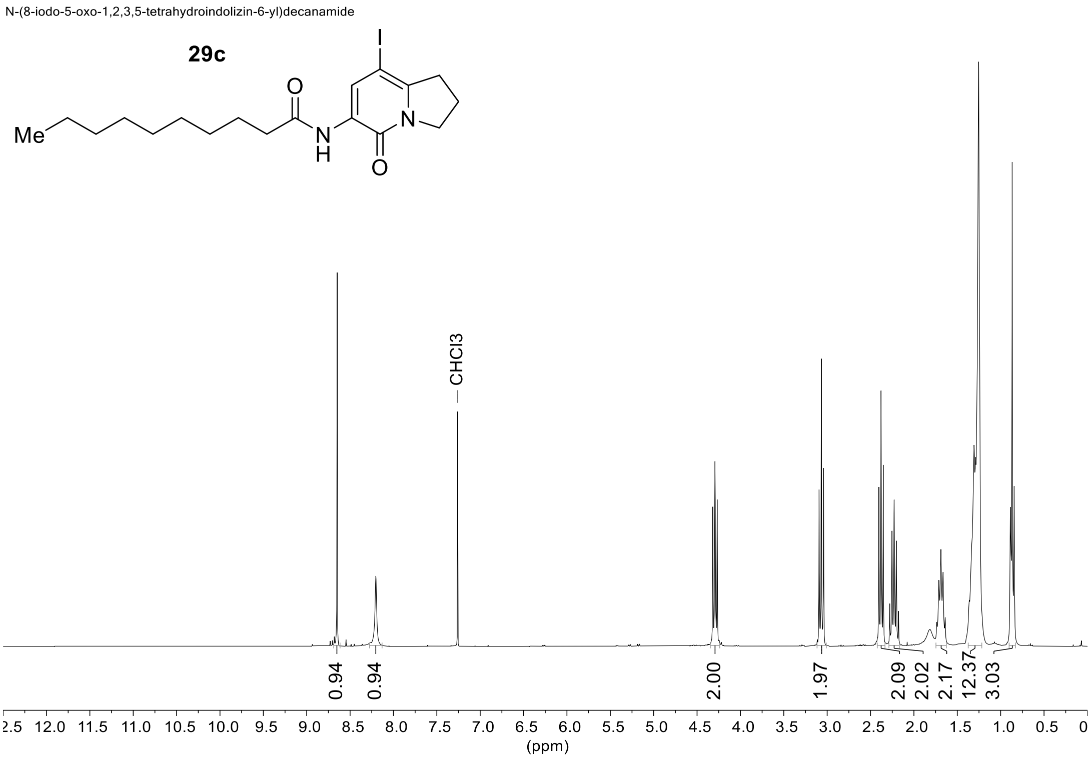

Figure S232. ^{1}H-NMR spectrum (300 MHz) of *N*-(8-iodo-5-oxo-1,2,3,5-tetrahydroindolizin-6-yl)decanamide (**29c**).

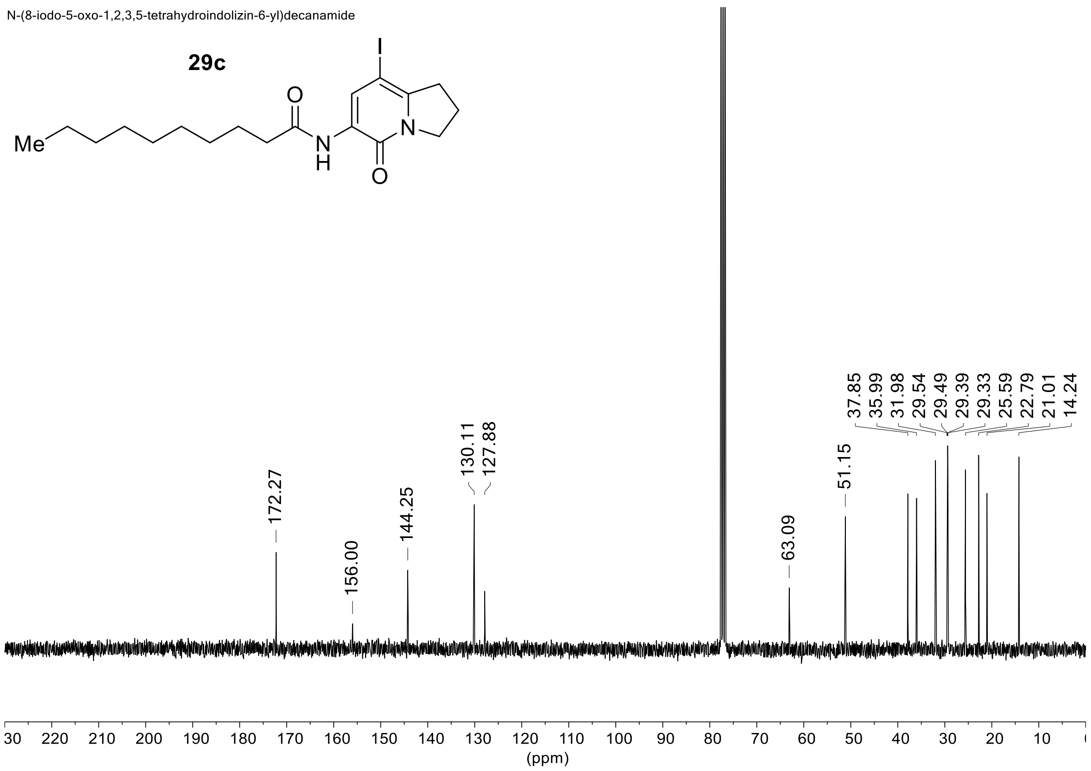

Figure S233. ^{13}C {^{1}H}-NMR spectrum (75.5 MHz) of *N*-(8-iodo-5-oxo-1,2,3,5-tetrahydroindolizin-6-yl)decanamide (**29c**).

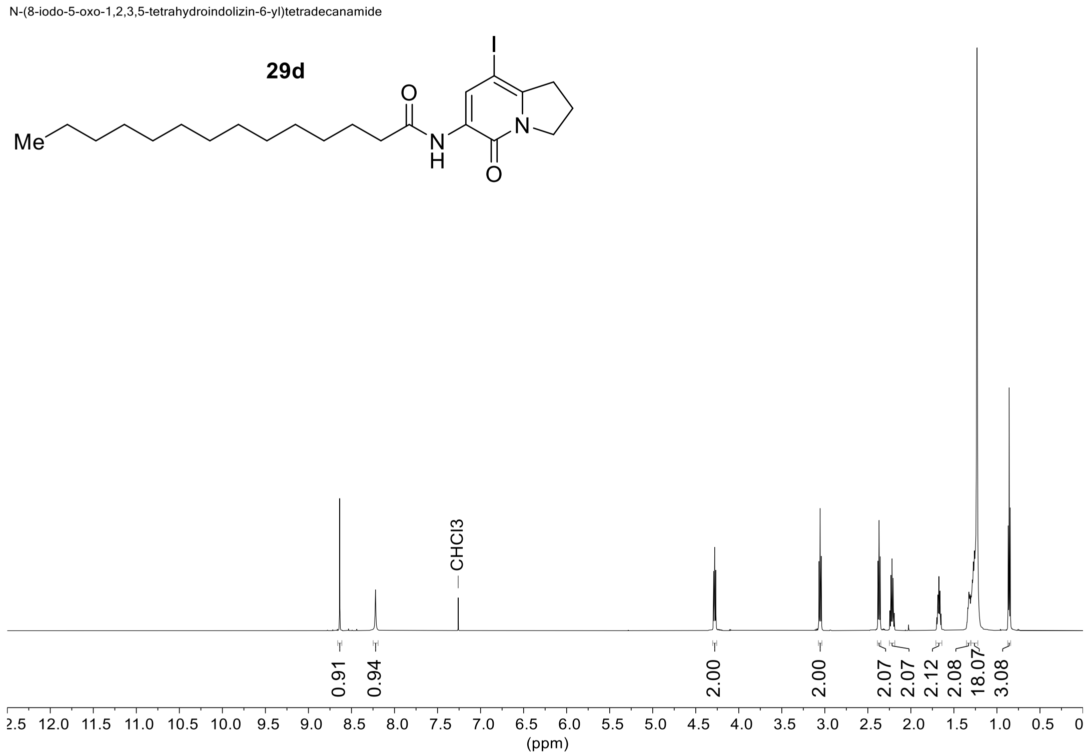

Figure S234. ^{1}H-NMR spectrum (600 MHz) of *N*-(8-iodo-5-oxo-1,2,3,5-tetrahydroindolizin-6-yl)tetradecanamide (**29d**).

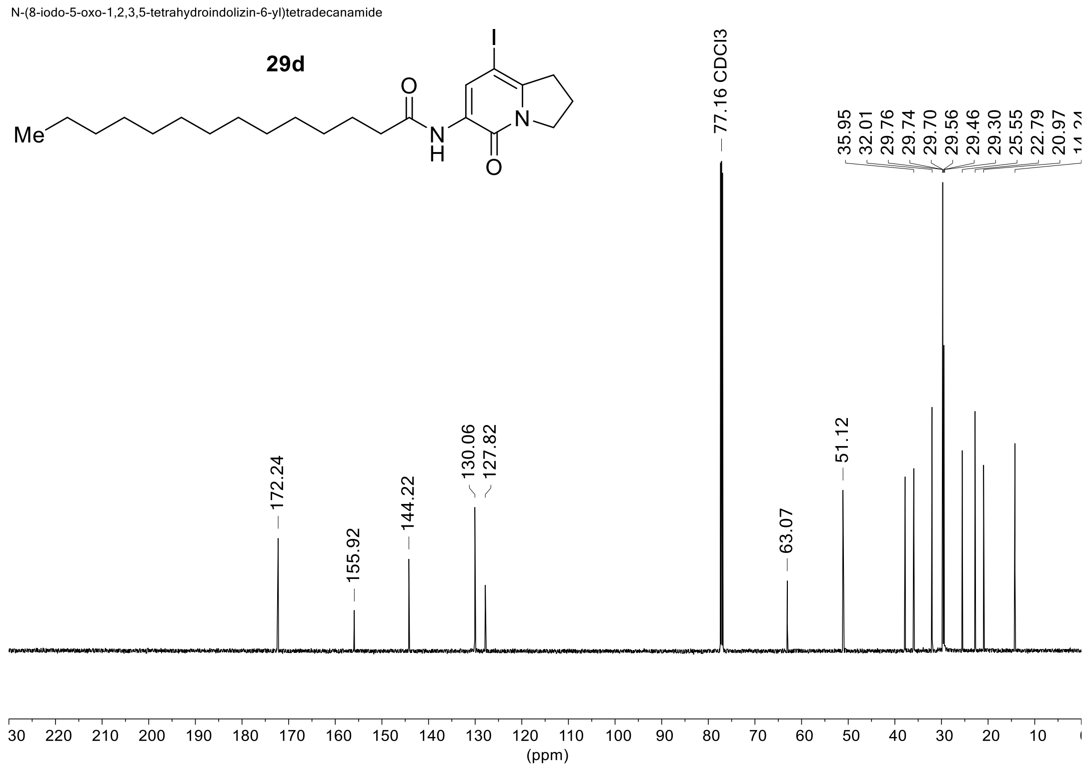

Figure S235. ^{13}C {^{1}H}-NMR spectrum (151 MHz) of *N*-(8-iodo-5-oxo-1,2,3,5-tetrahydroindolizin-6-yl)tetradecanamide (**29d**).

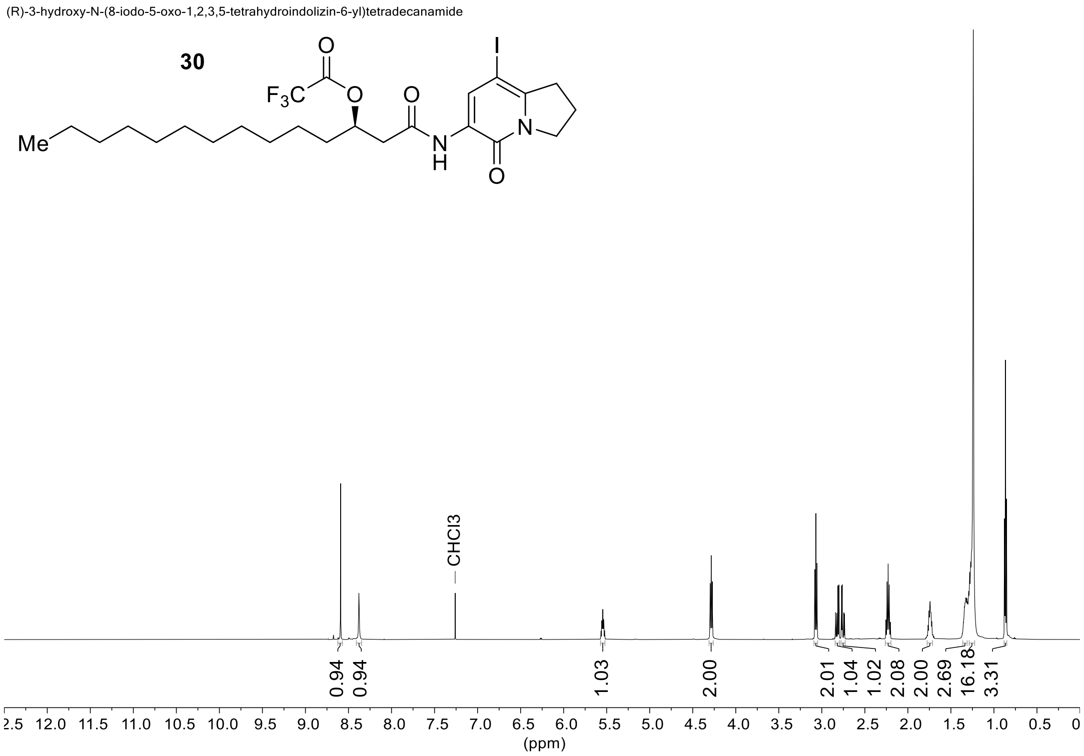

Figure S236. ^{1}H-NMR spectrum (600 MHz) of (*R*)-1-((8-iodo-5-oxo-1,2,3,5-tetrahydroindolizin-6-yl)amino)-1-oxotetradecan-3-yl 2,2,2-trifluoroacetate (**30**).

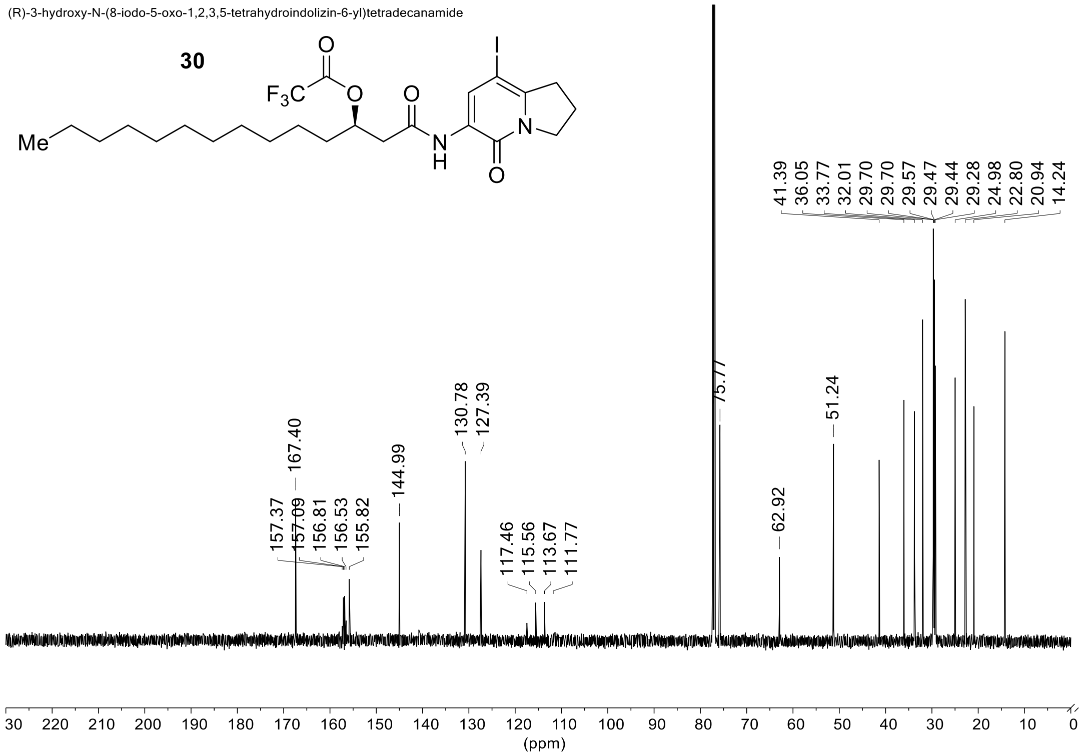

Figure S237. ^{13}C {^{1}H}-NMR spectrum (151 MHz) of (*R*)-1-((8-iodo-5-oxo-1,2,3,5-tetrahydroindolizin-6-yl)amino)-1-oxotetradecan-3-yl 2,2,2-trifluoroacetate (**30**).

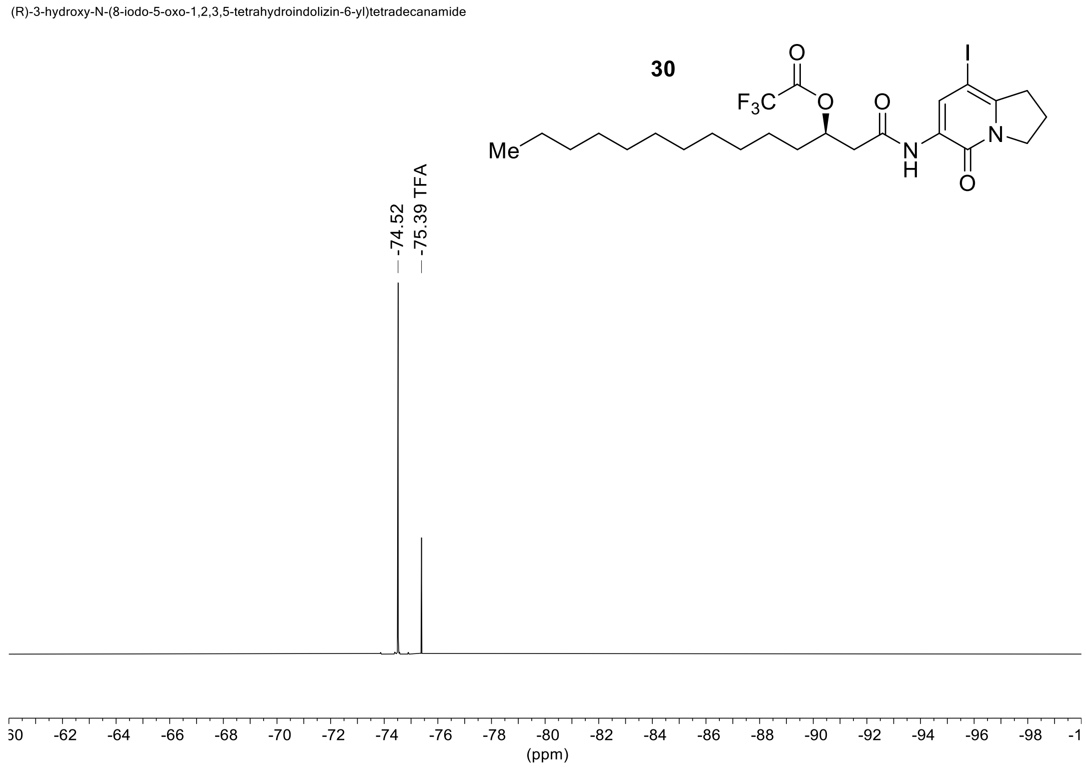

Figure S238. ^{19}F {^{1}H}-NMR spectrum (282 MHz) of (*R*)-1-((8-iodo-5-oxo-1,2,3,5-tetrahydroindolizin-6-yl)amino)-1-oxotetradecan-3-yl 2,2,2-trifluoroacetate (**30**).

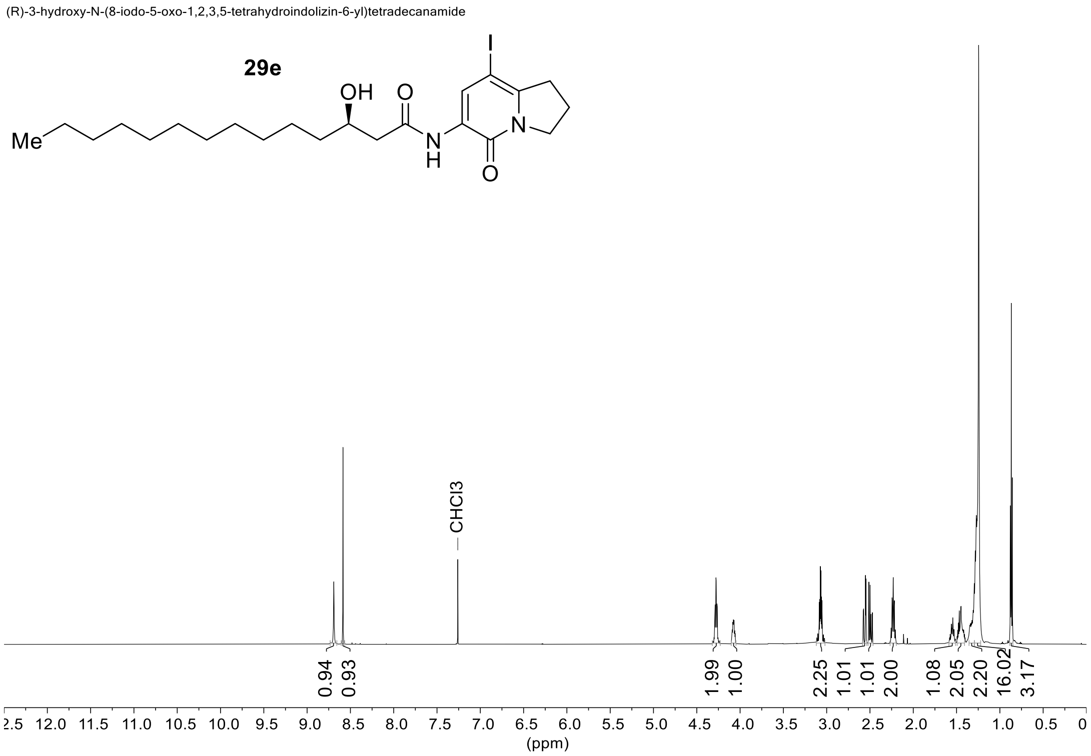

Figure S239. ^{1}H-NMR spectrum (600 MHz) of (*R*)-3-hydroxy-*N*-(8-iodo-5-oxo-1,2,3,5-tetrahydroindolizin-6-yl)tetradecanamide (**29e**).

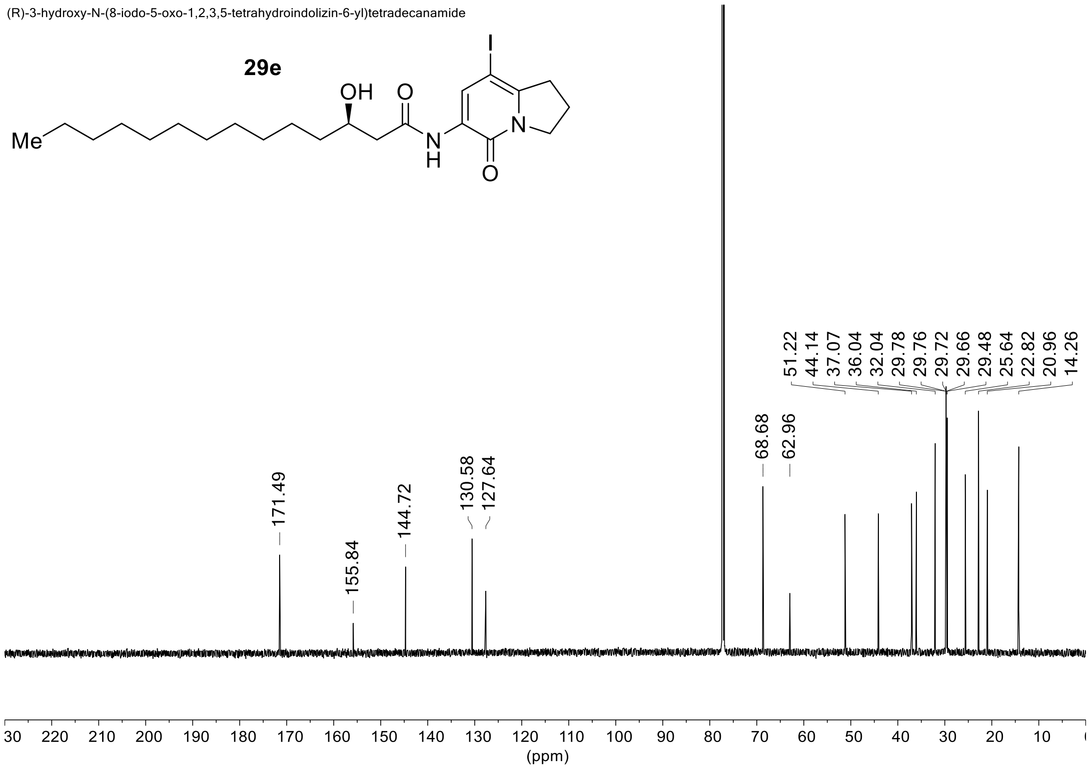

Figure S240. ^{13}C {^{1}H}-NMR spectrum (151 MHz) of (*R*)-3-hydroxy-*N*-(8-iodo-5-oxo-1,2,3,5-tetrahydroindolizin-6-yl)tetradecanamide (**29e**).

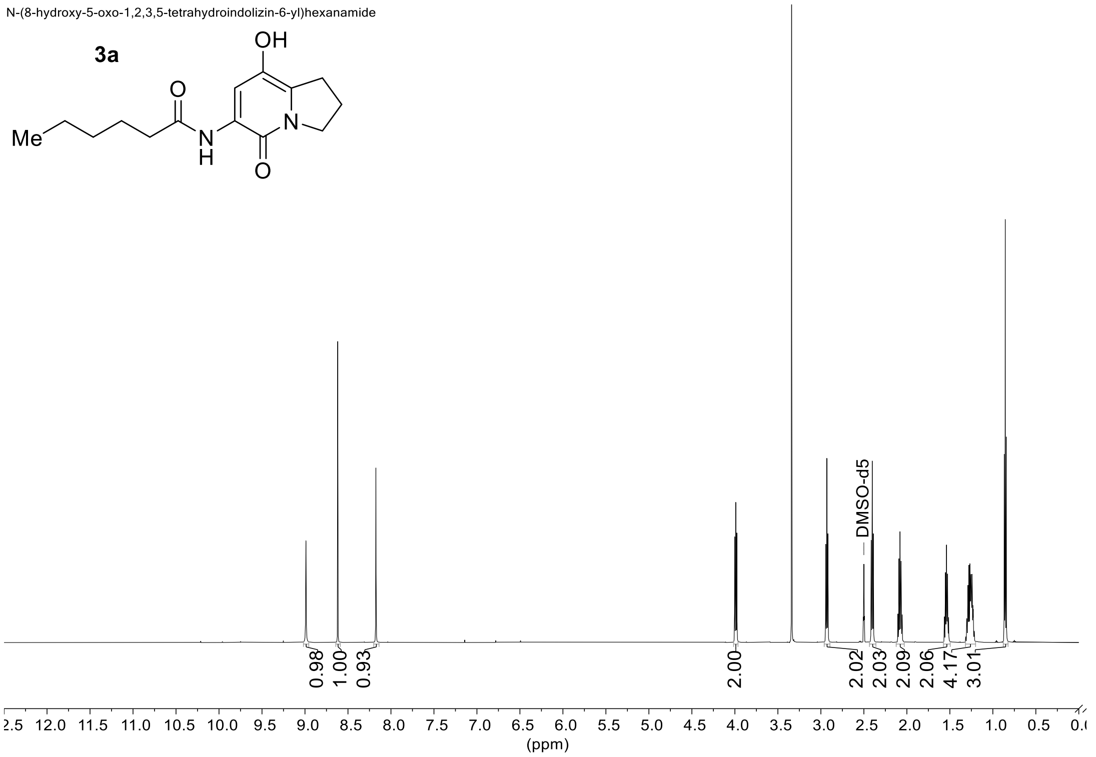

Figure S241. ^{1}H-NMR spectrum (600 MHz) of *N*-(8-hydroxy-5-oxo-1,2,3,5-tetrahydroindolizin-6-yl)hexanamide (**3a**).

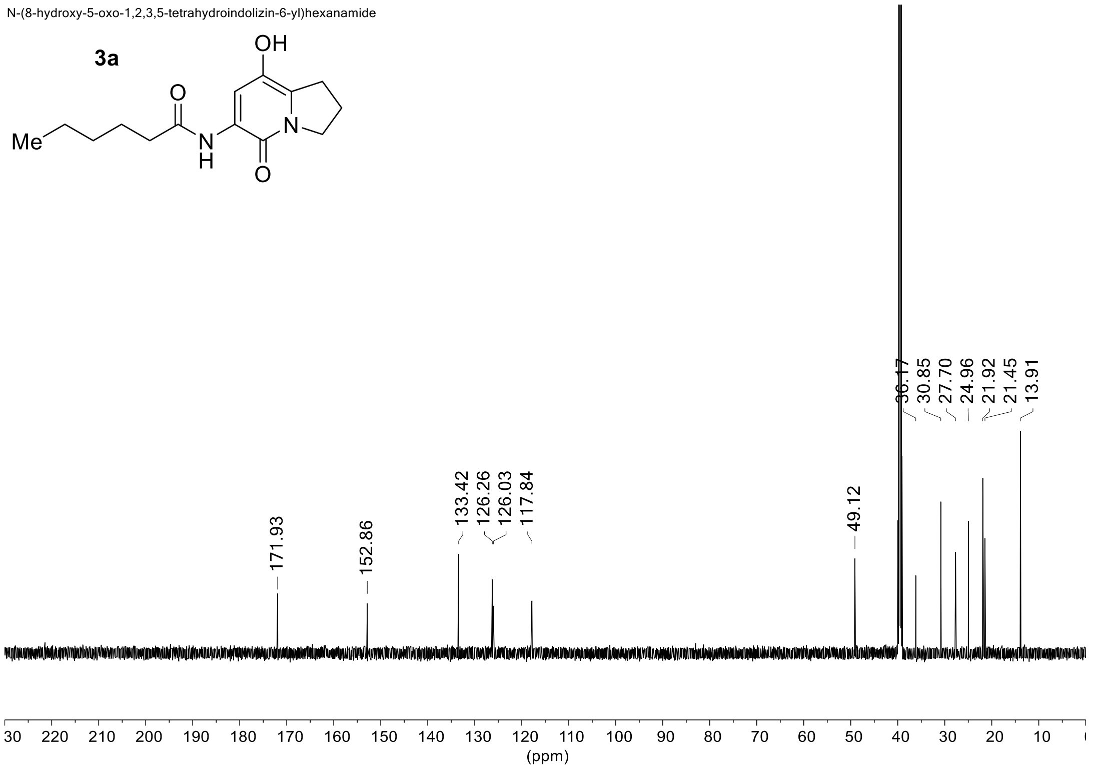

Figure S242. ^{13}C {^{1}H}-NMR spectrum (151 MHz) of *N*-(8-hydroxy-5-oxo-1,2,3,5-tetrahydroindolizin-6-yl)hexanamide (**3a**).

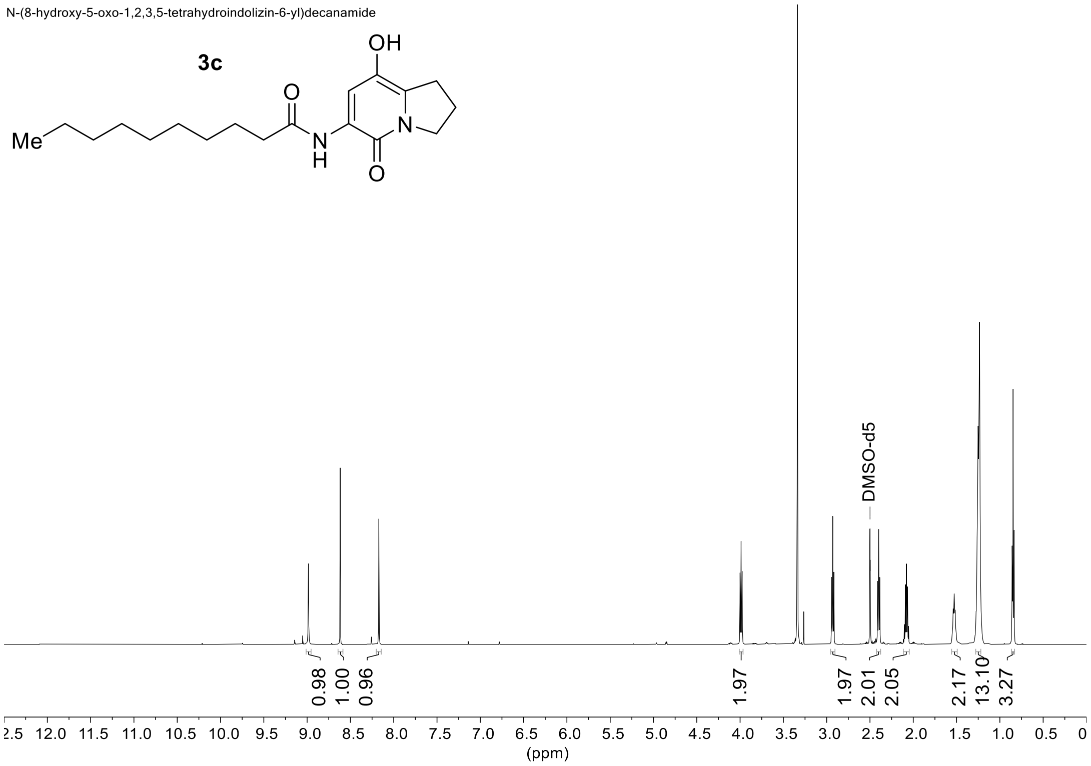

Figure S243. ^{1}H-NMR spectrum (600 MHz) of *N*-(8-hydroxy-5-oxo-1,2,3,5-tetrahydroindolizin-6-yl)decanamide (**3c**).

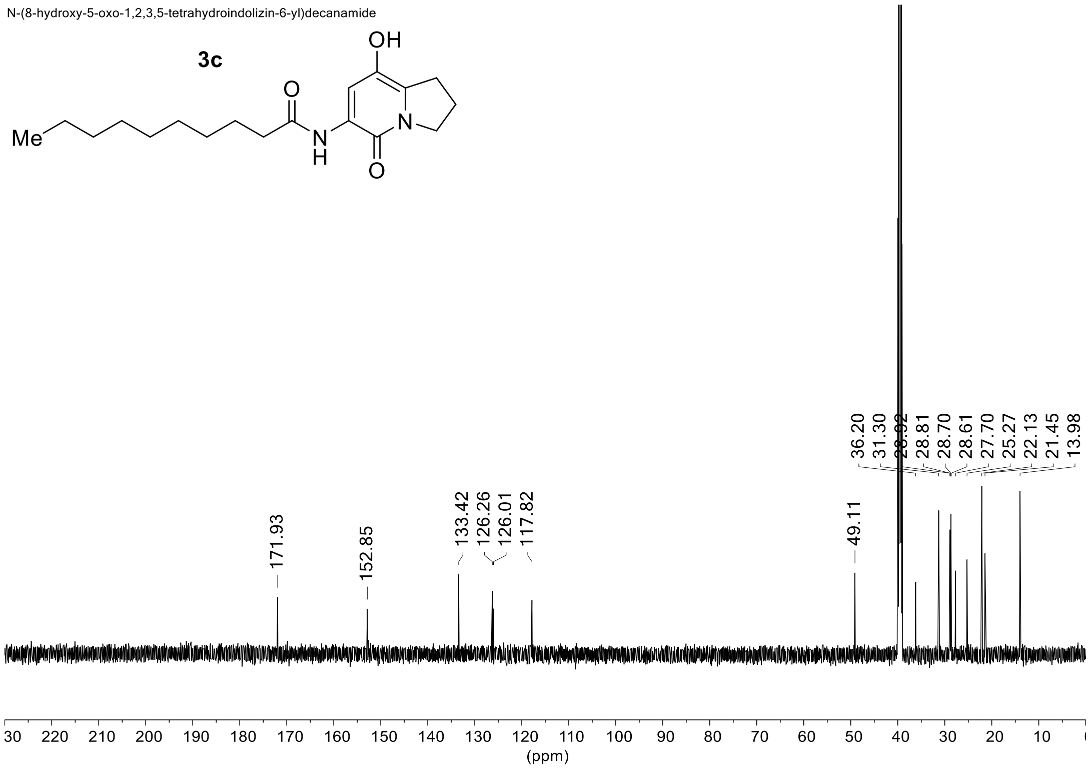

Figure S244. ^{13}C {^{1}H}-NMR spectrum (151 MHz) of *N*-(8-hydroxy-5-oxo-1,2,3,5-tetrahydroindolizin-6-yl)decanamide (**3c**).

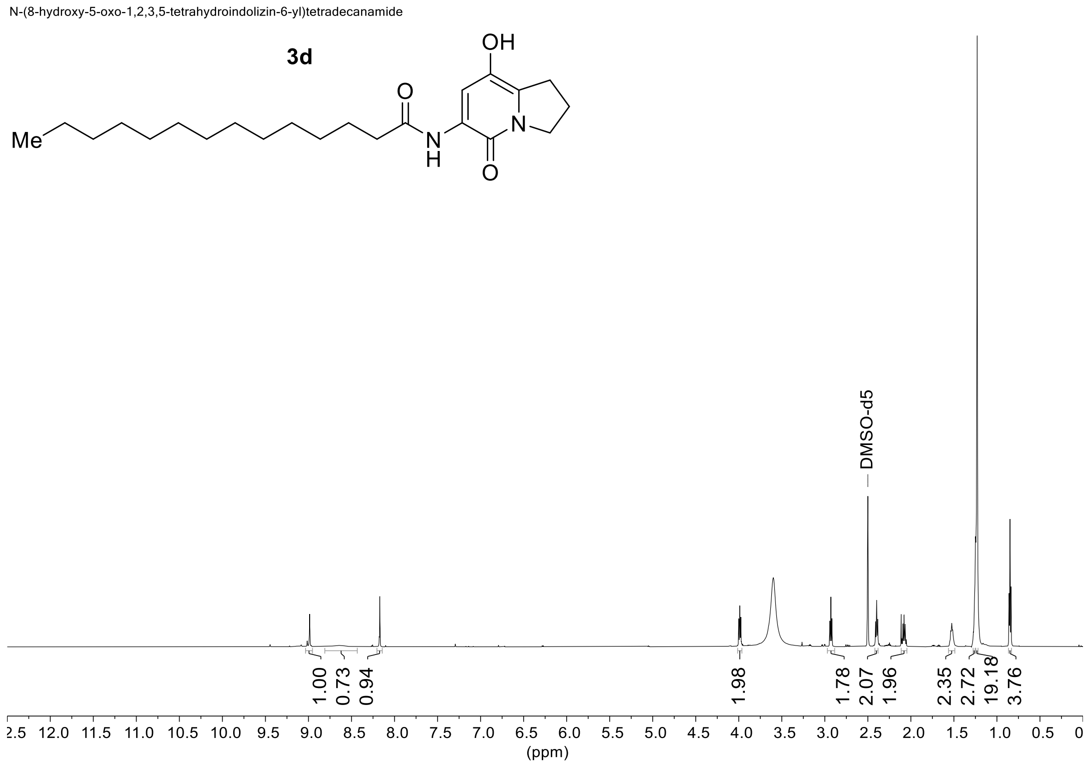

Figure S245. ^{1}H-NMR spectrum (600 MHz) of *N*-(8-hydroxy-5-oxo-1,2,3,5-tetrahydroindolizin-6-yl)tetradecanamide (**3d**).

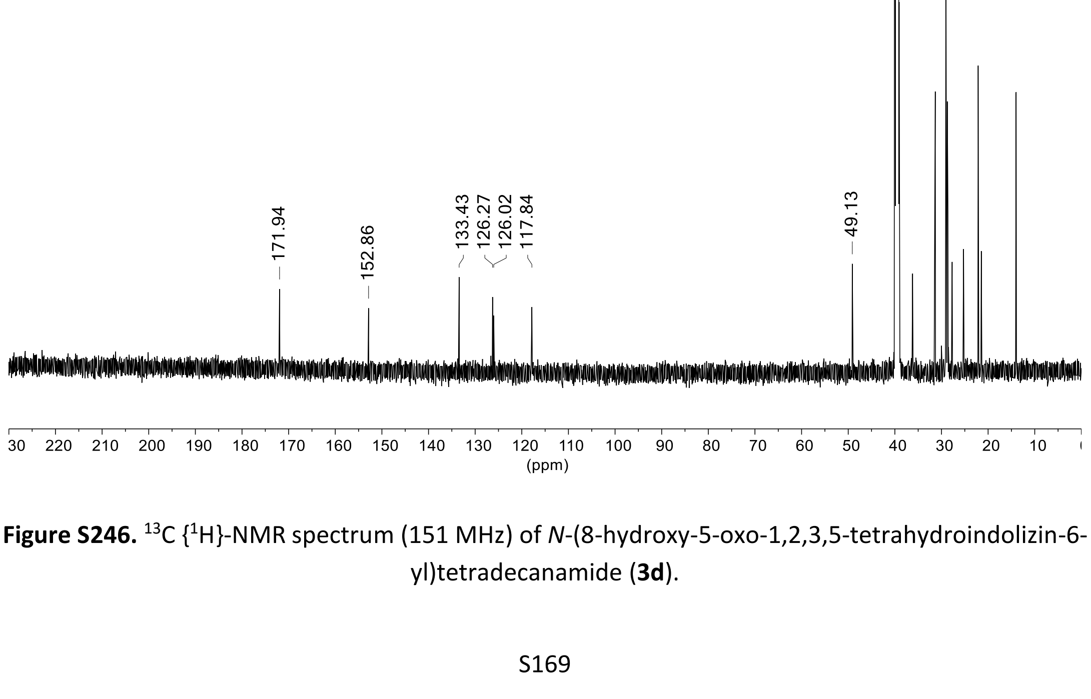

Figure S246. ^{13}C {^{1}H}-NMR spectrum (151 MHz) of *N*-(8-hydroxy-5-oxo-1,2,3,5-tetrahydroindolizin-6-yl)tetradecanamide (**3d**).

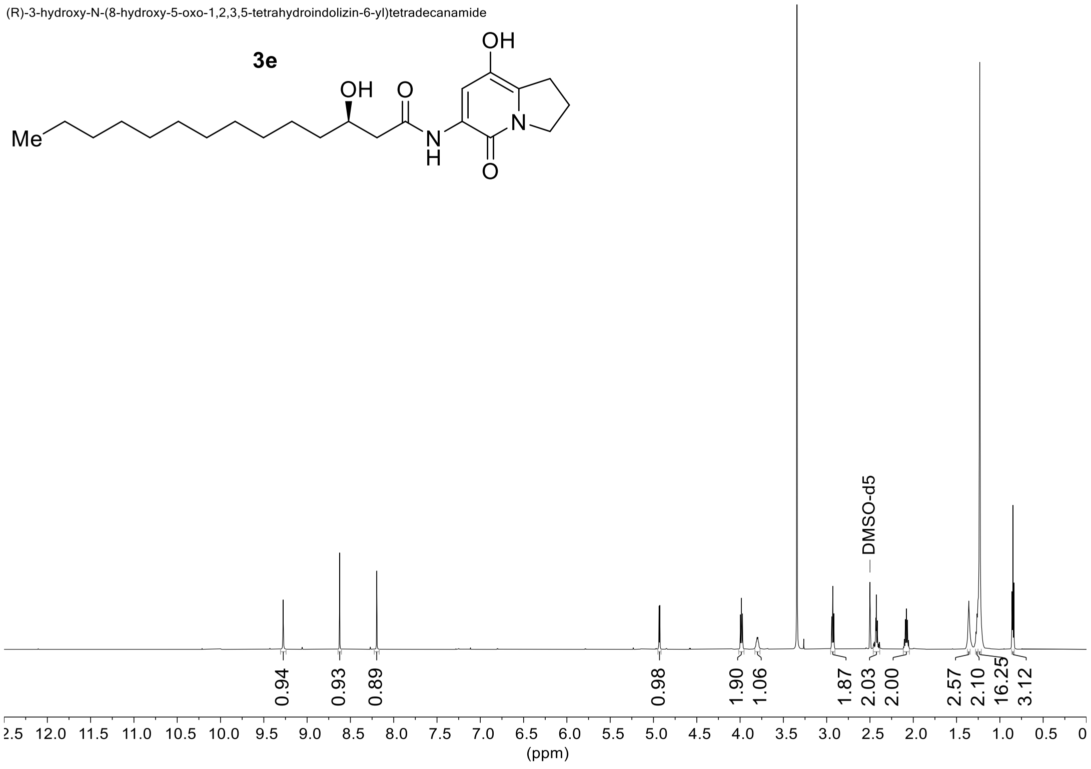

Figure S247. ^{1}H-NMR spectrum (600 MHz) of (*R*)-3-hydroxy-*N*-(8-hydroxy-5-oxo-1,2,3,5-tetrahydroindolizin-6-yl)tetradecanamide (**3e**).

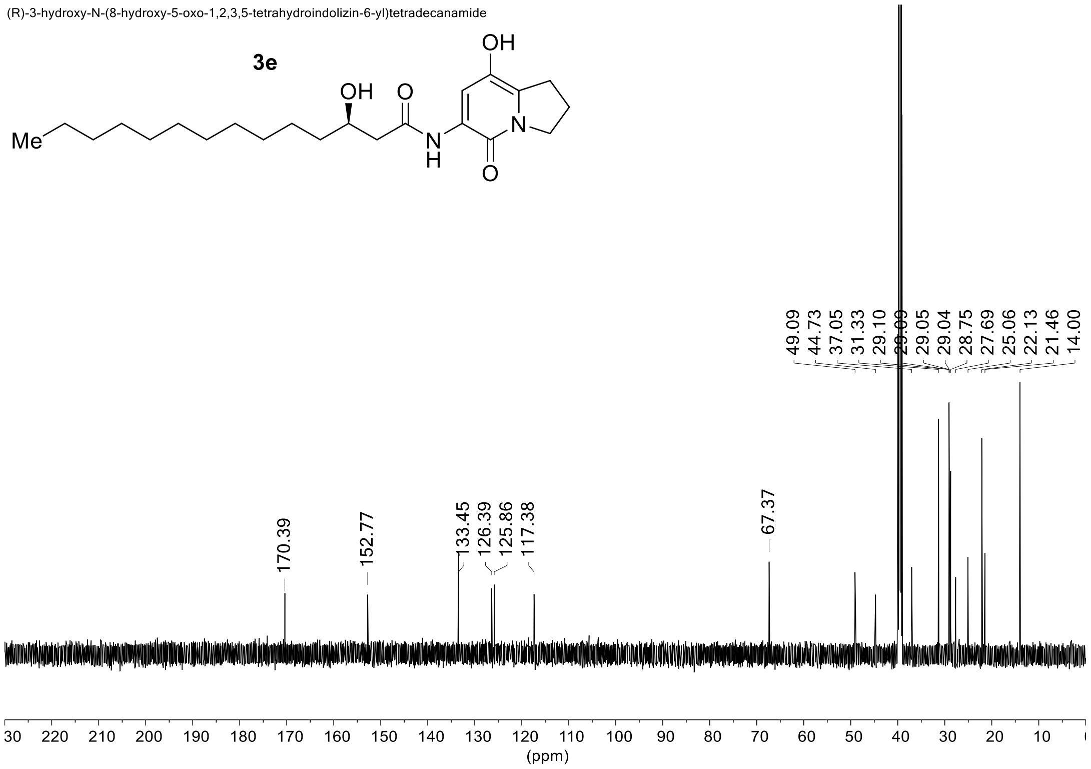

Figure S248. ^{13}C {^{1}H}-NMR spectrum (151 MHz) of (*R*)-3-hydroxy-*N*-(8-hydroxy-5-oxo-1,2,3,5-tetrahydroindolizin-6-yl)tetradecanamide (**3e**).

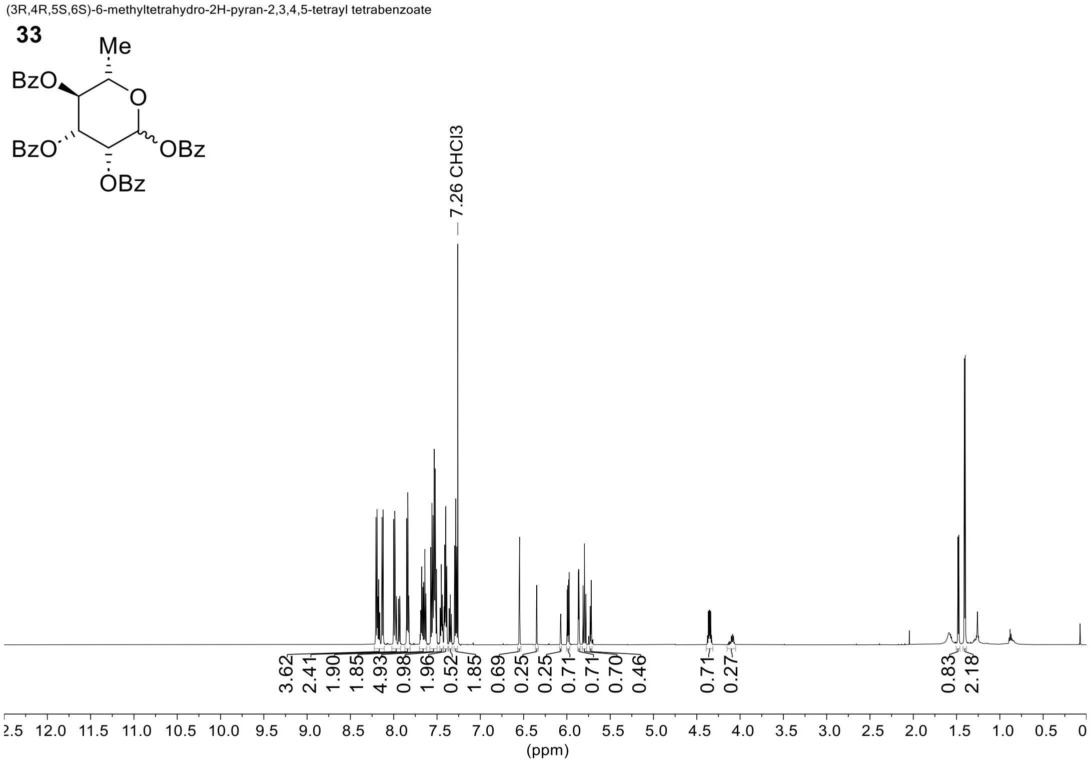

Figure S249. ^{1}H-NMR spectrum (300 MHz) of (3*R*,4*R*,5*S*,6*S*)-6-methyltetrahydro-2*H*-pyran-2,3,4,5-tetrayl tetrabenzoate (**33**).

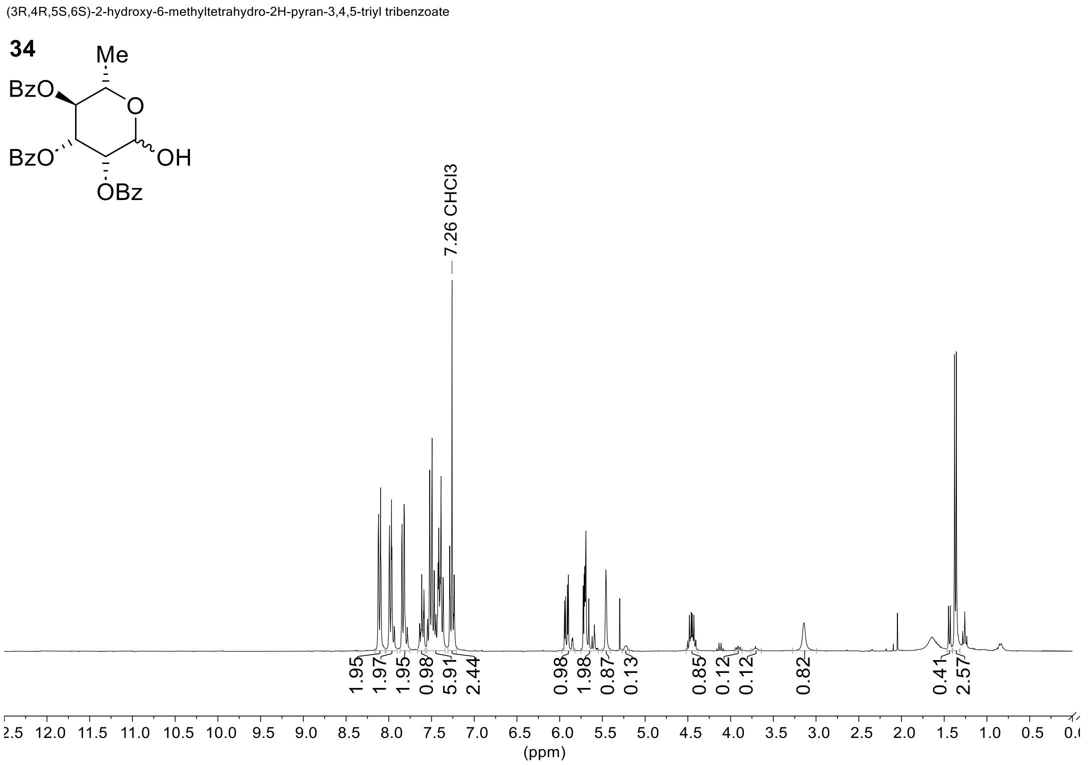

Figure S250. ^{1}H-NMR spectrum (300 MHz) of (3*R*,4*R*,5*S*,6*S*)-2-hydroxy-6-methyltetrahydro-2*H*-pyran-3,4,5-triyl tribenzoate (**34**).

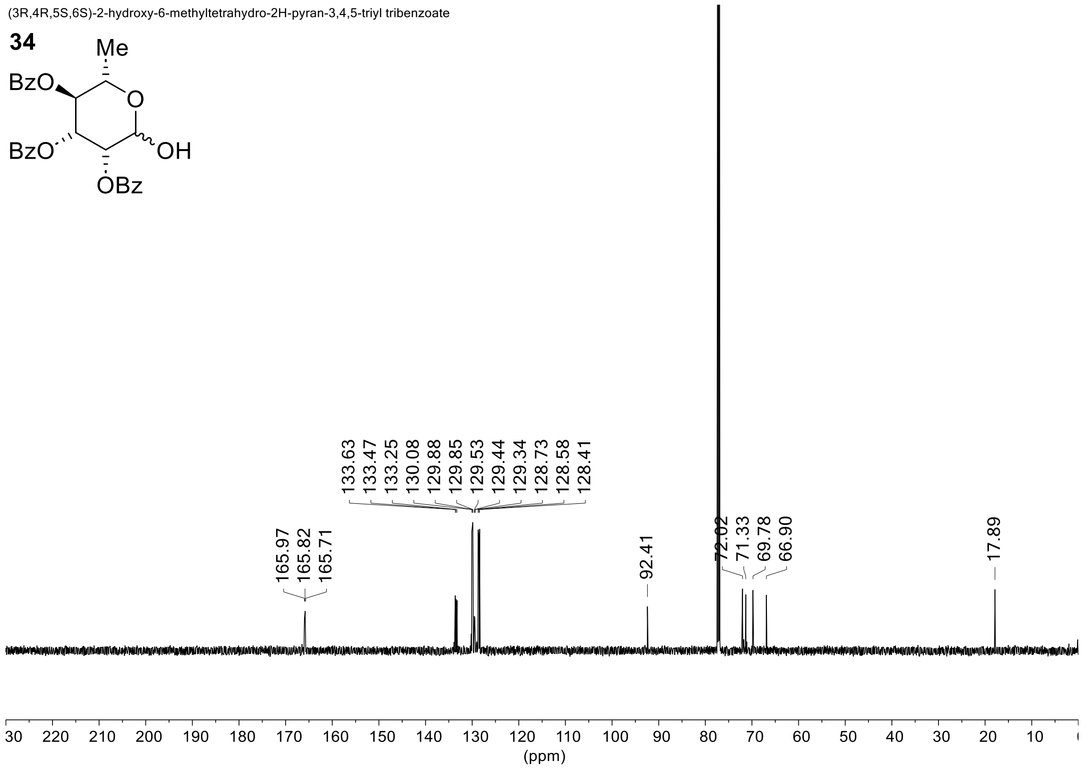

Figure S251. ^{13}C {^{1}H}-NMR spectrum (151 MHz) of (3*R*,4*R*,5*S*,6*S*)-2-hydroxy-6-methyltetrahydro-2*H*-pyran-3,4,5-triyl tribenzoate (**34**).

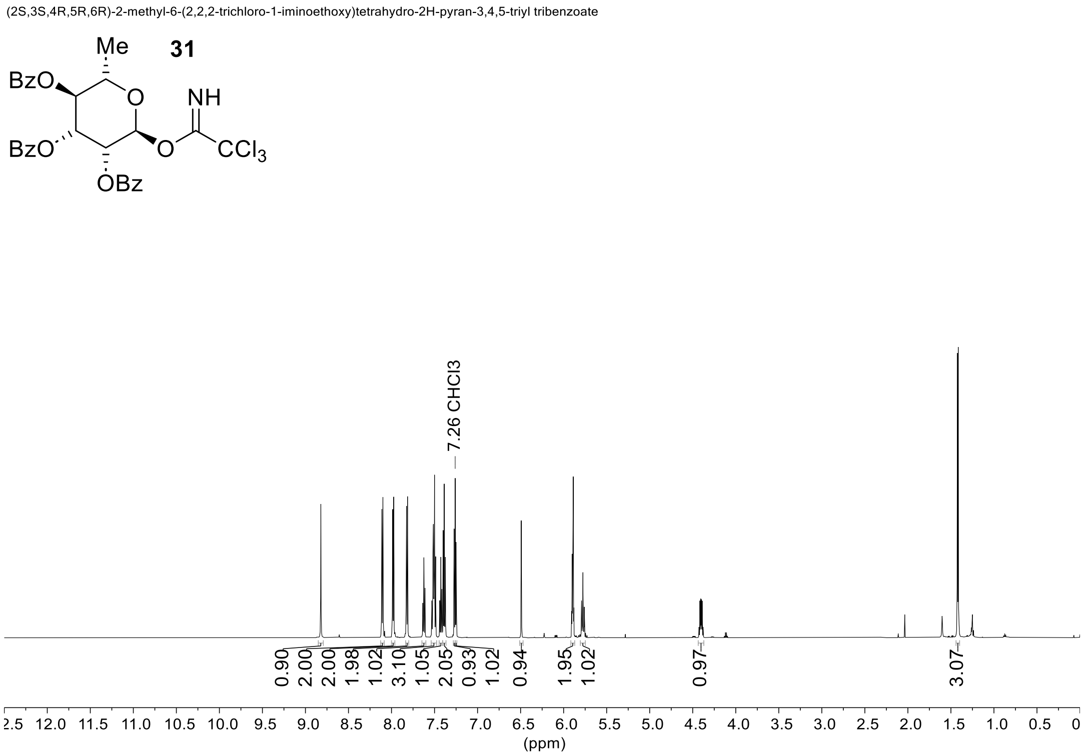

Figure S252. ^{1}H-NMR spectrum (600 MHz) of (2*S*,3*S*,4*R*,5*R*,6*S*)-2-methyl-6-(2,2,2-trichloro-1-iminoethoxy)tetrahydro-2*H*-pyran-3,4,5-triyl tribenzoate (**31**).

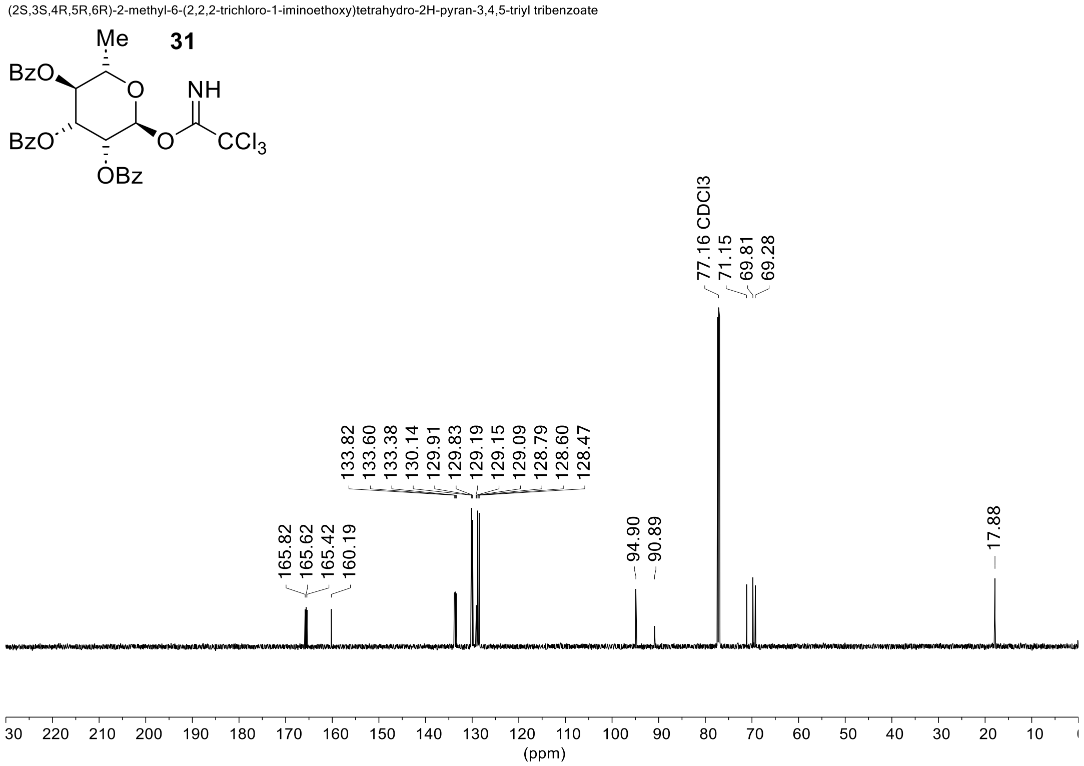

Figure S253. ^{13}C {^{1}H}-NMR spectrum (151 MHz) of (2*S*,3*S*,4*R*,5*R*,6*S*)-2-methyl-6-(2,2,2-trichloro-1-iminoethoxy)tetrahydro-2*H*-pyran-3,4,5-triyl tribenzoate (**31**).

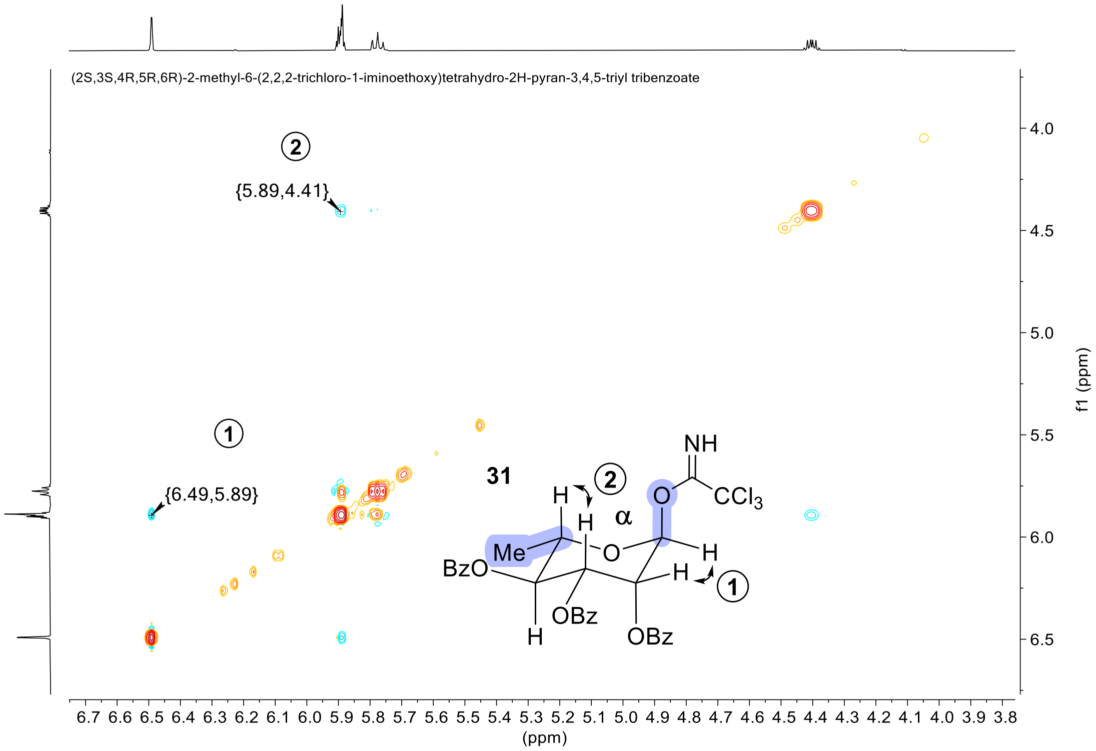

Figure S254. Excerpt from NOESY NMR spectrum of (2*S*,3*S*,4*R*,5*R*,6*S*)-2-methyl-6-(2,2,2-trichloro-1-iminoethoxy)tetrahydro-2*H*-pyran-3,4,5-triyl tribenzoate (**31**) for determination of imidate stereochemistry (α/β).

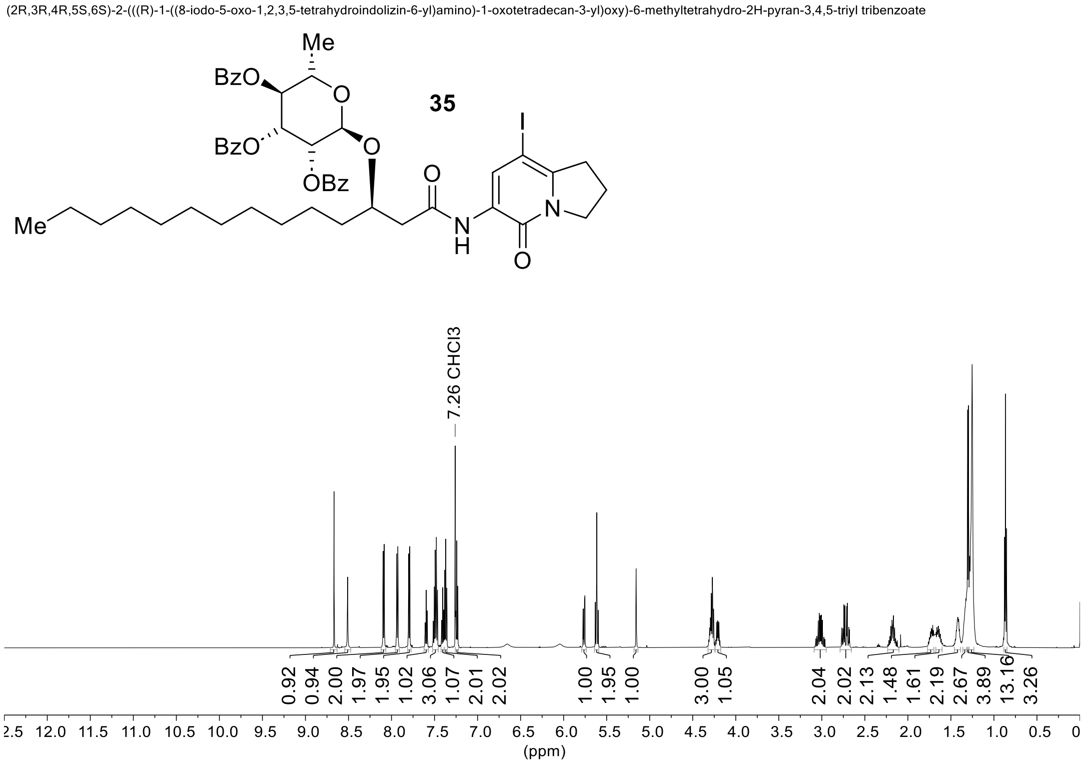

Figure S255. ^{1}H-NMR spectrum (600 MHz) of (2*R*,3*R*,4*R*,5*S*,6*S*)-2-(((*R*)-1-((8-iodo-5-oxo-1,2,3,5-tetrahydroindolizin-6-yl)amino)-1-oxotetradecan-3-yl)oxy)-6-methyltetrahydro-2*H*-pyran-3,4,5-triyl tribenzoate (**35**).

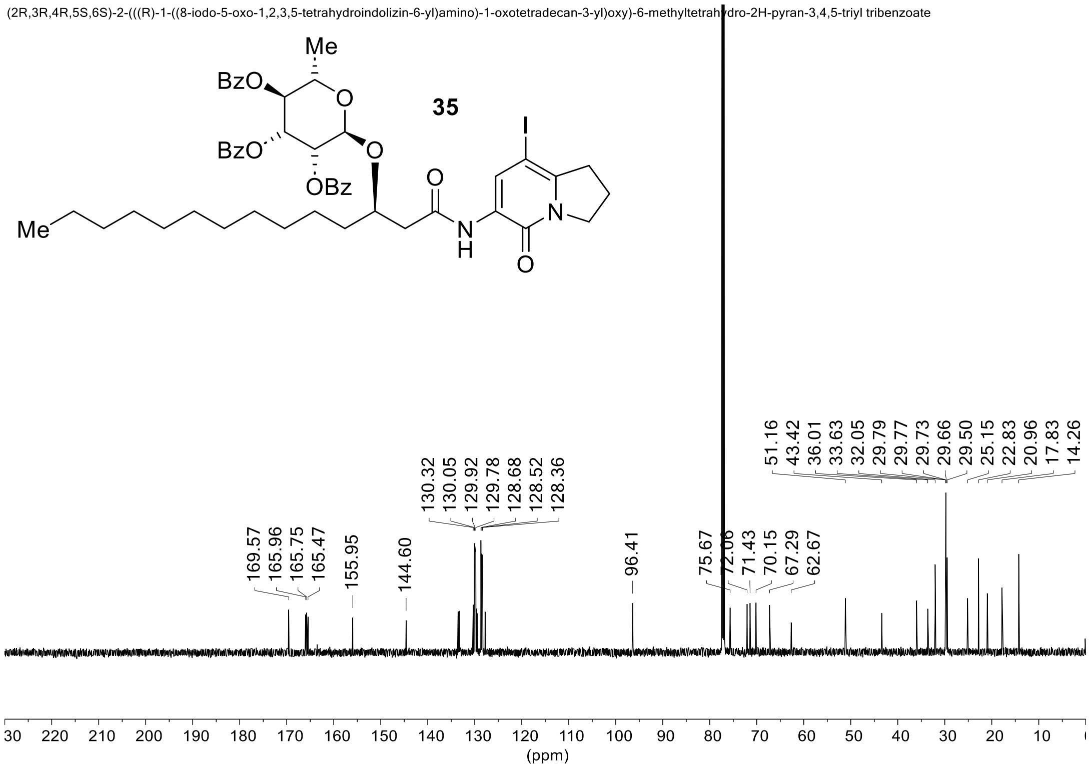

Figure S256. ^{13}C {^{1}H}-NMR spectrum (151 MHz) of (2*R*,3*R*,4*R*,5*S*,6*S*)-2-(((*R*)-1-((8-iodo-5-oxo-1,2,3,5-tetrahydroindolizin-6-yl)amino)-1-oxotetradecan-3-yl)oxy)-6-methyltetrahydro-2*H*-pyran-3,4,5-triyl tribenzoate (**35**).

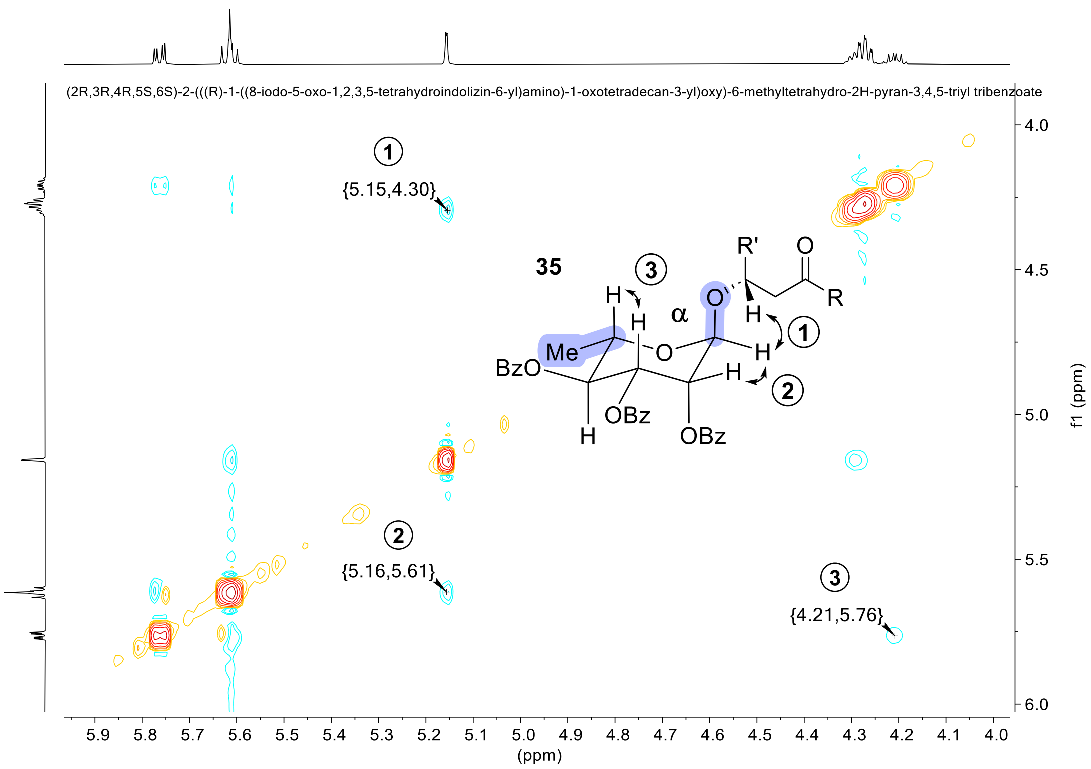

Figure S257. Excerpt from NOESY-NMR spectrum of (2*R*,3*R*,4*R*,5*S*,6*S*)-2-(((*R*)-1-((8-iodo-5-oxo-1,2,3,5-tetrahydroindolizin-6-yl)amino)-1-oxotetradecan-3-yl)oxy)-6-methyltetrahydro-2*H*-pyran-3,4,5-triyl tribenzoate (**35**) for determination of glycoside stereochemistry (α/β).

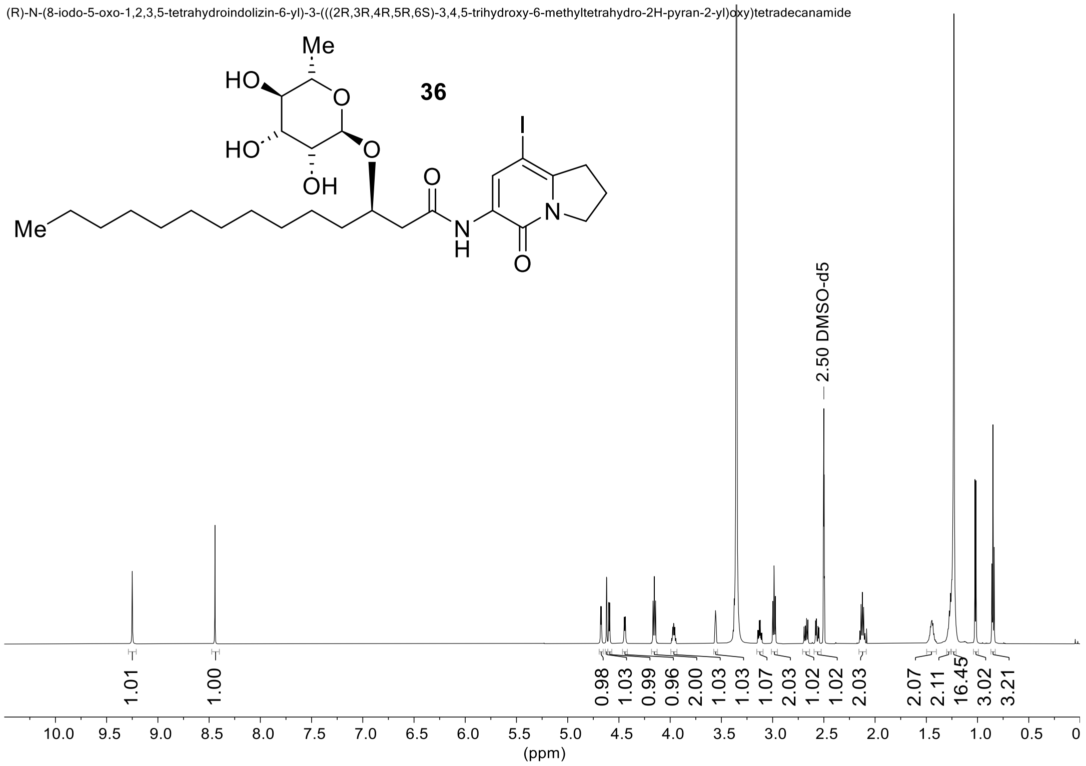

Figure S258. ^{1}H-NMR spectrum (600 MHz) of (*R*)-*N*-(8-iodo-5-oxo-1,2,3,5-tetrahydroindolizin-6-yl)-3-(((2*R*,3*R*,4*R*,5*R*,6*S*)-3,4,5-trihydroxy-6-methyltetrahydro-2*H*-pyran-2-yl)oxy)tetradecanamide (**36**).

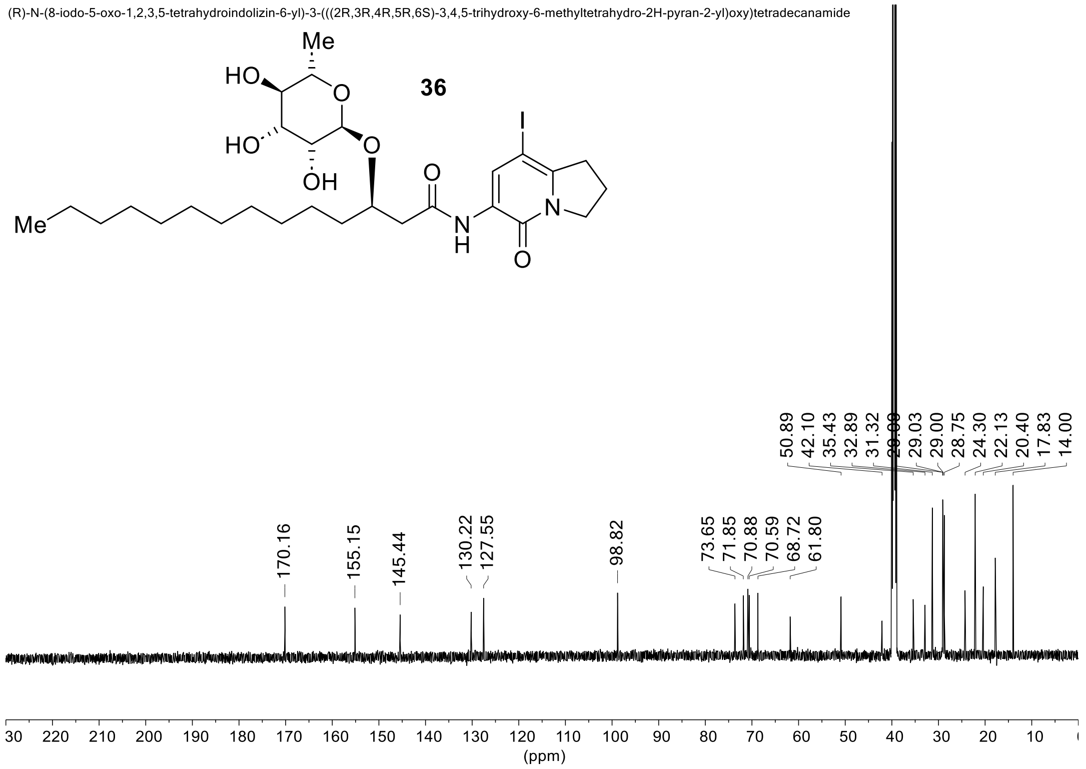

Figure S259. ^{13}C {^{1}H}-NMR spectrum (151 MHz) of (*R*)-*N*-(8-iodo-5-oxo-1,2,3,5-tetrahydroindolizin-6-yl)-3-(((2*R*,3*R*,4*R*,5*R*,6*S*)-3,4,5-trihydroxy-6-methyltetrahydro-2*H*-pyran-2-yl)oxy)tetradecanamide (**36**).

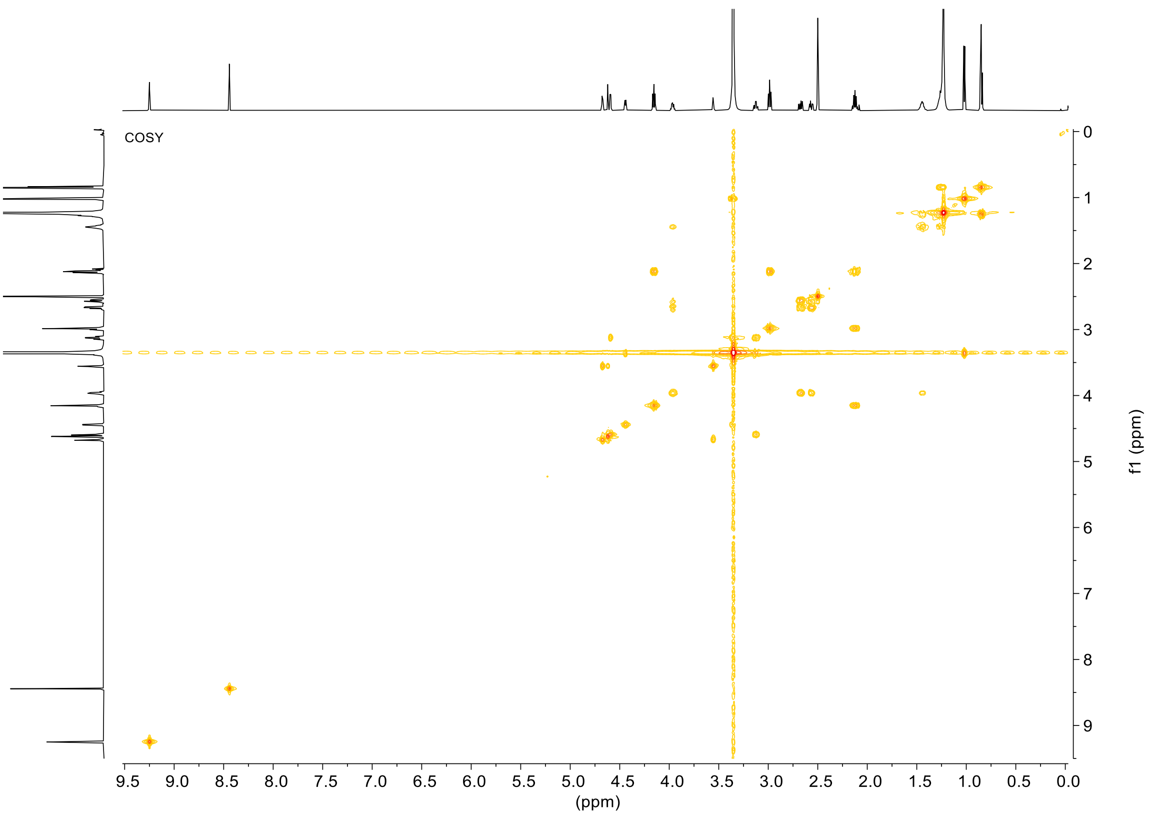

Figure S260. COSY-NMR spectrum of (*R*)-*N*-(8-iodo-5-oxo-1,2,3,5-tetrahydroindolizin-6-yl)-3-(((2*R*,3*R*,4*R*,5*R*,6*S*)-3,4,5-trihydroxy-6-methyltetrahydro-2*H*-pyran-2-yl)oxy)tetradecanamide (**36**).

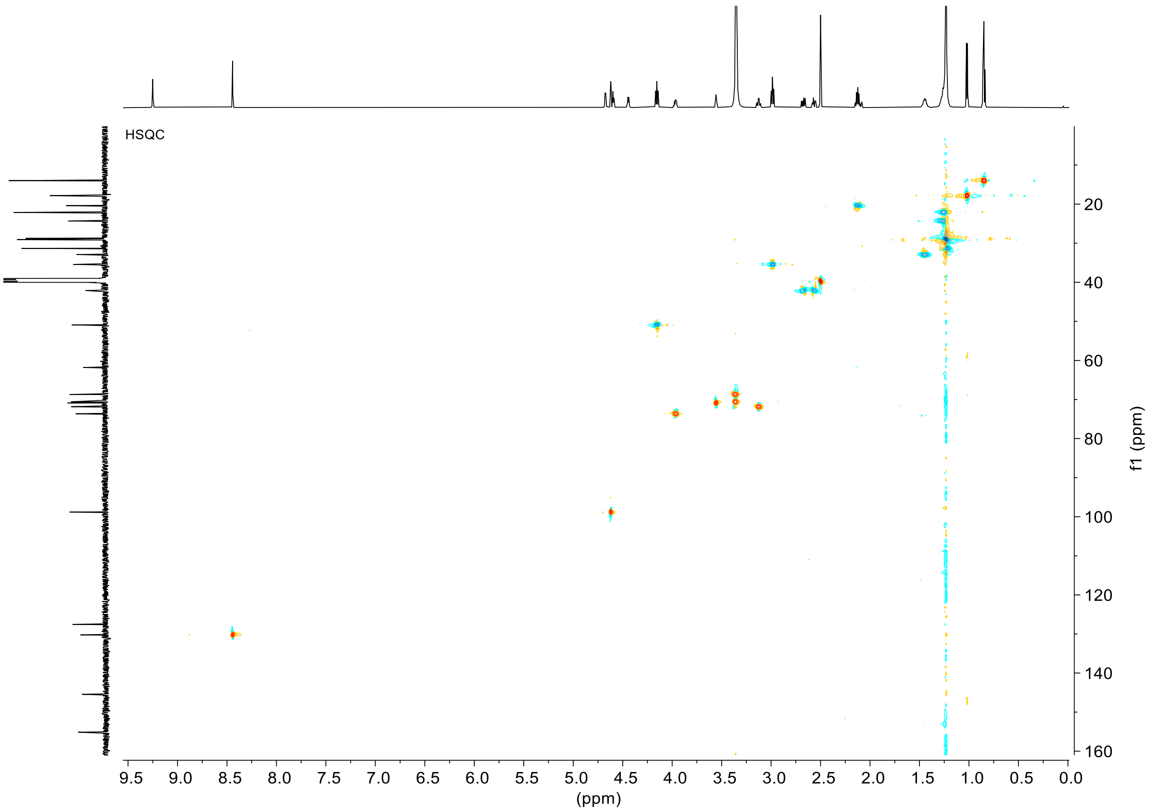

Figure S261. ^{1}H–^{13}C-HSQC-NMR spectrum of (*R*)-*N*-(8-iodo-5-oxo-1,2,3,5-tetrahydroindolizin-6-yl)-3-(((2*R*,3*R*,4*R*,5*R*,6*S*)-3,4,5-trihydroxy-6-methyltetrahydro-2*H*-pyran-2-yl)oxy)tetradecanamide (**36**).

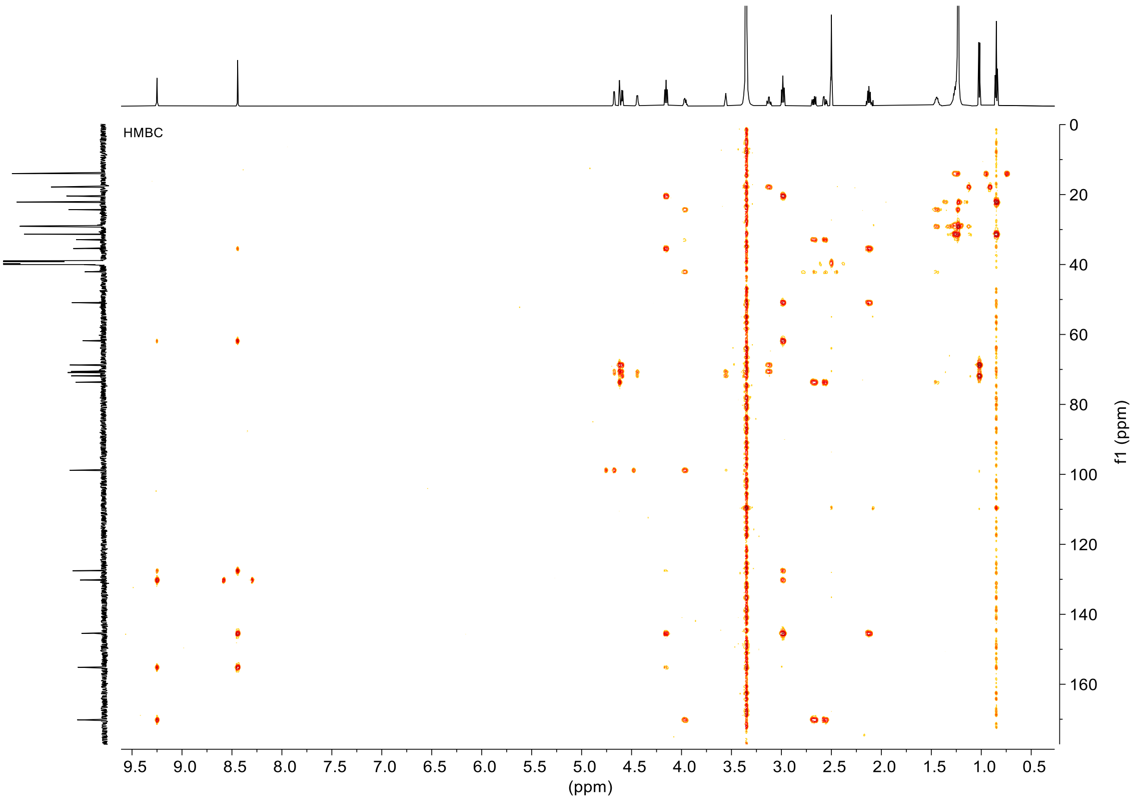

Figure S262. ^1H–^{13}C-HMBC-NMR spectrum of (*R*)-*N*-(8-iodo-5-oxo-1,2,3,5-tetrahydroindolizin-6-yl)-3-(((2*R*,3*R*,4*R*,5*R*,6*S*)-3,4,5-trihydroxy-6-methyltetrahydro-2*H*-pyran-2-yl)oxy)tetradecanamide (**36**).

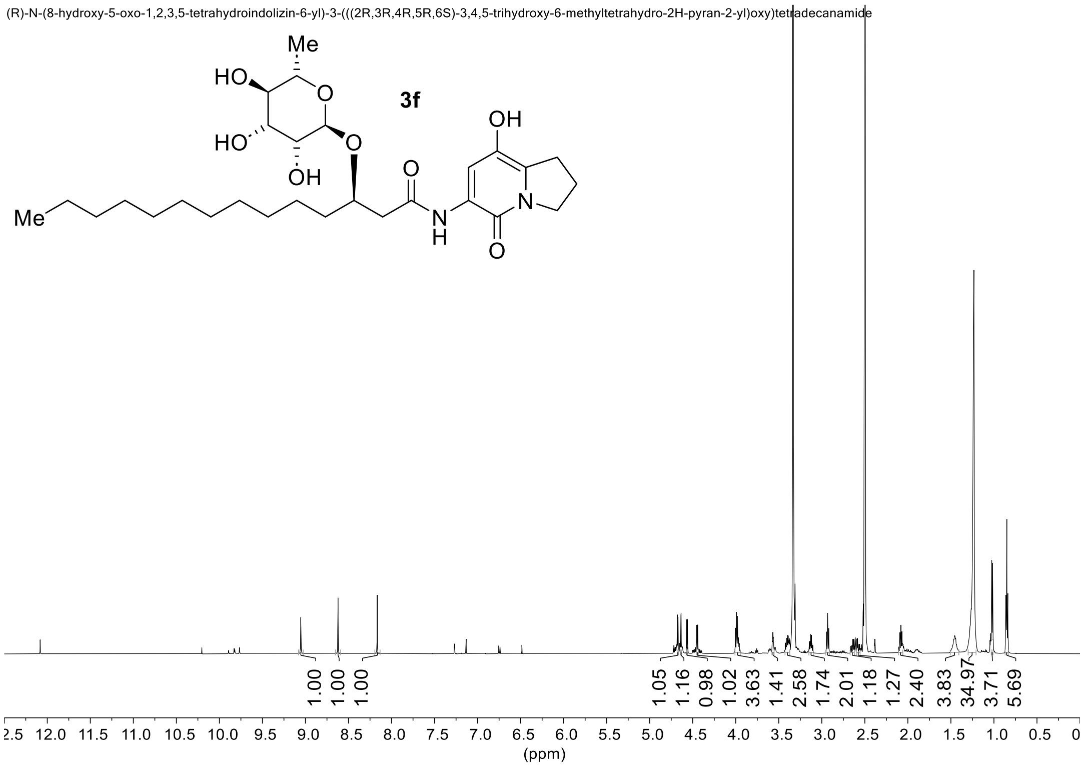

Figure S263. ^1H-NMR spectrum (600 MHz) of (*R*)-*N*-(8-hydroxy-5-oxo-1,2,3,5-tetrahydroindolizin-6-yl)-3-(((2*R*,3*R*,4*R*,5*R*,6*S*)-3,4,5-trihydroxy-6-methyltetrahydro-2*H*-pyran-2-yl)oxy)tetradecanamide (**3f**). Due to overlapping signals of decomposition products, integrals of these peaks are higher than expected.

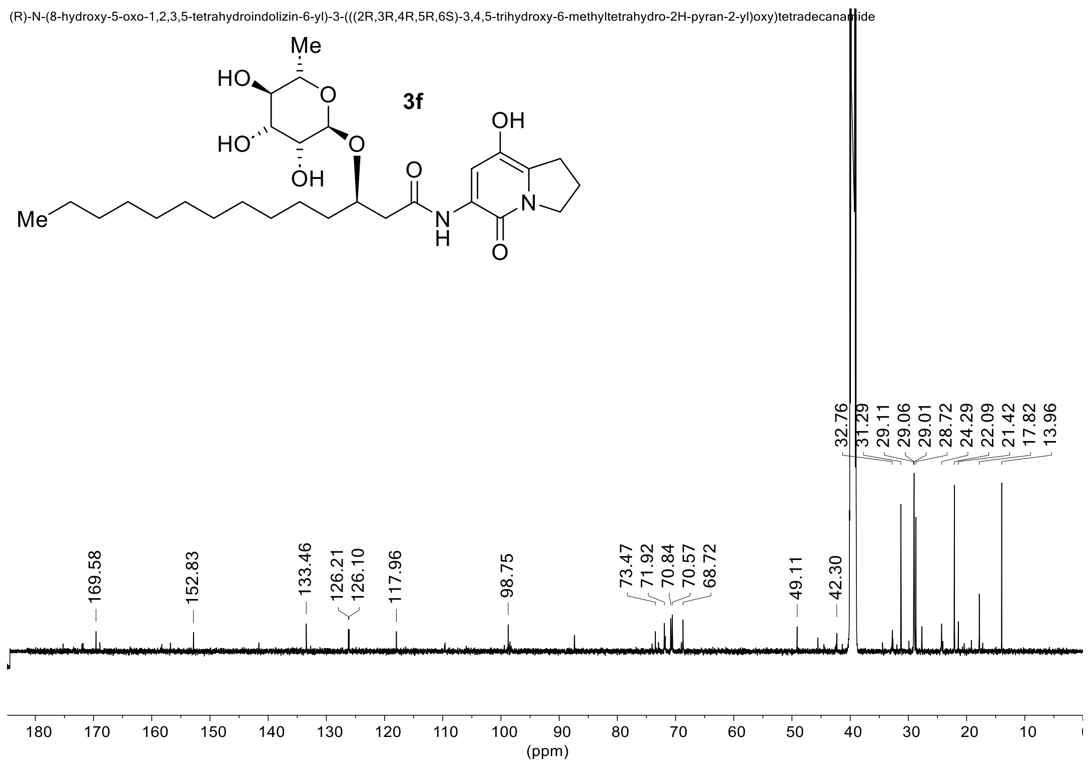

Figure S264. ^{13}C {^{1}H}-NMR spectrum (126 MHz) of (*R*)-*N*-(8-hydroxy-5-oxo-1,2,3,5-tetrahydroindolizin-6-yl)-3-(((2*R*,3*R*,4*R*,5*R*,6*S*)-3,4,5-trihydroxy-6-methyltetrahydro-2*H*-pyran-2-yl)oxy)tetradecanamide (**3f**). Additional signals arise from decomposition products.

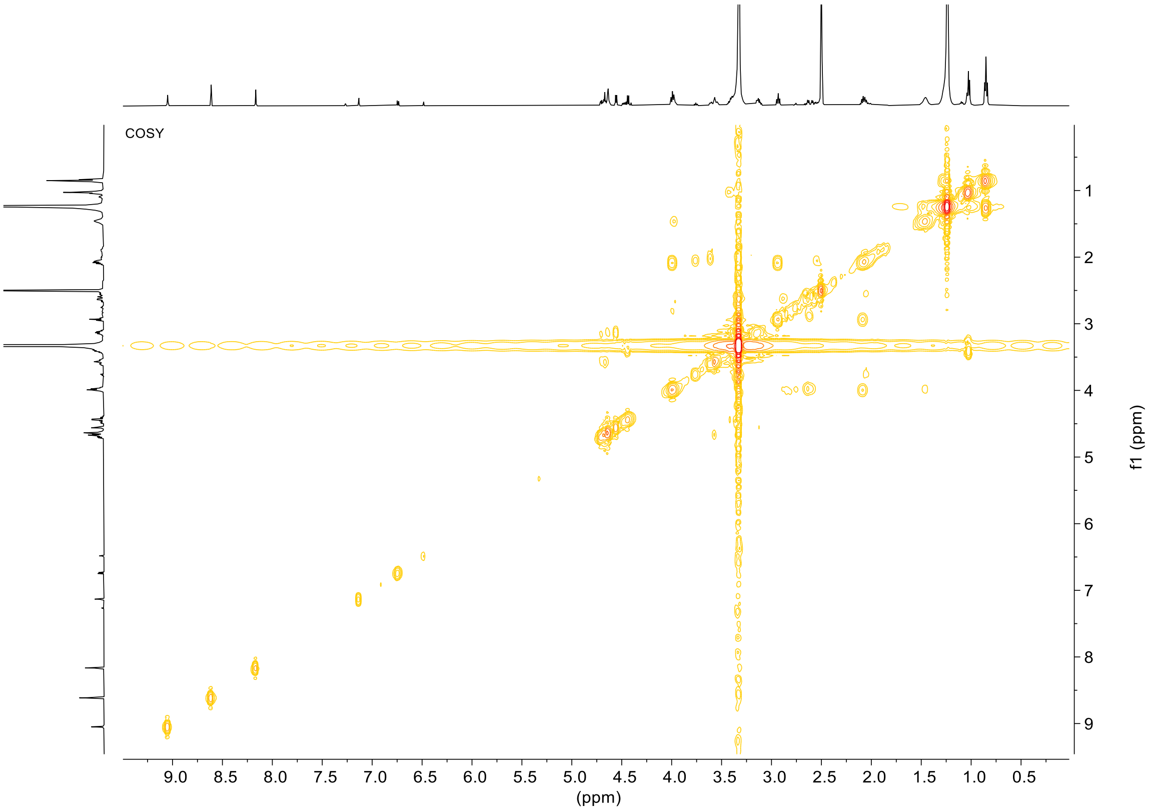

Figure S265. COSY-NMR spectrum of (*R*)-*N*-(8-hydroxy-5-oxo-1,2,3,5-tetrahydroindolizin-6-yl)-3-(((2*R*,3*R*,4*R*,5*R*,6*S*)-3,4,5-trihydroxy-6-methyltetrahydro-2*H*-pyran-2-yl)oxy)tetradecanamide (**3f**).

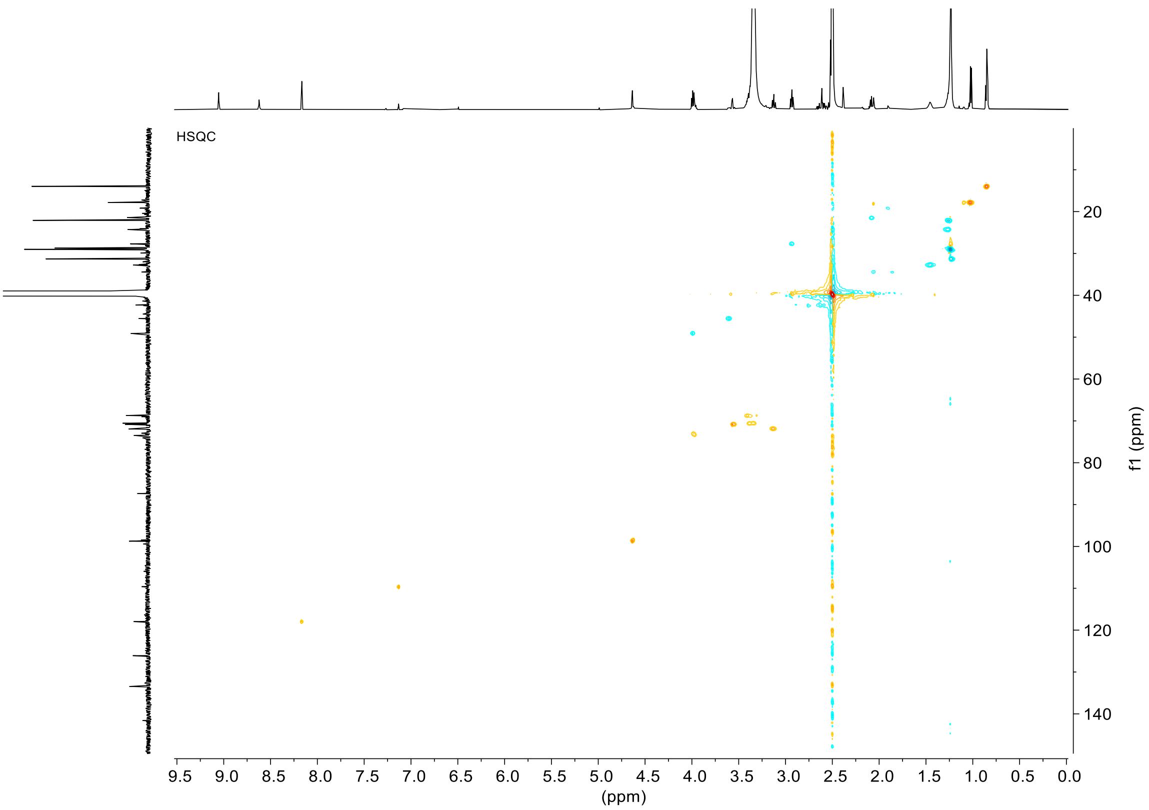

Figure S266. ^{1}H–^{13}C-HSQC-NMR spectrum of (*R*)-*N*-(8-hydroxy-5-oxo-1,2,3,5-tetrahydroindolizin-6-yl)-3-(((2*R*,3*R*,4*R*,5*R*,6*S*)-3,4,5-trihydroxy-6-methyltetrahydro-2*H*-pyran-2-yl)oxy)tetradecanamide (**3f**).

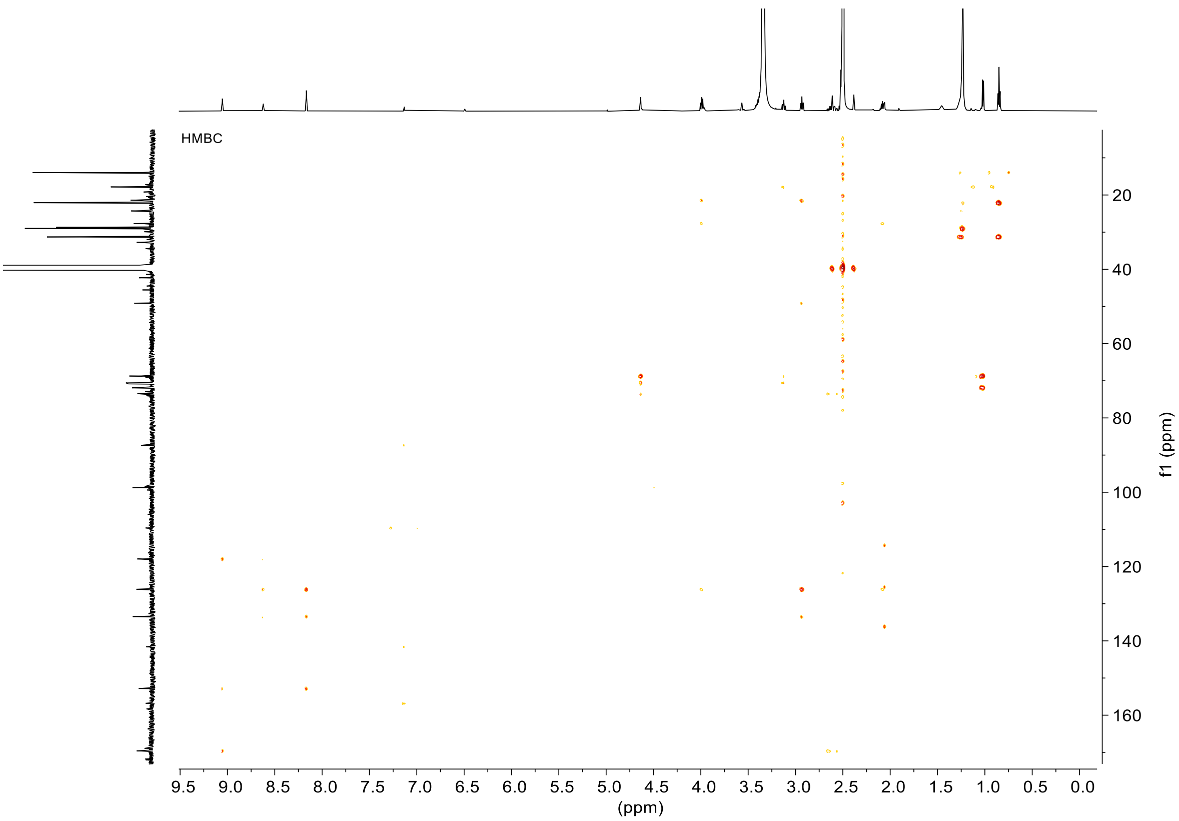

Figure S267. ^{1}H–^{13}C-HMBC-NMR spectrum of (*R*)-*N*-(8-hydroxy-5-oxo-1,2,3,5-tetrahydroindolizin-6-yl)-3-(((2*R*,3*R*,4*R*,5*R*,6*S*)-3,4,5-trihydroxy-6-methyltetrahydro-2*H*-pyran-2-yl)oxy)tetradecanamide (**3f**).

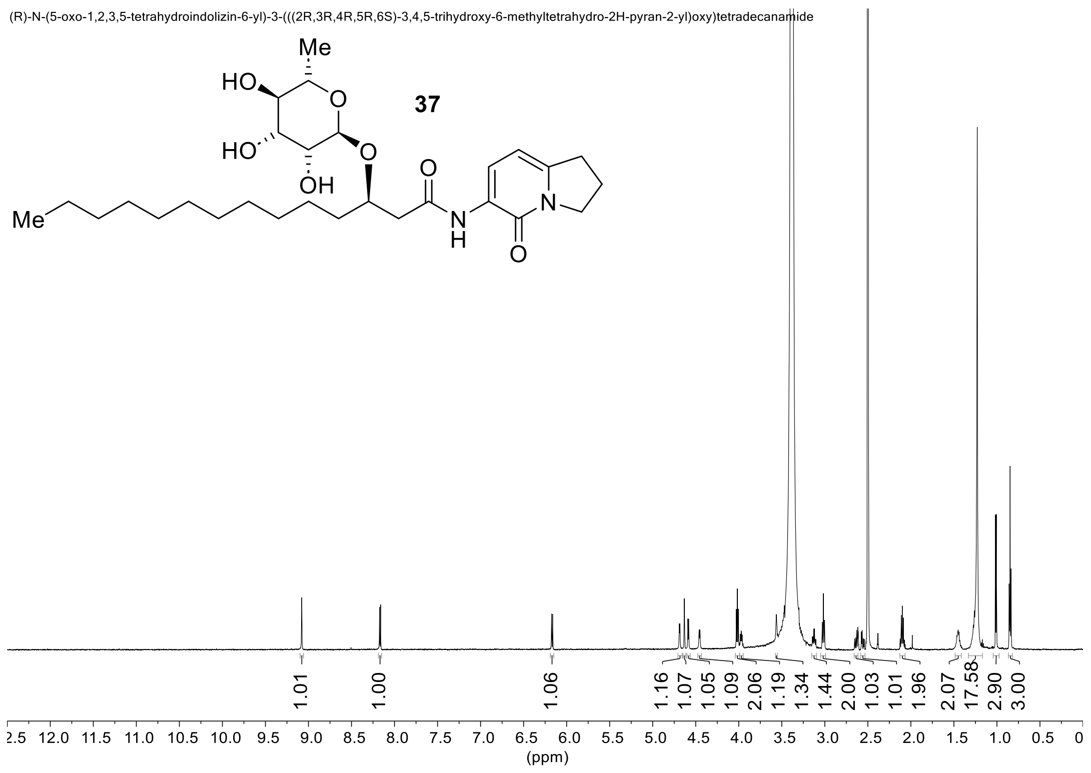

Figure S268. ¹H-NMR spectrum (600 MHz) of (*R*)-*N*-(5-oxo-1,2,3,5-tetrahydroindolizin-6-yl)-3-(((2*R*,3*R*,4*R*,5*R*,6*S*)-3,4,5-trihydroxy-6-methyltetrahydro-2*H*-pyran-2-yl)oxy)tetradecanamide (**37**).

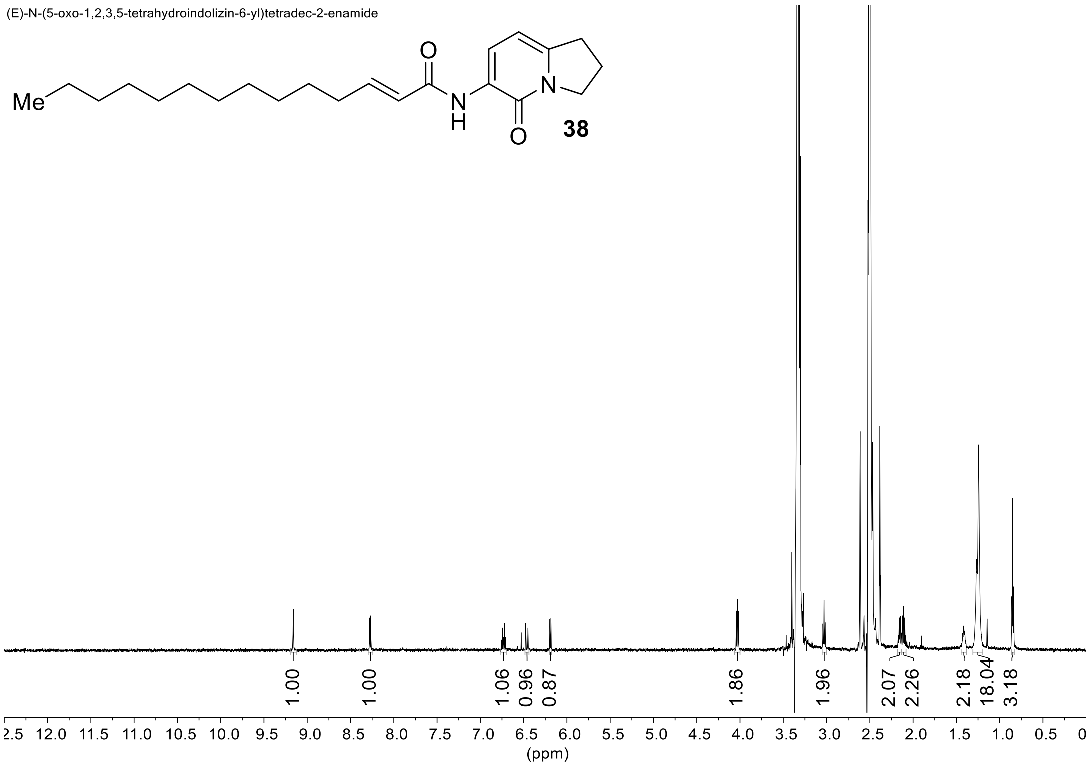

Figure S269. ¹H-NMR spectrum (600 MHz) of (*E*)-*N*-(5-oxo-1,2,3,5-tetrahydroindolizin-6-yl)tetradec-2-enamide (**38**).

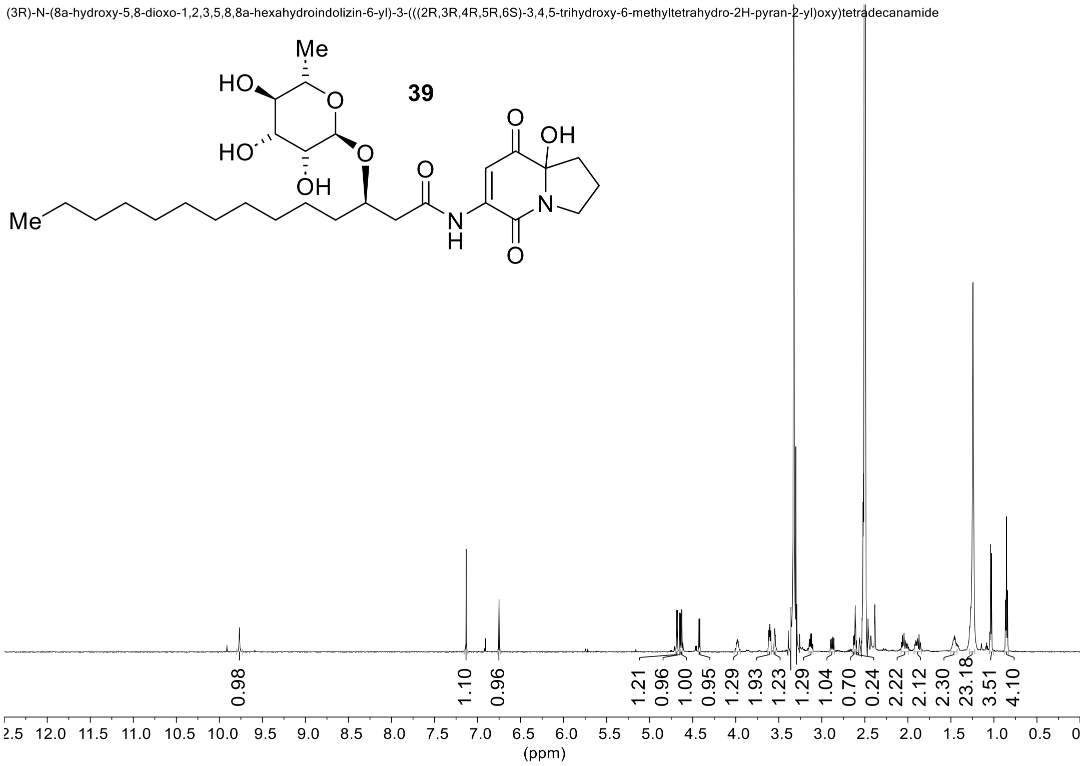

Figure S270. ^{1}H-NMR spectrum (600 MHz) of (3*R*)-*N*-(8a-hydroxy-5,8-dioxo-1,2,3,5,8,8a-hexahydroindolizin-6-yl)-3-(((2*R*,3*R*,4*R*,5*R*,6*S*)-3,4,5-trihydroxy-6-methyltetrahydro-2*H*-pyran-2-yl)oxy)tetradecanamide (**39**), isomer 1.

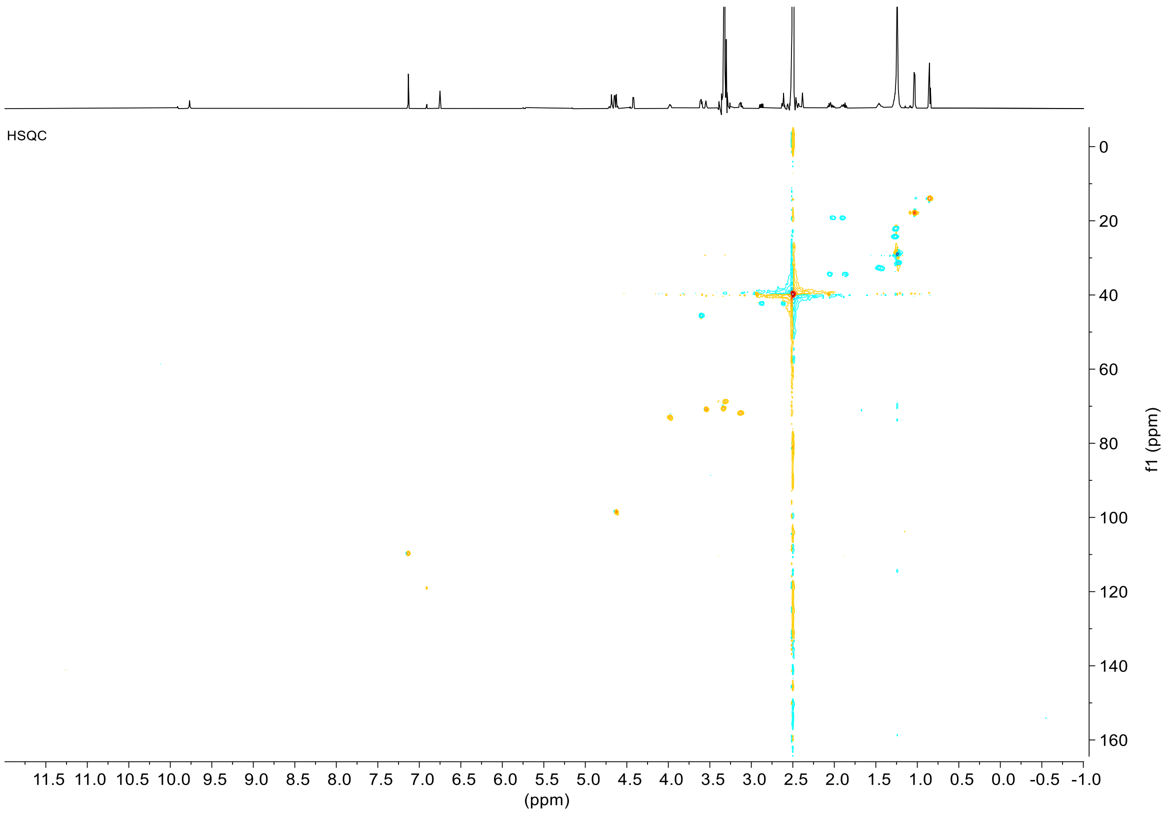

Figure S271. ^{1}H–^{13}C-HSQC-NMR spectrum of (3*R*)-*N*-(8a-hydroxy-5,8-dioxo-1,2,3,5,8,8a-hexahydroindolizin-6-yl)-3-(((2*R*,3*R*,4*R*,5*R*,6*S*)-3,4,5-trihydroxy-6-methyltetrahydro-2*H*-pyran-2-yl)oxy)tetradecanamide (**39**), isomer 1.

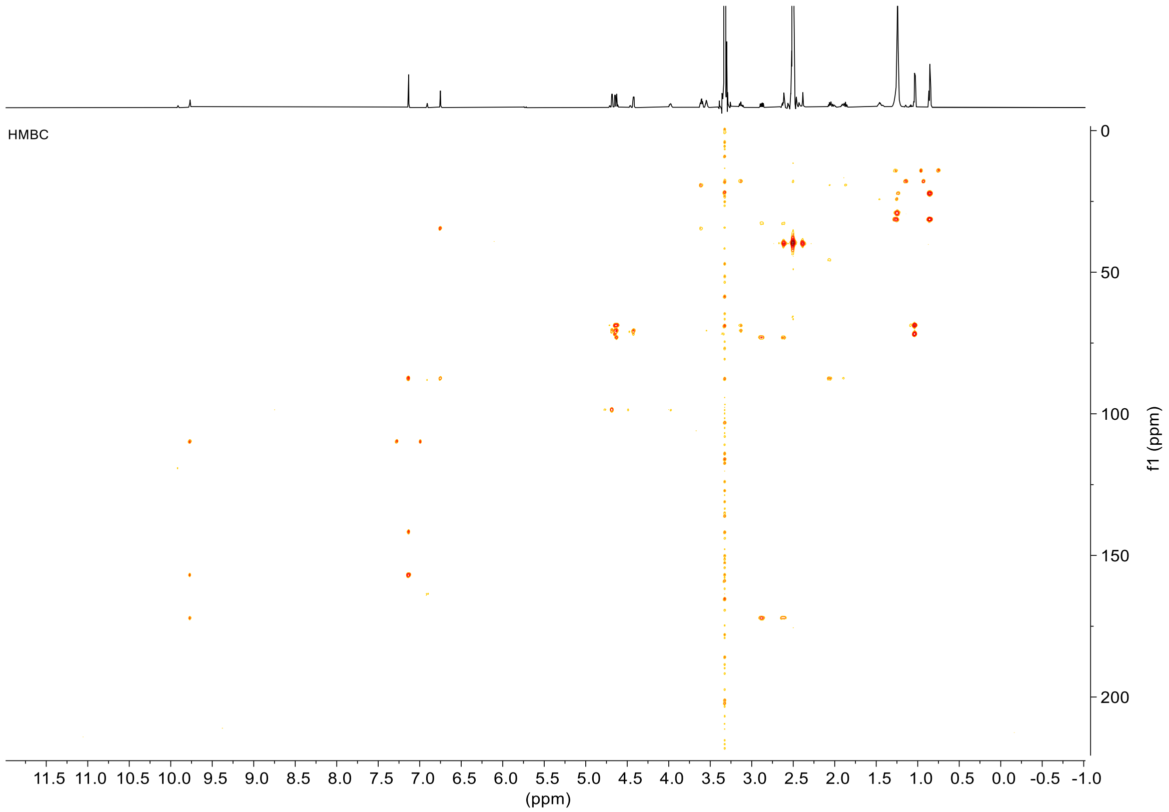

Figure S272. ^{1}H–^{13}C-HMBC-NMR spectrum of (3*R*)-*N*-(8a-hydroxy-5,8-dioxo-1,2,3,5,8,8a-hexahydroindolizin-6-yl)-3-(((2*R*,3*R*,4*R*,5*R*,6*S*)-3,4,5-trihydroxy-6-methyltetrahydro-2*H*-pyran-2-yl)oxy)tetradecanamide (**39**), isomer 1.

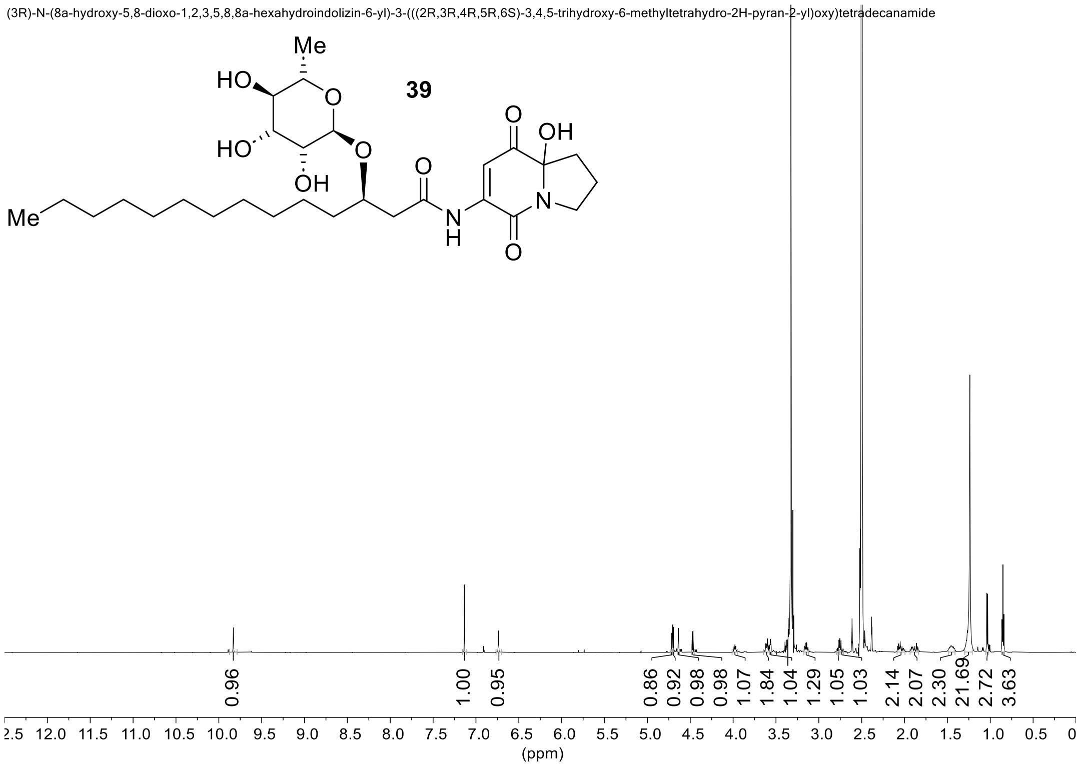

Figure S273. ^{1}H-NMR spectrum (600 MHz) of (3*R*)-*N*-(8a-hydroxy-5,8-dioxo-1,2,3,5,8,8a-hexahydroindolizin-6-yl)-3-(((2*R*,3*R*,4*R*,5*R*,6*S*)-3,4,5-trihydroxy-6-methyltetrahydro-2*H*-pyran-2-yl)oxy)tetradecanamide (**39**), isomer 2.

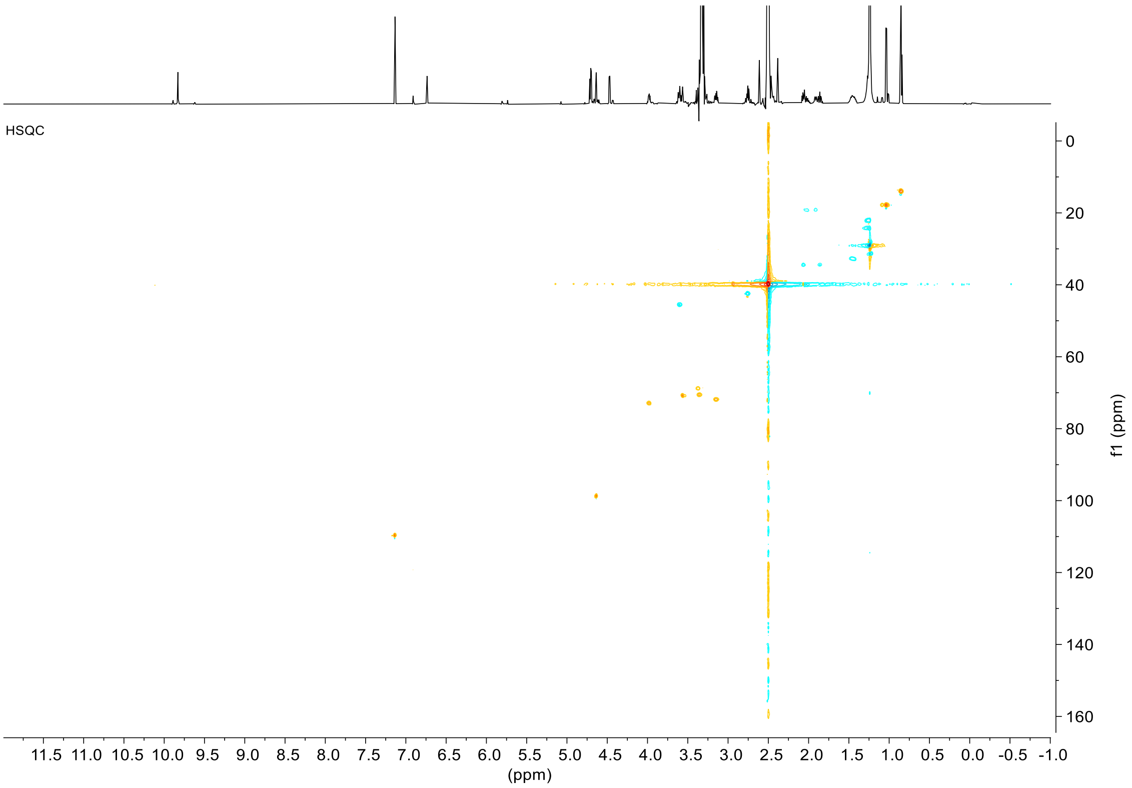

Figure S274. ^{1}H–^{13}C-HSQC-NMR spectrum of (3*R*)-*N*-(8a-hydroxy-5,8-dioxo-1,2,3,5,8,8a-hexahydroindolizin-6-yl)-3-(((2*R*,3*R*,4*R*,5*R*,6*S*)-3,4,5-trihydroxy-6-methyltetrahydro-2*H*-pyran-2-yl)oxy)tetradecanamide (**39**), isomer 2.

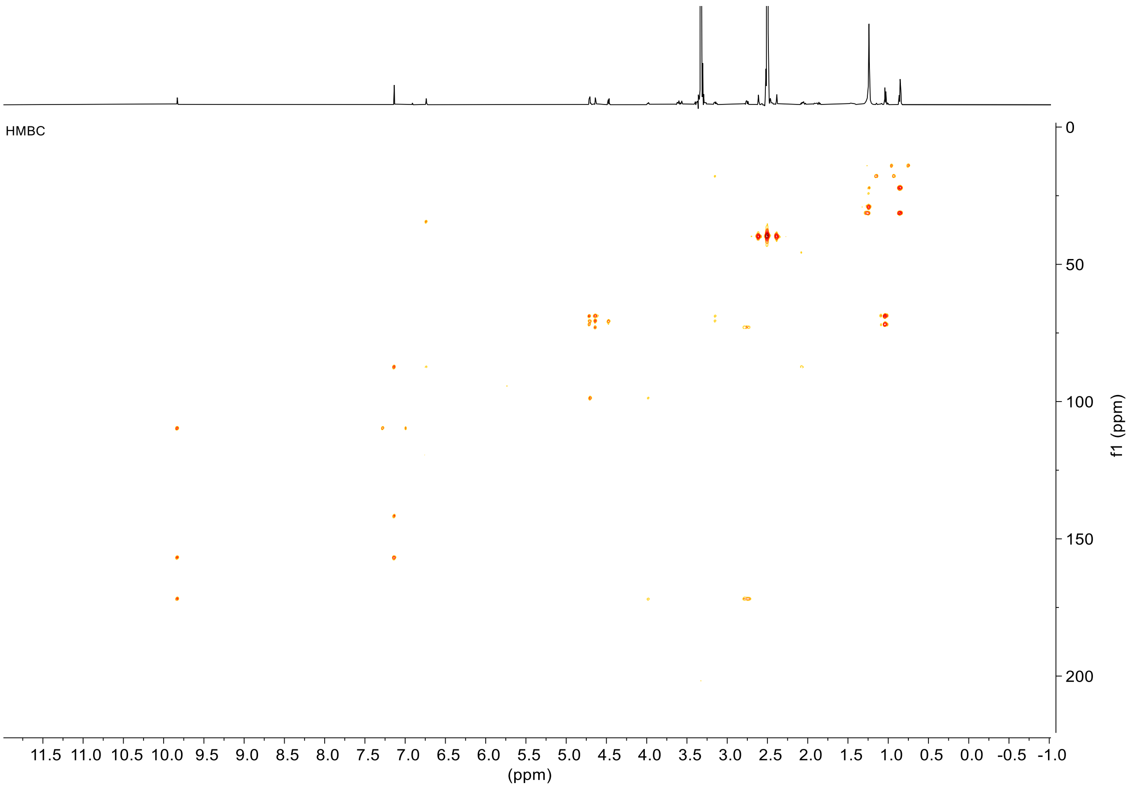

Figure S275. ^{1}H–^{13}C-HMBC-NMR spectrum of (3*R*)-*N*-(8a-hydroxy-5,8-dioxo-1,2,3,5,8,8a-hexahydroindolizin-6-yl)-3-(((2*R*,3*R*,4*R*,5*R*,6*S*)-3,4,5-trihydroxy-6-methyltetrahydro-2*H*-pyran-2-yl)oxy)tetradecanamide (**39**), isomer 2.

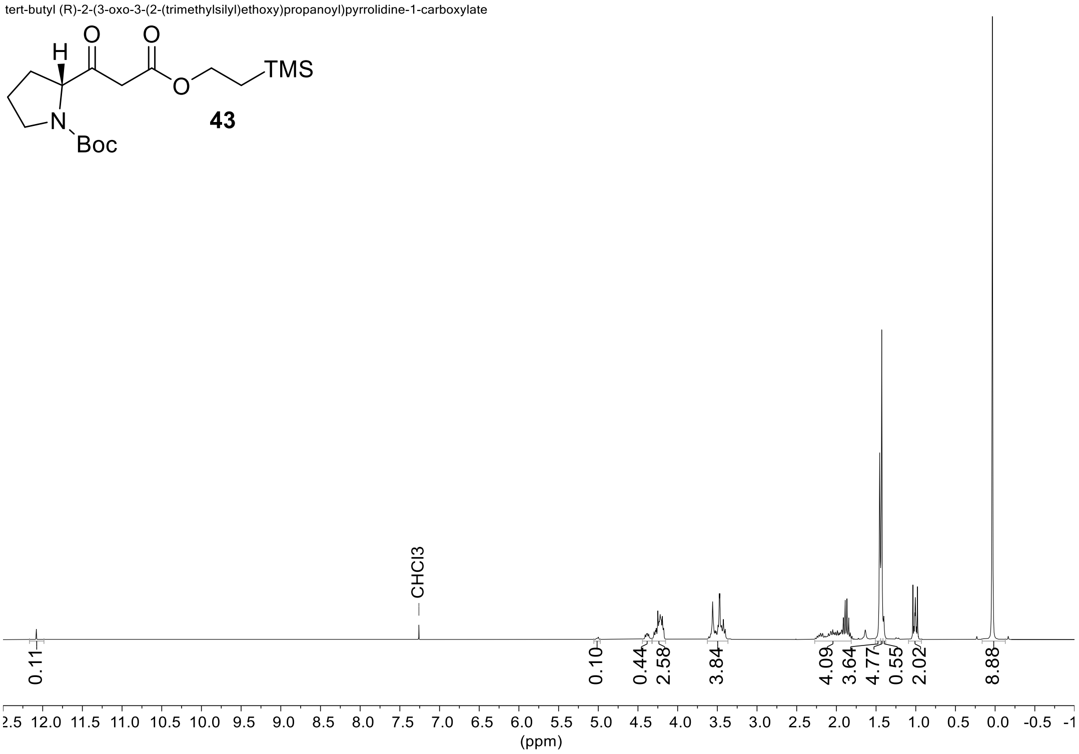

Figure S276. ^{1}H-NMR spectrum (300 MHz) of *tert*-butyl (*R*)-2-(3-oxo-3-(2-(trimethylsilyl)ethoxy)propanoyl)pyrrolidine-1-carboxylate (**43**).

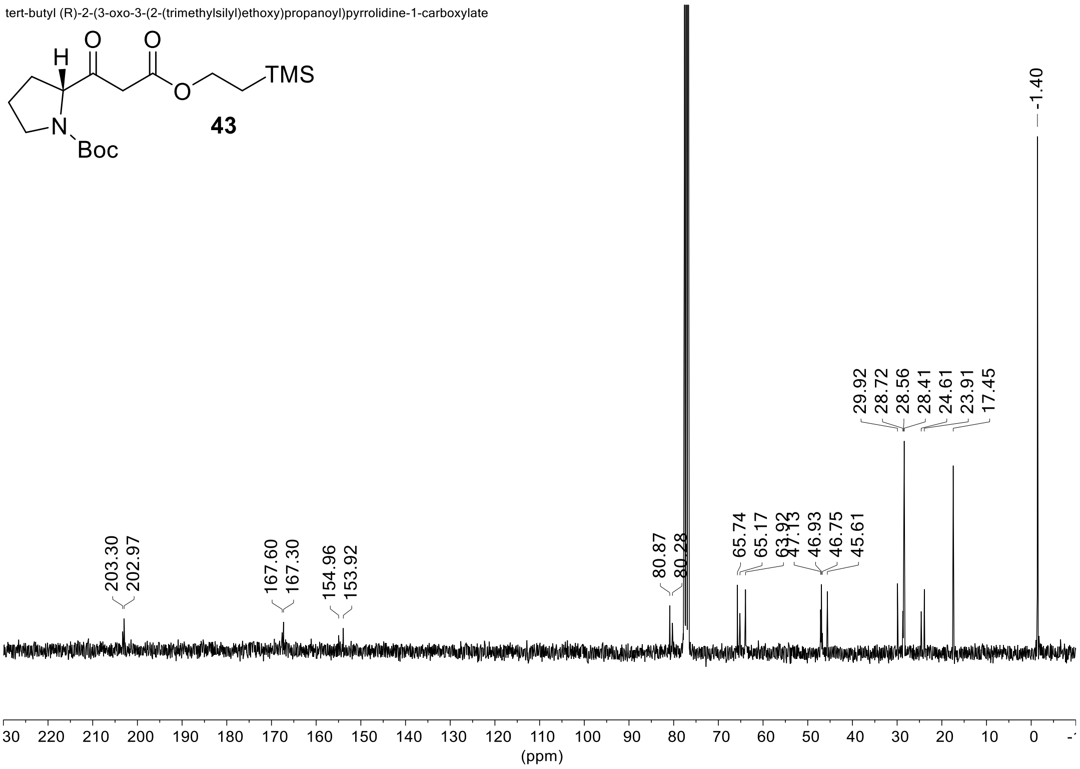

Figure S277. ^{13}C {^{1}H}-NMR spectrum (75.5 MHz) of *tert*-butyl (*R*)-2-(3-oxo-3-(2-(trimethylsilyl)ethoxy)propanoyl)pyrrolidine-1-carboxylate (**43**).

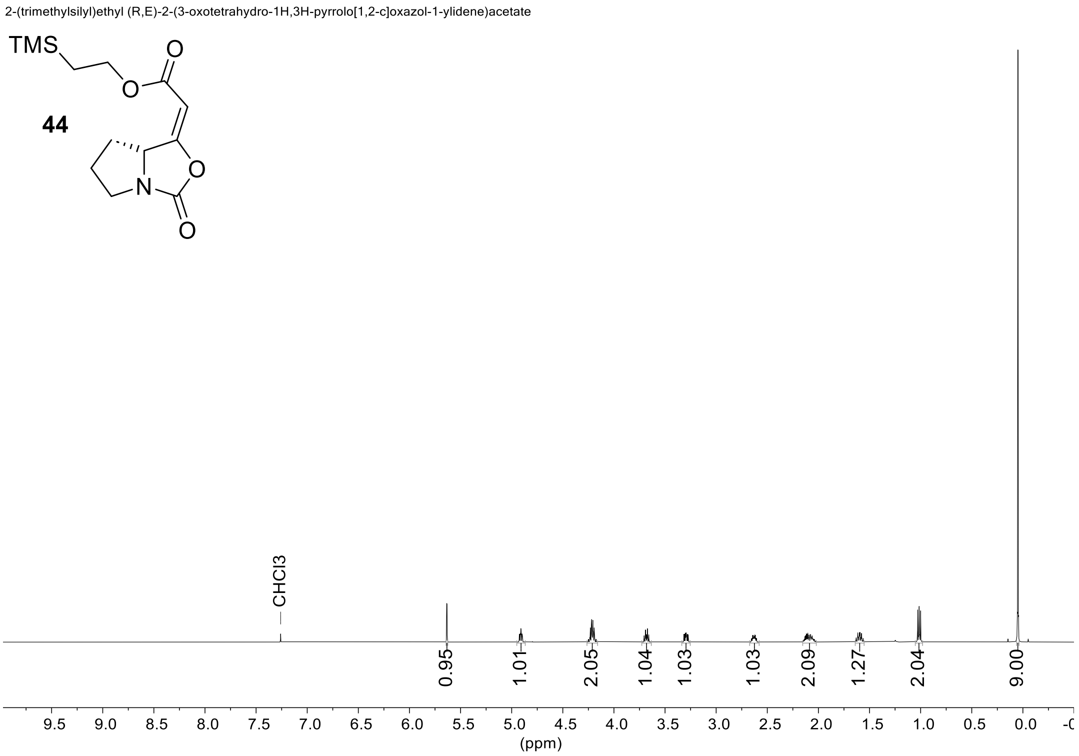

Figure S278. ^{1}H-NMR spectrum (600 MHz) of 2-(trimethylsilyl)ethyl (*R*,*E*)-2-(3-oxotetrahydro-1*H*,3*H*-pyrrolo[1,2-*c*]oxazol-1-ylidene)acetate (**44**).

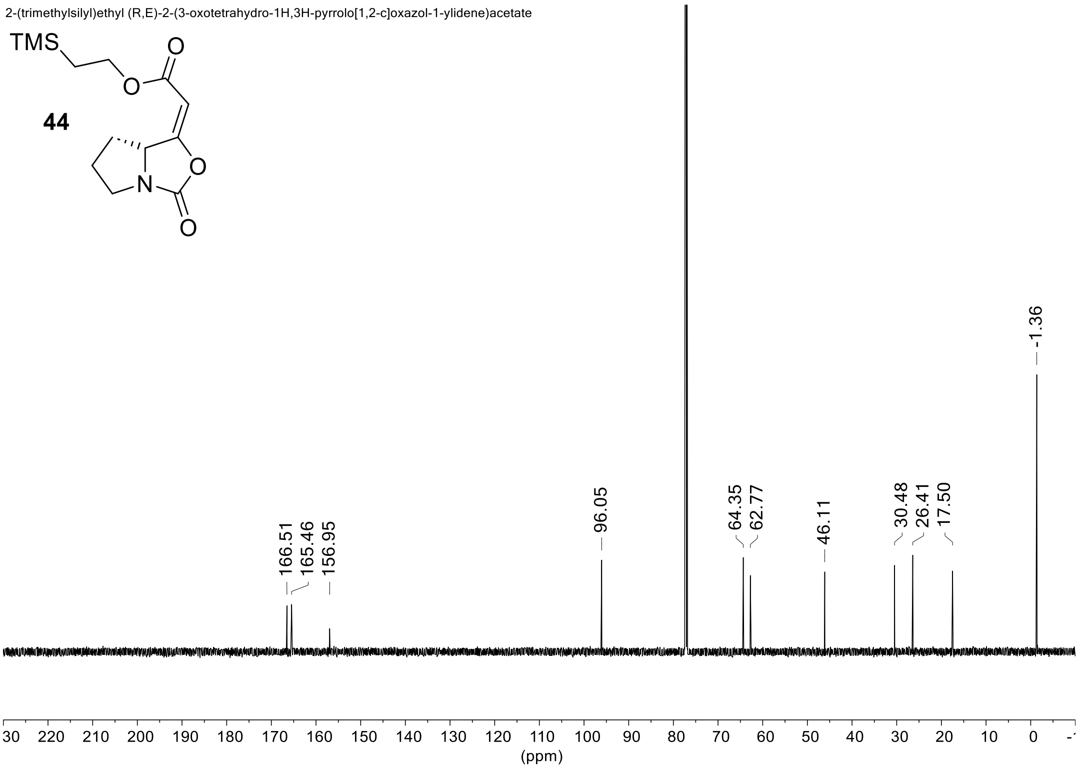

Figure S279. ^{13}C {^{1}H}-NMR spectrum (151 MHz) of 2-(trimethylsilyl)ethyl (*R*,*E*)-2-(3-oxotetrahydro-1*H*,3*H*-pyrrolo[1,2-*c*]oxazol-1-ylidene)acetate (**44**).

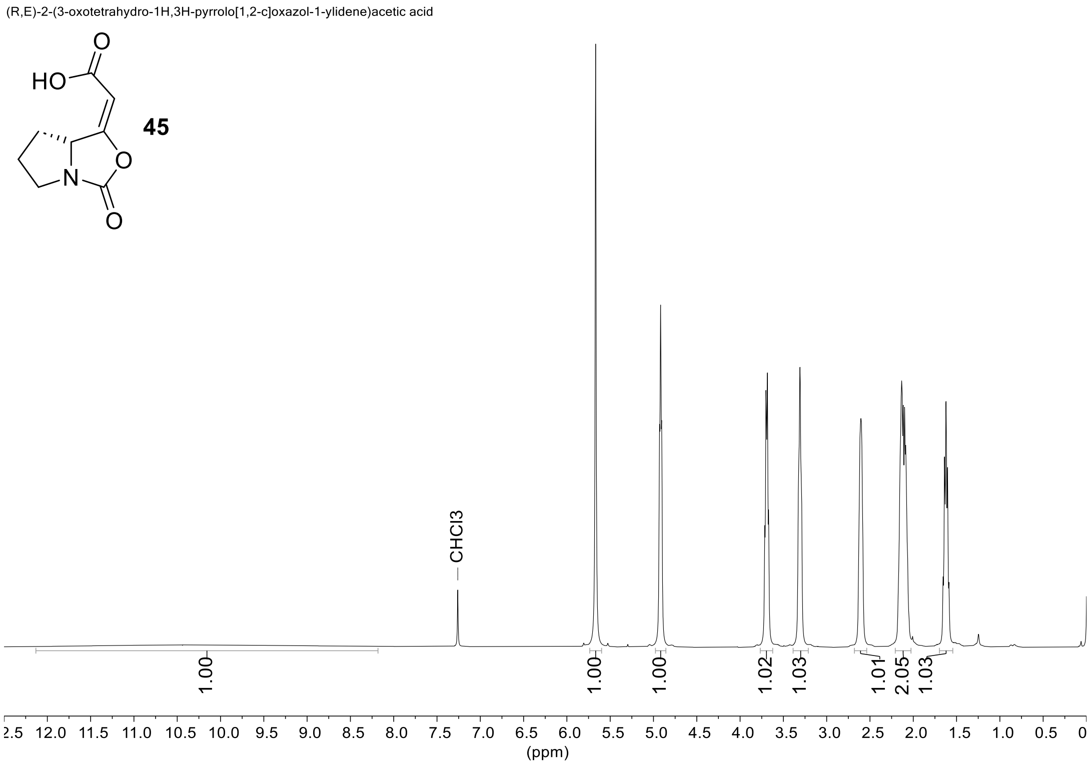

Figure S280. ^{1}H-NMR spectrum (600 MHz) of (*R*,*E*)-2-(3-oxotetrahydro-1*H*,3*H*-pyrrolo[1,2-*c*]oxazol-1-ylidene)acetic acid (**45**).

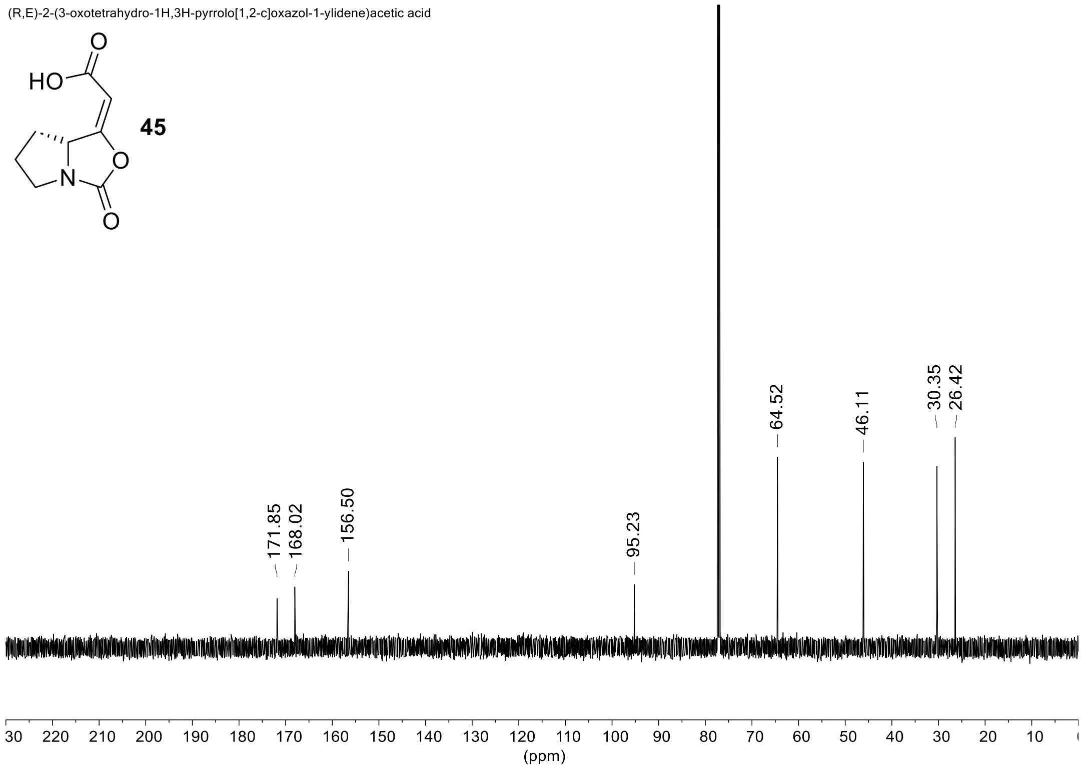

Figure S281. ^{13}C {^{1}H}-NMR spectrum (151 MHz) of (*R*,*E*)-2-(3-oxotetrahydro-1*H*,3*H*-pyrrolo[1,2-*c*]oxazol-1-ylidene)acetic acid (**45**).

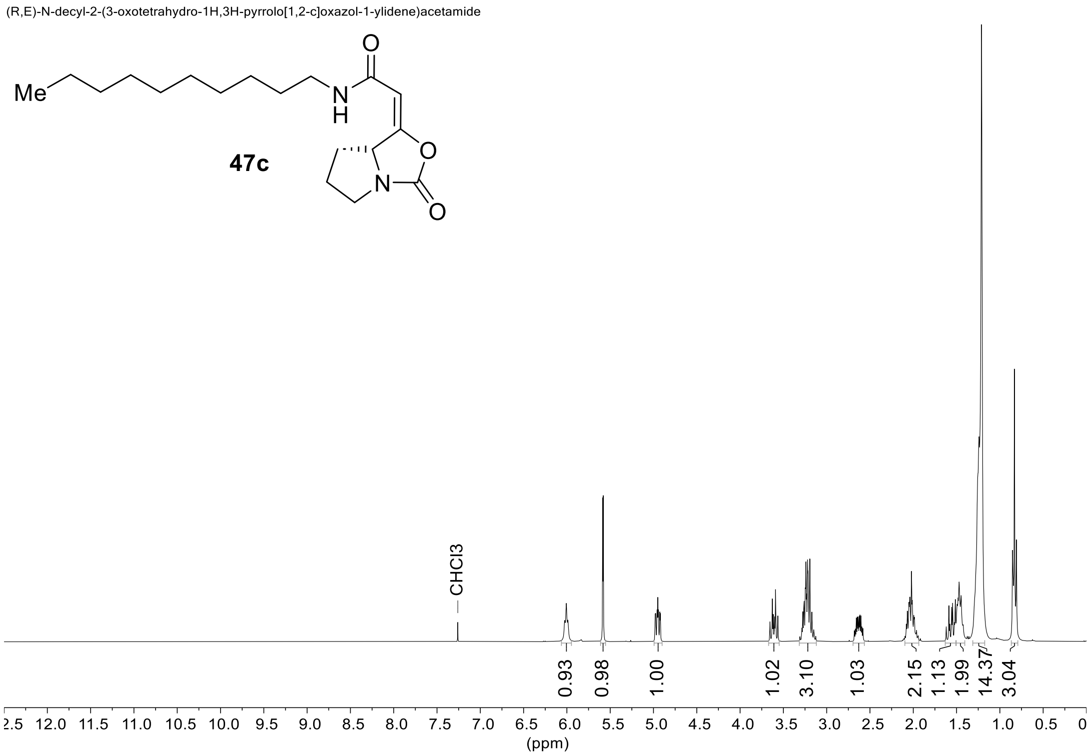

Figure S282. ^{1}H-NMR spectrum (300 MHz) of (*R*,*E*)-*N*-decyl-2-(3-oxotetrahydro-1*H*,3*H*-pyrrolo[1,2-*c*]oxazol-1-ylidene)acetamide (**47c**).

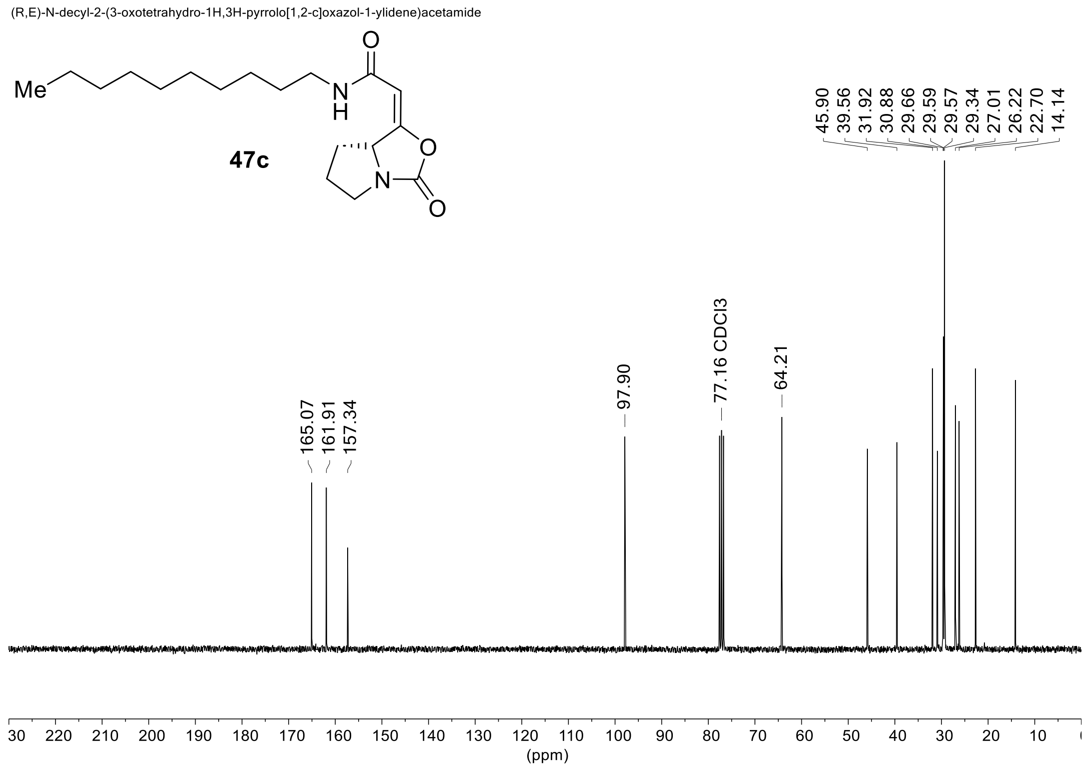

Figure S283. ^{13}C {^{1}H}-NMR spectrum (75.5 MHz) of (*R*,*E*)-*N*-decyl-2-(3-oxotetrahydro-1*H*,3*H*-pyrrolo[1,2-*c*]oxazol-1-ylidene)acetamide (**47c**).

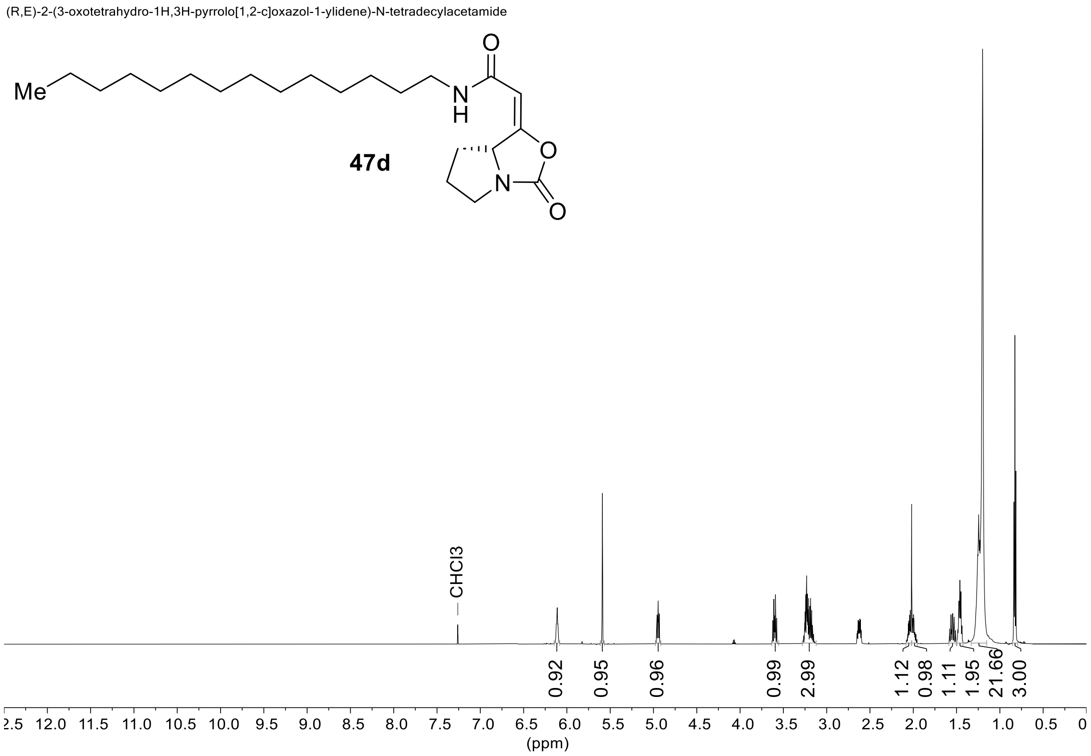

Figure S284. ^{1}H-NMR spectrum (600 MHz) of (*R*,*E*)-2-(3-oxotetrahydro-1*H*,3*H*-pyrrolo[1,2-*c*]oxazol-1-ylidene)-*N*-tetradecylacetamide (**47d**).

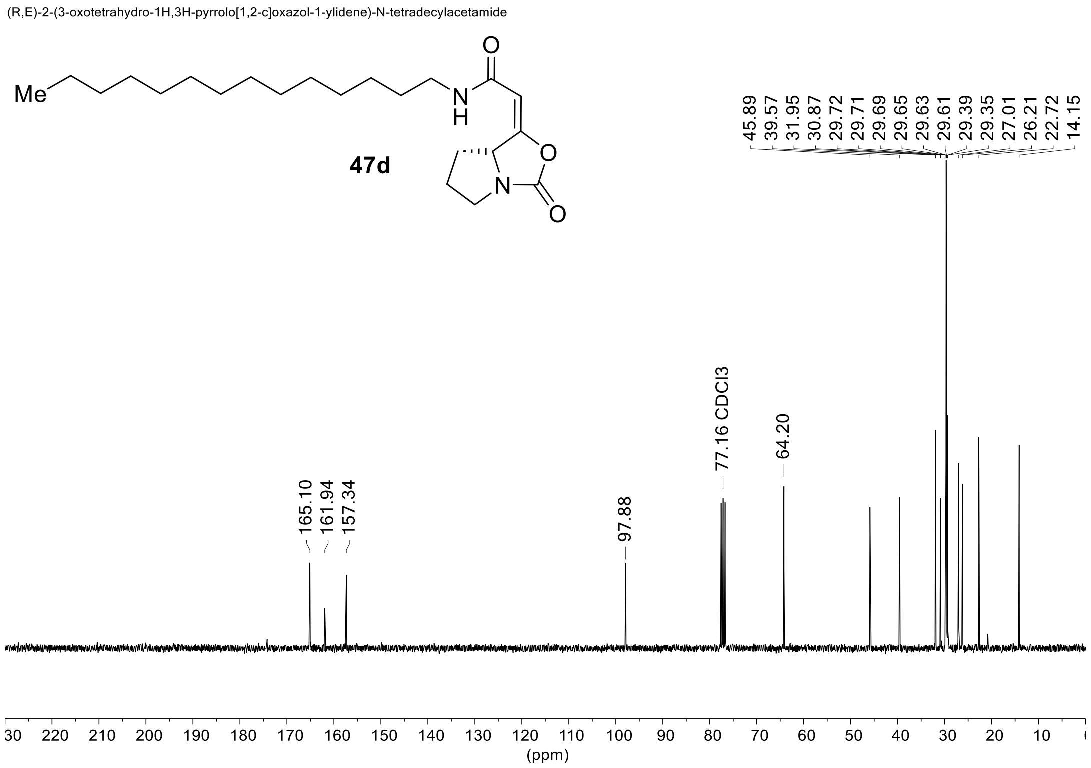

Figure S285. ^{13}C {^{1}H}-NMR spectrum (151 MHz) of (*R*,*E*)-2-(3-oxotetrahydro-1*H*,3*H*-pyrrolo[1,2-*c*]oxazol-1-ylidene)-*N*-tetradecylacetamide (**47d**).

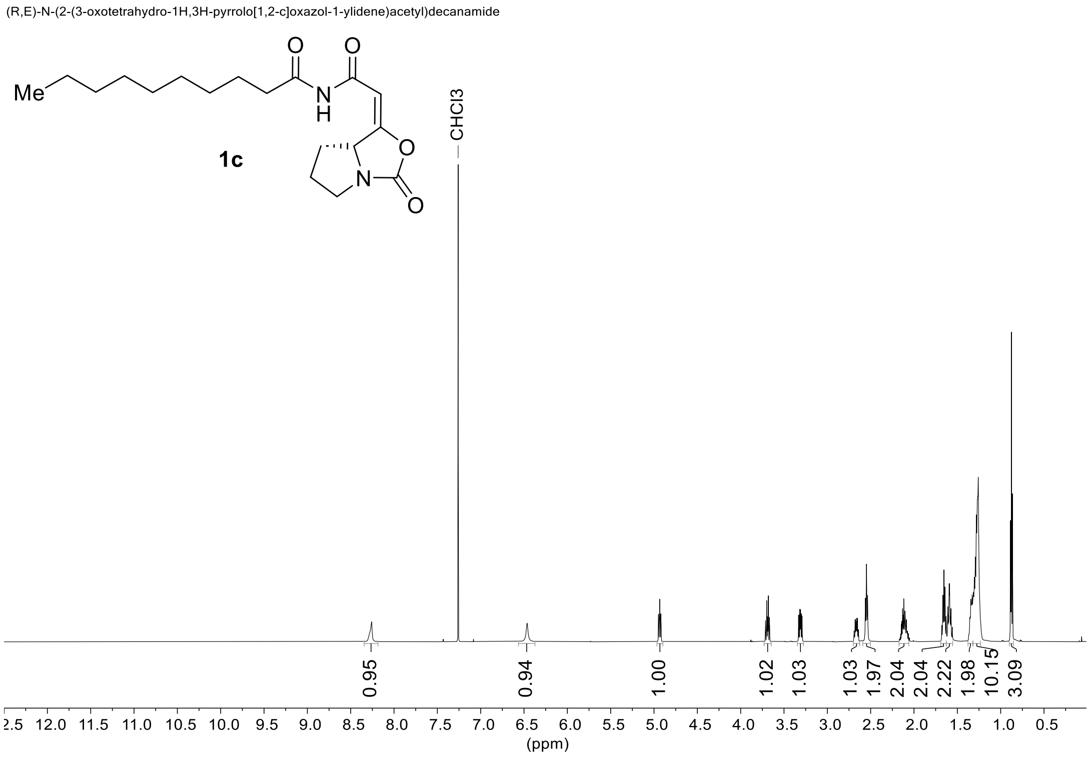

Figure S286. ^{1}H-NMR spectrum (600 MHz) of (*R*,*E*)-*N*-(2-(3-oxotetrahydro-1*H*,3*H*-pyrrolo[1,2-*c*]oxazol-1-ylidene)acetyl)decanamide (**1c**).

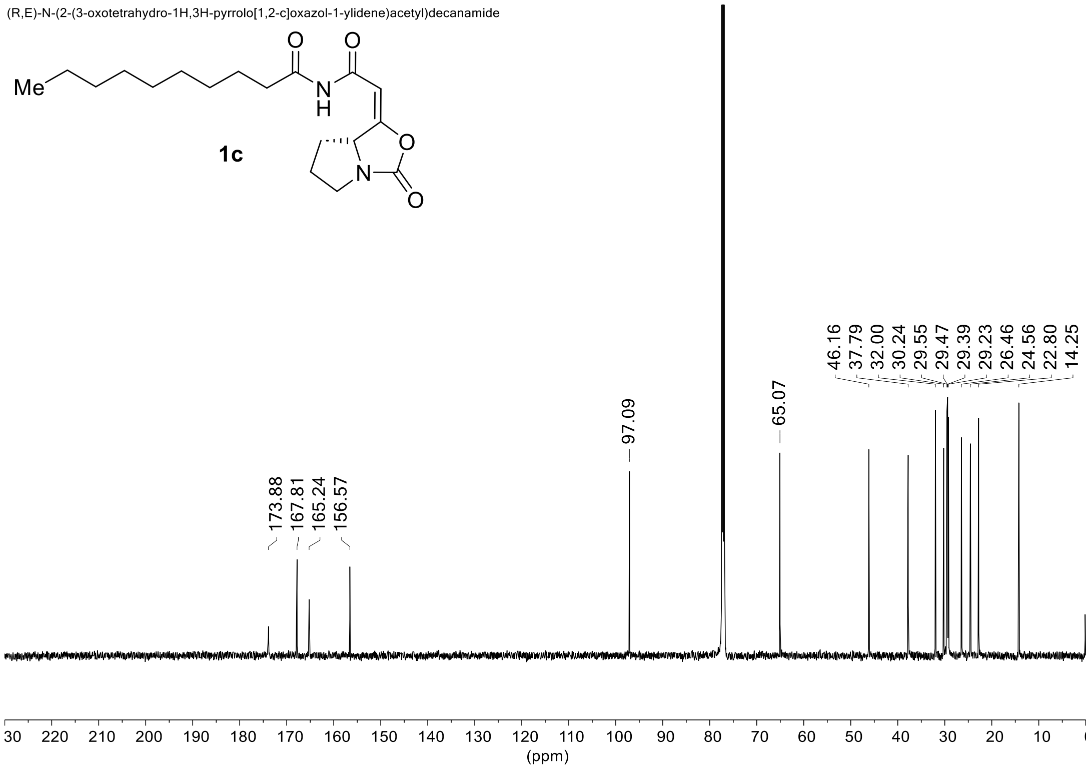

Figure S287. ^{13}C {^{1}H}-NMR spectrum (151 MHz) of (*R*,*E*)-*N*-(2-(3-oxotetrahydro-1*H*,3*H*-pyrrolo[1,2-*c*]oxazol-1-ylidene)acetyl)decanamide (**1c**).

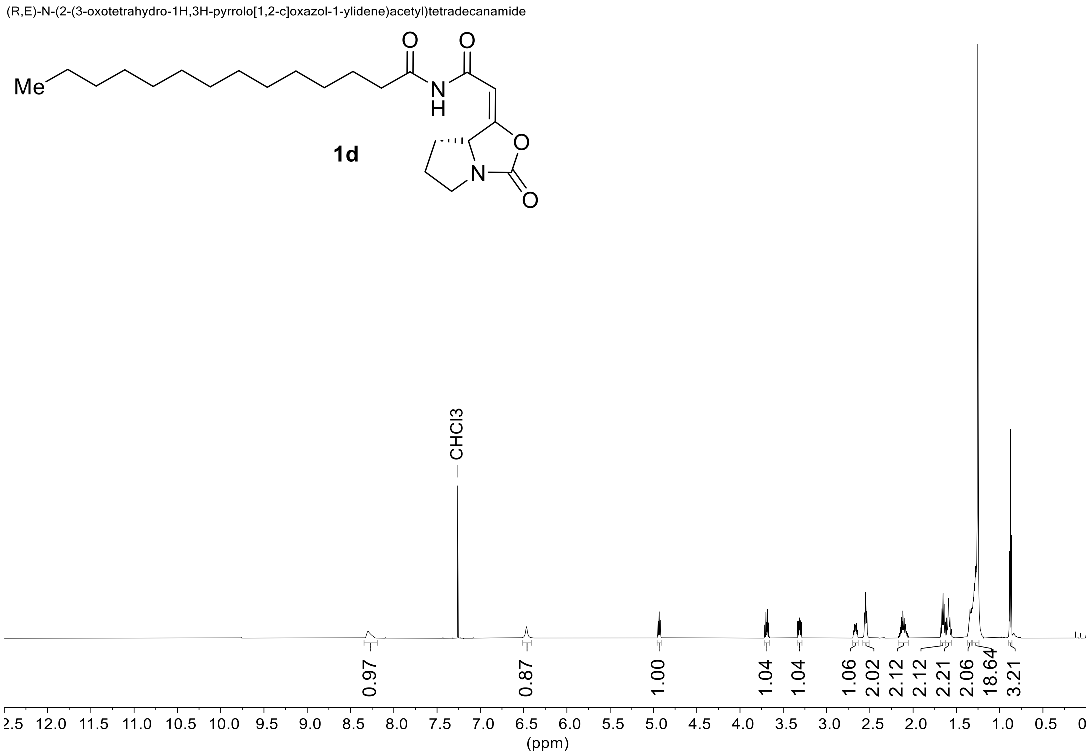

Figure S288. ^{1}H-NMR spectrum (600 MHz) of (*R*,*E*)-*N*-(2-(3-oxotetrahydro-1*H*,3*H*-pyrrolo[1,2-*c*]oxazol-1-ylidene)acetyl)tetradecanamide (**1d**).

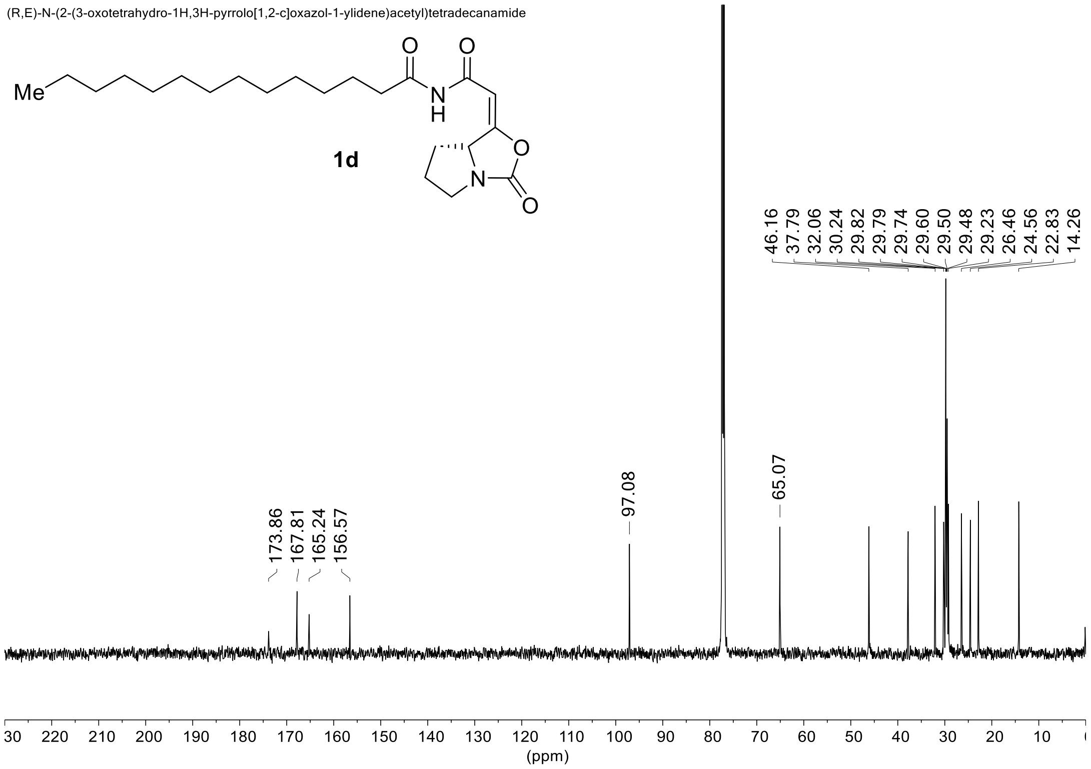

Figure S289. ^{13}C {^{1}H}-NMR spectrum (151 MHz) of (*R*,*E*)-*N*-(2-(3-oxotetrahydro-1*H*,3*H*-pyrrolo[1,2-*c*]oxazol-1-ylidene)acetyl)tetradecanamide (**1d**).

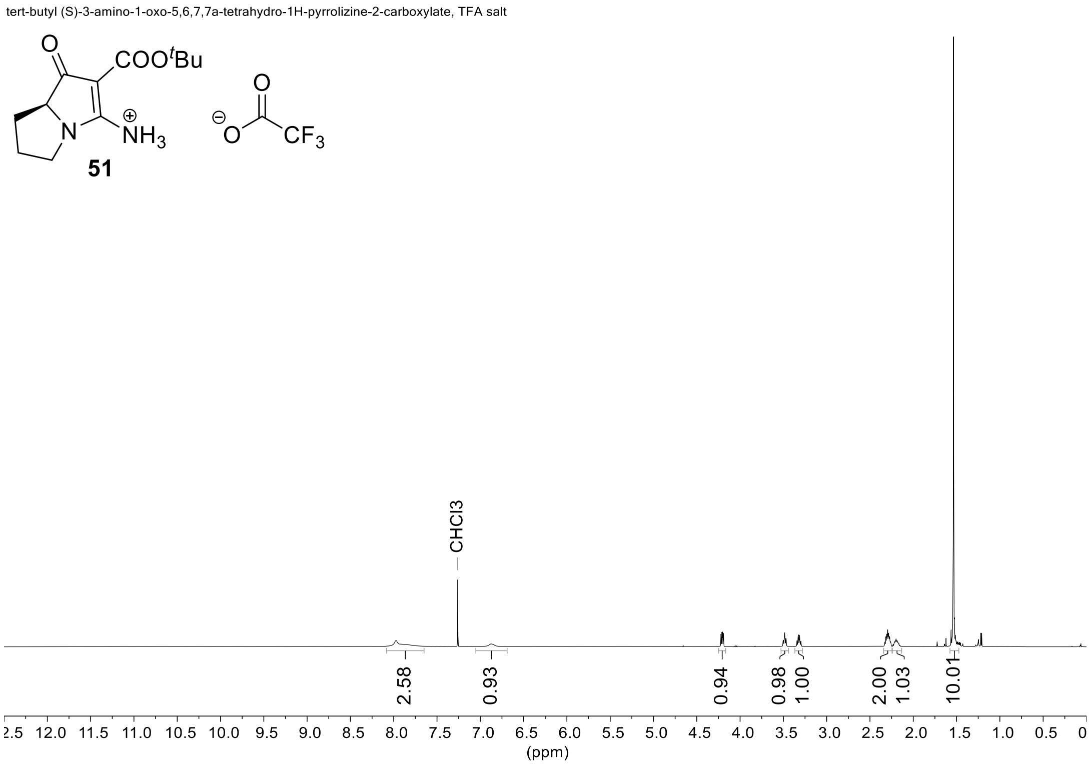

Figure S290. ^{1}H-NMR spectrum (600 MHz) of *tert*-butyl (*S*)-3-amino-1-oxo-5,6,7,7a-tetrahydro-1*H*-pyrrolizine-2-carboxylate (**51**, TFA salt).

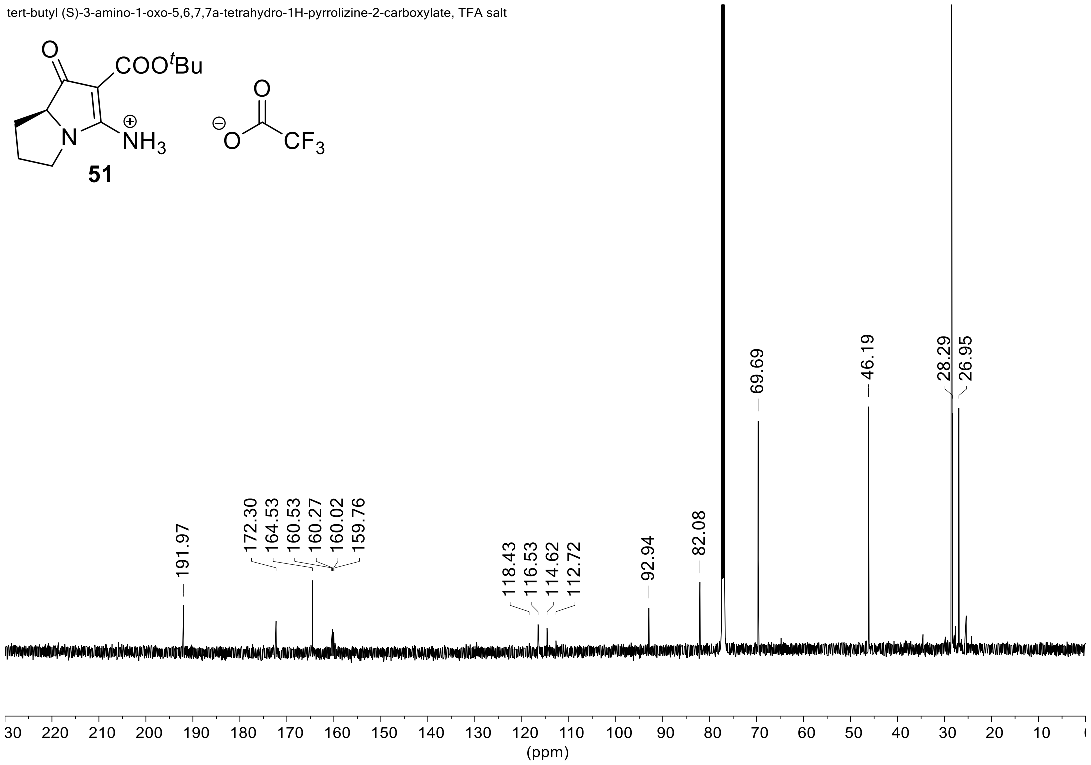

Figure S291. ^{13}C {^{1}H}-NMR spectrum (151 MHz) of *tert*-butyl (*S*)-3-amino-1-oxo-5,6,7,7a-tetrahydro-1*H*-pyrrolizine-2-carboxylate (**51**, TFA salt).

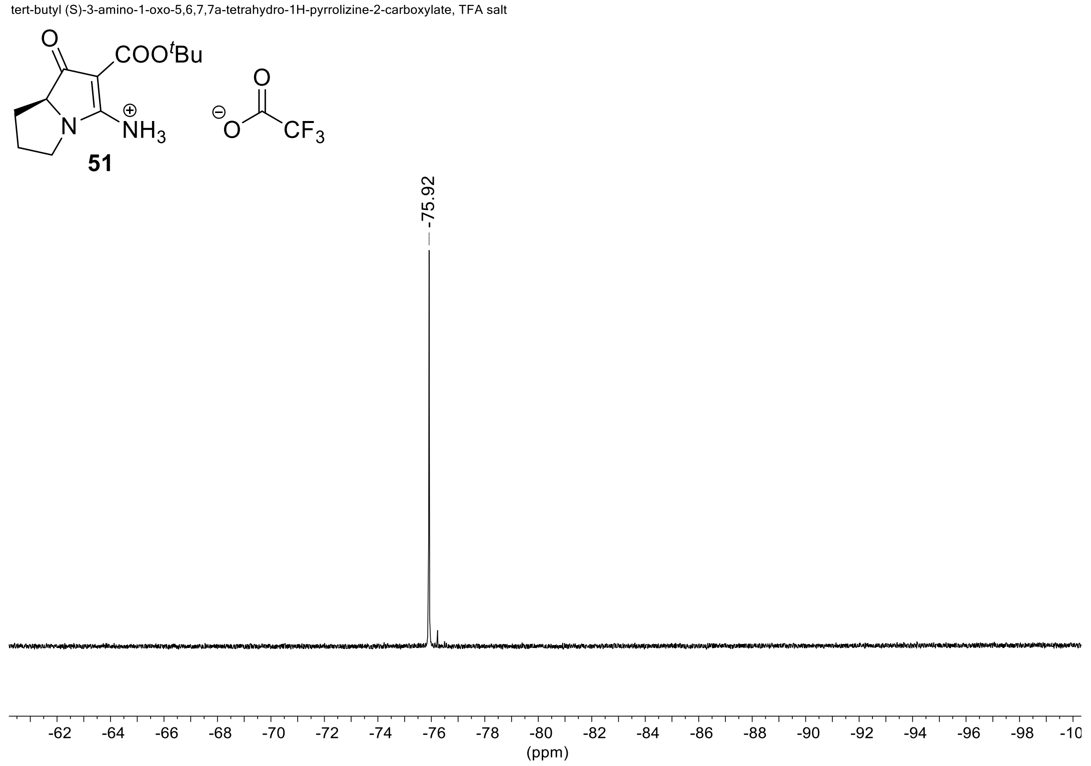

Figure S292. ^{19}F {^{1}H}-NMR spectrum (282 MHz) of *tert*-butyl (*S*)-3-amino-1-oxo-5,6,7,7a-tetrahydro-1*H*-pyrrolizine-2-carboxylate (**51**, TFA salt).

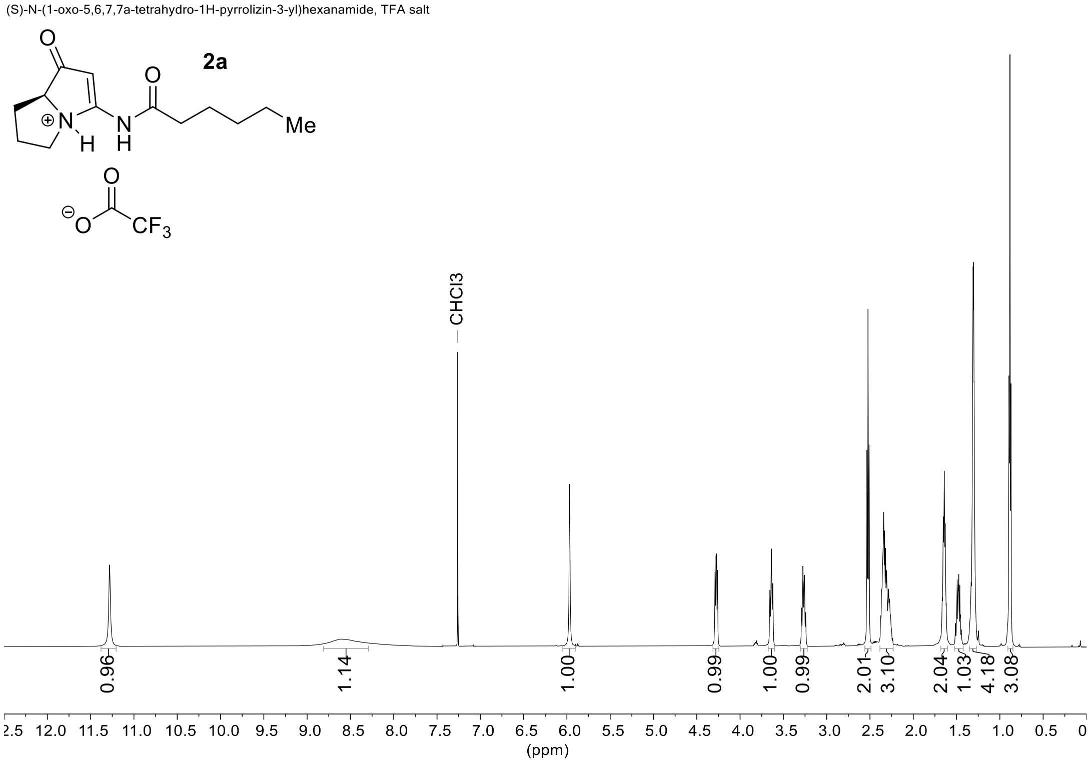

Figure S293. ^{1}H-NMR spectrum (600 MHz) of (*S*)-*N*-(1-oxo-5,6,7,7a-tetrahydro-1H-pyrrolizin-3-yl)hexanamide (**2a**, TFA salt).

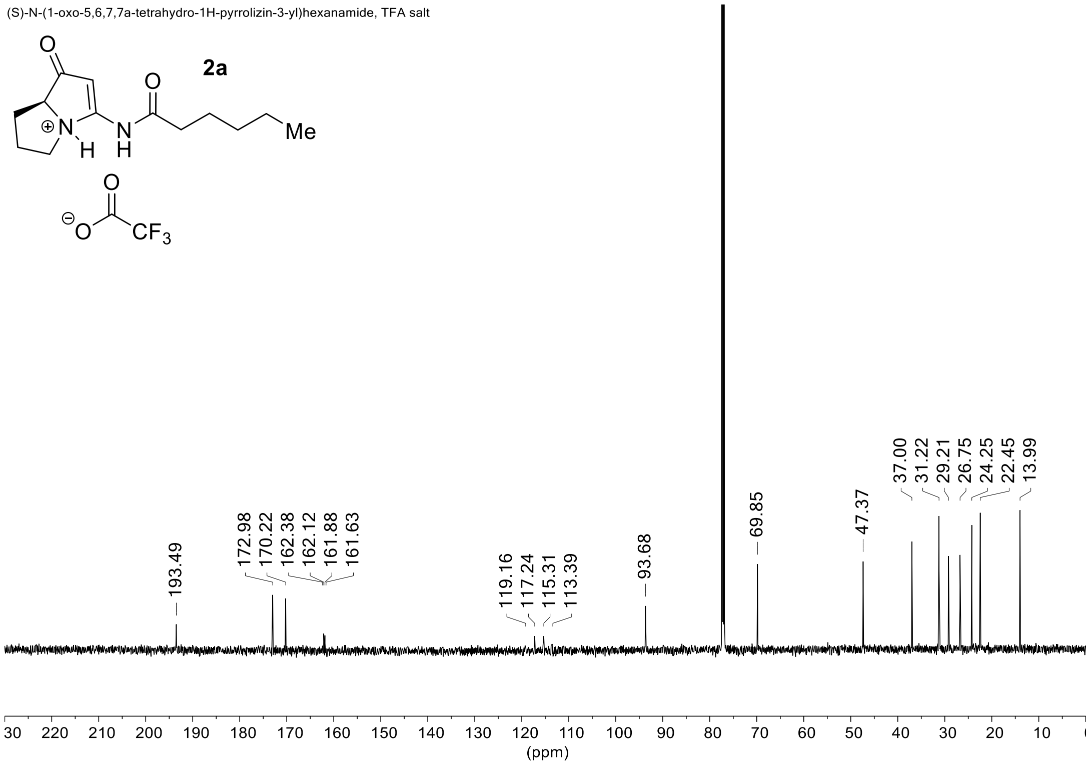

Figure S294. ^{13}C {^{1}H}-NMR spectrum (151 MHz) of (*S*)-*N*-(1-oxo-5,6,7,7a-tetrahydro-1H-pyrrolizin-3-yl)hexanamide (**2a**, TFA salt).

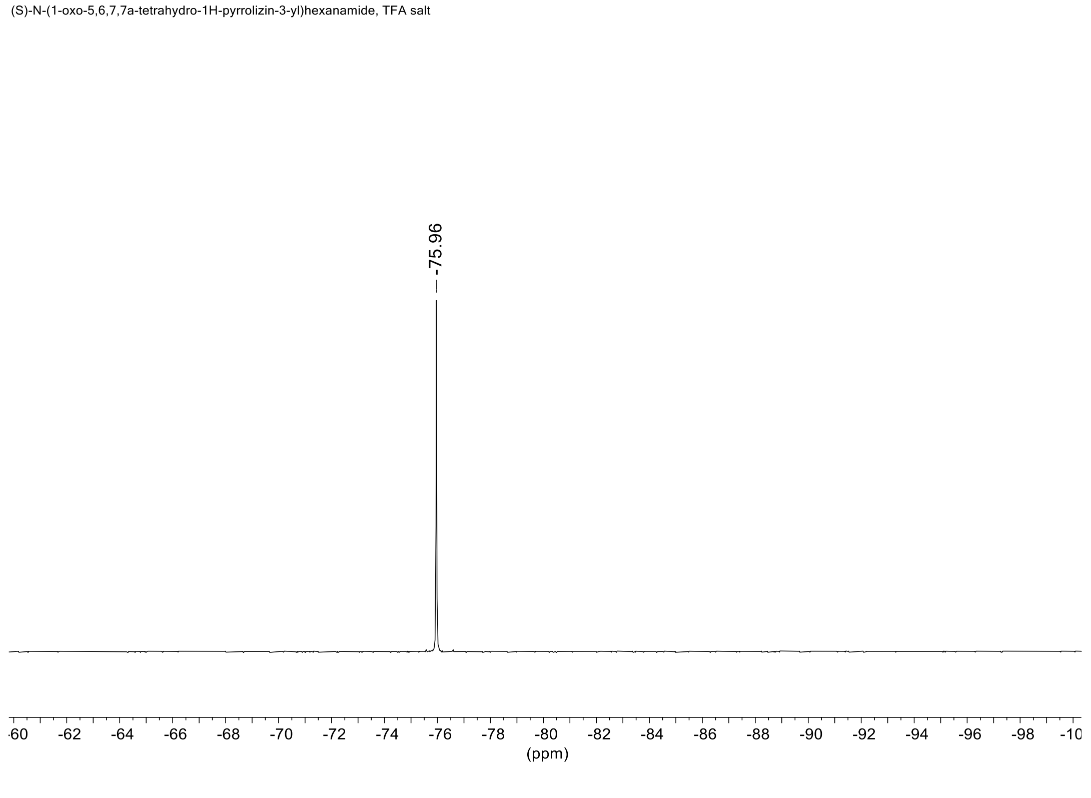

Figure S295. ^{19}F {^{1}H}-NMR spectrum (282 MHz) of (*S*)-*N*-(1-oxo-5,6,7,7a-tetrahydro-1H-pyrrolizin-3-yl)hexanamide (**2a**, TFA salt).

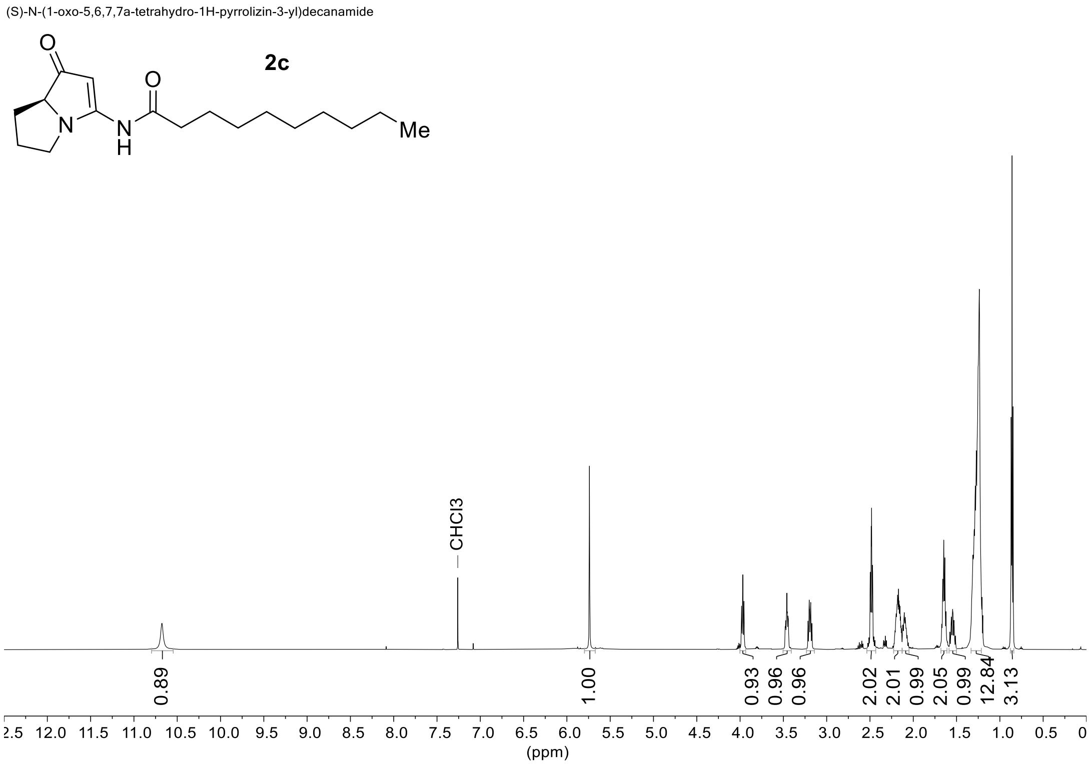

Figure S296. ^{1}H-NMR spectrum (600 MHz) of (*S*)-*N*-(1-oxo-5,6,7,7a-tetrahydro-1H-pyrrolizin-3-yl)decanamide (**2c**).

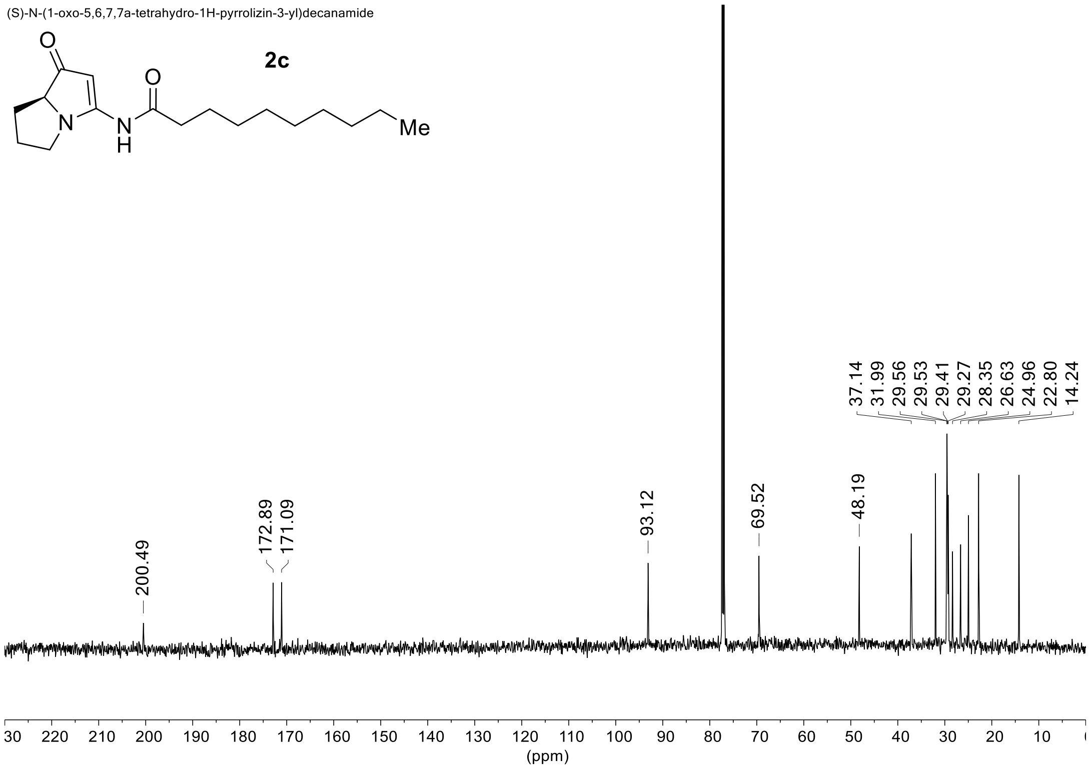

Figure S297. ^{13}C {^{1}H}-NMR spectrum (151 MHz) of (*S*)-*N*-(1-oxo-5,6,7,7a-tetrahydro-1H-pyrrolizin-3-yl)decanamide (**2c**).

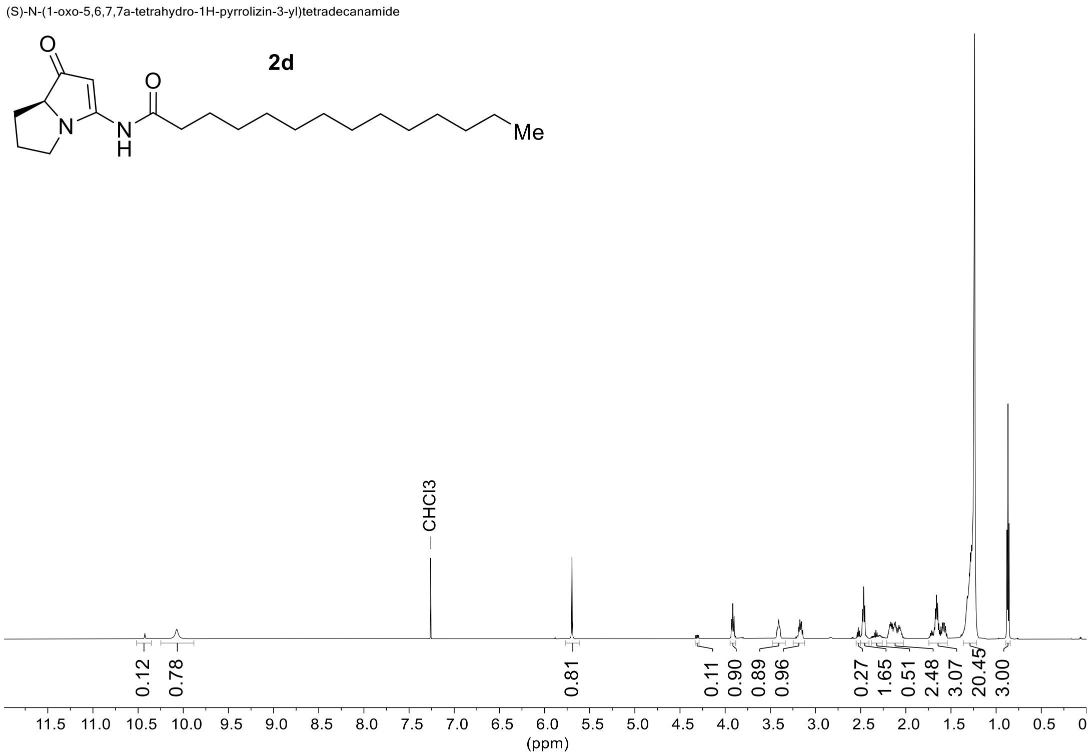

Figure S298. ^{1}H-NMR spectrum (600 MHz) of (*S*)-*N*-(1-oxo-5,6,7,7a-tetrahydro-1H-pyrrolizin-3-yl)tetradecanamide (**2d**). Minor peaks arise from TFA salt of **2d**.

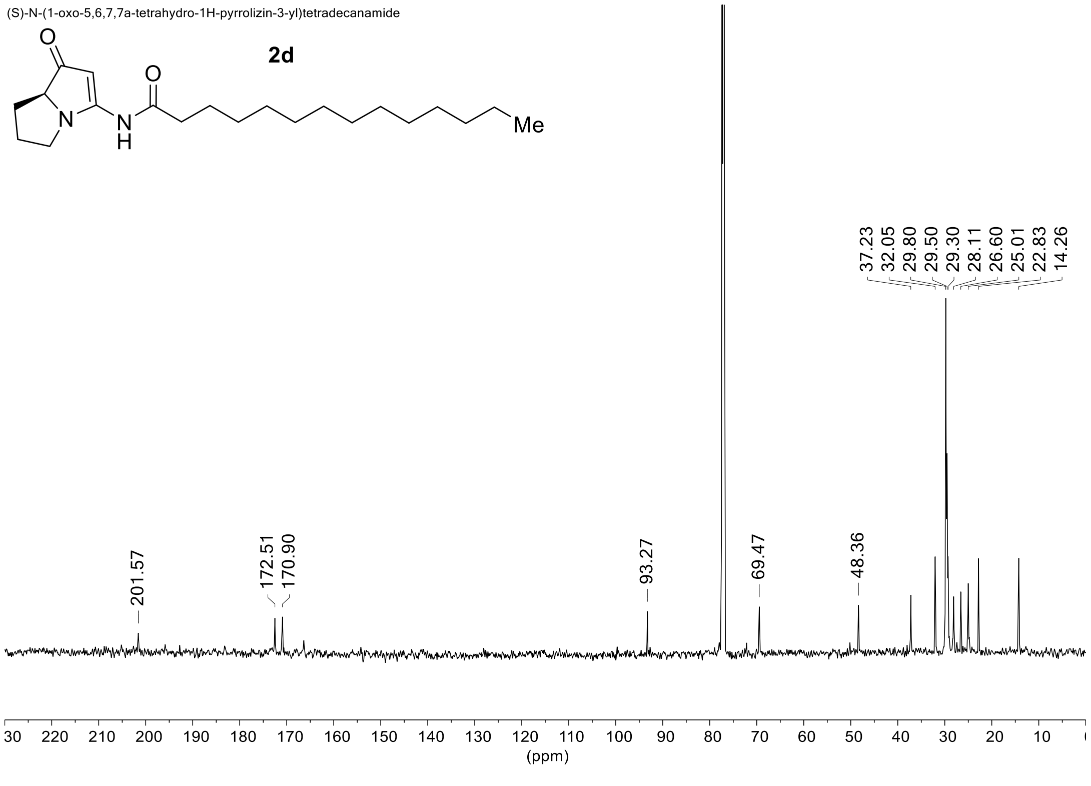

Figure S299. ^{13}C {^{1}H}-NMR spectrum (151 MHz) of (*S*)-*N*-(1-oxo-5,6,7,7a-tetrahydro-1H-pyrrolizin-3-yl)tetradecanamide (**2d**).

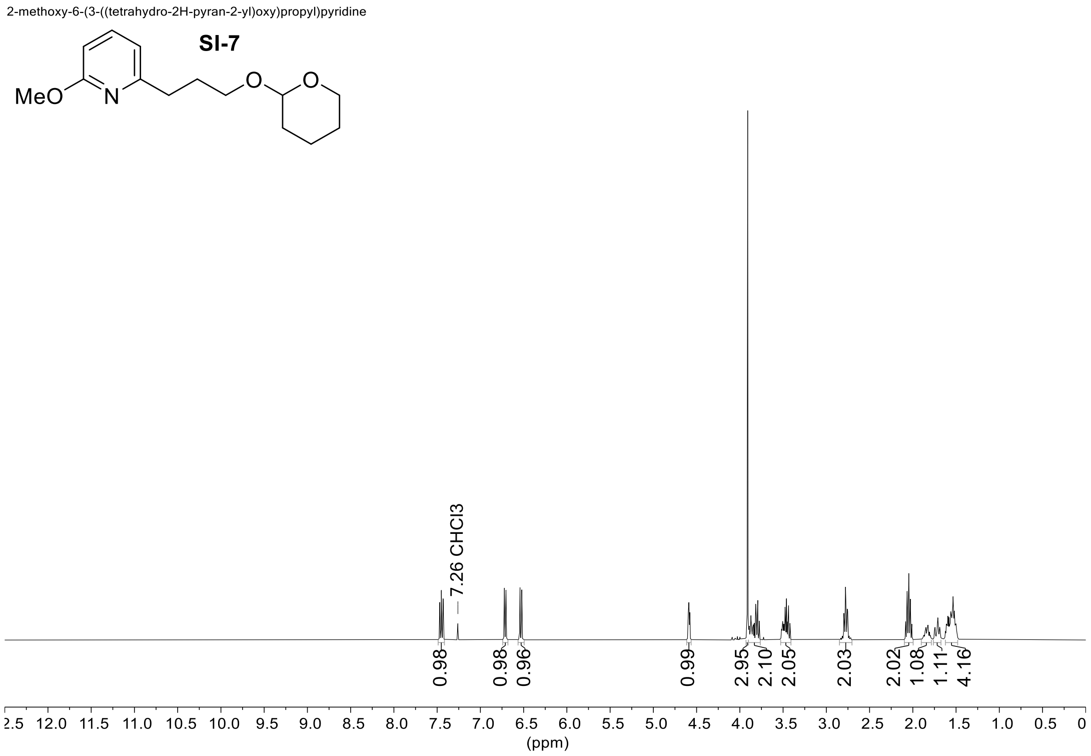

Figure S300. ^{1}H-NMR spectrum (400 MHz) of 2-methoxy-6-(3-((tetrahydro-2*H*-pyran-2-yl)oxy)propyl)pyridine (**SI-7**).

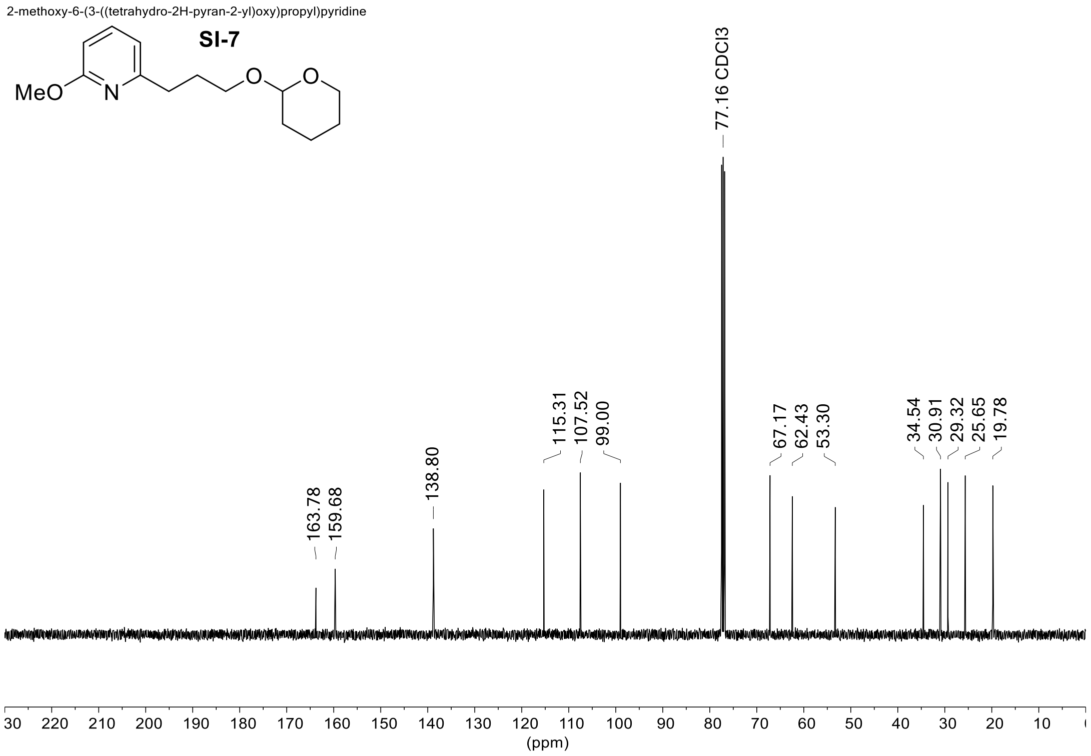

Figure S301. ^{13}C {^{1}H}-NMR spectrum (100.6 MHz) of 2-methoxy-6-(3-((tetrahydro-2*H*-pyran-2-yl)oxy)propyl)pyridine (**SI-7**).

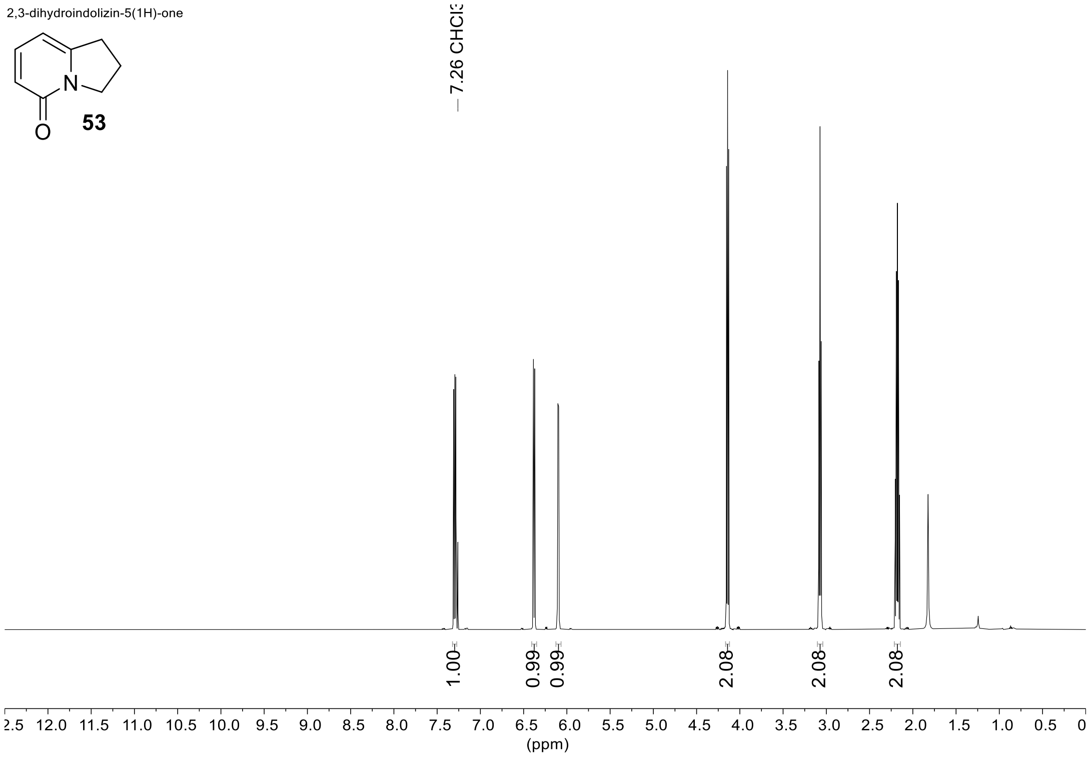

Figure S302. ^{1}H-NMR spectrum (600 MHz) of 2,3-dihydroindolizin-5(1*H*)-one (**53**).

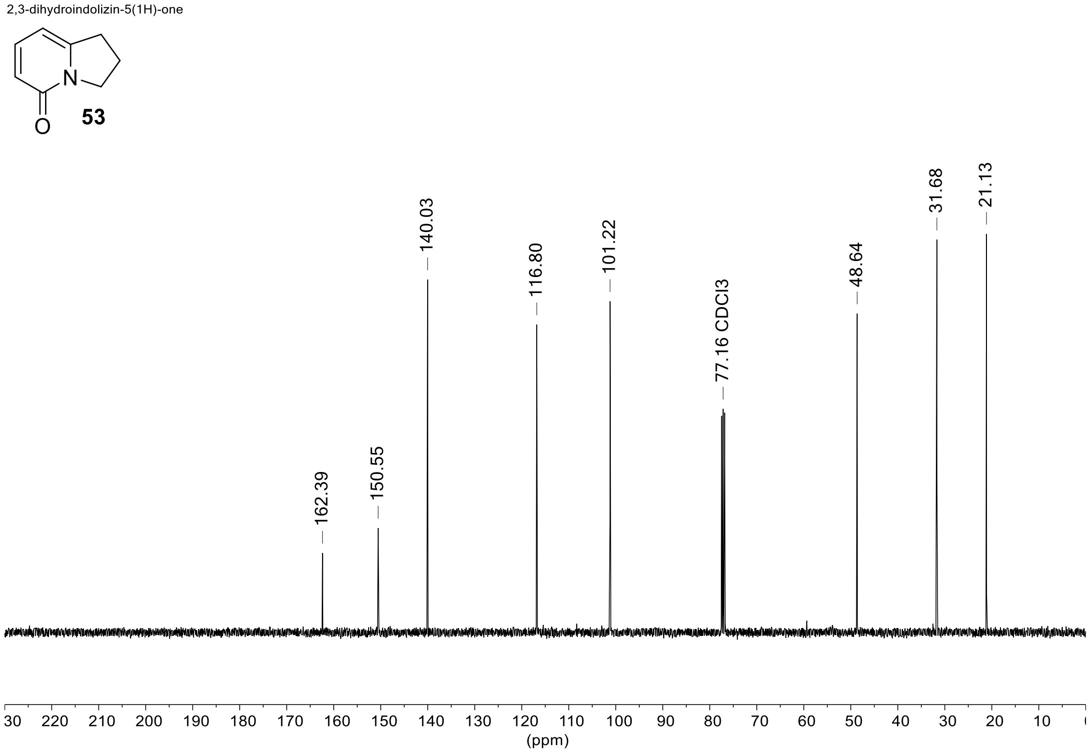

Figure S303. ^{13}C {^{1}H}-NMR spectrum (100.6 MHz) of 2,3-dihydroindolizin-5(1*H*)-one (**53**).

7. List of Abbreviations

Table S11. List of used abbreviations in this work.

Ace	acetone
BHMPO	*N*,*N′*-bis(4-hydroxy-2,6-dimethylphenyl)oxalamide
BINAP	2,2′-bis(diphenylphosphino)-1,1′-binaphthyl
BPC	base peak chromatogram
brsm	based on re-isolated starting material
BVMO	Baeyer-Villiger-monooxygenase
BVO	Baeyer-Villiger oxidation
Bz	benzoyl
CID	collision-induced dissociation
DMAP	4-(dimethylamino)pyridine
EDC HCl	1-ethyl-3-(3-dimethylaminopropyl)carbodiimide hydrochloride
EIC	extracted ion chromatogram
ESI	electrospray ionization
FA	formic acid
HATU	hexafluorophosphate azabenzotriazole tetramethyl uronium
HB	hydrogen bond
IPTG	*iso*-propyl-β-D-thiogalactopyranoside
LCC	lipocyclocarbamate
MOM	methoxymethyl
NBS	*N*-bromosuccinimide
NIS	*N*-iodosuccinimide
NOESY	nuclear Overhauser effect spectroscopy
PA	pyrrolizidine alkaloid
PDB	protein data bank
Py	pyridine
RMSD	root mean square deviation
TBAF	tetra-*n*-butylammonium fluoride
TBS	*tert*-butyldimethylsilyl
TEMPO	tetramethylpiperidinoxyl
TFA	trifluoroacetic acid
THP	tetrahydropyranyl
TMS	trimethylsilyl
Z	benzyloxycarbonyl (Cbz)

8. Literature

1. Rosenau, C. P.; Jelier, B. J.; Gossert, A. D.; Togni, A., Exposing the Origins of Irreproducibility in Fluorine NMR Spectroscopy. *Angew. Chem. Int. Ed.* **2018,** *57*, 9528–9533.
2. Harris, R. K.; Becker, E. D.; Cabral de Menezes, S. M.; Goodfellow, R.; Granger, P., NMR nomenclature. Nuclear spin properties and conventions for chemical shifts (IUPAC Recommendations 2001). *Pure Appl. Chem.* **2001,** *73*, 1795–1818.
3. Schimming, O.; Challinor, V. L.; Tobias, N. J.; Adihou, H.; Grün, P.; Pöschel, L.; Richter, C.; Schwalbe, H.; Bode, H. B., Structure, Biosynthesis, and Occurrence of Bacterial Pyrrolizidine Alkaloids. *Angew. Chem. Int. Ed.* **2015,** *54*, 12702–12705.
4. Hicks, K. A.; O'Leary, S. E.; Begley, T. P.; Ealick, S. E., Structural and Mechanistic Studies of HpxO, a Novel Flavin Adenine Dinucleotide-Dependent Urate Oxidase from Klebsiella pneumoniae. *Biochemistry* **2013,** *52*, 477–487.
5. Madeira, F.; Pearce, M.; Tivey, A. R. N.; Basutkar, P.; Lee, J.; Edbali, O.; Madhusoodanan, N.; Kolesnikov, A.; Lopez, R., Search and sequence analysis tools services from EMBL-EBI in 2022. *Nucleic Acids Res.* **2022,** *50*, W276–W279.
6. Jumper, J.; Evans, R.; Pritzel, A.; Green, T.; Figurnov, M.; Ronneberger, O.; Tunyasuvunakool, K.; Bates, R.; Žídek, A.; Potapenko, A.; Bridgland, A.; Meyer, C.; Kohl, S. A. A.; Ballard, A. J.; Cowie, A.; Romera-Paredes, B.; Nikolov, S.; Jain, R.; Adler, J.; Back, T.; Petersen, S.; Reiman, D.; Clancy, E.; Zielinski, M.; Steinegger, M.; Pacholska, M.; Berghammer, T.; Bodenstein, S.; Silver, D.; Vinyals, O.; Senior, A. W.; Kavukcuoglu, K.; Kohli, P.; Hassabis, D., Highly accurate protein structure prediction with AlphaFold. *Nature* **2021,** *596*, 583–589.
7. Mirdita, M.; Schütze, K.; Moriwaki, Y.; Heo, L.; Ovchinnikov, S.; Steinegger, M., ColabFold: making protein folding accessible to all. *Nat. Methods* **2022,** *19*, 679–682.
8. Soehano, I.; Yang, L.; Ding, F.; Sun, H.; Low, Z. J.; Liu, X.; Liang, Z.-X., Insights into the programmed ketoreduction of partially reducing polyketide synthases: stereo- and substrate-specificity of the ketoreductase domain. *Org. Biomol. Chem.* **2014,** *12*, 8542–8549.
9. De Vleeschouwer, M.; Sinnaeve, D.; Van den Begin, J.; Coenye, T.; Martins, J. C.; Madder, A., Rapid Total Synthesis of Cyclic Lipodepsipeptides as a Premise to Investigate their Self-Assembly and Biological Activity. *Chem. Eur. J.* **2014,** *20*, 7766–7775.
10. Vudhgiri, S.; Routhu, S. R.; Kumar, C. G.; Prasad, R. B. N.; Reddy Jala, R. C., Design, synthesis, and cytotoxicity evaluation of threonine-based galactoceramide with aromatic groups and various fatty-acyl side chains. *Med. Chem. Res.* **2018,** *27*, 285–307.
11. Grab, H. A.; Kirsch, V. C.; Sieber, S. A.; Bach, T., Total Synthesis of the Cyclic Depsipeptide Vioprolide D via its (Z)-Diastereoisomer. *Angew. Chem. Int. Ed.* **2020,** *59*, 12357–12361.
12. Niida, A.; Tanigaki, H.; Inokuchi, E.; Sasaki, Y.; Oishi, S.; Ohno, H.; Tamamura, H.; Wang, Z.; Peiper, S. C.; Kitaura, K.; Otaka, A.; Fujii, N., Stereoselective Synthesis of 3,6-Disubstituted-3,6-dihydropyridin-2-ones as Potential Diketopiperazine Mimetics Using Organocopper-Mediated *anti*-S_N2' Reactions and Their Use in the Preparation of Low-Molecule CXCR4 Antagonists. *J. Org. Chem.* **2006,** *71*, 3942–3951.
13. Zhang, H.; Sridhar Reddy, M.; Phoenix, S.; Deslongchamps, P., Total Synthesis of Ouabagenin and Ouabain. *Angew. Chem. Int. Ed.* **2008,** *47*, 1272–1275.
14. Záborský, O.; Petrovičová, Ľ.; Doháňošová, J.; Moncol, J.; Fischer, R., Simple and efficient synthesis of bicyclic enol-carbamates: access to brabantamides and their analogues. *RSC Adv.* **2020,** *10*, 6790–6793.
15. Nicolaou, K. C.; Mathison, C. J. N., Synthesis of Imides, N-Acyl Vinylogous Carbamates and Ureas, and Nitriles by Oxidation of Amides and Amines with Dess–Martin Periodinane. *Angew. Chem. Int. Ed.* **2005,** *44*, 5992–5997.
16. Duvall, J. R.; Wu, F.; Snider, B. B., Structure Reassignment and Synthesis of Jenamidines A_1/A_2, Synthesis of (+)-NP25302, and Formal Synthesis of SB-311009 Analogues. *J. Org. Chem.* **2006,** *71*, 8579–8590.
17. Knoepfel, T.; Nimsgern, P.; Jacquier, S.; Bourrel, M.; Vangrevelinghe, E.; Glatthar, R.; Behnke, D.; Alper, P. B.; Michellys, P.-Y.; Deane, J.; Junt, T.; Zipfel, G.; Limonta, S.; Hawtin, S.; Andre, C.; Boulay, T.; Loetscher, P.; Faller, M.; Blank, J.; Feifel, R.; Betschart, C., Target-Based Identification and Optimization of 5-Indazol-5-yl Pyridones as Toll-like Receptor 7 and 8 Antagonists Using a Biochemical TLR8 Antagonist Competition Assay. *J. Med. Chem.* **2020,** *63*, 8276–8295.
18. Padwa, A.; Sheehan, S. M.; Straub, C. S., An Isomünchnone-Based Method for the Synthesis of Highly Substituted 2(1*H*)-Pyridones. *J. Org. Chem.* **1999,** *64*, 8648–8659.
19. Bothner-By, A. A., Geminal and Vicinal Proton-Proton Coupling Constants in Organic Compounds. In *Advances in Magnetic and Optical Resonance*, Waugh, J. S., Ed. Academic Press: 1965; Vol. 1, 195–316.
20. Minch, M. J., Orientational dependence of vicinal proton-proton NMR coupling constants: The Karplus relationship. *Concepts Magn. Reson.* **1994,** *6*, 41–56.
